# Comprehensive analysis of bHLH transcription factors reveals candidate regulators of flower development and heat stress response in* Rhododendron simsii*

**DOI:** 10.1186/s12870-025-07868-x

**Published:** 2025-12-08

**Authors:** Cheng Wang, Shenghui Tu, Liping Zou, Xiaojing Wang, Changchun Li

**Affiliations:** 1 Hubei Key Laboratory of Resource Utilization and Quality Control of Characteristic Crops, College of Life Science and Technology, Hubei Engineering University, Xiaogan, 432000 China; 2 Hubei Province Research Center of Engineering Technology for Utilization of Botanical Functional Ingredients, Xiaogan, 432000 China; 3 The Key Laboratory of Plant Resources Conservation and Germplasm Innovation in the Mountainous Region (Ministry of Education), Institute of Agro-Bioengineering, Guizhou University, Guiyang, 550025 China

**Keywords:** BHLH transcription factors, *Rhododendron simsii*, Phylogenetic analysis, Flower development, Heat stress response, Expression patterns

## Abstract

**Background:**

The basic helix–loop–helix (bHLH) transcription factors constitute one of the largest families in plants and play vital roles in growth, development, and environmental stress responses. However, their involvement in flower development and heat stress response in *Rhododendron simsii* remains unclear.

**Results:**

A total of 116 bHLH genes were identified in the *R. simsii* genome and classified into 17 groups based on phylogenetic relationships with *Arabidopsis thaliana* bHLHs. Gene duplication analysis revealed that 57 genes originated from whole-genome duplication events, and 49 from ancient segmental duplications. Promoter analysis identified abundant cis-regulatory elements associated with light, gibberellin, abscisic acid, and stress responses, suggesting regulatory roles in flower development and stress adaptation. Expression profiling showed that 23 and 13 genes were specifically expressed in vegetative organs and flowers, respectively. Analysis of five flower developmental stages grouped 49 genes into four distinct expression patterns. Under high-temperature treatment, 12 flower-expressed genes exhibited altered expression, with nine being significantly upregulated. Functional annotation indicated that two genes, *RsbHLH53* and *RsbHLH59*, are orthologous to *Arabidopsis MYC2* and *JAM2*, which are key regulators of heat stress response.

**Conclusions:**

This study provides the first comprehensive characterization of the bHLH gene family in *R. simsii* and identifies candidate genes potentially involved in flower development and thermotolerance. These findings lay a theoretical foundation for understanding the molecular mechanisms underlying heat tolerance and for future molecular breeding of heat-resistant *R. simsii* varieties.

**Supplementary Information:**

The online version contains supplementary material available at 10.1186/s12870-025-07868-x.

## Background

Transcription factors (TFs) are key regulators of eukaryotic gene expression that function by binding to specific DNA sequences [1]. Among them, the basic helix–loop–helix (bHLH) family is one of the largest in plants, playing crucial roles in growth, development, and responses to diverse environmental stresses [2–4]. bHLH proteins are characterized by a conserved ~ 60-amino-acid domain containing a basic DNA-binding region (10–15 amino acids) at the N-terminus and a helix–loop–helix (HLH) region (~ 40 amino acids) at the C-terminus. The basic region specifically recognizes the E-box motif (CANNTG), whereas the HLH region mediates protein dimerization [5, 6].

The availability of plant genomic resources has facilitated the genome-wide identification of bHLH genes in many species, including cotton, *Pyrus ussuriensis*, *Arabidopsis*, potato, apple, grape, mango, and cabbage [7–14]. Functional studies have revealed their diverse biological roles. For example, GhPAS1 regulates cotton architecture through brassinosteroid (BR) signaling [15], SlPRE2 modulates fruit development via gibberellin metabolism in tomato, [16] and DTD interacts with TDR to control tapetum and pollen development in rice [17]. In addition, bHLHs are essential for secondary metabolite biosynthesis, such as anthocyanin accumulation in kiwifruit (*Actinidia chinensis*) and orchids [18, 19]. They are also critical mediators of stress responses: MdbHLH104 improves iron-deficiency tolerance in apple by activating plasma membrane H + -ATPase activity [20], SlICE1 enhances cold tolerance in tomato [21], In *Arabidopsis*, AtbHLH92 participated in plant’s response to osmotic stresses [22], and AtbHLH17 (AtAIB) enhances drought tolerance by modulating ABA signaling in *Arabidopsis* [23].

*Rhododendron simsii* (Indian azalea) is a valuable ornamental plant of the Ericaceae family, prized for its vibrant red corolla and horticultural significance [24]. However, its growth and flowering are highly sensitive to elevated temperatures, which can shorten blooming periods, reduce bud formation, cause floral deformities, and compromise overall plant health by increasing susceptibility to root rot and pests [24]. Despite the biological and horticultural importance of *R. simsii*, the bHLH transcription factor family in this species has not been systematically investigated. Specifically, it remains unclear whether RsbHLHs regulate flower development, which members respond to high-temperature stress, and how these genes may link stress responses to floral regulation.

To address these questions, we performed a comprehensive genome-wide characterization of the *RsbHLH* gene family in *R. simsii*, including chromosomal distribution, phylogenetic classification, gene structure, and duplication patterns. We further analyzed their expression profiles in different tissues and floral developmental stages using publicly available RNA-seq data and examined their responses to high-temperature treatment. Our findings provide new insights into the molecular roles of RsbHLHs in flower development and thermotolerance, offering candidate genes for breeding heat-tolerant azalea cultivars through molecular approaches.

## Materials and methods

### Plant materials and heat stress treatment

Two-year-old *R. simsii* seedlings were selected, and they were provided by Wanlvyuan Seedlings Co. Ltd., Bijie, Guizhou Province, China. Seedlings were cultivated in a growth chamber under a 12 h light (22 °C)/12 h dark (18 °C) cycle, 70% relative humidity, and 4000 lx light intensity. Twelve uniformly growing seedlings were subjected to heat stress by transferring them to an incubator maintained at 40 ± 0.5 °C for 3 or 6 days. The samples collected at 3 and 6 days represent consecutive time points from independent experimental groups. Leaves were collected immediately, frozen in liquid nitrogen, and stored at − 80 °C for subsequent RNA extraction and RT-qPCR analysis.

### Identification of RsbHLH genes

Genome sequences and annotation files of *R. simsii*, *R. irroratum*, and *R. williamsianum* were obtained from the TEGR database (http://www.tegr.com.cn). Candidate *bHLH* genes were identified by searching the Pfam database (PF00010; e-value < 1e − 4) [25], proteins containing a conserved bHLH domain were designated as RsbHLH, RibHLH, and RwbHLH according to their respective *Rhododendron* species. *Arabidopsis* bHLH protein sequences were downloaded from PlantTFDB (http://planttfdb.gao-lab.org/). Candidate proteins were further verified for the presence of the bHLH domain using Pfam, CDD, and SMART databases [26, 27]. The physicochemical properties of confirmed proteins were calculated using ExPASy-ProtParam [26, 28]. RibHLH and RwbHLH proteins were included only in the phylogenetic tree to provide an overview of bHLH evolution across *Rhododendron* species. They were not used in expression profiling or functional analyses, as this study focused specifically on the bHLH family in *R. simsii*, for which transcriptomic and experimental data were available.

### Sequence and structural characterization

Protein characteristics were analyzed using ProtParam (https://web.expasy.org/protparam/). Subcellular localization of RsbHLHs was predicted using PSORT [29]. Gene structure information was visualized with GSDS 2.0 based on *R. simsii* genome annotation. Conserved motifs were identified using MEME (default parameters) [30].

### Phylogenetic analysis

Full-length bHLH protein sequences from *R. simsii* (RsbHLHs), *R. irroratum* (RibHLHs), *R. williamsianum* (RwbHLHs), and *Arabidopsis* (AtbHLHs) (Table S1) were aligned using MUSCLE [31]. An unrooted maximum-likelihood (ML) phylogenetic tree was constructed with IQ-TREE (1,000 bootstrap replicates) [32]. RsbHLHs were classified into groups based on sequence similarity to AtbHLHs.

### Cis-regulatory element analysis

Promoter regions (1,500 bp upstream of the ATG start codon) of each *RsbHLH* gene (Table S2) were extracted and analyzed using PlantCARE [33]. Due to the lack of experimentally validated transcription start sites (TSS), these sequences may not fully represent the true promoters or include complete 5′-UTRs.

### Gene duplication and synteny analysis

Chromosomal locations of *RsbHLHs* were visualized using MapChart based on the gene annotation of the *R. simsii* genome. Gene duplication events in *R. simsii* were detected using MCScanX [34]. Homologous gene pairs with *R. simsii* genome were first identified by BLASTp (e-value < 1e^−5^) and then used to define syntenic blocks. Non-synonymous (Ka) and synonymous (Ks) substitution rates were calculated with the PAML package (v4.10.8) [35]. Collinearity analysis among *R. simsii* and three other species was also performed using MCScanX and visualized with Dual Synteny Plot**.**

### Expression profiling

RNA-seq datasets from flower, leaf, and stem tissues and from five floral developmental stages were downloaded from NCBI SRA (accession number SRP229032) of *Rhododendron simsii*, including immature flower bud with light green surface (T1), late bud stage with pinkish tinge and gradual swelling (T2), early blooming stage with partially unfolded petals (T3), half-blooming stage with further petal unfolding and visible stamens (T4), and full blooming stage with fully open flower, plump shape and clear structures (T5). Each sample contained three biological replicates. Expression levels were quantified with RSEM using Bowtie2 (default parameters, mismatch = 0). Genes with FPKM > 1 in at least one sample were considered expressed. Differentially expressed genes (DEGs), defined as |log_2_FC|> 1 (FC, fold change) and an adjusted *P*-value of < 0.05, were screened using the DESeq (version 1.10.1). Heatmaps are commonly used to visualize the expression levels of DEGs using the MeV (v.4.9.0) software.

### RT-qPCR validation

Total RNA was reverse-transcribed using a PrimeScript RT Reagent Kit (TaKaRa, Dalian, China). RT-qPCR was performed using three biological and three technical replicates per sample. Gene-specific primers were designed for candidate *RsbHLHs*, with *EF1α*, *GAPDH*, and *RG7* as reference genes (Table S3) [36]. Relative expression levels were calculated using the 2^−ΔΔCt^ method, and Spearman’s correlation coefficients were used to assess concordance between RNA-seq and RT-qPCR data.

### Subcellular localization

The full-length coding sequences of RsbHLH053 and RsbHLH059, both highly expressed during floral development and responsive to heat stress, were used for subcellular localization analysis. These CDS sequences are provided in Table S4. The coding regions were cloned into the pCAMBIA2300-GFP vector, and the recombinant plasmids were transformed into Agrobacterium tumefaciens GV3101 for transient expression in tobacco epidermal cells. GFP fluorescence was observed 48 h after infiltration using a confocal laser scanning microscope (Leica TCS SP5-II, Wetzlar, Germany) [37], with an excitation wavelength of 488 nm and an emission filter of 500–550 nm. Z-stacks of optical sections (1024 × 1024 resolution) were acquired using Leica Application Suite X (LAS X). All images were exported as TIFF files and processed in ImageJ using a customized macro.

### Protein–protein interaction network

Orthologous relationships between *R. simsii* and *Arabidopsis* bHLHs were identified using OrthoVenn2 [38]. Protein–protein interaction networks of RsbHLHs were predicted using the STRING database (https://string-db.org/) based on Arabidopsis ortholog interaction data [39].

### Statistical analysis

Statistical analyses were performed using IBM SPSS Statistics 20 (IBM, Armonk, NY, USA). Differences were analyzed by one-way ANOVA, and mean separation was conducted using t-tests or LSD tests at *p* < 0.05.

## Results

### Identification and phylogenetic analysis of RsbHLHs

In this study, we identified and characterized 116 nonredundant RsbHLH transcription factors (TFs) in the *R. simsii* genome (Table S6). The length of RsbHLH proteins ranged from 135 amino acids (RsbHLH016) to 1,174 amino acids (RsbHLH034), with an average of 391 amino acids. Their molecular weights ranged from 14.85 kDa to 130.67 kDa, and the predicted isoelectric points (pI) varied from 4.67 to 9.51 (average: 6.65). Instability index analysis showed that 111 out of 116 RsbHLH proteins had an index greater than 40, suggesting that most of these proteins are potentially unstable. The aliphatic index ranged from 46.34 to 97.40. All RsbHLH proteins exhibited negative GRAVY values, indicating their hydrophilic nature. Subcellular localization predictions indicated that 105 RsbHLH proteins are likely localized to the nucleus (Table S6). Chromosomal mapping revealed that 111 *RsbHLH* genes were anchored to chromosomes, whereas five genes were located on unassembled scaffolds (Figure S1 and Table S6).

**Fig. 1:**
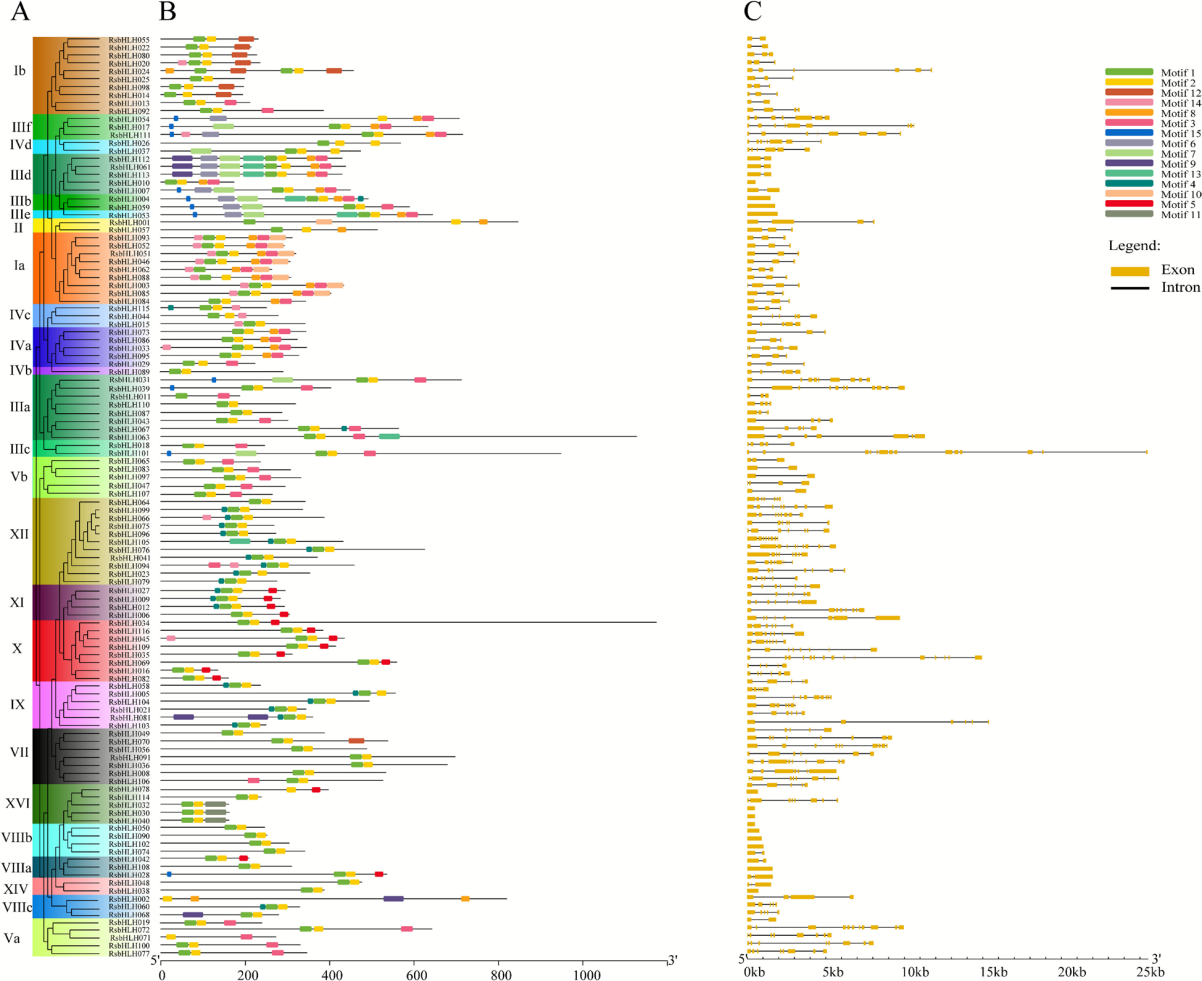
Schematic diagram of gene structure and conserved motifs of the RsbHLH family genes in *R. simsii*. **A** Phylogenetic tree of the RsbHLH family genes in *R. simsii*. **B** The distribution of conserved motifs in each RsbHLH proteins. Schematic diagram of motif distribution of RsbHLH proteins in *R. simsii* using MEME. The relative positions of each conserved motif within the RsbHLH proteins are shown in color. The black lines represent the non-conserved sequences. C Exon–intron organization of RsbHLH genes. Yellow rounded rectangles indicate exons, and black lines represent introns connecting adjacent exons

To explore their evolutionary relationships, we constructed an unrooted phylogenetic tree of bHLH proteins from *R. simsii*, *Arabidopsis thaliana*, *R. williamsianum*, and *R. irroratum* using the maximum likelihood (ML) method with 1,000 bootstrap replicates implemented in IQ-TREE. Based on tree topology and previously identified AtbHLH, the RsbHLH proteins were classified into 17 groups (Groups I–XVII) (Fig. 1 and Table S7). Group III contained the largest number of RsbHLHs (21), which were further divided into six subgroups (IIIa–IIIf). Group IV contained 11 RsbHLHs, partitioned into four subgroups (IVa–IVd), and Group VIII included 10 RsbHLHs divided into three subgroups (VIIIa–VIIIc). Further analysis revealed clusters in subgroups IIId, X, and XVI, suggesting species-specific expansions of these proteins in *R. simsii* after the divergence of core eudicots. Interestingly, some subgroups contained AtbHLHs but no bHLHs from *Rhododendron species* (e.g., Groups XVIIb, XIII, and VI), whereas Groups XIV and X contained only RsbHLHs without orthologs from other *Rhododendron* species. Similar lineage-specific loss or expansion of bHLH genes has also been observed in other plants [40–42]. These findings suggest that bHLH genes may have undergone lineage-specific gain or loss events during the evolution of *Rhododendron* and *Arabidopsis*. We further compared the physicochemical properties among different subgroups of the RsbHLH family. The results showed that the protein length and molecular weight of RsbHLHs in subgroups II, IIIe, and IIIf were significantly higher than those in other groups, suggesting that these RsbHLHs may possess more complex structures and functions, potentially forming multi-subunit protein complexes. In contrast, the predicted pI values of RsbHLHs in subgroups IX and VIIIb (pI > 8) were notably higher than those of other subgroups, indicating that these high-pI RsbHLH proteins may play important roles in nuclear import and DNA binding.

### Structure analysis of RsbHLH family genes

We next analyzed the conserved motif composition and exon–intron organization of *RsbHLH* genes (Fig. 1). Using the MEME suite, we identified 15 conserved motifs (Motifs 1–15) in RsbHLH proteins (Fig. 1A, B; Figure S2; Table S8). Motifs 1 and 2, located within the bHLH domain, were widely distributed across nearly all RsbHLH proteins. Notably, six RsbHLHs (RsbHLH002, RsbHLH011, RsbHLH054, RsbHLH062, RsbHLH071, and RsbHLH078) contained only one of these two motifs. Members within the same phylogenetic subgroup generally exhibited similar motif compositions. For instance, subgroup Ib shared three motifs (Motifs 1, 2, and 12), subgroup IIId contained nine motifs (Motifs 1, 2, 3, 6, 7, 8, 9, and 13), group XII contained three motifs (Motifs 1, 2, and 4), and group XI contained four motifs (Motifs 1, 2, 4, and 5). Some motifs were present across multiple subgroups. For example, Motif 3 was widely distributed in subgroups Va, Vb, IIIa-e, Ia, and IVa; Motif 4 was predominantly enriched in groups IX, XI, and XII; and Motif 5 was found exclusively in subgroups VIIIa, X, and XI. These patterns suggest that RsbHLHs within the same subgroup may share similar biological functions.

**Fig. 2:**
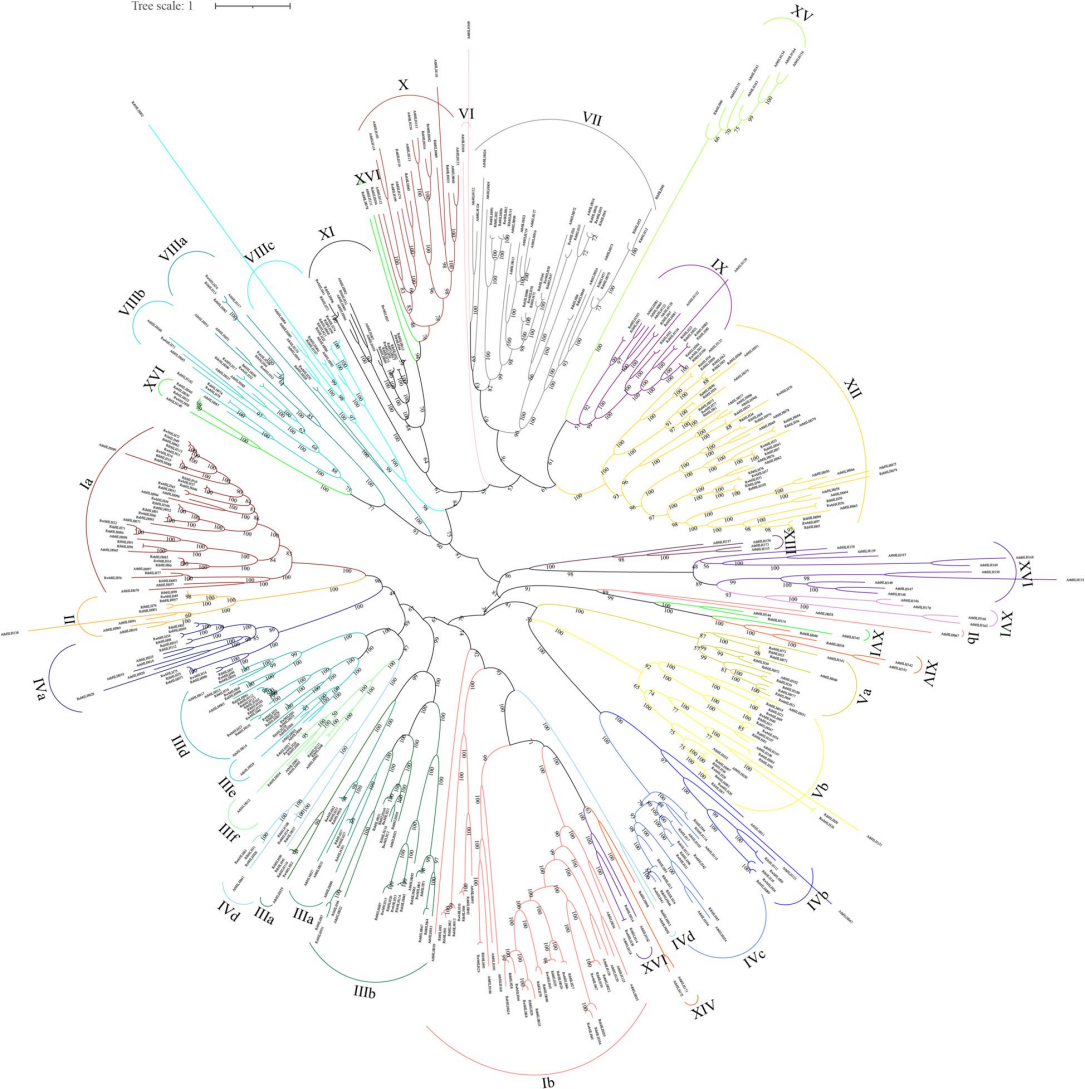
Phylogenetic analysis of bHLH family in *Arabidopsis*, *R. simsii*, *R. irroratum* and *R. williamsianum*. An unroot phylogenetic tree of bHLH family members from *Arabidopsis*, *R. simsii*, *R. irroratum* and *R. williamsianum* was constructed using ML method in IQ-TREE software with a bootstrap test (replicated 1000 times). The bHLH family members in *R. simsii*, *R. irroratum*, *R. williamsianum*, and *Arabidopsis* were marked by different symbols, respectively

The exon–intron organization of *RsbHLH* genes was visualized using the GSDS tool (Fig. 1C). The number of exons ranged from 1 to 15, with 13 *RsbHLHs* being intronless and 84 genes containing two to eight introns. *RsbHLH019* possessed the highest intron number (14 introns). Consistent with the motif results, members of the same phylogenetic subgroup generally shared similar gene structures. For example, genes in groups Va and VIII contained more than eight exons, whereas those in groups X, XI, and XII harbored five to seven introns. In contrast, genes in subgroup IIId contained only one intron, and members of group XVI were intronless. These results provide additional support for the reliability of the phylogenetic classification of *RsbHLHs* (Fig. 2).

### Cis-element profiling of RsbHLH gene promoters

**Fig. 3:**
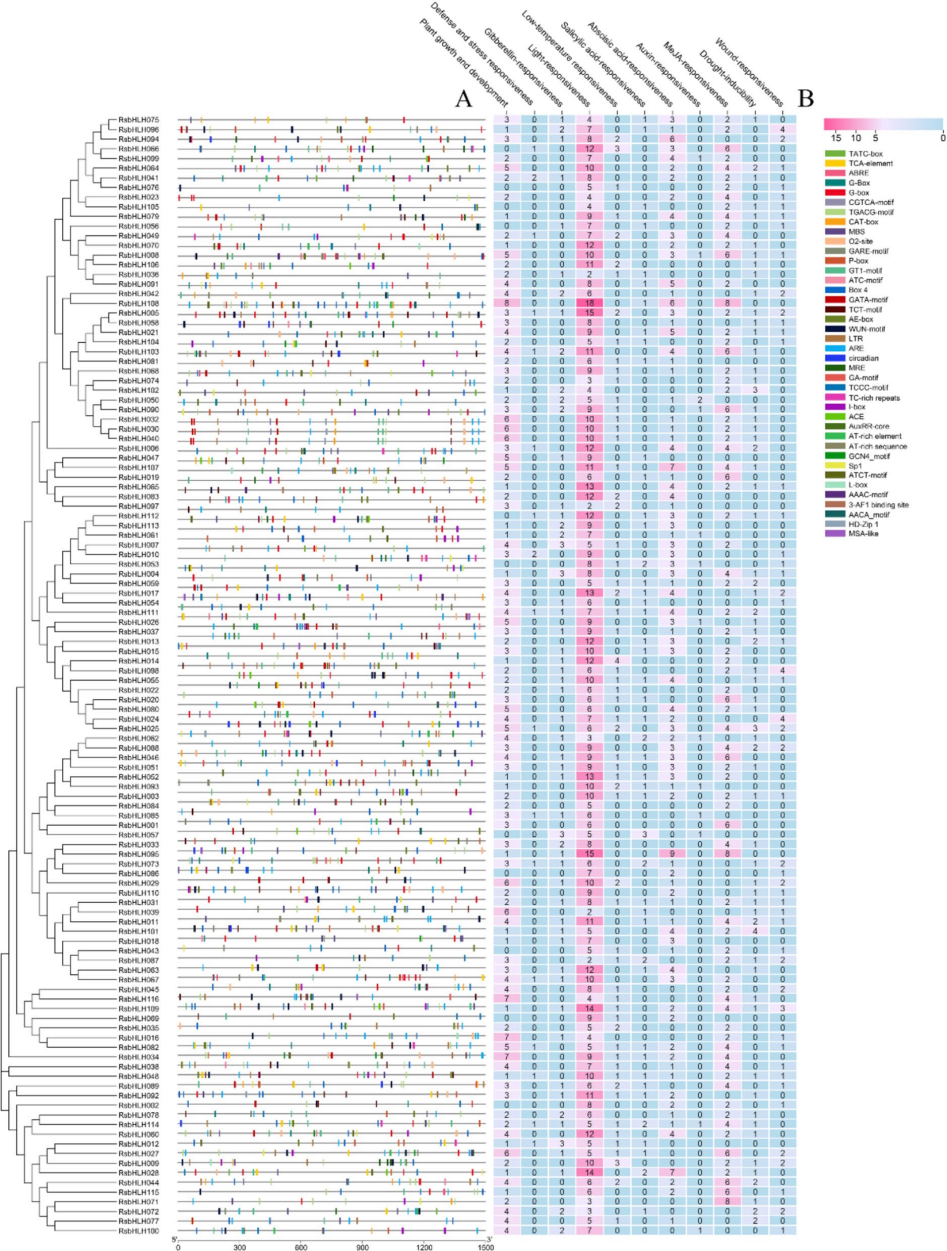
*Cis*-element analysis of *RsbHLH *genes from upstream 1500 bp sequence to the transcription start site. **A** Identification and classification of *cis-*acting elements in the promoter regions of *RsbHLH* genes in *R. simsii*. **B** Cis-acting elements analysis of the promoter regions of *RsbHLH* genes, the micro-parts in diverse colors are the sequence of the putative elements

To investigate the potential transcriptional regulation of *RsbHLH* genes, 1,500 bp upstream sequences from the translation start site of each gene were analyzed using the PlantCARE database (Fig. 3; Table S9). A total of 37 cis-regulatory elements were identified and classified into four categories: hormone-responsive elements (e.g., TCA, CGTCA, GARE, P-box, AuxRR-core) involved in salicylic acid, jasmonate, gibberellin, and auxin signaling; stress-responsive elements (ABRE, ARE, LTR, WUN-motif, TC-rich) associated with ABA response, anaerobic induction, low temperature, and wounding; light-responsive elements (GT1-box, I-box, G-box, CAT-box, Box 4, MRE, ACE, AE-box, SP1, etc.) implicated in light signaling and photomorphogenesis; and development-related elements (O2-site, GCN4-motif, circadian, AACA-motif, AT-rich element, HD-Zip1) potentially controlling tissue-specific expression and developmental processes. The abundance and diversity of these elements suggest that *RsbHLH* genes may integrate multiple hormonal, environmental, and developmental cues to coordinate plant growth, flower development, and stress adaptation. Among them, two heat stress-related cis-acting elements (ARE and TC-rich repeats) and four flower-related cis-regulatory elements (MSA-like, CAT-box, GATA-motif, and I-box) were identified, which were widely distributed across 12 (sub)groups (e.g., Ia, IIIa-d, IVc-d, Va-b, VII, VIIIa-c, IX, X, XI, XII, XIV, and XVIIa). Further analysis showed that almost all members of subgroups IIIb, IIId, IVd, VIIIa, and XIV contained both heat- and flower-related cis-elements, whereas only one RsbHLH gene in each of six subgroups (IIIc, IVc, Vb, VIIIc, IX, and XI) possessed two types of cis-regulatory elements (Figure S3).

### Gene duplication events of RsbHLH family genes

**Fig. 4:**
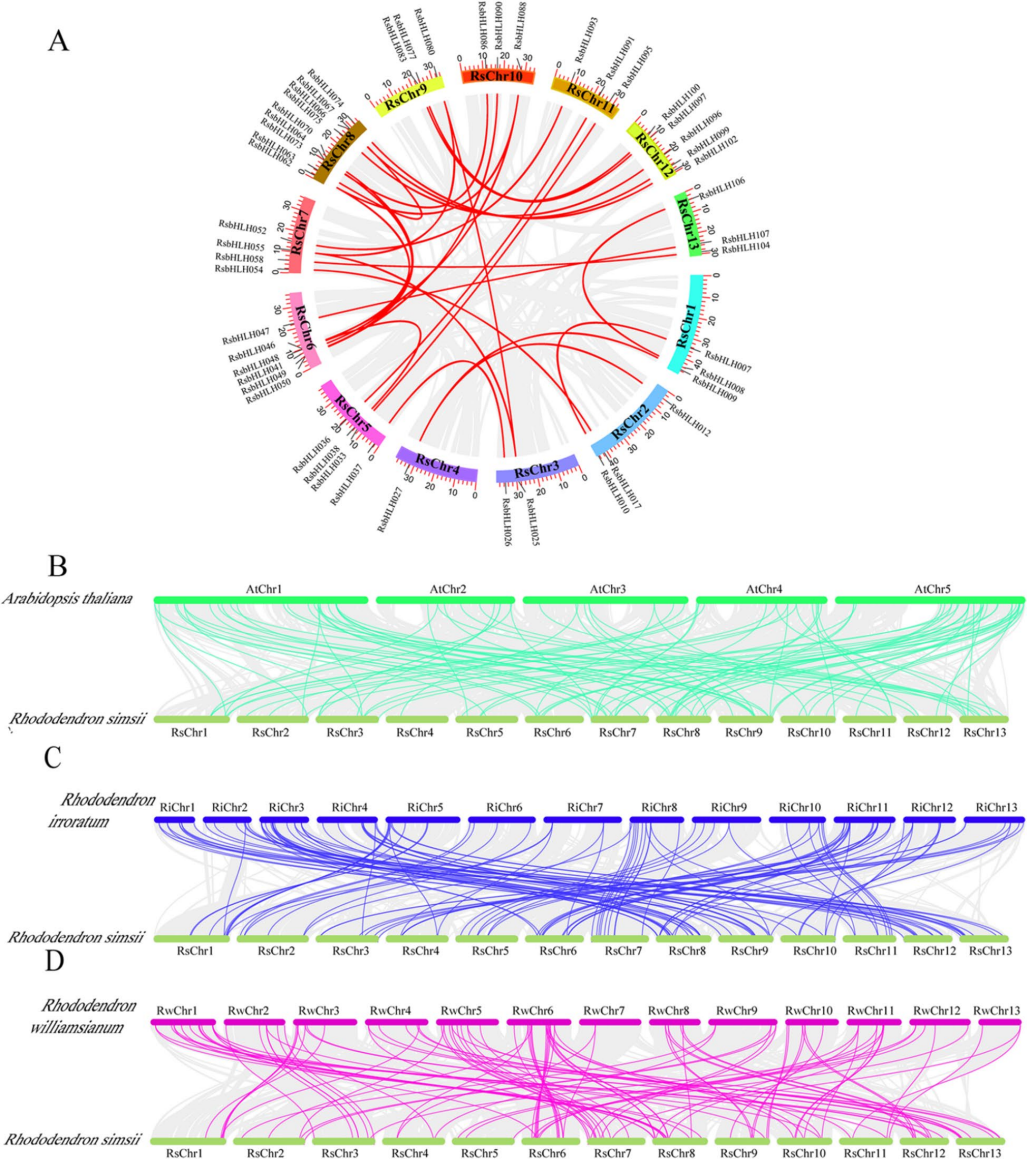
Collinear and synteny analyses of the *bHLH* genes. **A** Collinearity analysis of *RsbHLH* genes in *R. simsii* genome. The segmental duplicated *RsbHLH* gene pairs are shown with red lines. **B** Synteny analyses of the *bHLH* genes between *R.simsii* and *Arabidopsis.* **C** Synteny analyses of the *bHLH* genes between *R.simsii* and *R. irroratum.* **D** Synteny analyses of the *bHLH* genes between *R.simsii* and *R. williamsianum*. The gray line represents the syntenic gene sets of the entire genome. The green line represents the collinearity between *R. simsii* and *Arabidopsis bHLH* genes; the blue line represents the collinearity between *R. simsii* and *R. irroratum bHLH* genes; the pink line represents the collinearity between *R. simsii* and *R. williamsianum bHLH* genes

Gene duplication provides raw material for the evolution of new gene functions. To investigate the evolutionary patterns of the *RsbHLH* gene family, we performed a genome-wide analysis using MCScanX. Among the identified *RsbHLHs*, 42 and 57 genes originated from dispersed and whole-genome/segmental duplications, respectively, while seven genes were derived from tandem duplication.Given the major contribution of segmental duplication, we further examined its role in *RsbHLH* gene family expansion. Collinearity analysis based on all-against-all BLASTP searches identified 28 segmentally duplicated gene pairs (49 genes), accounting for 85.96% of WGD-derived *RsbHLHs* (Fig. 4A; Table S8). Selective pressure analysis using PAML revealed that all 28 gene pairs had Ka/Ks ratios < 1, indicating that these genes have been subjected to strong purifying selection (Fig. 4A; Table S10). To estimate the timing of these duplication events, Ks values were used for molecular dating. The Ks-based divergence times ranged from 58.84 Mya (Ks = 0.7178) to 301.89 Mya (Ks = 3.683), suggesting that most segmental duplication events predated the divergence between *Rhododendron* and *Vaccinium corymbosum* (~ 55.93 Mya) [43]. This finding implies that the expansion of the RsbHLH family largely occurred before the speciation of *Rhododendron*.

**Fig. 5:**
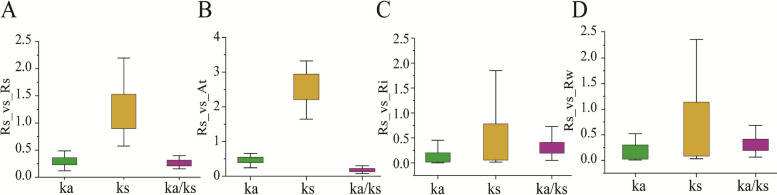
Ka, Ks, and Ka/Ks distributions of orthologous *bHLH* gene pairs. **A** Ka, Ks, and Ka/Ks ratio of segmental duplicated gene pairs; (**B**) Ka, Ks, and Ka/Ks ratio of orthologous *bHLH* gene pairs between *R. simsii* an*d Arabidopsis*; (**C**) Ka, Ks, and Ka/Ks ratio of orthologous *bHLH* gene pairs between *R. simsii* and *R. irroratum*; (**D**) Ka, Ks, and Ka/Ks ratio of orthologous *bHLH* gene pairs between *R. simsii* and *R. williamsianum*. The box plots are exhibiting the distributions of Ka, Ks, and Ka/Ks values among paralogs and orthologs. The small square and the line in the box represent average and median values of the Ka, Ks, and Ka/Ks values, respectively

To further elucidate the evolutionary relationships of RsbHLH family genes, we constructed comparative synteny maps between *R. simsii* and three other representative species (Fig. 4B-D; Table S11). We identified 69 orthologous groups between *Arabidopsis thaliana* and *R. simsii*, 130 between *R. irroratum* and *R. simsii*, and 120 between *R. williamsianum* and *R. simsii*. Notably, the number of orthologous pairs between *RsbHLH* and *RibHLH* was higher than that between *RsbHLH* and *RwbHLH*, suggesting closer evolutionary relationships between *R. simsii* and *R. irroratum*. We next calculated Ks, Ka, and Ka/Ks values for all orthologous gene pairs across the four species (Fig. 5). In all comparisons, Ka/Ks values were < 1, indicating that these orthologous genes have been subject to purifying selection. Moreover, the Ka/Ks ratios between *Arabidopsis* and *R. simsii* were markedly lower than those between *R. irroratum*-*R. simsii* and *R. williamsianum*-*R. simsii*, suggesting stronger functional constraint and evolutionary conservation across the more distantly related lineages.

### Transcript abundance of RsbHLHs in various organs

**Fig. 6:**
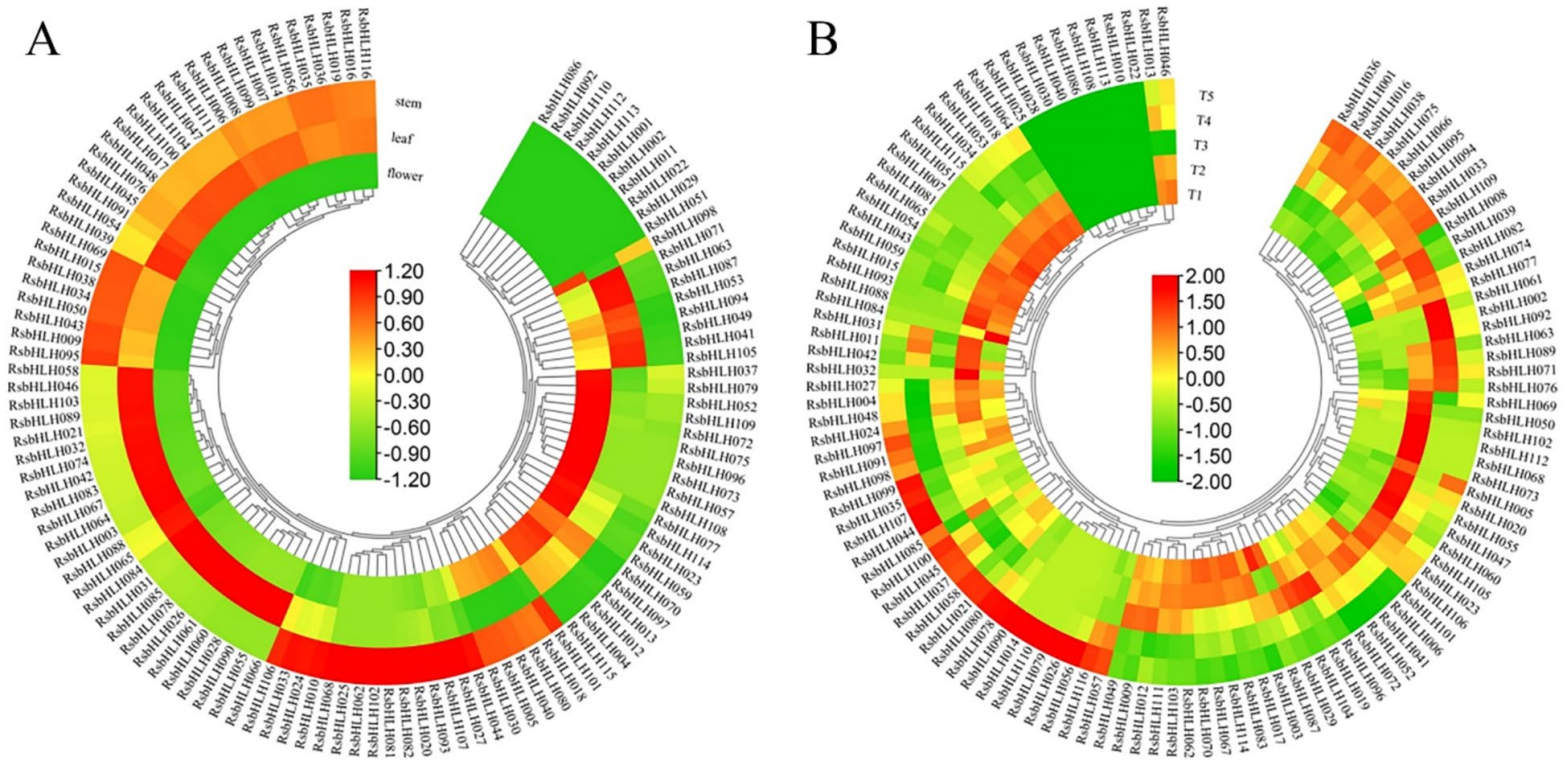
Expression patterns of the *RsbHLH* family genes in different tissues and different stages of flower development. **A** Expression profle of the *RsbHLH* family genes in different tissues of *R.simsii*. **B** The expression profle of the *RsbHLH* family genes in different stages of *R.simsii* flower development (T1-T5). The RPKM values obtained were normalized by log_2_ transformation and the heatmap constructed using the MeV software. The block colors represent the expression levels, like green, yellow and red correspond to low, medium and high expression values

bHLH transcription factors are known to play crucial roles in plant growth and development. To characterize the roles of *RsbHLHs* in *R. simsii*, we analyzed their expression profiles in stems, leaves, and flowers using RNA-seq data (Fig. 6A; Table S12). Among the identified genes, 49 *RsbHLHs* were highly expressed in flowers, 63 in leaves, and 58 in stems. Further analysis revealed that 46 *RsbHLHs* were expressed in all three organs, whereas 23 exhibited higher transcript abundance in vegetative tissues than in flowers, suggesting their potential involvement in leaf and stem development. Conversely, 13 *RsbHLHs* displayed flower-preferential expression, indicating putative roles in regulating floral development.

We further explored the expression dynamics of *RsbHLH* genes during five flower developmental stages (T1–T5) (Fig. 6B; Table S13). A total of 67 *RsbHLHs* showed differential expression, which could be classified into four major patterns: Ⅰ) Twenty-one *RsbHLHs* were highly expressed during the early stages (T1–T3). Ⅱ) Thirteen *RsbHLHs* displayed higher expression during the late stages (T4–T5). Ⅲ) Thirteen *RsbHLHs* were upregulated in T1–T3 and sharply downregulated in T4–T5. Ⅳ) Two *RsbHLHs* exhibited an initial decrease followed by a late-stage increase in expression. These distinct expression patterns suggest stage-specific regulatory functions of *RsbHLHs* in flower development.

Based on comparative orthology analysis, 20 species-specific *RsbHLHs* were identified, with 16 showing preferential expression in vegetative organs and 4 in flowers (Figure S4). Notably, three genes (*RsbHLH034*, *RsbHLH098*, and *RsbHLH114*) were specifically expressed in early flower stages (T1–T2), whereas *RsbHLH016* and *RsbHLH116* were preferentially expressed in late stages (T4–T5), implying their potential roles in stage-specific floral differentiation.

### Prediction of protein–protein interaction

**Fig. 7:**
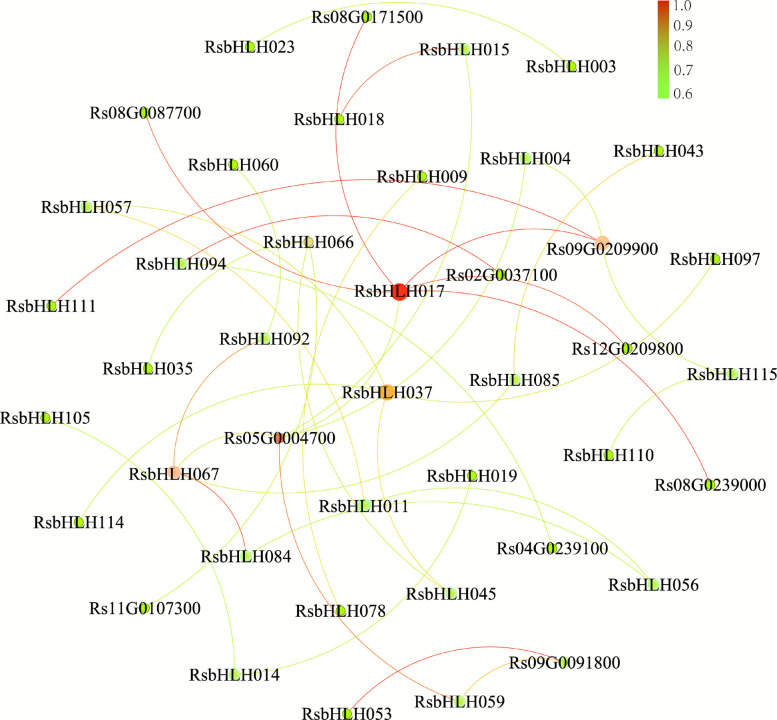
Prediction and analysis of the protein–protein interaction network between RsbHLH proteins and their closely associated partners. The network was predicted using the STRING database

bHLH proteins can form homo- or heterodimers, which is essential for DNA recognition and binding [9, 10]. To explore potential functional interactions of RsbHLHs, we constructed PPI networks using the STRING database (confidence score > 0.6) (Fig. 7; Table S14). The network contained 42 nodes (bHLH proteins) and 38 edges (interaction relationships), with an average degree of 1.5. A total of 32 RsbHLHs were included, of which 26 interacted with more than one other RsbHLH. Three key hub proteins (RsbHLH017, RsbHLH037, and RsbHLH067) were identified with degrees greater than 4. For instance, RsbHLH037 interacted with four RsbHLHs (RsbHLH045, RsbHLH057, RsbHLH097, and RsbHLH114), while RsbHLH067 interacted with three RsbHLHs (RsbHLH084, RsbHLH085, and RsbHLH092). We further examined interactions between RsbHLHs and other family proteins. RsbHLH017 showed extensive connectivity, interacting with six proteins, including JAZ1 (Rs05G0004700), MYB75 (Rs08G0087700), MYB0 (Rs08G0087700), MYB66 (Rs08G0171500), TTG1 (Rs09G0209900), and TT2 (Rs12G0209800). RsbHLH059 interacted with TIFY7 (Rs09G0091800) and JAZ (Rs05G0004700), and RsbHLH094 interacted with CRY2 (Rs02G0037100) and PHYB (Rs04G0239100). Additionally, four RsbHLHs (RsbHLH015, RsbHLH053, RsbHLH066, and RsbHLH115) were predicted to interact with Rs05G0004700, Rs09G0091800, Rs11G0107300, and Rs09G0209900, respectively. Functional annotation of interacting proteins revealed roles in flower development, flavonoid biosynthesis, and stress responses. These results suggest that RsbHLHs may act as key hubs in regulatory networks governing both development and stress adaptation.

### Validation of RNA-seq data using RT-qPCR

**Fig. 8:**
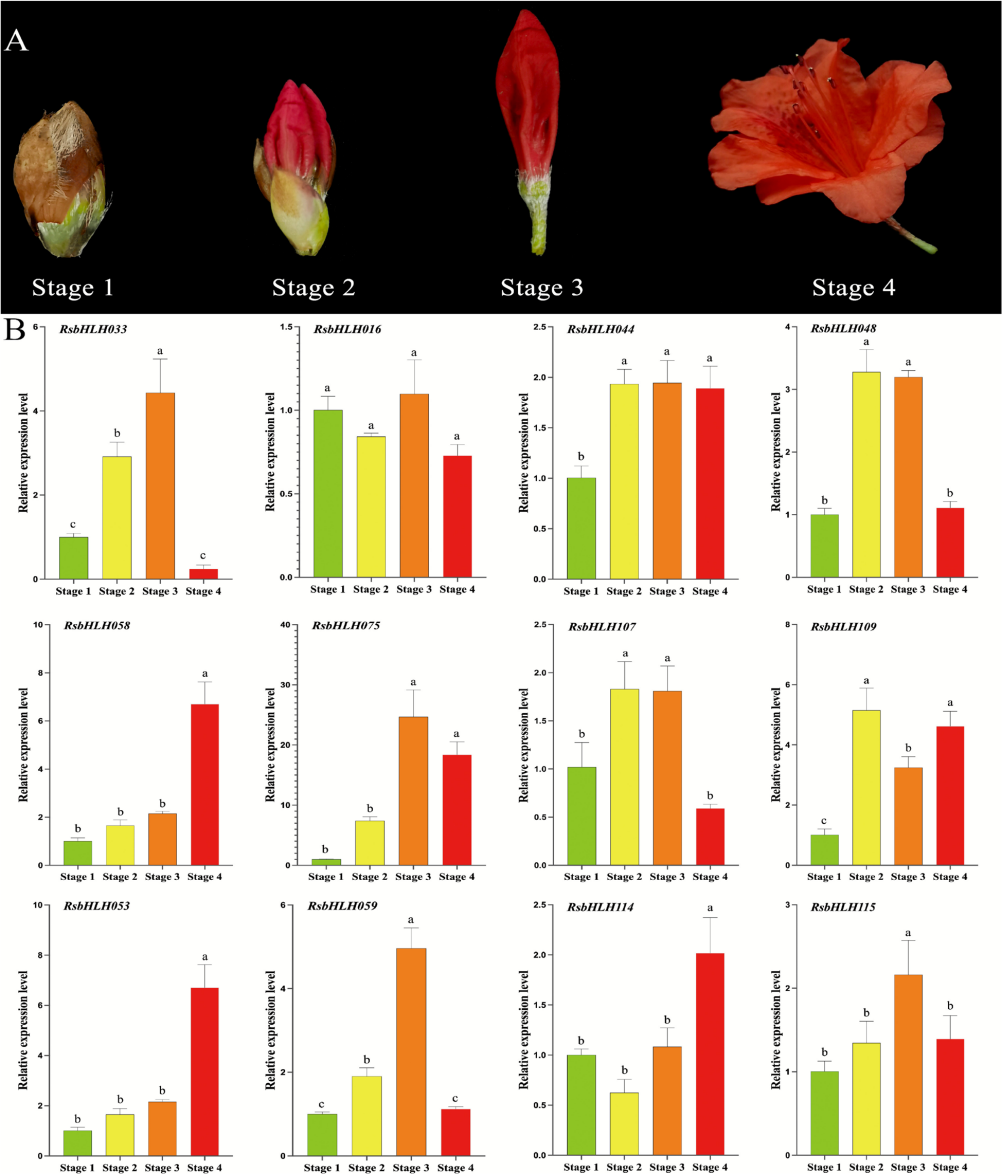
Expression profiles of 12 *RsbHLH* genes during four stages of flower development in *R. simsii*. **A** Four flower development stages (stage 1–4) of *R. simsii*. Four stages, including stage 1, stage 2, stage 3, and stage 4 in qRT-PCR analysis, correspond to T2, T3, T4, and T5 in RNA-seq data, respectively. **B** Transcript levels of 12 *RsbHLH* genes were quantified by RT-qPCR using *EF1a*, *GAPDH*, and *RG7* as reference genes. Each value represents the mean ± SD of three biological replicates, each with three technical replicates (*N* = 6). Different lowercase letters above the bars indicate significant differences among stages (*p* < 0.05). **C** A comprehensive regression analysis was performed to assess the correlation between RNA-seq and RT-qPCR data across all four developmental stages (T1, T2, T3, and T4). Scatter plots with linear regression models were generated using Microsoft Excel to visualize the quantitative relationships between the two techniques

To validate the RNA-seq results, RT-qPCR was performed on 12 *RsbHLH* genes exhibiting high transcript abundance across various organs and flower developmental stages of *R. simsii* (Fig. 8A, B, Table S15). The expression patterns obtained from RT-qPCR showed strong agreement with the RNA-seq data, with Pearson correlation coefficients ranging from 0.924 to 0.991 across the four flower developmental stages (Figure S5). Comparative analysis confirmed that the temporal expression trends observed in RT-qPCR were consistent with those from RNA-seq, supporting the reliability of the transcriptome data.

### Expression profiles of RsbHLHs under high-temperature treatment

**Fig. 9:**
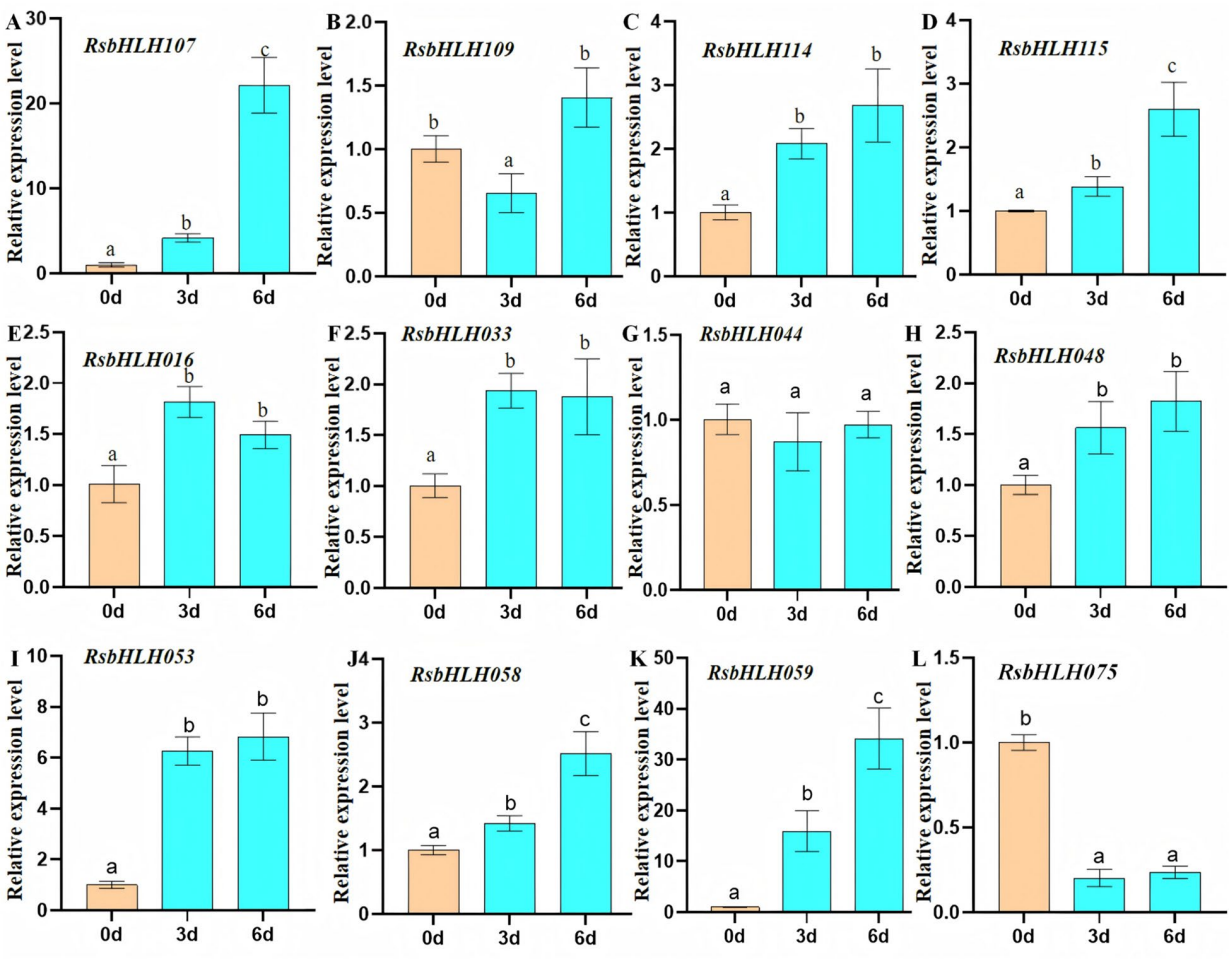
Expression patterns of 12 candidate *RsbHLH* genes in *R. simsii* treated with high-temperature. Total RNA was extracted from young leaves at different time points after high-temperature treatment (3d and 6 d) and without high-temperature treatment (0d) and subjected to quantitative RT-PCR analysis. The *EF1a, GAPDH, RG7* genes was used as an internal reference gene, the relative expression level was calculated by 2^−ΔΔCt^. The data are the mean ± SE of three independent biological samples. Lowercase letters indicate significant differences between treatments (*p* < 0.05)

High temperatures can significantly affect plant physiological processes, including photosynthesis, water balance, growth, and flowering (e.g., shortened bloom period, reduced bud formation, and floral deformities). To investigate the roles of *RsbHLHs* in heat stress response, the expression levels of 12 flower-expressed *RsbHLH* genes were examined under high-temperature treatment (Fig. 9, Table S16). Among these genes, nine *RsbHLHs* (*RsbHLH016*, *033*, *048*, *053*, *058*, *059*, *107*, *114*, and *115*) were markedly upregulated, suggesting that they may positively regulate the response to high-temperature stress. In contrast, *RsbHLH075* was strongly downregulated, indicating a potential role in negative regulation of heat stress response. These results highlight specific *RsbHLHs* as candidate regulators of high-temperature adaptation in *R. simsii*.

### Subcellular location of RsbHLH053 and RsbHLH059 proteins

**Fig. 10:**
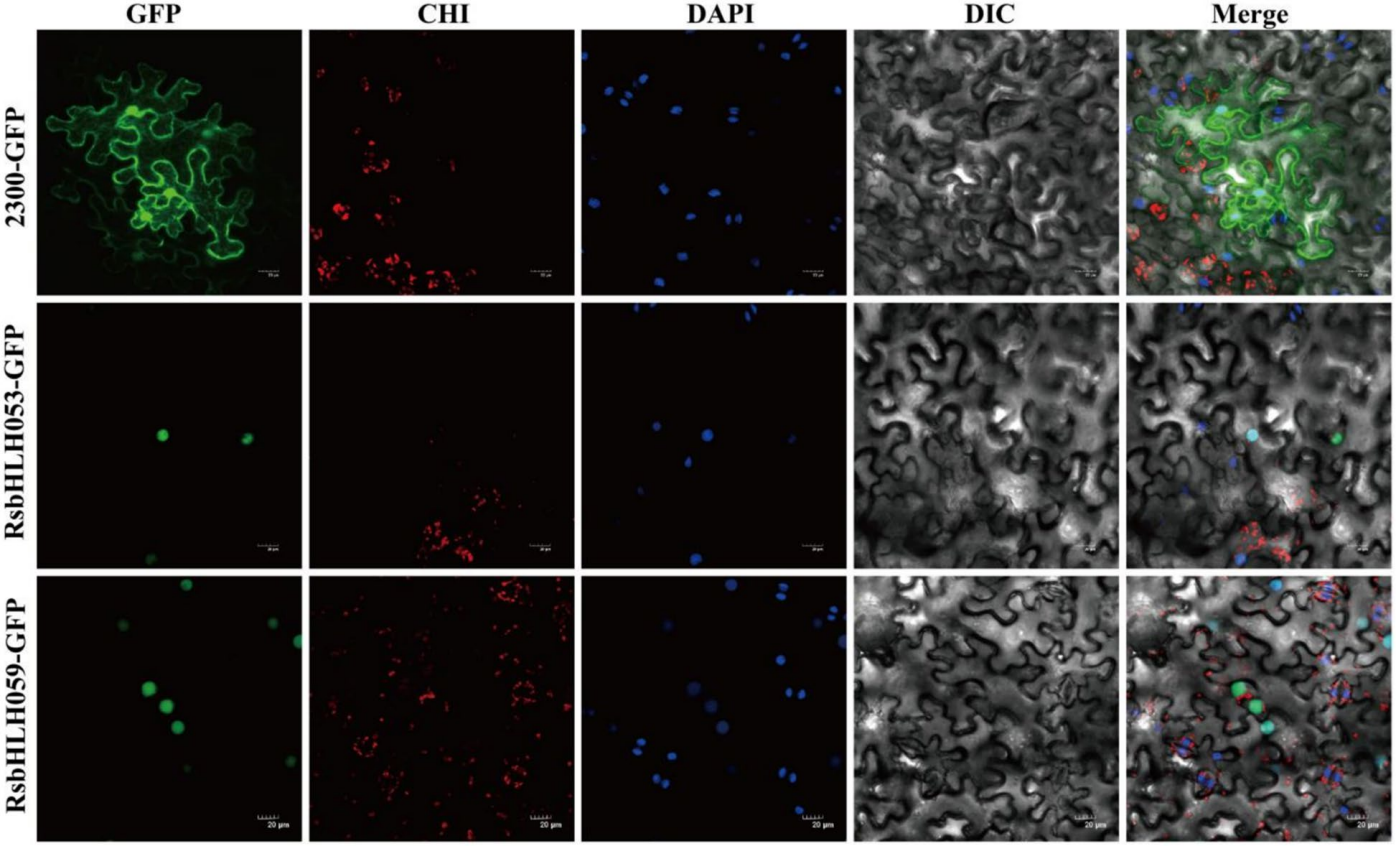
Subcellular distribution of RsbHLH053 and RsbHLH059 proteins. GFP alone or GFP fusions with RsbHLH053 and RsbHLH059, driven by the CaMV35S promoter, were transiently expressed in tobacco leaf epidermal cells. The localization of the fusion proteins was visualized using laser scanning confocal microscopy. Scale bar = 20 µm. GFP, Green fluorescence channel showing the protein-GFP fusion signal; CHI, Chloroplast autofluorescence channel (red) for chloroplast localization reference; DAPI, Blue fluorescence channel staining nuclei; DIC, Differential interference contrast image of the cell structure; Merge, Overlay of all channels to illustrate the co-localization of the protein, chloroplasts, and nuclei

To further verify the predicted subcellular localization of RsbHLHs, two *RsbHLH* genes, *RsbHLH053* and *RsbHLH059*, both highly expressed in floral stages and induced by heat stress, were selected for proforming subcellular location analysis. The full-length coding sequences of *RsbHLH053* and *RsbHLH059* were fused with GFP to generate 35S-RsbHLH053/RsbHLH059-GFP vectors, which were transiently expressed in tobacco leaf cells (Fig. 10). Fluorescence observation showed that both RsbHLH053 and RsbHLH059 proteins were localized predominantly in the nucleus, whereas free GFP was distributed throughout the cells. These results indicate that RsbHLH053 and RsbHLH059 are likely expressed and function in the nucleus, consistent with their roles as transcription factors.

## Discussion

The bHLH gene family is one of the largest transcription factor families in plants, playing crucial roles in development and stress responses [4, 44–47]. Despite their importance, a comprehensive characterization of *bHLH* genes in *R. simsii* had not been performed. In this study, we identified 116 *RsbHLH* genes and classified them into 17 subgroups based on phylogenetic relationships with *Arabidopsis bHLHs*. Compared with other species, such as *Arabidopsis thaliana* (21 subfamilies), apple (23), and peach (19), this indicates both evolutionary conservation and species-specific diversification [48–50].

The number of *bHLH* genes increases from lower plants (e.g., *Volvox carteri*, *Chlamydomonas reinhardtii*) to higher plants (e.g., *Oryza sativa*, *Solanum lycopersicum*, *Vitis vinifera*, *Fragaria vesca*, *Arabidopsis*, *R. simsii*), suggesting expansion during plant evolution [9, 51–54]. Gene duplication, including WGD/segmental duplication, tandem duplication, and dispersed duplication, is a major driver of gene family expansion [55–58]. In *R. simsii*, 99 *RsbHLHs* arose from dispersed or WGD/segmental duplication, with 28 gene pairs (49 genes) derived from ancient segmental duplications prior to *Rhododendron* diversification. Functional divergence likely occurred after these duplications, as indicated by limited positive correlation among three segmental gene pairs (*RsbHLH75*/*RsbHLH96*, *RsbHLH017*/*RsbHLH054*, *RsbHLH033*/*RsbHLH095*, *P* < 0.01). For example, the duplicated gene pair *RsbHLH033*/*RsbHLH095* exhibited divergent expression patterns, with *RsbHLH033* expressed at all five stages of flower development, whereas *RsbHLH095* was expressed only in the late stages (T3-T5). Analysis of cis-regulatory elements further revealed motifs involved in light, GA, ABA, and stress responses, suggesting roles in flower development and environmental adaptation. The subcellular localization of two RsbHLH proteins (RsbHLH053 and RsbHLH059) was detected in the nucleus, consistent with the prediction results. However, analyzing only two candidates is insufficient to draw broad conclusions, though it provides preliminary evidence supporting their nuclear localization and possible transcriptional regulatory roles.

Previous studies have demonstrated that bHLH TFs play crucial roles in floral development [59–61]. For example, *PhFBH4* regulates flower senescence via ethylene biosynthesis in petunia [62], *SlMS10* is required for tapetum development and pollen fertility in tomato [63], and *TIP2* regulates anther development in rice by controlling *TDR* and *EAT1* expression [64]*.* Similarly, *LlbHLH12* negatively regulates anthocyanin biosynthesis in *Lycoris longituba* petals [65], while *BIGPETALp* controls petal size in *Arabidopsis* [66]. Collectively, these findings highlight the crucial role of bHLH TFs in floral development. Our study identified 49 *RsbHLHs* with high expression in flowers, among which 13 exhibited significantly higher expression than in vegetative tissues, suggesting their involvement in *R. simsii* floral development. Orthology analysis revealed that these 13 genes correspond to *Arabidopsis BPE*, *JAM2*, *ILR3*, *MYC4*, *BHLH79*, *SACL3*, *BHLH48*, *JAM2*, *ATMYC1*, *BIM1*, *BIM2*, *BHLH103*, and *BHLH041*, all previously reported to regulate floral organ growth, flowering time, or fertility [66–70]. For example, *BPE* modulates petal size in *Arabidopsis* [66], and overexpression of *AtbHLH48* promotes early flowering by increasing FT transcript abundance under long-day conditions [67]. JAZ proteins inhibit the MYC4-MYB module to regulate stamen development [69], while AabHLH48 from *Adonis amurensis* activates *FRUITFULL* expression to induce early flowering in *Arabidopsis* [71]. In rice PTGMS line PA64S, hypermethylation of *BIM2* reduces BR target gene expression and affects male fertility[70]. Moreover, JAM2 and JAM3 act as transcriptional repressors influencing male fertility [72].

High temperature is a major abiotic stress that adversely affects *R. simsii* physiology, leading to reduced photosynthesis, shortened flowering period, decreased bud formation, and floral malformations [73]. To explore potential heat-responsive *bHLH* genes, we analyzed the expression of 12 flower-preferential *RsbHLHs* under heat stress. Nine genes (*RsbHLH016*, *033*, *048*, *053*, *058*, *059*, *107*, *114*, and *115*) were significantly upregulated compared with controls, suggesting their involvement in heat stress response. Notably, *RsbHLH053* and *RsbHLH059* are orthologous to *Arabidopsis MYC2* and *JAM2*/*bHLH13*, respectively, both of which have been implicated in thermotolerance regulation. For example, high temperature induces *NtMYC2a* expression to regulate nicotine and JA biosynthesis in tobacco [74], and *JAM* genes are required for *Arabidopsis* TWA1-mediated thermotolerance [75]. These findings, together with our RT-qPCR data, indicate that *RsbHLH053* and *RsbHLH059* likely play central roles in heat stress response in *R. simsii*.

## Conclusions

In this study, we totally characterized 116 RsbHLH family genes in *R. simsii* genome, which were divided into 17 groups based on the tree topology, structure analysis, and identified *AtbHLHs*. Characterization of cis-acting regulatory elements in promoter regions of *RsbHLHs* showed that the heat-stress-related (ARE and TC-rich repeats) and flower-related (MSA-like, CAT-box, GATA-motif, and I-box) cis-regulatory elements were widely distributed in 39 *RsbHLH* genes from 12 groups. Expression analysis of the RsbHLH family genes in different organs of *R. simsii* revealed that 23 and 13 *RsbHLH* genes were specifically expressed in vegetative organs and flowers, respectively. Investigation of the RsbHLH family genes in five distinct stages of flower development showed that 49 *RsbHLH* genes were differentially expressed in different stages of flower development. Then, we further explored the expression patterns of 12 randomly selected *RsbHLH* genes with high expression levels in flower in high-temperature-treated *R. simsii,* found that nine *RsbHLHs* were significantly upregulated after high-temperature treatment. Function annotation revealed that *RsbHLH53* (torthologous gene of *MYC2*) and *RsbHLH59* (orthologous gene of *JAM2/bHLH13*) might play essential roles in regulating high temperature stress response. This study not only systematically identified and analyzed the RsbHLH family genes in *R. simsii*, but also screened two candidate genes potentially involved in flower development and heat stress response. However, functional validation of these candidate genes and the limited number of experimental samples represent potential limitations, which should be addressed in future studies.

## Supplementary Information

Supplementary Material 1: Supplementary Figure S1. Chromosome localization of RsbHLH gene family. Supplementary Figure S2. Sequence logos of RsbHLH proteins. Supplementary Figure S3. The Spearman’s corrlation coefficient of RNA-seq data and RT-qPCR. Supplementary Figure S4. The expression patterns of species-sepcific *RsbHLH *genes in different organs and in different stages of flower development. Supplementary Table S1. Protein sequences of *bHLH *genes from *A. thaliana*, *R.simsii*,*R.williamsianum*, and *R.irroratum*. Supplementary Table S2. The promoters of the *bHLH *genes *in R. simsii.*Supplementary Table S3. The primers of 12 candidate genes used in this study. Supplementary Table S4. Coding sequences of RsbHLH053 and RsbHLH059 cloned for GFP fusion and subcellular localization. Supplementary Table S5. Physicochemical properties and subcellular localization of bHLH protein in *R. simsii.*Supplementary Table S6. The distribution of *RsbHLH* genes on each chromosome in *R. simsii*. Supplementary Table S7. The classification of the *bHLH* genes in*Arabidopsis*, *R. simsii*, *R. irroratum* and *R. williamsianum* based on the phylogenetic analysis. Supplementary Table S8. Conservative motifs of RsbHLH protein in *R. simsii*. Supplementary Table S9. The promoter Cis-element functional classification of *RsbHLH* genes. Supplementary Table S10. Segmentally duplicated RsbHLH gene pairs. Supplementary Table S11. One-to-one orthologous relationships between* R. simsii *and other plants. Supplementary Table S12. The expression patterns of RsbHLH family genes. Supplementary Table S13. The expression patterns of RsbHLH family genes in different stages of flower development. Supplementary Table S14. The protein interaction network. Supplementary Table S15. The RT-qPCR data of 12 *RsbHLH* genes in different stages of flower development. Supplementary Table S16. The RT-qPCR data of *RsbHLH* genes in high temperature-treated *R. simsii*.

## Data Availability

All data supporting the findings of this study are available within the paper and its Supplementary Information.

## References

[1] Ogbourne S, Antalis TM. Transcriptional control and the role of silencers in transcriptional regulation in eukaryotes. Biochem J. 1998;331((Pt 1)(Pt 1)):1–14. 9512455 10.1042/bj3310001 PMC1219314

[2] Zuo ZF, Lee HY, Kang HG. Basic helix-loop-helix transcription factors: regulators for plant growth development and abiotic stress responses. Int J Mol Sci. 2023;24(2):1419. 10.3390/ijms24021419. 10.3390/ijms24021419 PMC9867082 36674933

[3] Gao M, Zhu Y, Yang J, Zhang H, Cheng C, Zhang Y, et al. Identification of the grape basic helix–loop–helix transcription factor family and characterization of expression patterns in response to different stresses. Plant Growth Regul. 2019;88:19–39.

[4] Qian Y, Zhang T, Yu Y, Gou L, Yang J, Xu J, et al. Regulatory mechanisms of bHLH transcription factors in plant adaptive responses to various abiotic stresses. Front Plant Sci. 2021;12:677611. 34220896 10.3389/fpls.2021.677611 PMC8250158

[5] Buck MJ, Atchley WR. Phylogenetic analysis of plant basic helix-loop-helix proteins. J Mol Evol. 2003;56(6):742–50. 12911037 10.1007/s00239-002-2449-3

[6] Massari ME, Murre C. Helix-loop-helix proteins: regulators of transcription in eucaryotic organisms. Mol Cell Biol. 2000;20(2):429–40. 10611221 10.1128/mcb.20.2.429-440.2000 PMC85097

[7] Hao Y, Zong X, Ren P, Qian Y, Fu A. Basic Helix-Loop-Helix (bHLH) transcription factors regulate a wide range of functions in arabidopsis. Int J Mol Sci. 2021;22(13):7152. 10.3390/ijms22137152. 10.3390/ijms22137152 PMC8267941 34281206

[8] Jin C, Huang XS, Li KQ, Yin H, Li LT, Yao ZH, et al. Overexpression of a bHLH1 transcription factor of *Pyrus ussuriensis* confers enhanced cold tolerance and increases expression of stress-responsive genes. Front Plant Sci. 2016;7:441. 27092159 10.3389/fpls.2016.00441 PMC4820633

[9] Wang P, Su L, Gao H, Jiang X, Wu X, Li Y, et al. Genome-wide characterization of bHLH genes in grape and analysis of their potential relevance to abiotic stress tolerance and secondary metabolite biosynthesis. Front Plant Sci. 2018;9:64. 29449854 10.3389/fpls.2018.00064 PMC5799661

[10] Wang R, Zhao P, Kong N, Lu R, Pei Y, Huang C, et al. Genome-wide identification and characterization of the potato bHLH transcription factor family. Genes. 2018;9(1):54. 10.3390/genes9010054. 10.3390/genes9010054 PMC5793205 29361801

[11] An JP, Li HH, Song LQ, Su L, Liu X, You CX, et al. The molecular cloning and functional characterization of MdMYC2, a bHLH transcription factor in apple. Plant Physiol Biochem. 2016;108:24–31. 27404131 10.1016/j.plaphy.2016.06.032

[12] Salih H, Tan L, Htet NNW. Genome-wide identification, characterization of bHLH transcription factors in mango. Trop Plant Biol. 2021;14:72–81.

[13] Song XM, Huang ZN, Duan WK, Ren J, Liu TK, Li Y, et al. Genome-wide analysis of the bHLH transcription factor family in Chinese cabbage (*Brassica rapa* ssp. *pekinensis*). Mol Genet Genomics. 2014;289(1):77–91. 24241166 10.1007/s00438-013-0791-3

[14] Lu R, Zhang J, Liu D, Wei YL, Wang Y, Li XB. Characterization of *bHLH*/*HLH* genes that are involved in brassinosteroid (BR) signaling in fiber development of cotton (*Gossypium hirsutum*). BMC Plant Biol. 2018;18(1):304. 30482177 10.1186/s12870-018-1523-y PMC6258498

[15] Wu H, Ren Z, Zheng L, Guo M, Yang J, Hou L, et al. The bHLH transcription factor GhPAS1 mediates BR signaling to regulate plant development and architecture in cotton. Crop J. 2021;9(5):1049–59.

[16] Zhu Z, Liang H, Chen G, Li F, Wang Y, Liao C, et al. The bHLH transcription factor SlPRE2 regulates tomato fruit development and modulates plant response to gibberellin. Plant Cell Rep. 2019;38(9):1053–64. 31123809 10.1007/s00299-019-02425-x

[17] Ji C, Li H, Chen L, Xie M, Wang F, Chen Y, et al. A novel rice bHLH transcription factor, DTD, acts coordinately with TDR in controlling tapetum function and pollen development. Mol Plant. 2013;6(5):1715–8. 23519457 10.1093/mp/sst046

[18] Wang L, Tang W, Hu Y, Zhang Y, Sun J, Guo X, et al. A MYB/bHLH complex regulates tissue-specific anthocyanin biosynthesis in the inner pericarp of red-centered kiwifruit *Actinidia chinensis* cv. Hongyang. Plant J. 2019;99(2):359–78. 30912865 10.1111/tpj.14330

[19] Li C, Qiu J, Ding L, Huang M, Huang S, Yang G, et al. Anthocyanin biosynthesis regulation of DhMYB2 and DhbHLH1 in *Dendrobium* hybrids petals. Plant Physiol Biochem. 2017;112:335–45. 28131062 10.1016/j.plaphy.2017.01.019

[20] Zhao Q, He F, Reeves MJ, Pan Q-H, Duan C-Q, Wang JJ. Expression of structural genes related to anthocyanin biosynthesis of *Vitis amurensis*. J For Res. 2016;27(3):647–57.

[21] Miura K, Shiba H, Ohta M, Kang SW, Sato A, Yuasa T, et al. Slice1 encoding a MYC-type transcription factor controls cold tolerance in tomato, *Solanum lycopersicum*. Plant Biotechnol. 2012;29(3):253–60.

[22] Guo J, Sun B, He H, Zhang Y, Tian H, Wang B. Current understanding of bHLH transcription factors in plant abiotic stress tolerance. Int J Mol Sci. 2021;22(9):4921. 10.3390/ijms22094921. 10.3390/ijms22094921 PMC8125693 34066424

[23] Babitha KC, Ramu SV, Pruthvi V, Mahesh P, Nataraja KN, Udayakumar M. Co-expression of AtbHLH17 and AtWRKY28 confers resistance to abiotic stress in Arabidopsis. Transgenic Res. 2013;22(2):327–41. 22948308 10.1007/s11248-012-9645-8

[24] Wang X, Zhou P, Hu X, Bai Y, Zhang C, Fu Y, et al. T2t genome, pan-genome analysis, and heat stress response genes in Rhododendron species. Imeta. 2025;4(2):e70010. 40236772 10.1002/imt2.70010 PMC11995181

[25] Mistry J, Chuguransky S, Williams L, Qureshi M, Salazar GA, Sonnhammer ELL, et al. Pfam: the protein families database in 2021. Nucleic Acids Res. 2021;49(D1):D412–9. 33125078 10.1093/nar/gkaa913 PMC7779014

[26] Lei B, Song M, Li X, Dang X, Qin R, Zhu S, et al. SMART v1.0: a database for small molecules with functional implications in plants. Interdiscip Sci Comput Life Sci. 2022;14(1):279–83. 10.1007/s12539-021-00480-1 34648133

[27] Lu S, Wang J, Chitsaz F, Derbyshire MK, Geer RC, Gonzales NR, et al. CDD/SPARCLE: the conserved domain database in 2020. Nucleic Acids Res. 2020;48(D1):D265–8. 31777944 10.1093/nar/gkz991 PMC6943070

[28] Rackham OJ, Madera M, Armstrong CT, Vincent TL, Woolfson DN, Gough J. The evolution and structure prediction of coiled coils across all genomes. J Mol Biol. 2010;403(3):480–93. 20813113 10.1016/j.jmb.2010.08.032

[29] Gillani M, Pollastri G. Protein subcellular localization prediction tools. Comput Struct Biotechnol J. 2024;23:1796–807. 38707539 10.1016/j.csbj.2024.04.032 PMC11066471

[30] Bailey TL, Johnson J, Grant CE, Noble WS. The MEME Suite. Nucleic Acids Res. 2015;43(W1):W39-49. 25953851 10.1093/nar/gkv416 PMC4489269

[31] Tamura K, Stecher G, Kumar S. MEGA11: molecular evolutionary genetics analysis version 11. Mol Biol Evol. 2021;38(7):3022–7. 33892491 10.1093/molbev/msab120 PMC8233496

[32] Nguyen LT, Schmidt HA, von Haeseler A, Minh BQ. IQ-TREE: a fast and effective stochastic algorithm for estimating maximum-likelihood phylogenies. Mol Biol Evol. 2015;32(1):268–74. 25371430 10.1093/molbev/msu300 PMC4271533

[33] Lescot M, Déhais P, Thijs G, Marchal K, Moreau Y, Van de Peer Y, et al. PlantCARE, a database of plant cis-acting regulatory elements and a portal to tools for in silico analysis of promoter sequences. Nucleic Acids Res. 2002;30(1):325–7. 11752327 10.1093/nar/30.1.325 PMC99092

[34] Wang Y, Tang H, Debarry JD, Tan X, Li J, Wang X, et al. MCscanX: a toolkit for detection and evolutionary analysis of gene synteny and collinearity. Nucleic Acids Res. 2012;40(7):e49. 22217600 10.1093/nar/gkr1293 PMC3326336

[35] Yang ZJMb. Evolution: PAML 4: phylogenetic analysis by maximum likelihood. Mol Biol Evol. 2007;24(8):1586–1591. 10.1093/molbev/msm088 17483113

[36] Luypaert G, Witters J, Van Huylenbroeck J, De Clercq P, De Riek J, De Keyser E. Induced expression of selected plant defence related genes in pot azalea, *Rhododendron simsii* hybrid. Euphytica. 2017;213(10):227. 10.1007/s10681-017-2010-5.

[37] Chen K, Liu H, Lou Q, Liu Y. Ectopic expression of the grape hyacinth (*Muscari armeniacum*) R2R3-MYB transcription factor gene, MaAN2, induces anthocyanin accumulation in tobacco. Front Plant Sci. 2017;8:965. 28642775 10.3389/fpls.2017.00965 PMC5462982

[38] Xu L, Dong Z, Fang L, Luo Y, Wei Z, Guo H, et al. OrthoVenn2: a web server for whole-genome comparison and annotation of orthologous clusters across multiple species. Nucleic Acids Res. 2019;47(W1):W52–8. 31053848 10.1093/nar/gkz333 PMC6602458

[39] von Mering C, Huynen M, Jaeggi D, Schmidt S, Bork P, Snel B. STRING: a database of predicted functional associations between proteins. Nucleic Acids Res. 2003;31(1):258–61. 12519996 10.1093/nar/gkg034 PMC165481

[40] Qin S, Liang Y, Xie Y, Wei G, Lin Q, Qin W, et al. Genome-wide analysis of the bHLH gene family in *Spatholobus suberectus* identifies SsbHLH112 as a regulator of flavonoid biosynthesis. BMC Plant Biol. 2025;25(1):594. 40329176 10.1186/s12870-025-06452-7 PMC12054232

[41] Tong C, Jia Y, Hu H, Zeng Z, Chapman B, Li C. Pangenome and pantranscriptome as the new reference for gene-family characterization: a case study of basic helix-loop-helix (bHLH) genes in barley. Plant Commun. 2025;6(1). 10.1016/j.xplc.2024.101190. 10.1016/j.xplc.2024.101190 PMC11783906 39521956

[42] Zhou X, Liao Y, Kim S-U, Chen Z, Nie G, Cheng S, et al. Genome-wide identification and characterization of bHLH family genes from *Ginkgo biloba*. Sci Rep. 2020;10(1):13723. 32792673 10.1038/s41598-020-69305-3 PMC7426926

[43] Yang FS, Nie S, Liu H, Shi TL, Tian XC, Zhou SS, et al. Chromosome-level genome assembly of a parent species of widely cultivated azaleas. Nat Commun. 2020;11(1):5269. 33077749 10.1038/s41467-020-18771-4 PMC7572368

[44] Sun X, Wang Y, Sui N. Transcriptional regulation of bHLH during plant response to stress. Biochem Biophys Res Commun. 2018;503(2):397–401. 30057319 10.1016/j.bbrc.2018.07.123

[45] Mao K, Dong Q, Li C, Liu C, Ma F. Genome wide identification and characterization of Apple bHLH transcription factors and expression analysis in response to drought and salt stress. Front Plant Sci. 2017;8:480. 28443104 10.3389/fpls.2017.00480 PMC5387082

[46] Pires N, Dolan L. Origin and diversification of basic-helix-loop-helix proteins in plants. Mol Biol Evol. 2010;27(4):862–74. 19942615 10.1093/molbev/msp288 PMC2839125

[47] Wang K, Liu H, Mei Q, Yang J, Ma F, Mao K. Characteristics of bHLH transcription factors and their roles in the abiotic stress responses of horticultural crops. Sci Hortic. 2023;310:111710.

[48] Wu Y, Wu S, Wang X, Mao T, Bao M, Zhang J. Genome-wide identification and characterization of the bHLH gene family in an ornamental woody plant *Prunus mume*. Hortic Plant J. 2022;8(4):531–44.

[49] Yang J, Gao M, Huang L, Wang Y, van Nocker S, Wan R, et al. Identification and expression analysis of the apple (*Malus x domestica*) basic helix-loop-helix transcription factor family. Sci Rep. 2017;7(1):28. 28174429 10.1038/s41598-017-00040-y PMC5428380

[50] Gao F, Dubos C. The Arabidopsis bHLH transcription factor family. Trends Plant Sci. 2024;29(6):668–80. 38143207 10.1016/j.tplants.2023.11.022

[51] Zhao F, Li G, Hu P, Zhao X, Li L, Wei W, et al. Identification of basic/helix-loop-helix transcription factors reveals candidate genes involved in anthocyanin biosynthesis from the strawberry white-flesh mutant. Sci Rep. 2018;8(1):2721. 10.1038/s41598-018-21136-z. 10.1038/s41598-018-21136-z PMC5807450 29426907

[52] Khan I, Asaf S, Jan R, Bilal S, Lubna, Khan AL, et al. Genome-wide annotation and expression analysis of WRKY and bHLH transcriptional factor families reveal their involvement under cadmium stress in tomato (*Solanum lycopersicum* L.). Front Plant Sci. 2023;14:1100895. 36760632 10.3389/fpls.2023.1100895 PMC9905835

[53] Carretero-Paulet L, Galstyan A, Roig-Villanova I, Martinez-Garcia JF, Bilbao-Castro JR, Robertson DL. Genome-wide classification and evolutionary analysis of the bHLH family of transcription factors in Arabidopsis, poplar, rice, moss, and algae. Plant Physiol. 2010;153(3):1398–412. 20472752 10.1104/pp.110.153593 PMC2899937

[54] Jia M, Munz J, Lee J, Shelley N, Xiong Y, Joo S, et al. The bHLH family NITROGEN-REPLETION INSENSITIVE1 represses nitrogen starvation-induced responses in *Chlamydomonas reinhardtii*. Plant J. 2022;110(2):337–57. 35043510 10.1111/tpj.15673

[55] Panchy N, Lehti-Shiu M, Shiu S-H. Evolution of gene duplication in plants. Plant Physiol. 2016;171(4):2294–316. 27288366 10.1104/pp.16.00523 PMC4972278

[56] Jiang Q, Wang Z, Hu G, Yao X. Genome-wide identification and characterization of AP2/ERF gene superfamily during flower development in *Actinidia eriantha*. BMC Genomics. 2022;23(1):650. 36100898 10.1186/s12864-022-08871-4 PMC9469511

[57] Qian Z, Rao X, Zhang R, Gu S, Shen Q, Wu H, et al. Genome-wide identification, evolution, and expression analyses of AP2/ERF family transcription factors in *Erianthus fulvus*. Int J Mol Sci. 2023;24(8):7102. 10.3390/ijms24087102. 10.3390/ijms24087102 PMC10139229 37108264

[58] Li D, He Y, Li S, Shi S, Li L, Liu Y, et al. Genome-wide characterization and expression analysis of AP2/ERF genes in eggplant (*Solanum melongena* L.). Plant Physiol Biochem. 2021;167:492–503. 34425394 10.1016/j.plaphy.2021.08.006

[59] Hoang XLT, Nhi DNH, Thu NBA, Thao NP, Tran LP. Transcription factors and their roles in signal transduction in plants under abiotic stresses. Curr Genomics. 2017;18(6):483–97. 29204078 10.2174/1389202918666170227150057 PMC5684650

[60] Liu W, Stewart CN Jr. Plant synthetic promoters and transcription factors. Curr Opin Biotechnol. 2016;37:36–44. 26524248 10.1016/j.copbio.2015.10.001

[61] Hrmova M, Hussain SS. Plant transcription factors involved in drought and associated stresses. Int J Mol Sci. 2021;22(11):5662. 10.3390/ijms22115662. 10.3390/ijms22115662 PMC8199153 34073446

[62] Yin J, Chang X, Kasuga T, Bui M, Reid MS, Jiang CZ. A basic helix-loop-helix transcription factor, PhFBH4, regulates flower senescence by modulating ethylene biosynthesis pathway in petunia. Hortic Res. 2015;2:15059. 26715989 10.1038/hortres.2015.59 PMC4680862

[63] Jung YJ, Kim DH, Lee HJ, Nam KH, Bae S, Nou IS, et al. Knockout of SlMS10 gene (Solyc02g079810) encoding bHLH transcription factor using CRISPR/Cas9 system confers male sterility phenotype in tomato. Plants. 2020;9(9):1189. 10.3390/plants9091189. 10.3390/plants9091189 PMC7570381 32933074

[64] Wang R, Sun Y, Liu W, Chen X, Xu J, Yuan Z, Liang W, Zhang D. TIP2-UDT1-OsUPEX1/2 module regulates tapetum development and function in rice. New Phytol. 2025;247(2):651–68. 10.1111/nph.20435 40016947

[65] Feng K, Tan H, Zhou L, Shi T, Wang L, Yue Y, Yang X. A novel bHLH transcription factor LlbHLH12 negatively regulates anthocyanin biosynthesis during Lycoris longituba petal development. Hortic Plant J. 2024.

[66] Szecsi J, Joly C, Bordji K, Varaud E, Cock JM, Dumas C, et al. BIGPETALp, a bHLH transcription factor is involved in the control of Arabidopsis petal size. EMBO J. 2006;25(16):3912–20. 16902407 10.1038/sj.emboj.7601270 PMC1553195

[67] Sun W, Jin X, Ma Z, Chen H, Liu M. Basic helix-loop-helix (bHLH) gene family in Tartary buckwheat (*Fagopyrum tataricum*): genome-wide identification, phylogeny, evolutionary expansion and expression analyses. Int J Biol Macromol. 2020;155:1478–90. 31734362 10.1016/j.ijbiomac.2019.11.126

[68] Sasaki-Sekimoto Y, Jikumaru Y, Obayashi T, Saito H, Masuda S, Kamiya Y, et al. Basic helix-loop-helix transcription factors JASMONATE-ASSOCIATED MYC2-LIKE1 (JAM1), JAM2, and JAM3 are negative regulators of jasmonate responses in Arabidopsis. Plant Physiol. 2013;163(1):291–304. 23852442 10.1104/pp.113.220129 PMC3762649

[69] Chen X, Huang H, Qi T, Liu B, Song S. New perspective of the bHLH-MYB complex in jasmonate-regulated plant fertility in Arabidopsis. Plant Signal Behav. 2016;11(2):e1135280. 26829586 10.1080/15592324.2015.1135280 PMC4883827

[70] Hu J, Chen X, Zhang H, Ding Y. Genome-wide analysis of DNA methylation in photoperiod- and thermo-sensitive male sterile rice Peiai 64S. BMC Genomics. 2015;16(1):102. 25887533 10.1186/s12864-015-1317-7 PMC4367915

[71] Feng S, Ren L, Dai S, Wang H, Zhang F, Zhou A, et al. AabHLH48, a novel basic helix-loop-helix transcription factor from *Adonis amurensis*, promotes early flowering in Arabidopsis by activating FRUITFULL expression. J Plant Physiol. 2024;297:154256. 38657393 10.1016/j.jplph.2024.154256

[72] Nakata M, Ohme-Takagi M. Two bHLH-type transcription factors, JA-associated MYC2-LIKE2 and JAM3, are transcriptional repressors and affect male fertility. Plant Signal Behav. 2013;8(12):e26473. 24056034 10.4161/psb.26473 PMC4091362

[73] Capovilla G, Schmid M, Posé D. Control of flowering by ambient temperature. J Exp Bot. 2015;66(1):59–69. 25326628 10.1093/jxb/eru416

[74] Yang L, Li J, Ji J, Li P, Yu L, Abd Allah EF, et al. High temperature induces expression of tobacco transcription factor NtMYC2a to regulate nicotine and JA biosynthesis. Front Physiol. 2016;7:465. 27833561 10.3389/fphys.2016.00465 PMC5081390

[75] Bohn L, Huang J, Weidig S, Yang Z, Heidersberger C, Genty B, et al. The temperature sensor TWA1 is required for thermotolerance in Arabidopsis. Nature. 2024;629(8014):1126–32. 38750356 10.1038/s41586-024-07424-x PMC11136664

