# Supplementary material for: Comprehensive analysis of bHLH transcription factors reveals candidate regulators of flower development and heat stress response in Rhododendron simsii
Source: BMC Plant Biol. 2025 Dec 8;26:69. doi: 10.1186/s12870-025-07868-x (PMC12797679; doi:10.1186/s12870-025-07868-x)
Supplement: Supplementary file 1 — Supplementary Material 1: Supplementary Figure S1. Chromosome localization of RsbHLH gene family. Supplementary Figure S2. Sequence logos of RsbHLH proteins. Supplementary Figure S3. The Spearman’s corrlation coefficient of RNA-seq data and RT-qPCR. Supplementary Figure S4. The expression patterns of species-sepcific RsbHLH genes in different organs and in different stages of flower development. Supplementary Table S1. Protein sequences of bHLH genes from A. thaliana, R.simsii,R.williamsianum, and R.irroratum. Supplementary Table S2. The promoters of the bHLH genes in R. simsii.Supplementary Table S3. The primers of 12 candidate genes used in this study. Supplementary Table S4. Coding sequences of RsbHLH053 and RsbHLH059 cloned for GFP fusion and subcellular localization. Supplementary Table S5. Physicochemical properties and subcellular localization of bHLH protein in R. simsii.Supplementary Table S6. The distribution of RsbHLH genes on each chromosome in R. simsii. Supplementary Table S7. The classification of the bHLH genes inArabidopsis, R. simsii, R. irroratum and R. williamsianum based on the phylogenetic analysis. Supplementary Table S8. Conservative motifs of RsbHLH protein in R. simsii. Supplementary Table S9. The promoter Cis-element functional classification of RsbHLH genes. Supplementary Table S10. Segmentally duplicated RsbHLH gene pairs. Supplementary Table S11. One-to-one orthologous relationships between R. simsii and other plants. Supplementary Table S12. The expression patterns of RsbHLH family genes. Supplementary Table S13. The expression patterns of RsbHLH family genes in different stages of flower development. Supplementary Table S14. The protein interaction network. Supplementary Table S15. The RT-qPCR data of 12 RsbHLH genes in different stages of flower development. Supplementary Table S16. The RT-qPCR data of RsbHLH genes in high temperature-treated R. simsii. [file 12870_2025_7868_MOESM1_ESM.zip › Supplementary Materials/Table S1.docx]

**Table S1. Protein sequences of *bHLH* genes from *A. thaliana*, *R.simsii*, *R.williamsianum*, and *R.irroratum*.**

>RsbHLH005

MNTGLFSSNPKHPDVQNMKGRESMNTDDLYNQYQNQQQGSALVRYRSAPSSFFASLIDDGGEEEDLLNRQSSSGSESIFDVTAEKKQYSSIYETSLDRRDLVNQNESWVTSTCGGDGGGDLRGFYYKDDVAMENNSIMEHGKRSGGGAVGGGSSNCSNLIRQSSSPAVFFSALNADTAWRGGYPGRWADVLKDVAKALGALGKLESLSEIGKQNLALAAAVVDDETPTPNESTSPKAPKAFATSLIAFALRPRCPQHHARKKRMSPSTKTCPNIFEPDDWIVDSRTRTMGPDGWRRMRRRRVLYSIDHAPNLYNWLLKNLNKFAGFGDLKNVGSFRAGNGTNGKVSTSNRFNGIINFSSGPSSSSRFMPKITESGNGNGTIEASSPQTRFQNDSWTVTGLKRNRDDNESQLFGFNALNGDSSSYATGLTHHLSLPKNSAERDAVEEFLQFQQDSVPCKIRAKRGCATHPRSIAERTRRTR

ISERMRKLQELFPKMDKAITNSSLSVIYWQQTSTADMLDWAVEYIKDLQKEVKTLTDKGS

KCTCSGEGKQVPNPTL

>RsbHLH002

MSTPKADRASVVGDAIGYIKELLRTVNELRMLVERKRCSIERIKRHKTESDGPDHQDQSS

YNGLRSSWLQRKFKDSEIDVRIVDDEVTIKFAHPQKKINCLLPVSKALDELQLDIQHVGG

GLVVDNYCFLFNSKILVEKESLEIQEFSEYTKRSPSLNCSTLVSERRGDEISSVPCRAFP

WLNLLSTFLVFQEINIQGFCLYSNFGIAQNCFTRFLHLFDPPIEGYSTKLIKRGSYWAVS

QELSKTEKLNGANFSTWKRRIRHILFHDKVEYVIDIDDVPTPPPENSNAANRRMYEKFLE

DDKTARHIMLTFMEPDIEILFEEYAHAKTMFDAISNAYRATSETYIQLLMERFTGTEMKE

DENVIDHVNKLSVIAKELATLDNPLPDRLQVSTILQSLPKSWESAVVALNFSSTTLTMKN

LPVLLGIEAERRAKKKTTKSFLTLAPAATFEPPQFENKKFHSSSGSSKSGQQKNYSKNFK

GKGKEFKNGGNFKKKGSCFNCGKFGHFQADCRGPKNNKKPNFGGSKDIVCVVSESLLADTDIGAWWVDSASSRHVAKNKESFVELKEVKAGDHRIYMGNNSYCDVVGIGTVKVMLPGDKNLYLTGVLYSPTMRRNLISVPRLDEKDFEVRFRSGKVSIGKHGRIMMWGSKVDGLYRLNIVSDVNNNALAGCSAYIVDSTHSYDSLYVDDPYIWHLRLGHINKDKMKRMMNMELIPKIDIDFSICESCLSGKMSRLPFPKANKPIAIMCDNQAAIQTIKNGEIGSRGKHIDRQYHYVVDVLQRNEIVIDYLSSKEMLADPLTKPIASVDFRKHVHLMGVKSD

>RsbHLH001

MYQENAMRLPENGFLQTVPNFTPTFSSIEELSFYQNSQQEASAGMEIEELLHHMGFERNT

HLTQEMILESNLFHLPNLSFSYPDQAQNSPSFLPTLGFLGDVSSNTHSALDSNSANFDPL

FHLNLPPPQPLFRGLMFESPIPNGGYNMTTGSLFGGVVDEREGSGGGYGYDNGVFEFNGG

DWICRGRKSNGKGSNRQLNTEKQRRGQLSDKYEVLRNLVPNPTKVSSMASGSSSSSTSTE

SSSSSYLPASLSFLIANFQSFITLKLETSNYFAWKTQVENALRATCLSEYIDGSVVIPTS

HIVDTSGNTVPNPEFSRWNTVDRMLLSCLIATLTPPILPHVVGSDHTFQLWLKLEEKFSV

LSRSHIHDLKRKLYSLHKTGTMETYLDSIKEIVQKLAASGTQIDDEELIFHTLNGLKKGY

KSLKQMVRNTTEPLTFSGVSSMLMVEELHISQDQVDSSSTILVAPHQNPNPTPILQAPMT

PNPSSSSSGPMPFPNTAVQTPSGSTSFQFPFPVASSQAFLPTFPQPTFNGQRFNRNNNRF

RGNQFSKPFFPNYQGSSTPQMFSPGSCQICGKPNHQASTCHHRQNLGYRPFARPFGQFGN

HFGNHFGQQTGIFPSQNPPGFQSQNSQAFYVGSGPINWYGGPSAVGQSEFALHTSSPYNG

GSYPTTPYAPPSFSQFGDNSASSFGSAQFGASVVGDAIGYIKELLRTVNELKMLVERKRC

SIERIKRHKTESDGPDHQDQSSYNGLRSSWLQRKFKDSEIDVRIVDDEVTIKFAHLQKKI

NCLLPVSKALDELQLDIQHVGGGLVVDNYCFLFNSKICEGSSVYASAIANKIMEVVDKQY

LAIPTN

>RsbHLH007

MDELIISPSSSSSFISSQETPPTLQQRLQLILQTQPDHWTYAIFWQSSSPNDQNTPALLS

WGDGHFQPTNNKNPLPKDNENSAEWFYIMSLTQSFSGAGDRGAPSKAFSSGSLVWLTGGNQLKFYDCERAKEAQIHGIKTLAFVPTSVGVLELGSSDVVKENWGLVQQAKALFGSDLDVIPNHGNGSSEFLDGNVCFAEEDMVNGGGTKQEAIVETECSDLGPFPVPTTFGFHHNGNAEPEQQQRTQKKRGRKPGVGRETPMNHVEAERQRREKLNHRFYALRAVVPNVSKMDKASLLSDAVSYINELKTQIQDLKSQLHDSKSSNKKAKTELSSQLTADTTDNHSSAATSVDQTISANNNSDNTSCGVQLEVEVKIVGADAMVRVSSDNVNYPAARLMEALRDLELQVQHASVSSVNDMMLQDVVIMGPHRGMISEEGLRTALLSRLG

>RsbHLH003

MEGRLQGPINPCFLEEEEEEHLDVDMECLEQETSNNTIRKLGFGSPSDSSFEEMRIPFLE

MLHGGDSPAFSPFCELSFQALLSLQQFKKPPWEGSHSHHHHHHQYSPELTECSGGGRFKG

QQDCLTHDAVEVQISPVKSETMDLRNTHSAIYVEGGANSDGNQHDGSKAVELEERPVEAAAGKGQKRKRVKRTRPSAKNKEEVESQRMTHIAVERNRRRLMNDHLTALRSLMPSSYIQRGDQASIVGGAIDYVKELEQLHQSLQAQKRLKKSDLLDQSGGCGGSSETTSTSTSSSSSTTTGILATSSPQTCQLGNMKSEIGELGNCTTEGINVRRSYEFTAVRKSGGVEVHVTVIQNHVN

LKVECPRRAGLLLNAIVALEDLGLTVLHLNIMALENSAQYSFNLKIEEGCEIGSADAIAV

VVLQIFCFIHRTT

>RsbHLH008

MNHLVPDFEMDDDYSIPSSSGLTRPKKSPMAEEELMELLWQNGQVVMQSQNQRSLRRSHIGDGVNPPENTAAAKEIRTAEESATQLFIQEDEMASWLHYPIDDSFDRDFYSDLIYPAPTA

PVAPIPDDVRVSTPPAAATAAAPRPPIPPPARRPEVESPARTQNFLHFSRPKKGRTEAGP

SICNKAARDSTVVDSSETPAPGSTVSHAAARVSGGNVWYGAAASGAAAVASSAAGEETGLASSSGGSGASVSASAEPVHKPVLAAVTEDRKRKGIEADDVECQSEDVEFDFPDAKKQARGSTSTKRSRAAEVHNLSERRRRDRINEKMKALQELIPRCNKSDKASMLDEAIEYLKSLQLQVQMMSMGCTMVPMMFPGVQQYMPPMGMGMGMGMGMGMGMEMGMNHPMMPFPSVPPGSALPTAAVNARLGPTFPIPTFHIPQPVPLPDPSRIQAPSQSDLMPNSMGTQNPNPPRMPSFADPYQQYRGLHHMQLPLPQNQAMMQPSPSKPNSSREVETHQNLQSGMPITPVVWFD

>RsbHLH004

MGEKFWLNEVDKGMVESVLGSEASEFLVASASGNVLSEFASSAGDLGLQKGLSKIVEGSN

WNYAIFWQVSKSKSGNSALIWGDGNCREARGSEVGTGNGSQDRKYEGGDRKKRVLQKLHACFGGVEEDNCALNLDLVSDVEMFYLTSMYYSFPFDKPSTPSQAFNTGRVIWASDTKSCSEHYQSRSYLANLARFETVVFVPLKSGVVEIGSIKTVPEEQNLVQVVKAAIGGPLPSQGKLVPKIFGHELSLGGTKSRAMTISFSPKVEDDSGFASESYELQGVGSNQVYGNSSNGCRSDEG

EAKLFPHMNLGGLEQGHEDLLIQSDERKPRKRGRKPANGREEPLNHVEAERQRREKLNQRFYALRAVVPNISKMDKASLLGDAISYINDLQGKIRILEAEKDMVNYKQKQGSVPEIDFQA

RHDDAVVRVSCPLDSHPVSRVIKAFREHQVIAQESKVSTTEEGEIVHTFSIRAQGGTAEH

LKEKLVTALSR

>RsbHLH009

MANNPSDGPTDDFFEQILGFPAYAPTDANLAGNSGTPMMLQLSSGDGSAHLGGGGGGGGGGGFHGSVFPLGLSLDQGKQGFREDVGDGRSSSSSMKNTFSGQPMPNTVNAMAHPPSIRPRVRARRGQATDPHSIAERLRRERIAERIRALQDLVPSVNKTDRAAMLDEIVEYVKFLRLQV

KVLSMSRLGGAGAVAPLVTDIPMSSLEQDAGESGRNQAAWEKWSNDGTEKQVAKLMEENIGAAMQFLQSKALCIMPISLASAIYHTQPPDATTVVKSESHPPS

>RsbHLH006

MEGKYGHENPLQLMSSAFGGTSNDASDSYQGNPNHKYVTADGKNHIPPVPSPLSQWIHCQETSPGSVEFITERSVTEISREMSYHNAIVPFGGTDVWKAGGNAVLQEVKYARPENWPPMNSFTDAEYQLHAQGQESNELHSRASVAAPAFLAKPHNHASRRRASVTAAATDRARRMRITERHQALNELLPRHKEGSKASQLDDITDYIKYLQLQIKVCFHYNVVAYHHVKDLSRSRLGGEPTTDPFVFLEGYGHYILHEQMLNEPLEEMMGKLLEVNPSATSRLLESRGLVIMPTNLVEG

LNQAM

>RsbHLH013

MLPSCGKRRERAGDEEDHKEMIIMEKSVIHPLMAAIDGKGSSLKAGRAKLGRVESIHKER

ERRGKMAEMFSVLQSIVPNIFPKATRENIVTETIQYIQRLQEERDRLETLKKSQESTAVM

PTLTQCTNRRDSAVNVTVSGNGVVFFGIRTATNHRHSAVEILGVFERHEAEVLAASVSVN

GQQRRMDLTVTAFVGGNEDVVEKIKRELLNL

>RsbHLH012

MANNPSEAYPDEFLEQILAMPSYSSLAGTDGASSQSAASPLSFTGGMGLQHPFIPLGLSL

DNGREEINGGGFAGKSERESMNMGSLFPVFEHLQPHSVRQSVSHQVFHGQPTTSTTVSVP

HPPSIRPRVRARRGQATDPHSIAERLRRERIAERMRALQELVPSCNKTDKAAMLDEILDY

VKFLRLQVKVLSMSRLGGAGAVAQLVADIPLSSIEGDNSGGCNQNAWDKWSDHDVEQEVAKLMEEDMGAAMQYLRSKALCVMPVSLAALIYPTLPPDAPELVKAERPEPNGPS

>RsbHLH015

MDEVFDRYYQLAGNIFAAEDTFWEGEEEVFPADQSAFARHEEQNGRSPLHNREKKNRVNEVFQYYQSPGNILATEDTFWVGEEVFPASQRAFVGHEEQSGRSPLQNRMDEVFQYYQSAGNTFATEDKFWVGEGVFPANRSAFVGYKAGRAGGGSRGKTLGAKRNYQNVNKRMIEFMRRRWIPVAEGEEYERERCRRHMINERMRREKQKNSYSSLRSLLPPRTKSDKNSIIQEAAKEVKELQRHREELDRRNREIEGILAERERGKKNEGAKIKLRVANPTSGVDSMVEVLKCLKNMGSKTGAVQSNFSAQEFSAKEAAEVEKVVQRTLFEVERKLLFHFPN

>RsbHLH016

MIEHKRSPISIDQSSHTAFAPKRHKADLSISSKETKEKLGEKIAALQPLVSPYGKTDTAS

VLLEAMEYITFLHEQVTVLSAPYLQSIPTANMQEIEPYSLRSKGLCLVPISWTSGVDSSN

GADIWAPIKTTSPKY

>RsbHLH011

MNEAGNSNGNSGRRRAHIDDDDDDYEIDDRVHKSKNLDAERRRRKKLNDRLLELRSLMNKGTIVTDAITYIEELEKSATDLRNQLLEMEATVVEESKLPIEAIDAAAEETMNWGIEPEIK

VNQIDRNKVWIKMVFQKKRGGFTKLVEAVSVIGYEFTDTSVTTSRGAILVTACLEVRLKY

TFALFQH

>RsbHLH014

MEQNPRADRKTIEKNRRNHMKALYSKLHSLVPHRPSMVSLSLSLSMFLSEAMSLPDQLDE

ASTYIKKLEKELEEMKEKRDSKMMRAGKNPNSRMMSTSSSAGMMIRSPQIEIHEMGSALEVVLITGLDCQPIFNEIIRLIHEEEAEVVNASFSTVGDTVFHTIHCKVGESSGFACGAARI

AEKLKKSFYDAGPF

>RsbHLH017

MASVPENLTKQLAVAVRSIQWSYAIFWSISSRHPGALEWGDGYYNGDIKTRKTIQAVEFN

AEQLGLLQRSEQLRELYESLAAGESSPQARRPSAALSPEDLTDTEWLPGRTLSKGQPIWV

CNAHYADSKVFSRSLLAKTVVCFPFLGGVVELGVTDLVLEDPSLIQHIKTSFLETPSPKI

SKISNSFTGNANQGLIVHAKLDHEILDTNLNPVFQCEDFEVCSPNNSSNCFGINPQPEES

FLVEGLYEANSQVQSWQLMDDEVCNGVHNSFSSSDCISQTLVNPEKASPLPDEMTKIGLQ

ANDIHYQSVISTLLKSSQHLILGPNFRNSSQESSFVRWKKGDLSSIQTPRSRTPQFLLKK

VLLEVAQMHGDHMSECSREDSGRKDAIWRPQVDDIDSNHVLAERRRRENLNKRFSVLGSLVPSTSKVDKVSILDHTIEYLRELERRVEELDSGREVSEPEVRTRKTPKDSAERTSESYGH

NKKPSINKRKVRDIDEIKDPYNTTVSIIEKEVLIEIRCPWRECLLLEIMDAISHLHLDSH

SVQSTNSDGILSLTVKSKHSKMYRIQQPFELSFWVVAGRTTGCFRSVIRPKLSEISLVGF

VGAKNVGWRSLVLVLLPGDLVAGGDLGGVNGME

>RsbHLH010

MMAARETQINRVEAERLRREKLNHRFNELRSVVPNVSKMDRASVLTDAVAYIKQLKAQREELEAQVRVESQTAAKACVASTSTAGGHHAVPSLGEVEVMIVGSEAMIRVQCVDANYPCARLMEALRGLEIEVYHASMTKVKEIMLQDVVVRVPKGLTSDPEALTTAIQRRFQI

>RsbHLH021

MNNQDEYRNYQNNHNHSNQSNSNSGLLRFRSAPSSLLSNFNSVGTDYGEKSSSPENVMHDLEESKPFANGYCLSSSQLPPQYPRQSNTSSYGGVVGSNHLMRQSSSPAGIFCPQNGYASL

RSFGNYRVGNGTNDGDVSPSSNRLRSPMNFSPGVHSSLGILSQISELEDESVVRSDIKLG

NGSREPQFFPFGSWSESSHFPENYSLMKRDLDYDGKLFGSTQNGELEHRAHNFSHHLSLP

KTAAEMERLLQFQDTVPCKVRAKRGCATHPRSIAERVRRTRISERMRKLQDLVPNMDKQT

NTADMLDLAVEYIKDLQKQYKILNEYRANCKCSSIQKAFANLSA

>RsbHLH023

MEGNSGTDSRSAENGVESLQLGEEIQRLVMAPETGNSFTALLELPPNQAMELLHAPGGND

ETPPPFNRLLRSLDSTPTFPSNAVLLERAAVFSVFAAENSSETSLIPVNSVANSRERVKR

EPVDTDSNPNSSDPNSNQKSTKRKEREKKGKGLTKKSKSKANETSDDSEKLPYVHVRARRGQATDSHSLAERARREKINARMKLLQELVPGCNKHLHLCTEEKIALQISGTAMVLDEIIN

HVQSLQRQVEFLSMRLAAVNPRIDFNLDSLLAAESESPVDSNFPGTVMPLMWPDSQVNGNRPHYQQIWHFDGLHQPVWERESHNFINADNSLLSYDSPTNSASLLSNLLKMEL

>RsbHLH019

MDTMECKAGGSKDTSFVVPWPPCAESVSSFQCHSYDPWSMRFGGVQEDISGASASRSHSQAEKRRRDRINAQLATLRELIPKSDKMDKAALLGSVVEHVKDLKRKALEVSKTLSVPSDVDEVTIDCFSDVQDGSSTATSTNNIYIRASVCCDDRPELFSELTQALKGLKLKTVQADVASLGGRIKSILVLCSKDNKEVACLNTVVQSLRGVLSRIAGSSSSMGSSYRMKSKRQRFFLRSH

>RsbHLH025

MFHRANFTTPSKEEEIAQDLITGIAILESGNHARNSFGKRGKRQQDSPAVRSGDNDGSGE

GNIKERKAVHRDVERQRRREMANLFASMRSLLPLEYIKGKRSASDQMHEAVNYISHLKNNVKELEIKRDNLKKLSAGSSTTTATGTGSIRSSADNRHRSSSVTVSQCRGGVEISDLMYID

LFELEQKLSGVINGELKL

>RsbHLH024

MFSLQQGDELVFQISSIEQDLISSHASLETTGILTPPKRKRKKSLLYSNHDHQQEDKYGG

SSTCTTTGSNGNKEKIKMTAHRDVERLRRKEMAELYASLRSLLPLEYVKKNINELSIKRD

RLKNVANSTANTEGSTESCSSGRVTASLCCGGVEILINSGFEEVGFPISRVLEILVEEGL

DVVSCVSTQMESKLLHVIKSEVISDPTCFDLSALEEKLAEVIIGIGREDEIEQDLAMGNA

VVESSNLARKNVGKGGKRQQNLPAAAQPGDNDGSGKGNKERRMVHRDIERQRRQGMANLFASMRSLLPLEYIKGRRSASDQIHEAVNYINHLKNNVKELEIKRDNLKKLPDSVSTSIATVSTSSVTVTVNQCRGGGVEVLIGSGCGFAEEGGLPLSKVLGVLVEQGLDVVSYVSSDVEGRLFHTIRCEVSDLMYIDLFELQQKMSVVINGEQNLQL

>RsbHLH020

MFSLQHGDELVFQISKIEQDLISARASLDSTHLISTPPAGKKKRKKSYSNQQKDKYENNN

EKKMIAHREVERQRRQEMAKLYASLRSLLPAEYVRGKRSVCDHMNESVNYIKQLQENIEQLSMKRDNLKNMSDSIGNNTEATGSSENCFPGRVTVGLCWGGVEILISTSTGSEDEGFPMS

RVLEILAEEGLDVVSCVSTQINKKLLHVIKSEVSDPTCVDLSILEEKLADVINGN

>RsbHLH022

MSSFEENDDLWFQIYSNYPDQEKTTQLQDQSKNDVPENFGERQPVPDANDHTCFNKRVIR

REIEKQRRKKMAALFSSLRSLLPPELVKGKRSVGDYINEATKYIKHLEKKIKDSGAKRDQ

LQILSSSSTATTSCSHGNGSSSNCSANYVTVCCRSMGQVEVILSTGGGFDPAGKLQLSSV

LKLLLEEGLSVVNCVSTKLNERLLHTIQSQVLQV

>RsbHLH026

MFMDYIFLLDDGARPTFLQNLVHSSGCTYICLWRSYLPQRSNSWLISSLDGFYNEENIRQ

PSSSSGSHARRLFDEYRRSVVAVDNIDRIPGLAFRNRAPFMELKELELRRLASVEPQLQF

YLVIIFLEARIKTAVFMGCTAGEIELGFSNDTTQVNWEMEMRKWMPENFLDQLVPYQDLP

PPPDRQTRASSSSSSLRSLSTDNSLEPSPFLFNIPNPCNYHQEPPKQPPAHEQAFRPTSS

SVLLSPLHQAIQSFNQTRNFPFPTPETEEEAITKAILAVLSSPSPSASPSASSPQPSVAQ

NFPSFGRNATSAFNRYRPGFAHSSSTPMIARVRRSSMLKRAISFFRSLSSMRNLGRVQVH

GGSRPTENQVYHMISERKRREKLNESFQALRSLLPPGTKVTRNSSSITILTVKDKASVLS

GTSEYLESLRSQVVELTRRNQVLEAQLLPKTKPLDDQEGSDHESSYRRQDVKVVNVVAAEPSSSGAQIVDLFVVVRGGGCDILDLMIGILEFLKNDERVGFISVEADTKVEESISVNRVV

SRLKIEGNEWDESTFQEAVEKVVADLAK

>RsbHLH018

MENIGDEYKHYWETNMFLQTEEFDSWGLDEAFSGYYDSSSPDGAQSSAASKNIVSERNRRKKLNERLFALRSVVPNISKMDKASIIKDAIDYIQELQEQERRIQAEIVELESGKMKKKNS

SVYDLEEEMPGLLRSKKKRTTDQSFDSRGSRASSPVEDLELRVSYMGEKTVVVSVTCCKR

RDTMVKLCEAFESLKLKIITANITAFSGRLLKTVFLQADEEERELLKVKIESAIASLNDP

ESPVSF

>RsbHLH028

MDSNPSLSLSLHIYAYTYGIVFTVAFRNSSEEMERGFQALLLGGESSDDDYSPPAFIGTT

AGDACISALYSSAMNAQTSKSFLSDEKYLLQSLGYGYGAEPAAPSVDPVACGYESYDVVG

DWKLKSVGYADEKLEMPKYETVEYESVSDVPEPPFDFLSKSSGSVSREFDSRFSQLPGMN

SQTSSFLSLSDEIYLLQSLEKYGYGTLPVAYGYDVLEDSKLKYVDYADDKLKMSKYETVD

YESVSDVISTEPPFYFISKSSGSVSGEFDSRFSQLPAMKAQTSSFLSISDEKYLLQSLEC

GYGALPVAPSVDKSVEYVDGKFKVPKYEPVDYESLGELVSAEPPFDFISKTSGSNSGVFD

SRFGKLPSLLWLEPVRDSFGLTASPKKRVRSLAPATSNNSYFTGSIPPEIELMKARKRRQ

RISEKSSCLQKLLPWDKKMDRATMLEEAYKYVKFLQAQVSVLQSMPVDSSFPTRNPTGNGAVNVFGGLGRLNRQQLLQVMVNSPGVQTTLYSKGLCVYSVEQLVTMKKVAERILR

>RsbHLH029

MKSSSLEFDEHVDESTSTNPPKSGTCKPTPDDDPHEYDSSSSESSTTSTAITRKLSRKRR

SKKLIRQFRELSAIVPEASNKEVMLMDVKEASSILEDAIKYMKQLEERVNKLEEELTRPN

HGSYSDEEEEEDRLPEIDVKTDGQDQQNVLIRIICEKRKGQIARIMSEMEELHLSVSSIS

ALPFGDQLLVISVSAQMGNEFCMTANDLKKNLRAGILKFMRHV

>RsbHLH027

MANNPPDGYGDDFLDQILAVPSSYSTLTDATAASQLSSTAGGGGGSGFQQQQQFVPLGLS

LDNGRDDFHSGGFGVKSERESANMGSFFPAFEPLQTHSFRQSAPQFHQAFPGQAAPSTTT

SMPQPPGIRPRVRARRGQATDPHSIAERLRRERIAERMRALQELVPSCNKTDRAAMLDEI

IDYVKFLRLQVKVLSMSRLGGAGAVAQLVADIPLSSVEGDNSEGECSQSAWDKWSNHDIE

QEVAKLMEDDVGAAMQYLQSKALCIMPISLASAIYPTHSPDGSMLVKPEPNGPS

>RsbHLH034

MKVSSLPNLNRETTSFPNFFTIIQDPADHHHHHDTGLQIDEHSCDTEASETSPYKAILSP

SVSFDQTNEIGFPAGTSSVGMFRSESEHNDQTRPNSVSVGSNQFGGSEWPNLPSCGGNRA

TSPISQETIEQPFSPLCPTKRAFTQIDTFQFLEIKEATMTEAGTSKSSRRETPAPLPPFK

GHYFASHLVRKERLADKITALHQLVSPFGKADTASVLLEAMEYIKFLHEQVNALSIPFMK

SGASMQLQQNPGKSKEDEGPKQDLRSKGLCLMPISNSLPTDLEIATTPPSTCAQIDERSC

DTETSPYKDILSPFDEMNEIRLFAATSSVGLIRHESVQTQHNTSSVGSNQFGGGSATSGS

VGPNPAQLRELDPVINNKDTMLRLAIPVRQRVAVCIWWLATGEPLRRVSKKFGLGISTCR

ELVLEVCGAIWSVLMSKFLKRPDEEKMTKVKWEFELISGIPNVGGSIYTTHVPVIEPEVS

APAYFNKSHTERNQKMSYSITVQGVVDSKGVFTDACIGWPGSMSDDKILENSALSQLASG

GLLKDVWIVGNSGYPLMDWMLVPYAHKPLTWSQHTFNVKVGEVQRVAKEAFLRLKGRWSCLQKRIEVKPWRLPVVLGAACVLHNICEMRNEAMDPAETSFNLFDDEIVPEINSVRRRCSGVETLLFPTLSLGLSCFLQILTSPPPRFRFTLQFETPSSNLRTNPKMKVSSLPNLNRETTS

FPNFFTLFQHPADHHHHDTALQIDERSCDTEASETSPYKDILSPFNEMNEIRLCAGTSSV

GLIRRESVQTQHNTSSVGSNQFSGGGSSATSGSVGPNPAQLRWWDQCSHPDFPESEFRRA

FKMSKSTFQFICSELDPVISKDNTMLRLAIPLVSQIIPVRQCVAVCIWWLATGEPLRLVS

QKFGLGISTCRELVLVVCGAIRSVLMSKFLNWPDEEKMTTVKREFELLSGIPNVAGSIYT

THVPINEPEVNGPAYFNKRHTERNQKMSYSITVQGVVDSKGVFTDVCIGWPGSTSDDKIL

ENSALSQRASGGLLKDVWIVGNRGYPLMDWVLVPYAQPIHWCSHMVFNESVGVVQKVAKKAFSRLKGRWRCLQKITEVMLQDLPVVLGACCVLHNLCEMRNEALMDSSETSFDLFDDVIVPENDVRSVDAMRARDQIALWYTIFFWASTFPPFD

>RsbHLH037

MHDLHSCLISTDGLYIGENNQPSSSSGTGSRARRLFDEYRQGLFVLGNDHRVPGLAFRNG

VPYMELKELDLLSLATIEPQLQFYQTAIFMGCNTGEIELGFSNNTQVNLEMEMRSWFPDD

FSRQLAVPQIELPPTDQPRLELPPTDQPRTSSSSSSLRSLSMGSPEASPFLFNIPSITSS

LLEPPIEASSIGQVVRPLSSSTSPLHQAFIQFPSIQFPTPETTETKTTEAGTSQKPSAFK

GFRSALGPSTMTIAAGVRKPNIQKRAITFFRSLSLMRIQGRAQGSRPMSSTQLHHMISER

KRREKLNESFQALRSLLPPGAKKDKASVLASTADYLASLKAQVEELTKRSRDLEEKVQLL

ANKKDHDQEFSLSPDQRLDVRITHEAESTSQARVIGLQVFLRGERNVMDLVTRILEFLRR

VENASLLSVEADAQVVDSNLINIVRLRLRIEGGEWDESAFQEAVRRVVADMAQ

>RsbHLH031

MKATRRRTKYRPKVERTTPVYPEFINALVFEETTKAANRTAEMVASSGGAIDLPLLLCTI

NALFPIRFLITSTFVYASSRELLGEAADYLRPMYVQCYYYGTMNLVQKLLERLRAIVNFK

SWDYCVLWQLADDQRFLDWVDCCCAGSENIQNVGEDLFPVSSVPHCRDVMFQHPIAKPCELLAELPSSIPLDSGFSLSLSLSLTHTHTCAHCFSLKHNYHKKKRPSISKKILLLIPMNLS

NCTTRFYRVYAQALMSNQASWLNFSNNADSKPLEETVGTRVLIPVPVGLVELFVAKQVPE

DKEIVDCIASQCTMLLEQQSMIHSSSMDPNSSMNENNQMDLNNNLFLSSPENSNLPYGVT

TDRMQLHSPMNSFQPFDHASETRASSMAALFDGFNSNGLQVMDSTGQPITMESSSLVLNK

ETDKDSVKHETGRSESISDDSDGNEDEDDAKWRRRTGKGPQSKNLVAERNRRKKLNDRLFALRSLVPKITKLDKASILSDAIEFVKELQKQVNDLQHELEEQSDDEGIKNNGQSEKLNVG

LKCEHEETPDGNNVGAANNGVADPKKQNHDASIYDHKPRQMEPQVEVVQLDGNEFFVKVFCEHKSGGFVSLMEALNSLGLEVTSLNVTSYRSLVSNVFQVEKMDSEMVQADDVRDSLLQITRFGGWPDQMAKTSENGDGMDCHHHHHVHGGNCSNGLHNHQTCSHHLHDLRH

>RsbHLH039

MGKMNLVRKLLERLRPIVNFKSWDYCIIWQLAEDQRFLEWMDCCCAGSENIQNGGEDLFTVSSSVTHCRDVMFQHPRTKPCDLLAEFRPSIPLDSGIYAQALMSNQARWLNFSANNSDSNPLEETVGTRVLIPVPIGLVELFVAKQVKQFSLLICRHDMGRSESISDCSDANEDEDDAKY

RRRTGKGPQSKNLQAERKRRKKLNDRLYTLRSLVPIITKLDRASILGDAIEFVKELLKQV

SDLQLELEEQSGDDEGTENNGQSEILNAGLKREHENTLVGGNNMGAASYGSVAEPTNRKHDTDAYDHKSRQMEPQVEVAQIDENEFFVKVFCEHKSGGFVRLMEALNSLGLEVTNVNVTSCRSLVSNVFQVKKMDSEMVQADYVRDSLLEITRFGGWPAGSSG

>RsbHLH033

MGIKRCKLRLKSPKNLGGLEKRKAYAITQRGVDDPGFVPMWQFNSFGELSTASVADSFEG

NLHHPFTHQMFDVKPCPETPHTGASRPLKLQKTSSWDSSITDLGSNPPANSSPNLVSFSN

SSNYANQFGILKPKEDIQDSFGNQNYVLKASQGAKRISTNTRFPSNQDHVMAERKRREKL

SQRFIALSAIIPGLKKVDKASVLGDAIKYLKQLQEQVKTLEEQVRKQSTESVVLVRKYEL

YADGENSSSGENSSGGPFDEPLPEIEARFCDKNVLIKIHCERTKGVVEKTVAEIEKLHLS

IINSSVMAFGASTLDITILAQMDVEFTMSMQDLVKNLRAALKLFM

>RsbHLH038

MVCQAASQTRFRALKHESGIAGRPTIIVRVIACFQPLQDCQAEYFRALLKPVTFFGWMVK

PEDCWLFQRHSARPLLNLNCTSTPLHQGQQNGSPFSSSTFSPDVAFPGFRVPSSRGPAET

NGAHGLIQFLPPHLDAKEGPSCLYHGHGVKATQNALSESRQKRFVIFDQSGNQTRLFFSP

LFSPGQNPIVTPRKSVGACELGNKGTTVNMEQNFPIKATMQEKYVEDGTIGGRSEMHEDT

DEINALLYSDDYDGCDSEEEDEVTSTDRSPFAIKGCYNKREHEEEITAELANPERPMKKQ

RLIDGGYTKSFTKPAAESSCAKGRNQGRASDSILGNKQSRKDKIRDTLKILKSLIPVVKS

KDPLVVIDEAISYLKYLKLKAKASGVS

>RsbHLH036

MPLSEFYAMARGKVGSVQQKTTRYSTDQSPLRDNEFVELVWDNGQIMMQGQSIRRIPIPN

NFQSQTMKLRDKDPANFTNSKTGKFGTLESGFNDFATAMPSGDMGLSQDDDMVPWWSYPFSDSLQHDYCSELLPELSGVTVNDISAQNSFSSIDKRNSCNQTIRGTTSISGQNGCSLELV

NGSNVSSPEVGKSSRSRSGQFFPWSFQESPAPIPSLRSGVSGVISNDTSNTKHDSCGDSI

QAQLSAGDLSSIKIQKQDSGLPNSSSSFMNFSHFSRPAALVRANLQNVGAMGGENKGTAP

GSSILAEPMLVDLSNGLRNEMGSYSQPNSVPPKVDAKPVVAKPLEEPHAVYQEDSDKNDK

LPNQVLGASASEGMPDGEKTRDPLVACSSVGSGNSAERASNDATHNFKRKYQETDDSEDRSEDIEEESTGVRKAAAVRGATGSKRSHAAEVHNLSERRRRDRINEKMRALQELIPNCNKVDKASMLDEAIEYLKTLQLQVQIMSMGAGLYMPPMVVPSGMQHMHAAHMAQFSPMAVGMGLGMGMNSGSLGWPTIQVPSMQGPHFSAPRPQPILGTTNFQGMGVSNLQVFGHPGQGLAMSSPRAHLMHLPGGPPINLAGGLNANGTAAAVEVPNSAPTTNSKDLLGTTNSQMVHNTDASSSMNQESSQVCELYVMKNSIS

>RsbHLH035

MMAGSPNWFNMMGNMKPPPTPPSVQQPSACLFPQNSFLFPQYMPPPAWQENHEDLPEGGMVGDQEEKPGLSPIIQAKKMENWEEQLVLHQEASNGGAAAEVKQENSSSSSTYVYGHGNAVVDCEGGKPANWSQMMAASSPKSCVTSLSSNMLDFSNKADGRHPPPDRTTATGGALKKARVQPSSTQSFKVRKEKLGDRITALHQLVSPFGKTDTASVLLEAIGYIRFLHSQVEALSLPYLGNGPGNTRPQRSIQGEKNSLFPEDPGQESGEEAKKDLRSRGLCLVPISCTMQVGSDNGADYWAPTFGGGFR

>RsbHLH030

MDRFSYYNQNNTHTNSSNFASVPETHTKQKVGNDKSGKKSRKELKLSTAPQSVAARERRHRISDRFKILQSLVPGGAKMDTVAMLDEAIHYVKFLKAQIWLHENMFSIMDDYCVVDPSLLFSGHGQNDLCWPENVVGGGVELSPPFRLPEVYFQGGEAMGFD

>RsbHLH032

MDHCSYYNQNNTHTNSSNFASLETHTKQKVGNDKNGKKSRKELKLSTAPQSVAARERRHRISDRFKILQSLVPGGAKMDTVAMLDEAIHYVKFLKAQIWLHENMFSIMDDYCVVDPSLLFSGHGQSDLCWPENVVGGGVELSPPFRLPEVYFQGGEAMGFD

>RsbHLH040

MDRFSYYNQNNTHTNSSNFASLETHTKQKVGNDKSGKKSRKELKLSTAPQSVAARERRHRISDRFKILQSLVPGGAKMDTVAMLDEAIHYVKFLKAQIWLHENMFSIMDDYCVVDPSLLFSGHGQNDLCWPENVVGGGVELSPPFRLPEVYFQGGEAMGFD

>RsbHLH050

MDIDFLKSASQDQIDMIMMQMEKLPDFCCGPYTDLGVQFPPGMEFPGGSSSSSSSQGHVL

PFPDNSSPTGMNLPSTLSFSGVQEMSGGRWRGGGGGEFPGGLGFPSQTQKRNSMAAMREMIFRIAAMQPIQIDPEAVKPPKRRNVKISKDPQSVAARHRRERISERIRILQRLVPGGTKM

DTASMLDEAIHYVKFLKTQVQSLERVAANRPAAGLGFPVAVGNGGFVPVAKGFQLGSQGVQQYADG

>RsbHLH049

MADLYNANLHSSPLESEEMTSFLHNLIHKHPFSSPPQPPPVATGLVGRSQAFADSDGRFG

ERDSVMVDSSAGINFSDPGRGFMADARAVYGGDSDGITSMFKRKFLEESDFDEFGSDFTG

PEASEAPVNPALPRSSKRTRAAEVHNLSEKRRRSRINEKMKALQKLIPNSNKTDKASMLD

EVIEYLKQLQLQVQVQPQETFDPRIVVALPHPIPASRRKNVTCKVELRSPVPKEAVLSVT

GDCRGPASVFRESLDVGAGQNHTGVMLSMRNGLSLHPLCFPESVHSALMPQPVLSFDEGNEVLNSNRGQDVFSRNQEISVQAAYDFPNQAPMTSINNSETSFGLEPSMQSHYGPFHHHSKELCHEDGLSQLQLDMSCSVKNSSSGVSS

>RsbHLH041

MGTEENGNMGFQQRNGDSILNCPTSGMNTTNPFFGSGWDPLVSLSQSENFGGSFGNSNYS

HLVHYPSDSGLVEMVPKLPFFGGGGNFSEMVGSFGLPNYAPNEEDGTEKTSTNGGDQQVS

EEGTMGISPCGKRKKRVSESNSPFNPSKNSEGEQQMDISGDSSQEEKKQKSEQNPNANSR

GKQTGKQAKDKSDSGEAPKENYIHVRAKRGQATNSHSLAERVRRERISERMRLLQELVPG

CNKITGKAVMLDEIINYVQSLQQQVEFLSMKLATVNPELNIDIDRILSKEILHSGASNAA

IPGFGPGMSSSHPYPSGIPQGVLSGIPSTSPYHSLPQNLWEHELQSLLQMGYDSNPAIGN

LGPTGRSKLEL

>RsbHLH043

MSDFDTNKWNFGGFDESTFHVPSNFPLEPLDQSLNCSINEFYFPFGGDPFQFSAPGVTDT

PPFPTPDDCSMSMVENEEKPQMGNGVQMLEFQSPNNCKLERIQSTEVPVFGMGLGAERKNRVKKVDGQPSKNLMAERRRRKRLNDRLSMLRSVVPKISKMDRTSILGDTIDYMKELLDRINNLQEEIHEVDTSNQFNLMSIFKDVKPNEVLIRNSPKFDVERRNMDTRIEISCVGKPGLL

LSTVSTLEALGLEIQQCVISCFNEFAMQATCSEELEQRAILSPEDIKEALFRNAGYGGRC

L

>RsbHLH048

MVCQAASQTRFRALKHESGIAGSATIIVRVIACFQPLQDCQVRGRIRFKSYWSSFISSFR

CLYLRCLVYLLMVENLILFCLLEKHQVISDLQLLLYCCVMFSFLILGDCCRWMENDFGSW

FHHQISDRQSPNLNALGAPFDIGLRNTIPAHIIPAHMSPCPNVLPTKENAPAFPFSGVPY

FNAGQQLNEPQGWFYCLPRFRQAFTPAPSSIFKEKLSSGPNPEAGCAQKRFLVFDQSGDQ

TTLMFTPGVGSPPVQCATTFGPKLNSAFDSNNIGLGTERDTRLHLEPILTCEYDENKKDD

TGSEMYHEDTEEINALLYSDDDEYDDDGVSSEDDEVASTGHSPCGMTDDGKQELLEESEE

VASSRGPIKRRKLFDGGYEVEPLMDTATSVKSKICFNLEDDAESSCADSKNKGLGKFGSL

SGNKRSRKDKIRETVDILQSIIPGGEGKDAIRILDEAIDYLRSLKYKAKALGVDTL

>RsbHLH046

MALQTVEYPQDPFGYVGIFDTIMDHGNNIIGANWDSSSSSVRQNVMECWECPNSSPEACT

GDRFFGGEGPPVEAPLTEAVRRQKRRRTKSSKNKEELESQRMTHIVVERNRRKQMNEYLAAVRSLMPASYVQRGDQASIIGGAINFVKELEHLLQTLEAQKRTTPQQQQHPPNNNGLSSP

SLFADFFAFPQYSTSSKNNFIDTSSPAATDKPRPVAFGDIEVTMVESHASLKVLSKRRPR

QLLKLVAGLQCLRLFILHLSVTTADQMVLYSLSVKIEDGCQLTTVDEIADAVNQLLGRVE

EEAAFV

>RsbHLH047

MVVAKAHDYVQEGGPPSHEANTVADVLAKKAAEEQRIFADYIMEFSSGFSWNPVFQEIIR

RGSSSSTSSLVLDGERGELVQAMVRPSHKGVKAEKALEALRNHSEAERRRRERINGHLTT

LRNLIPGTDKMEKASLLAEVVNHLKELRRNVTEATDGMLVPTDIDEVKVEQQEDGSDGASCAIRASLCCDHKYEIWSDLRQALDALHLQTVRAEIATFGGRMIYVFIISCCKEKKMEDIE

GCRLLASSIRHTMRSVLDKFYASEEISSRNTLSNKRRRVPFFESSNSSSIGDLW

>RsbHLH042

MDLLGFQRNFSFYPKYAFVDQPLTDPNDLSLPYLAPPPVLDNSIPQELDQYYQYPYPYPK

CQEIYEDDYNYNYPGETSCLADEFFLNPQPLVPDFSPGLELPLSAQSVAARNRRRKITEK

TRELGKIVPGGEKMNTAEMFQAASKYIKYLQAQLGILQFLASSIQENEEALQIQELKALA

SSPSVQEKLYSLEKCLVPKSLSPNQTSSG

>RsbHLH044

MYRNSMEIDSATENSIWLFDYGLMEDISAIPDGDLPAAPPVTTCFSWPSQEINSCSNVRV

DSFPFPPTEDSQMKGSNQCVDIDSSFGDSEAVEETGLQKRLKSEACNASGTKACREKSRR

DRLNERHALCFFFTHWFVELGSVMEPGRPPKTDKAAILGDAVRMVRQLRSEAQKLRESNEDLQKKIKELKAEKNELRIEKQTMKEGKEKLEHQVKALTSHPGFLPHPFAMPVAYGAHGQTTDHKLMPFIPFPSIAMWQFIPPAVCDTSHDHVLHPPVA

>RsbHLH045

MKPSHKNKESLRGAKKNLKTKTPQRKREREREREREREEKSTSDLIKGSSTFLAMADEFP

IGGANWWESSRNRFESGSSASSSAPLTSNLLVDPNLQMMDLSLSSQAMDWNQPLLRGGKAESSFRSAIQEDLNSSASFRSEIGMESQANEYKQINRGFLLDQPQLMDQAAYGSPSTLLQG

LLSTDHNQQQVQQQSGFDNQPMNYPYPATVSYGMSSGEVLLPNSWSKFPNFLRPNSPPKQHQAPPSQLQFSNSGQFWNASTTDVRSSFFPSLQSQFPPPSFDEKPKQSCTSEVKECSTVG

KKSSGGEASSKRSRSEAPAPLPPFKVRKEKMGDKITALQQLVSPFGKTDTASVLSEAIEY

IKFLHEQVNVLSTPYMKSGASMQLQQNPGKSKEDEGPKQDLRSRGLCLVPVSSTYPVTHE

TTVDFWTPTFGGTYR

>RsbHLH059

MKVEICMGDGGWSEEDKAMGAAVLGTRGFDYLISSSVSAECSLMSVGSDENFQNKLSDLVERPNASNFSWNYAFFWQISRSKAGDLVLGWGDGSCREPREGEESEHTQIAHSRLDEVTQQRMRKRVLQKLHTLFGGSDEDSYAFGLDKVTDTEMFFLASMYFSFPRGEGGPGKCFESGKHVWISNSLKTSSDYCVRSFLAKSAGIQTIVLIPTDVGVVEFGSLRSIPESFELVKSIKAAF

SSFSSLAKAKPTKDVNAHFFKLGNGERPDGIPKIFGQDLYSGRSQLREKLAVRKSGEKPW

EAYSNGNRTPFPNARNGPHGSSWTQFQGVKQVATAGIYSPHTPSDNLPEVINGVRDEFRL

NHYQPQKAAAPMQIDFTGGATSRPPTTSHPVSVESEHSDVEASGKDELAGPVDEKRPRKR

GRKPANGREEPLNHVEAERQRREKLNQRFYALRAVVPNISKMDKASLLGDAIAYITELQK

KLKDMESERENLGANPILENQTRVPDIEIQATGHDEVVVRVSCPLNTHPVARVIQAFKEA

QITVLESKLATGNETIFHTFVIKSQGSEQITKERLVAAFSHESNSMQPL

>RsbHLH054

MCWRMAKGSQNQQGVPENLRRQLAVAVRSVQWSYGIFWAMSTKQEGSGKSKLVLEWGDGYYNGEIRTRKTVPAMELKPDKMGLQRSEQLKELYECLLEGEESEQQANMPSAALSPDDLTDAEWYYLVCMSFVFNPGQGLPGRALANGQAIWLCNAHSADSKVFSRSLLAKTVICFPHLGGVIELGVTELVISVLLLDLPLYVLKMHLMLYELSQKLLFLLYFCCPKVLEDPSLIQHIKTSLLEFSKPVCCEKSSSGPSHRDDDADFMYAEVDHEMVNTISLESFYSPAEDMGFDREGLNDLEKNTMDNLTMGSPDECSNGCEHNHQTEDSFLLENVNDDFSNCVQVSMNSSDCISQVVVNQEKFISPPMGDKNKLKELQEGNDMKFGSLDIGTDDDLHYKRTLSAVLMRNSYRLLDNLCFHSWDYKSSFVSWKKGEIRNGHRPRVPQQMLKKILVDVPLLHGFSLKSQQGNGGKCWLLKQESDRMSVQHALPDEGIENEKFLVLKSMIPSTTKVDKATILGDTIEYLKELEGRVGELESCIDLAEHEARRKYPDEVEQISESNENRRIGNSKKPWINKRKACDIDETDPELSRVVPTDGPLFDMKVKIKEQEVLIELRCPSREYLLLDIIDAMNNLNLDAHTVQSSTIDGILTVTLQSKVVFFPKLLLSRSSSCISTDDQTSAFNCRRIVLRNTRLQIINLCRGICF

>RsbHLH056

MSQCVPSWELDENPSPPRLCSTLRPHSNSNSNSIPPYIIPMLEYEVAELTWENGQLAMHG

LGPPRVPTKYPNWDKPRAGGTLESIVNQATYLPQGKSGSGDEPVPWFNHHRPAASSASAT

MTMDALVPCSTTSRGGGGAGQRTASTWTHVMDGLGKHVVGCSTRVGSCSGAKSCDDEVLLKRARVARKQVVAQEWSYSRDNNSMDGSATFGRESWELTLDTCDRELGTSGGFTTTSMGSLGNTISSALQSTKATTTATTDDHDSVGQSRFQATPLFYTLSKRKAGDGQEKKKGGGKSSISTKRSRAAAIHNQSERKRRDKINQRMKTLQKLVPNSSKTDKASMLDEVIEYLKQLQAQISMMSRMNMSSMMLPMTTLQQQLQLSMMAAAPLGMGMGMGMGCMDINSLGRHNIPGIPPVLHPAGTPFVPITSWDGTAGSDRLPAPAGVIPDPMSAFLACQSQPMSMDAYSRMAALYHQQMHQPPPSGTKN

>RsbHLH057

MYQDSVCFDPNTMQKGLPENGFSQTLPNITTTTTTSFSMEELSYQEESAAAAAMEMELQQQLGLDTENCYANNHHSNLMDTSHLMQEVVGHDSNQVLMPNYDHNSSWDINNTNMNTHDFQEECLMNHVNEMQNSHHLLNLLRLPKVSPSSVLPNSSFSLGFLGDHFPSTDGSGGSASNVAYDPLFHLNLPPQPPYFRDLFQSLPHGYNLPGSRSGSLFGGVEEREGSGGGEVGRNFDNGVLEFQTDWDMNAKVGNGKKNTKHFATERDRREHLNSKFTALKSLIPNPTKVLSLSLSLSLSLSLSLILHPLVFYYPCLKCSPILFFLFGGYTTKPDRATIVGDAIDYIKELLRTVNELKIL

VEKKQCGRERIKRHKTDDGTSTTTAGEVLEDSCTKIKPDPDHSYSNGSSTLRSSWLQRKS

QDTEVDVRIVDDEVTIKLAQRKKINCLLIVSKVLDELQLDIQHVAGGLAGDYYSFLFNSK

IGEGSSVYASAIANKLIDVVNRQYAAIPPTHSY

>RsbHLH058

MAGFAVMGDVGNFRAGNGTNGKASPTKRLNGHINFSSGTSSCSRFMPQISENANESSSPD

NEHLENDNSSNGFYVPSFPNDSWNDSGFTGQKRNRDGEEKFSGFNALESQHRDSRNYATGLTHHLSLPKTSAEMAAVEKFLQFQQDSVPCKIRAKRGFATHPRSIAERMRRTRISERMRK

LQELFPNMDKQTNTADMLDLAVEYIKDLQKQVKTLEDTRSKCTCSSKQNQSSNPSG

>RsbHLH061

MDEIIHPSSSLSLQQRLQFIVQSRNERWVYAIFWQATKDVNGNFLLSWGGGHFQGTKQCS

VPNKLANNGPDQHKFRAKRGIQDIYSDIDHLVDGHVPDPEWFYMMSITNSFVAGDGILGQ

TFSGGAYAWLAGEHELKLYDCERAKEANVQGIQTLVCISTAYGVVELGSTEIIQEELDLL

LLAKSLFGPNNTTSVRKQPIGPGETSTSTCQSYSGRSNFKGLLQSDLEVVTKRGRKNSKA

RGETPPNQVEAEKQRRHKLNSLFYVLRGIVPNVSKMDRASLLAGNTTRDLLFDAVAYIKE

LQENVGELEAKLICAKSQETKISFPDEQDVNQSTSITRVDHRHSRSTLLSGNGNGIMSAE

VDVKVMGSEAVIQVQCMDVNYPVARLMDAIGALECHVHHATISKVKEMVLQHVVVDQVPDGLRSEAALRIAIVRRFLN

>RsbHLH055

MDFVSSAFQCDPIDELLEFSSIPCQQRRIQKDPLQVMSSSCNNDVAEKPDYHACQQSSVV

FDDSDGKPMDCMRKKLIHREAERQRRQEMAALYRSLRSLLPVEYVKGKRSKSDHMLVAVDYIRRLQKRVEEKGQKRDELKSSFEPSIDINAKSRCFPSCLKESVTVESSRAGFQITMSTA

MSGGLSVSKVLDALLREGLNVVSCISINVEERLHHVIDSEVLVVNFIEEQC

>RsbHLH053

MTDYRLSSTMNMWTDDNTSMMEAFMTSDLTSLWPPPPPSSSTSTSIHVPPMQPIAPFNPD

TLQQRLQNLIEGARENWTYAIFWLSTPNDFPGGPGPVVSWGDGYYKGEEDKGKRKTASSSAEEQAHRKKVLRELNSLISGASPSENDAVDEEVTDTEWFFLVSMTQSFGPGFGLPGQAMYNSSPIWVAGAELLAGSHCERARQGQVFGLQTMVCIPSANGVVELGSTEQIFQTSDLMNKVRFLFNFNDTDMGSWTVPADHGETDPSSFYLTDPSPSVVELNITPANTMIPPSNPHLSKQP

TFGSSTLTENPSSNPHQMQSQSFFTRELNFSGNDGISGARKGNSHSCKPESGELLSFGES

KRSITATSNGNGNLFSGQSQFEDDNNRKKKKSPTSRGSNDEGMLSFTSGVILPSSGVVKS

SGGGGDSDHSDLEASVVREADSSRVVDPEKRPRKRGRKPANGREEPLNHVEAERQRREKLNQRFYSLRAVVPNVSKMDKASLLGDAIAYINELKSKLQASESDKEEMKNQMEVIKKESASKGAPPPDHQDLKMSNHHVMKLVDVDIDVKIIGWDAMIRIQSSKKNHPAARVMLALKELDLDVHHASVSVVNDLMIQQATVKMGSRFYRQEQLREALSAKIADAR

>RsbHLH052

MALELEALSTNESLDYIVYDTISADPTFLPENSLKKPRELGCGASNSSSCMMRRCRQVES

PEVSKKRQNATAQGRKKRRRRPKICKNKEEAETQRMTHIVVERNRRRQMNEHLAVLRSLMPESYVQRGDQASIVGGAIDYVKQLEHLLQSLEAQKLLLPQQGGIRPPTTTAAAEFLEAPF

AQFFACPQFTWSQFPNKCLSKSAAAVADIEVTLIETHANLRILCRRNLRQLSKLVAGFQT

MYLTILHLNVTTLEPLVLYSISAKVEEVCQLTSPDEIAGAVHHMLGIIEEEGT

>RsbHLH051

MELEAILSQKEHFGYTSKDLYDLLEGNWGCEFGLPEQEEEEYGSFEFHEGQINNSNHTNW

NSPPPTSSIWPYSYLDQWGANNPNSSSEVPDTTSIYPCEEFQQLDPISTRPRPRRSRSKK

NMEEIESQRMTHIAVERNRRKQMNEYLSALRGLMPDSYVQRGDQASIVGGAINYVKELEHKLHFLGGEKRTSQKFEAGTSSLPFDEFFAFPQYSTSSTTPCESSGDPISANADIEVTVVE

SHANLKIRTRKRPKQLMKMVSGLQSLRLTVLHLNVSTVDQTVLYSLSVKVEDDCSLTSVD

EIAAAVNQMLGRIQPEAALL

>RsbHLH060

MEGVGAFLDEEWESLNRMFSTGSEHENGFSFGTSSTFWPISDDQASINFGPIDESLSQLT

IPNCDAFSSTNDSVFIPTNQNHHSSDPNNFQETNHAPESSYFYATDNAAENKTSLPLFCD

NAIQEILHEKEEERSDDLENKNHNGKEELQLKRKNDSCEAPKKRTRASGDAPNGKKNVTS

KKKQKLSLNGNGEDQENINVATNGSGCCSSEEDSVVSQELNGEETSESKSISALDSDGKT

RAGRGAATDPQSLYARKRRERINERLRILQSLVPNGTKVDISTMLEEAVQYVKFLQLQIK

ILSSDELWMYAPIAYNGMDTGLYQKILPSQ

>RsbHLH062

MDLGTVVFQQDPFSNLNSYGYKEVEEYHCRDTNSSSPEVCAGDGLLAGASVEVPARRKRQRNYKSLKNKEEMENQRMTHIAVERNRRKQMNDYLTGDQASIVGGAINFVKELEQLLQSLESKNQLKQQSESPQHFANFFTFPQYSTRWTHQSNLLLTGHESRMAAEKESTIADIEVSIVE

SHANIKVLSRRQRKQLLNMVSWFYSTGLTILHINVTSTVDQLVLYSFSVKVEDYCQLSTV

NEIASAVHEMMTKIKEEVMPSYN

>RsbHLH065

MFSIPTYCELGSCSSSGNFLQGVVLSSSRTTTTNMGNSSCSTAEKKAEAASKNHSEAERR

RRKRINTHLATLRTLLPKTIKTDKASLLAEVVRRLRELKKTTSEFAANDTNSETSQSNLF

PTECDELNLCHSETEPGTIKATLCCEDRPELISEITAAVKAAEGKVVRAEMATVGGRTKN

ILWVQFVSPTGCGSGGGEGRLRRGLKGVVNRAAALSSTGPGLQALPENKRARLSQY

>RsbHLH063

MQPSGNAMTWTERDEEGQEDAVSWTKNSNNHQADSKDNSSMNPSLSTFKSILETGWYTNPSHFQTLSNPHQDFKDTNITFCSNSTFQPENLMLQPMDSSSSCSPSQAFNFDPLSQPCFSS

LNAISNSPFDSGFDLGGCDPSFLANLSSNSPVFMGLNPQIEMGNNSQLSQLSSNSELTHL

LPMPNNNTVISGGFGPNDFEGFGDALFLNRFSSSKVLRPLEVSPPVGAQPTLFQKRAALR

QSSNELGNLGISELRSDGIWGKRESVVGEFSKKRKRKEEEEFEEGSIDGSGLDLDSDEFV

ENDYKGDENGGDNGGNNSNANSSVTGGDQKGKKKGLPAKNLMAERRRRKKLNDRLYMLRSVVPRISKMDRASILGDAIEYLKELLQRINDLHNELEATPPGSLLQPATSFNPLTPTLPYR

VKDELGPSSLPSPKNQPARVEVRLREGRTVNIHMFCGSRPGLLLSTMRALDNLGLDIQQA

VISCFNGFALDVFKAEVLLILSPQSGVLKVDDLVYCVLSAMQGRPRTPTRANQSSTSGFS

WFPDLALLLHFAKRQGRRNVIVRSGTGFNLVGKQPHSKIDPSRSSDVRKGPLGWMISMAV

QFEMNKQMSEPSTTGIQTEVCDLCINHDMLFSNWEASDFPPSRVRYMKFKEELIAIMALG

EERSIEFSVASNGDVNVEDEAGRLKKEMNITIQEVEESKFYNELADQLRNDMVIQSNMRR

VLGPNSCLQMVIYGLGSMEYSYISQYQLALVMLLKRDFSHWIGGVEVFDPMMSPVDCKVVEMFGCSVLAINEQCKRQVEKPTLFFLPYLDNDLVGNLLEANWCPTRLNKMVVLSNSLEEMAGGCRNFSKKFVSNGKIYYNRQRERLEYVEAIKKHMTEVKIDRGYSRVVDGFSWHFFHLDLDLNLENLLPGDPPMDVRLKSRRLDKHPNDYRHLRNLEEKYVMEPFQNTDPSVYCWTEMDFTYRNTRRMHCFWRPPHAGWVKLNFSGKCTSDGGNAPAGFGGIFRDEHGSCLVMYSGSIWGADTVVANAEALRQGLRCLQYLSSPVRKLIVEGDDVRVIRWMNGGPEPPTRVAEALSEIFELLVGIEPAIRHVYEEANSMAVELAKRGTGLPNLRVWVSPSLEDLFA

>RsbHLH071

MLKDLLPQNDQKRDKASLLLEVIEYVQFLQEKLQLYEGPYQGWTQVPSKLMPWKSNSAPVESCMDPSQLMRNGSGQEDNTVVMPAMLSNARNSAESDLGEADAYDALNHPPMASNQATPSQIPLQSGVLEDMHTQAHQTSVSYSGQLASQSQSQFWQVRQSTTENAVPSYALGEQEELKVGSAEASFSNAYSQGLLNTLTQALQSSGVDLSQASISVQLDVGKRANTGQTSTFGEKDPQNHSAGNQLRAVHGLGSSYDDSGQAHKRLRTEHN

>RsbHLH068

MALTKDRIPNNTQMGLVQAYTYYGNVFGGSSAQVNSDQSYQPAEGGAQSVINFKTTTAGYNNIIHSNGSSFLSFDQNPDHHHHQNSNPNKISDDQQLDDDYSIWEDNLIQDFSTTRVQST

SFYGADQPFGWPNSEENPNCGNNFPEEFGKQEEGFNKRPHKGESNQAIKKQCTNATKKAKQKSTTPSKDPQSVAAKNRRERISERLKVLQDLVPNGSKVDLVTMLEKAISYVKFLQLQVKVLATDEFWPVQGGKAPDISQVKEAIDAILSSQKEKNSSLE

>RsbHLH073

MQGMEDPIFTDHFDIMEYFDEELAAALGDDFQNSLSSESNSCSSTLLNNIPNSSSTTNLL

CASSTLEALESPNSKHHKPNTWPTNSNSNNNNGSTAAASLDQQPSVFSSGCILSFGNSGS

TENPKIPPGALNSEDEEAVAAGMLLSNKSSQGAKKSGSSNPRPASQTYDHIIAERKRREQ

LSQLFVALSAIVPGLKKMDKTSVLGEAVKYLKQLQDRVKTLEEQSTRQTIESVVLVKKSQ

LLVEDEAAVDESFVSGSSEPLPQIEARVCNRSVLLRIHCEKHKGVLAKIFGEMERLNLAI

VNTSVARFGGLALDITIIAEMEAELCVTVKDLVRSLRPALQPFM

>RsbHLH064

MDAGGNGDLGCGNRGGGGLLDCSSLGNFTALPADEVLGMTTGSEHWNRVHSSFNSGWTPLIGSIAHSNMEVGTSPFSSFESGTFPDMDGFLNLQRCRYSSCSGDNPPERSGDKNEIALTT

QCAADDFAKSQGGEGTIKPSLDGKKRRRASDDWSQITQFEDTKTENDQCPGEEDEEKQKQKPEIKPVSKKCKQIIGREVNDSSSSGDAPKEDYVHVRREKIRERMKFLQDLVPGCDKITG

KAVMLDEIINYVLSLQKQVEFLSMKVSTVYPGANVEPEQILQRDIHYSQGGSATILCDPG

TRPYPNVGVWGNDQLQSKSQMDFNPDLAPDNAESNGRLHELL

>RsbHLH070

MADMYNNKSTCASSTSPPETDDLSLFLHQILLRSSDPLAAAASLPGNPHRPVQPSVLSEY

GCHVTDRISTAESSSGLNSSPGAVFSSSSYYFPAGATNASSSVGTVDIDADEYDGESEDI

LTRVNTVKKALTRYDQLKELLSGSLANGAFARPSSFGAPISDEEIAMNEVGKLINREDRV

SMGKRKSDGSGESSSKSMRSERRRTEEIPPSWTGKDIYIDLIEWLELGSLYEREGFEALV

EEVPTKQVPSRSSLKRGRAAEFHNLSEKRRRSRINEKMKALQNLIPNSNKTDKASMLDDA

IEYLKQLQLQVQMLTMRNGLSLYPMCLPGALQPVQLTQMPKGFYEGNGMDVTGISFNQETSTNTFFDIPNQSTNPAQLTAVDLSSTLSSETLFGRESAIEGDLRPLQFHTSSNSKVNELY

RKDMPHDQQLNVDDHSPKNPLGGLSKPISTIDSVLVLTCVDVVKGFETGAKATVSISINT

QASEVKDKVEACISRMQRPENVHLSNLECEPTLAPNLDGLLFGRSAAKDDIKSQRQDF

>RsbHLH072

MELRQPRPFGAEGRKSTHDFLSLYSPVQQDPRPTSQVLKLKQYIVQFNSGFPSVIFVFLH

PHLKSGAYLKTHDFLQPLEGLGKNITKEENSTVEIITTLDKPPPPAPPPITTSGEHLLPG

GIGTYSISHISNYFNQTVLKPEGAAAVFTVAQSSSTDRNEENSNSSSFNTGSGFTLWEES

AVKKGKTGKENVACGDSKRVSKADPRMAEERVNIRRGALGGGIGGGGQWATSGQRPSQSSSNHVATNHRNATTTFSSLSSSQPPSSQKNQTFMDMLKSAKVSQDDDDDDEEEFVLKKEPSPYPKNDLSVKLAGEGGKSNDQKPNTPRSKHSATEQRRRSKINDRHDLKFQMLRDLIPHSDQKRDKASFLLEVIEYIQFLQEKVDKYEVSYQGWNQEPPKLTPWVNPELGFAVRPEIPLAS

KNNQSSVEGFVDQSRATNSGSGPKLTFAAKFDEDNIVVSPNNPQNGHNGIESDLSTVTTL

KEIDLRTVLTNKAVPLSLPLQPNMLTPTGSSSLMVPIPAGVTSGMDNVMSQPQSPFLQSR

SHATDNTVARNKLKEQELTIESGKICISSVYSQGLLNTLTQALQSSGIDLSQASISVQID

LGKRANGRLPASTSTAKDVVARSRAASSEEESDQALKRLKKS

>RsbHLH075

MDPPIIHEASFSVANPSSYSLAEIWPFPINNGSGGLGLRMNNLSGFGEATLNRDVSVDES

TVTEQSGGGGRRKQQRREANSEDESSKLVSTSSGNDMVEFTLSGDENGVSKSEDEENSGL

GNNPAEQRTKSSEPPKQDYIHVRARRGQATDSHSLAERARREKISERMKILQDLVPGCNK

VIGKALVLDEIINYIQSLQRQVEFLSMKLEVVNSRVSSPIEGFPPKELAPTFDATGMIFG

TQGTREYVQVSQPEWLHMQIGNCFDQAP

>RsbHLH066

MSAFSHQHQPFLLDSSFFTPIKIMSGLLEEPNTPLFSQFFYPPEPVHQIPVHHFSTRCLE

NSTKVDAGINDDDSSSVVDGKAESGEQVTQKLIPMAMDKKRKNRDGSAQSKDAREVKGKKQKRCNGGMEGDEEKKTNKAGNKKKLNEEAPTGFIHVRARRGQATDSHSLAERVRREKISERMKLLQALVPGCDKVTGKALMLDEIINYVQSLQNQVEFLSMKLASVNPMFYDFGMDLDAFMVTPEIRLNGLVSPLMPKSNHHQAIAFSDTTTSAAAALTGPNNYSLVDTPTSSSLLFQQVQRPNILSQKYSSGRKQTQVSKFDITFRIIMNRSCGMWRTKDKELLTNLASATTCVPSINKIPSCSCLPYKQQHARKFYSSKKQREDT

>RsbHLH067

MLPRVDGMMWMQDTGEDQEPPSWPQNNTNINDEMGSLSTYKSMLGVQDEEEDQEWYIHSSNNNSNINFPSNFATEAENNHLLLHSVGSSSSCSPSSASVFQDHHYFLNPPPKPTIPSLLN

NPLDNSFDMGFIDSQLNNRVLTGFNDLTSQTQMGISNLASDPQFSTTHLLQLAENKSMAG

FSSLGFQGFHENSLFLNRSKVLKPLDNFASIGEQPTLFQKRVRKNLETNGSNLGVLGSEG

GGELLRNGEGYNGNMEVSEKKRKLISMEDVDEFSIDGSGLNYDSDEFLESCKGEESGRNG

GNSSNGNSTVTGGDQKGKKKGLPAKNLMAERRRRKKLNDRLYMLRSVVPKISKMDRASILGDAIEYLKELLQKINDLHNELESNPPGSSLTPTTTSFYPLTPTPPSLPCRIKDELCPSSL

PSPNGQPARVEVRLREGRAVNIHMFCSRRPGLLLSTMRALDNLGLDIQQAVISCFNGFAL

DIFRAEVPVIPVILPQHDMEDLRVLRGMEISISINAEKAKMSIQTKSKQYCWIQLASWHD

VGFLSSHIKFAAWLVDRIVFPTK

>RsbHLH069

MQRETLASSSTSSSSYNNPPPQDAWVGTYLRLLPHWQSLTPSNQPIIPISISRVNQVDAH

RLDIEMSAMLKEQLVNVFSLMKPGFLFQYEPELDAFLEFLIWRFSIWVDKPTPGNALMNL

RYRDERAIGLGGKAVKLDCMKRGKGDQIAGFKLCSWRVRTGLEGPGLTVGQKLWYCVATVGGQYIWARLQSFSAFRRWGNSEQRSVARRAWTLIQRIEGIYKAASFVNLLIFLYTGRYRN

LIERALKARLVYGSPHMNRSVSFEYMNRQLVWNEFSEMLLLLLPLLNSSSVKNFLHPFSK

DKSSSSGGDETQCPICLVTPTVPFLALPCQHSYCYYCLRTRCSAAQSFSKDTSVAWGGLA

SEEDAHDNKFGGGSHFQLKRLENWQDQQQQQQQILINPSLLRVPVFDVKQEDAHSSQVYGTHRNVADEFQTSSRPNSWSQVVMGVSSSPSTAAGGASKKARVHSTPTLPPLKVRKEKLGDRITALHQLVSPFGKTDTASVLFEAIGYIEFLQGQIEAREFDEPRDLRSRGLCLVPLSCTQ

HVGSENGADYWAPSLGGGF

>RsbHLH074

MDSLGWDSSTVLANTPPLWSHQQHELEEIFMSSSSNYCTHGGGKFDFSTEEIFTPIHHHL

QKPHVNSNSVIVSQMEQQITRVEDGENPIILSECKNLWPDFCGSTRALSSGESGSNENDK

GNYKDTLIGEDITTLDKPNPNKRRNENSSQFDLFQSDSSTTGGVFQLISETNPPKPKKPR

SEKFPNSSNISFQQPNSSAGEPDSEAIAQMKEMIYRAAVFRPVNLGVEIVEKPKRKNVKV

SSDPQTVAARQRRERISERIRVLQRLVPGGSKMDTASMLDEAANYLKFLRSQVKALETLG

QKLDLVNCSTPNTLLPLSSLVPFNHSFPMQINSFSTAPNYS

>RsbHLH082

MLEHKRSPVSVDQGSLDSFAPKRRKADMSMTSKVSFSLLYFCITLLCFVLLVTLDGDQER

KDKLGEKIAALQQLVSPYGKTDTASVLLETMEYITFLHEQVKVLCSPYLQCTPAANLQEL

ERYSLRSKGLCLVPNSSTGGVASSNGADIWAPIKTSTPKF

>RsbHLH081

MYPSSSSPPSHPSTNNPTGLTRYGSAPCSFLTTAVDSLTTTAVAATRDFSTLGPHHQPTH

HNNNNNNHPNNIHSSINNNTTPSNASQGGSRPYCLNAIATTVGGEFTAAGGGRRINLNSK

SGGGGGGGLVRHSSSPAGFLDHLNATNNSATGVSNGNGFPLTRSIGSYNSKGVSDTGHGI

SRLSSQLSFTSDDALSCISGERENGRRKTAHSYATTSFGIGSWDDSNDGFFSASPGKRAR

GDHCANTAESQFQFSLSQTDLEMSTTENRLHIPYDSVPCKIRAKRGFATHPRSIAERDRR

TRINGKLKKLQDYVPNLDKQQTSYSDMLDLAVQHIKGLQNQVQKLKEEVENCTCGCKQTK

>RsbHLH085

LQGNFQGADYSIDHHHQLMKPRINEETYVDASNQIADYTSLTSHPRSSTSPDKLSFADVM

QFADFGPRLGLINQTKIPEEGQEEEVETGIDDPVYFLRFPVLNEDRYKGLGEERGGGEER

GDGEGRGNSENNNNATSEGLGIVGGNFEKNLAADDHGVMSNKNKRKRARSVKTSEEVESQRMTHIAVERNRRKQMNEHLRVLRSLMPGSYVQRGDQASIIGGAIEFVRELEQLLQCLESQKRRRLYGDPPPPRPMGDSSTSSLANIPQPSQTPPPFFPPPVVPSLLPNSHDNQLKLVEFD

PTAGLREETAESKSVLADVEVKMLGFDAMIKILSRRRPGQLIKTIAALEDLQLIILHTNI

TTIEQTVLYSFNVKVTSEARFSADDIASSVQQIFSFIHANNTM

>RsbHLH078

MAGECTESSIATSSATPNWWPDLRTNSLSSWSSNTNPWHPHQNPNSNSSGEEDMSISNQS

GLSVDSSRHLVDSSANDFIGETASDSQLWSHILLNVGSREELNNTQEVGGNSTNLFASKS

LSTGMFEPACSDYLKKMDNSWEFTNSPSFNNFDQKHFNGFNHGVMETKRLTKLSSLVSNWSIAPPDSAGILNHQFDLSHMKQTMSNPTPFEGILNRNLGSFSCYGHDSKVKDEHQIGLSN

SFVGDNKYYYGTSEVPCNTSTRSFSDVMNFSSFLSKPSVGIHGSKSIPRSLPDCKKQTDT

ASVLGEAIGYIKFLQEQVQLLSNPYMKTNTCKESKKLLCPIQDPWGGLERKDRGDLVLFD

LQSRGLCLVPISCTPHACRENTGSDYWTPTYRGCLYR

>RsbHLH079

MADFNADFQNPKPSNDISLMNSNMELLNNLGPFENLHSNVQGFAGLLDPMTSNFMSDNIP

NSFHSDGAFYEAVDNFMPSTEGPSRQGKRAKELSQTDALGISSRQVCGTSDQSKKTYSYG

GKKRKRSNEREVKTPGEVVHVRAKRGQATDSHSLAERLRREKINEKLRSLQDLVPGCYKTMGMAVMLDVITNYVRSLQNQIEFLSMKLSAASLFYDFNSEMDAMETMQGTNGYEAQVMERVVGDHHHQGYGGGGAGEEGGAFSNYFPSSAWPSLT

>RsbHLH083

MICGKKELEEAESFQEQLLLEQFPWSLPPIHYSFNPTHFDPNPVHDHNPFLLPPSVPSPY

GGLLFSRGSSEHDHLRLVSEAVGHAAAQYSSSSAPFGLQAELDKMTAQEMMDAKALAASKSHSEAERRRRERINNHLARLRSLLPNTTKTDKASLLAEVIQHVKELKLQTSQIAETSPVP

AEVDELTVDASDENGSLVIKASLCCEDRSDLLPDLIKTLKALRLRTLKAEITTLGGRMRN

VLFVTAEEQSNVDEFGDQHRQDYSVSSIQEAFRAVMGRRNGDECSTSSGGVTKRHRTNVNMVENRSL

>RsbHLH076

MDVEGKDEFEQEKGDEDLMSYHSPNVSSDWRFGGPNLTNPLMGSIPAAKPMTVCSKGGLMESSSSSSAPLMDSFCHTVWDHPTSTSQNLGFCDINLQNSATTSNNTLGIRKGPLAPLSVD

RTVDIGWAPPNPMVKGGGVFLPNAHGMLLPQSLSQFPADSGFIERAARFSCFGRGNFSNM

VNSFSVSESMNPFARSGPVMMQQAQEVFGGNGLKSDVKMNEASKGVSLSAEQGATTEGSPLKNERRSNSFGMSANGSDEAENSGGDGQDNPSSSQPIGLNKRKRGGGGQDNELCQNEALQPTGEATKDNTEIDPKGDLTPTSTSNKPSGKHGKQGPQPSDSQKEEYIHIRARRGQATNSHSLAERVRREKISERMKFLQDLVPGCSKVTGKAVMLDEIINYVQSLQRQVEIEILELLDFM

FLSMKLATVNPRLDFNLEGLLTKEILQSRAGPSSTLGYSHDMTHMTMPFAPLHPSQPGVP

VMANSSDALRRSINTQLAAMSEGYKEPSCQVPNMWGEDELHNVVQMGFNSSAPLDSQELNGMTDYIPVLWVIAGAWVFNCDTANLYLMLCQCPSFENPVVNLIYCSARKAKFATTAMELFCRQEKIWIKVVLLDKTKMHACCKGF

>RsbHLH077

MKSGQQEEAEEEDEVIVSKREGLSSNGEDGKNRNKANATRSKHSVTEQRRRCKINERFQI

LRDLIPLSEQKRDTASFLLEVIEYVQYLQEKVQKYEGSYQGWSMEPTKMMPWTAWATYEVSATVFCLKQRNSHWRVQSLVGPTQAVKNDSGPASTFPGRFDENNITSSVAMHPISWNSTE

SDSSRDPSGQIDDQQSNLANKAIALPIPLHTNVSSSGPGDDVVPHPIHRPISDAQSTECP

ITSGALTEQEDLIVEGGSISLPSFYSQGLLNNLSHALQCAGVDLSQSTISVQIDLGKRAN

RRLTSGAVVSNTKDSESASPSHQPIGHFRDSTNGEELDQAQKRLKI

>RsbHLH080

MFPIKESDKELAVFEDLIMDDDDVSQLERSKTYSTNYRVGKRQQKLAAIPEENDEVVVAS

DNKQRAVHKEIERKRRQEMGYLNASLRSLLPLEYVKGRRSISDHIHESVNYIKHQEKKIK

ELRIKRESLQMLSAASSSSSNMLPISITVSPCRGGVEILINSGLREEGFPFSKVLEILLE

EGLDVVSYVSAQVNDRFLHTIKSEVSDMTCVDLSAVQQKLNDMINLL

>RsbHLH084

MGVSDSLSDHFFEESELEDIFSILENLENVEEFPQSDPENGLGSNEGETGGGLVSQKSTC

SSAGNLQPESEAEVELAANNYLPKSNKRQKLSTAATSTDEEIPQGEGQLNRISHITVERN

RRKQMNEHLTVLRSLMPCFYVKRGDQASIIGGVVDYINELQQVLQSLEAKKQRKVYSEVLSPRLIPSPRPSPLSPRKPPLSPRLPPNLPISPRTPQPCSPYMPRLMQHLSPLPSPCSSSS

SAASSVVVDNATTNELVANSKSAIADVEVKFSGPNLLLKTISPRIPGQATKIMSTIEELS

LEILHVSISTIDETMLNSFTIKIGIECQLSAEELAHQIQQTFC

>RsbHLH087

MEFTQQGLLDELLAAPRRDTWTNFPSNVDFSNGWIFESLEHESQVLSPPNAQFLGLTSPT

EPSFSFPLHGLHYPYADTFTVPEIDSSYDSNAPPLIPFPTQEQHPSMVKNDGEYGFVGSD

LHTLEDRLNGSKVEMEPTANTPEFNMDFGGQRKNRAKKVEGQPSKNLMAERRRRKRLNDRLLMLRSIVPNISKTDRTSILRDTIDYMKELLEKVKNLGGEQDAKGSMNQVQLMGKSKELKPNELLVRNSPKCKLLVQSQGMQQQAITSSEGIKQALFRNAGYGGRYL

>RsbHLH086

MEEPMFVDPCDFMASMDEEFFNVPNNGSATNLSSASISSNTDLAPQIHELQPSKQYHMAK

TFTSSSSGFILSFGNSSNNPTENPQQAVPMNLEDEVISSFLISQQRSLFENQEEGITKSG

RVGMKRKSSGSSSSNPRPRSHTYDHIIAERKRREQLTQLFVALSGMVPGLKKMDKTSVLG

EAVKYLKQLQERVKKLEEQSPKEKTMESVVLVKKWQLSFGDDESCSDEKFTGGSNIKPLP

EIEARVCNKDVLLRIHCEKHKGVLAKLLLELEKLDLVVVNTSVVPFGGLSLDITIIAEME

TEFQMTVKDLVKRLRPALQQYLM

>RsbHLH090

MEVELMKPSTLDQTEMMKMMMQLDMLPQLSLPQTNTFELPEIKFYGENAAAANRTTEKTPIFHNPFLTSTTSTNPPSSMSFLSTPFQEPMTPPLSSTSFRTDSTIRSLSPPTITNQKYFE

TVSQTMGCNFRKAQKNMLIWPEASEDLVPLLIDPLHERNSIGSVREMMFRMAVMQPIQMDPESVKPVKRRNVKISRDPQSVAARHRREKISERIRILQRLVPGGTKMDTACMLDEAIHYV

KFLKKKCSRGNK

>RsbHLH088

MALETLDFQQDPFNYDHKDLYTKGASYGYWDSLLSNADSFLPDVDLTGNDGVASGICPSPVGAPVAAQGKRKRRRTKSLKNKEEMESQRMTHIAVERNRRKLMNDYLAVLRSLMPPSYAQRVLSLSLSLSLSLSTHIIFQHKNGDQASIVGGAINFVKELEQLVQFLESHKQAKQKPHDS

PLFANFFSFPQYSTRLTGHNGSVTGQDQYSTAEKGSTIADVEVALIESHANVKILSRKQP

KQLLKMVAGFHSLGLPILHLNVTTVDQMVLHSLSVKVGDECQLNTVNEIATAIHEMMGKI

QKEEAISS

>RsbHLH089

MDKRQRPEVEVKDPNAARKVQKADREKLRRDRLNEHFIELGNALDPDRPKNDKATIINDTIQVLKDLTTEVNRLKAECAALSEESRELTQEKNELREEKATLKSDVDNLNLQYQQRMRVMFPWGTIDPSAVMAPPYSYPVPIPVPTGPIPMHPVLQPFPFFQNQNPGGIPTPCSTLIPYP

GPPNHPMDQPSCQCASTSRDSSKQDSKSKTSDHCTGSIDDKSDDSNDVVTELELKMPGSK

AQQEHSPGGTAGKQATRKEKSIADGNSSSSRYSSSPGFQDSISDGLEHHED

>RsbHLH093

MALETLSSNEILNFLMYDNVFASKFDYNDPSVPTMLPDTSLKPPQFGGASVISSMMQRCP

PVEPESVGKRRNPAMLGRKKRRRRQNVCKNKEESETQRMTHIAVERNRRKLMNEHLAVLRSLMPESYLQRGDQASIVGGAIEYVKELEHLLQSLEAQKLLLLKGGTPPHDNATAPSTAEF

FSPPFSQFIMFPQYTLSQVPNKNSSKSKTEVADIEVTLIETHANLRILSRKSLRQVSKIV

AGFRTLYLTILHLNVTTLDPLVLYTISAKVEERCQLKSANDIAGAVHHMLGIIEGNLPSG

DQPFKLPQIPL

>RsbHLH091

MPLSEFYKMAGGSLESAQQRTTTTSSADLSHLPGNELVELVWDNGQIVMQGQSTRARKTSSSFDFQSQTPKVRDKDRLNATHSKIGKFGVMDCVLNDFASAVPSGEMGLDQDDDMGPWLNYPLDESIPHDYCSDILPELSGVTANQISSHSSFATNKRGSYNRTTRGSNYVQNGLGLEHE

NAAKVSSSEVGEGSGSRISHFFPWSFQENQTSLPSLKSGVSSIISNNITNTKHAVCGDPS

SGVKIQKQDSVLPNTNSGIMNFSHFSRPAALVHANLQNVSAIASPITLGIEGMGSKNKGP

SLSSTNFAESTLINLSSGLSKGMDSNSQPNSVPPKVDSVVMDKPIEELRAVEQSEAVCRE

DVVKNDKLLSQVVGEKTVEPVVASSSLCSGNSADRASNDLTPNLKRKCRETDDSEGRSED

IEEESVGIRKAAPVRRGTGSKRSRAAEVHNLSERRRRDRINEKMRALQELIPNCNKVDKA

SMLDEAIEYLKTLQLQVQMMSMGAGMYMPPMMFPAGMAQLHAAHMARFSPMGMGFGMGMMDMNGVSPGCPMIQVPPLHGAHFPAPCPAPIFAPTSFQGMAGPNFQVFGHPAPRVPFSGVAPINSAIELNATGMATPVELPNSAPSSNPKDLIQNANSQMMHNASTSMNHTSTQCQVSNAGFGQSSLVYRSDQAPDDVCRDDMNLTKEADVLLRPGCK

>RsbHLH095

MEDVGFMRQWPTNSLDEIVNHHFSHQIFDVKPNITEYSSHDTGIINPPGKILKTNTWNSS

SNSSSPHQISFSNSNCETQFGTIKPKEEAALSPSSNITTLPSGVYDFQQSFGNQIYGFKA

RQGSKKNSTNGTRFSSSQEHIMAERKRREKLSQRLIALSAIVPGLKKMDKASVLGDAIKY

VKQLQEQVKTLEEQTTRTKSMETVVYVRKCELNSDHNLDNFSSENNFSGGGGSGGGGSQYDETLPEIEARFCDKNVLIRIHCEKRKGVVEKTVAEVEKLHLSIVNSSAITFGSSVLDITI

NAQMDEDFSLTMKDLVRNLHAALKLFM

>RsbHLH092

MSEVGEHENFVWESQSWAFANSDNSGSGGKPPDLGSNTQTPTGKEMEAVQAATGGKKRSGGGKKKGKRDDGGSGGDDGEGNEGKSGGESDHEIHIWTERERRKKMRNMFSNLHALLPQLPPKADKSTIVDEAVNYIRTLQQTLQKLQRKKLERLHGVTPINYDSSVVTPQKLAIDSREAFIADQVSSSNLANTSTPANSNSSNSVLPFVSRFPPIFQTWTSQNVILNVCGEQAQINICAP

KKPGLFTAICYVLEKHKIDVVSAHVSSDQNWTMLMIQAHVSPKNSRFPFIKNFNLSLLHL

YRWMNMIFFSIKEKGIRVLEFSPQPRLGKSENINSHLNVGESETESMGYRACNKARIAHD

QLPEAFPVEEIYKQAAGEIMLWLSS

>RsbHLH094

MNRALPEMLHCINTPGNVAGSFTDMSVLERQRARIKWQQEQLLNQPPCYFDGNDEFNGYSMPNHAQDFHNLINSEVGLVVKADPGLDNGWQEFGQFGGEGSGLGSSGFVNGSGFELNYGISRTLSCPQEVAAEMEVAAVTETARRESVSPEKMSSAVGRESFKKRKADRNQSLKAVAEEEPNEKRIKECNEEDESKITEEYSNNSKNTTNNDNNIKRETSADTSKDNSKVSEVQKPDYIH

VRARRGQATDSHSLAERVRREKISERMKYLQDLVPGCNKITGKAGMLDEIINYVQSLQRQ

VEFLSMKLAAVNPSLDFNIDSFFAKEVFPASTSNFPIGVQSEITNPAYLQFNPLQQLASC

TGLEMGINTPDMALRRTTSTPLSLPETFLNSSCFDVISSTSIVFRISVSMLGTFFLTKVF

SHYSKSKPQQLGMEICKTFTIIVEEEGSNDASNLKMEM

>RsbHLH103

MQHITTSSGGGGGGNRGGEAHRGLARFRSAPASWLEALLESEEDEDDPLLNPTTQFQTQL

KPPTAQFPPTSYADPASFDAGGGPAFLRHNSTPADFLAQISAGPDGYFSSFGIPAGYDYT

ASSVNVSPSSKRHKEADTSAKFLPQLKAEPSGMSGLLDVEMEKLLEDSVPCRVRAKRGCATHPRSIAERVRRTRISDRIRKLQELVPNMDKQTNTADMLEEAVEYVKLLQKKIQDLKELQ

KNCTCADTE

>RsbHLH100

MKSAKVHLEEEEEEGETPPSNSKDGKSNDKASATRSKHSVTEQRRRSKINERFQLLRELI

PHSDQKRDTASFLLEVIDYVHYMQEKVQKYEGSYQGWVSEPSKLMPWSLSLLLLEQRNSHWRVQSFVGHPQPITNGSSPASAFTGRLDENNSTISPTIHPNQQHSIESDSSGDPSGKMVD

LQPELTNKSMAMPMPLQATIPSSIQCDGVFSHSLQRPISEAQSTEFPSRNDGSNHEEDLM

VEGGTINIRSAYSQGLLNNLTQALQSAGVDLSQATISVQIDLGKRANTGPTPGISISKDP

QNSPSHPSMGRFDDAGNGEDLGQAQKRLKK

>RsbHLH097

MMCGKQEEEQGDYSQNIPNQQNYQERFLVQQQHMQQQQQQQQQQNTASDAYIGDLYPFLPWTLPPVHSFNPVREPDPFLLPPQPYGGLLFNNRRSSQPPLQFSYDGLISESLGHVVQHPG

SAPFGLQAELGKMTAQEIMDAKALAASKSHSEAERRRRERINNHLAKLRSLLPSTTKTDK

ASLLAEVIQHVKELKRQTSLIAETSPVPTECDELTVDTSDEDGRFVIKASLCCEDRSDLL

PDLIKTLKALRLRTLKAEITTLGGRVRNVLFITGEDEEESNSGDNHQQQQYSISEIQEAL

KAVMEKTNGDDSPSGSVKRQRTNVHIIEHRSL

>RsbHLH101

MGVIHLPSRRDSQGWSYARIAKVSREWYRARVGSQKVGGQIANGSSTEDRKGPAGVVAREDHEAPEYFSLENLPESSGDESESDRTEFGVVPETRYIEWVGCCCGGAYGDSNGIKIEEER

PEGKPQLLIPQCRDTHIQHPANTKACLALSHFPPSIPLYSGVHGEAVLSTEPKWLSCTKD

SDSNQSHKSNGTQVLIPVVGGLIELFSTKHLPRDQKIIAFIVSQYNNTMEQDSITAKSCS

NMRFNVQSHDTLPGAHYPVNNLPALFEAKKFFPRLPFLPPVSQLNLHPTLEGSSTGSNPS

NEHSISFNSGSVHVSPNVSVNGTFGEDPTNDKSNKCRNLSPKQDHLVETDKLKSKQRMEK

EQYHSKNLVTERNRRRRIKDGLFALRALVPKISKMDRAAIVGDAIEYIEELHKNVKELKA

ELAEMEEVERKKKNDELVIPKLNTKKGTKTSTTTADEKEKMEVQVEVHQIGTRDFFVKLL

CTQKTGGFARLMEAMHSIGLQVVDANVTTHNGKVLNILKVEMAHRETPVPVFQGQESDQFFAFLSQHGMTRTEEGNREHDIIKTCLLRGMGFFGNDTNVVAIHQNTNSSPTAQARLERFRIFTRAVAEKCGGNANIKHAWYGASKGEICEIMSHGFISRSRQPEEDGYGLGVYLSPLDFA

INGALSSEADENGLRHLLLCRVILGKMEEICPGSRQFQPSSEHFDSGVDNLSAPRRHIIW

SAYMNSHIMPNYVVTFRAPNLRGLETIQTNVPEPTSPCIDIPTLMSKLSNFLPRSSKELI

EKYHTEFQMAHHETPVPVSHGQESDQFFAVLSQHGMTRTEEGNIVHEIIKTCLLKGMGIF

GNDTNVVGIHRNTNSGPNRAGSVGKVSHIYSGGGGKCDGNANIKHAWYGASKEADNLKRMAYGLGVYLSPLDFAINGFPVRLESLDPYIEARVDKTQGHCYWIHVCTY

>RsbHLH096

MDPNEGAFQTARYNFAEIWPFPVNGGEAGGGLDLRRLQFGQSLGLFAEDVNVNREVENDPTVPDHGRARNGGSKKRRDQNPEEESAKAVSTGGNAMDDCDGKRTKMGGSSDENLDSKAEAEANSSNPTEQNTKPPEPPKQDYIHVRARRGQATDSHSLAERARREKISERMKILQDLVPGCNKVIGKALVLDEIINYIQSLQRQVEFLSMKLEAVNSRMEPGPERFPTKDFGQQTFDTAGAAFGSQATREYERSSSPEWLHMQIGGSFERTT

>RsbHLH098

MKNGKSNSTPTSSNSPEKLDRKTVERNRRMHMKDLCFKLTSLVPPRHFQPSKDMLSQQEQLDQVATYVKQLKERVDELKARKAIAMGNNNNITGTKNSNKETTSSSSMSEYFRSPVVELRDLGSSLEVNLVTGLEKNFKLSEVIGILHDEGTEVVSAVFSTFGDRVFHTLHAQVRVSRVG

VETSRVCQRLQELVCS

>RsbHLH099

MAAFSNPNHPFLIHPIFLPNNSAFSQEPNAITPTPCFPQFYPPESHNTEPYVTNNINSAD

SSSVVDHKAESGEQVTQKAVSMAKKRSHSDGSSLSSVQSMGAREVKGKKQKKSDGTRKDENEKKLNKAGKKDQKIKAAEEAPADYVHVRARRGQATDSHSLAERVRREKISERMKMLQALVPGCDKMTGKALILDEIINYVQSLQNQVEFLSMKLGSANSMFSIDSGVEFDAFLLTPERLSSMESPLPNGQPCNNSSQLPIGFPHSTTFPPQDTYPLLDNISSLLFDQQLQFTHTLHPQY

FQDNGQLVWDVDEQRQKLVNQSGLISHNNFNLCSFH

>RsbHLH102

MMPDSNCYVQGGNNKMCFPEEIFPSIHDHLREVQPSSLANSNSEKESSKMVPQKLTPVIL

DSTTGAPMVFSECQDLWSTFGSISAVSSSDSVISKELDNKPKPMKRRFDQIQPKFGANSS

LDSSTSEGGFQLISTENPPKRTKKTHPSLSTISFHHPSSSASSVQQPDSEAIATMKEMIY

RAAAFRPVNMGVEEVAEKPKRKNVKISSDPQTIAARQRRERISERIRVLQRLVPGGSKMD

TASMLDEAANYLKFLRSQVEALETLGGHHKLGLVNFSTPNLPLIPMQTLFPLQTTPRAIR

HPNI

>RsbHLH111

MAAPPSTRLQSMLQTAVQSVQWTYSLFWQLCPQQGILVWGDGYYNGAIKTRKTVQPMEVSAEEASLQRSQQLRELYDSLSAGESNQQTRRPCASLSPEDLTESEWFYLMCVSFSFPPGVGLPGKAYAKRQHVWLTGANEVDSKVFSRAILAKVYATDFSARIQVQEDVGLVHQVKSFFIDHHPPQPPKPALSEHSTSNPATSTDHPRFHSPPIPTTVYAPVNPPVNPNQIDEEEEEEEEE

DEEEEEEDEEGESESEADTGRNQNPGVVNVAHAAAAEPSELMQLEMSEDIRLGSPDDGSN

NLDSAEFQLLAASQGGNTVDHQRRADSYRAESTRRWPLLQDPMSSSLQPPPSGPALEELT

QEDTHYSQTVSTILQQQSSRWSDSSSSSSAAAAGYLMYSSQSSFSKWNPRPSDHHYDAAI

PADGTSQWLLKYILFSVPFLHTKHHNDNNSPKSASAADSASRFRKGTTPQDELSANHVLA

ERRRREKLNERFIILRSLVPFVTKMDKASILGDTIEYVKQLRKKIQDLESKSRQIELDQR

DQNQRSRSSGDLQRSSSLSLKEQRSGITTVVNTDRARVGGGPTGSDKRKLRIVEGTGGAK

VKAVDSSPVPVLSPPPPPPPPPPPQPVAGVGVQVQVSIIESDALVELQCPHREGILLDVM

VVLRDHRVEVTAVQSSLTNGIFVAELRAKVKDNGSGKKPSIVEVKRAIHQIIPPY

>RsbHLH106

MNYCVPDFDMDEEYSIPISTSTRPKKLPVVDQDEIMELLWENGQIVMQSQSQRSFPKRPL

IENGAVTSLDRREIRSSHPIESGLNNDQLFMQEDEMAFWLHHYPPIEDADLYSDVIDPAP

PRTAPNQDVRIPPPSTAAPMSQPRRSDAEESGAVQSFVHCSRAKARAVEQGPSGSNKAVR

ESTVVDSCETPRAAPESVASRVAGSTVEVSGGVASLCVAAEGGGRDTGTYELTSSSGDSA

SLEPVQKPPVTAGDQKRKGREADDDIESPGEDVEFESPDAKKQIRGSTPAKKSRAAEVHN

LSERRRRDRINEKMRALQELIPRCNKSDKASMLDEAIEYLKSLQLQVQMMSMGCSMVPMMFPGFQQYVPPMGMAMGMGMGMEMGMSRPMLPFPSVLAGSALPTPAAAALMGPKFPVPAFHMPSVPLPSPSNQSDPVPKSLSPQNRNQPQMMNFPGPYHQYLGLHHTQLPLPQNQAMMQPSTTKPSTSREVENHKNLQLVEFRDKRGDLDSEINNLQPCGQKFTNDG

>RsbHLH105

MDNRYFLSSGIIPPPFYSEQSPMPMWQSVSNLNSPPRLRLPIVEHLVKENMPNLGFPMAL

NPNLPTISGDSGFTERAARFSCFGSRSFNERTSQLLGSTESPYRSSSALMEREKMPRVSS

SGSFKGAGSPMSARENRNSTQSHTGMMCASDFDMNLSQVPMSDRAEFPSSNEGSSVSGQIPNGETGSKGPCDSSSKKRKPACRGKSKVVEAEDDGITKRCKSTEGNGIENDNVKTENKTDEEKGSPKPAEAPKDYIHVRARRGQATDSHSLAERVRREKIGERMKLLQDLVPGCNKVTGKALMLDEIINYVQSLQRQVEFLSMKLASVNPRLDSTNMDGIFPKDVSQPNGSFPHPVAVTQSSVHPLDTTYCQNIGTLVDELGQGLPQFPTLCEDDLQSIVQMGLGRSPNRDMGFYSQFFP

GSSQKSHMKSEL

>RsbHLH110

MERDTSGTQLGHIMGDFNPLLGLMDETNFLQFIDIIRGESSDPIAKFCPNFDCEHIGGCL

VDNQFESSTRLNPFDHFHTDQSVSNPDSDLIINTVLQLATDADEEENDNHEESSTTTTTT

PPTKRKGGDRSRTLVSERRRRGRMKEKLYALRALVPNITKMDKASIVGDALLYLQDLQTQ

AEKLRSEIAGLESSLGGREKNQRGIVGNQKNTQFTNTNHPICKNIFQLDMFQVEERGFYA

RVECNRGRGVAPSLYRAIESLTRLNVLSSNLGTRAERFILTFNLKVEEWEPDTNLQKLKL

GLTEALLKQGFVFQTQASA

>RsbHLH108

MALSVYNSNWAPLKHLNFPPPEITNPAFPVPPQALPELDMELLAFQDHLFYPDYPCSTSI

DPLFHHPANSDLNILPCLALPPPPPPLDNSIPPQPFQYSYQYPKRQKCYEDEYYDYCYYP

EEELMPSLKPDGFIANPPLLTPEFSLPEIYGPIVPAFDYGGCGDQEASGGKRASGGGGGG

SLSAQSIAARQRRRKITEKTQELGKLIPGGHKMNTAEMFHAASKYIKCLQAQVGILEFMG

SVQENQEESLLHTRELQALASSPSIQEKLYSLDKCLVPKQFIGTLANYPDLQSNALISTE

LHQLMRTSSG

>RsbHLH109

MAEDFQAGVCNSTWWNLPKSPLGYWPSSHDQLVEMSNARSSDDSGGSVVSDQQPDSGGGVWMDSSTLEMMGINGISSPTSPTTTDWNHTLFGRRESDYNSRMIQEDMNPRLDFGQETGSNCPSKIQKEWSTGNTTTNGFNQTNQDFSVLDHRQSSHGLIPSILLQTLFDTTNPQPQQSLFDNHRSTKYSDEFPPSEKFSPLVLKGSFTKQPAVSNPLNLTTTAPFWNTSNASVKDTRAAGFLPSSVQTQFLASTFEEKSIRPNLIAKANNIEVVQDSCNKKSSGGPVFKRPRIETPSPLP

TFKVRKEKLGDRITALQQLVSPFGKTDTASVLQEAIEYVKFLHDQVNVLSTPYMQNGVPV

HHQPQMFDKLKNTEGTKQDLRSRGLCLVPISSTFPVANETPVDFWTPTFGGTYR

>RsbHLH107

MTSYSWNSNVSNFGSNCLEMLEPFSGNLGGNWGGGSDSVSVAQSLVLDGERGELVKAPIGVGKKSVGVPEEKVVAALKSHSEAERRRRERINAHLNTLRGLVPCTEKMDKAALLAEVITQVKQLQKTSRESATGLIIPMDNDEVRVEPHHDEDGSFSLRVSLCCDYRPELMSELRQALDS

LHLNTVKAEISTLGDRIKNVFVFTSRKDIDSENAESCQLLVNSVHQALSGILDKASASAE

YSPRTTLPTKRRRISYIDSSSSSS

>RsbHLH104

MDTNRQIPQYSHDTNQQQRQVPSGSGLMRYRSAPSSYFAELINSSGLGNDVVENCDEFLD

PRQTSPETERILSRLMSGGGGTGQNASVNEALQSQFPTESAKHGGAEIFGHSDQRRQQQQ

NSNYSSGSNIMYHNSVASNSGIENTYRMESLPQMKIGGGGGGGGGGGNSNLIRHSSSPAG

LFSNINIENVSSFKWFCGVNIEIIQQLAGYGVMRGMRNFGGGNGTNAEASFPSPSRLDGK

SQIDFSSAPSSSLGRMTPILENRSKSMEITGFPIGSWDDSAIISDSFLKRIEEDEDRKTF

SAMNNASENQNAEGGMRPPHVLSHHLSLPTSSAELSAMENLLQFQDSVPCRIRAKRGFAT

HPRSIAERVRRTRISERMRKLQELVPNMDKQTNTADMLDLAVDYIKDLQSQVKVYNTTFE

YFRILKRSASVRISRGHSTFGMACFYVERTETGMKIESKLVVHLGKIMSSCSLIIGQMVH

YRFGILVQKDGESV

>RsbHLH112

MDEIIQPSSSLSLQQRLQFIVQSRIERWAYAIFWQATKDINGNFLLSWGGGHFQGTQQYS

VPNKLANNGDDQHKFGVKRGIQDNYSDIDHLVNGHVPDPEWFYMMSITNSFVAGDGILGQTFSSGAYAWLASEHELKLYDCERAKEANVQGIQTLVCISTAYGVVELGSTEIIQEDLDLL

LLAKSLFGPNNTTSVRKQPIGPGETSTSTCQSYSGRSNFKGLLQSDLKEVTKRGRKNSKA

RGETPPNHVEAEKQRRHKLNSLFYVLRGIVPNVSKMDRASLLADAVAYIKELQENVGELE

AKLICAKSQETKISFPNKHDVNQSTSITRVDHRHSGSTSLSGNGNGIMSAEVDVKVMGSE

AVIQVQCMDVNYPVARLMDAIRVLECHVHHATISKVKELVLQHVVVDQVPDGLRSEAALKIAIVRRFLI

>RsbHLH113

MDEIIQPSSSLSLQQRLQFIVQSRNERWAYAIFWQTTEDINGNFLLSWGGGHFQGTQQCS

VPNKLANNGDDQHKFGVKRGIQDNYSDIDRLVDGHVPDPEWFYMMSITNSFVAGDGILGQTFSGGVYAWLAGEHELKLYDCERAKEANVQGIQTLVCISTAYGVVELGSTEIIQEDLDLL

LLAKSLFGPNNTTSVRKQPSGPGETSTSTCQSYPGRSNFKGLLQSDSKVVTKRGRKNSKA

RGETPPNHVEAEKQRRHKLNSLFYVLRGIVPNVSKMDRASLLADAVAYIKELQENVGELE

AKLICAKSQETKISFPNKHDVNQSTSITRVDHRHSGSTSLSGNGNGIMSGEVDVKVMGSE

AVIQVQCMDVNYPVARLMDAIRVLECHVHHATISKVKELVLQHVLVDQVPDGLRSEAALRIAIMRRFLN

>RsbHLH114

MQSDQRFFPQNPVFPHSNQVCDSYMFNAHNESVLGGTILPTGLKHSGPLHGVEFQPSEVC

PKNFIIFDQTQNRSQIMFHPAIATKFCYPGFNVHSCYAHDNVEQEENIEKENPSSLREDS

DDINALLDSEDYEQGDEDEVSTARTQGNYGSDSSDSCSSYGSKPRKSKLPPSQKSSGGSS

CNSEGKRQKMRKMVKVLRGIVPGGSNRMNTAAVLDEAVRYLKSLKVEAQKLGVANLNN

>RsbHLH115

MVSPENTNWLYDYGLIEDIAVPASNFGATNSGFSWAMQPLNGPSDVGYENDGSFGDSDNHRETGSKKSIDVTAILTLRCVLARPESCGTSSSKACREKLRRDRLNDKFIELGSILEPGRP

PKTDKSAILVDALRMVTQLRGEAQKLKDSNSNLQEKIKELKAEKNELRDEKQRLKAEKDKLEQQLKTMNAQPRFMPPPPAIPTAAFAAPGQAAGNKMVPIISYPGVAMWQFMPPAEVDTSQDHVLRPPVA

>RsbHLH116

MAEEFQIGGGSWWGSSRTRFEGGTTPSSSSGLISMSNSFGWPAEIMDLNYKAISSSNFTN

SISASVSSSPMPFQDTRKLAQDPNSQMMGLGLSSHAMDWHQNNPRRGGEKAETRYLPGLQQNTSFAANFDQANEYKQFSTPNMSSSFQIDPAAYGSLSSTISSQGTVLGSDQNQHRPNFM

YPTNNYGLNNPGELSPSLSKLPRYLRNSPPKQQSNSQLQFSNDAPSWNAASASMNDVRSS

TFFSPPTFDDRPKNKSVVRDLGKVATKKSSSEASNKRSRSETSTSLPPFKVRKEKMGDRV

TALQQLVSPFGKTDTASVLTEAIEYIKFLHEQVNGFDKPRDCKGPKQNLRSRGLCLVPVS

STFPITCETPVDYWSTPPYGATLR

>RwbHLH01

MTDYRLSSTMNMWTDDNTSMMEAFMTSDLTSLWPPPPPSSSTSTSIHLPPPQPIAPFNPD

TLQQRLQALIEGARENWTYAIFWQSTNNDFPGGPAEEQAHRKKVLRELNSLISGASPSEN

DAVDEEVTDTEWFFLVSMTQSFGPGFGLPGQAMYNSSPIWVAGAELLSGSHCERARQGQVFGLRTMVCIPSANGVVELGSTELIFQTSDLMNKVRVLFNFNDTDMGSWTVPADHGETDPSSFYLTDPSPSVVELNITPANTMIPPSNPHLSKQPTFGSSTLTENPSSNPHQMQSQSFFTR

ELNFSGNXGISGARKGNSQSCKPESGELLSFGESKRSITATSNGNGNLFSGQSQFEEDNN

RKKKKSPTSRGSNEEGMLSFTSGVILPSSGVVKSSGGGGDSDHSDLEASVVREADSSRVV

DPEKRPRKRGRKPANGREEPLNHVEAERQRREKLNQRFYSLRAVVPNVSKMDKASLLGDAIAYINELKSKLQASESDKEEMKSQMEVMKKELASKGAPPPDHQDLKMSNQHVMKLVDVDIDVKIIGWDAMIRIQSSKKNHPAARVMSALKELDLDVHHASVSVVNDLMIQQATVKMGSRFYRQEQLXEALSAKIADAR

>RwbHLH02

MLLFFSGKVGDGFCPFGCRRDNDFVELVWDNGQIMMQGQSIRRIPIPNNFQSQTMKLRDK

DPGNVTNFKTGKFGTLESGFNDFATAMPSGEMGLSQDDDTVPWWSYPFSDSLQHDYCSELLPELSGVTVNDISAQNSFSSIDKRNSCNQTIRGTTSISGQNGFSLELVNGSNVSSSEVGK

SSRSRSGQFFPWSFQESPAPIPSLRSGVSGVISNDTSNTKHDSCGDSIQAQLSADDSSSI

KIQKQDSGLPNASSSFMNFSHFSRPAALVRANLQNVGSMGGENKGTAPSSGILAEPMPVD

LSNGLRNEMGSYSQPNSVPPKVDAKPVVAKPLEEPHAIYQEDSDKNDKLPNQVLGASASE

GMPDGEKTRDPLVACSSVGSGNSAERASNDPAHNFKRKYRETDDSEGRSEDVEEESTGVR

KAAAVRGATGSKRSRAAEVHNLSERRRRDRINEKMRALQELIPNCNKVRNYSLHFLANLNICNLYRRCNVDKLLAPFASQLHLDFDVIEVESILYSVHDLLPHAWLMDYALWYYKVDKASMLDEAIEYLKTLQLQVQIMSMGAGLYMPPMMVPTGMQHMHAAHMAQFSPMAVGMGLGMGMNGGSLGWPTIQVPSMQGPHFSAPRPQPILGTTNFQGMGVSNLQVFGNPGQGLAMSSPRAHLMPLSGGPPINLAGGLNANGTAAAVEVPNLAPTTNSKDLLETTNSQMVHNTDANSSMNQESSQVCELYVMKNSIS

>RwbHLH03

MSQCVPSWELDENPSPPRLKSTLRPHSNSNSNSIPPDIIPMLDYEVAELTWENGQLAMHG

LGPPRVHTKYPNWDKPRAGGTLESIVNQATYLPQGKSGSGDELVPWFDHHRPAASSXTMTMDALVPCSTTSGGGGGGAGRGTASTWTHVMDGLGKHVVGCSTRVGSCSGAKTCDDEVLLKRERVARKQGAALEWSCSRDNNSVDGSATCGRESWQLTLDTCDRELGTMSGFTTTSMGSLENTTSSALQSTKATTTATTDDHDSVGHSRFQATLLIYTPSKRKAGDVQEKKKGGGKSSISTKRSRAAAIHNQSERKRRDKINQRMKTLQKLVPNSSKTDKASMLDEVIEYLKQLQAQINMMSRMNMSSMMLPMTTLQQQLQLSMMAAAPLGMGMGMGMGMGMGCVDINTLGRHNIPGIPPVLHPAATAFMPITSWDGTAGXDRLPAPAG

>RwbHLH04

MKNRNQQLVLEELSCYSRFICFDLSVFFSFVVGFADRQRPEVEVKDPNAARKVQKADREK

LRRDRLNEHFIELGNALDPDRPKNDKATIINDTIQVLKDLTTEVNRLKAECAALSEESRE

LTQEKNELREEKATLKSDVDNLNLQYQQRMRVMFPWGTIDPSAVMAPPYSYPVPIPVPTG

PIPMHPVLQPFPFFQNQNPGAIPTPCSTLIPYPGPPNPPMDQPXYQCASTSRVSSKQDSK

SKTSDHCTGSIDDKSDNSNDVVTELELKMPGSKAQQEHSPGGTAGKQATRKEKSIADGNS

SSSRYSSSPGFQDSISDGIEHRED

>RwbHLH05

MQPSGNAMTWTERDEEEQEDAVSWTKNSNNHQADSKDNSNMNPSLSTFKSILETGWYTNPSHFQTLSNPHQDFKDTNITFCSNPAFQPENLMLQPMDSSSSCSPSQAFNFDPLSQPCFSS

LNAISNSPFDGGFDLXGCDPSFLANLSSNSPVFMGLNPQIEMGNNSQLSQNSELTHLLPM

PNNNAVISGGFGPNDFEGFGDALFLNRSSSNKVLRPLEVSPPVGAQPTLFQKRAALRQSS

NELGNLGISELRSDGIWGKRESVVGELSKKRKRKEEEEFEEGSIDGSGLDLDSDEFVDND

YKGDENGGDNGGNNSNANSSVTGGDQKGKKKGLPAKNLMAERRRRKKLNDRLYMLRSVVPRISKMDRASILGDAIEYLKELLQRINDLHNELEATPPGSLLQPATSFHPLTPTLPYRVKD

ELGPSSLPSPKNQPARVRFI

>RwbHLH06

MSLETLSSNEILNFLMYDTVFASKFDCNDSSDTTMLPETSLKPPQFGGASVISSSMMQRC

PPVGPEAVGKRRNPAMQGRKKRRRRQNVCKNKEEAETQRMTHIAVERNRRKLMNEHLAVLRSLMPESXVRRGDQASIVGGAIEYVKELEHLLQSLEAQKLLLLKGGTPPHDTATAATAEFFPPPFSRFIVFPQYTLSQIPNKNSSKSKTEVADIEVTLIETHANLRILSRKSLRQVSKIV

AGFQTLYLTILHLNVTTLDPLVLFTISAKVCNYL

>RwbHLH07

MNRALPEMLHCINTPGNIAGSFTDMSVLERQRARIKWQQEQLLNQPPCYFEGNDQFNGYSMPNHAQDFHNLISSEVGLVVKADPGLDNGWQEFGQFGGEGSGLGSSGFVNGRESVSPEKMSSAVGRESFKKRKADRNQSLKAVAEEEPNEKRIKECNEEDESKITEEYSNNSKNTTNNDNNIKRETSADTSKVSEVQKPDYIHVRARRGQATDSHSLAERVRREKISERMKYLQDLVPGCNKITGKAGMLDEIINYVQSLQRQVEFLSMKLAAVNPSLDFNIDSFFQKR

>RwbHLH08

MDHFSYYNQNNTHTNSSNFASLETHTKQKVGNDKNGKKSRKELKLSTAPQSVAARERRHRISDRFKILQSLVPGGAKMDTVSMLDEAIHYVKFLKAQIWLHENMFSIMDDYCVVDPSLLFSGHEQSDLCWPENVVGGGVELSPPFRLPEVYFQGGEAMGFD

>RwbHLH09

PEAELQESVAARKFQKADREKLRRDRLNEQFLELGKVLGASVGASLGTEPEVVITLLGLG

LGLRLWNLDVIAYYWATGCWLDGHFLDLEEEGVRSKCLVGCKCAFLSMKKISVITRYLLL

TYDVRTLTIFLSILFGVLAEPVRWIENKSYYYVCILSYKLQRALIDSSILPYADPDRPKN

DKASILSDTVQIVKDLTAQVNRLKAEYATLNEESRELTQEKNDLREEKASLKSDIENLNA

QYQQRLRATFPWAAIDHSVVMHPPSYPLPVPLPMPIPPGAISMHPSLQPYPFFGNQNHGV

IPNPCSTFVPYVTPNPVIEQPPTQYVSPAMHPSNRFNIPSKQDSRNNQSDCQGESKFEKS

EASDDVTTDLELKTPGSTPDEDSSSGQRKSKKLLTKENSHTDVSSSSGCSSSQSAQDSSS

NSVAGGGKDKD

>RwbHLH10

MSEVGEHENFVWESQSWAFANSDNSGSGGKPPDLGSNTQTPTGKEVEVVQAATGGKKRSGGGKKKGKGSGGGGGGGGSEERNEGKSGGESDHEIHIWTERERRKKMRNMFSNLHALLPQLPPKADKSTIVDEAVNYIRTLQQTLQKLQRKKLERLHGVAPINYEPSVVTPQKLAIDSREAFVADQVSSSNLANNSTPANSNSSNSVLPFVSRFPPIFQTWTSQNVILNVCGEQAQINICA

PKKPGLFTAICYVLEKHRIDVVSAHVSSDQNWTMLMIQAHARIAHDQLPEAFPVEEIYKQ

AAGEIMLWLSS

>RwbHLH11

VEEYHYRDTNSSSPDVCAGDGLLAGAPVEAPARRKRRRNYKSLKNKVEMENRRMAHIAVERNRRKQMNDYFSVLRSLMPPSYAQRGDQASTVGGAINFVKELEQLLQSLESKNQLKQQSESPRLFANFFTFPQYSTRXTHQXNLLLTGHESRVTXEKESTIADIEVTIVEXHANIKVLSR

XQRKQLLNMVSWFYSIGLTILHINVTSTVDQLVLYSFSVKVEDYCQLSTVNEIATAVHEM

VTKIQVEVMPSYN

>RwbHLH12

MNGASASGGIKSAFSYCVQQVRNYDYHHYLCLLELPPSMRKAAFALRAFNVETARAMDVASDPKIGLMRLLWWREAIDKIFAHKLIEHPTAQALSSVISEHKTSKSWLKRSVEARINDAQREVNEIPETIEDLEKYAEDTVSTILYMTLQAGGIKSTAADHAASHIGKASGLLLLLKSLP

YHASRNRLCSYIPSKVAANHGLLVNQGGPLEIRMDSREGMCDAVFEMSSAANAHLQKARELAATVPKEARAVLLPAVPAQVLLDSLNRVQFDVFDPRLATGILGPVFEMHSGSVEFPSNR

GSCALVERSRYVVSLLHMLGYEIKEAIHHLAFSCGAACIEELGKGFLEWMDCCCAGSENI

QNGGEDPFTVSSVTHCRDAMFQHPRTKPCDLLAEFPPSIPLDSGCNSGDLNLKRRIYAQA

LMSNQARWLNFSASNSDSNPLEVPEDQEIIDYIASQCTMLLEQQSTINSSSMDPNFSMNE

NNQIDLTNNPFLPSPENSNLPYDITADRIQLNSPMNFMQPSFFDGSNYEDDAKYRRRTGK

GPQSKNLQAERKRRKKLNDRLYTLRSLVPIITKLDRASILGDAIEFVKELLKQVSDLQLE

LEEQSGDDEGTENNGRSEILNAGLKREHENTLVGGNNMGAASYGSVAEPTNRKHDSDAYDHKSHQMEPQVEVAQIDENEFFVKVFCEHRSGGFVRLMEALNSLGLEVTNVNVTSCRSLVSNVFQVKKMDSEMVQADYVRDSLLEITRFGGWPDQVAKTSKNDQDGMDCHHHDHRSGGDRPNGLHNHQTSSHNLHHLHR

>RwbHLH13

MFVAGTMNLVQKLLERLRAIVNFKSWDYCVLWQLADDQRFLDWVDCCCAGSENIQNVGEDLFPVSSVPHCRDETVGTRVLIPVPVGLVELFVAKQVPEDQEIVDCIASQCTMLLEQQSMI

HSSSMDPNFSMNENNQMDLNNNLFLSSPENSNLPYGVTADRMQLHSPMNSFQPFDHTSETRASSKAAFFDGFNSNGLQVTDSTGQPMTMESSSLVLNKETDKDSVKHETGRSESISDDSDGNEDEDDAKWRRRTGKGPQSKNLVAERNRRKKLNDRLFALRSLVPKITKLDKASILSDAIEFVKELQKQVNDLQHELEEQSDDEGIKNNGQSGKLNVGLKCEHEETPDGNNVGAANNGVADPKKQNHDASIYDHKPRQMEPQVEVAQLDGNEFFVKVFCEHKSGGFVSLMEALNSLGLEVTSLNVTSYRSLVSNVFQVEKMDSEMVQADDVRDSLLEITRFGGWPDQMAKTSENGDGIDYHHHHHVHGGNCSNGLHNHQTCSHHLHDLRH

>RwbHLH14

MLHSFASTYICLWSYLPQPSNCLISTDGLYIGENNQPSSSSGTGSRARRLFDEYRQGLFV

LGTDHRVPGIAFRNGVPYMELRELDLLSLATIDPQLQFYQEARIKARLIILYATSISQVN

LEMEMRSWFPXDFSRQLAVPPIELPPTDQPRASSSSSSLRSLSMSSPEPSPFLFNIPSIT

SSLLEPPIEPSNIGQVVRPLSSSTGPLHQAKQAFSQFPSFQFPTPETTATTTTIEAGTSQ

KPSAFKRFRSALGPSTVTIRAGVRKPNIQKRAITFFRSLSLMRIQGRXQGSRPMSXTQLH

HMISERKRREKLNDSFQALRSHLPPGTKKDKASVLASTTDYLASLKAQVEELAKRSRDLE

EKVQLLATKKDHDQELSLSPDQRLDVRITQEGESTSQARVFGLQVVLRGECNVMDLVTRI

LEFLRRVENASLLSVEADAQVVDSNPINIVRLRLRIEVWYYSTFH

>RwbHLH15

MEFTQQGLLDELLAAPRRDIWTNFPSIDDFSNGWIFESLEHESQVLSPPNPQFLGFTSPT

EPSFSFPLHGLHYPFADTFTVPEIDSSYDSNAPPPIPFPTQEEHPSMVKNDGEYGFVGSD

LYTLEDRLNGCKVEMEPTANTPEFNMDFGRQRKNRAKKVEGQPSKNLMAERRRRKRLNDRLLMLRSVVPKISKTDRTSILGDTIDYMKELLEKVKNLRGEEDAKGSMNQVQLMGKSKELKPNELLVRNSPKCKLLVQSQGMQQQAIISSEDIKQALFRNAGYGGRYL

>RwbHLH16

MYYRESMCLQGTEDPMFVDPCDFMASMDEEFFNIPSNSSATNLSSASISSNTDLAPQIDE

LQPSKQYHMAKTFTSSPSRFILSFGNSGNNPTENPQQVPMNLEDEVVSSFLISQQRSSFV

NHEEGITKSGRLGMKRKSSGSSSSNPSPRSHTYDHIIAERKRREQLTQLFVALSGIVPGL

KKMDKTSVLGETVKYLKQLQERVKKLEEQPAKQKTMESVVLVKKSQLSFGDESCTSDEKFTGGSNIKPLPEIEARVCNKDVLLRIHCEKHKGVLAKLLLELEKLDLVVVNTSV

>RwbHLH17

MEVELMKPSTLDQTEMMKMMMQLDMLPQLSLPQTNTSELPEIKIYGENAAAAAAAAAAVNRTTEKTPIFHNPFLTSTTFTNPPSSMPFLRTPFQEPMTPPLSSTSFRTDSTIRSLSPPTI

TNQKYFETLSQTILGSNFPKAQKNMLIWPEASENLVPLLTDPLHERNSIGSMREMIFRMA

VMQPIQMDPGSVKPVKRRNVKISRDPQSVAARHRREKISERIRILQRLVPGGTKMDTACM

LDEAIHYVKFLKKQVQSLEQVRVNVSFDGVGLIQKRAMLAXVNYSSLVKALPGYSNGGLYSNA

>RwbHLH18

MALETLDFQQDPFNYGHKDLYTKGASYGYWDSLLSNADSLLPDVDLTGNDGVATGICPSPVGAPVAVQGRRKRRRTKSLKNKEEMESQRMTHIAVERNRRKLMNNYLAVLRSLMPPSYAQRGDQASIVGGAINFVKELEQLVQFLESHKQAKQKPYDSPPFANFFSFPQYSTRLTGHNGSVTGQDQYSTAEKGSTVADVEVALIESHANVKILSRKQPKQLLRMVAGFHSLGLPILHLNV

TTVDQMVLHSLSVKVGDECQLNTVNEIATAIHEMMGKIQEEAISS

>RwbHLH19

MKPRINEETYVDTSNQIADYTSLTSHPRSSTSPDKLSFADVMQFADFGPRLGLINQTKIP

EEGQEEEVETGIDPVYFLRFPVLNEDRYKGLGEERGGEEIGEGEGRGNSENNNNATSEGL

GIVGGNFEKNLAADDHGVSNKNKRKRARSVKTSEEVESQRMTHIAVERNRRKQMNEHLRVLRSLMPGSYVQRGDQASIIGGAIEFVRELEQLLQCLESQKRRRLYGDPPPPPRPMGDSSTSSLANIPQPSQPPPPFFPPPVVPSLLPNGHDNQLKLVEFDPTAGLREETAESKSVLADVE

VKMLGFDAMIKILSRRRPGQLIKTIAALEDLQLIILHTNITTIEQTVTSEARFSAEDIAS

SVQQIFSFIHANNTM

>RwbHLH20

MICGKKELEEAESFQEQLLLEQFPWSLPPIHYSFSPAHFDPNPVHDHNPFLLPPSVPSPY

GGLLFSRGSSEHDHLRLVSEAVGHAAAQYSSSSAPFGLQAELDKMTAQEMMDAKALAASKSHSEAERRRRERINNHLARLRSLLPNTTKTDKASLLAEVIQHVKELKRQTSQIAETSPVP

AEVDELTVDASDENGSLVIKASLCCEDRSDLLPDLIRTLKALRLRTLKAEITTLGGRMRN

VLFVTAEEESNVTNSGTNTDRIAL

>RwbHLH21

MKSGQQEEVEKEDEVIVSKREGLSSNSEDGKNKNKANATRSKHSVTEQRRRCKINERFQI

LRDLIPHSEQKRDTASFLSEIAWATYEVSATMFCLKQRNSHWRAQSLVGPTQAVKNDSGP

ASTFPGRFDENNITCSVAMHPISWNSTESDSSRDPSGQIDDQQSKLANKAIALPIPLHTN

VSSSGPGDDVVLHPIHRPISDAQSTECPITSGALTEQEDLIVEGGSISLTSVYSQGLLNN

LTQALQCAGVDLSQSTISVQIDLGKRANRRLTSGAVVPNTKDSESASPSHQPIGHFRDST

NGEELDQAQKRLKI

>RwbHLH22

MGVCDNLSDHFFEESELEDIFSILENLENVEEFPPLEPENGLGSNEGETGGGLVSQKSTC

SSAGNLQLESEAEVELAADNYLPKRNKRQKLSTAATSTDEEIPQGEGQLNRISHITVERN

RRKQMNEHLTVLRSLMPCFYVKRGDQASIIGGVVDYINELQQVLQSLEAKKQRKVYSDQV

LSPRLIPSPRPSPLSPRKPPLSPRLPPNLPISPRTPQPSSPYMPRLMQHLSPLPSPCSSS

SSAPSSVVLDNATTNELVANSKSAIADVEVKFSGPNLLLKTISPRIPGQATKIMSTIEEL

SLEILHVSISTIDETMLNSFTIKVRYTITSSNFLFLPQSTAYSQHL

>RwbHLH23

MITARETQISRVEAERLRREKLNHRFNELRSVLPNVSKMDRASVLTDAVAYIKQLEAQRE

ELEAQVGVESQTAAKACVGSSSIAGGHHAVPSLGEVEVMIVGSEAMIRVQCVDVNYPCARLMEALRELEIEVYHASMTKVKEIMLQDVVVRPLR

>RwbHLH24

MASVPENLTKQLAVAVRSIQWSYAIFWSISSRHPGALEWGDGYYNGDIKTRKTIQAVEFN

ADQLGLLQRSEQLRELYESLAAGESSPQARRPSAALSPEDLTDTEWLPGRTLSKGQPIWV

CNAHYEDSKVFSRSLLAKSASIQTVVCFPFLGGVVELGVTDLVLEDPSLIQHIKTSFLET

PYPKVSKISNSFTGNANQGLIVHAKLDHEILDTNLNPVFQCEDFEVCSPNNSSNGLGINP

QPEESFLVEGLYEANSQVQSWQLMDDEVCNGVHNSFSSSDCISQTLVNPEKASPLPDEMT

KIGLQANDIHYQSVISTLLKSSQHLILGPNFRNSNQESSFVRWKKGVLSSIQTPKSRTPQ

FLLKKVLLEVARMHGDHMSECSREDSGRKDAIWRPQVDDIDSNHVLAERRRREKLNKRFSVLGSLVPSDPSTSKVDKVSILDHTIEYLRELERRVEELDSGREVSEPEVRTRKTPKDSAE

RTSESHGHNKKPSINKRKVRDIDEIKDPYNTTVSIIEKEVLIEIRCPWRECLLLEIMDAI

SHLHLDSHSVQSTNSDGILSLTVKSKFKGSSFASAGAIRQSIQRVVRKF

>RwbHLH25

MANNPSEAYPDEFLEQILAMPSYSSLAGTDGASSQSAASPLSFTGGMGLQHPFIPLGLSL

DNGREEINGGELGFEIATGLRIVLQVFHGQPTTSTTVSVPHPPSIRPRVRARRGQATDPH

SIAERLRRERIAERMRALQELVPSCNKTDKAAMLDEILDYVKFLRLQVKVLSMSRLGGAG

AVAQLVADIPLTSIEKNEGSTALFCRGQATGDIFNVIFIYCGTQRLLLGGSGTQGKRWDW

>RwbHLH26

MLFDSENYLKLRPNYEVAELTWQNGQLAMNGLSSGGLLPTGPTKPTWNRAGDTLESVVHQATWPNQNLYPPLPKRDHNPAXVSSAVGSTIVKRAETLGRMHVGPKRVRSETDQCGGSFGSSIQEERSACAAAASATFCKENDATMVTWASFESPRNLKTKTTDEDSAGDLDGSENLEDQERETTKGETGRSHSTRRSRAAAVHNQSERKRRDRINQKMRALQKLVPNASKTDKASMLDEVIEYLKQLQAQVHLMSSATRNNMPQMMTPTALGLHHQQQIQMSLLARMGMGMGVGFGMGMGPTFLPPPHPFVVPPMIPNHNLSQATTDAAAVRSSVPFNNPYCTFLGQPINMDLYNKMAAIYQQQVNHTSLQTSSPFPSTHVQGGKEHLT

>RwbHLH27

MGVLDRALEWLRPFVGTKIWDYCVVWKLGDDPSRHSRKSPNIPSFVYIEWVGCCCGGAYGDSNGIKIKEERPEGKPQLLIPQCRDTHIQHPANTKACLALSRFPPSIPLYSGVHGEAVLS

TEPKWLSCTEDSDSNQSHKLNGTQVLIPVVGGLIELFSTKHLPRDQKIIAFIVSQYNNTM

EQDSITTKSCSNMRFNVQSHDTLPGAHYPVNNWPALFEAKKIFPRLPFLPPVSQLNHLPT

LEGSSTGSNPSNEHSISFNSGSVRVSPNVSVNGTFGEYPTDDKSNKCRNLSPKQDHLVET

DKLKSKQRMEKEQYHSKNLVTERNRRRRIKDGLFALRALVPKISKMDRAAIVGDAIEYIE

ELHKNVKELKAELAEMEEVEHKKKNDELVIPKLNAKKGTKTSTTTADEKGKMEVQVEVHQIGTRDFFVKLLCTQKTGGFARLMEAMHSIGLQVVDANVTTHNGKVLNILKVEDIICCMAAFLA

>RwbHLH28

RSSQPPLQFSYDGLISESLGHVVQHPGSVPFGLQAELGKMTAQEIMDAKALAASKSHSEA

ERRRRERINNHLAKLRSLLPSTTKTDKASLLAEVIQHVKELKRQTSLIAETSPVPTECDE

LTVDTSDEDGRFVIKASLCCEDRSDLLPDLIKTLKALRLRTLKAEITTLGGRVRNVLFIT

GEEEEESNSGDNHQQQYSISEIQEALKAVMEKTNGDDSPSGSVKRQRTNVNIIEHRSL

>RwbHLH29

MLSWSPPLFSPFGWPLEDSVSITHEENNFFTREAEANSSTPSFLNSPPLLSHEPLIAVNS

GEGECKNSNDDVDRECDDNIATTTPTITAKKLNHNASERDRRKRINDLYSSLRSLLPAAD

QTVEESILISEILLNLEEDGLILLNASCFESFGDKIFYNIHLEVQGGQRVEFETLRERLL

SFV

>RwbHLH30

MERGFQALLLGGESSDDDYSPPAFTGTTADDACISALYSSAMNAQTSKSFLSDEKYLLQS

LEYGYGAVPAAPSVDPVACGYESYDVVGDWKLKSVGYADEKLKVPTYETVDYESVSDVTELPFDFLSKSSGSVSREFDSRFSQLPGMNSQTSSFLSLSDEMYLLQSLEKYGYGTLPVAYG

YDVVEDSKLKYVDYADDKLKMPKYETVDYESVSDVVSAEPQFDFISKSSGSVSREFDSRF

SQLPAMKARTSSFLSLSDEKYLLQSLECGYGALPVAPSVDKSVEYVDVKLKVPKYEPVDY

ESLGELVSTEPPFDFISKTSGSNSGVFDSRFGKLPNLLSLEPVHDSFGLTESPKKRVRSL

SPATSNNSYFTGSIPQEIELMKARKRRQTISEKTSCLQKLLPWDKKMDRATMLEEAYKYV

KFLQAQVSVLQSMPVDSSFSTRNPAGNGAVNVFGGLGRLNRQQLLQVMVNSPGVQTTLYSKGLCVXSVEQLVTMKKVAERKALYQQLGWNPSLIS

>RwbHLH31

MVSPENTNWLFDYGLIEDIAVPASNFGAPNSGFSWAMQPLNGPSDVGYENDGSFGDSDNHRETGSKKRQKEFHLPFLPIVLTALMSRPSSLCVTNLNPSSHLFPHLFLFLGYRARSELSG

ASSSKACREKLRRDRLNDKFIKLASVLEPGRPPKTDKSAILVDAVRMVTHLRGDAQKLKD

SNSCLQDKIKELKAEKIELRDEKQRLKAEKERLEQQLKTMNAQPRFLPPPPAIPAAAFAA

PGQAAGNKMVPIISYPGVAMWQFMPPAEVDTSQDHVLRPPVA

>RwbHLH32

MIMMQMEKLPDFCSGPYTDLGVQLPPGMEFXGGSSSSSSQGHVLPFTDNSSPTGMNLPST

LSFSGVQEISGGRWRGGGGGGEFPGGLGFPSQSQKRNSMAAMREMIFRIAAMQPIQIDPE

AVKPPKRRNVKISKDPQSVAARHRRERISERIRILQRLVPGGTKMDTASMLDEAIHYVKF

LKTQVQSLERAATNRPAAGLGFPVAVGSGGFVPVAKGFQLGSQGVQQYADG

>RwbHLH33

MGTEENGNMGFQQRNGDSILNCPTSGMNTTNPFFGSGWDPLVSLSQSENFGGSFGNSNYS

HLVHYPSDSGLVEMVPKLPFFGGGGNFSEMVGSFGLPNYAPNEEDGTEKTSTNGGDQQVS

EEGTMGISPCGKRKKRVSESNSPFNPSKNSEGEQQMEISGDSSQEEKKQKSEQNPSANSR

GKQTGKQAKDKSDSGEAPKENYIHVRAKRGQATNSHSLAERVRRERISERMRLLQELVPG

CNKITGKAVMLDEIINYVQSLQQQVEFLSMKLATVNPELNIDIDRILSKEILHSGASNAA

IPGFGLGMSSSXPYPPGIPQGVLSGIPSTSPYHSLPQNLWEHELQSLLQMGYDSNPAIGN

LGPNGRSKLEL

>RwbHLH34

MEDVGFMNHWPINCLNDILNHPISPQIVGVKPNSMEYSSHARNTWNSSGNSSSTHQISFS

NPNYETQSATIKPKEKAVPAGNITTLPSGVLNFQGSLGNPISGFKACQGSKRNCTNSKRV

SSSQEHVLSERKRREKISQRIIALSAMVPGLKKMDKASVIGDAIDYMKQLQEQVKTLEEQ

ASKTISMGSVVYLRKCELHSDHWDNLSLENSNYTGGGGNSGAHYDEPLPEIGARSCDKNVLIRIHCVKRKGVVEKTVAEIEKLHLSVVSSNVMAFGSYALDMTILAQV

>RwbHLH35

MEYDGHGFLEELVATTIPIEMSDFDTNKWSFGGFDESTFHVPTNFPLEPLDQSLNCSINE

FYFPFGGDPFQFSAPGVTDTPPFPTPDDCSMSMVENEEKPQMGNGVQMLEFQSPNNCKLE

RIQSREVPVFDMGLGAERKNRVKKVDGQPSKNLMAERRRRKRLNDRLSMLRSVVPKISKMDRTSILGDTIDYMKELLDRINNLQEEIHEVDNSNQFNLMSIFKDVKPNEFPWQFDVEKRNMDTRIEISCVGKPGLLLSTVSTLEALGLEIQQCVISCFNEFAMQATCSEELEQRAILSSE

DIKEALLRNAGYGGRCL

>RwbHLH36

MLPHQNFSYGLQSIWNEESLVQKQDSMKRMGMDRDSISRAQKHKSEAAAKKHAKAEQLRRKRINTHIQTLKKLLLPNMTSKKVKASILTETIRQLKELHKRAADVALQQYGHADVSGCFTFPDGSDEVTVSRCEGGEGKTVKATVCCEDRPGLNQDLTDAIRSAGGKPVRVEMATVGGRTKAVVVVRWVEGGGEEEEVGLLTRALKALVESGPLGFSRLGQIGRVNGRTGLGLEMSRNERARMCSSFEGEREWEKLLLSTV

>RwbHLH37

MALETVEYPQDPFGFLGIFDTIMDHGNNIIGANWDSSSSSVRQNVMECWECPNSSPEACT

GDRFFGGEGPPVEAPSTEAVRRQKRRRTKSSKNKEELESQRMTHIVVERNRRKQMNEYLAAVRSLMPASYGDQASIIGGAINFVKELEHLLQTLEAHKRTTPQQQQQQQHPPNNNGQSSTSLFADFFAFPQYSTSSKNNCTDTSSPAAADKPRPVAFGDIEVTMVESHASLKVLSKRRPR

QLLKLVAGLQCLRLCVLHLSVTSADQMVLYSLSVK

>RwbHLH38

MASGSCHSSSTPKIERRVIEKNRRNHMKSLYANLYSLLPSHASKEAVPLPDQIDEAVKYI

ESLEMKLKKSKEKKETLSGRKRSHSCTSEFKSPQLEIHEMGSTLDVVLITGEDDKFIFHE

IIRLIHEEGAEVLNANFSVFGNSIFHAIHAKAGEFEKNVVAPRVSSRLKEFVHGSMSDVE

SVLPAESWEFDIEPDAWELKVPQVLPMRLY

>RwbHLH39

MADIFPLQAQPELAMDLLGFQGNFNDYNYPSKMFCLANGFFLNPRPLVPGFSPGLELPLS

AQSVAVRNRRRKITEKTRELGKIVPSGEKMNTAEMFQAASKYIKYLQAQLGILQFLASSI

QVITLC

>RwbHLH40

MEFSSGFSWNPVFQEIIRRGSSSSTSSLVLDGERGELVQAMVRPSHKGVKAEKALEALRN

HSEAERRRRERINGHLTTLRNLIPGTDKMEKASLLAEVVNHLKELRRNVTEATDGMLVPT

DIDEVKVEQQEDGSDGASCAIRASLCCDHKYEILSDLRQALDALHLQTVRAEIATFGGRM

IYVFIISCCKEKKMEDIEGCRLLVSSIRHTMRSVLDKFYASEEISSRNTLSNKRRRVPFF

ESSNSSSIGDLW

>RwbHLH41

MYGNSMEIDSTTENSIWLFDYGLIEDISAIPDGDLPAAPPVTACFSWPSQEINSCSNVSV

DIDSSFEDSEAVEETGLRKRLKSESCNASGTKACREKSRRDRLNERHALCFFFTHWFGEL

GSVLEPGRPPKTDKAAILGDAVRMVRQLRSEAQKLRESNEDLQEKIKELKAEKNELRIEK

QTMKEEKEKLEHQVKALTSHPGFLPHPFAMPVAYGAHGQTTDHKLMPFITFPSAAMWQFI

PPTVRDTSHDHVLHPPVA

>RwbHLH42

MVSPENTSWLFDYGLMEDIAVPASGFSWPVQPLDGSSKWGMDILLLISCPLDGSSKSGDQ

EQIYAPVILFDCSYSICLLTSFSCGIWYVSSVEIDSFGESDSLKETGSKKRTRPESCGTS

SSKACREKLRRDKLNDKFIELGSILEPGRPPKTDKSAILVDAVRMVTQLRGEAQKLKDSN

SNLQEKIKELKAEKNELRDEKQRLKAEKDKLEQQLKTMPTQPGFLLHIRESQLHLLLKAA

KISCQSWATQGLPCGSSCRLLPWIPHRIMCSPACSLVGALA

>RwbHLH43

MELEAILSQKDHFGYTSKDHYDLLEGNWGSEFGLPEQEEYGSFEFHVGQTENSNHTSWDSPPPTSSMWPYSYLDQWGANNPNSSSEVPDTTSIYPREEFQQLDPISTRPRPRRSRSKKNI

EEIESQRMTHIAVERNRRKQMNEYLSALRGLMPDSYVQRGDQASIVGGAINYVKELEHKL

NFLGGEKRTNQKFEAGSSSLPFDEFFAFPQYSTSTTTTSCESSGDPISANADIEVTVVES

HANLKIRTRKRPKQLMKMVSGLQILRLSVLHLNVSTFDQTVLYSLSVKVEDDCSLTSVDE

IAAAVNQMLGRIQPEAALL

>RwbHLH44

MALELEALSTNESLDYIVYDTISADPTFLPENSLKKPWELGCGASDSSSCMMRRCRQVGS

PEVSKKRRNAVAQGQKKRRRRPKICKNKEEAETQRMTHIVVERNRRRQMNELLAVLRSLMPESYVQRGDQASIVGGAXDYVKELEHLLQSLEAQKLILPQQGGIRPPTTTAATAESFEAP

FAQFFACPQFTWSLLPNKCLSKSAAAVADIEVTLIETHANLRILCRRNLRQLSKLVAGFQ

TMYLTVLHLNVTTLEPLVEEVCQLTSPDEIAGAVHHMLIRIIEEEGT

>RwbHLH45

ISSIGHYSSCCGGADLGLLFGVAIMASYISSIGHYSSCCGGADLGLLFGVAIMASYVLIV

LACSWQEAKRVTSELFGMDFVSSAFQCDPIDELLEFSSIPGQQRRIQKDPLQVVSASRNN

DVAEKPDYHPWRKSSVVFDDSGGNPMDYKRKKIIHREAERQKRQEMAALYRSLRSLLPVEYVKGKRSMSDHMLVAVDYIRQLRKRVEEKGRKRDELKSSFEPSIDTNAKSRCFPSCLKES

VTVESSRAGFQITMSTAMSGGLCVSKVLDVLLREGLNVVSCISINAEERLHHVIDSEVRV

VNFIEEQCYMKG

>RwbHLH46

MRMGFKRCVTKQPSGPGETAASTCQLYSGRSNFKGLLQSDSKVVLRSTKRGXNSKARGETPPNHVEAEKQRRQKLNSLFYVLRGIVPNVSKMDRASLLADAVAYIKELQENVGELEAKLICAKSQETKISFPNKHDNQSTSITRVDHCHSRSTSLSGNGNGIMPAEVDVKVKGSEAVIRV

QCMDVNYPVARLMDAIRVLECHVRHASISKVKELVFQQVVVDQVPDGLRSEAALKIAIGR

RFLI

>RwbHLH47

MAGFAVMGDVGNFRVGNGTNGKASPTKRLNGHINFSSGTSSCSRFMPQISENANESSSPD

NEHLENDNSSNGFYVPSFPNDSWNDSGFTGQKRNRDGEEKFXGFNALESQHRDSRNYATGLTHHLSLPKTSAEMAAVEKFLQFQQDSVPCKIRAKRGFATHPRSIAERMRRTRISERMRK

LQELFPNMDKQTNTADMLDLAVEYIKDLQKQVKTLEDTRSKCTCSSKQNQSSNPSG

>RwbHLH48

MEELSYQEESAAAAAMEMELQQQLGLDTENCYANNHHSNLMDTSHLMQEVVGHDSNQVLIPNYDHNSSWDINNTNMNTHDFQEEHLMNHVNEMQNSHHLLNLLRLPKVSPSSVLPNSSFSLGFLGDHFPSTDGSGGSASNVAYDPLFHLNLPPQPPYFRDLFQSLPHGYNLPGSRSGSLFGGVEEREGSGGGEVGRNFDNGVLEFQTDWDMNGMAKVGNGKKNTKHFATERDRREHLNSKFTALRSLIPNPTKPDIMLTFFGVIFLAQVYLNNIRATIVGDAIDYIKELLRTVNELKILVEKKQCGRERIKRHKTDDGTSTTTAGEVLEDSCNKIKPDPDHSYSNGSSTLRSSWLQRKSQDTEVDVRIVDDEVTIKLAQRKRINCLLIVSKVLDELQLDIQHVAGGLAGDYYSFLFNSKI

GEGSSVYASAIANKLIDVVNRQYAAIPPTHSY

>RwbHLH49

MKVEVCMGDGGWSEEDKAMGAAVLGTRGFDYLISSSVSAECSLMSVGSDENFQNKLSDLVERPNASNFSWNYAFFWQISRSKAGDLVLGWGDGSCREPREGEESEYTQIAHSRLEEVTQQRMRKRVLQKLHTLFGGSDEDSYAFGLDKVTDTEMFFLASMYFSFPRGEGGPGKCFESGKHVWISNSLKTSSDYCVRSFLAKSAGIQTIVLIPTDVGVVEFGSLRSIPESFELVKSIRASF

SPFSSLAKAKPTKDVNAHFFKLGNGERPDGIPKIFGQDLYSGRSQLREKLAVRKAGEKPW

EAYSNGNRTPFPNARNGPHGSSWTQFQGVKQVAAAGIYSPHTPSNNLPEVINGVRDEFRL

NHYQPQKAAAPMQIDFTGGATSRPPITSVESEHSDVEASGKDELAGPVDEKRPRKRGRKP

ANGREEPLNHVEAERQRREKLNQRFYALRAVVPNISKMDKASLLGDAIAYITELQKKLKD

MESERENLGANPILENQTRVPDIEIQATGHDEVVVRVSCPLNTHPVSRVIQAFKEAQITV

LESKLATGNETIFHTFVIKSQGSEQITKERLVAAFSHESNSMQPL

>RwbHLH50

MDEEYSIPTPTSTCRPKKLPVVDPDEIMELLWENGQIVMQTQNQRSFPKXPLIEDGAVIS

LDRREIRSSRPIESGLSNDQLFMQEDETPFWLHHYPPIEAADLYSDVIDPAPPRTAPNQD

VRIPPPSTAAPMSQPRRSDPEESEAVQSSVRFSRAKARAVEQGPSGSNEAXRESTVVDSC

ETPRXAPESVASRVAGSTVEVSGGVAGLCVAAEGGGRDTGTYXLTSSSGGSASLEPVQKP

PVTAGDRKRKGREADDDDDSESPGEDVEFESPDAKKQIRGSTPAKKSRAAEVHNLSERVC

LYTDVIAAILFLCGSFFLMGLMLNVMQRRRDRINEKMRALQELIPRCNKSDKASMLDEAI

EYLKSLQLQVQVFATFCSYLSHDSILLFTCQSHSTSRLPSRNLVCHQFLQMMSMGCSMVP

MMFPGVQQYVPPMGMAMGMGMGMGMSRPMLPFPSVLAGSALPTPAAAALMGXKFPVPAFHMPSVPLPSPSNQSDPVLKSLSPQNHNQPRMMNFPGPYHQYLGLHHTQLPLPLPQVRLKSTFLSSNQAMMQPSTTKPSTSREVENH

>RwbHLH51

MDNRYFLSSGIIPPPFYSEQSPMPMWQSVSNLNSPSRLHLPIVEHLVKENMPNLGFPMAL

NPNLPTISGDPGFTERAARFSCFGSRSFNERTSQLLGSTESPYRSSSALMEREKMPRVSS

SGSFKGAGSPMSARENWNSTQTHTGMRCAGDFDMNLSQVPMSDRAEFPSSNEGSSVSGQIPNGETGSKGPCDSSSKKRKAASRGKSKVVEAEDDGNTKRCKSTEGNGIENDNVKTENKTDEEKGSPKPAEPPKDYIHVRARRGQATDSHSLAERVRREKIGERMKLLQDLVPGCNKVTGKALMLDEIINYVQSLQHQVEFLSMKLASVNPRLDSTNMDSIFPKDVSQPNGSFPHPVAVTQSSVHPLDTTYCQNIGTLVDELGQGLPQFPTLCEDDLQSIVQMGLGRSPNRDMGFYSQFFP

GKS

>RwbHLH52

MGDFNPLLGLMDETNFLQFIDIIRGESSDPIAKFCPNFDCEHIGGCLVDNQFESSTRLNP

FDHSHTDQSVSNPDSDLIIDTVLQLATDADEEENDNHEESSTTTTTTPPTKRKGGDRSRT

LVSERRRRGRMKEKLYALRALVPNITKMDKASIVGDALLYLQDLQTQAKKLRSEIAGLES

SLGGREKNQRGIVGNQKNTQYTNANHPICKNIFQLDVFQVEERGFYARVECNRGRGVAPS

LYRAIESLTRLNVPSSNLGTRAERFILTFSLKVKECEPDTNLQKLKLGLTEALLKQGFVF

QTQASA

>RwbHLH53

MALSVYNSNWAPLQHLNFPPDQILTNAAAFPPPQAALPELDMXLLAFQDHLFYHPDYPCS

TSIDPGLFHHPANSDLNILPCLALPPPPLDNSIPPQPFQYSYHYPKRQKCYEDEYYDYSY

YPEEEFMPSLKPDGFVANPPLLIPEFSLPSEIYGPVVPAFDYGGCGDQXASGGGGGGGGS

LSAQSIAARQRRRKITEKTQELGKLIPGGHKMNTAEMFHAASKYIKYLQAQVGILEFMGS

AAQENIHEESLVHTRELQALASSPSIQEKLYSSDKCLVPKQFVGTLPNYPDLQSNDXIST

ELHQLMRTSSG

>RwbHLH54

VGAVFGEFGGFGWELGRWVGFSVGCTSLVLDGERGELVKAPIGVGKKSVGLPEEKVVAALKSHSEAERRRRERINAHLNTLRGLVPCTEKVVCFSMNLWFLADFNSGCHLIVFKLFIANS

GELGSHQRFNGQSALLAEVITQVKQLQKTARESATGLIIPMDDDEVRVEPHHDEDGSFSL

RVSLCCDYRPELMSELRQALDSLHLNTVKAEISTLGERIKNVFVFTSRKDIDSENAESCQ

LLVNSVHQALSGILDKASASAEYSPRTTLPTKRRRTSYVDSSSSSS

>RwbHLH55

MDELIISPSSSSSFISSQETPPTLQQRLQLILQTQPDHWTYAIFWQSSSSSSSPNDQNTP

ALLSWGDGHFQPTNNKNPLPKDNENSAEWFYIMSLTRSFSGAGDGGGAPSKAFSSGSLVWLTGGNQLRFYDCERAKEAQIHGIKTLAFIPTSVGVLELGSSDVVKENWGLVQQAKALFGSDLDVIPNRGNGSSEFLDGNVCFAEEDMVNGGGTKQEAIVETECSDLDPFPVPTTFGFHHNAEPEQQQRTQKKRGRKPGVGRETPMNHVEAERQRREKLNHRFYALRAVVPNVSRMDKASLLSDAVSYINELKTQIQDLKSQLHDSKSSNKKMKTELSSQLTADTTDNHSSAATSVDQTISPNNNSDNNSSGVPLEVEVKIVGADAMVRVSSDNVNYPAARLMEALRDLELQVQHASASSINDLMLQDVVIMGPHRGVISEEGLRTALLSRLG

>RwbHLH56

MEESSHRHGQIIEISGEVSGGEKSVGGSKLCGEAPCGFSDAVTITKDAKERAASMRKLFI

AVALCVVFMGVEVAGGIKANSLAILTDAAHLLSDVAAFAISLFSLWAAGWEATPRQTYGF

FRIEILGALVSIQLIWLLAGILVYEAILRLINDTGEVNGFLMFLVAAFGLLVNIAMAVLL

GHDHGHGGHDHGHGHSHVGHGHGHSHGHGHSHSHDDHDHNGHGHRHGVRITTHPFSHEEHPRDEEHGHAHEEDLVEPLLKQNSHDKNKPSSEKEEKEKRNINIQGAYLHVLGDSIQSVGVMIGGALIWYKPNWKIIDLVCTLIFSVIVLGTTIKMIRNILEVLMESTPREIDATKLEKGL

CEMDDVVAVHELHIWAITVGKILLACHVKIKPEADADMVLDKVIDYIRREYNISHVTIQI

ERFYLILRASLQVAAASLFLEKASLQRSRVNLGKLERLASPPNDTAGGACFFLIYTDLCF

SELLGSTGNCWLVQYIDESNKEIIMLFIACGLWGFLEEEEEEHLDVEMECLEQETSNNTM

RKLGFVSPSDSSFEEMRIPFLEMLQSGDTPAFSPFCELSFQALLSLQQFKKPPWEGSHSH

HQYSPELRECSGGGRFKGQQDSCLTHDAVEVQISPVKSETMDHHNTHSAIYVEGGANSDG

NQHDGSKAVELEERPGEAAAWKGQKRKRVKRTRPSAKNKEEVESQRMTHIAVERNRRRLMNDHLTALRSLMPSSYIQRGDQASIVGGAIDYVKELEQLHQSLQAQKRMKKSDLLEQSGCRGSSSSETTSTSTSSSSTTTGILATSSPQTCQFGNMKSETGELGNCTTTEGINVRQSYEFT

AVRKSGGVEVHVTVIQNHVNLKVECPRRAGLLLNAIVALEDLGLTVLHLNIMALENSAQY

STWVTMRLCGLAPENNSGMGNSAHSCLDQETTEGGVAGGVGQRARSDDAYRYGFCFR

>RwbHLH57

MDKEYFLNAGIPPPLHFEPSQIPNWQSQSSEFLFNPNWDSNKSTDQQYSNFDSALSSLVS

SPAASNSTISQDNFVIRELIGKLNTTANSGEFSPAPYVGSRSTTSSPSKSAQYLVDHFGN

EKMLPNLGNSMGMNPNLVSTDPGFAERAAKFSCFGSRSFNGRISQLPYRSSSASMACSEK

QESDTNPCGDGPREWTDAGFGKASEQDSARFASSNEGSSVSEQIPSGLKTPSDSNSRKRK

AVSRGKSKEVASKAVEEKDDPNAKRSKSIEGSSELGSVKTEEDTKTGDEKANQKAPEPLK

DYIHVRARRGQATDSHSLAERVRREKISERMKLLQDLVPNCNKVTGKALMLDEIINYVQS

LQRQVEFLSMKLASVDFNMDTLLSKDEFQANGSLSHPMYPLDSSASAFFGHQTKETPQVHTNFSIGAMTQCSIDPNVCRNNLMQSTLVDEFGRGLPQFPTLCEDDLQSIVQMGFGQNLEL

>RwbHLH58

MDDDYAIPSSSGFTRPKKSPMAEEEIMELLWQNGQVVMQSQSQRSLRRPHVGDGVNPPEHAAAAKEIRTEEESATQLFIQEDEMASWLHYPIDDSFDRDFYADLIYPAPTAPVAPIPDDV

RVSTPPAAATAAAPRPPIPPPARRPEVESPARTQNFLLFSRPKKGRTEAGPSICNKAARD

STVVDSSETPAPGSTVSHAAAQVSGGNVWYGAAASGAAAVAVASSAAGEETGLASSPGGSGTSVSTSAEPVHKPVLAAVTEDRKRKGIEADDVECQSEDXEFDFPDAKKQARGSTSTKRSRAAEVHNLSERRRRDRINEKMKALQELIPRCNKSDKASMLDEAIEYLKSLQLQVQMMSMGCTMVPMMYPGVQQYMPPMGMGMGMGMGMGMGMGMEMGMNHPMMPFPSVLPGSALPTAAANARLGPTFPMPAFHMPQPVPLPDPSRIQATSQSDLMLNSMGTQNPNPPWMPSFADPYQQYRGLHHMQLPLPQVCLDSSPLTFQLELFM

>RwbHLH59

MANNTSDGPTDDFFEQILGFPAYASTDANLAGNSGTPMMLQLSSGDGSAHLGGGGGGGGGFHGSVFPLGLSLDQGKQGFREDVGDGRSSSSSMKNTFSGQPMPNTVSAMAHPPSIRPRVRARRGQATDPHSIAERLRRERIAERIRALQDLVPSVNKTDRAAMLDEVVEYVKFLRLQVKVLSMSRLGGAGAVAPLVTDIPISSLEQDAGESGRNQAAWEKWSNDGTEKQVAKLMEENVGAAMQFLQSKALCIMPISLASAIYHTQPPDATTVINKWNAILECIMVGCPVKAAVLQS

>RwbHLH60

MGEKFWLNEVDKGMVESVLGSEAFEFLVASASGKVLSEFASSAGDLGLQKGLSKIVEGSN

WNYAIFWQVSKSKSGNSALIWGDGYCREAKGSEVGTGNGPQDRKYEGGDRKKRVLQKLHACFGGVEEDNCALNLDLVSDVEMFYLTSMYYSFPFDKPSTPSQAFNTGRVIWASDTKSCSEHYQSRSYLANLARFETVVFVPLKSGVVEIGSIKTVPEEQNLVQVVKTAFAGPLPSQGKLVPKIFGHELSLGGTKSRAMTISFSPKVEDDSGFASESYELQGVGSNQVYGNSSNGCRSDEG

EAKLFPHMNLGGLEQGHEDLLIQSDERKPRKRGRKPANGREEPLNHVEAERQRREKLNQRFYALRAVVPNISKMDKASLLGDAISYITDLQGKIRILEAEKDMVNYKQKQGSVPEIDFQA

RHDDAVVRVSCPLDSHPVSRVIKAFREHQVIAQESKVSTTEEGEIVHTFSIRAQGGTAEH

LKEELVTALSR

>RwbHLH61

MDTLPGNISWFRRVFTERSVTEISREMSYHNAIVPSGGMDVWKVGGDAVLQEVKYARPQNWPPINSFTDAEYQLHAQGQESNELHSRASVAAPAFLAKPHNHASRRRASVTATATDRARRMRITERHHALNELLPRHKEGSKASQLDDITDYIKYLQLQIKVCFHYNVVAYHHLKGYGHYILHEQMLNEPLEEMMGKLLEVNPSAASRLLESRGLVIMPTNLVEGLHQAM

>RwbHLH62

MENIGDEYKHYWETNMFLQTEEFDSWGLDEAFSGYYDSSSPDGAQSSAASKNIVSERNRRKKLNERLFALRSVVPNISKMDKASIIKDAIDYIQELQEQERRIQAELVELESGKMKKKNS

SVYDLEEEMPGLLRSKKKRTTDQSFDSRGSRASSPVEDLELRVSYMGEKTVVVSVTCCKR

RDTMVKLCEAFESLKLKIITANITAFSGRLLKTVFVQADEEERELLKIKIESAIASLNDP

ESPVSF

>RwbHLH63

MVVKLINEQIKWVLTFDYSSVNPCVYEQSKCVLITLDLLTDKLSIGLEKDRGLYDQSWLI

SLDGFYNEENIRRPSSSSGSLARRLFDEYRQSVVAVDNIDRVPGLAFRDSVPFMELKELD

LQRLASVQPQLQFYQEARIKTAIFMGCMAGEIELGFSSDTTQVNWEMEMRKWMPENFPDQQAPYQDLPPPVSDHQTRASSSSSSLRSLSTDNSLEPSPFLFNIPPNTSYHQEPPKQPPIH

EQAFRPTSNDAITRAILAVLSSPSPSASPSASSPQPSVAQNFPSFSRNATSAFNRYRPGF

AHSSSTPMIARVGRSSMLKRAISFFRSLSSTRSQGRVPVQGGSRPTENQLYHMISERKRR

EKLNESFQALRSLLPPGTKKDKASVLSGTSEYLNSLRSQVVELTRRNQVLETQLLPKTKP

RDDQEGSDESSYRRLDVKVVNVAAAESSSSSGARIVDLLVAVRGGGCDILDLVIRIMEFL

RKDERGNEWDESTFQEAVRKVVADLAK

>RwbHLH64

MFQINFTPFREDEIEQDLVMGNAVVESSNLAAPNDVGKRGKRQQNLPAAQPAGDNGGSGKGNKERRVVHRDIERQRRQGMANLFASMRSLLPLEYIKRKEGCPYQKYWEX

>RwbHLH65

MFSLQHGDELVFQISKIEQDLISARASLDGTHLTSTPPAGKKKRKKSYSNQQEDKYENNN

EKKMIAHRDVERQRRQEMAKLYASLRSLLPAEYVKGKRSVCDHMNESVNYIKQLQENIEQLSMKRDNLKNMSDSIGNNTEATGSSENCFPGRVTVGLCWGGVEILISTSTGSEDEGFPMS

RVLEILVEEGLDVVSCVSTQINKKLLHVIKSEVSDPTCVDLSMLEEKLADVINGN

>RwbHLH66

MSSHASLETTGILTPPKRKRKKSLLYSNHDHQQEDKYGGSSTCTTTGSNGNKEKIKMTAH

RDVERLRRKEMAELYASLRSLLPLEYVKGKRSICDHTNESVNYIKKLQKNINELSIKRDR

LKNMANSTANTEGSTESCSSGRVTASLCWGGVEILINSGSEEVGFPMSRVLEILVEEGLD

VVSCVSTQINKKLLHVIKSEVSDPTCFDLSTLEEKLAEVIIGIGG

>RwbHLH67

MSSFEENDDLWFQIYSNYPNQEKTTQLQDQSKIDVPENFGERQPVPDANDHTCFNKKVIH

REIEKQRRKKMAALFSSLRSLLPPELVKGKRSVGDHINEATKYIKHLEKKIKDSGAKRDQ

LQSLSSSSTTTTTTSCSHGNGSSSNCSANCVTVCCRSMGQVEVILSTGGGFDPAGKLQLS

SFLKLLLEEGLSVVSCVSTKVNERLLHTIQSQVLQV

>RwbHLH68

MFQANFTATPSKEEEIEQDLITGNAILESGNHAPNSFRKRGKRPQDSPAGDNHGSGDQGN

IKERKAVHRDVERQRRREMANHFASMRSLLPLEYIKGKRSASDQMHEAVNYISHLKNNVK

ELEIKRDNLKKLSAGSSTTTATGTGSIRSSANNRHRSSSVTVSQCRGGVEVLIYSCGFEE

EGGLVI

>RwbHLH69

MYLLKKDAKFVWAEHCEQALLKIKKELLLPPNLMVPIPAQKMDNWDQCKAGGSKDTSFAV

PWPPCAESVSSFQCHSYNPWSMRFGGVQEDRSGASASRSHSQAEKRRRDRINAQLATLRE

LIPKSDKMDKAALLGSVVEHIKDLKRKALEVSKTLSVPSDVDEVTIDCFSDVQDGSSTAT

STNNIYIKASVCCDDRPELFSELTQALKGLKLKMVQADVASLGGRIKSIMVLCSKDNKEV

ACLNTVVQSLRGVLSRIACSSSSMGSSYRMKSKRQRFFLHSH

>RwbHLH70

MEGNSGTDSRSADNGVESLQLGEEIQRLVMAPETGNSFTALLELPPNQAMELLHAPGGND

ETPPPFNRLLRSLDNTPTFPSNAELLERAAVFSVFAAENSMETSLIPGKGLTKKSKSKAN

ETSDDGEKLPYVHVRARRGQATDSHSLAERARREKINARMKLLQELVPGCNKGKGLTKKS

KSKANETSDDGEKLPYVHVRARRGQATDSHSLAERARREKINARMKLLQELVPGCNKVEG

DRELNSGIEVQLGFHMIIAGSLACVCPIVIPVHLHLCTEEKIALQISGTAMVLDEIINHV

QSLQRQVEVKIFPILKGGDANELLNSRTRIKLHDHMAISESASTIYLLRFDFPEEYVSD

>RwbHLH71

MAMDQGHILHDPLPLETLIWPNYPPPQTHQISSSTVPTLQLPPTSHVFISNQTVLLENHH

QMEAEVEVLEEQEEPEEELGAMKEMMYKIAAMQPVDIDPATIRRPKRRNVRISDDPQSVA

ARHRRERISEKIRILQRLVPGGTKMDTASMLDEATRYVKFLKRQIRQLQSNHHPLGHVAN

GDWPQNKALSFATTSCSMGPPGLGGYVFGGGSGGVDPT

>RwbHLH72

MDLGTVVFQQDPFSNLNSYGYKEVEGCHWNTNSSSPEVCAGDGLLAGASVEAPARRKRQR

NYKSLKNKVEMENQRMTHIAVERNRRKQMNDYLAVLRSLMPPSYAQRGDQASIVGGAINF

VKELEQLLQSLESKNQLKQHSKSPRHFANFFTFPQYSTRSTHQSNLPLTGHESRMAAEKE

STIADIEVSIVESHANIKVLSRRQQKQLLKMVSWFYSIGLTILHINITSTVDQLVLYSFS

VKVSLLN

>RwbHLH73

MVRSLKSHHEEEEEDEDEEFVSRTTDATSHIAKADGKCTGQKGNAPRSKHSETEQRRRCK

INERHLCLGLISLMSRFQMLKDLLPQNDLKRDKASLLLEVIEYVQFLQEKLQLYEGPYQG

WTQVPSKLMPWKSNSAPVESCMDPSQLMRNGSGQEDNXVVMPAMLSNARNSAESDLSEAD

AYDALNHPPMASNQATPSQIPLQSGVLEDMHTQAHQTSVSYSGQLASQSQSQFWQVRQST

TENAVPSYALGEQEELKVGSAETSFSNAYSQGLLNTLTQXLQSSGVDLSQASISVQLDVG

KRANTGPTTTFGEKDPQNHSPGNQLRATHGLGSSYDDSGQAHKRLRTERI

>RwbHLH74

MGLVQAFTYYGNVFGGSSAQVNSDQSYQPAEGGAQSVINFKTTTAGYNNVMHSNGSSFLS

FDQNPDHQQNSNPNKISDDQQYLDDDYSIWEDNLIQDFSTTRVQSTSSYGADQPFGWHNS

EENPNCGNNFHEEFGKQEEGFNKRPHKNRRERISERLKVLQDLVPNGSKVDLVTMLEKAI

SYVKFLQLQVKVLATDEFWPMQGGKAPDISQVKEAIDAILSSQKDRNSSSE

>RwbHLH75

MEDPIFTDHFDIMEYFDEELAAALGDDFQNSLSSESNSSSSTLLNDIPNSSSTTNLLLAS

STLEALESPNSKHHKPNSWPTNSNNNNDSTAAAILDQQPSVFSSGCILSFGNSGSTENPK

IPPGALNSEDEEVVAAGMLLSNKSSQGAKKSGSSNPRPASQTYDHIIAERKRREQLSQLF

VALSAIVPGLKKMDKTSVLGEAVKYLKQLQDRVKTLEEQSARQTIESVVLVKKSQLLVED

EAAVDENFVSGSSEPLPQIEARVCNRSVLLRIHCEKHKGVLAKIFGEMERLNLAIVNTSV

ARFGALALDITIIAEVLRLHS

>RwbHLH76

MLHMDDSFTGNSTDMTVLQRHQAIFGRQQEDQRQDNNAIESYAMPRLQSLIGDDSMVYQE

LMSRSMNADQYLGSMFPAFRELQLAGTEFVGETISIAPLQLVTSSSPEVNHSFSVGLNLK

KRKADEGESAAIETTNLETSTDNSKVSDVQKPDYIHVRARRGEATDSHSLAERARREKIN

KKMKCLQDLVPGCSKVTGKAGMLDEIINYVQSLQRQIEFLSMKLAALDPRLXFDMDRFSM

EEFPAHVTSFPAAVAPPEMADLAYQFSQVQKGAAAYGIDMPINPMQLAPQRSTTSSSSLS

IPKAPVMDGYSLRMEWLFLDNNHCRFP

>RwbHLH77

MDPPIIHEASFSAANPSSYSLAEIWPFPINNGSGGLGLRMNNLSGFGEATLNRDVSVDES

TVTEQSGGGGRRKQQRREANSEDESSKLVSTSSGNDMNNSNGKRMKTSRSRDENGVSQSE

DEENSGLGNNPAEQRTKSSVPPKQDYIHVRARRGQATDSHSLAERARREKISERMKILQD

LVPGCNKVIGKALVLDEIINYIQSLQRQVEFLSMKLEVVNSRVSSPIEEFPPKELTPTFD

ATGMIFGTQGTRDYVQVSQPEWLHMQIGNCFDRAP

>RwbHLH78

MDSLGGWDSSTVLANTPPLWSHQQHELEEIFMSSSSNYCTHGGGKFDFSTEEIFTPIHHH

LQKPHVNSNSVIVSQMEQQINRVEDGENPIILSECKNLWPDFCGSTRALSSGESGSNEND

KGNYXDTLIGEDITTLDKPNPNKRRNENXSQFDLFQSDSSTTGGGFQLISETNPPKPKKP

RSEKFPNSSNISFQQPNSSXGEPDSEAXAQMKEMIYRAAVFRPVNLGAEIVEKPKRKNVK

VSSDPQTVAARQRRERISERIRVLQRLVPGGSKMDTASMLDEAANYLKFLRSQVKALETL

GQKLDLVNCSTPNTLLPLSSLVPFNHSFPMQINSFSTTPNYS

>RibHLH01

MSLETLSSNEILNFLMYDTVFASKFDCNDSSDTTMLPETSLKPPQFGGASVISSSMMQRC

PPVGPEAVGKRRNPAMQGRKKRRRRQNVCKNKEEAETQRMTHIAVERNRRKLMNEHLAVL

RSLMPESYVRRGDQASIVGGAIEYVKELEHLLQSLEAQKLLLLKGGTPQHDTATAATAEF

FPPPFSRFIVFPQYTLSQIPNKNSSKSKTEVADIEVTLIETHANLRILSRKSLRQVSKIV

AGFQTLYLTILHLNVTTLDPLVLFTISAKVEEGCQLKSANDIAGAVHHMLGIIEESSNLP

SGDQLFKLPQIPL

>RibHLH02

MPLSEFYKMARGSLESAQQRTTTTSSADLSHLPGNELVELVWDNGQIVMQGQSTRARKTS

SSIDFQSQTPKIRDKDRLNATHSKMGKFGVMDCVLNDFASAVPSGEMGLDQDVDMVPWLN

YPLDESIPHDYCSNILPELTGVTVNQISSHSSFATNKRGSYNRTTRGSNYVQNGLGLEHE

NAAKVSSSEVGEGSGSRISNFFPWSFQENQTSLPSLKSGVSSIISNNITNTKHAVCGDPS

TGVKIQKQDSVLPNTNSGIMNFSHFSRPAALVHANLQNVSAIASPVTLGIEGMGGKNKGP

SLSSTNFAELSKGMDSNSQPNSVPPKVDYVVVGKPIEELRAAEQSEAVCQEDVVKNDKLL

SQVVGEKTVEPVVASSSLCSGNSAERASNDLTHNLKRKCRETDDSEGRSEDIEEESVGIR

KAAPVRRGTGSKRSRAAEVHNLSERRRRDRINEKMRALQELIPNCNKVDKASMLDEAIEY

LKTLQLQVQMMSMGAGMYMPPMMFPAGMAQLHAAHMVRFSPMGMGFGMGMMDMNGGSPGC

PMIQVPPLHGAHFPAPRPAPIFAPTSFQGMAGPNFQVFGHPAPRVPFSGVPPINSAIELN

ATGMAAPVELPNSAPSSNPKDLIQNANSQMMHNANAKASTSMNHTSTQCQVSNAGFGQSS

LLHRSDQAPDVVCRDDMNLTKEADVLLRPGCE

>RibHLH03

MEDVGFMRQWPTNSLDEIVNHHFSRQIFDVKPNISEYSSHDTGIIDPPGKILITNTWNSS

SNSSSPHQISFSNSNCETQFGTIKPKEEAVLSPSSNITTLPSGVFDFQESFGNQIYGFKA

RQASKKNSTNGTRFSSSQEHIMAERKRREKLSQRLIALSAMVPGLKKMDKASVLGDAINY

VKQLQEQVKTLEEQTTRTKSMETVVYVRKCELINSDHNLDNFSGDGGGGGSQYDETLPEI

EARFCDKNVLIRIHCEKRKGVVEKTLAEIEKLHLSVVNSSVITFGISVLDITINAQMDED

FSLTTKDLVKNLHAALKLFM

>RibHLH04

MRTFVWESQSWAFANSDHSGSDGKPPDLGSNTQTPTGKEVKAVQAATGGKKRSGGGKKKG

KGSGGGGDGGEGNEGKSGGESDHEIHIWTERERRKKMRNMFSNLHALLPQLPPKADKSTI

VDEAVNYIRTLQQTLQKLQRKKLERLHGVAPINYEPSVVTPQKLAIDSREAFVADQVSSS

NLANNSTPANSNSSNSVLPFVSRFPPIFQTWTSQNVILNVCGEQAQINICAPKKPGLFTA

ICFVLEKHKIDVVSAHVSSDQNWTMFMIQAHARIAHDQLPEAFPVEEIYKQAAGEIMLWL

SS

>RibHLH05

MNRALPEMLHCINTPGNVAGSFTDMSVLERQRARIKWQQEQLLNQPPCYFEGNDQFNGYS

MPNHAQDFHNLISSEVGLVVKADPGLDTGWQEFGQFGGEGSGLGSSGFVNGSGFELNYGI

SRTLSCPQEVAAAVTETARRESVSPEKMSSAVGRESFKKRKADRNQSLKAVAEEEPNEKR

IKECNEEDESKITEENSNNSKNTTNNDNNIKRETSADTSKVSEVQKPDYIHVRARRGQAT

DSHSLAERVRREKISERMKYLQDLVPGCNKITGKAGMLDEIINYVQSLQRQVEFLSMKLA

AVNPSLDFNIDSFFAKEVFPASTSNFAIGVQSEITNPAYLQFNPLQQLASCTGLEMGINT

PDMALRRTTSTPISLPETFLNSSCFDQIQTSTAWDGNLQNIYSMEFLQGRSTSFSAQQFT

GSNEASNLKMEM

>RibHLH06

MADLYNANLHSSPLESEEMTSYLHNLLHKHPFSSPPQPPPAATGLVGRSSVFADSDGRFG

ERDSVMVDSSAGINFSDPGRGFMADARAVYGGDSDGITSMFKRKFSEESDFDEFGSDFTG

PEASEAPVNPALPRSSKRTRAAEVHNLSEKRRRSRINEKMKALQKLIPNSNKTDKASMLD

EVIEYLKQLQLQVQMLSMRNGLSLHPLCFPESLHSALMPQPVLSFDEGNEVLNSNRGQDT

FSRNQEISVQAAYDFPNQAPMTNINNSETSYGLEPSMQSHYGPFRHHSKELCREDGLSQL

QLDMSCSVKNSSSGVSS

>RibHLH07

MGTEENGNMGFQQRNGDSILNCPTSGMNTTNPFFGSGWDPLVSLSQSENFGGSFGNSNYS

HLVHYPSDSGLVEMVPKLPFFGGGGNFSEMVGSFGLPNYAPNEEDGTEKTSTNGGDLQVS

EEGTMGISPCGKRKKRVSESNSPFNPSKNSEGEQQMDISGDSSQEEKKQKSEQNPSANSR

GKQTGKQAKDKSDSGEAPKENYIHVRAKRGQATNSHSLAERVRRERISERMRLLQELVPG

CNKITGKAVMLDEIINYVQSLQQQVEFLSMKLATVNPELNIDIDRILSKEILHSGASNAA

IPGFGLGMSSSHPYPPGIPQGVLSGIPSTSPYHSLPQNLWEHELQSLLQMGYDSNPAIGN

LGPNGRSKLEL

>RibHLH08

MAAERNQGMLPSLAAPPPSFFSPFPEPLPRSFSPQPPFPAIDISAGVDLFSPLIFVAALS

SPTLEVYHIPLLHTDALVHPQEPKAAAEVERNYSERACSSTMHQGKAKTAPCIWVARGRL

CPDGGALRGRAQLDRDIRPSTSSTPLYQFNHSKGLDDGGMEDGGFMNHWPINCLNEILNH

PVSPQIVDVKPNSMEYSSHARKTWNSSGNSSSTHQISFSNPNYETQSATIKPKEKAVPAG

NITTFPSGVLNFQGSLGNPISGFKACQGSKRNSTNSKRVSSSQEHVLSERKRREKISQRI

IALSAMIPGLKKMDKASVIGDAIDYMKQLQEQVKTLEEQASKTISMESVVYLRKCELHSD

HWDNLSLENSNYTGGGGNSGAHYEEPLPEIGARSCDKNVLIRIHCVKRKGVVEKTVAEIE

KLHLSVVSSNVMAFGSYALDMTILAQMDDEFSLTIIDLAKNLHAALKLFT

>RibHLH09

MLPHQNFSYGLQSIWNEESLVQKQDSMKRMGMDRDSISRAQKHKSEAAAKKHAKAEQLRR

KRINTHIQTLKKLLLPNMTSKKVKASILTETIRQLKELHKRAADVALQQYGHTDISGCFT

FPDGSDEVTVSRCEGGEGKTVKATVCCEDRPGLNQDLTDAIRSAGGKPVRVEMATVGGRT

KAVVVVRWVEGGGEEEEVGLLTRALKALVESGPLGFSRLGQIGRVNGRTGLGLEMSRNKR

ARMCSSFEGEREWEKLLLSTV

>RibHLH10

MALETVEYPQDPFGFLGIFDTIMDHGNNIIGANWDSSSSSVRQNVMECWECPNSSPEACT

GDRFFGGEGPPVEAPSTEAVRRQKRRRTKSSKNKEELESQRMTHIVVERNRRKQMNEYLA

AVRSLMPASYVQRGDQASIIGGAINFVKELEHLLQTLEAHKRTTPQQQQQQHPPNNNGQS

STSLFADFFAFPQYSTSSKNNCTDTSSPAAADKPQPVAFGDIEVTMVESHASLKVLSKRR

PRQLLKLVAGLQCLRLSVLHLSVTSADQMVLYSLSVKVEDGCRLTTVDEIADAVNQLLGR

VEEEAAFV

>RibHLH11

MEFSSGFSWNPVFQEIIRRGSSSSTSSLVLDGERGELVQAMVRPSHKGVKAEKALEALRN

HSEAERRRRERINGHLTTLRNLIPGTDKMEKASLLAEVVNHLKELRRNVTEATDGMLVPT

DIDEVKVEQQEDGSDGASCAIRASLCCDHKYEILSDLRQALDALHLQTVRAEIATFGGRM

IYVFIISCCKEKKMEDIEGCRLLASSIRHTMRSVLDKFYASEEISSRNTLSNKRRRVPFF

ESSNSSSIGDLW

>RibHLH12

MRRVYRRVEALVEEVPTKQVPSRSSLKRGRAAEFHNLSEKVVYMCYEDIGGGAGSMKKMK

ALQNLIPNSNKTDKASMLDDAIEYLKQLQLQVQLDG

>RibHLH13

MADIFPLQAQPELAMDLLGFQGNFSFYLEYAFVDQPLTVPNDLSLPYFAPPPEIYEDDYN

YPSKTSCLANGFFLNPRPLVPHFSPGLELPLSAQSVAARNRRRKITEKTRELGKIVPSGE

KMNTVEMFQAASKYIKYLQDQLGILQFLASSIQVITLC

>RibHLH14

MASGSCHSSSTPKIERRVIEKNRRNHMKSLYANLYSLLPSHASKEAVPLPDQIDEAVKYI

ESLEMKLKKSKEKKETLSGRKRSHSCTSEFKSPQLEIHEMGSTLDVVLITGEDDKFIFHE

IIRLIHEEGAEVLNANFSVFGNSIFHAIHAKAGEFEKNVVAPRVSSRLKEFVHGSMSDVE

SVLPAESWEFDIEPDAWELKVPQVLPMRLY

>RibHLH15

MYGNSMEIDSTTENSIWLFDYGLMEDISAIPDGDLPAAPLATACFSWPSQEINSCSNVSV

DIDSSFEDSEAVEETGLRKRLKSESCNASGTKACREKSRRDRLNERFGELGSVLEPGRPP

KTDKAAILGDAVRMVRQLRSEAQKLRESNEDLQEKIKELKAEKNELRIEKQTMKEEKEKL

EHQVKALTSHPGFLPHPFAMPVAYGAHGQTTDHKLMPFITFPSAAMWQFIPPAVRDTSHD

HVLHPPVA

>RibHLH16

MYGNSMEIDSTTENSIWLFDYGLMEDISAIPDGDLPAASGDRLLQLAVAGDQHVDIDSSF

GDSEAVEETGLRKRLKSESCNASGTKACREKSRRDRLNERFVELGSVLEPGRPPKTDKAA

ILGDAVRMVRQLRSEAQRLRESNEDLQEKIKELKAEKNELRIEKQTMKEEKEEKLEHQVK

ALTSHPGFLPHPFAMPVAYGAHGQTTDHKLMPFITFPSAAMWQFIPPAVRDTSHDHVLHP

PVA

>RibHLH17

MVSPENTNWLFDYGLMEDIAVPASGFSWPVQPLDGSSNVGVEIDSFGESDSLKETGSKKR

TRPESCGTSSSKACREKLRRDKLNDKFIELGSILEPGRPPKTDKSAILVDAVRMVTQLRG

EAQKLKDSNSNLQEKIKELKAEKNELRDEKQRLKAEKDKLEQQLKTMPTQPGFLPPHPGI

PAAFAAQGGKNFMPVMGYPGVAMWQFMPPAAVDTSQDHVLRPPVA

>RibHLH18

MDKRQRPEVEVKDPNAARKVQKADREKLRRDRLNEHFIELGNALDPDRPKNDKATIINDT

IQVLKDLTTEVNRLKAECAALSEESRELTQEKNELREEKATLKSDVDNLNLQYQQRMRVM

FPWGTIDPSAVMAPPYSYPVPIPVPTGPIPMHPVLQPFPFFQNQNPGAIPTPCSTLIPYP

GPPNPPMDQPPYQCASTSRVSSKQDSKSKTSDHCTGSIDDKSDDSNDVVTELELKMPGSK

AQQEHSPGGTAGKQATRKEKSIADGNSSSSISDGIEHRED

>RibHLH19

MALETLDFQQDPFNYGHKDLYTKGASYGYWDSLLSNADSFLPDVDLTGNDGVATGICPSP

VGAPVAVQGRRKRRRTKSLKNKEEMESQRMTHIAVERNRRKLMNNYLAVLRSLMPPSYAQ

RGDQASIVGGAINFVKELEQLVQFLESHKQANKKPYDSPPFANFFSFPQYSTRLTGHNGS

VTGQDQYSTAEKGSTVADVEVALIESHANVKILSRKQPKQLLKMVAGFHSLGLPILHLNV

TTVDQMVLHSLSVKVGDECQLNTVNEIATAIHEMMGKIQEEAISS

>RibHLH20

MEFTQQGLLDELLAAPRRDIWANFPSNDDFSNGWIFESLEHESQVLSPPNPQFLGFTSPT

EPSFSFPLHGLHYPFADTFTVSEIDSSYDSNAPPPIPFPTQEEHPSMVKNDGEYGFVGSA

LYTLEDRLNGCKVEMEPTANTPEFNMDFGRQRKNRAKKVEGQPSKNLMAERRRRKRLNDR

LLMLRSIVPKISKTDRTSILGDTIDYMKELLEKVKNLRGEEDAKGSMNQVQLMGKSKELK

PNELLVRNSPKFNVERTNSETRVKISCAPKPGLLLSTVSTIEALGLDIQQCVISCFNDFS

VQASCSEGMQQQAIISSEDIKQALFRNAGYGGRYL

>RibHLH21

MALANSNGYTATSSQREESHSVISFKTGYSNSIHNSGSLLIFDQNDQIPQNPHPQQHGYT

IWEDDYNPIQNYQNQLNPQRTASNSRLLQDIDSVDQCGNAFGWPDSDEANAIPTSGTNRE

FGTQEACSNKRPHTGESMQPLKKQCSSTDTATRKPKPKPTDHQSKDPQSIAAKNRRERIT

ERLKVLQDLVPNGSKVDLVTMLEKAISYVKFLQLQVKVLATDEFWPVRGGKAPDISQVKE

AIDSILSSHKDRNSSSD

>RibHLH22

MALANSNGYTATSSQREESHSVINFKTGYNNSIHNSGSLLIFDQNDQTPQNPHPQQHGYT

IWEDDYDPIQNYQNQLNPQRTPSNSRLLQDINSVDQCGNAFGWPDSDEANAIPTSGTNRE

FGTQEACSNKRPHTGESMQPLKKQCSSTDTATRKPKPKPTHQSKDPQSIAAKNRRERITE

RLKVLQELVPNGSKVDLVTMLEKAISYVKFLQLQVKVLATDEFWPVRGGKAPDISQVKEA

IDSILSSHKDRNSSSD

>RibHLH23

MNNQDEFRNYQSNHNHSNQSNSNSGLLRFRSAPSSLLSNFNSVGTDYGEKSSSPENVMHD

LEESKPFANGYCFSSSQLPPQYPRQNNTGSSYGGVVGSNHLMRQSSSPAGIFSPQNGYAS

LRSFGNYRVGNGTNDGDVSPSSSRLRSPMNFPPGVPSSLGMLSQISELEDESVVRSDMKL

GNGSREPQFFPFGSWSESSHFPENYSLMKRDLDYDGKLFAGTQNGELEHRAHNLSHHLSL

TKTAAEMERLLQFQDTVPCKVRAKRGCATHPRSIAERVNLSLFQNCYSLIEKDTFVLKIL

WKVKKEWIDMITIWILILQVRRTRISERMRKLQDLVPNMDKQTNTADMLDLAVDYIKDLQ

KQYKILNEYRANCKCSSIQKAFANLSA

>RibHLH24

MEGNSGTDSRSADNGVESLQLGEEIQRLVIAPETGNSFTALLELPPNQAMELLHAPGGND

ETPPPFNRLLRSLDNTPTFPSNAELLERAAVFSVFAAENSMETSLIPVNSVANSRERVKR

EPMDTDSNPNSSDPNSNQKSTKRKEREKKGKGLTKKSKSKANETSDDGEKLPYVHVRARR

GQATDSHSLAERARREKINARMKLLQELVPGCNKISGTAMVLDEIINHVQSLQRQVEFLS

MRLAAVNPRIDFNLDSLLAAESESPVDGNFPGTVMPLMWPDSQVNGNRPPYQQIWHFDGL

HQPVWERESHNFINADNSLLSYDSPTNSASLISNQLKMEL

>RibHLH25

MDNWDQCKAGGSKDTSFAVPWPPCAESVSSFQCHSYNPWSMRFGGVEEDRSGASASRSHS

QAEKRRRDRINAQLATLRELIPKSDKMDKAALLGSVVEHIKDLKRKALEVSKTLSVPSDV

DEVTIDCFSDVQDGSSTATSTNNIYIKASVCCDDRPELFSELTQALKGLKLKMVQADVAS

LGGRIKSIMVLCSKDNKEVACLNTVVQSLRGVLSRIACSSSSMGSSYRMKSKRQRFFLHS

H

>RibHLH26

MFQANFTTPSKEEEIEQDLITGNAILESGNHAPNCFRKRGKRPQDSPAGNNHGSGEGNIK

ERKAVHRDVERQRRREMANHFASMRSLLPLEYIKGKRSASDQMHEAVNYISHLKNNVKEL

EIKRDNLKKLSAGSSTTTATGTGSIRSSADNRHRSSSVTVSQCRGGVEVLIDSCGFEEEG

GLPLSKVFGVLTERGLNVVSYVSSEVNGRLFHTIRSEVLHPSQFNSLSFYSNWIKN

>RibHLH27

MFSLQQGDELVFQISSIEQDLMSSHASLETTGILTPPKRKRKKSLLYSNHDHQQEDKYGG

SSTCTTTGSNGNKEKIKMTAHRDVERLRRKEMAELYASLRSLLPLEYVKGKRSICDHTNE

SVNYIKKLQKNINELSIKRDRLKNMANSTANTEGSTESCLSGRVTARLCWGGVEILINSG

SEEVGFPMSRVLEILVEEGLDVVSCVSTQMERKLLHVIKSEVSDPTCFDLSTLEEKLAEV

IIGIGG

>RibHLH28

MFQINFAPFREDEIEQDLVMGNAVVESSNLAAPNNFGKRGKRQQNLPAGDNDGSGKGNKE

RRVVHRDIERQRRQGMANLFASMRSLLPLEYIKGRRSASDQMHEAVNYINHLKNNVEELE

IKRDNLKKLSDSGEWIKKLHQ

>RibHLH29

MFSLQHGDELVFQISKIEQDLISARASLDGTHLISTPPAGKKKRKKSYSNQQEDKYENNN

EKKMIAHRDVERQRRQEMAKLYASLRSLLPAEYVKGKRSVCDHMNESVNYIKQLQENIEQ

LSMKRDNLKNMSDSVGNNTEATGSSENCFPGRVTVGLCWGGVEILISTSTGSEDEGFPMS

RVLEILVEEGLDVVSCVSTQINKKLLHVIKSEVSDPTCVDLSMLEEKLADVINGN

>RibHLH30

MSSFEENDDLWFQIYSNYPDQEKTTQLQDQSKIDVPENFGVRQPVPDANDHTCFNKKVIH

REIEKQRRKKMAALFSSLRSLLPPELVKGKRSVGDHINEATKYIKHLEKKIKDSGAKRDQ

LQSLSSSSTTTTTTSCSHGNGSSSNCSANCVTVCCRSMGQVEVILSTGGDFDPAGKLQLS

SFLKLLLEEGLSVVSCVSTKVNERLLHTIQSQVLQV

>RibHLH31

MFMDYIFLLDEGARPTFLRNLAHSSGCTYICLWRSYLPQRSNSWLFSLDGFYNEENIRQP

SSSSGSLARRLFDEYRQSVVAVDNIDRVPGLAFRDSVPFMELKELDLQRLASVQPQLQFY

QEARIKTAIFMGCLAGEIELGFSSDTTQVNWEMEMRKWMPENFPDQQAPYQDLPPPVPDH

QTRASSSSSSLRSLSTDNSLEPSPFLFNILPNTSYHQEPPKQPPIHEQAFRPTSSSVSLS

PLHQAIQSFNQTRNFPFPTPETEDDAITRAILAVLSSPSPSASPSTSSPQPSVAQNFPSF

SRNATSAFNRYRPGFAHSSSTPMIARVRSSSMLKRAISFFRSLSSTRSQGRVPVQGGSRP

TENQLYHMISERKRREKLNESFQALRSLLPPGTKKDKASVLSGTSEYLNSLRSQVVELTR

RNQVLEAQLLPKTKPRDDQEGSDESSYRRLDVKVENVVAAESSSSSGARIVDLFMAVRGG

GCDILDLVIRIMEFLRKDERVGFISVEADTKVEESISVNRVVSRLKIEGNEWDESTFQEA

VRKVVADLAK

>RibHLH32

MQPCSREMQALKSVLNQSQMSLQTIQQHHNQQQQIDTSNNQIQHFDPATPSHDDFLDQML

STLPSCSWPPDLAGPNPKSPWDLSASTPDDQFHFDHDQSAILAAKLRQHQINGGGGSSPA

AKSLLLQQQLLFQSRGGISGGESGLMPLSLGGSGDDVADGSSFKSPHSGDDGPVQALYNG

FSTGQHFHHVQGGAMQAQNYGVAAAVGVVNQTPAGGGGVGQPRQRVRARRGQATDPHSIA

ERLRRERIAERMKALQELVPNANKTDKASMLDEIIDYVKFLQLQVKVLSMSRLGGAAAVA

PLVADMSSEGGGDCTALTAGGRTTNGTQAASASTNNETTMTVTEHQVAKLMEEDMGSAMQ

YLQGKGLCLMPISLANAISTATCHPRNPMIPNNHHPLLGSNGVGGNDGFGGPSSPSISAL

TVSSAAMGNGGGADGSVKDAASVSKP

>RibHLH33

MENIGDEYKHYWETNMFLQTEEFDSWGLDEAFSGYYDSSSPDGAQSSAASKNIVSERNRR

KKLNERLFALRSVVPNISKMDKASIIKDAIDYIQELQEQERRIQAEIVELESGKMKKKNS

SVYDLEEEMPGLLRSKKKRTTDQSFDSRGSRASSPVEDLELRVSYMGEKTVVVSVTCCKR

RDTMVKLCEAFESLKLKIITANITAFSGRLLKTVFVQADEEERELLKIKIESAIASLNDP

ESPVSF

>RibHLH34

MAAFSNPNHPFLIHPIFLPNNSAFSQEPIAITPTPCFPQFYPPESHNTEPYVTNKINSAD

SSSVDDHKAESGEQVTQKAVSMAKKRSHSDGSSLSSVQSMEAREVKGKKQKKSDGTRKDE

NEKKLNKAGKKDQKIKAAEEAPTDYVHVRARRGQATDSHSLAERVRREKISERMKMLQAL

VPGCDKMTGKALILDEIINYVQSLQNQVEFLSMKLGSANSMFSVDSGVEFDASLLTPERL

SSMESPLSNGQPCNNSSQPIGFPHSPTFPPQDSTYPLLDNISSLLFDQQLQFPHTLHPQD

NGQLVWDVDEQRQKLVNQSGLISHNNFNLSSFH

>RibHLH35

MMSECMVPNWKERRQRQEPVVVVVGGGEGNRSSHVQYSQSQIQPHHQHHYLNSTVPIMPN

YEVAELTWQNGQLAMNGLSSGGLLPTGPTKPTWNRAGDTLESVVHQATWPNQNLYPPLPK

RDHNPANVSSAVGSTIVKRAETLGRMHVGPKRVRSETDQCGGSFGSSIQEERSACAAAAS

ATFCKENDATMVTWASFESPRNLKTKTTDEDSAGDLDGSENLEDQERETTKGETGRSHST

RRSRAAAVHNQSERKRRDRINQKMRALQKLVPNASKTDKASMLDEVIEYLKQLQAQVHLM

SSATRNNMPQMMTPAALGLHHQQQIQMSLLARMGMGMGVGFGMGMGPTFLPPPHPFVVPP

MIPNHNLSQATTDAAAVRSSVPFNNPYCTFLGQPINMDLYNKMAAIYQQQVNHTSLQTSS

PFPSTHVQGGKEHLT

>RibHLH36

MDPNEGAFQTARYNYAEIWPFPVNGGEAGGGLDLRRLQFGQSLGLFAEDVNVNREVENDP

TVPDHGRARNGGSKKRRDPNPEEESAKAVSTGGNAMDDCDGKRTKMGGSSDENLDSKAEA

EANSSNPTEQNTKPPEPPKQDFIHVRARRGQATDSHSLAERARREKISERMKILQDLVPG

CNKVIGKALVLDEIINYIQSLQRQVEFLSMKLEAVNSRMEPGPERFPTKDFGQQTFDTAG

AAFGSQATREYERSSSPEWLHMQIGGSFERTT

>RibHLH37

MGVLDRALEWLRPFVATKIWDYSVVWKLGDDPSRYIEWVGCCCGGAYGDSNGIKIKEERP

EGKPQLLIPQCRDTHIQHPANTKACLALSHFPPSIPLYSGVHGEAVLSTEPKWLSCTEDS

DSNQSHKSNGTQVLIPVVGGLIELFSTKHLPRDQKIIAFIVSQYNNTMEQDSITTKSCSN

MRFNAQSHDTLPGAHYPVNNWPALFEAKKIFPRLPFLPPVSQLNHRPTLEGSSTGSNPSN

EHSISFNSGSVRVSPNVSVNRTFGEYPTDDKSNKCRNLSPKQDHLVETDKLKSKQRMEKE

QYHSKNLVTERNRRRRIKDGLFALRALVPKISKMDRAAIVGDAIEYIEELHKNVKELKAE

LAEMEEVEHKKKNDELVIPKLNAKKGTKTSTTTADEKGKMEVQVEVHQIGTRDFFVKLLC

TQKTGGFARLMEAMHSIGLQVVDANVTTHNGKVLNILKVEVNKMEVVQPKRLKDSLTQLI

RKTLQQ

>RibHLH38

MMCGKQEEEQGDYSQNIHTQQNYQEQFLVQQQQMQQQQQQQHNTTGDAYIGDLYPFLPWT

LPPVHSFNPAREPDPFLLPPQPYGGLLFNNRRSSQPPLQFSYDGLISESLGHVVQHPGSV

PFGLQAELGKMTAQEIMDAKALAASKSHSEAERRRRERINNHLAKLRSLLPSTTKTDKAS

LLAEVIQHVKELKRQTSLIAETSPVPTECDELTVDTSDEDGRFVIKASLCCEDRSDLLPD

LIKTLKALRLRTLKAEITTLGGRVRNVLFITGEEEEESNSGDNHQQQYSISEIQEALKAV

MEKTNGDDSPSGSVKRQRTNVNIIEHRSL

>RibHLH39

MKSAKVHLEEEEEEEETPPSNSKDGKSNDKASATRSKHSVTEQRRRSKINERFQILRDLI

PHSDQKRDTASFLLEVIDYVHYMQEKVQKYEGSYQGWVSEPSKLMPWRNSHWRVQSFVGH

PQPITNGSSPGSAFTGRLDENNCTISPTIHPNQQHSIESDSSGDPSGKMVDLQPELTNKS

MATPMPLQATIPSSIQCDGVLSHSLQRPISEAQSTEFPSRNDGSNHEEDLMVEGGTINIR

SAYSQGLLNNLTQALQSAGVDLSQATISVQIDLGKRANTGPTPGISIPKDPQNSPSHPSM

GRFEDAGNGEDLGQAQKRLKK

>RibHLH40

MPMSLFLAMIFSYIIKQVGFWLLHLAAAAPLPVGVPSRSSLKRDRAAEFHNLSEKVVYMC

YEDIGGGAGSMKKMKALQNLIPNSNKTDKASMLDDAIEYLKQLQLQVQLDG

>RibHLH41

MQHSTTSSGGGGGGNRGGEAHRGLARFRSAPASWLDALLESEEDEDDNDPLLNPTTQFQT

QLKPPTAQFPPTSYADPASFDAGGAPAFLRHNSTPADFLAQISAGPDGYFSSFGIPSGYD

YTASSVDVSPSSKRHKEADTSAKFLPQLKAEPSGMSGLLDVEMEKLLEDSVPCRVRAKRG

CATHPRSIAERVRRTRISDRIRKLQELVPNMDKQTNTADMLEEAVEYVKILQKKIQELKE

LQKNCTCADTE

>RibHLH42

MDTNRQVPQYSHDTNQQQRQVPSSSGLMRYRSAPSSYFADLINSSGLRNDVVENCDEFLD

PRQTSPETERILSRLMSGGGGTGQNASVNEALQSQFPTESAKHGGAEIFGHSDQHRQQQQ

NSNYSSGSNIMYHNSVASNSGIESTYRMESLPQMKIGGGGGNSNLIRHSSSPAGLFSNIN

IENGYGVMRGMRNFGGGNGTNAEASFPSPSRLDGKSQIDFSSAPSSSLGRMTPILENRSK

GMEITGFPIGSWDDSAIISDSFLKGIEEDEDRKTFSAMNNASENQNAEGGMRPPHVLSHH

LSLPTSSAELSAMENLLQFQDSVPCRIRAKRGFATHPRSIAERVRRTRISERMRKLQELV

PNMDKQTNTADMLDLAVDYIKDLQSQVKILSDTQAKCKCSNKQRP

>RibHLH43

MTSYSWNSNVSNFGSNCLEMLEPFSGNLGDLGGNWGGGSDSVSVAQSLVLDGERGELVKA

PIGVGKKSVGLPEEKVVAALKSHSEAERRRRERINAHLNTLRGLVPCTEKMDKAALLAEV

ITQVKQLQKTARESATGLIIPMDDDEVRVEPHHDEDGSFSLRVSLCCDYRPELMSELRQA

LDSLHLNTVKAEISTLGERIKNVFVFTSRKDIDSENAESCQLLVNSVHQALSGILDKASA

SAEYSPRTTLPTKRRRTSYVDSSSSSS

>RibHLH44

MVNPEENSNWLVDYGIIENIPVPGGDLPSLEPGFDWCPDALAGSTGLSVDFEDSFGNLDG

IKECGSRKRMRPESCSASDSKACREKMRRDRLNERFLELSSILEPGRKPKVDKAVILSDA

VQTLTQLRDEAQKLKESSDSLQEKINELKAEKNELRDEKQKLKVEKEKLEQQLKAMSSQP

AFFPYPSGIPTPFGAPGQVVGSKLVPFMGYPGVPMWQFMPPTAVDTSEDHALRPPVA

>RibHLH45

MERDTSGNQLGHIMGDFNPLLGLMDETNFLQFIDIIRGESSDPIAKFCPNFDCEHIGGCL

VDNQFKSSTRLNPFDHFHTDQSVSNPDSDLIINTVLQLATDADEEENDNLEESSTTTTTT

PPTKRKGGDRSRTLVSERRRRGRMKEKLYALRALVPNITKMDKASIVGDAMLYLQDLQTQ

AKKLRSEIAGLESSLGGREKNQRGIVGNQKNTQYTNTSHPICKNIFQLDVFQVEERGFYA

RVECNRGRGVAPSLYRAIESLTRLNVPSSNLGTRAERFILTFSLKVKECEPDTNLQKLKL

GLTEALLKQGFVFQTQASA

>RibHLH46

MDNRYFLSSGIIPPPFYSEQSPMPMWQSVSNLNSPPRLHLPIVEHLVKENMPNLGFPMAL

NHNLPTISGDPGFTERAARFSCFGSRSFNERTSQLLGSTESPYRSSSALMEREKMPRVSS

SGSFKGAGSPMSARENWNSTQTHTGMRCAGDFDMNLSQVPMSDRAEFPSSNEGSSVSGQI

PNGETGSKGPCDSSSKKRKAASRGKSKVVEAEDDGNTKRCKSTEGNGIENDNVKTENKTD

EEKGSPKPAEPPKDYIHVRARRGQATDSHSLAERVRREKIGERMKLLQDLVPGCNKVTGK

ALMLDEIINYVQSLQRQVEFLSMKLASVNPRLDSTNMDSIFPKDVSQPNGSFPHPVAVTQ

SSVHPLDTTYCQNIGTLVDELGQGLPQFPTLCEDDLQSIVQMGLGRSPNRDMGFYSQFFP

GSSQKSQMKSEL

>RibHLH47

MNYLCVPDFDMDEEYSIPTPPPTSTCRPKKLPVVDPDEIMELLWENGQIVMQSQNQRSFP

KRPLIEDGAVISLDRREIRSSRPIESGLSNDQLFMQEDETPFWLHHYPPIEAADLYSDVI

DPAPPRTAPNQDVRIPPPSTAAPMSQPRRSDAEESEAVQSSVRFSRAKARAVEQGPSGSN

EAVRESTVVDSCETPRAAPESVASRVAGSTVEVSGGVAGLCVAAEGGGRDTGTYELTSSS

GGSASLEPVQKPPVTAGDRKRKGREADDDDSESPGEDVEFESPDAKKQIRGSTPAKKSRA

AEVHNLSERRRRDRINEKMRALQELIPRCNKSDKASMLDEAIEYLKSLQLQVQMMSMGCS

MVPMMFPGVQQYVPPMGMAMGMGMGMSRPMLPFPSVLAGSALPTPAAAALMGPKFPVPAF

HMPSVPLPSPSNQSDPVLKSLSPQNHNQPRMMNFPGPYHQYLGLHHTQLPMPQNQAMMQP

STTKPSTSREVENH

>RibHLH48

MAAPPSTRLQSMLQTAVQSVQWTYSLFWQLCPQQGILVWGDGYYNGAIKTRKTVQPMEVS

AEEASLQRSQQLRELYDSLSAGESNQQTRRPCASLSPEDLTESEWFYLMCVSFSFPPGVG

LPGKAYAKRQHVWLTGANEVDSKVFSRAILAKSARIQTVLCIPLLDGVVEFGTTERVQED

VGLVQQPSPNTPLPNPATSSDHPRFHSPPIPTTVYAPVNPPVNPNQIDEEEEEEDEEDHD

EEEEEEEEDEEGESESEADTGRNQNPGVVNVAHAAAAEPSELMQLEMSEDIRLGSPDDGS

NNLDSAEFQLLAASQGGNTVDHQRRADSYRAESTRRWPLLQDPMSSSLQPPPSGPALEEL

TQEDTHYSQTVSTILQHQSSRWSDSSSSSSAAASGYLMYSSQSSFSKWNPRPSDHHYDAA

IPTDGTSQWLLKYILFTVPFLHTKHHNDNNSPKSASAADSASRFRKGTTPQDELSANHVL

AERRRREKLNERFIILRSLVPFVTKMDKASILGDTIEYVKQLRKKIQDLESKARQMELDQ

RDQSQRSRSSGDLQRSSSLSLKEQRSGITTVVNTDRARVGGGPTGSDKRKLRIVEGTGGA

KVKAVDSSPVPVLSPPPPPPPPPPPQPVAGVVVQVQVSIIESDALVELQCPHKEGILLDV

MVVLRDHRVEVTAVQSSLTNGIFVAELRAKVKDNGSGKKPSIVEVKRAIHQIIPPY

>RibHLH49

MDLGTVVFQQDPFSNLNSYGYKEVEGCHWNTNSSSPEVCAGDGLLAGASVEAPARRKRQR

NIYKSLKNKVEMENQRMTHIAVERNRRKQMKDYLAVLRSLMPPSYAQRGDQASIVGGAIN

FVKELEQLLQSLESKNQLKQHSKSPRHFANFFTFPQYSTRSTHQSNLPLTDHESRMAAEK

ESTIADIEVSIVESHANIKVLSRRQQKQLLKMVSWFYSIGLTILHINITSTVDQLVLYSF

SVKVEDYCQLSTVNEIATAVHEMMTKIQEEVMPSYN

>RibHLH50

MYSIPSYYELGSCSSSGNFLQGVVLSSSRTTTTNMGNSSCSTAEKKAEAASKNHSEAERR

RRKRINTHLATLRTLLPKTIKTDKASLLAEVVRRLRELKKTTAEFAANDTNSETSQSNLF

PTECDELNLCHSETEPGTIKATLCCEDRPELISEITAAVKAAEGKVVRAEMATVGGRTKS

ILWVQFVSPTGCGGGGGGEGRLRRGLKGVVNRAAALSSTGPGLQALPENKRARLSQY

>RibHLH51

MQPSGNAMTWTERDEEEQEDAVSWTKNSNNHQSDSKDNSNMNPSLSTFKSILETGWYTNP

SHFQTLSNPHQDFKDTNITFCSNPAFQPENLMLQPMDSSSSCSPSQAFNFDPLSQPCFSS

LNAISNSPFDGGFDLGGCDPSFLANLSSNSPVFMGLNPQIEMGNNSQLSQNSELTHLLPM

PNNNAVISGGFGPNDFEGFGDALFLNRSSSNKVLRPLEVSPPVGAQPTLFQKRAALRQSS

NELGNLGISELRSDGIWGKRESVVGELSKKRKRKEEEEFEEGSIDGSGLDLDSDEFVEND

YKGDENGGDNGGNNSNANSSVTGGDQKGKKKGLPAKNLMAERRRRKKLNDRLYMLRSVVP

RISKMDRASILGDAIEYLKELLQRINDLHNELEATPPGSLLQPATSFHPLTPTLPYRVKD

ELGPSSLPSPKNQPARVEVRLREGRTVNIHMFCGSRPGLLLSTMRALDNLGLDIQQAVIS

CFNGFALDVFKAEQCREGPELPPEQIKAVLLDSAGFLI

>RibHLH52

MRRVYRRVEALVEEVPTKQVPSRSSLKCGRAAEFHNLSEKVVYMCCEDIGGGAGSMKKMK

ALQNLIPNSNKTDKASMLDDAIEYLKQLQLQVQVC

>RibHLH53

MVRSLKSHHEEEEEDEDEEFVSRTTDATSHIAKADGKCTGQKGNAPRSKHSETEQRRRCK

INERFQMLKDLLPQNDLKRDKASLLLEVIEYVQFLQEKLQLYEGPYQGWTQVPSKLMPWK

SNSAPVESCMDPSQLMRNGSGQEDNTVVMPAMLSNARNSAESDLSEADAYDALNHPPMAS

NQATPSQIPLQSGVLEDMHTQAHQTSVSYSGQLASQSQSQFWQVRQSTTENAVPSYALGE

QEELKVGSAETSFSNAYSQGLLNTLTQALQSSGVDLSQASISVQLDVGKRANTGPTTTFG

EKDPQNHSPGNQLRATHGLGSSYDDSGQAHKRLRTERI

>RibHLH54

MALTKDRIPNNTQMGLVQAFTYYGNVFGGSSAQVNSDQSYQPAEGGAQSVINFKTTTAGY

NNVMHSNGSSFLSFDQNPDHQQNPNPNKISDDQQYLDDDYSIWEDNLIQDFSTTRVQSTS

SYGADQPFGWHNSEENPNCGNNFHEEFGKQEEGFNKRLHKGESNQAIKKQCTNATKKAKQ

KSTTPSKDPQSVAAKNRRERISERLKVLQDLVPNGSKVDLVTMLEKAISYVKFLQLQVKV

LATDEFWPMQGGKAPDISQVKEAIDAILSSQKDRNSSSE

>RibHLH55

MQGMEDPIFTDHFDITEYFDEELAAALGDDFQNSLSSESNSSSSTLLNDIPNSSSTTNLL

CASSTQEALESPNSKHHKPNSWPTNSNNNNNDSTAAAILDQQPSVFSSGCILSFGNSGST

ENPKIPPGALNSEDEEVVAAGMLLSNKSSQGAKKSGSSNPRPASQTYDHIIAERKRREQL

SQLFVALSAIVPGLKKMDKTSVLGEAVKYLKQLQDRVKTLEEQSARQTIESVVLVKKSQL

LVEDEAAVDENFVSGSSEPLPQIEARVCNRSVLLRIHCEKHKGVLAKIFGEMERLNLAIV

NTSVARFGALALDITIIAEMEAELCVTVKDLVRSLRPALQPFM

>RibHLH56

MDTGGNGDLGCGNGGGGGLLDCSSLGNFTALPADEVLGMTTGSEHWNRVHSSFNSGWTPL

IGSITHSNMEVGTSPFSSFGSGTFPDMDSFLILQRCRYSSCSGDNPPERSGDKNEKALTT

QCAADDFAKSQGGEGTIKPSLDGNKRRRASDDWSQITQFEDTKTENDQCTGEEDEEKEKQ

KPEIKPVSKKCKQIIGREVNDSSSSGDAPKEDYVHVRAKRGQATNSHSLAERVRREKIRE

RMKFLQDLVPGCDKITGKAVMLDEIINYVLSLQKQVEFLSMKVSTVYPEANVEPEQILQR

DIRYSQGGSATILCDPGTRPYPNVAGVWGNDQLQRTSRMDFNHDLAPDNAESNGRLHKLL

>RibHLH57

MADMYNNKSTCASSSSPPETDDLSLFLHQILLRSSDPLAAAASLPGNPHRPVQPSVLSEY

GCHVTDRISTAESSSGLNSSPGAVFSSSGYYFPAGATNASSSVGTVDIDADEYDGESEEG

FEALVEEVPTKQVPSRSSLKRGRAAEFHNLSEKRRRSRINEKMKALQNLIPNSNKTDKAS

MLDDAIEYLKQLQLQVQMLTMRNGLSLYPMCLPGALQPAQLTQMPKGFYEGNGTDVTGIS

FNQETSTNTLFDIPNQSTNPAQLTAVDLSSTLSSETLFGRESAIEGDLRPLQFHTSSNSK

KDMPHDQQLNVDDHSPKNPLGFETGAKATVSISINTQASEVKDKVPEACIPGTQRPENVL

LSILECEPTLAPNLDGLLSGRSAAKDDIKSQRQDF

>RibHLH58

MLHLDDSFTGNSTNMTVLQRHQAIFGRQQEDQRQDNNAIESYAMPWLQSLVGDDSMVYQE

LMSRSMNADQYLGSMFPAFRELQFAGTEFIGNTISIAPLQLVTSSSPEVNHSFSVGLNLK

KRKADELIVEECCNDDEIGVEVRQGESAAIETTNLETSTDNSKVSDVQKPDYIHVRARRG

EATDSHSLAERVRREKINKKMKCLQDLVPGCSKVTGKAGMLDEIINYVQSLQRQIEFLSM

KLAALDPRLDFDMDRFSMEEFPAHVTSFPAAVAPPEMADLAYQFSQVQKGAAAYGIDTPI

NPMQLAPQRSTTSSSSLSIPKAPVMDGYSLRMEWLFLDNNHCRFP

>RibHLH59

MAEARVNIRQGALGGGGGGQWATSRQRPSQSSSNHVATNHRNATTTFSSLSSSQPPSSQK

NQTFMDLLKSAKVSQDDDDDEEEEFVLKKEPSPYPKSDLSVKLAGEGGKSNDQKPNTPRS

KHSATEQRRRSKINDRFQMLRDLIPHSDQKRDKASFLLEVIEYIQFLQEKVDKYEGSYQG

WNQEPPKLMPWKNNQSSVEGFVDQSLATNSGSGPKLMFAAKFDEDNIVVSPTNPRNGHNG

IESDLSTVTTLKEIDLRTVLTNKAVPLSLPLQPNMLTPTGSSSLMVPIPAGVTSGMDNVM

SQPQSPFLQSRAHATDNTVASNKLKEQELTIESGKICISSVYSQGLLNTLTHALQSSGID

LSQASISVQIDLGKRANGRLPASTSTAKDPVVAHSRAASSEEESDQALKRLKKS

>RibHLH60

MSSRRSRSRQAATGVSRNISDDQINELVSKLQELLPELRRSRSRSDKVSAARVLQETCNY

IRSLHRDVDDLSERLSELLATTDGPQAAMIRSLLSQ

>RibHLH61

MDPPIIHEASFSAANPSSYSLAEIWPFPINNGSGGLGLRMNNLSGFGEATLNRDVSVDES

TVTEQSGGGGRRKQQPREANSEDESSKLVSTSSGNDMNNSNGKRMKTSRSRDENGVSQSE

DEENSGLGNNPAEQRTKSSVPPKQDYIHVRARRGQATDSHSLAERARREKISERMKILQD

LVPGCNKVIGKALVLDEIINYIQSLQRQVEFLSMKLEVVNSRVSSPIEGFPPKELAPTFD

ATGMIFGTQGTRDYVQVSQPEWLHMQIGNCFDRAP

>RibHLH62

MSAFSHQHQPFLLDSSFSTPIKIITSCLENSTKVDAGINDDDSSSVVDVKAESGEQVTQK

LIPMDKKRKYRDGSAQSKDAREVKGKKQKRCNGGMEGDEERKTNKAGNKKKLNEEAPTGF

IHVRARRGQATDSHSLAERVRREKISERMKLLQALVPGCDKVTGKALMLDEIINYVQSLQ

NQVEFLSMKLASVNPMFYDFGMDLDAFMVTPEIRLNELVSPLMPKSNHHQAIAFSDTTTG

AAAALTGPNSYSLVDTPTSSSLLFQQVQRPNILSQDNEQVLWDVEDQRQRVINQSGFSNN

LCSFHQ

>RibHLH63

MSAFSHQHQPFLLDSSFSTPIKIMSGLLEEPNTPFFSQFVYPPEPVHQIPVHQIPVHHFS

TSCLENSTKVDAGINDDDSSSVVDVKAESGEQVTQKLIPMDKKRKNRDGSAQSKDAREVK

GKKQKRCNGGMEGDEEKKTNKAGNKKKLNEEAPTGFIHVRARRGQATDSHSLAERVRREK

ISERMKLLQALVPGCDKVTGKALMLDEIINYVQSLQNQVEFLSMKLASVNPMFYDFGMDL

DAFMVTPEIRLNELVSPLMPKSNHHQAIAFSDTTTGAAAALTGPNSYSLVDTPTSSSLLF

QQVQRPNILSQDNEQVLWDVEDQRQRVINQSGFSNNLCSFHQ

>RibHLH64

MGSLSTYKSMLGVHDEEEDQEWYIHSSNNNSNINFSSNFATEAENNNLLLHSVGSSSSCS

PSSASVFQDHHYFLNPPPKPTIPSLLNNPLDNSFDMGFLDSQLNNRVLTGFNDLTSQTQM

GISNLASDPQFSTTHLLQLAENKSMAGFSSLGFQGFHENSLFLNRSKVLKPLDNFASIGE

QPTLFQKRVRKNLETNGVNLGVLGSEGGGELLRNGEGYNGNMEVSEKKRKLISMEDVDEF

SIDGSGLNYDSDEFLESCKGEESGRIGGNSSNGNSTVTGGDQKGKKKGLPAKNLMAERRR

RKKLNDRLYMLRSVVPKISKMDRASILGDAIEYLKELLQKINDLHNELESNPPGSSLTPT

TTSFYPLTPTPPSLPCRIKEELCPSSLPSPNGQPARVEVRLREGRAVNIHMFCSRRPGLL

LSTMRALDNLGLDIQQAVISCFNGFALDIFRAEQCREGQDVHPDQIKAVLLDSAGFLA

>RibHLH65

MYPSSSSPSSHPSTNNPTGLTRYGSAPCSFLTTAVDSLTTTAVAATRDFSVLGPHHQPTH

NNNNANHPNNIHSSINNTTSSNASQGDSRPYCLNAIATTVGGEFTAAGGGSRINLNSKSG

GGGGGSGGLVRHSSSPAGFLDHLNASNNSATGVSNGNGFPLTRSIGSYNSKGVSDTGHGI

SRLSSQSSFTRDDALSCISGERENGRRKTAHSYATTSFGMGSWDDSSAGFFSVSPGKRAR

NIGGDNCANTAESQFQFSLSQTDLEMSTTETRFHIPHDSVPCRIRAKRGYATHPRSIAER

DRRTRINGKLKKLQDYVPNLDKQTSYADMLDLAVQHIQGLQKQVQKLKEEVENCTCGCKQ

T

>RibHLH66

MDKEGNYSGNFQGADYSIDHHHQLMKPRINEETYVETSNQIADYTSLTSHPRSSTSPDKL

SFADVMQFADFGPRLGLINQTKIPEEGQDEEVETGIDPVYFLRFPVLNEDRYKGLGEERG

GEEIGEGEGRGNSENNNNAASEGLGIVGGNFEKNLAADDHGVSNKNKRKRARSVKTSEEV

ESQRMTHIAVERNRRKQMNEHLRVLRSLMPGSYVQRGDQASIIGGAIEFVRELEQLLQCL

ESQKRRRLYGDPPPPRPMGDSSTSSLANIPQPSQPPPPFFPPPVVPSLLPNGHDNQLKLV

EFDPTAGLREETAESKSVLADVEVKMLGFDAMIKILSRRRPGQLIKTIAALEDLQLIILH

TNITTIEQTVLYSFNVKVTSEARFSAEDIASSVQQIFSFIHANNTM

>RibHLH67

MICGKKELEEAESFQEQLLLEQFPWSLPPIHYSFSPAHFDPNPVHDHNPFLLPPSVPSPY

GGLLFSRGSSEHDHLRLVSEAVGHAAAQYSSSSAPFGLQAELDKMTAQEMMDAKALAASK

SHSEAERRRRERINNHLARLRSLLPNTTKTDKASLLAEVIQHVKELKRQTSQIAETSPVP

AEVDELTVDASDENGSLVIKASLCCEDRSDLLPDLIRTLKALRLRTLKAEITTLGGRMRN

VLFVTAEEESNVDEFGDQHRQDCSVSSIQEAFRAVMGRRNGDECSTSSGGVTKRHRTNVN

MVENRSL

>RibHLH68

MDVEGKDEFEQEKGDEDLMSYHSPNVSSDWRFGGPNLTNPLMGSIPTAKPMTVCSKGGLM

ESSSSSSAPLMDSFCHTVWDHPTSTSQNLGFCDINLQNSATTSNNTLGIRKGPLAPLSVD

RTVDIGWAPPNPMVKGGGVFLPNAHGMLLPQSLSQFPADSGFIERAARFSCFGRGNFSNM

VNSFSVSESMNPFARSGPVMMQQAQEVFGGNGLNSDVKMNEASKGVSLSAEQGATTEGSP

LKNERRSNSFGMSANGSDEAENSGGDGQDNPSSSQPIGLTKRKRGGGGQDNELGQNEALQ

PTGEATKDNTEIEPKGDLTPTSTSNKPSGKHGKQGPQPSDSQKEDYIHIRARRGQATNSH

SLAERVRREKISERMKFLQDLVPGCSKVTGKAVMLDEIINYVQSLQRQVEFLSMKLATVN

PRLDFNLEGLLTKEILQSRAGPSSTLGYSHDMTHMTMPFAPLHPSQPGVPVMANSSDALR

RSINTQLAAMSEGYKEPSSQVPNMWGEDELHNVVQMGFNSSAPLDSQELNAIGSLLELGW

STVTQQICI

>RibHLH69

MKSGQQEEVEEEDEVIVSKREGLSSNREDGKNKNKANATRSKHSVTEQRRRCKINERFQI

LRDLIPHSEQKRDTASFLSEVIEYVQYLQEKVQKYEGSYQGWSMEPTKLMPWRNSHWRVQ

SLVGPTQAVKNDSGPASTFPGQFDENNITCSVAMHPISWNSTESDSSRDPSGQIDDQQSK

LPNKAIALPIPLHTHVSSSGPGDDVVPHPIHRPISDAQLTECPITSGALTEQEDLIVEGG

SISLTSVYSQGLLNNLTQALQCAGVDLSQSTISVQIDLGKRANRRLTSGSVVSNTKDSES

ASPSHQPIGHLRDSTNGEELDQAQKRLKI

>RibHLH70

MFPIKESDEESAVFEDLIMDDDASQLERSNTYSTNYRVGKRQQKLAAIPEENDEVVASDN

KQRAVHKEIERKEIERKRRQEMGYLNASLRSLLPLEYIKGRRSISDHIHESVNYIKHLEK

KTKELRIKRENLQMLSNSSVLSAASSSSSNLLPISITVSPCRGGVEILINSGSREEGFPF

SKVLEILLEEGLDVFSYVSAQVNDRFLHTIKSEVLLSLGFLFIFFFFFAYFRVSDTACVD

LSAVQQKLNDMINLFLFGISLETAWESSMNMAQFITFKASVNMRATPSNHEPPDLCRGST

FLPSIC

>RibHLH71

MGVCDNLSDHFFEESELEDIFSILENLENVEEFPPLEPENGLGSNEGETGGGLFSQKSTC

SSAGNLQLESEAEVELAADNYLPKRNKRQKLSTAATSTDEEIPQGEGQLNRISHITVERN

RRKQMNEHLTVLRSLMPCFYVKRGDQASIIGGVVDYINELQQVLQSLEAKKQRKVYSDQV

LSPRLIPSPRPSPLSPRKPPPSPRLLPPNLPISPRTPQPSSPYMPRLMQHLSPLPSPCSS

SSSAPSSVVVDNATTNELVANSKSAIADVEVKFSGPNLLLKTISPRIPGQATKIMSTIEE

LSLEILHVSISTIDETMLNSFTIKIGIECQLSAEELAHQIQQTFC

>RibHLH72

MEGKYGHENPLQLMSSAFGGTSNDASDSYQGNPNHKYVTADGKNHIPPVPFPLSQWIHCQ

ETSPGSVEFITERSVTEISREMSYHNAIVPSGGMDVWKVGGDAVLQEVKYARPQNWPPMN

SFTDAEYQLHAQGQESNELHSRASVAAPAFLAKPHNHASRRRASVTATATATATDRARRM

RITERHHALNELLPRHKEGSKASQLDDITDYIKYLQLQIKDLSRSRLGGEPTTDPFVFLE

GYGHYILHEQMLNEPLEEMMGKLLEVNPSAASRLLESRGLVIMPTNLVEGLHQAM

>RibHLH73

MANNTSDGPTDDFFEQILGFPAYASTDANLAGNSGTPMMLQLSSGDGSAHLGGGGGGGGG

FHGSVFPLGLSLDQGKQGFREDVGDGRSSSSSMKNTFSGQPMPNTVSAMAHPPSIRPRVR

ARRGQATDPHSIAERLRRERIAERIRALQDLVPSVNKTDRAAMLDEVVEYVKFLRLQVKV

LSMSRLGGAGAVAPLVTDIPISSLEQDAGESGRNQAAWEKWSNDGTEKQVAKLMEENVGA

AMQFLQSKALCIMPISLASAIYHTQPPDATTVIKSESHPPS

>RibHLH74

MSSIVHEKLSQTDNMKFPPLPPNYAESRSSCIFYDSYAYTYTHTRLQAYGIAFVSIPLEK

EVSKSKSGNSALIWGDGYCREAKGSEVGTGNRPQDRKYEGGDRKKRVLQKLHACFGGVEE

DNCALNLDLVSDVEMFYLTSMYYSFPFDKPSTPSQAFNTGRVIWASDTKSCSEHYQSRSY

LANLARFETVVFVPLKSGVVEIGSIKTVPEEQNLVQVVKTAFAGPLPSQGKLVPKIFGHE

LSLGGTKSRAMTISFSPKVEDDSGFASESYELQGVGSNQVYGNSSNGCRSDEGEAKLFPH

MNLGGLEQGHEDLLIQSDERKPRKRGRKPANGREEPLNHVEAERQRREKLNQRFYALRAV

VPNISKMDKASLLGDAISYITDLQGKIRILEAEKDMVNYKQKQGSVPEVDFQARHDDAVV

RVSCPLDSHPVSRVIKAFREHQVIAQESKVSTTEEGEIVHTFSIRAQGGTAEHLKEELVT

ALSR

>RibHLH75

MNHLVPDFEMDDDYSIPSSSGFTRPKKSPMAEEEIMELLWQNGQVVMQSQSQRSLRRPHV

GDGVNPPEHTAAAKEIRTEEESATQLFIQEDEMASWLHYPIDDSFDRDFYADLIYPAPTA

PVAPIPDDVRVSTPPAAATAPRPPIPPPARRPEVESPARTQNFLLFSRPKKGRTEAEPSI

CNKAARDSTVVDSSETPAPGSTVSHAAAQVSGGNVWYGAAASGAAAVAVAVAVASSAAGE

ETGLASSPGGSGASVSTSAEPVHKPVLAAVTEDRKRKGIEADDVECQSEDVEFDFPDAKK

QAHGSTSTKRSRAAEVHNLSERRRRDRINEKMKALQELIPRCNKSDKASMLDEAIEYLKS

LQLQVQMMSMGCTMVPMMYPGVQQYMPPMGMGMGMGMGMEMGMNHPMMPFPSVLPGSALP

TAAANARLGPTFPMPAFHMPQPVPLPDPSRIQATSQSDLMLNSMGTQNPNPPWMPSFADP

YQQYRGLHHMQLPLPQNQAMMQPSPSKPNSSREVETHQNHQSG

>RibHLH76

MDKEYFLNAGIPPPLHFEPSQIPNWQSQSSEFLFNPNWDSNKSTDQQYSNFDSALSSLVS

SPAASNSTISQDNFVIRELIGKLNTTANSGEFSQAPYVGSRSTTSSPSKSAQYLVDHFGN

EKMLPNLGNSMGMNPNLVSTDPGFAERAAKFSCFGSRSFNGRISQLPYRSSSASMACSEK

VSRVSSSPLPKADGSPIQSKNPTQTHVEMGPANGADAGFGKASEQDSARFASSNEGSSVS

EQIPSGLKTPSDSNSRKRKAVSRGKSKEVASKAVEENDDPNAKRSKPIEGSSELGSVKTE

EDTKTGDEKANQKAPEPLKDYIHVRARRGQATDSHSLAERVRREKISERMKLLQDLVPNC

NKVTGKALMLDEIINYVQSLQRQVEFLSMKLASVDFNMDTLLSKDEFQANGSLSHPMYPL

DSSASAFFGHQTKETPQVHTNFSIGAMTQCSIDPNVCRNNLMQSTLVDEFGRGLPQFPTL

CEDDLQSIVQMGFGQNLEL

>RibHLH77

MEESSHRHGQIIEISGEVSGGEKSVGGSKLCGEAPCGFSDAVTITKDAKERAASMRKLFI

AVALCVVFMGVEVAGGIKANSLAILTDAAHLLSDVAAFAISLFSLWAAGWEATPRQTYGF

FRIEILGALVSIQLIWLLAGILVYEAILRLINDTGEVNGFLMFLVAAFGLLVNIAMAVLL

GHDHGHGGHDHGHGHSHVGHGHGHSHGHGHSHGHGHDDHDHNGHGHRHGVHITTHPFSHE

EHPRDEEHGHAHEEDLVEPLLKQNSHDKNKPSSEKEEKEKRNINIQGAYLHVLGDSIQSV

GVMIGGALIWYKPNWKIIDLVCTLIFSVIVLGTTIKMIRNILEVLMESTPREIDATKLEK

GLCEMDDVVAVHELHIWAITVGKILLACHVKIKPEADADMVLDKVSSLFSEVMNQMRAKS

WEPAIRLRALPTPLCSQSRDLVRWVQLHFRQKSGSNMGNLLPGRTGFPMVGESLEFLSTG

WKGHPDKFILDHHTLTQDLKPHFLEEEEEHLDVEMECLEQETSNNTMRKLGFVSPSDSSF

EEMRIPFLEMLQSGDSPAFSPFCELSFQALLSLQQFKKPPWEGSHSHHQYSPELRECSGG

GRFKGQQDSCLTHDAVEVQISPVKSETMDHHNTHSAIYVEGGANSDGNQHDGSKAVELEE

RPGEAAAWKGQKRKRVKRTRPSAKNKEEVESQRMTHIAVERNRRRLMNDHLTALRSLMPS

SYIQRGDQASIVGGAIDYVKELEQLHQSLQAQKRMKKSDLLEQSGCRGSSSETTSTSTSS

SSTTTGILATSSPQTCQFGNMKSETGELGNCTTTEGINVRQSYEFTAVRKSGGVEVHVTV

IQNHVNLKVECPRRAGLLLNAIVALEDLGLTVLHLNIMALENSAQYSFNLKIEEGCEIVS

ADGIAVVVLQIFCFIHRTTLPS

>RibHLH78

MDELIISPSSSSSFISSQETPPTLQQRLQLILQTQPDHWTYAIFWQSSSSSSSPNDQNTP

ALLSWGDGHFQPTNNKNPLPKDNENSAEWFYIMSLTRSFSGAGDRGGAPSKAFSSGSLVW

LTGGNQLRFYDCERAKEAQIHGIKTLAFIPTSVGVLELGSSDVVKENWGLVQQAKALFGS

DLDVIPNRGNGSSEFLDGNVCFAEEDMVNGGGTKQEAIVETECSDLGPFPVPATFGFHHN

GNAEPEQQQRTQKKRGRKPGVGRETPMNHVEAERQRREKLNHRFYALRAVVPNVSKMDKA

SLLSDAVSYINELKTQIQDLKSQLHDSKSSNKKMKTELSSQLTADTTDNHSSAATSVDQT

ISPNNNSDNNSSGVPLEVEVKIVGADAMVRVSSDNVNYPAARLMEALRDLELQVQHASAS

SVNDLMLQDVVIMGPHRGVISEEGLRTALLSRLG

>RibHLH79

MYQENAMRLPENEFSQTVPNFTPTFSSMEELSFHQNPQQKASAGMEIEELLHRMGFERNT

PLIQEMATESNLFHLPNSSFSYPDQTQNSPSFLPTLGFLGDISSNAHSALDSNSVNLDPL

FHLNLPPPQPPLFRGLMFESPIPNGGYNMNTGSLFGVVDEREGSGGGYGFDNRVFEFNGG

DRSCRGRKSNGKGSNRQLNTEKQRRGQLSDKYEVLRNLVPNPTKADRASVVGDAIGYIKE

LLRTVNELKMLVKRKSWSIERIKRHKTESDGPDHQDQSSYNGLRSSWLQRKFKDSEIDVR

IVDDEVTIKFAHPQKKINCLLPVSKALDELQLDIQHVGGGLVGDNYCFLFNTKICEGSCV

YASAIANKIIAVVDKQYPAIPTNQ

>RibHLH80

MDGFQSSQMNTGLFSSNSKHPDVQNMKGRESMNTDDLYNQYQNQQQGSTLVRYRSAPSSF

FASLIDDGGEEEEDLLNRQSSSGSESIFETTAEKQQYSSIYETSLDRRDLVNQNESWVTS

TCGGGDLQGFYFKDDVAMENNSIMEHGKKSGGGAVGGGSSNCSNLIRQSSSPAVFFSALT

ADTGFGDMKDVGSFRAGNGTNGKASTSNRFNGIINFSSGPSSSSRFMPKITESGNGNGTI

EASSPQTRFQNDSWTVTGLKRNRDDNESQLFGFNALNGDSSSYATGLTHHLSLRKNSAEI

DAVEKFLQFQQDSVPCKIRAKRGCATHPRSIAERTRRTRISERMRKLQELFPKMDKQTST

ADMLDWAVEYIKDLQKEVKTLTDKGSKCTCSGEGKQVPKQTNLS

>RibHLH81

MAAIDGMGSSLKAGRAKLGRIESIQKERERRGKMVEMFSVLQSIVPNIFPKATRENIVTE

TIQYIQRLEEERDRLETLKKSQESTAVMPTLTQCTNRRDSAVNVAISGNGVVFFGLRTAA

NRRHSVVEILGVFESHEAEVLAASVSVNGQQRR

>RibHLH82

MLPSCGKRRERGGDEEDDKGMIIMEKSVIHPLMAAIDGKVSSLKTGRAKLGRIESIQKER

ERRGKMVEMFSVLQSIVPNIFPKATRENIVTETIQYIQRLEEERDRLETLKKSQESTAVM

PTLTQCTNRRDSAVNVTVSGNGVVFFGIRTAANRRHSAVEILGVFERHEAEVLAASVSVN

GQQRRLDLTVTAFVGGNGDVVEKIKRELLNL

>RibHLH83

MLPSCGKRRERGGDEEDDKGMIIMEKSVIHPLMAAIDGKGSILKTGRTKLGRIESIQKER

ERRGKMVEMFSVLQSIVPNIFPKATRENIVTETIQYIQRLEEERDRLETLKKSQESTAVM

PTLTQCTNRRDSAVNVAISGNGVVFFGLRTAANHRHSVVEILGVFERHEAEVLAASVSVN

GQQRRQLDLTVTAFVGGNGDIVEKIKRELLNL

>RibHLH84

MANNPSEAYPDEFLEQILAMPSYSSLAGTDGASSQSAASPLSFTGRMGLQHPFIPLGLSL

DNGREEINGGGFAGKSERESMNMGSLFPVFEHLQPHSVRQSVSHQVFHGQPTTSTTVSVP

HPPSIRPRVRARRGQATDPHSIAERLRRERIAERMRALQELVPSCNKTDKAAMLDEILDY

VKFLRLQVKVLSMSRLGGAGAVAQLVADIPLTSIEGDNSGGCNQNAWDKWSDHDVEQEVA

KLMEEDMGAAMQYLRSKALCVMPVSLAALIYPTLPPDAPELVKAERPKPNGPS

>RibHLH85

MVPVGIFSIISSTMFHRPDLYWGSQSVDVQVDFSLTEPMLQDEDCIEKECPKKRARNDSC

SRLGTKACRERLRREKLNERFVELSSSLEPGRPPKNDKLAILGDAIRVVNQLRAESQEFK

ETNEKLVEEIKSLKAEKNELREEKVVLKEDKERIEQQLKAMTVSPPGFLPAPHPAAALPA

YHAAANKMAVFPSYGYVPMWQYLPPNVLDTTQDRELRPPAA

>RibHLH86

MNEAGNTNGNSSRRRTHIDDDDDYEIDDRVHKSKNLDAERRRRKKLNDRLLELRSLVPHI

TNMNKGTIVTDAITYIEELEKSSTGLRNQLLEMEATVVEESKLPIDAAEEMKNWGIEPEI

KVNQIDRNKLWIKMVFQKKRGGFTKLVEAVSVIGYEFTDTSVTTSSGAILVTACLEGNRG

GILEPEHVRELLLEIMGGI

>RibHLH87

MNEAGNSNGNSSRRRTHIDDDDDDYEIDDRVHKSKNLDAERRRRKKLNDRLLELRSLVPH

ITNMNKGTIVTDAITYIEELEKSSTGLRNQLLEMEATVVEESKLPIDAAEEMKNWGIEPE

IKVNQIDRNKLWIKMVFQKKRGGFTKLVEAVNVIGYEFTDTSVTTSSGAILLTACLEVRF

KYTFALSNTKIESSKRKI

>RibHLH88

MASVPENLTKQLAVAVRSIQWSYAIFWSISSRHPGALEWGDGYYNGDIKTRKTIQAVEFN

ADQLGLLQRSEQLRELYESLAAGESSPQARRPSAALSPEDLTDTEWYYLVCMSFVFNIGQ

GLPGRTLSKGQPIWVCNAHYEDSKVFSRSLLAKSASIQTVVCFPFLGGVVELGVTDLVLE

DPSLIQHIKTSFLETPYPKVSKISNSFTGNANQGLIVHAKLDHEILDTNLNPVFQCEDFE

VCSPNNSSNGFGINPQPEESFLVEGLYEANSQVQSWQLMDDEVCNSVHNSFSSSDCISQT

LVNPEKASPLPDEMTKIGLQANDIHYQSVISTLLKSSQHLILGPNFRNSNQESSFVRWKK

GGLSSIQTPRSRTPQFLLKKVLLEVARMHGDHMSECSREDSGRKDAIWRPQVDDIDSNHV

LAERRRREKLNKRFSVLGSLVPSDPSTSKVDKVSILDHTIEYLRELERRVEELDSGREVS

EPEVRTRKTPKDSAERTSESHGHNKKPSINKRKVRDIDEIKDPYNTTVSIIEKEVLIEIR

CPWRECLLLEIMDAISHLHLDSHSVQSTNSDGILSLTVKSKFKGSSFASAGVIRQSIQRV

VRKF

>RibHLH89

MKASSCPNLNRETTSFPNFFTLFQDPDRHDNTTTLNKRPKIDRGDNTEDSEASPFKRPKI

DRGDNTEDSEASPFKRPKIDRGDNTEASEASPFKRPKIDRGDDTEASEASPFKRPKIDRG

DDTEASEASPFKRPKIYRVDNTQASEASPFKLPKIDRGDDTEASEASPFKRPKIDHGDNT

QASKALPFMRPKIDCGDDTEASEASPFKRPKIDRGDNTQASEALPFKRPKIDRGDDTEAS

PFKDILSSFISVDEMNEPGYPPDCWDEEDDQTLAKWPPSPNGSDTASVGSNQFNPESPGI

HGGDSKMSESTFDFFRNELEPPRFEQALGSLSTSSVLDPLYHEHYSRSLFKRFSSDSGPS

TMNIIAVVRKPNNQERAITFFRNLNLVTVQERAQRSRPMSSTQLHHMISERNRREKLIES

FQALQSLLPPLGTKKDKASVLATTTDYLASLKAQVEELTKTSRDLEVKVQLQLATRKEAA

NDHDHQEVSLSPDQRLDVRITHEAESTSQAKVIGLQVVWRGERNVMDSVTKILEFMRQVE

NASLLSVEVDAHSINPINIVRLRLRIEVNLEMEVRNLFPDDFSQQLAPPIELPHPTDQPR

PSSPSSSTSSLYQIPTSQFTSLETKEAITTEAGTSQKPSAFKRFRPALGPSIMHNITAGV

RKPNLQKRAITFLTFSSLIRIPGRKQGCRAMSSTQLHHMISERKRTRKLFESFQALKSLL

PPGTKKDEASLLASTTDHLVSLKAQVEELDKRAKHLEEQVQLLTTKEDANDHQEASLFPN

QRLDVRITEAAQALIIGLLSQTNLYPNPHFTPNQRNDDDRS

>RibHLH90

MSHIAVERNRRRQMNDHLKVLRSLTPCFYIKRGDQASVIGGVIEFIKELHQVLQSLEAQK

QRKSLSPGPGTPSPRPLQLSPQPDNAPFGIDLNNNVKELGGACCNSPVADVEAKISGSNV

NLRTISRRIPGQVVKIIDVLEKFSFEILHLNISSMEDTVLYSFVIKIGLECQLSVEELAI

EVQKSFCSDQAYINNM

>RibHLH91

MSHIAVERNRRRQMNDHLKVLRSLTPCFYIKRGDQASVIGGVIEFIKELHQVLQSLEAQK

QRKSLSPGPGTPSPRPLQLSPQPDNAPFGIDLNNNVKELGGACCNSPVADVEAKISGSNV

NLRTISRRIPGQVVKIIDVLEKFSFEILHLNISSMEDTVLYSFVIKIGLECQLSVEELAI

EVQKSFCSDQAYINNM

>RibHLH92

MANNPPDGYGDDFLDQILAVPSSYSTLTDATAASQLSSTAGGGGGSGFQQQQQFVPLGLS

LDNGRDEFHSGGFGVKSERESANMGSFFPAFEPLQPHSFRQSAPQFHQAFPGQAAPSTTT

SMPQPPGIRPRVRARRGQATDPHSIAERLRRERIAERMRALQELVPSCNKTDRAAMLDEI

IDYVKFLRLQVKVLSMSRLGGAGAVAQLVADIPFSSVEGDNSEGECSQSAWDKWSNHDIE

QEVAKLMEDDVGAAMQYLQSKALCIMPISLASAIYPTHSPDGSMLVKPEPNGPS

>RibHLH93

MLSWSPPLFSPFGWPLEDSVSITHEENNFFTREAEANSSTPSFLNSPPLLSHEPLIAINS

GEGECKNSNDDVDRECDDNIATTTPTITAKKLNHNASERDRRKRINDLYSSLRSLLPAAD

QTKKLSIPATVSRVLKYIPDLQREVEGLAEKKEELVSRIHKSKKIQDLAHIKRRTKCVNQ

NTLSVVSASPLGEREVLIQICTIKVEESILISEILLNLEEDGLILLNASCFESFGDKIFY

NIHLEVQGGQRVEFETLRERLLSFV

>RibHLH94

LDMELLAFQDHLFYHPDYPCSTSIDPGLFHHPANSDLNILPCLALPPPPPPPLDNSIPPQ

PFKCSYQYPKRQKCYEDEYYDYCYYPEEELMPSLMPDGFVANPPLLIPEFSLPSEIYGPV

EASGGGGGGGGGSLSAQSIAARQRRRKITEKTQELGKLIPGGHKMNTAEMFHAASKYIKY

LQAQVGILEFIGVRRS

>RibHLH95

MDIDSNGNSNWFFDCGLMEDISAVPGGDFRAEAPAPATCFSWPAQPLNSSSYGSAEVDCS

FGDSESQKEGASRKRSKPGSCNASGSKACREKLRRDKLTERFLELGSVLEPGRPPKTDKA

AILSDAVRIVTQLRSEAQKLRESNEEIQERIKELKAEKNELRIEKQRLKEEKEKVVQQVK

AMTAQPGFLPHPSAAMPAGYGAQGQAADNKLMPFISYPGVAMWQFIPPSVRDTSQDHVLR

PPVA

>RibHLH96

MVSPENTNWLFDYGLIEDIAVPASNFGAPNSGFSWAMQPLNGPSDVGYENDGSFGDSDNH

RETGSKKRARSELSGASSSKACREKLRRDRLNDKFIKLASVLEPGRPPKTDKSAILVDAV

RMVTHLRGDAQKLKDSNSCLQDKIKELKAEKIELRDEKQRLKAEKERLEQQLKTMNAQPR

FLPPPPAIPAAAFAAPGQAAGNKMVPIISYPGVAMWQFMPPAEVDTSQDHVLRPPVA

>RibHLH97

MKVEVCMGDGGWSEEDKAMGAAVLGTRGFDYLISSSVSAECSLMSVGSDENFQNKLSDLV

ERPNASNFSWNYAFFWQISRSKAGDLVLGWGDGSCREPREGEESEYTQIAHSRLEEVTQQ

RMRKRVLQKLHTLFGGSDEDSYAFGLDKVTDTEMFFLASMYFSFPRGEGGPGKCFESGKH

VWISNSLKTSSDYCVRSFLAKSAGIQTIVLIPTDVGVVEFGSLRSIPESFELVKSIRASF

SSFSSLAKAKPTKDVNAHFFKLGNGERPDGIPKIFGQDLYSGRSQLREKLAVRKAGEKPW

EAYSNGNRTPFPNARNGPHGSSWTQFQGVKQVAAAGIYSPHTPSNNLPEVINGVRDEFRL

NHYQPQKAAAPMQIDFTGGATSRPPITSVESEHSDVEASGKDELAGPVDEKRPRKRGRKP

ANGREEPLNHVEAERQRREKLNQRFYALRAVVPNISKMDKASLLGDAIAYITELQKKLKD

MESERENLGANPILENQTRVPDIEIQATGHDEVVVRVSCPLNTHPVSRVIQAFKEAQITV

LESKLATGNETIFHTFVIKSQGSEQITKERLVAAFSHESNSMQPL

>RibHLH98

MSQCVPSWELDENPSPPRLKSTLRPHSNSNSIPPDIIPMLDYEVAELTWENGQLAMHGLG

PPRVNPKYPNWDKPRAGGTLESIVNQATYLPQGKSGSGDELVPWFDHHRSAASSASATMT

MDALVPCSTTSGGGGGGAGRGTASTWTHVMDGLGKHVVGCSTRVGSCSGAKTCDDEVLLK

RERVARKQGAAQEWSCSRDNNSVDGSATCGRESWQLTLDTCDRELGTMSGFTTTSMGSLE

NTTSSALQSTKATTTATTDDHDSVGHSRFQNRKAGDVQEKKKGGGKSSISTKRSRAAAIH

NQSERKRRDKINQRMKTLQKLVPNSSKTDKASMLDEVIEYLKQLQAQISMMSRMNMSSMM

LPMTTLQQQLQLSMMAAAPLGMGMGMGMGMGCVDINTLGRHNIPGVPPVLHPAATAFMPI

TSWDGTAGSDRLPAPAGVIPDPMSAFLACQSQPMSMDAYSRMAALYHHQMHQPPPSGTKN

>RibHLH99

MYQDSVCFDPNTMQKGLPENGFSQTLPNITTTTTSFSMEELSYQEESAAAAAMEMELQQQ

LGLDTENCYANNHHSNLMDTSHLMQEVVGHDSNQVLIPNYDHNSSWDINNTNMNTHDFQE

EHLMNHVNEMQNSHHLLNLLRLPKVSPSSVLPNSSFSLGFLGDHFPSTDGSGGSASNVAY

DPLFHLNLPPQPPYFRDLFQSLPHGYNLPGSRSGSLFGGVEEREGSGGGEVGRNFDNGVL

EFQTDWDMNGMAKVGNGKKNTKHFATERDRREHLNSKFTALRSLIPNPTKPDRATIVGDA

IDYIKELLRTVNELKILVEKKQCGRERIKRHKTDDGTSTTTAGEVLEDSCNKIKPDPDHS

YSNGSSTLRSSWLQRKSQDTEVDVRIVDDEVTIKLAQRKRINCLLIVSKVLDELQLDIQH

VAGGLAGDYYSFLFNSKIGEGSSVYASAIANKLIDVVNRQYAAIPPTHSY

>RibHLH100

MYGTMSRDTNLLFPSTFKQTDGELVKNRESMNTDLYQQQHHNQSSSGLMRYRSAPSSFFS

NLVGGGEDSESEIMFTKFMSAVGSGDSGPQDLQFTIKHETKPEQENQYSGAPNTAPLVYR

APVDQNPFSNHGDSGCGKDTSLSPVRGKGSNLVRQSSSPAGLFSNLTVENGFAVMGDVGN

FRVGNGTNGKASPTKRLNGHINFSSGTSSCSRFMPQISENANESSSPDNEHLENDNSSNG

FYVPSFPNDSWNDSGFTGQKRNRDGEEKFSGFNALESQHRDSRNYATGLTHHLSLPKTSA

EMAAVEKFLQFQQDSVPCKIRAKRGFATHPRSIAERMRRTRISERMRKLQELFPNMDKQT

NTADMLDLAVEYIKDLQKQVKTLEDTRSKCTCSSKQNQSSNPSG

>RibHLH101

MDEIIHPSSSLSLQQRLQFIIQSRNEWWAYAIFWQATKDINGNFLLSWGGGHFQGTKQCS

VPNKLANNGHDQHEFGVKRGIQDNYSDIDRLVNGHVLDAEWFYMMSTTNSFVAWDGILGQ

PFSGGVYAWLAGEHELKLCDCERAKEANAHGIQTLVCISTAYGVVELGSTEIIQEELDLL

LLAKSLFGPNNTTSVTKQPSGPGETAASTCQLYSGRSDFKGLLQSDSKVVLRSTKRGGNS

KARGETPPNHVEAEKQRRLKLNSLFYVLRGIVPNVSKMDKASLLADAVAYIKELQENVGE

LEAKLICAKSQETKISFPNKHDNQSTSITRVDHRHSRSTSLSGNGNGIMPAEVDVKVKGS

EAVIRVQCMDVNYPVARLMDAIRVLECHVRYASISKVKELVFQHVVVDQVPDGLRSEAAL

KIAIVRRFLI

>RibHLH102

MQLTMDEIIHPSSSLSLQQRLQFIIQSRNEWWAYAIFWQATKDINGNFLLSWGGGHFQGT

KQCSVPNKLANNGHDQHKFGVNCGIQDNYSDIDRLVNGHVPDAEWFYMMSTTNSFVAGDG

ILGQTFSGGVYAWLAGEHELKLYDCERAKEANEHGIQTLVCISTAYGVVELGSTEIIQEE

LDLLLLAKSLFGPNNTTSVTKQPSGPGETSTSTCQSYSGRSNFKGLLQSDSKVVLCSTKR

GRNSKVRGETPPNHVKAEKQRRHKLNSLFCVLRGIVPNVSKMDRASLLAGNTTRDLFDAV

AYIKELQENVGELEAKLICAKSQETKISFPNMHDNQSTSITRVDHRHSRSTSLSGNGNGI

MSAEVDVKVIGSEAVIQVQCMDVNYPVARLMDAIRVLECHVHHASISKVKELVLQHVVVD

QVPDGLRSEAALKIAIVRRFLI

>RibHLH103

MKIIHPSSSQSLQQRLQFIIQSRNEWWAYAIFWQATKDINGKFLLSWGGGHFQGTKQCSV

PNKLANNGHDQHEFGVKHGIQDNYSDIDRLVNGYVPDIEWFYMMSTTNSFVAGDGILGQT

FSGGVYAWLAGEHELKLYDCERAKEANEHGIQTLVCISTAYGVVELGSTEIIQEELDLLL

LAKSLFGPNNTTSVTKQPSGPGETSTSTCISYSGRSNFKGLLQSDSKVVLCSTKSGRNSK

VRGETPPNHVEAEKQRRHKLNSLFCVLRGIVPNVSKMDRASLLAGNTTRDLFDAVAYIKE

LQENVGELEAKLICAKSQETKISFPNMHDNQSTSITRVDHRHSRSTSLSGNGNGIMSAEV

DVKVIGSEAVIQVQCMDVNYPVARLMDAIRVLECHVHHASISKVKELVLQHVVVDQVPDG

LRSEAALKIAIVRRFLI

>RibHLH104

MDFVSSAFQCDPIDELLEFSSIPGQQRRIHKDPLQVVSASRNNDVAEKPDYHPWRKSSVV

FDDSGGNPVEYKRKKIIHREAERQKRQEMAALYRSLRSLLPVEYVKGKRSMSDHMLVAVD

YIRQLQKRVEEKGRKRDELKSSFEPSPDTNAKSRCFPSCLKESVTVESSRAGFQITMSTA

MSGGLSVSKVLDVLLREGLNVVSCISINAEERLHHVIDSEVGDGTSINRSELRMKLTELI

DGVISE

>RibHLH105

MNMWTDDNTSMMEAFMTSDLTSLWPPPPPSSSTSTSIHLPPPQPIAPFNPDTLQQRLQAL

IEGARENWTYAIFWQSTMNDFPPGPVLGWGTGTTKARKTKASAKRRRPQPKSKRTGRNRS

GRGSGSRARPCTTPPRFGSPEPSSSQARTASEPGRARCSVYGPWSAYPRQRPSSSWVRPE

TDFTRPRDMMNKVRVLFNFNDTDMGSWTVPADHGETDPSSFYLTDPSPSVVELNITPANT

MIPPSNPHLSKQPTFGSSTLTENPSSNPHQMQSQSFFTRELNFSGNDGISGARKGNSQSC

KPESGELLSFGESKRSITATSNGNGNLFSGQSQFEEDNNRKKKKSPTSRGSNEEGMLSFT

SGVILPSSGVVKSSGGGGDSDHSDLEASVVREADSSRVVDPEKRPRKRGRKPANGREEPL

NHVEAERQRREKLNQRFYSLRAVVPNVSKMDKASLLGDAIAYINELKSKLQASESDKEEM

KNQMDVIKKELASKGAPPPDHQDLKMSNHLVMKLVDVDIDVKIIGWDAMIRIQSSKKNHP

AARVMSALKELDLDVHHASVSVVNDLMIQQATVKMGSRFYRQEQLKEALSAKIADAR

>RibHLH106

MALELEALSTNESLDYIVYDTISADPTFLPENSLKKPWELGCGASDSSSCMMRRCRQVGS

PEVSKKRQNAVAQGRKKRRRRPKICKNKEEAETQRMTHIVVERNRRRQMNELLAVLRSLM

PESYVQRGDQASIVGGAIDYVKELEHLLQSLEAQKLILPQQGGIRPPTTTAATAESFEAP

FAQFFACPQFTWSQLPNKCLSKSAAAVADIEVTLIETHANLRILCRRNLRQLSKLVAGFQ

TMYLTVLHLNVTTLEPLVLYSISAKVEEVCQLTSPDEIAGAVHHMLRIIEEEGT

>RibHLH107

MEGVGAFLDEEWESLNRMFSTGSEHENGFSFGTSSTFWPINSDVASINFGPTDESLSQLI

PNCDAFSSSNDSVFIPTNQNHHSSDPNNFQETNYAPESSYFYTMDNAAENKTSLPLFAPK

GKKNVTSKKNQKLSLNGIISEEENINVTTNGSGCCSSEEDSVVSQELNGEETSESKSISA

LDSDGKTRAGRGAATDPQSLYARKRRERINERLRILQSLVPNGTKVDISTMLEEAVQYVK

FLQLQIKILSSDDLWMYAPIAYNGMDTGLHQKIPPSQ

>RibHLH108

MDSIFQLDDAGRATFLQHMLHSFASTYICLWSYVPQPSNCLISTDGLYIGENNQPSSSSG

TGSRARRLFDEYRQGLFVLGNDHRVPGIAFRNGVPYMELRELDLLSLATIDPQLQFYQEA

RIKTAIFMGCNIGEIELGFSNDTQVNLEMEMRSWFPGDFSRQLAAPPIELPSTDQPRASS

SSSSLRSLSMSSPEPSPFLFNIPSITSSLLEPPIEPSNIGQVVRPLSSSTSPLHQAKQAF

IQFPSFQFPTPETTATTTTIEAGTSQKPSAFKRFRSALGPSTVTIRAGVRKPNIQKRAIT

FFRSLSLMRIQGQAQGSRPMSSTQLHHMISERKRREKLNDSFQALRSLLPPGTKKDKASV

LASTTDYLASLKAQVEELTKRSRDLEEKVQLLATKKDHDQELSLSPDQRVDVRITQEAES

TSQARVIGLQVVLRGECNVMDLVTRILEFLRRVENASLLSVEADAQVVDSNPINIVRLRL

RIEGGEWDESAFQEAVRRVVADMAQ

>RibHLH109

MFVAGTMNLVQKLLERLRAIVNFKSWDYCVLWQLADDQRFLDWVDCCCAGSENIQNVGED

LFPVSSVPHCRDVMFQHPIAKPCELLAELPSSIPLDSGIYAQALMSNQASWLNFSNNADS

KPLEETVGTRVLIPVPVGLVELFVAKQVPEDQEIVDCIASQCTMLLEQQSMIHSSSMDPN

FSMNENNQMDLNNNLFLSSPENSNLPYGVTADRMQLHSPMNSFQPFDHTSETRASSKAAF

FDGFNSNGLQVMDSTGQPMTMESSSLVLNKETDKDSVKHETGRSESISDDSDGNEDEDDA

KWRRRTGKGPQSKNLVAERNRRKKLNDRLFALRSLVPKITKLDKASILSDAIEFVKELQK

QVNDLQHELEEQSDDEGIKNNGQSEKLNVGLKCEHEETPDGNNVGAANNGFADPKKQNHD

ASIYDHKPRQMEPQVEVAQLDGNEFFVKVFCEHKSGGFVSLMEALNSLGLEVTSLNVTSY

RSLVSNVFQVEKMDSEMVQADDVRDSLLEITRFGGWPDQMAKTSENGDGMDYHHHHVHGS

NCSNGLHNHQTCSHHLHDLRH

>RibHLH110

MNGASASGGIKSAFSYCVQQVRNYDYHHYLCLLELPPSMRKAAFALRAFNVETARAMDIA

SDPKIGLMRLLWWREAIDKIFAHKLIEHPTAQALSSVISEHKTSKSWLKRSVEARINDAQ

REVNEIPETIEDLEKYAEDTVSTILYMTLQAGGIKSTAADHAASHIGKASGLLLLLKSLP

YHASRNRLCSYIPSKVAANHGLLVNQGGPLEIRMDSREGMCDAVFEMSSAANAHLQKARE

LAATVPKEARAVLLPAVPAQVLLDSLNRVQFDVFDPRLATGILGIPPLWYQLKLKWHSLR

GKFLEWMDCCCAGSENIQNGGEDPFTVSSVTHCRDAMFQHPRTKPCDLLAEFPPSIPLDS

GIYAQALMSNQARWLNFSTSNSDSNPLEETVGTRVLIPVPIGLVELFVAKQVPEDQEIID

YIASQCTMLLEQQSTINSSNMDPNFSMNENNQIDLTNNPFLPSPENSNLPYDITADRIQL

NSPMNFVQPSFFDGSNCNEFLEMDSIAKPMTVGSSSCVNKETDKDSGRHDMGRSESISDC

SDANEDEDDAKYRRRTGKGPQSKNLQAERKRRKKLNDRLYTLRSLVPIITKLDRASILGD

AIEFVKELLKQVSDLQLELEEQSGDDEGTENNGRSEILNAGLKREHENTLVGGNNMGAAS

YGSVAEPTNRKHDSDAYDHKSHQMEPQVEVAQIDENEFFVKVFCEHRSGGFVRLMEALNS

LGLEVTNVNVTSCRSLVSNVFQVKKMDSEMVQADYVRDSLLEITRFGGWPDQVAKTSKND

QDGMDCHHHDHRSGGDRPNGLHNHQTSSHNLHHLHR

>RibHLH111

MDQLNDDVFRQSIQTVNPPPEFRQPPTFSRPEAELQESVAARKFQKADREKLRRDRLNEQ

FLELGKVLDPDRPKNDKASILSDTVQIVKDLTAQVNRLKAEYATLNEESRELTQEKNDLR

EEKASLKSDIENLNAQYQQRLRATFPWAAIDHSVVMHPPSYPLPVPLPMPIPPGAISMHP

SLQPYPFFGNQNHGVIPNPCSTFVPYVTPNPVIEQPPTQYVSPAMHPSNRFNIPSKQDSR

NNPSDCQGESKFEKSEASDDVTTDLELKTPGSTPDEDSSSGQRKSKKLLTKENSHTDVSS

SSGCSSSQSAQDSSSNSVAGGGKDKD

>RibHLH112

MEISHIRSLADFGMDDPSFVPMWQFNSFGELSTASVADSFEGNLHHSFTHQMFDVKPCPE

TPHTGASRPLKIQKTNSWDSSITDLGSNPPANSSPNLVSFSNSSNYANQFGILKPKEDTQ

DSFGNQNYVLKASEGAKRISTNTRFPSNQDHVMAERKRREKLSQRFIALSAIIPGLKKVD

KASVLGDAIKYLKQLQEQVKTLEEQVRNQSTESVVLVRRYELYADGENSSSGENSSSGPF

DEPLPEIEARFCDKNVLIKIHCERKKGVVEKTVTEIEKLHLSIVNSSVMAFGTSALDITI

LAQMDAEFAMSMQDLVKNLRALKLFV

>RibHLH113

MALETVVFQQDPFSNLNSYGYKEVEEYHCRDTNSSSPDVCAGDGLLAGSPVEAPARRKRR

RNYKSLKNKVEMENRRMAHIAVERNRRKQMNDYFSVLRSLMPPSYAQRGDQASIVGGAIN

FVKELEQLLQSLESKNQLKQQSESPRLFANFFTFPQYSTRSTHQSNLLLTGHESRVTAEK

ESTIADIEVTIVESHANIKVLSRRQRKQLLNMVSWFYSIGLTILHINVTSTVDQLVLYSF

SVKVEDYCQLSTVNEIATAVHEMVTKIQVEVMPSYN

>RibHLH114

MVNPEENSNWLVDYGVIENIPVPGGDLPSLEPGFEWCPDALAGSTGLSVDFEDSFGNLDG

IKECGSRKRMRPESCSASDSKACREKMRRDRLNERFLELSSILEPGRKPKVDKAVILSDA

VQTLTQLRDEAQKLKESSDSLQEKINELKAEKNELRDEKQKLKVEKEKLEQQLKAMSSQP

AFFPYPSGIPTPFGAPGQVVGSKLVPFMGYPGVPMWQFMPPTAVDTSEDHALRPPVA

>RibHLH115

MPLSEFYAMARGKVGSVQQKTTPYSTDQSPLRDNEFVELVWDNGQIMMQGQSIRRIPIPN

NFQSQTMKLRDKDPGNVTNFKTGKFGTLESGFNDFATAMPSGEMGLSQDDDTVPWWSYPF

SDSLQHDYCSELLPELSGVTVNDISAQNSFSSIDKRNSCNQTIRGTTSISGQNGFSLELV

NGSNVSSSEDGKSSRSRSGQFFPWSFQESPAPIPSLRSGVSGVISNDTSNTKHDSCGDSI

QAQLSADDSSSIKIQKQDSGLPNASSSFMNFSHFSRPAALVRANLQNVGSMGGENKGTAP

SSSILAEPLPVDLSNGLRNEMGSYSQPNSVPPKVDAKPVVAKPLEEPHAIYQEDSDKNDK

LPNQVLGASASEGMPDGEKTRDPLVACSSVGSGNSGERASNDPTHNFKRKYRETDDSEGR

SEDIEEESTGVRKAAAVRGATGSKRSRAAEVHNLSERRRRDRINEKMRALQELIPNCNKV

DKASMLDEAIEYLKTLQLQVQIMSMGAGLYMPPMMVPTGMQHMHAAHMAQFSPMAVGMGL

GMGMNGGSLGWPTIQVPSMQGPHFSAPRPQPILGTTNFQGMGVSNLQVFGNPGQGLAMSS

PRAHLMPLPGGPPINLAGGLNANGTAAAVEVPNLAPTTNSKDLLETTNSQMVHNTDANSS

MNQESSQCQATSEVDQLVVT

>RibHLH116

MEYDGHGFLEELVATTIPIEMSDFDTNKWSFGGFDESTFHVPTNFPLGPLDQSLNCSVNE

FYFPFGGDPFQFSAPGVTDTPPFPTPDDCSMSMVENEEKPQMGNGVQMLEFQSPNNCKLE

RIQSREVPIFDMGLGAERKNRVKKVDGQPSKNLMAERRRRKRLNDRLSMLRSVVPKISKM

DRTSILGDTIDYMKELLDRINNLQEEIHEVDNSNQFNLMSIFKDVKPNEVLIRNSPKFDV

EKRNMDTRIEISCVGKPGLLLSTVSTLEALGLEIQQCVISCFNEFAMQATCSEELEQRAI

LSSEDIKEALFRNAGYGGRCL

>AtbHLH074

MGGESNEGGEMGFKHGDDESGGISRVGITSMPLYAKADPFFSSADWDPVVNAAAAGFSSS

HYHPSMAMDNPGMSCFSHYQPGSVSGFAADMPASLLPFGDCGGGQIGHFLGSDKKGERLI

RAGESSHEDHHQVSDDAVLGASPVGKRRLPEAESQWNKKAVEEFQEDPQRGNDQSQKKHK

NDQSKETVNKESSQSEEAPKENYIHMRARRGQATNSHSLAERVRREKISERMRLLQELVP

GCNKITGKAVMLDEIINYVQSLQQQVEFLSMKLATVNPEINIDIDRILAKDLLQSRDRNT

PTLGLNPFAGFQGNIPNLSATTNPQYNPLPQTTLESELQNLYQMGFVSNPSTMSSFSPNG

RLKPEL

>AtbHLH038

MGKGPLSFRRLSSIRHRKKGSAVKDDSAQTSTPSSPPPPLPIHAGGSAVGATGKAKKKTG

GARLWMRFDRTGAMEVVECDKSTIIKRASVPARDLRILGPVFSHSSNILAREKAIVVNLE

VIKAIVTAEEVLLLDPLRPEVLPFVERLKQQFPQRNGNENALQASANVQSPLDPEAAEGL

QSELPFEFQVLEIALEVVCSFVDKSVAALETEAWPVLDELTKNVSTENLEYVRSLKSNLT

RLLARVQKVRDELEHLLDDNEDMADLYLTRKWIQNQQTEAILAGTASNSIALPAHNTSNL

HRLTSNRSASMVTSNTEEDDVEDLEMLLEAYFMQLDGMRNKILTVREYIDDTEDYVNIQL

DNQRNELIQLQLTLTIASFAIAAETLLASLFGMNIPCPLYSIHGVFGYFVWSVTALCIVL

FMVTLGYARWKKLLGS

>AtbHLH159

MQPTSSMNEEFLKKWQMGLQIFRPSIDNTSVHERKKAIKLSADVAMASLRKGTTCWSRAL

IEKTATEDNFLVRQMLSGIKAETLINKKLPKKTVCHRKIVRRSKKILRRKSKSASEEAAA

KAKRLVKRRTQGLRNVVPGGELMSNDVLLLQETLDYIVSLQTQVNVMRSIVDAAEAEIER

>AtbHLH052

MIIPETDSFFFQEQPQHQPLYPDEALSPSLFGFDHYDHFYESFLPSQEIFLPSPKTRVFN

ESQELDSFHTPKHQKLIDSSFHFNSHDPFSPSPESNYLLDSYITEASNISKFQAPDFSST

FKVGWTEQGDTKKRELSAQSIAARKRRRRITEKTQELGKLIPGSQKHNTAEMFNAAAKYV

KFLQAQIEILQLKQTKMQTLDSSKVGREMQFLLGSQEIQEKLSTEEVCVVPREMVQVLKA

EECILTNPKISRDINKLLSTNLMN

>AtbHLH082

MENGNGEGKGEFINQNNDFFLDSMSMLSSLPPCWDPSLPPPPPPPQSLFHALAVDAPFPD

QFHHPQESGGPTMGSQEGLQPQGTVSTTSAPVVRQKPRVRARRGQATDPHSIAERLRRER

IAERMKSLQELVPNTNKTDKASMLDEIIEYVRFLQLQVKVLSMSRLGGAGSVGPRLNGLS

AEAGGRLNALTAPCNGLNGNGNATGSSNESLRSTEQRVAKLMEEDMGSAMQYLQGKGLCL

MPISLATAISSSTTHSRGSLFNPISSAVAAEDSNVTATAVAAPEASSTMDDVSASKA

>AtbHLH167

MMNTYNMVKQEFIKKWINTLHMLDSSIEHPLNVTERKNAIRLSSDLAMAAARNGSTVWSR

ALISRSGNKTANKPMARRILKKARNRMKNRCNILRRNGNFTAKTWVRKRTDLLKSLVPGG

ELIDDKDYLIRETLDYIVYLRAQVDVMRTVAAVDLFTRNLTNDRRNK

>AtbHLH013

MNIGRLVWNEDDKAIVASLLGKRALDYLLSNSVSNANLLMTLGSDENLQNKLSDLVERPN

ASNFSWNYAIFWQISRSKAGDLVLCWGDGYCREPKEGEKSEIVRILSMGREEETHQTMRK

RVLQKLHDLFGGSEEENCALGLDRVTDTEMFLLSSMYFSFPRGEGGPGKCFASAKPVWLS

DVVNSGSDYCVRSFLAKSAGIQTVVLVPTDLGVVELGSTSCLPESEDSILSIRSLFTSSL

PPVRAVALPVTVAEKIDDNRTKIFGKDLHNSGFLQHHQHHQQQQQQPPQQQQHRQFREKL

TVRKMDDRAPKRLDAYPNNGNRFMFSNPGTNNNTLLSPTWVQPENYTRPINVKEVPSTDE

FKFLPLQQSSQRLLPPAQMQIDFSAASSRASENNSDGEGGGEWADAVGADESGNNRPRKR

GRRPANGRAEALNHVEAERQRREKLNQRFYALRSVVPNISKMDKASLLGDAVSYINELHA

KLKVMEAERERLGYSSNPPISLDSDINVQTSGEDVTVRINCPLESHPASRIFHAFEESKV

EVINSNLEVSQDTVLHTFVVKSEELTKEKLISALSREQTNSVQSRTSSGR

>AtbHLH007

MANNNNIPHDSISDPSPTDDFFEQILGLSNFSGSSGSGLSGIGGVGPPPMMLQLGSGNEG

NHNHMGAIGGGGPVGFHNQMFPLGLSLDQGKGHGFLKPDETGKRFQDDVLDNRCSSMKPI

FHGQPMSQPAPPMPHQQSTIRPRVRARRGQATDPHSIAERLRRERIAERIRSLQELVPTV

NKTDRAAMIDEIVDYVKFLRLQVKVLSMSRLGGAGAVAPLVTEMPLSSSVEVRLERDTSL

KKEQNFGNGFSCTDLWCKNFLQDETQAVWEKWSNDGTERQVAKLMEENVGAAMQLLQSKA

LCIMPISLAMAIYHSQPPDTSSSIVKPEMNPPP

>AtbHLH091

MYEESSCFDPNSMVDNNGGFCAAETTFTVSHQFQPPLGSTTNSFDDDLKLPTMDEFSVFP

SVISLPNSETQNQNISNNNHLINQMIQESNWGVSEDNSNFFMNTSHPNTTTTPIPDLLSL

LHLPRCSMSLPSSDIMAGSCFTYDPLFHLNLPPQPPLIPSNDYSGYLLGIDTNTTTQRDE

SNVGDENNNAQFDSGIIEFSKEIRRKGRGKRKNKPFTTERERRCHLNERYEALKLLIPSP

SKGDRASILQDGIDYINELRRRVSELKYLVERKRCGGRHKNNEVDDNNNNKNLDDHGNED

DDDDDENMEKKPESDVIDQCSSNNSLRCSWLQRKSKVTEVDVRIVDDEVTIKVVQKKKIN

CLLLVSKVLDQLQLDLHHVAGGQIGEHYSFLFNTKIYEGSTIYASAIANRVIEVVDKHYM

ASLPNSNY

>AtbHLH163

MSSRRSSRSRQSGSSRISDDQISDLVSKLQHLIPELRRRRSDKVSASKVLQETCNYIRNL

HREVDDLSDRLSELLASTDDNSAEAAIIRSLLNY

>AtbHLH011

MDQPMKPKTCSESDFADDSSASSSSSSGQNLRGAEMVVEVKKEAVCSQKAEREKLRRDKL

KEQFLELGNALDPNRPKSDKASVLTDTIQMLKDVMNQVDRLKAEYETLSQESRELIQEKS

ELREEKATLKSDIEILNAQYQHRIKTMVPWVPHYSYHIPFVAITQGQSSFIPYSASVNPL

TEQQASVQQHSSSSADASMKQDSKIKPLDLDLMMNSNHSGQGNDQKDDVRLKLELKIHAS

SLAQQVSDLFNSFANKLFHGLTRVYFHAGCFWKREESKLDNHCKLIE

>AtbHLH145

MGQDRGFGFPTQRLCSLSSLALSHLGKQDLNLVSKTCGDTTDMFSTRGSYQVSTQVSQSY

FDGYCGWVHGSSHLQQQFLPPQNQCMKQVPLQVDGVISKAEEQCSQKRFLVFDQSGDQTT

LLLASDIRKSFETLKQHACPDMKEELQRSNKDLFVCHGMQGNSEPDLKEDSEELNALLYS

EDESGYCSEEDEVTSADHSPSIVVSGREDQKTFLGSYGQPLNAKKRKILETSNESMRDAE

SSCGSCDNTRISFLKRSKLSSNKIGEEKIFETVSLLRSVVPGEELVDPILVIDRAIDYLK

SLKMEAKNREA

>AtbHLH077

MNMDKETEQTLNYLPLGQSDPFGNGNEGTIGDFLGRYCNNPQEISPLTLQSFSLNSQISE

NFPISGGIRFPPYPGQFGSDREFGSQPTTQESNKSSLLDPDSVSDRVHTTKSNSRKRKSI

PSGNGKESPASSSLTASNSKVSGENGGSKGGKRSKQDVAGSSKNGVEKCDSKGDNKDDAK

PPEAPKDYIHVRARRGQATDSHSLAERARREKISERMTLLQDLVPGCNRITGKAVMLDEI

INYVQSLQRQVEFLSMKLATVNPRMEFNANASLSTEMIQPGESLTQSLYAMACSEQRLPS

AYYSLGKNMPRFSDTQFPSNDGFVHTETPGFWENNDLQSIVQMGFGDILQQQSNNNNNNC

SEPTLQMKLEP

>AtbHLH028

MINTDDNLLMIEALLTSDPSPPLLPANLSLETTLPKRLHAVLNGTHEPWSYAIFWKPSYD

DFSGEAVLKWGDGVYTGGNEEKTRGRLRRKKTILSSPEEKERRSNVIRELNLMISGEAFP

VVEDDVSDDDDVEVTDMEWFFLVSMTWSFGNGSGLAGKAFASYNPVLVTGSDLIYGSGCD

RAKQGGDVGLQTILCIPSHNGVLELASTEEIRPNSDLFNRIRFLFGGSKYFSGAPNSNSE

LFPFQLESSCSSTVTGNPNPSPVYLQNRYNLNFSTSSSTLARAPCGDVLSFGENVKQSFE

NRNPNTYSDQIQNVVPHATVMLEKKKGKKRGRKPAHGRDKPLNHVEAERMRREKLNHRFY

ALRAVVPNVSKMDKTSLLEDAVCYINELKSKAENVELEKHAIEIQFNELKEIAGQRNAIP

SVCKYEEKASEMMKIEVKIMESDDAMVRVESRKDHHPGARLMNALMDLELEVNHASISVM

NDLMIQQANVKMGLRIYKQEELRDLLMSKIS

>AtbHLH024

MISQREEREEKKQRVMGDKKLISSSSSSSVYDTRINHHLHHPPSSSDEISQFLRHIFDRS

SPLPSYYSPATTTTTASLIGVHGSGDPHADNSRSLVSHHPPSDSVLMSKRVGDFSEVLIG

GGSGSAAACFGFSGGGNNNNVQGNSSGTRVSSSSVGASGNETDEYDCESEEGGEAVVDEA

PSSKSGPSSRSSSKRCRAAEVHNLSEKRRRSRINEKMKALQSLIPNSNKTDKASMLDEAI

EYLKQLQLQVQMLTMRNGINLHPLCLPGTTLHPLQLSQIRPPEATNDPLLNHTNQFASTS

NAPEMINTVASSYALEPSIRSHFGPFPLLTSPVEMSREGGLTHPRLNIGHSNANITGEQA

LFDGQPDLKDRIT

>AtbHLH033

MNSDGVWLDGSGESPEVNNGEAASWVRNPDEDWFNNPPPPQHTNQNDFRFNGGFPLNPSE

NLLLLLQQSIDSSSSSSPLLHPFTLDAASQQQQQQQQQQEQSFLATKACIVSLLNVPTIN

NNTFDDFGFDSGFLGQQFHGNHQSPNSMNFTGLNHSVPDFLPAPENSSGSCGLSPLFSNR

AKVLKPLQVMASSGSQPTLFQKRAAMRQSSSSKMCNSESSSEMRKSSYEREIDDTSTGII

DISGLNYESDDHNTNNNKGKKKGMPAKNLMAERRRRKKLNDRLYMLRSVVPKISKMDRAS

ILGDAIDYLKELLQRINDLHTELESTPPSSSSLHPLTPTPQTLSYRVKEELCPSSSLPSP

KGQQPRVEVRLREGKAVNIHMFCGRRPGLLLSTMRALDNLGLDVQQAVISCFNGFALDVF

RAEQCQEDHDVLPEQIKAVLLDTAGYAGLV

>AtbHLH060

MDLTGGFGARSGGVGPCREPIGLESLHLGDEFRQLVTTLPPENPGGSFTALLELPPTQAV

ELLHFTDSSSSQQAAVTGIGGEIPPPLHSFGGTLAFPSNSVLMERAARFSVIATEQQNGN

ISGETPTSSVPSNSSANLDRVKTEPAETDSSQRLISDSAIENQIPCPNQNNRNGKRKDFE

KKGKSSTKKNKSSEENEKLPYVHVRARRGQATDSHSLAERARREKINARMKLLQELVPGC

DKGTDFGGKIKIKVCFGVHLLMISGKKVAIFLWKVSCEDLIDCSFSPPRIQGTALVLDEI

INHVQSLQRQVEMLSMRLAAVNPRIDFNLDTILASENGSLMDGSFNAAPMQLAWPQQAIE

TEQSFHHRQLQQPPTQQWPFDGLNQPVWGREEDQAHGNDNSNLMAVSENVMVASANLHPN

QVKMEL

>AtbHLH056

MSSWLHHSHPGVTSTPASSVSLPPPPNAPREDDIVELLWQSGQVVGTNQTHRQSYDPPPI

LRGSGSGRGEENAPLSQPPPHLHQQNLFIQEGEMYSWLHHSYRQNYFCSELLNSTPATHP

QSSISLAPRQTIATRRAENFMNFSWLRGNIFTGGRVDEAGPSFSVVRESMQVGSNTTPPS

SSATESCVIPATEGTASRVSGTLAAHDLGRKGKAVAVEAAGTPSSGVCKAETEPVQIQPA

TESKLKAREETHGTEEARGSTSRKRSRTAEMHNLAERRRREKINEKMKTLQQLIPRCNKS

TKVSTLDDAIEYVKSLQSQIQGMMSPMMNAGNTQQFMPHMAMDMNRPPPFIPFPGTSFPM

PAQMAGVGPSYPAPRYPFPNIQTFDPSRVRLPSPQPNPVSNQPQFPAYMNPYSQFAGPHQ

LQQPPPPPFQGQTTSQLSSGQASSSKEPEDQENQPTA

>AtbHLH087

MEGLESVYAQAMYGMTRESKIMEHQGSDLIWGGNELMARELCSSSSYHHQLINPNLSSCF

MSDLGVLGEIQQQQHVGNRASSIDPSSLDCLLSATSNSNNTSTEDDEGISVLFSDCQTLW

SFGGVSSAESENREITTETTTTIKPKPLKRNRGGDGGTTETTTTTTKPKSLKRNRGDETG

SHFSLVHPQDDSEKGGFKLIYDENQSKSKKPRTEKERGGSSNISFQHSTCLSDNVEPDAE

AIAQMKEMIYRAAAFRPVNFGLEIVEKPKRKNVKISTDPQTVAARQRRERISEKIRVLQT

LVPGGTKMDTASMLDEAANYLKFLRAQVKALENLRPKLDQTNLSFSSAPTSFPLFHPSFL

PLQNPNQIHHPEC

>AtbHLH096

MALEAVVYPQDPFSYISCKDFPFYDLYFQEEEDQDPQDTKNNIKLGQGQGHGFASNNYNG

RTGDYSDDYNYNEEDLQWPRDLPYGSAVDTESQPPPSDVAAGGGRRKRRRTRSSKNKEEI

ENQRMTHIAVERNRRKQMNEYLAVLRSLMPPYYAQRGDQASIVGGAINYLKELEHHLQSM

EPPVKTATEDTGAGHDQTKTTSASSSGPFSDFFAFPQYSNRPTSAAAAEGMAEIEVTMVE

SHASLKILAKKRPRQLLKLVSSIQSLRLTLLHLNVTTRDDSVLYSISVKVEEGSQLNTVE

DIAAAVNQILRRIEEESSFS

>AtbHLH026

MSNNQAFMELGWRNDVGSLAVKDQGMMSERARSDEDRLINGLKWGYGYFDHDQTDNYLQI

VPEIHKEVENAKEDLLVVVPDEHSETDDHHHIKDFSERSDHRFYLRNKHENPKKRRIQVL

SSDDESEEFTREVPSVTRKGSKRRRRDEKMSNKMRKLQQLVPNCHKTDKVSVLDKTIEYM

KNLQLQLQMMSTVGVNPYFLPATLGFGMHNHMLTAMASAHGLNPANHMMPSPLIPALNWP

LPPFTNISFPHSSSQSLFLTTSSPASSPQSLHGLVPYFPSFLDFSSHAMRRL

>AtbHLH019

MDEDFFLPDFSLVDIDFDFNIYEENNLSPDESLSNSRRADQSSKFDHQMHFECLREKPKA

AVKPMMKINNKQQLISFDFSSNVISSPAAEEIIMDKLVGRGTKRKTCSHGTRSPVLAKEH

VLAERKRREKLSEKFIALSALLPGLKKADKVTILDDAISRMKQLQEQLRTLKEEKEATRQ

MESMILVKKSKVFFDEEPNLSCSPSVHIEFDQALPEIEAKISQNDILIRILCEKSKGCMI

NILNTIENFQLRIENSIVLPFGDSTLDITVLAQMDKDFSMSILKDLVRNLRLAMV

>AtbHLH113

MGDTAEDQDDRAMMEAEGVTSFSELLMFSDGVLSSSSDHQPEGNVGDGGEDSLGFVFSGK

TGSRMLCFSGGYQNDDESLFLEPSVPTSGVSDLDPSCIKIDCRNSNDACTVDKSTKSSTK

KRTGTGNGQESDQNRKPGKKGKRNQEKSSVGIAKVRKERLGERIAALQQLVSPYGKTDAA

SVLHEAMGYIKFLQDQIQVLCSPYLINHSLDGGVVTGDVMAAMKAKDLRSRGLCLVPVSS

TVHVENSNGADFWSPATMGHTTSPSLPQGF

>AtbHLH078

MSDKDEFAAKKKDLVNTPVDLYPPENPMLGPSPMMDSFRETLWHDGGFNVHTDADTSFRG

NNNIDIPLEMGWNMAQFPADSGFIERAAKFSFFGCGEMMMNQQQSSLGVPDSTGLFLQDT

QIPSGSKLDNGPLTDASKLVKERSINNVSEDSQSSGGNGHDDAKCGQTSSKGFSSKKRKR

IGKDCEEEEDKKQKDEQSPTSNANKTNSEKQPSDSLKDGYIHMRARRGQATNSHSLAERV

RREKISERMKFLQDLVPGCDKVTGKAVMLDEIINYVQSLQCQIEFLSMKLSAVNPVLDFN

LESLLAKDALQSSAPTFPHNMSMLYPPVSYLSQTGFMQPNISSMSLLSGGLKRQETHGYE

SDHHNLVHMNHETGTAPDHEDTTADMKVEP

>AtbHLH021

MESNMQNLLEKLRPLVGARAWDYCVLWRLNEDQRFVKWMGCCCGGTELIAENGTEEFSYG

GCRDVMFHHPRTKSCEFLSHLPASIPLDSGIYAETLLTNQTGWLSESSEPSFMQETICTR

VLIPIPGGLVELFATRHVAEDQNVVDFVMGHCNMLMDDSVTINMMVADEVESKPYGMLSG

DIQQKGSKEEDMMNLPSSYDISADQIRLNFLPQMSDYETQHLKMKSDYHHQALGYLPENG

NKEMMGMNPFNTVEEDGIPVIGEPSLLVNEQQVVNDKDMNENGRVDSGSDCSDQIDDEDD

PKYKKKSGKGSQAKNLMAERRRRKKLNDRLYALRSLVPRITKLDRASILGDAINYVKELQ

NEAKELQDELEENSETEDGSNRPQGGMSLNGTVVTGFHPGLSCNSNVPSVKQDVDLENSN

DKGQEMEPQVDVAQLDGREFFVKVICEYKPGGFTRLMEALDSLGLEVTNANTTRYLSLVS

NVFKVEKNDNEMVQAEHVRNSLLEITRNTSRGWQDDQMATGSMQNEKNEVDYQHYDDHQH

HNGHHHPFDHQMNQSAHHHHHHQHINHYHNQ

>AtbHLH148

MASLISDIEPPTSTTSDLVRRKKRSSASSAASSRSSASSVSGEIHARWRSEKQQRIYSAK

LFQALQQVRLNSSASTSSSPTAQKRGKAVREAADRALAVSARGRTLWSRAILANRIKLKF

RKQRRPRATMAIPAMTTVVSSSSNRSRKRRVSVLRLNKKSIPDVNRKVRVLGRLVPGCGK

QSVPVILEEATDYIQALEMQVRAMNSLVQLLSSYGSAPPPI

>AtbHLH102

MRTGKGNQEEEDYGEEDFNSKREGPSSNTTVHSNRDSKENDKASAIRSKHSVTEQRRRSK

INERFQILRELIPNSEQKRDTASFLLEVIDYVQYLQEKVQKYEGSYPGWSQEPTKLTPWR

NNHWRVQSLGNHPVAINNGSGPGIPFPGKFEDNTVTSTPAIIAEPQIPIESDKARAITGI

SIESQPELDDKGLPPLQPILPMVQGEQANECPATSDGLGQSNDLVIEGGTISISSAYSHE

LLSSLTQALQNAGIDLSQAKLSVQIDLGKRANQGLTHEEPSSKNPLSYDTQGRDSSVEEE

SEHSHKRMKTL

>AtbHLH022

MGGGSRFQEPVRMSRRKQVTKEKEEDENFKSPNLEAERRRREKLHCRLMALRSHVPIVTN

MTKASIVEDAITYIGELQNNVKNLLETFHEMEEAPPEIDEEQTDPMIKPEVETSDLNEEM

KKLGIEENVQLCKIGERKFWLKIITEKRDGIFTKFMEVMRFLGFEIIDISLTTSNGAILI

SASVQTQELCDVEQTKDFLLEVMRSNP

>AtbHLH168

MRTLKTQTTRGRRRANVSSRTRVLHTCCGNGSSDGGKTVMEKLLALKSLLPPPVNVGGGE

TEELFQETAEYIVKLRTQVVVLKKLIEIYDNSSDQKKDVVL

>AtbHLH079

MDPPLVNDSSFSAANPSSYTLSEIWPFPVNDAVRSGLRLAVNSGRVFTRSEHSGNKDVSA

AEESTVTDLTAGWGSRKTRDLNSEDDSSKMVSSSSSGNELKESGDKKRKLCGSESGNGDG

SMRPEGETSSGGGGSKATEQKNKPEPPKDYIHVRARRGQATDRHSLAERARREKISEKMT

ALQDIIPGCNKIIGKALVLDEIINYIQSLQRQVEFLSMKLEVVNSGASTGPTIGVFPSGD

LGTLPIDVHRTIYEQQEANETRVSQPEWLHMQVDGNFNRTT

>AtbHLH140

MDDFNLRSENPNSSSTTSSSSSSFHRHKSETGNTKRSRSTSTLSTDPQSVAARDRRHRIS

DRFKILQSMVPGGAKMDTVSMLDEAISYVKFLKAQIWYHQNMLLFINDHETTSSCTYSPG

AGEFGPKLFGYDDDYAPIMDTYSQGVPLTVADSKYTPWFGSVDDEQERDSS

>AtbHLH162

MEPSHSNTGQSRSVDRKTVEKNRRMQMKSLYSELISLLPHHSSTEPLTLPDQLDEAANYI

KKLQVNVEKKRERKRNLVATTTLEKLNSVGSSSVSSSVDVSVPRKLPKIEIQETGSIFHI

FLVTSLEHKFMFCEIIRVLTEELGAEITHAGYSIVDDAVFHTLHCKVEEHDYGARSQIPE

RLEKIVNSVH

>AtbHLH068

MNRGVLESSPVQQLMAAGNPNWWNVSGGMRPPPPLMGHQQAPLPPHMTPNNNYLRPRMMP

TPFPHFLPSPATSSSSSSSSPSLPNNPNLSSWLESNDLPPESWSLSQLLLGGLMMGEEER

LEMMNHHNHHDEQQHHGFQGKIRLENWEEQVLSHQQASMVAVDIKQEGNINNNNGYVISS

PNSPPNKSCVTTTTTTSLNSNDDNINNNNNMLDFSSNHNGLHLSEGRHTPPDRSSECNSL

EIGGSTNKKPRLQPSPSSQSTLKVRKEKLGGRIAALHQLVSPFGKTDTASVLSEAIGYIR

FLQSQIEALSHPYFGTTASGNMRHQQHLQGDRSCIFPEDPGQLVNDQCMKRRGASSSSTD

NQNASEEPKKDLRSRGLCLVPISCTLQVGSDNGADYWAPALGSAGFH

>AtbHLH003

MGQKFWENQEDRAMVESTIGSEACDFFISTASASNTALSKLVSPPSDSNLQQGLRHVVEG

SDWDYALFWLASNVNSSDGCVLIWGDGHCRVKKGASGEDYSQQDEIKRRVLRKLHLSFVG

SDEDHRLVKSGALTDLDMFYLASLYFSFRCDTNKYGPAGTYVSGKPLWAADLPSCLSYYR

VRSFLARSAGFQTVLSVPVNSGVVELGSLRHIPEDKSVIEMVKSVFGGSDFVQAKEAPKI

FGRQLSLGGAKPRSMSINFSPKTEDDTGFSLESYEVQAIGGSNQVYGYEQGKDETLYLTD

EQKPRKRGRKPANGREEALNHVEAERQRREKLNQRFYALRAVVPNISKMDKASLLADAIT

YITDMQKKIRVYETEKQIMKRRESNQITPAEVDYQQRHDDAVVRLSCPLETHPVSKVIQT

LRENEVMPHDSNVAITEEGVVHTFTLRPQGGCTAEQLKDKLLASLSQ

>AtbHLH133

MNRGVLESSPVQHLTAAGNPNWWNNVSRGLRPPTPLMSHEPPSTTAFIPSLLPNFFSSPT

SSSSSSPSFPPPNSNPNFSSWLEMSDLPLDQPWSLSQLLLGGLMMGEEEKMEMMNHHHHQ

NQHQSYQAKRIQNWEEQVLRHQASMKQESSNNNSYGIMSSPNSPPNKSCATIINTNEDNN

NNIHSGLNLSECNSSEMIGSSFANKKPKLQVPSSQSTLKVRKEKLGGRIASLHQLVSPFG

KTDTASVLSEAIGYIRFLHSQIEALSLPYFGTPSRNNMMHQHAQRNMNGIFPEDPGQADP

PQKNLGCCEVGQPVLSPILPLDHTFNIILNIYAEEIFSDLVNQLVNEYCMKRGVSLSSTD

NQKSNPNEEPMKDLRSRGLCLVPISCTLQVGSDNGADYWAPAFGTTLQ

>AtbHLH031

MDPSGMMNEGGPFNLAEIWQFPLNGVSTAGDSSRRSFVGPNQFGDADLTTAANGDPARMS

HALSQAVIEGISGAWKRREDESKSAKIVSTIGASEGENKRQKIDEVCDGKAEAESLGTET

EQKKQQMEPTKDYIHVRARRGQATDSHSLAERARREKISERMKILQDLVPGCNKVIGKAL

VLDEIINYIQSLQRQVEFLSMKLEAVNSRMNPGIEVFPPKEVMILMIINSIFSIFFTKQY

MFLSRYSRGRSLDVYAVRSFKHCNKRSDLCFCSCSPKTELKTTIFSQNMTCFCRYSRVGV

AISSSKHCNEPVTLCFYSYCLRKIYHFLLWNLKYKIQKSVLFS

>AtbHLH075

MARFEPYNYNNGHDPFFAHINQNPELINLDLPASTPSSFMLFSNGALVDANHNNSHFFPN

LLHGNTRRKGNKEESGSKRRRKRSEEEEAMNGDETQKPKDVVHVRAKRGQATDSHSLAER

VRREKINERLKCLQDLVPGCYKAMGMAVMLDVIIDYVRSLQNQIEFLSMKLSAASACYDL

NSLDIEPTDIFQGGNIHSAAEMERILRESVGTQPPNFSSTLPF

>AtbHLH041

MDAFFLTDDPNTRNQLIRSLAQSFGCVYVCLWSYYFPRPSNYLISMDGYYNEASEEPSSS

SSSGSLARSLFHEYRQSVIPLQNGHVPSMAFMNNLPYVEIRPQESQRLAFNDTQRLFYQE

ARIQTVIFMGCRSGEIELGMTYDTTNMKIEASLREWFPEDFNRKSSPANSDYLRPPHYPS

SSSSSLSPNNISEYSSLLFPLIPKPSTTTEAVNVPVLPPLAPINMIHPQHQEPLFRNRQR

EEEAMTQAILAVLTGPSSPPSTSSSPQRKGRATAFKRYYSMISDRGRAPLPSVRKQSMMT

RAMSFYNRLNINQRERFTRENATTHGEGSGGSGGGGRYTSGPSATQLQHMISERKRREKL

NESFQALRSLLPPGTKKDKASVLSIAREQLSSLQGEISKLLERNREVEAKLAGEREIEND

LRPEERFNVRIRHIPESTSRERTLDLRVVLRGDIIRVDDLMIRLLEFLKQINNVSLVSIE

ARTLARAEGDTSIVLVISLRLKIEGEWDESAFQEAVRRVVADLAH

>AtbHLH062

MENELFMNAGVSHPPVMTSPSSSSAMLKWVSMETQPVDPSLSRNLFWEKSTEQSIFDSAL

SSLVSSPTPSNSNFSVGGVGGENVIMRELIGKLGNIGDIYGITASNGNSCYATPMSSPPP

GSMMETKTTTPMAELSGDPGFAERAARFSCFGSRSFNSRTNSPFPINNEPPITTNEKMPR

VSSSPVFKPLASHVPAGESSGELSRKRKTKSKQNSPSAVSSSKEIEEKEDSDPKRCKKSE

ENGDKTKSIDPYKDYIHVRARRGQATDSHSLAERVRREKISERMKLLQDLVPGCNKVTGK

ALMLDEIINYVQSLQRQVEFLSMKLSSVNTRLDFNMDALLSKDIFPSSNNLMHHQQVLQL

DSSAETLLGDHHNKNLQLNPDISSNNVINPLETSETRSFISHLPTLAHFTDSISQYSTFS

EDDLHSIIHMGFAQNRLQELNQGSSNQVPSHMKAEL

>AtbHLH035

MEDIVDQELSNYWEPSSFLQNEDFEYDRSWPLEEAISGSYDSSSPDGAASSPASKNIVSE

RNRRQKLNQRLFALRSVVPNITKMDKASIIKDAISYIEGLQYEEKKLEAEIRELESTPKS

SLSFSKDFDRDLLVPVTSKKMKQLDSGSSTSLIEVLELKVTFMGERTMVVSVTCNKRTDT

MVKLCEVFESLNLKILTSNLTSFSGMIFHTVFIELRPNIYWVVWFLVFMSIFGPTIIVIW

SIWFIKKKIILSLWRMKKNKRCCG

>AtbHLH125

MDCVPSLFMPDSTYEDGLLFSDSFLLSPFISYQNNDVFHSITNKIGGSNKKRSLCDITYG

ANEANKNDDDRESKKMKHRDIERQRRQEVSSLFKRLRTLLPFQYIQGKRSTSDHIVQAVN

YIKDLQIKIKELNEKRNRVKKVISATTTTHSAIEECTSSLSSSAASTLSSSCSCVGDKHI

TVVVTPCLVGVEIIISCCLGRNKSCLSSVLQMLAQEQRFSVVSCLSARRQQRFMHTIVSQ

VEDGKQINILELKDKIMTM

>AtbHLH092

MDNFFLGLSCQEENNFWDLIVADISGDRSVSVPIRSAFRSYMKDTELRMMSPKISSSKVN

VKKRMVNLLRKNWEEKKNTVAPEKERSRRHMLKERTRREKQKQSYLALHSLLPFATKNDK

NSIVEKAVDEIAKLQRLKKELVRRIKVIEEKSAKDGHDEMSETKVRVNLKEPLSGLDSML

EALHYLKSMGTKLKTVHANFSPQEFSATMTIETQIRGEEVEKRVERRLQETEWKLLFLPE

ASFYKDY

>AtbHLH149

MVESLFPSIENTGESSRRKKPRISETAEAEIEARRVNEESLKRWKTNRVQQIYACKLVEA

LRRVRQRSSTTSNNETDKLVSGAAREIRDTADRVLAASARGTTRWSRAILASRVRAKLKK

HRKAKKSTGNCKSRKGLTETNRIKLPAVERKLKILGRLVPGCRKVSVPNLLDEATDYIAA

LEMQVRAMEALAELLTAAAPRTTLTGT

>AtbHLH036

MDDCRDKRRRRCTKLTCGTDNNDMEKMMHRETERQRRQEMASLYASLRSLLPLHFIKGKR

STSDQVNEAVNYIKYLQRKIKELSVRRDDLMVLSRGSLLGSSNGDFKEDVEMISGKNHVV

VRQCLVGVEIMLSSRCCGGQPRFSSVLQVLSEYGLCLLNSISSIVDDRLVYTIQAEVNDM

ALMIDLAELEKRLIRMK

>AtbHLH030

MCAKKEEEEEEEEDSSEAMNNIQNYQNDLFFHQLISHHHHHHHDPSQSETLGASGNVGSG

FTIFSQDSVSPIWSLPPPTSIQPPFDQFPPPSSSPASFYGSFFNRSRAHHQGLQFGYEGF

GGATSAAHHHHEQLRILSEALGPVVQAGSGPFGLQAELGKMTAQEIMDAKALAASKSHSE

AERRRRERINNHLAKLRSILPNTTKTDKASLLAEVIQHVKELKRETSVISETNLVPTESD

ELTVAFTEEEETGDGRFVIKASLCCEDRSDLLPDMIKTLKAMRLKTLKAEITTVGGRVKN

VLFVTGEESSGEEVEEEYCIGTIEEALKAVMEKSNVEESSSSGNAKRQRMSSHNTITIVE

QQQQYNQR

>AtbHLH006

MTDYRLQPTMNLWTTDDNASMMEAFMSSSDISTLWPPASTTTTTATTETTPTPAMEIPAQ

AGFNQETLQQRLQALIEGTHEGWTYAIFWQPSYDFSGASVLGWGDGYYKGEEDKANPRRR

SSSPPFSTPADQEYRKKVLRELNSLISGGVAPSDDAVDEEVTDTEWFFLVSMTQSFACGA

GLAGKAFATGNAVWVSGSDQLSGSGCERAKQGGVFGMHTIACIPSANGVVEVGSTEPIRQ

SSDLINKVRILFNFDGGAGDLSGLNWNLDPDQGENDPSMWINDPIGTPGSNEPGNGAPSS

SSQLFSKSIQFENGSSSTITENPNLDPTPSPVHSQTQNPKFNNTFSRELNFSTSSSTLVK

PRSGEILNFGDEGKRSSGNPDPSSYSGQTQFENKRKRSMVLNEDKVLSFGDKTAGESDHS

DLEASVVKEVAVEKRPKKRGRKPANGREEPLNHVEAERQRREKLNQRFYALRAVVPNVSK

MDKASLLGDAIAYINELKSKVVKTESEKLQIKNQLEEVKLELAGRKASASGGDMSSSCSS

IKPVGMEIEVKIIGWDAMIRVESSKRNHPAARLMSALMDLELEVNHASMSVVNDLMIQQA

TVKMGFRIYTQEQLRASLISKIG

>AtbHLH080

MQSTHISGGSSGGGGGGGGEVSRSGLSRIRSAPATWIETLLEEDEEEGLKPNLCLTELLT

GNNNSGGVITSRDDSFEFLSSVEQGLYNHHQGGGFHRQNSSPADFLSGSGSGTDGYFSNF

GIPANYDYLSTNVDISPTKRSRDMETQFSSQLKEEQMSGGISGMMDMNMDKIFEDSVPCR

VRAKRGCATHPRSIAERVRRTRISDRIRRLQELVPNMDKQTNTADMLEEAVEYVKALQSQ

IQELTEQQKRCKCKPKEEQ

>AtbHLH105

MVSPENANWICDLIDADYGSFTIQGPGFSWPVQQPIGVSSNSSAGVDGSAGNSEASKEPG

SKKRGRCESSSATSSKACREKQRRDRLNDKFMELGAILEPGNPPKTDKAAILVDAVRMVT

QLRGEAQKLKDSNSSLQDKIKELKTEKNELRDEKQRLKTEKEKLEQQLKAMNAPQPSFFP

APPMMPTAFASAQGQAPGNKMVPIISYPGVAMWQFMPPASVDTSQDHVLRPPVA

>AtbHLH120

MKTTPLPRLHYLVSLLCFFLSSKIKEDRPNYVRAVSPINLTSSLEKTREKKKRLLLRSTI

SKPQPMNPSNNPKKTRHQSHMPQERDETKKEKKLLHRNIERQRRQEMAILFASLRSQLPL

KYIKGKRAMSDHVNGAVSFIKDTQTRIKDLSARRDELKREIGDPTSLTGSGSGSGSSRSE

PASVMVQPCVSGFEVVVSSLASGLEAWPLSRVLEVLHGQGLEVISSLTARVNERLMYTIQ

VEVNSFDCFDLAWLQQKLIEQLVLSTTRH

>AtbHLH064

MLEGLVSQESLSLNSMDMSVLERLKWVQQQQQQLQQVVSHSSNNSPELLQILQFHGSNND

ELLESSFSQFQMLGSGFGPNYNMGFGPPHESISRTSSCHMEPVDTMEVLLKTGEETRAVA

LKNKRKPEVKTREEQKTEKKIKVEAETESSMKGKSNMGNTEASSDTSKETSKGASENQKL

DYIHVRARRGQATDRHSLAERARREKISKKMKYLQDIVPGCNKVTGKAGMLDEIINYVQC

LQRQVEFLSMKLAVLNPELELAVEDVSVKQFQAYFTNVVASKQSIMVDVPLFPLDQQGSL

DLSAINPNQTTSIEAPSGSWETQSQSLYNTSSLENSCGNYNKISKILLSTKCTHQYVPIR

RVWV

>AtbHLH112

MAEEFKATASICGGGGGAWWNSPRSVMSPSDHFLSPCFGAAITSNDFSSQENHLKSRMTC

TDNNNIVFGQREADSDSGGSTVTMDSTLQMMGLGFSSNCSSDWNQTILQEDLNSSFIRSS

QDQDHGQGFLSTTTSPYILNPACSSSPSTSSSSSLIRTFYDPEPSPYNFVSTTSGSINDP

QLSWANKTNPHHQVAYGLINSFSNNANSRPFWNSSSTTNLNNTTPSNFVTTPQIISTRLE

DKTKNLKTRAQSESLKRAKDNESAAKKPRVTTPSPLPTFKVRKENLRDQITSLQQLVSPF

GKTDTASVLQEAIEYIKFLHDQVTVLSTPYMKQGASNQQQQQISGKSKSQDENENHELRG

HGLCLVPISSTFPVANETTADFWTPTFGGNNFR

>AtbHLH170

MERQIINKRKRVFSLQPNKNPKAVFARRYVSHLVPALKKINMNKSSSKTNKQSLEQTVKH

EVDMAFALSAQEFAWSRFLQQKLLSSPYDDPISTSSSPSEILERSSKRQGGEKHQDSDEE

EEGGEIKKRLKELQKLLPGGEEMNMEEILSEIGSYIVCLELQMIVLKSIVQDNTS

>AtbHLH101

MCTLTPMFPSKQQEWYSASTMEYPWLQSQVHSFSPTLHFPSFLHPLDDSKSHNINLHHMS

LSHSNNTNSNNNNYQEEDRGAVVLEKKLNHNASERDRRRKLNALYSSLRALLPLSDQKRK

LSIPMTVARVVKYIPEQKQELQRLSRRKEELLKRISRKTHQEQLRNKAMMDSIDSSSSQR

IAANWLTDTEIAVQIATSKWTSVSDMLLRLEENGLNVISVSSSVSSTARIFYTLHLQMRG

DCKVRLEELINGMLLGLRQS

>AtbHLH085

MEAMGEWSNNLGGMYTYATEEADFMNQLLASYDHPGTGSSSGAAASGDHQGLYWNLGSHH

NHLSLVSEAGSFCFSQESSSYSAGNSGYYTVVPPTVEENQNETMDFGMEDVTINTNSYLV

GEETSECDVEKYSSGKTLMPLETVVENHDDEESLLQSEISVTTTKSLTGSKKRSRATSTD

KNKRARVNKRAQKNVEMSGDNNEGEEEEGETKLKKRKNGAMMSRQNSSTTFCTEEESNCA

DQDGGGEDSSSKEDDPSKALNLNGKTRASRGAATDPQSLYARKRRERINERLRILQNLVP

NGTKVDISTMLEEAVHYVKFLQLQIKLLSSDDLWMYAPIAFNGMDIGLSSPR

>AtbHLH107

MQPEVSDQIFYAFLTGGLCASSTSTTVTSSSDPFATVYEDKALASLRNHKEAERKRRARI

NSHLNKLRKLLSCNSKTDKSTLLAKVVQRVKELKQQTLEITDETIPSETDEISVLNIEDC

SRGDDRRIIFKVSFCCEDRPELLKDLMETLKSLQMETLFADMTTVGGRTRNVLVVAADKE

HHGVQSVNFLQNALKSLLERSSKSVMVGHGGGGGEERLKRRRALDHIIMV

>AtbHLH072

MSNYGVKELTWENGQLTVHGLGDEVEPTTSNNPIWTQSLNGCETLESVVHQAALQQPSKF

QLQSPNGPNHNYESKDGSCSRKRGYPQEMDRWFAVQEESHRVGHSVTASASGTNMSWASF

ESGRSLKTARTGDRDYFRSGSETQDTEGDEQETRGEAGRSNGRRGRAAAIHNESERRRRD

RINQRMRTLQKLLPTASKADKVSILDDVIEHLKQLQAQVQFMSLRANLPQQMMIPQLPPP

QSVLSIQHQQQQQQQQQQQQQQQQQFQMSLLATMARMGMGGGGNGYGGLVPPPPPPPMMV

PPMGNRDCTNGSSATLSDPYSAFFAQTMNMDLYNKMAAAIYRQQSDQTTKVNIGMPSSSS

NHEKRD

>AtbHLH012

MSLTMADGVEAAAGRSKRQNSLLRKQLALAVRSVQWSYAIFWSSSLTQPGVLEWGEGCYN

GDMKKRKKSYESHYKYGLQKSKELRKLYLSMLEGDSGTTVSTTHDNLNDDDDNCHSTSMM

LSPDDLSDEEWYYLVSMSYVFSPSQCLPGRASATGETIWLCNAQYAENKLFSRSLLARSA

SIQTVVCFPYLGGVIELGVTELISEDHNLLRNIKSCLMEISAHQDNDDEKKMEIKISEEK

HQLPLGISDEDLHYKRTISTVLNYSADRSGKNDKNIRHRQPNIVTSEPGSSFLRWKQCEQ

QVSGFVQKKKSQNVLRKILHDVPLMHTKRMFPSQNSGLNQDDPSDRRKENEKFSVLRTMV

PTVNEVDKESILNNTIKYLQELEARVEELESCMGSVNFVERQRKTTENLNDSVLIEETSG

NYDDSTKIDDNSGETEQVTVFRDKTHLRVKLKETEVVIEVRCSYRDYIVADIMETLSNLH

MDAFSVRSHTLNKFLTLNLKAKFRGAAVASVGMIKRELRRVIDFREPICDVPLSLHQVFR

VFVCKVCQSLVGIFDNVVSSSSTKPRSILIHNSWAICIFH

>AtbHLH057

MSGLMSFGELEDQFGQISDTTMEEKIPFLQMLQCIEHPFTTTEPNQFLQSLLQIQTLESK

SCLTLETNIKRDPGQTDDPEKDPRTENGAVTVKEKRKRKRTRAPKNKDEVENQRMTHIAV

ERNRRRQMNEHLNSLRSLMPPSFLQRGDQASIVGGAIDFIKELEQLLQSLEAEKRKDGTD

ETPKTASCSSSSSLACTNSSISSVSTTSENGFTARFGGGDTTEVEATVIQNHVSLKVRCK

RGKRQILKAIVSIEELKLAILHLTISSSFDFVIYSFNLKMEDGCKLGSADEIATAVHQIF

EQINGEVMWSNLSRT

>AtbHLH151

MGVTLEGQRKESIWVLMRRQRARRALVKKIMIRPRKSVEASRRPCRAIHRRVKTLKELVP

NTKTSEGLDGLFRQTADYILALEMKVKVMQTMVQVLTETNCV

>AtbHLH173

MGYTLQQILRSICSNTDWNYAVFWKLNHHSPMVLTLEDVYCVNHERGLMPESLHGGRHAH

DPLGLAVAKMSYHVHSLGEGIVGQVAISGQHQWIFSEYLNDSHSTLQVHNGWESQISAGI

KTILIVAVGSCGVVQLGSLCKVEEDPALVTHIRHLFLALTDPLADHASNLMQCDINSPSD

RPKIPSKCLHEASPDFSGEFDKAMDMEGLNIVSQNTSNRSNDLPYNFTPTYFHMERTAQV

IGGLEAVQPSMFGSNDCVTSGFSVGVVDTKHKNQVDISDMSKVIYDEETGGYRYSRELDP

NFQHYSRNHVRNSGGTSALAMESDRLKAGSSYPQLDSTVLTALKTDKDYSRRNEVFQPSE

SQGSIFVKDTEHRQEEKSESSQLDALTASLCSFSGSELLEALGPAFSKTSTDYGELAKFE

SAAAIRRTNDMSHSHLTFESSSENLLDAVVASMSNGDGNVRREISSSRSTQSLLTTAEMA

QAEPFGHNKQNIVSTVDSVISQPPLADGLIQQNPSNICGAFSSIGFSSTCLSSSSDQFPT

SLEIPKKNKKRAKPGESSRPRPRDRQLIQDRIKELRELVPNGSKCSIDSLLECTIKHMLF

LQSVSQHADKLTKSASSKLQMQHKDTGTLGISSTEQGSSWAVEIGGHLQVCSIMVENLDK

EGVMLIEMLCEECSHFLEIANVIRSLELIILRGTTEKQGEKTWICFVVEGQNNKVMHRMD

ILWSLVQIFQPKATNR

>AtbHLH103

MTEEFDTTGVCTGTWWSSSNGMFSGCSLPRSAEIVVDFGEIEWQNIDTLDAKTYNENYLS

TSTFLGNANLDTTSQIYVSSPSNIHEEERYNQINSFLEGLFDSSEQLLVPNCPKPELFES

FHFFDDVFPNESRMISVFDHQKPKEDMQACKSLTTCKRASEKSGELEDIESSQPLKRPRL

ETPSHFPSFKVRKEKLGDRITALQQLVSPFGKTDTASVLHDAIDYIKFLQEQITEKVSTS

PHLNSIGSGEQKQWSDKSSNNTHNQNCSPRQDLRSRGLCLMPISSTFSTPPQHLDTSSLW

N

>AtbHLH090

MMMMRGGERVKEFLRPFVDSRTWDLCVIWKLGDDPSRFIEWVGCCCSGCYIDKNIKLENS

EEGGTGRKKKASFCRDDHNKHRIRTLACEALSRFPLFMPLYPGIHGEVVMSKSPKWLVNS

GSKMEMFSTRVLVPVSDGLVELFAFDMRPFDESMVHLIMSRCTTFFEPFPEQRLQFRIIP

RAEESMSSGVNLSVEGGGSSSVSNPSSETQNLFGNYPNASCVEILREEQTPCLIMNKEKD

VVVQNANDSKANKKLLPTENFKSKNLHSERKRRERINQAMYGLRAVVPKITKLNKIGIFS

DAVDYINELLVEKQKLEDELKGINEMECKEIAAEEQSAIADPEAERVSSKSNKRVKKNEV

KIEVHETGERDFLIRVVQEHKQDGFKRLIEAVDLCELEIIDVNFTRLDLTVMTVLNVKAN

KDGIACGILRDLLLKMMITSI

>AtbHLH018

MATAMNVFSTKWSSELDIEEYSIIHQFHMNSLVGDVPQSLSSLDDTTTCYNLDASCNKSL

VEERPSKILKTTHISPNLHPFSSSNPPPPKHQPSSRILSFEKTGLHVMNHNSPNLIFSPK

DEEIGLPEHKKAELIIRGTKRAQSLTRSQSNAQDHILAERKRREKLTQRFVALSALIPGL

KKMDKASVLGDAIKHIKYLQESVKEYEEQKKEKTMESVVLVKKSSLVLDENHQPSSSSSS

DGNRNSSSSNLPEIEVRVSGKDVLIKILCEKQKGNVIKIMGEIEKLGLSITNSNVLPFGP

TFDISIIAQKNNNFDMKIEDVVKNLSFGLSKLT

>AtbHLH144

MQNNQFPHFSDEVGDRNMHNPYASGSSYDALFPPCAKLPYHGVELQPSAVCPKNFVIFDQ

TYDRSQVMYHPELTHKLMNTPSLNNLASTFQNEYVGGSYGNYGNYEQEVSSSYQEDPNEI

DALLSADEDYEENDDNEGEEDGGDSEEVSTARTSSRDYGNTTAESCCSSYGYNNNNNNNS

RKQSLSGSASSSNNDGKGRKKMKKMMGVLRRIVPGGEQMNTACVLDEAVQYLKSLKIEAQ

KLGVGHFSNQS

>AtbHLH029

MEGRVNALSNINDLELHNFLVDPNFDQFINLIRGDHQTIDENPVLDFDLGPLQNSPCFID

ENQFIPTPVDDLFDELPDLDSNVAESFRSFDGDSVRAGGEEDEEDYNDGDDSSATTTNND

GTRKTKTDRSRTLISERRRRGRMKDKLYALRSLVPNITKMDKASIVGDAVLYVQELQSQA

KKLKSDIAGLEASLNSTGGYQEHAPDAQKTQPFRGINPPASKKIIQMDVIQVEEKGFYVR

LVCNKGEGVAPSLYKSLESLTSFQVQNSNLSSPSPDTYLLTYTLDGTCFEQSLNLPNLKL

WITGSLLNQGFEFIKSFT

>AtbHLH111

MLREECTPSSSWWEDVQHHHNDHANSISSTSFYHKSSNNNSHANASCEEDNLSVSTVRAS

NRLDLTAESSNHHSLSASNQPASSSDELLRDHVVSSHNHLWSLAFLPGRSLGDQMMDHHH

HIASRNSSTTSELPSFEPACHNGNGNGWIYDPNQVRYDQSSDQRLSKLTDLVGKHWSIAP

PNNPDMNHNLHHHFDHDHSQNDDISMYRQALEVKNEEDLCYNNGSSGGGSLFHDPIESSR

SFLDIRLSRPLTDINPSFKPCFKALNVSEFNKKEHQTASLLFLQAAVRLGTTNAGKKKRC

EEISDEVSKKAKCSEGSTLSPEKELPKAKLRDKITTLQQIVSPFGKTDTASVLQEAITYI

NFYQEQVKLLSTPYMKNSSMKDPWGGWDREDHNKRGPKHLDLRSRGLCLVPISYTPIAYR

DNSATDYWNPTYRGSLYR

>AtbHLH051

MENSYDSSKWSDSTTPYMVSWSLQSESSDSDWNRFNLGFSSSSFGGNFPADDCVGGIEKA

ESLSRSHRLAEKRRRDRINSHLTALRKLVPNSDKLDKAALLATVIEQVKELKQKAAESPI

FQDLPTEADEVTVQPETISDFESNTNTIIFKASFCCEDQPEAISEIIRVLTKLQLETIQA

EIISVGGRMRINFILKDSNCNETTNIAASAKALKQSLCSALNRITSSSTTTSSVCRIRSK

RQRWFLSSHYSHNE

>AtbHLH032

MYAMKEEDCLQTFHNLQDYQDQFHLHHHPQILPWSSTSLPSFDPLHFPSNPTRYSDPVHY

FNRRASSSSSSFDYNDGFVSPPPSMDHPQNHLRILSEALGPIMRRGSSFGFDGEIMGKLS

AQEVMDAKALAASKSHSEAERRRRERINTHLAKLRSILPNTTKTDKASLLAEVIQHMKEL

KRQTSQITDTYQVPTECDDLTVDSSYNDEEGNLVIRASFCCQDRTDLMHDVINALKSLRL

RTLKAEIATVGGRVKNILFLSREYDDEEDHDSYRRNFDGDDVEDYDEERMMNNRVSSIEE

ALKAVIEKCVHNNDESNDNNNLEKSSSGGIKRQRTSKMVNRCYN

>AtbHLH129

MYPPNSSKSTAHDGGGDADTNQYDSAAGATRDFSSLGPQTHHHPPPQRQQQHQQNPNLVG

HYLPGEPSSIGFDSNASSSSSLFRHRSSPAGFYDQHLPTDPNGFSLGRPNGGYGGGGEQG

PSRLKSELRFSSGSSSHQEHNSLPRISEVEAAAAARNGVASSSMSFGNNRTNNWDNSSSH

ISFTIDQPGKRSKNSDFFTLETQYSMPQTTLEMATMENLMNIPEDSVPCRARAKRGFATH

PRSIAERERRTRISGKLKKLQELVPNMDKQTSYADMLDLAVEHIKGLQHQVESLEKGMER

CTCGACKKR

>AtbHLH153

MEFSRDAGMMMENKRNVCSLGESSIKRHKSDLSFSSKERKDKVGERISALQQIVSPYGKT

DTASVLLDAMHYIEFLHEQVKVCSSIPSMIHSSLSEFPCSFVQVLSAPYLQTVPDATQEE

LEQYSLRNRGLCLVPMENTVGVAQSNGADIWAPVKTPLSPAFSVTSQSPFR

>AtbHLH086

MSLINEHCNERNYISTPNSSEDLSSPQNCGLDEGASASSSSTINSDHQNNQGFVFYPSGE

TIEDHNSLMDFNASSFFTFDNHRSLISPVTNGGAFPVVDGNMSYSYDGWSHHQVDSISPR

VIKTPNSFETTSSFGLTSNSMSKPATNHGNGDWLYSGSTIVNIGSRHESTSPKLAGNKRP

FTGENTQLSKKPSSGTNGKIKPKATTSPKDPQSLAAKNRRERISERLKVLQELVPNGTKV

DLVTMLEKAIGYVKFLQVQVKVLAADEFWPAQGGKAPDISQVKEAIDAILSSSQRDSNST

RETSIAE

>AtbHLH045

MSHIAVERNRRRQMNEHLKSLRSLTPCFYIKRGDQASIIGGVIEFIKELQQLVQVLESKK

RRKTLNRPSFPYDHQTIEPSSLGAATTRVPFSRIENVMTTSTFKEVGACCNSPHANVEAK

ISGSNVVLRVVSRRIVGQLVKIISVLEKLSFQVLHLNISSMEETVLYFFVVKIGLECHLS

LEELTLEVQKSFVSDEVIVSTN

>AtbHLH160

MSSQPNHQTSISSLLHDRLHIPPAETIVEKESAEKDTCQSQRKRKEPVLHEVDGSSSGAA

KKQDHNAKERLRRMRLHASYLTLGTLLPDHSSSSSKVLFSLLLLQVRYVLLVVELYITFL

ADWQKKWSAPSIIDNVITYIPKLQNEVGELTLRKQKLVELERRGPSIRAISVLELGESGY

EAVVQICLKKENEDEFSNLLHVMEVQGLSVLSASTSQVCREQRVVCYNFHVKMDEKPCEG

DDYITVLKNNIISSLRDNTKCK

>AtbHLH093

MELSTQMNVFEELLVPTKQETTDNNINNLSFNGGFDHHHHQFFPNGYNIDYLCFNNEEED

ENTLLYPSSFMDLISQPPPLLLHQPPPLQPLSPPLSSSATAGATFDYPFLEALQEIIDSS

SSSPPLILQNGQEENFNNPMSYPSPLMESDQSKSFSVGYCGGETNKKKSKKLEGQPSKNL

MAERRRRKRLNDRLSMLRSIVPKISKMDRTSILGDAIDYMKELLDKINKLQDEEQELGNS

NNSHHSKLFGDLKDLNANEPLVRNSPKFEIDRRDEDTRVDICCSPKPGLLLSTVNTLETL

GLEIEQCVISCFSDFSLQASCSEGAEQRDFITSEDIKQALFRNAGYGGSCL

>AtbHLH017

MNMSDLGWDDEDKSVVSAVLGHLASDFLRANSNSNQNLFLVMGTDDTLNKKLSSLVDWPN

SENFSWNYAIFWQQTMSRSGQQVLGWGDGCCREPNEEEESKVVRSYNFNNMGAEEETWQD

MRKRVLQKLHRLFGGSDEDNYALSLEKVTATEIFFLASMYFFFNHGEGGPGRCYSSGKHV

WLSDAVNSESDYCFRSFMAKSAGIRTIVMVPTDAGVLELGSVWSLPENIGLVKSVQALFM

RRVTQPVMVTSNTNMTGGIHKLFGQDLSGAHAYPKKLEVRRNLDERFTPQSWEGYNNNKG

PTFGYTPQRDDVKVLENVNMVVDNNNYKTQIEFAGSSVAASSNPSTNTQQEKSESCTEKR

PVSLLAGAGIVSVVDEKRPRKRGRKPANGREEPLNHVEAERQRREKLNQRFYALRSVVPN

ISKMDKASLLGDAISYIKELQEKVKIMEDERVGTDKSLSESNTITVEESPEVDIQAMNEE

VVVRVISPLDSHPASRIIQAMRNSNVSLMEAKLSLAEDTMFHTFVIKSNNGSDPLTKEKL

IAAFYPETSSTQPPLPSSSSQVSGDI

>AtbHLH039

MDRISNLPNEIICHIVSFLSAKEAAFASILSKRWRNLFTIVIKLQFDDSVKNEGSLKDFV

DGVLALPTSSRVRSCSLECRREFDPTHYDDFNRCICALLKRGILDLKLDICAGRRYSLPL

EVFTCKTLVKLELGSDFGGFVVDLVPEDAFLPALETLLLNYIRFKDLRRCAFEKLLSACL

VLKELVIHNMEWERWKWSGNISSPTLERLTISHVDLYECEFTRINLDTPNLTYLELSDAV

PDDYPIVNLDSLVEVKLDLTLMVDHKYHGYVDDNDTISSNPTNLINGLRNVEIMNLQSPN

TFQAFSYFHEAIPVFKNLYHLTITNNDTVIGFCWEFLPFVIKKCPNLKTLVIDGPLHYNE

DRPKSVCHCLSGYSFLLSCPLEVLQITDYSGTPGEVEQLKHFLEKLSGLKLVKLHSLTRF

GSDKKKLLMLPRASSKCKIKHYDSLENALLPSLKTLILDSVKFYDRCGCCAFQKLLSACP

VLVESVMRNLEWEDWEWSGCASSQTLERLTIDHRYWAEHNLESFTFDTPSLTYLDYNAHV

PGSYPTVNLDSLVEAKLNLGFTTDLVEDDDDPFTSDPTNLIKGLRNVEILRLWM

>AtbHLH109

MERNNRNEGTHEEEQCSLSDIIYSFCSENHSELNPLQEIFGVTKNNDHEKHDEEPDEESY

RMAKRQRSMEYRMMMEKKRRKEIKDKVDILQGLMPNHCTKPDLASKLENIIEYIKSLKYQ

VDVMSMAYTTTPVYTPPFYAAAQAPCMSPWGYYTPGVPMMPQQNMTYIPQYPQVYGTVPP

NQTQP

>AtbHLH048

MDLTQGFRARSGVVGPVAGLESLNFSDEFRHLVTTMPPETTGGSFTALLEMPVTQAMELL

HFPDSSSSQARTVTSGDISPTTLHPFGALTFPSNSLLLDRAARFSVIATEQNGNFSGETA

NSLPSNPGANLDRVKAEPAETDSMVENQNQSYSSGKRKEREKKVKSSTKKNKSSVESDKL

PYVHVRARRGQATDNHSLAERARREKINARMKLLQELVPGCDKIQGTALVLDEIINHVQT

LQRQVEMLSMRLAAVNPRIDFNLDSILASENGSLMDGSFNAESYHQLQQWPFDGYHQPEW

GREEDHHQANFSMGSATLHPNQVKMEL

>AtbHLH138

MERYTKKNERFKAEEGKGSKKSRTFLTERERRALFNDRFFDLKNLIPNPTKGGEASIVQD

GIVYINELQRLVSELKYLVEKKKCGARHNNIEVDNKNTIYGTSKIEHPFSKNKNTFNCLI

RTLRFVHHF

>AtbHLH130

MDSNNHLYDPNPTGSGLLRFRSAPSSVLAAFVDDDKIGFDSDRLLSRFVTSNGVNGDLGS

PKFEDKSPVSLTNTSVSYAATLPPPPQLEPSSFLGLPPHYPRQSKGIMNSVGLDQFLGIN

NHHTKPVESNLLRQSSSPAGMFTNLSDQNGYGSMRNLMNYEEDEESPSNSNGLRRHCSLS

SRPPSSLGMLSQIPEIAPETNFPYSHWNDPSSFIDNLSSLKREAEDDGKLFLGAQNGESG

NRMQLLSHHLSLPKSSSTASDMVSVDKYLQLQDSVPCKIRAKRGCATHPRSIAERVRRTR

ISERMRKLQELVPNMDKQTNTSDMLDLAVDYIKDLQRQYKVKFLIIVEKKQRGLFSLLLN

>AtbHLH150

MSSEQGNGSNPSTSPEVEGTKTIPFRRRLQRGQRVFAPKLMEALRRSRVSSEEAPVRHLS

RRWRATTAQKVYSLKLYDALQRSRRSATVRDTADKVLATTARGATRWSRAILVSRFGTSL

RRRRNTKPASALAAAIRGSGGSGRRRKLSAVGNRVRVLGGLVPGCRRTALPELLDETADY

IAALEMQVRAMTALSKILSELQPSTNLGSAL

>AtbHLH046

MELPQPRPFKTQEFRTGRKPTHDFLSLCSHSTVHPDPKPTPPPSSQGSHLKTHDFLQPLE

CVGAKEDVSRINSTTTASEKPPPPAPPPPLQHVLPGGIGTYTISPIPYFHHHHQRIPKPE

LSPPMMFNANERNVLDENSNSNCSSYAAASSGFTLWDESASGKKGQTRKENSVGERVNMR

ADVAATVGQWPVAERRSQSLTNNHMSGFSSLSSSQGSVLKSQSFMDMIRSAKGSSQEDDL

DDEEDFIMKKESSSTSQSHRVDLRVKADVRGSPNDQKLNTPRSKHSATEQRRRSKINDRF

QMLRQLIPNSDQKRDKASFLLEVIEYIQFLQEKADKYVTSYQGWNHEPAKLLNWQSNNNQ

QLVPEGVAFAPKLEEEKNNIPVSVLATAQGVVIDHPTTATTSPFPLSIQSNSFFSPVIAG

NPVPQFHARVASSEAVEPSPSSRSQKEEEDEEVLEGNIRISSVYSQGLVKTLREALENSG

VDLTKASISVEIELAKQSSSSSFKDHEVREPVSRTRNDNVKQTRKPKRLKTGQ

>AtbHLH171

MGRAREIGEGNSSSLREQRNLREKDRRMRMKHLFSILSSHVSPTRKLPVPHLIDQATSYM

IQLKENVNYLKEKKRTLLQGELGNLYEGSFLLPKLSIRSRDSTIEMNLIMDLNMKRVMLH

ELVSIFEEEGAQVMSANLQNLNDRTTYTIIAQAIISRIGIDPSRIEERVRKIIYGYIYFE

A

>AtbHLH117

METPAYDFDSLTDLPPLPPSDFTPSNAFTFPDHNLDFSFLDSTLSLLNRHHLSESTRLEQ

IFYDSTHTQLFHNDDTTTTTTPFLHLPDLKSIDAVEEPTTMKLFPSLSPPLPAAKRQKLN

STSSSTTSGSPTASNDGGIITKRRKISDKIRSLEKLMPWERKMNLAMTLEESHKYIKFLQ

SQIASLRWMPLESVYNTAGEVGETDLLKSLTRQQILQVLANSPGSRNVLSSRGVCVFSYE

QLLSLKTMSRNL

>AtbHLH040

MENGMYKKKGVCDSCVSSKSRSNHSPKRSMMEPQPHHLLMDWNKANDLLTQEHAAFLNDP

HHLMLDPPPETLIHLDEDEEYDEDMDAMKEMQYMIAVMQPVDIDPATVPKPNRRNVRISD

DPQTVVARRRRERISEKIRILKRIVPGGAKMDTASMLDEAIRYTKFLKRQVRILQPHSQI

GAPMANPSYLCYYHNSQP

>AtbHLH095

MTNAQELGQEGFMWGISNSDDSGGGCKRIEKEPLPSHPSHPSPEIQTTTVKKGKKRTKRN

DKNHEEESPDHEIHIWTERERRKKMRDMFSKLHALLPQLPPKADKSTIVDEAVSSIKSLE

QTLQKLEMQKLEKLQYSSASTNTTPTTTFAYAPSSSSSPTALLTPISNHPIDATATDSYP

RAAFLADQVSSSSAAAANLPYPCNDPIVNFDTWSSRNVVLTICGNEAFFNLCVPKHKPGV

FTSVCYLFEKYNMEVLFANVSSNVFWSTYVIQAQVNPSCENQLLGNGLGVVDVFKQVSQE

LVLYFSSL

>AtbHLH126

MDPYKNLNPKGYQRQRPFSSAGESGGSGGSGTAHETDDNKKKKKLLHRDIERQRRQEMAT

LFATLRTHLPLKYIKGKRAVSDHVNGAVNFIKDTEARIKELSARRDELSRETGQGYKSNP

DPGKTGSDVGKSEPATVMVQPHVSGLEVVVSSNSSGPEALPLSKVLETIQEKGLEVMSSF

TTRVNDRLMHTIQVEVNSFGCIDLLWLQQKLVEDLILSTGY

>AtbHLH088

MDSDIMNMMMHQMEKLPEFCNPNSSFFSPDHNNTYPFLFNSTHYQSDHSMTNEPGFRYGS

GLLTNPSSISPNTAYSSVFLDKRNNSNNNNNGTNMAAMREMIFRIAVMQPIHIDPEAVKP

PKRRNVRISKDPQSVAARHRRERISERIRILQRLVPGGTKMDTASMLDEAIHYVKFLKKQ

VQSLEEQAVVTGGGGGGGGRVLIGGGGMTAASGGGGGGGVVMKGCGTVGTHQMVGNAQIL

R

>AtbHLH114

MSHATSTLNSKVNYTIKNMTEEFEIAGISTGAWWSSPTNTAAVFSGYSLPCSTEISPDVT

NFGWQNFDNKINDHNDGCMNMHNSFFEGLLIDPNDQLLPDPWSKSTIPNAKSELLENFPF

LDNMFLVDSEAESLLDHEIRNHKSSKEQITQDYKNLTSKRSEELEENSDEYSPRLLKRPR

LETLSPLPSFKVRKEKLGDRITALQQLVSPFGKTDTASVLNEAVEYIKFLQEQVTVLSNP

EQNTIGSVQQQQCSNKKSINTQGEVEEDECSPRRYVDLSSRGLCLMPISASYPVAAAAAS

AAEMNVHLVSGIFHSL

>AtbHLH158

MASADKLINTDVPEKDVFAFHFLQSLSNLRKQNPFDTPDQKNYRVRKIKKAAYVSMARAA

GGSSRLWSRALLRRADKDDNKIVRFSRRKWKISSKRRRSNQRAPVVEEAAERLRNLVPGG

GGMETSKLMEETAHYIKCLSMQVKVMQCLVDGLSPK

>AtbHLH067

MDGYEATRIVLSRIQSLDPENASKIMGLLLLQDHGEKEMIRLAFGPETLVHSVIVKAKKE

LGLMNCSRSPWSHQDELISPKNNRGSSLNPASLPFYANGGRSSRDLTNDFELMDDMNSRS

TDFLGSVHARSGSCVLDGLGYGGDSDLGFGGVPCSYFARGFCKNGASCRFVHSDGGADLV

GSPSRIELLRSNSVPPRLAHHFMTRSSLPSFSTKGVNLQQNDVQRAAAALMIGDELQKLG

RWRPERIDLSAMACPASRQIYLTFPADSRFREEDVSNYFSTFGPVQDVRIPYQQKRMFGF

VTFVYPETVKSILAKGNPHFVCDSRVLVKPYKEKGKVPDKYRTNQTTERELSPTGLDSSP

RDVLGGRGFYNNTQDVLWRSKFEEEILELQSRRLMNLQLLDVKKHFQLNSPTNIHSPNPF

SQSLISPRPLSVIKREYDGGEKGKGSSKEGSDDDTMNLPERLEDSLPDSPFASPAHHLLL

FADSADNNGSDLWSPSSDNDDNSTPSTLSDSFNSFNYQMPRLPAIGMLPGRGGPTCRVGI

>AtbHLH043

MNNYNMNPSLFQNYTWNNIINSSNNNNKNDDHHHQHNNDPIGMAMDQYTQLHIFNPFSSS

HFPPLSSSLTTTTLLSGDQEDDEDEEEPLEELGAMKEMMYKIAAMQSVDIDPATVKKPKR

RNVRISDDPQSVAARHRRERISERIRILQRLVPGGTKMDTASMLDEAIRYVKFLKRQIRL

LNNNTGYTPPPPQDQASQAVTTSWVSPPPPPSFGRGGRGVGELI

>AtbHLH004

MSPTNVQVTDYHLNQSKTDTTNLWSTDDDASVMEAFIGGGSDHSSLFPPLPPPPLPQVNE

DNLQQRLQALIEGANENWTYAVFWQSSHGFAGEDNNNNNTVLLGWGDGYYKGEEEKSRKK

KSNPASAAEQEHRKRVIRELNSLISGGVGGGDEAGDEEVTDTEWFFLVSMTQSFVKGTGL

PGQAFSNSDTIWLSGSNALAGSSCERARQGQIYGLQTMVCVATENGVVELGSSEIIHQSS

DLVDKVDTFFNFNNGGGEFGSWAFNLNPDQGENDPGLWISEPNGVDSGLVAAPVMNNGGN

DSTSNSDSQPISKLCNGSSVENPNPKVLKSCEMVNFKNGIENGQEEDSSNKKRSPVSNNE

EGMLSFTSVLPCDSNHSDLEASVAKEAESNRVVVEPEKKPRKRGRKPANGREEPLNHVEA

ERQRREKLNQRFYSLRAVVPNVSKMDKASLLGDAISYISELKSKLQKAESDKEELQKQID

VMNKEAGNAKSSVKDRKCLNQESSVLIEMEVDVKIIGWDAMIRIQCSKRNHPGAKFMEAL

KELDLEVNHASLSVVNDLMIQQATVKMGNQFFTQDQLKVALTEKVGECP

>AtbHLH141

MNSHDIDDQLEADVYSNLPSRNDSSTGRRNRNSCRSKHSETEQRRRSKINERFQSLMDII

PQNQNDQKRDKASFLLEVIEYIHFLQEKVHMYEDSHQMWYQSPTKLIPWRNSHGSVAEEN

DHPQIVKSFSSNDKVAASSGFLLDTYNSVNPDIDSAVSTKIPEHSPVSAVSSYLRTEPSL

QFVQHDFWQPKTSCGTINCFTNELLTSDEKTSASLSTVCSQRVLNTLTEALKSSGVNMSE

TMISVQLSLRKREDREYSVAAFASEDNGNSIADEEGDSPTETRSFCNDIDHSQKRIRR

>AtbHLH027

MEDLDHEYKNYWETTMFFQNQELEFDSWPMEEAFSGSGESSSPDGAATSPASSKNVVSER

NRRQKLNQRLFALRSVVPNISKLDKASVIKDSIDYMQELIDQEKTLEAEIRELESRSTLL

ENPVRDYDCNFAETHLQDFSDNNDMRSKKFKQMDYSTRVQHYPIEVLEMKVTWMGEKTVV

VCITCSKKRETMVQLCKVLESLNLNILTTNFSSFTSRLSTTLFLQVTLSLSPSLISLFGN

VITSTNYKILNASREYCTCLVLV

>AtbHLH121

MGIRENGIMLVSRERERARRLENRESIFAEPPCLLLAHRISPSPSILPAEEEVMDVSARK

SQKAGREKLRREKLNEHFVELGNVLDPERPKNDKATILTDTVQLLKELTSEVNKLKSEYT

ALTDESRELTQEKNDLREEKTSLKSDIENLNLQYQQRLRSMSPWGAAMDHTVMMAPPPSF

PYPMPIAMPPGSIPMHPSMPSYTYFGNQNPSMIPAPCPTYMPYMPPNTVVEQQSVHIPQN

PGNRSREPRAKVSRESRSEKAEDSNEVATQLELKTPGSTSDKDTLQRPEKTKRCKRNNNN

NSIEESSHSSKCSSSPSVRDHSSSSSVAGGQKPDDAK

>AtbHLH069

MSKSNPSNLKYYNLHPSFKHHKALNLKRHFNNIHFHSHSPPKKKKTEAMNSSSLLTPSSS

PSPHLQSPATFDHDDFLHHIFSSTPWPSSVLDDTPPPTSDCAPVTGFHHHDADSRNQITM

IPLSHNHPNDALFNGFSTGSLPFHLPQGSGGQTQTQSQATASATTGGATAQPQTKPKVRA

RRGQATDPHSIAERLRRERIAERMKSLQELVPNGNKTDKASMLDEIIDYVKFLQLQVKVL

SMSRLGGAASASSQISEDAGGSHENTSSSGEAKMTEHQVAKLMEEDMGSAMQYLQGKGLC

LMPISLATTISTATCPSRSPFVKDTGVPLSPNLSTTIVANGNGSSLVTVKDAPSVSKP

>AtbHLH122

MESEFQQHHFLLHDHQHQRPRNSGLIRYQSAPSSYFSSFGESIEEFLDRPTSPETERILS

GFLQTTDTSDNVDSFLHHTFNSDGTEKKPPEVKTEDEDAEIPVTATATAMEVVVSGDGEI

SVNPEVSIGYVASVSRNKRPREKDDRTPVNNLARHNSSPAGLFSSIDVETAYAAVMKSMG

GFGGSNVMSTSNTEASSLTPRSKLLPPTSRAMSPISEVDVKPGFSSRLPPRTLSGGFNRS

FGNEGSASSKLTALARTQSGGLDQYKTKDEDSASRRPPLAHHMSLPKSLSDIEQLLSDSI

PCKIRAKRGCATHPRSIAERVRRTKISERMRKLQDLVPNMDTQTNTADMLDLAVQYIKDL

QEQVKALEESRARCRCSSA

>AtbHLH161

MSSRKSRSRQTGASMITDEQINDLVLQLHRLLPELANNRRSGKVSASRVLQETCSYIRNL

SKEVDDLSERLSQLLESTDSAQAALIRSLLMQ

>AtbHLH071

MTLEALSSNGLLNFLLSETLSPTPFKSLVDLEPLPENDVIISKNTISEISNQEPPPQRQP

PATNRGKKRRRRKPRVCKNEEEAENQRMTHIAVERNRRRQMNQHLSVLRSLMPQPFAHKG

DQASIVGGAIDFIKELEHKLLSLEAQKHHNAKLNQSVTSSTSQDSNGEQENPHQPSSLSL

SQFFLHSYDPSQENRNGSTSSVKTPMEDLEVTLIETHANIRILSRRRGFRWSTLATTKPP

QLSKLVASLQSLSLSILHLSVTTLDNYAIYSISAKVEESCQLSSVDDIAGAVHHMLSIIE

EEPFCCSSMSELPFDFSLNHSNVTHSL

>AtbHLH165

MEETLATPDATRRSLSPSCSATVKSRAAGFERRTKRRLSETNASVREDREEAEEEEDEVK

EKIEALQRIIPGGAALGVDALFEETAGYILSLQCQIKTIKVLTSFLQRIDQEDMKFGG

>AtbHLH084

MEAMGEWSTGLGGIYTEEADFMNQLLASYEQPCGGSSSETTATLTAYHHQGSQWNGGFCF

SQESSSYSGYCAAMPRQEEDNNGMEDATINTNLYLVGEETSECDATEYSGKSLLPLETVA

ENHDHSMLQPENSLTTTTDEKMFNQCESSKKRTRATTTDKNKRANKARRSQKCVEMSGEN

ENSGEEEYTEKAAGKRKTKPLKPQKTCCSDDESNGGDTFLSKEDGEDSKALNLNGKTRAS

RGAATDPQSLYARLKQLNKVHCMMVQKRRERINERLRILQHLVPNGTKVDISTMLEEAVQ

YVKFLQLQIKLLSSDDLWMYAPIAYNGMDIGLDLKLNALTR

>AtbHLH094

MPLEAVVYPQDPFGYLSNCKDFMFHDLYSQEEFVAQDTKNNIDKLGHEQSFVEQGKEDDH

QWRDYHQYPLLIPSLGEELGLTAIDVESHPPPQHRRKRRRTRNCKNKEEIENQRMTHIAV

ERNRRKQMNEYLAVLRSLMPSSYAQRGDQASIVGGAINYVKELEHILQSMEPKRTRTHDP

KGDKTSTSSLVGPFTDFFSFPQYSTKSSSDVPESSSSPAEIEVTVAESHANIKIMTKKKP

RQLLKLITSLQSLRLTLLHLNVTTLHNSILYSISVRVRTFSTSHHYFVCFWGNHEIKVSN

DCVCGAYGFTLLIYFFVSYCWGEILD

>AtbHLH002

MATGENRTVPDNLKKQLAVSVRNIQWSYGIFWSVSASQPGVLEWGDGYYNGDIKTRKTIQ

AAEVKIDQLGLERSEQLRELYESLSLAESSASGSSQVTRRASAAALSPEDLTDTEWYYLV

CMSFVFNIGEGIPGGALSNGEPIWLCNAETADSKVFTRSLLAKSASLQTVVCFPFLGGVL

EIGTTEHIKEDMNVIQSVKTLFLEAPPYTTISTRSDYQEIFDPLSDDKYTPVFITEAFPT

TSTSGFEQEPEDHDSFINDGGASQVQSWQFVGEEISNCIHQSLNSSDCVSQTFVGTTGRL

ACDPRKSRIQRLGQIQEQSNHVNMDDDVHYQGVISTIFKTTHQLILGPQFQNFDKRSSFT

RWKRSSSVKTLGEKSQKMIKKILFEVPLMNKKEELLPDTPEETGNHALSEKKRREKLNER

FMTLRSIIPSISKIDKVSILDDTIEYLQDLQKRVQELESCRESADTETRITMMKRKKPDD

EEERASANCMNSKRKGSDVNVGEDEPADIGYAGLTDNLRISSLGNEVVIELRCAWREGIL

LEIMDVISDLNLDSHSVQSSTGDGLLCLTVNCKHKGTKIATTGMIQEALQRVAWIC

>AtbHLH124

MEAKPLASSSSEPNMISPSSNIKPKLKDEDYMELVCENGQILAKIRRPKNNGSFQKQRRQ

SLLDLYETEYSEGFKKNIKILGDTQVVPVSQSKPQQDKETNEQMNNNKKKLKSSKIEFER

NVSKSNKCVESSTLIDVSAKGPKNVEVTTAPPDEQSAAVGRSTELYFASSSKFSRGTSRD

LSCCSLKRKYGDIEEEESTYLSNNSDDESDDAKTQVHARTRKPVTKRKRSTEVHKLYERK

RRDEFNKKMRALQDLLPNCYKDDKASLLDEAIKYMRTLQLQVQMMSMGNGLIRPPTMLPM

GHYSPMGLGMHMGAAATPTSIPQFLPMNVQATGFPGMNNAPPQMLSFLNHPSGLIPNTPI

FSPLENCSQPFVVPSCVSQTQATSFTQFPKSASASNLEDAMQYRGSNGFSYYRSPN

>AtbHLH053

MSMDCLSYFFNYDPPVQLQDCFIPEMDMIIPETDSFFFQSQPQLEFHQPLFQEEAPSQTH

FDPFCDQFLSPQEIFLPNPKNEIFNETHDLDFFLPTPKRQRLVNSSYNCNTQNHFQSRNP

NFFDPFGDTDFVPESCTFQEFRVPDFSLAFKVGRGDQDDSKKPTLSSQSIAARGRRRRIA

EKTHELGKLIPGGNKLNTAEMFQAAAKYVKFLQSQVGILQLMQTTKKGSSNVQMETQYLL

ESQAIQEKLSTEEVCLVPCEMVQDLTTEETICRTPNISREINKLLSKHLAN

>AtbHLH008

MPLFELFRLTKAKLESAQDRNPSPPVDEVVELVWENGQISTQSQSSRSRNIPPPQANSSR

AREIGNGSKTTMVDEIPMSVPSLMTGLSQDDDFVPWLNHHPSLDGYCSDFLRDVSSPVTV

NEQESDMAVNQTAFPLFQRRKDGNESAPAASSSQYNGFQSHSLYGSDRARDLPSQQTNPD

RFTQTQEPLITSNKPSLVNFSHFLRPATFAKTTNNNLHDTKEKSPQSPPNVFQTRVLGAK

DSEDKVLNESVASATPKDNQKACLISEDSCRKDQESEKAVVCSSVGSGNSLDGPSESPSL

SLKRKHSNIQDIDCHSEDVEEESGDGRKEAGPSRTGLGSKRSRSAEVHNLSERRRRDRIN

EKMRALQELIPNCNKVDKASMLDEAIEYLKSLQLQVQIMSMASGYYLPPAVMFPPGMGHY

PAAAAAMAMGMGMPYAMGLPDLSRGGSSVNHGPQFQVSGMQQQPVAMGIPRVSGGGIFAG

SSTIGNGSTRDLSGSKDQTTTNNNSNLKPIKRKQGSSDQFCGSS

>AtbHLH055

MNFPDSSLFTPNFAYENDLDFSSLITPSTRVSFQEPKPCNPVIHSAGIENDGRQNCETTM

TLSEIMKGDDEPKNKRAKHKELERQRRQENTSLFKILRYLLPSQYIKGKRSSADHVLEAV

NYIKDLQKKIKEVSEKRDRIKRSITHPSSRGEFSIRSLASSTCSCVGDTNIAVVVRPCLI

GLEIVVSCCNRHESCLSSVLQLLAQEQCFNIVSCISTRLHQGFIHTIASEVEEGIEVYFS

ELQEKIIKIGTSRVTTR

>AtbHLH099

MMFQQDYPHGFSLVETSLSYEMLDYFQNIVVSNSEDVASQQNSISSSSYSSATLSCSITE

QKSHLTEKLSPLRERYGCGDFLSRKRRRRSEKTIVDKENQRMNHIAVERNRRKQMNHFLS

ILKSMMPLSYSQPNDQASIIEGTISYLKKLEQRLQSLEAQLKATKLNQSPNIFSDFFMFP

QYSTATATATATASSSSSSHHHHKRLEVVADVEVTMVERHANIKVLTKTQPRLLFKIINE

FNSLGLSTLHLNLTTSKDMSLFTFSVKVEADCQLTPSGNEVANTVHEVVRRVHKER

>AtbHLH137

MATFSYFQNYPHSLLDPLLFPTPHSSINLTSFIDQNHLYPLPNISTVEDISFLEYNVDKT

ENSGSEKLANTTKTATTGSSSCDQLSHGPSAITNTGKTRGRKARNSNNSKEGVEGRKSKK

QKRGSKEEPPTDYIHVRARRGQATDSHSLAERVRREKISERMRTLQNLVPGCDKVTGKAL

MLDEIINYVQTLQTQVEFLSMKLTSISPVVYDFGSDLDGLILQSEMGSPEVGTSFTNAMP

TTTPIFPSLLDNSVVPTHAQVQEEGEERENFVDRSGFNNNNFCSFP

>AtbHLH001

MGYRDEETMATGQNRTTVPENLKKHLAVSVRNIQWSYGIFWSVSASQSGVLEWGDGYYNG

DIKTRKTIQASEIKADQLGLRRSEQLSELYESLSVAESSSSGVAAGSQVTRRASAAALSP

EDLADTEWYYLVCMSFVFNIGEGMPGRTFANGEPIWLCNAHTADSKVFSRSLLAKSAAVK

TVVCFPFLGGVVEIGTTEHITEDMNVIQCVKTSFLEAPDPYATILPARSDYHIDNVLDPQ

QILGDEIYAPMFSTEPFPTASPSRTTNGFDQEHEQVADDHDSFMTERITGGASQVQSWQL

MDDELSNCVHQSLNSSDCVSQTFVEGAAGRVAYGARKSRVQRLGQIQEQQRNVKTLSFDP

RNDDVHYQSVISTIFKTNHQLILGPQFRNCDKQSSFTRWKKSSSSSSGTATVTAPSQGML

KKIIFDVPRVHQKEKLMLDSPEARDETGNHAVLEKKRREKLNERFMTLRKIIPSINKIDK

VSILDDTIEYLQELERRVQELESCRESTDTETRGTMTMKRKKPCDAGERTSANCANNETG

NGKKVSVNNVGEAEPADTGFTGLTDNLRIGSFGNEVVIELRCAWREGVLLEIMDVISDLH

LDSHSVQSSTGDGLLCLTVNCKHKGSKIATPGMIKEALQRVAWIC

>AtbHLH142

MPLDKRQRDLPLGLSPQACFKDIVGRSVLPRIPLPELGKLYAAKLQARCLQPPPFQSLLC

SHDKESYGKRFSRSDMRSWCAAATTTTTPLGALESSQKRLLIFDQSGDQTRLLQCPFPLR

FPSHAAAEPVKLSELQGIEKAFKEDGEEFHKSDGTESEMHEDTEEINALLYSDDDYDDDC

ESDDEVMSTGHSPYPNEGVCNKRELEEIDGPCKRQKLLDKVNNISDLSSLVGTESSTQLN

GSSFLKDKKLPESKTISTKEDTGSGLSNEQSKKDKIRTALKILESVVPGAKGNEALLLLD

EAIDYLKLLKRDLISTEVKNQSSTTHKSPILLLKETTWGTRNLQTDKA

>AtbHLH059

MASNNPHDNLSDQTPSDDFFEQILGLPNFSASSAAGLSGVDGGLGGGAPPMMLQLGSGEE

GSHMGGLGGSGPTGFHNQMFPLGLSLDQGKGPGFLRPEGGHGSGKRFSDDVVDNRCSSMK

PVFHGQPMQQPPPSAPHQPTSIRPRVRARRGQATDPHSIAERLRRERIAERIRALQELVP

TVNKTDRAAMIDEIVDYVKFLRLQVKVLSMSRLGGAGAVAPLVTDMPLSSSVEDETGEGG

RTPQPAWEKWSNDGTERQVAKLMEENVGAAMQLLQSKALCMMPISLAMAIYHSQPPDTSS

VVKPENNPPQ

>AtbHLH127

MMIISSQILLLFGFKLFFETRGEDDIVELLCKIGQTQIPSSDPLPILRGSGSGGREENTP

LPPPLPHQNLFIQEDEMSSWPHHPLRQDYLCSELYASTPAPHPQSSVSLAPPPPKPPSSA

PYGQIIAPRSAPRIQGTEEARGSTSRKRSRAAEMHNLAERRRREKINERMKTLQQLIPRC

NKSTKVSMLEDVIEYVKSLEMQINQFMPHMAMGMNQPPAYIPFPSQAHMAGVGPSYPPPR

YPFPNIQTFDPSRVWLQSPQPNPVSNQPQMNPYGQFVGHHQMQQSLPPPLQVILSQYPLC

LFLCSNK

>AtbHLH131

MVLLHHVSLSHYQNSSSLFSSSSESILCLFLVLCVMQLEQGMRPISRCYNPTAYSTTMGR

SFFAGAATSSKLFSRGFSVTKPKSKTESKEVAAKKHSDAERRRRLRINSQFATLRTILPN

LVKQDKASVLGETVRYFNELKKMVQDIPTTPSLEDNLRLDHCNNNRDLARVVFSCSDREG

LMSEVAESMKAVKAKAVRAEIMTVGGRTKCALFVQGVNGNEGLVKLKKSLKLVVNGKSSS

EAKNNNNGGSLLIQQQ

>AtbHLH097

MDKDYSAPNFLGESSGGNDDNSSGMIDYMFNRNLQQQQKQSMPQQQQHQLSPSGFGATPF

DKMNFSDVMQFADFGSKLALNQTRNQDDQETGIDPVYFLKFPVLNDKIEDHNQTQHLMPS

HQTSQEGGECGGNIGNVFLEEKEDQDDDNDNNSVQLRFIGGEEEDRENKNVTKKEVKSKR

KRARTSKTSEEVESQRMTHIAVERNRRKQMNEHLRVLRSLMPGSYVQRGDQASIIGGAIE

FVRELEQLLQCLESQKRRRILGETGRDMTTTTTSSSSPITTVANQAQPLIITGNVTELEG

GGGLREETAENKSCLADVEVKLLGFDAMIKILSRRRPGQLIKTIAALEDLHLSILHTNIT

TMEQTVLYSFNVKITSETRFTAEDIASSIQQIFSFIHANTNISGSSNLGNIVFT

>AtbHLH143

MPLDTKQQKWLPLGLNPQACVQDKATEYFRPGIPFPELGKVYAAEHQFRYLQPPFQALLS

RYDQQSCGKQVSCLNGRSSNGAAPEGALKSSRKRFIVFDQSGEQTRLLQCGFPLRFPSSM

DAERGNILGALHPEKGFSKDHAIQEKILQHEDHENGEEDSEMHEDTEEINALLYSDDDDN

DDWESDDEVMSTGHSPFTVEQQACNITTEELDETESTVDGPLLKRQKLLDHSYRDSSPSL

VGTTKVKGLSDENLPESNISSKQETGSGLSDEQSRKDKIHTALRILESVVPGAKGKEALL

LLDEAIDYLKLLKQSLNSSKGLNNHW

>AtbHLH115

MVSPENTNWLSDYPLIEGAFSDQNPTFPWQIDGSATVRIGSYIWRSCYGCVITQLVFRCS

CCDRDIGLLHKSILCFFFCLRFAYVPLFPKEVDESRSLPIAKGSVEVDGFLCDADVIKEP

SSRKRIKTESCTGSNSKACREKQRRDRLNDKFTELSSVLEPGRTPKTDKVAIINDAIRMV

NQARDEAQKLKDLNSSLQEKIKELKDEKNELRDEKQKLKVEKERIDQQLKAIKTQPQPQP

CFLPNPQTLSQAQAPGSKLVPFTTYPGFAMWQFMPPAAVDTSQDHVLRPPVA

>AtbHLH014

MYNLTFSPSLSSSLLSFTQQTPAAIVSSSPPDLVLQQKLRFVVETSPDRWAYVIFWQKMF

DDQSDRSYLVWVDGHFCGNKNNNSQENYTTNSIECELMMDGGDDLELFYAASFYGEDRSP

RKEVSDESLVWLTGPDELRFSNYERAKEAGFHGVHTLVSIPINNGIIELGSSESIIQNRN

FINRVKSIFGSGKTTKHTNQTGSYPKPAVSDHSKSGNQQFGSERKRRRKLETTRVAAATK

EKHHPAVLSHVEAEKQRREKLNHRFYALRAIVPKVSRMDKASLLSDAVSYIESLKSKIDD

LETEIKKMKMTETDKLDNSSSNTSPSSVEYQVNQKPSKSNRGSDLEVQVKIVGEEAIIRV

QTENVNHPTSALMSALMEMDCRVQHANASRLSQVMVQDVVVLVPEGLRSEDRLRTTLVRT

LSL

>AtbHLH089

MGGGGMFEEIGCFDPNAPAEMTAESSFSPSEPPPTITVIGSNSNSNCSLEDLSAFHLSPQ

DSSLPASASAYAHQLHINATPNCDHQFQSSMHQTLQDPSYAQQSNHWDNGYQDFVNLGPN

HTTPDLLSLLQLPRSSLPPFANPSIQDIIMTTSSSVAAYDPLFHLNFPLQPPNGSFMGVD

QDQTETNQGVNLMYDEENNNLDDGLNRKGRGSKKRKIFPTERERRVHFKDRFGDLKNLIP

NPTKNDRASIVGEAIDYIKELLRTIDEFKLLVEKKRVKQRNREGDDVVDENFKAQSEVVE

QCLINKKNNALRCSWLKRKSKFTDVDVRIIDDEVTIKIVQKKKINCLLFVSKVVDQLELD

LHHVAGAQIGEHHSFLFNAKISEGSSVYASAIADRVMEVLKKQYMEALSANNGYHCYSSD

>AtbHLH104

MYPSLDDDFVSDLFCFDQSNGAELDDYTQFGVNLQTDQEDTFPDFVSYGVNLQQEPDEVF

SIGASQLDLSSYNGVLSLEPEQVGQQDCEVVQEEEVEINSGSSGGAVKEEQEHLDDDCSR

KRARTGSCSRGGGTKACRERLRREKLNERFMDLSSVLEPGRTPKTDKPAILDDAIRILNQ

LRDEALKLEETNQKLLEEIKSLKAEKNELREEKLVLKADKEKTEQQLKSMTAPSSGFIPH

IPAAFNHNKMAVYPSYGYMPMWHYMPQSVRDTSRDQELRPPAA

>AtbHLH020

MDDSSFMDLMIDTDEYLIDDWESDFPICGETNTNPGSESGSGTGFELLAERPTKQMKTNN

NMNSTSSSPSSSSSSGSRTSQVISFGSPDTKTNPVETSLNFSNQVSMDQKVGSKRKDCVN

NGGRREPHLLKEHVLAERKRRQKLNERLIALSALLPGLKKTDKATVLEDAIKHLKQLQER

VKKLEEERVVTKKMDQSIILVKRSQVYLDDDSSSYSSTCSAASPLSSSSDEVSIFKQTMP

MIEARVSDRDLLIRVHCEKNKGCMIKILSSLEKFRLEVVNSFTLPFGNSTLVITILTKMD

NKFSRPVEEVVKNIRVALAE

>AtbHLH146

MERQIINRKKRVFSLEPNKNPSAVFTRKYTSHLVPALKKLNMNKNSSKQTVKHEVDMALA

LSAQEFAWSRFLLQKLSSSSNPTTTTSSSSDGIRILERPDKEGGNEEGGIEERLRELKKL

LPGGEEMNVEEMLSEIGNYIKCLELQTIALKSIVQDST

>AtbHLH154

MEYSRDSAEMMMETKRNVYSLEDNKIKRHKSSDLSFSSKERKDKLAERISALQQLVSPYG

KTDTASVLLEGMQYIQFLQEQVKVLSAPYLQATPSTTEEEVEEYSLRSKGLCLVPLEYTS

EVAQTNGADIWAPVKTPTSSHAFNLSSSNSPFQ

>AtbHLH132

MMFLPTDYCCRLSDQEYMELVFENGQILAKGQRSNVSLHNQRTKSIMDLYEAEYNEDFMK

SIIHGGGGAITNLGDTQVVPQSHVAAAHETNMLESNKHVDDSETLKASSSKRMMVDYHNR

KKIKFIPPDEQSVVADRSFKLGFDTSSVGFTEDSEGSMYLSSSLDDESDDARPQVPARTR

KALVKRKRNAEAYNSPERNQRNDINKKMRTLQNLLPNSHKDDNESMLDEAINYMTNLQLQ

VQMMTMGNRFVTPSMMMPLGPNYSQMGLAMGVGMQMGEQQFLPAHVLGAGLPGINDSADM

LRFLNHPGLMPMQNSAPFIPTENCSPQSVPPSCAAFPNQIPNPNSLSNLDGATLHKKSRK

TNR

>AtbHLH076

MDNELFMNTEFPPPPEMATHFEHQQSSSSAMMLNWALMDPNPHQDSSFLWEKSTEQQQQQ

SIFDSALSSLVSSPTPSNSNFSGGGGDGFLIRELIGKLGNIGNNNNNSGEIYGTPMSRSA

SCYATPMSSPPPPTNSNSQMMMNRTTPLTEFSADPGFAERAARFSCFGSRSFNGRTNTNL

PINNGNNMVNNSGKLTRVSSTPALKALVSPEVTPGGEFSRKRKSVPKGKSKENPISTASP

SPSFSKTAEKNGGKGGSKSSEEKGGKRRREEEDDEEEEGEGEGNKSNNTKPPEPPKDYIH

VRARRGQATDSHSLAERVRREKIGERMKLLQDLVPGCNKVTGKALMLDEIINYVQSLQRQ

VEFLSMKLSSVNDTRLDFNVDALVSKDVMIPSSNNRLHEEGLQSKSSSHHHQQQLNIYNN

NSQLLPNISSNNMMLQSPMNSLETSTLARSFTHLPTLTQFTDSISQYQMFSEEDLQSIVG

MGVAENPNNESQHMKIEL

>AtbHLH054

MDVFVDGELESLLGMFNFDQCSSSKEERPRDELLGLSSLYNGHLHQHQHHNNVLSSDHHA

FLLPDMFPFGAMPGGNLPAMLDSWDQSHHLQETSSLKRKLLDVENLCKTNSNCDVTRQEL

AKSKKKQRVSSESNTVDESNTNWVDGQSLSNSSDDEKASVTSVKGKTRATKGTATDPQSL

YARKRREKINERLKTLQNLVPNGTKVDISTMLEEAVHYVKFLQLQIKLLSSDDLWMYAPL

AYNGLDMGFHHNLLSRLM

>AtbHLH157

MGSEYKHILKSLCLSHGWSYAVFWRYDPINSMILRFEEAYNDEQSVALVDDMVLQAPILG

QGIVGEVASSGNHQWLFSDTLFQWEHEFQNQFLCGFKILIRQFTYTQTIAIIPLGSSGVV

QLGSTQKILESTEILEQTTRALQETCLKPHDSGDLDTLFESLGDCEIFPAESFQGFSFDD

IFAEDNPPSLLSPEMISSEAASSNQDLTNGDDYGFDILQSYSLDDLYQLLADPPEQNCSS

MVIQGVDKDLFDILGMNSQTPTMALPPKGLFSELISSSLSNNTCSSSLTNVQEYSGVNQS

KRRKLDTSSAHSSSLFPQEETVTSRSLWIDDDERSSIGGNWKKPHEEGVKKKRAKAGESR

RPRPKDRQMIQDRIKELRGMIPNGAKCSIDTLLDLTIKHMVFMQSLAKYAERLKQPYESK

LVKEKERTWALEVGEEGVVCPIMVEELNREGEMQIEMVCEEREEFLEIGQVVRGLGLKIL

KGVMETRKGQIWAHFIVQAKPQVTRIQVLYSLVQLFQHHTKHDDLLS

>AtbHLH025

MSILSTRWFSEQEIEENSIIQQFHMNSIVGEVQEAQYIFPHSFTTNNDPSYDDLIEMKPP

KILETTYISPSSHLPPNSKPHHIHRHSSSRILSFEDYGSNDMEHEYSPTYLNSIFSPKLE

AQVQPHQKSDEFNRKGTKRAQPFSRNQSNAQDHIIAERKRREKLTQRFVALSALVPGLKK

MDKASVLGDALKHIKYLQERVGELEEQKKERRLESMVLVKKSKLILDDNNQSFSSSCEDG

FSDLDLPEIEVRFSDEDVLIKILCEKQKGHLAKIMAEIEKLHILITNSSVLNFGPTLDIT

IIAKKESDFDMTLMDVVKSLRSALSNFI

>AtbHLH023

MTWKPKMLILSHDLISPEKYIMGEDDIVELLGKSSQVVTSSQTQTPSCDPPLILRGSGSG

DGEGNGPLPQPPPPLYHQQSLFIQEDEMASWLHQPNRQDYLYSQLLYSGVASTHPQSLAS

LEPPPPPRAQYILAADRPTGHILAERRAENFMNISRQRGNIFLGGVEAVPSNSTLLSSAT

ESIPATHGTESRATVTGGVSRTFAVPGLGPRGKAVAIETAGTQSWGLCKAETEPVQRQPA

TETDITDERKRKTREETNVENQGTEEARDSTSSKRSRAAIMHKLSERRRRQKINEMMKAL

QELLPRCTKTDRSSMLDDVIEYVKSLQSQIQMFSMGHVMIPPMMYAGNIQQQYMPHMAMG

MNRPPAFIPFPRQAHMAEGVGPVDLFRENEETEQETMSLLLREDKRTKQKMFS

>AtbHLH083

MALVNDHPNETNYLSKQNSSSSEDLSSPGLDQPDAAYAGGGGGGGSASSSSTMNSDHQQH

QGFVFYPSGEDHHNSLMDFNGSSFLNFDHHESFPPPAISCGGSSGGGGFSFLEGNNMSYG

FTNWNHQHHMDIISPRSTETPQGQKDWLYSDSTVVTTGSRNESLSPKSAGNKRSHTGEST

QPSKKLSSGVTGKTKPKPTTSPKDPQSLAAKNRRERISERLKILQELVPNGTKVDLVTML

EKAISYVKFLQVQVKVLATDEFWPAQGGKAPDISQVKDAIDAILSSSQRDRNSNLITN

>AtbHLH119

MGEDDIVELLWNGQVVRTSQPQRPSSGKPSPTPPILRGSGSGSGEENAPLPLPLLQPPRP

LHHQNLFIREEEMSSWLHYSYTGVTSTPATHPQSSVSLPPPPPIAPSEDDVVELLWKSGQ

VVQSIQTQRPIPPPIFRGSGSGGGEETVLPLPPLHPSHQNIFIQEDEMASWLYHPLRQDY

FSSGVASTSATRPQSSASLAPTPPPPSVPYGQIPVERRTENFMNFLRLRGNIFSGGRVEA

GPVVIESTQIGSSATPSSSAAESCVIPATHGTESRAAAITGVSRTFAVPGLGRRGKEVAT

ETAGTSYSGVNKAETERVQIQPERETKITEDKKREETIAEIQGTEEAHGSTSRKRSRAAD

MHNLSERRRRERINERMKTLQELLPRCRKTDKVSMLEDVIEYVKSLQLQIQMMSMGHGMM

PPMMHEGNTQQFMPHMAMGMKGMNRPPPFVPFPGKTFPRPGHMAGVGPSYPALRYPFPDT

QASDLSRVHVPSLHSNPVPNQPRFPAYINPYSQFVGLHQMQQPPLPLQGQPTSQPSFSHA

STSK

>AtbHLH050

MANLSSDFQTFTMDDPIRQLAELSNTLHHFQTFPPPFSSSLDSLFFHNQFPDHFPGKSLE

NNFHQGIFFPSNIQNNEESSSQFDTKKRKSLMEAVSTSENSVSDQTLSTSSAQVSINGNI

STKNNSSRRGKRSKNREEEKEREVVHVRARRGQATDSHSIAERVRRGKINERLKCLQDIV

PGCYKTMGMATMLDEIINYVQSLQNQVEFLSMKLTAASSYYDFNSETDAVESMQKAKARE

AVEMGQGRDGSSVFHSSSWTL

>AtbHLH066

MMNSSLLTPSSSSSSHIQTPSTTFDHEDFLDQIFSSAPWPSVVDDAHPLPSDGFHGHDVD

SRNQPIMMMPLNDGSSVHALYNGFSVAGSLPNFQIPQGSGGGLMNQQGQTQTQTQPQASA

STATGGTVAAPPQSRTKIRARRGQATDPHSIAERLRRERIAERMKALQELVPNGNKTDKA

SMLDEIIDYVKFLQLQVKVLSMSRLGGAASVSSQISEAGGSHGNASSAMVGGSQTAGNSN

DSVTMTEHQVAKLMEEDMGSAMQYLQGKGLCLMPISLATAISTATCHSRNPLIPGAVADV

GGPSPPNLSGMTIQSTSTKMGSGNGKLNGNGVTERSSSIAVKEAVSVSKA

>AtbHLH009

MLLLVSLNHKLSLALFTSAIKYILRLIKLFFLFILKCLQRSDMEHQGWSFEENYSLSTNR

RSIRPQDELVELLWRDGQVVLQSQTHREQTQTQKQDHHEEALRSSTFLEDQETVSWIQYP

PDEDPFEPDDFSSHFFSTMDPLQRPTSETVKPKSSPEPPQVMVKPKACPDPPPQVMPPPK

FRLTNSSSGIRETEMEQYSVTTVGPSHCGSNPSQNDLDVSMSHDRSKNIEEKLNPNASSS

SGGSSGCSFGKDIKEMASGRCITTDRKRKRINHTDESVSLSDAIGNKSNQRSGSNRRSRA

AEVHNLSERRRRDRINERMKALQELIPHCSKTDKASILDEAIDYLKSLQLQLQVMWMGSG

MAAAAASAPMMFPGVQPQQFIRQIQSPVQLPRFPVMDQSAIQNNPGLVCQNPVQNQIISD

RFARYIGGFPHMQAATQMQPMEMLRFSSPAGQQSQQPSSVPTKTTDGSRLDH

>AtbHLH047

MVSKTPSTSSDEANATADERCRKGKVPKRINKAVRERLKREHLNELFIELADTLELNQQN

SGKASILCEATRFLKDVFGQIESLRKEHASLLSESSYVTTEKNELKEETSVLETEISKLQ

NEIEARANQSKPDLNTSPAPEYHHHHYQQQHPERVSQFPGLPIFQGPGFQQSATTLHPPA

TVLVLPIQPDPQTQDISEMTQAQQPLMFNSSNVSKPCPRYASAADSWSSRLLGERLKASE

>AtbHLH073

MGDSDVGDRLPPPSSSDELSSFLRQILSRTPTAQPSSPPKSTNVSSAETFFPSVSGGAVS

SVGYGVSETGQDKYAFEHKRSGAKQRNSLKRNIDAQFHNLSEKKRRSKINEKMKALQKLI

PNSNKTDKASMLDEAIEYLKQLQLQVQTLAVMNGLGLNPMRLPQVPPPTHTRINETLEQD

LNLETLLAAPHSLEPAKTSQGMCFSTATLL

>AtbHLH098

MQEIIPDFLEECEFVDTSLAGDDLFAILESLEGAGEISPTAASTPKDGTTSSKELVKDQD

YENSSPKRKKQRLETRKEEDEEEEDGDGEAEEDNKQDGQQKMSHVTVERNRRKQMNEHLT

VLRSLMPCFYVKRGDQASIIGGVVEYISELQQVLQSLEAKKQRKTYAEVLSPRVVPSPRP

SPPVLSPRKPPLSPRINHHQIHHHLLLPPISPRTPQPTSPYRAIPPQLPLIPQPPLRSYS

SLASCSSLGDPPPYSPASSSSSPSVSSNHESSVINELVANSKSALADVEVKFSGANVLLK

TVSHKIPGQVMKIIAALEDLALEILQVNINTVDETMLNSFTIKIGIECQLSAEELAQQIQ

QTFC

>AtbHLH042

MDESSIIPAEKVAGAEKKELQGLLKTAVQSVDWTYSVFWQFCPQQRVLVWGNGYYNGAIK

TRKTTQPAEVTAEEAALERSQQLRELYETLLAGESTSEARACTALSPEDLTETEWFYLMC

VSFSFPPPSGMPGKAYARRKHVWLSGANEVDSKTFSRAILAKSAKIQTVVCIPMLDGVVE

LGTTKKVREDVEFVELTKSFFYDHCKTNPKPALSEHSTYEVHEEAEDEEEVEEEMTMSEE

MRLGSPDDEDVSNQNLHSDLHIESTHTLDTHMDMMNLMEEGGNYSQTVTTLLMSHPTSLL

SDSVSTSSYIQSSFATWRVENGKEHQQVKTAPSSQWVLKQMIFRVPFLHDNTKDKRLPRE

DLSHVVAERRRREKLNEKFITLRSMVPFVTKMDKVSILGDTIAYVNHLRKRVHELENTHH

EQQHKRTRTCKRKTSEEVEVSIIENDVLLEMRCEYRDGLLLDILQVLHELGIETTAVHTS

VNDHDFEAEIRAKVRGKKASIAEVKRAIHQVIIHDTNL

>AtbHLH016

MSQCVPNCHIDDTPAAATTTVRSTTAADIPILDYEVAELTWENGQLGLHGLGPPRVTASS

TKYSTGAGGTLESIVDQATRLPNPKPTDELVPWFHHRSSRAAMAMDALVPCSNLVHEQQS

KPGGVGSTRVGSCSDGRTMGGGKRARVAPEWSGGGSQRLTMDTYDVGFTSTSMGSHDNTI

DDHDSVCHSRPQMEDEEEKKAGGKSSVSTKRSRAAAIHNQSERKRRDKINQRMKTLQKLV

PNSSKTDKASMLDEVIEYLKQLQAQVSMMSRMNMPSMMLPMAMQQQQQLQMSLMSNPMGL

GMGMGMPGLGLLDLNSMNRAAASAPNIHANMMPNPFLPMNCPSWDASSNDSRFQSPLIPD

PMSAFLACSTQPTTMEAYSRMATLYQQMQQQLPPPSNPK

>AtbHLH005

MNGTTSSINFLTSDDDASAAAMEAFIGTNHHSSLFPPPPQQPPQPQFNEDTLQQRLQALI

ESAGENWTYAIFWQISHDFDSSTGDNTVILGWGDGYYKGEEDKEKKKNNTNTAEQEHRKR

VIRELNSLISGGIGVSDESNDEEVTDTEWFFLVSMTQSFVNGVGLPGESFLNSRVIWLSG

SGALTGSGCERAGQGQIYGLKTMVCIATQNGVVELGSSEVISQSSDLMHKVNNLFNFNNG

GGNNGVEASSWGFNLNPDQGENDPALWISEPTNTGIESPARVNNGNNSNSNSKSDSHQIS

KLEKNDISSVENQNRQSSCLVEKDLTFQGGLLKSNETLSFCGNESSKKRTSVSKGSNNDE

GMLSFSTVVRSAANDSDHSDLEASVVKEAIVVEPPEKKPRKRGRKPANGREEPLNHVEAE

RQRREKLNQRFYSLRAVVPNVSKMDKASLLGDAISYINELKSKLQQAESDKEEIQKKLDG

MSKEGNNGKGCGSRAKERKSSNQDSTASSIEMEIDVKIIGWDVMIRVQCGKKDHPGARFM

EALKELDLEVNHASLSVVNDLMIQQATVKMGSQFFNHDQLKVALMTKVGENY

>AtbHLH110

MDSANLHQLQDQLQLVGSSSSSSSLDNNSDPSCYGASSAHQWSPGGISLNSVSLSHNYNN

EMLNTRAHNNNNNNNTSECMSLSSIHNHSLIQQQDFPLQWPHDQSSYQHHEGLLKIKEEL

SSSTISDHQEGISKFTDMLNSPVITNYLKINEHKDYTEKLLLKSMSSGFPINGDYGSSLP

SSSSSSSPSSQSHRGNFSQIYPSVNISSLSESRKMSMDDMSNISRPFDINMQVFDGRLFE

GNVLVPPFNAQEISSLGMSRGSLPSFGLPFHHHLQQTLPHLSSSPTHQMEMFSNEPQTSE

GKRHNFLMATKAGENASKKPRVESRSSCPPFKVRKEKLGDRIAALQQLVSPFGKTDTASV

LMEAIGYIKFLQSQIETLSVPYMRASRNRPGKASQLVSQSQEGDEEETRDLRSRGLCLVP

LSCMTYVTGDGGDGGGGVGTGFWPTPPGFGGGT

>AtbHLH169

MFLFFTMRILKTQRSRGGRRTSKKFGNRRTSGGEKFSEKLQALKSLLPPPSKMTEQSRQD

AYVEEDSSVGETEQLFQETADYIVRLRGQVVVLQKLIEIYGSSDQKEDNFVS

>AtbHLH070

MFVLRVSNQSFKLHQQVQCKDEIFCLDQKVNVRRSLQVQETVEDHQSFALEEEEQQLSTP

SLLQDTTIPFLQMLQQSEDPSPFLSFKDPSFLALLSLQTLEKPWELENYLPHEVPEFHSP

IHSETNHYYHNPSLEGVNEAISNQELPFNPLENARSRRKRKNNNLASLMTREKRKRRRTK

PTKNIEEIESQRMTHIAVERNRRRQMNVHLNSLRSIIPSSYIQRGDQASIVGGAIDFVKI

LEQQLQSLEAQKRSQQSDDNKEQIPEDNSLRNISSNKLRASNKEEQSSKLKIEATVIESH

VNLKIQCTRKQGQLLRSIILLEKLRFTVLHLNITSPTNTSVSYSFNLKMEDECNLGSADE

ITAAIRQIFDS

>AtbHLH100

MCALVPPLYPNFGWPCGDHSFYETDDVSNTFLDFPLPDLTVTHENVSSENNRTLLDNPVV

MKKLNHNASERERRKKINTMFSSLRSCLPPTNQTKKLSVSATVSQALKYIPELQEQVKKL

MKKKEELSFQISGQRDLVYTDQNSKSEEGVTSYASTVSSTRLSETEVMVQISSLQTEKCS

FGNVLSGVEEDGLVLVGASSSRSHGERLFYSMHLQIKNGQVNSEELGDRLLYLYEKCGHS

FT

>AtbHLH123

MYQSSSSTSSSSQRSSLPGGGGLIRYGSAPGSFLNSVVDEVIGGGSSNARDFTGYQPSSD

NFIGNFFTGAADSSSLRSDSTTCGVNNSSDGQKQLGNNNNNNSNKDIFLDRSYGGFNEIS

QQHKSNDIGGGNSSGSYSLARQRSSPADFFTYLASDKNNFSLNQPTSDYSPQGGSNGGRG

HSRLKSQLSFTNHDSLARINEVNETPVHDGSGHSFSAASFGAATTDSWDDGSGSIGFTVT

RPSKRSKDMDSGLFSQYSLPSDTSMNYMDNFMQLPEDSVPCKIRAKRGCATHPRSIAERE

RRTRISGKLKKLQDLVPNMDKQTSYSDMLDLAVQHIKGLQHQLQNLKKDQENCTCGCSEK

PS

>AtbHLH118

MNNFQEKKRRRSKTPRVCNNEENMEKLVHKEIEKRRRQEMASLYASLRSLLPLEFIQGKR

STSDQVKGAVNYIDYLQRNIKDINSKRDDLVLLSGRSFRSSNEQEWNEISNHVVIRPCLV

GIEIVLSILQTPFSSVLQVLREHGLYVLGYICSSVNDRLIHTLQAEVQYLELTNFTAIEA

LLTLLRI

>AtbHLH134

MSSSRRSRQASSSSRISDDQITDLISKLRQSIPEIRQNRRSNTVSASKVLQETCNYIRNL

NKEADDLSDRLTQLLESIDPNSPQAAVIRSLING

>AtbHLH106

MQPETSDQMLYSFLAGNEVGGGGYCVSGDYMTTMQSLCGSSSSTSSYYPLAISGIGETMA

QDRALAALRNHKEAERRRRERINSHLNKLRNVLSCNSKTDKATLLAKVVQRVRELKQQTL

ETSDSDQTLLPSETDEISVLHFGDYSNDGHIIFKASLCCEDRSDLLPDLMEILKSLNMKT

LRAEMVTIGGRTRSVLVVAADKEMHGVESVHFLQNALKSLLERSSKSLMERSSGGGGGER

SKRRRALDHIIMV

>AtbHLH172

MERAREIGEGSASSLREQRNLREKERRMRMKHLFSILSSHVSPTRRLPVPQLIDQAVSYM

IQLKEKVNYLNEMKRRMLGGEVKNRSEGSSLLPKLSIRSLDSIIEMNLVMDLNMKGVMLH

KLVSVFEEEGAQVMSANLQNLNDRTFYTIIAQAIICRIGIDPSRIEERLRDIIS

>AtbHLH108

MNKDEVFLRQWFEILYSLTNPEANSDLRRINNEKGVEKVGQKRSAESRREGKKKRVKTQC

VIKSSDKSDHDTLLKKKRRERIRRQLETLKEITPNCPQSDINAILDCVIEYTNNLRLAHY

KGSQGICDDWRLFTEAGAVLYYIDT

>AtbHLH015

MHHFVPDFDTDDDYVNNHNSSLNHLPRKSITTMGEDDDLMELLWQNGQVVVQNQRLHTKK

PSSSPPKLLPSMDPQQQPSSDQNLFIQEDEMTSWLHYPLRDDDFCSDLLFSAAPTATATA

TVSQVTAARPPVSSTNESRPPVRNFMNFSRLRGDFNNGRGGESGPLLSKAVVRESTQVSP

SATPSAAASESGLTRRTDGTDSSAVAGGGAYNRKGKAVAMTAPAIEITGTSSSVVSKSEI

EPEKTNVDDRKRKEREATTTDETESRSEETKQARVSTTSTKRSRAAEVHNLSERKRRDRI

NERMKALQELIPRCNKSDKASMLDEAIEYMKSLQLQIQMMSMGCGMMPMMYPGMQQYMPH

MAMGMGMNQPIPPPSFMPFPNMLAAQRPLPTQTHMAGSGPQYPVHASDPSRVFVPNQQYD

PTSGQPQYPAGYTDPYQQFRGLHPTQPPQFQNQATSYPSSSRVSSSKESEDHGNHTTG

>AtbHLH034

MYPSIEDDDDLLAALCFDQSNGVEDPYGYMQTNEDNIFQDFGSCGVNLMQPQQEQFDSFN

GNLEQVCSSFRGGNNGVVYSSSIGSAQLDLAASFSGVLQQETHQVCGFRGQNDDSAVPHL

QQQQGQVFSGVVEINSSSSVGAVKEEFEEECSGKRRRTGSCSKPGTKACREKLRREKLND

KFMDLSSVLEPGRTPKTDKSAILDDAIRVVNQLRGEAHELQETNQKLLEEIKSLKADKNE

LREEKLVLKAEKEKMEQQLKSMVVPSPGFMPSQHPAAFHSHKMAVAYPYGYYPPNMPMWS

PLPPADRDTSRDLKNLPPVA

>AtbHLH123

MGDHHDFINSGSWWKVSSSSSPSSSSSMRASSIESGGSAVFHDKLHHHSLATDHHLQMIG

LGLSSQSPVDQWNQSLLRGDSKAETSFGVMLQENLNLDATSNANANTTSSTSSYQLQESD

SSHHHQALWRDPQSDFKPQILTSGGNRGFFLDHQFSPHGSSSTDSSTVTCQGFAVDNSSN

AMYAATTTTPNSSSGMFHHQQAGGFGSSDQQPSRNHQQSSLGYSQFGSSTGNYDQMASAL

PSTWFLRSSPPPKPHSPLRFSNNATFWNPAAAGNAGAPPPHDASSNFFPALQPPQIHPQS

FDEQPKNISEIRDSSSNEVKRGGNDHQPAAKRAKSEAASPSPAFKRKEKMGDRIAALQQL

VSPFGKTDAASVLSEAIEYIKFLHQQVSALSNPYMKSGASLQHQQSDHSTELEVSEEPDL

RSRGLCLVPVSSTFPVTHDTTVDFWTPTFGGTFR

>AtbHLH058

MDLSVLDRLKWLQQQQMVSPEFLQILGSDGREELKRVESYLGNNNDELQSFRHFPEFGPD

YDTTDGCISRTSSFHMEPVKNNGHSRAITLQNKRKPEGKTEKREKKKIKAEDETEPSMKG

KSNMSNTETSSEIQKPDYIHVRARRGEATDRHSLAERARREKISKKMKCLQDIVPGCNKV

TGKAGMLDEIINYVQSLQQQVEFLSMKLSVINPELECHIDDLSAKQFQAYFTGPPEGDSK

QSIMADFRSFPLHQQGSLDYSVINSDHTTSLGAKDHTSSSWETHSQCLYNSLRTDSVSNF

FSLK

>AtbHLH166

MEKTLATSHTKRSSPPSPSSAVNTSSTGFNRRTRQRLSDATASVSETDVEDEDEDEEGVE

EKIEALQTIVPGGTELGVDALFEETASYILALQCQINAIKVLTTFLERCEKKDMKFGG

>AtbHLH010

MEEERESLYEEMGCFDPNTPAEVTVESSFSQAEPPPPPPQVLVAGSTSNSNCSVEVEELS

EFHLSPQDCPQASSTPLQFHINPPPPPPPPCDQLHNNLIHQMASHQQQHSNWDNGYQDFV

NLGPNSATTPDLLSLLHLPRCSLPPNHHPSSMLPTSFSDIMSSSSAAAVMYDPLFHLNFP

MQPRDQNQLRNGSCLLGVEDQIQMDANGGMNVLYFEGANNNNGGFENEILEFNNGVTRKG

RGSRKSRTSPTERERRVHFNDRFFDLKNLIPNPTKIDRASIVGEAIDYIKELLRTIEEFK

MLVEKKRCGRFRSKKRARVGEGGGGEDQEEEEDTVNYKPQSEVDQSCFNKNNNNSLRCSW

LKRKSKVTEVDVRIIDDEVTIKLVQKKKINCLLFTTKVLDQLQLDLHHVAGGQIGEHYSF

LFNTKICEGSCVYASGIADTLMEVVEKQYMEAVPSNGY

>AtbHLH164

MSNRRSRQTSNASRISDDQMIDLVSKLRQFLPEIHERRRSDKVSASKVLQETCNYIRKLH

REVDNLSDRLSQLLDSVDEDSPEAAVIRSLLM

>AtbHLH063

MNGAIGGDLLLNFPDMSVLERQRAHLKYLNPTFDSPLAGFFADSSMITGGEMDSYLSTAG

LNLPMMYGETTVEGDSRLSISPETTLGTGNFKKRKFDTETKDCNEKKKKMTMNRDDLVEE

GEEEKSKITEQNNGSTKSIKKMKHKAKKEENNFSNDSSKVTKELEKTDYIHVRARRGQAT

DSHSIAERVRREKISERMKFLQDLVPGCDKITGKAGMLDEIINYVQSLQRQIEFLSMKLA

IVNPRPDFDMDDIFAKEVASTPMTVVPSPEMVLSGYSHEMVHSGYSSEMVNSGYLHVNPM

QQVNTSSDPLSCFNNGEAPSMWDSHVQNLYGNLGV

>AtbHLH081

MQPTSVGSSGGGDDGGGRGGGGGLSRSGLSRIRSAPATWLEALLEEDEEESLKPNLGLTD

LLTGNSNDLPTSRGSFEFPIPVEQGLYQQGGFHRQNSTPADFLSGSDGFIQSFGIQANYD

YLSGNIDVSPGSKRSREMEALFSSPEFTSQMKGEQSSGQVPTGVSSMSDMNMENLMEDSV

AFRVRAKRGCATHPRSIAERVRRTRISDRIRKLQELVPNMDKQTNTADMLEEAVEYVKVL

QRQIQELTEEQKRCTCIPKEEQ

>AtbHLH147

MESISPVSNQLLQPTTTSSNSDRSRRKRKKKSSPSSVEKSPSPSISLEKWRSEKQQQIYS

TKLVHALRELRISQQPSSSSSSSIPRGGRAVREVADRALAVAARGKTLWSRAILSKAVKL

KFRKHKRQRISNPTTTTLTTGSIRSKKQRATVLRLKAKGLPAVQRKVKVLSRLVPGCRKQ

SLPVVLEETTDYIAAMEMQIRTMTAILSAVSSSPPPPTPGHEGGQTHMLG

>AtbHLH135

MSGRRSRSRQSSGTSRISEDQINDLIIKLQQLLPELRDSRRSDKVSAARVLQDTCNYIRN

LHREVDDLSERLSELLANSDTAQAALIRSLLTQ

>AtbHLH139

MENEAFVDGELESLLGMFNFDQCSSNESSFCNAPNETDVFSSDDFFPFGTILQSNYAAVL

DGSNHQTNRNVDSRQDLLKPRKKQKLSSESNLVTEPKTAWRDGQSLSSYNSSDDEKALGL

VSNTSKSLKRKAKANRGIASDPQSLYARKRRERINDRLKTLQSLVPNGTKVDISTMLEDA

VHYVKFLQLQIKLLSSEDLWMYAPLAHNGLNMGLHHNLLSRLI

>AtbHLH136

MSNRRSRQSSSAPRISDNQMIDLVSKLRQILPEIGQRRRSDKASASKVLQETCNYIRNLN

REVDNLSERLSQLLESVDEDSPEAAVIRSLLM

>AtbHLH156

MGVLLREALRSMCVNNQWSYAVFWKIGCQNSSLLIWEECYNETESSSNPRRLCGLGVDTQ

GNEKVQLLTNRMMLNNRIILVGEGLVGRAAFTGHHQWILANSFNRDVHPPEVINEMLLQF

SAGIQTVAVFPVVPHGVVQLGSSLPIMENLGFVNDVKGLILQLGCVPGALLSENYRTYEP

AADFIGVPVSRIIPSQGHKILQSSAFVAETSKQHFNSTGSSDHQMVEESPCNLVDEHEGG

WQSTTGFLTAGEVAVPSNPDAWLNQNFSCMSNVDAAEQQQIPCEDISSKRSLGSDDLFDM

LGLDDKNKGCDNSWGVSQMRTEVLTRELSDFRIIQEMDPEFGSSGYELSGTDHLLDAVVS

GACSSTKQISDETSESCKTTLTKVSNSSVTTPSHSSPQGSQLFEKKHGQPLGPSSVYGSQ

ISSWVEQAHSLKREGSPRMVNKNETAKPANNRKRLKPGENPRPRPKDRQMIQDRVKELRE

IIPNGAKCSIDALLERTIKHMLFLQNVSKHSDKLKQTGESKIMKEDGGGATWAFEVGSKS

MVCPIVVEDINPPRIFQVEMLCEQRGFFLEIADWIRSLGLTILKGVIETRVDKIWARFTV

EASRDVTRMEIFMQLVNILEQTMKCGGNSKTILDGIKATMPLPVTGGCSM

>AtbHLH155

MGSTSQEILKSFCFNTDWDYAVFWQLNHRGSRMVLTLEDAYYDHHGTNMHGAHDPLGLAV

AKMSYHVYSLGEGIVGQVAVSGEHQWVFPENYNNCNSAFEFHNVWESQISAGIKTILVVA

VGPCGVVQLGSLCKVNEDVNFVNHIRHLFLALRDPLADHAANLRQCNMNNSLCLPKMPSE

GLHAEAFPDCSGEVDKAMDVEESNILTQYKTRRSDSMPYNTPSSCLVMEKAAQVVGGREV

VQGSTCGSYSGVTFGFPVDLVGAKHENQVGTNIIRDAPHVGMTSGCKDSRDLDPNLHLYM

KNHVLNDTSTSALAIEAERLITSQSYPRLDSTFQATSRTDKESSYHNEVFQLSENQGNKY

IKETERMLGRNCESSQFDALISSGYTFAGSELLEALGSAFKQTNTGQEELLKSEHGSTMR

PTDDMSHSQLTFDPGPENLLDAVVANVCQRDGNARDDMMSSRSVQSLLTNMELAEPSGQK

KHNIVNPINSAMNQPPMAEVDTQQNSSDICGAFSSIGFSSTYPSSSSDQFQTSLDIPKKN

KKRAKPGESSRPRPRDRQLIQDRIKELRELVPNGSKCSIDSLLERTIKHMLFLQNVTKHA

EKLSKSANEKMQQKETGMQGSSCAVEVGGHLQVSSIIVENLNKQGMVLIEMLCEECGHFL

EIANVIRSLDLVILRGFTETQGEKTWICFVTEVGSRITQFMKEIPKQIKSQNSKVMQRMD

ILWSLVQIFQPKANEKG

>AtbHLH049

MDLSAKDEFSAEKRNPDNYDSVNNPSGDWRVDSYPSENLISAGPASCSPSQMMDSFGQTLWYDPTSVQAVGYAGFNGGNASSSSFRGSIDRSLEMGWNLPNLLPPKGNGLFLPNASSFLP

PSMAQFPADSGFIERAARFSLFSGGNFSDMVNQPLGNSEAIGLFLQGGGTMQGQCQSNEL

NVGEPHNDVSVAVKESTVRSSEQAKPNVPGSGNVSEDTQSSGGNGQKGRETSSNTKKRKRNGQKNSEAAQSHRSQQSEEEPDNNGDEKRNDEQSPNSPGKKSNSGKQQGKQSSDPPKDGYIHVRARRGQATNSHSLAERVRREKISERMKFLQDLVPGCNKVTGKAVMLDEIINYVQSLQRQVEFLSMKLATVNPQMDFNLEGLLAKDALQLRAGSSSTTPFPPNMSMAYPPLPHGFMQQTLSSIGRTITSPLSPMNGGFKRQETNGWEGDLQNVIHINYGAGDVTPDPQAAATASLPAANMKVEP

>AtbHLH044

MANFENLSSDFQTIAMDIYSSITQAADLNNNNSNLHFQTFHPSSTSLESLFLHHHQQQLL

HFPGNSPDSSNNFSSTSSFLHSDHNIVDETKKRKALLPTLSSSETSGVSDNTNVIATETG

SLRRGKRLKKKKEEEDEKEREVVHVRARRGQATDSHSLAERVRRGKINERLRCLQDMVPGCYKAMGMATMLDEIINYVQSLQNQVEFLSMKLTAASSFYDFNSETDAVDSMQRAKARETVEMGRQTRDGSPVFHLSTWSL

>AtbHLH037

MDNSDILMNMMMQQMEKLPEHFSNSNPNPNPHNIMMLSESNTHPFFFNPTHSHLPFDQTMPHHQPGLNFRYAPSPSSSLPEKRGGCSDNANMAAMREMIFRIAVMQPIHIDPESVKPPKR

KNVRISKDPQSVAARHRRERISERIRILQRLVPGGTKMDTASMLDEAIHYVKFLKKQVQS

LEEHAVVNGGGMTAVAGGALAGTVGGGYGGKGCGIMRSDHHQMLGNAQILR
