# Supplementary material for: Comprehensive analysis of bHLH transcription factors reveals candidate regulators of flower development and heat stress response in Rhododendron simsii
Source: BMC Plant Biol. 2025 Dec 8;26:69. doi: 10.1186/s12870-025-07868-x (PMC12797679; doi:10.1186/s12870-025-07868-x)
Supplement: Supplementary file 1 — Supplementary Material 1: Supplementary Figure S1. Chromosome localization of RsbHLH gene family. Supplementary Figure S2. Sequence logos of RsbHLH proteins. Supplementary Figure S3. The Spearman’s corrlation coefficient of RNA-seq data and RT-qPCR. Supplementary Figure S4. The expression patterns of species-sepcific RsbHLH genes in different organs and in different stages of flower development. Supplementary Table S1. Protein sequences of bHLH genes from A. thaliana, R.simsii,R.williamsianum, and R.irroratum. Supplementary Table S2. The promoters of the bHLH genes in R. simsii.Supplementary Table S3. The primers of 12 candidate genes used in this study. Supplementary Table S4. Coding sequences of RsbHLH053 and RsbHLH059 cloned for GFP fusion and subcellular localization. Supplementary Table S5. Physicochemical properties and subcellular localization of bHLH protein in R. simsii.Supplementary Table S6. The distribution of RsbHLH genes on each chromosome in R. simsii. Supplementary Table S7. The classification of the bHLH genes inArabidopsis, R. simsii, R. irroratum and R. williamsianum based on the phylogenetic analysis. Supplementary Table S8. Conservative motifs of RsbHLH protein in R. simsii. Supplementary Table S9. The promoter Cis-element functional classification of RsbHLH genes. Supplementary Table S10. Segmentally duplicated RsbHLH gene pairs. Supplementary Table S11. One-to-one orthologous relationships between R. simsii and other plants. Supplementary Table S12. The expression patterns of RsbHLH family genes. Supplementary Table S13. The expression patterns of RsbHLH family genes in different stages of flower development. Supplementary Table S14. The protein interaction network. Supplementary Table S15. The RT-qPCR data of 12 RsbHLH genes in different stages of flower development. Supplementary Table S16. The RT-qPCR data of RsbHLH genes in high temperature-treated R. simsii. [file 12870_2025_7868_MOESM1_ESM.zip › Supplementary Materials/Table S11 .docx]

**Table S10 One-to-one orthologous relationships between *R. simsii* and other plants.**

| **Seq_1** | **Seq_2** | **Ka** | **Ks** | **Ka_Ks** | **Divergence-Time**（Mya*） |
| --- | --- | --- | --- | --- | --- |
| RsbHLH009 | AtbHLH007 | 0.18646860643895197 | 2.818689000263031 | 0.06615437404465385 | 231.040082 |
| RsbHLH003 | AtbHLH070 | 0.447582 | 2.91732 | 0.153422 | 239.1245902 |
| RsbHLH008 | AtbHLH015 | 0.36782693575995984 | 2.2922509917026295 | 0.1604653840663176 | 187.8894255 |
| RsbHLH009 | AtbHLH059 | 0.2062619912333457 | 2.189593872148488 | 0.09420102689224095 | 179.4749076 |
| RsbHLH007 | AtbHLH014 | 0.4760957205767481 | 3.4585505518038797 | 0.13765758616089344 | 283.4877501 |
| RsbHLH008 | AtbHLH023 | 0.536578 | 2.51772 | 0.213121 | 206.3704918 |
| RsbHLH017 | AtbHLH002 | 0.363111 | 3.24708 | 0.111827 | 266.1540984 |
| RsbHLH010 | AtbHLH013 | 0.573343 | 2.32965 | 0.246107 | 190.954918 |
| RsbHLH011 | AtbHLH022 | 0.4978830908932216 | 2.072377125242472 | 0.24024733955455543 | 169.8669775 |
| RsbHLH017 | AtbHLH001 | 0.390551 | 3.09822 | 0.126057 | 253.952459 |
| RsbHLH015 | AtbHLH092 | 0.509815 | 2.69581 | 0.189114 | 220.9680328 |
| RsbHLH024 | AtbHLH125 | 0.6378840040350244 | 1.8866539900518773 | 0.3381033339438593 | 154.6437697 |
| RsbHLH024 | AtbHLH055 | 0.643643 | 2.15883 | 0.298144 | 176.9532787 |
| RsbHLH019 | AtbHLH051 | 0.46507088940003566 | 4.401972684850294 | 0.10565056230371685 | 360.8174332 |
| RsbHLH021 | AtbHLH130 | 0.37707 | 3.16412 | 0.119171 | 259.3540984 |
| RsbHLH023 | AtbHLH048 | 0.321646 | 3.30977 | 0.0971809 | 271.292623 |
| RsbHLH023 | AtbHLH060 | 0.3192056454361331 | 2.9091720353471957 | 0.10972388073228452 | 238.4567242 |
| RsbHLH024 | AtbHLH118 | 0.5315554221764166 | 1.7991581933613672 | 0.2954467395572991 | 147.4719831 |
| RsbHLH018 | AtbHLH027 | 0.3058895203519993 | 1.521168725075674 | 0.2010884889424624 | 124.6859611 |
| RsbHLH024 | AtbHLH036 | 0.4393841372762203 | 2.2462392947251626 | 0.19560878411664545 | 184.117975 |
| RsbHLH018 | AtbHLH035 | 0.28674023857562553 | 1.6784829340224983 | 0.17083297825879598 | 137.5805684 |
| RsbHLH028 | AtbHLH117 | 0.68484 | 2.00785 | 0.341082 | 164.5778689 |
| RsbHLH036 | AtbHLH008 | 0.5172126901190095 | 1.6945814533796697 | 0.3052155970947762 | 138.9001191 |
| RsbHLH031 | AtbHLH021 | 0.550652 | 2.65201 | 0.207635 | 217.3778689 |
| RsbHLH037 | AtbHLH041 | 0.495671 | 2.72076 | 0.182181 | 223.0131148 |
| RsbHLH038 | AtbHLH142 | 0.576715 | 2.61385 | 0.220638 | 214.25 |
| RsbHLH038 | AtbHLH143 | 0.5233870630425876 | 2.2054161742441307 | 0.23731895555811353 | 180.7718176 |
| RsbHLH046 | AtbHLH094 | 0.491716 | 2.86572 | 0.171586 | 234.895082 |
| RsbHLH041 | AtbHLH074 | 0.34843237421669493 | 2.088308454164121 | 0.16684909430975828 | 171.1728241 |
| RsbHLH050 | AtbHLH037 | 0.416159 | 2.98618 | 0.139362 | 244.7688525 |
| RsbHLH050 | AtbHLH088 | 0.355714 | 3.33214 | 0.106752 | 273.1262295 |
| RsbHLH043 | AtbHLH093 | 0.35288065089165616 | 4.216658115721729 | 0.08368728059217023 | 345.6277144 |
| RsbHLH046 | AtbHLH099 | 0.46730387669294465 | 2.9535693294508714 | 0.15821666078169425 | 242.0958467 |
| RsbHLH059 | AtbHLH013 | 0.307092 | 3.3918 | 0.0905397 | 278.0163934 |
| RsbHLH053 | AtbHLH006 | 0.275728 | 3.54972 | 0.0776761 | 290.9606557 |
| RsbHLH055 | AtbHLH125 | 0.6083233429333829 | 3.0953507887838088 | 0.19652807854207652 | 253.7172778 |
| RsbHLH055 | AtbHLH055 | 0.619187 | 2.28243 | 0.271285 | 187.0844262 |
| RsbHLH054 | AtbHLH002 | 0.44785885670559206 | 3.3644106858652956 | 0.13311658371171975 | 275.7713677 |
| RsbHLH060 | AtbHLH054 | 0.485636 | 2.74343 | 0.177018 | 224.8713115 |
| RsbHLH059 | AtbHLH017 | 0.35514248354124184 | 3.8020274763105997 | 0.09340871041938496 | 311.6415964 |
| RsbHLH054 | AtbHLH012 | 0.518752 | 2.96087 | 0.175203 | 242.6942623 |
| RsbHLH053 | AtbHLH004 | 0.286709 | 3.58034 | 0.0800785 | 293.4704918 |
| RsbHLH056 | AtbHLH016 | 0.233159 | 3.54766 | 0.0657219 | 290.7918033 |
| RsbHLH053 | AtbHLH028 | 0.41384103092479 | 2.9979340896903337 | 0.13804207115425177 | 245.7323024 |
| RsbHLH054 | AtbHLH001 | 0.493452 | 2.88689 | 0.170929 | 236.6303279 |
| RsbHLH060 | AtbHLH139 | 0.504992 | 3.02715 | 0.166821 | 248.1270492 |
| RsbHLH053 | AtbHLH005 | 0.3540851718705267 | 2.810582347582821 | 0.12598284913268945 | 230.3756023 |
| RsbHLH052 | AtbHLH071 | 0.376694 | 2.86274 | 0.131585 | 234.6508197 |
| RsbHLH055 | AtbHLH036 | 0.5116493791369671 | 2.8262163576665476 | 0.18103687559129691 | 231.6570785 |
| RsbHLH075 | AtbHLH031 | 0.42206 | 2.77589 | 0.152045 | 227.5319672 |
| RsbHLH068 | AtbHLH083 | 0.41486442730890805 | 3.7907130440739154 | 0.10944231929068661 | 310.7141839 |
| RsbHLH071 | AtbHLH102 | 0.46464381013200307 | 1.9662491901775405 | 0.23630972740034467 | 161.1679664 |
| RsbHLH073 | AtbHLH018 | 0.597000251112191 | 3.365179699120721 | 0.17740516242510904 | 275.8344016 |
| RsbHLH074 | AtbHLH087 | 0.3912180468862444 | 1.7936030022709715 | 0.21811852811960253 | 147.0166395 |
| RsbHLH070 | AtbHLH024 | 0.5573849240270929 | 2.509079568781714 | 0.22214716940911194 | 205.6622597 |
| RsbHLH073 | AtbHLH025 | 0.494232 | 2.66438 | 0.185496 | 218.3918033 |
| RsbHLH066 | AtbHLH137 | 0.54277 | 2.12059 | 0.255953 | 173.8188525 |
| RsbHLH062 | AtbHLH099 | 0.527037 | 2.63379 | 0.200106 | 215.8844262 |
| RsbHLH071 | AtbHLH046 | 0.6250975027163022 | 2.6531459125694834 | 0.2356061533422849 | 217.4709764 |
| RsbHLH071 | AtbHLH141 | 0.5782542738153263 | 4.364014162659147 | 0.13250513226175595 | 357.7060789 |
| RsbHLH068 | AtbHLH086 | 0.46962951571484235 | 5.329093453360732 | 0.08812559205894124 | 436.8109388 |
| RsbHLH070 | AtbHLH073 | 0.41347479877032384 | 4.571868421824287 | 0.09043891044557606 | 374.7433133 |
| RsbHLH072 | AtbHLH046 | 0.34471724595377784 | 4.755269477795933 | 0.07249163219106444 | 389.7761867 |
| RsbHLH075 | AtbHLH079 | 0.29071425194324707 | 2.452369823297082 | 0.1185442135119723 | 201.0139199 |
| RsbHLH076 | AtbHLH078 | 0.40628800166180434 | 1.6458769210236954 | 0.24685199511097286 | 134.9079443 |
| RsbHLH076 | AtbHLH049 | 0.4180647634928514 | 1.6551127327707367 | 0.25258990231619527 | 135.6649781 |
| RsbHLH077 | AtbHLH102 | 0.34734 | 3.32066 | 0.1046 | 272.1852459 |
| RsbHLH080 | AtbHLH125 | 0.657625 | 2.16333 | 0.303987 | 177.3221311 |
| RsbHLH080 | AtbHLH055 | 0.6165706034482573 | 2.4736299867490184 | 0.24925740985966482 | 202.7565563 |
| RsbHLH083 | AtbHLH030 | 0.26741625293145277 | 2.5246234561097696 | 0.10592322284112757 | 206.9363489 |
| RsbHLH083 | AtbHLH032 | 0.30942050735979765 | 2.707754720433177 | 0.11427198520783952 | 221.9471082 |
| RsbHLH085 | AtbHLH097 | 0.24253202930031983 | 2.29636512473571 | 0.10561562126503424 | 188.2266496 |
| RsbHLH080 | AtbHLH118 | 0.42812087546756217 | 2.0038853643286916 | 0.2136453926400047 | 164.2528987 |
| RsbHLH080 | AtbHLH036 | 0.495175 | 2.66378 | 0.185892 | 218.342623 |
| RsbHLH084 | AtbHLH098 | 0.2588817456059429 | 1.8317121085168582 | 0.1413332064587159 | 150.1403368 |
| RsbHLH090 | AtbHLH037 | 0.3902003089776819 | 1.669533761903787 | 0.23371813010401918 | 136.8470297 |
| RsbHLH089 | AtbHLH011 | 0.421101 | 2.94732 | 0.142876 | 241.5836066 |
| RsbHLH088 | AtbHLH099 | 0.57282 | 2.47001 | 0.23191 | 202.4598361 |
| RsbHLH087 | AtbHLH093 | 0.511057 | 2.60838 | 0.195929 | 213.8016393 |
| RsbHLH090 | AtbHLH088 | 0.42042660792592434 | 1.9099700714594083 | 0.22012209207271835 | 156.5549239 |
| RsbHLH092 | AtbHLH095 | 0.38454766600974133 | 2.191025893698831 | 0.1755103246911237 | 179.5922864 |
| RsbHLH094 | AtbHLH064 | 0.580245 | 2.60838 | 0.222454 | 213.8016393 |
| RsbHLH094 | AtbHLH063 | 0.446258 | 3.04002 | 0.146794 | 249.1819672 |
| RsbHLH094 | AtbHLH058 | 0.543242 | 2.94117 | 0.184703 | 241.0795082 |
| RsbHLH093 | AtbHLH071 | 0.426382 | 2.80308 | 0.152112 | 229.7606557 |
| RsbHLH100 | AtbHLH102 | 0.3181451218092135 | 2.9395561909720413 | 0.10822896421789796 | 240.9472288 |
| RsbHLH097 | AtbHLH032 | 0.284232841804118 | 2.5330958263224765 | 0.11220769417822003 | 207.6308054 |
| RsbHLH099 | AtbHLH137 | 0.4526033550195717 | 2.2381312967232088 | 0.20222377287794366 | 183.453385 |
| RsbHLH096 | AtbHLH079 | 0.393786 | 2.95906 | 0.133078 | 242.5459016 |
| RsbHLH109 | AtbHLH112 | 0.46587322482285376 | 2.0844409855454797 | 0.2235003188161448 | 170.8558185 |
| RsbHLH104 | AtbHLH122 | 0.548943317084406 | 2.5368789364874678 | 0.21638530289682106 | 207.9408964 |
| RsbHLH108 | AtbHLH052 | 0.633442 | 2.15664 | 0.293717 | 176.7737705 |
| RsbHLH108 | AtbHLH053 | 0.6440623328949803 | 4.164719688691147 | 0.15464722263154063 | 341.3704663 |
| RsbHLH110 | AtbHLH029 | 0.438522 | 2.79834 | 0.156708 | 229.3721311 |
| RsbHLH106 | AtbHLH015 | 0.55789 | 2.48512 | 0.224492 | 203.6983607 |
| RsbHLH105 | AtbHLH062 | 0.4382426457806817 | 2.9603357439295017 | 0.14803815637444068 | 242.6504708 |
| RsbHLH106 | AtbHLH023 | 0.623889 | 2.2421 | 0.27826 | 183.7786885 |
| RsbHLH109 | AtbHLH103 | 0.5079044367200828 | 2.596667733355726 | 0.1955985473981716 | 212.8416175 |
| RsbHLH105 | AtbHLH076 | 0.4543779577574226 | 2.206668839629785 | 0.20591125845310532 | 180.8744951 |
| RsbHLH008 | RibHLH47 | 0.2351263087368421 | 0.788519552863174 | 0.29818703655867596 | 64.63275023 |
| RsbHLH007 | RibHLH78 | 0.01359150535351776 | 0.04695581800873132 | 0.28945306311116664 | 3.848837542 |
| RsbHLH008 | RibHLH75 | 0.02049074518054165 | 0.0541545707678874 | 0.37837517480043 | 4.438899243 |
| RsbHLH004 | RibHLH74 | 0.025836515109959414 | 0.0764610051544611 | 0.33790446591391676 | 6.267295504 |
| RsbHLH009 | RibHLH73 | 0.010983459891148298 | 0.06221181223556318 | 0.17654942841979518 | 5.099328872 |
| RsbHLH006 | RibHLH72 | 0.015110055261144421 | 0.04535386631626309 | 0.33315914360593823 | 3.717530026 |
| RsbHLH005 | RibHLH80 | 0.02378298638492967 | 0.04957205307446646 | 0.47976601552498127 | 4.063283039 |
| RsbHLH001 | RibHLH79 | 0.04151713195524193 | 0.10521422976756475 | 0.39459616866425756 | 8.624117194 |
| RsbHLH009 | RibHLH92 | 0.23228021653036682 | 1.202252445167487 | 0.19320419556144686 | 98.54528239 |
| RsbHLH013 | RibHLH81 | 0.035136944931549674 | 0.1079410354141952 | 0.3255198062231007 | 8.847625854 |
| RsbHLH012 | RibHLH84 | 0.0045011387915234145 | 0.07487509391676023 | 0.06011530077715026 | 6.13730278 |
| RsbHLH017 | RibHLH88 | 0.029217742497737252 | 0.09321152665801437 | 0.3134563239687599 | 7.64028907 |
| RsbHLH011 | RibHLH86 | 0.07023392000624071 | 0.12088650286658278 | 0.580990584893955 | 9.908729743 |
| RsbHLH012 | RibHLH92 | 0.11656227582715345 | 0.6802883988356861 | 0.17134244245036345 | 55.76134417 |
| RsbHLH019 | RibHLH25 | 0.02664905149607952 | 0.02758227074365938 | 0.9661659746489731 | 2.260841864 |
| RsbHLH025 | RibHLH26 | 0.05909770540209655 | 0.14616901254687578 | 0.4043107658207937 | 11.9810666 |
| RsbHLH026 | RibHLH31 | 0.05273671253161067 | 0.09285307565201237 | 0.5679587042356387 | 7.61090784 |
| RsbHLH018 | RibHLH33 | 0.0034512571686559444 | 0.06648869366252219 | 0.051907429346913475 | 5.449892923 |
| RsbHLH021 | RibHLH23 | 0.018855083301341304 | 0.09398278124397234 | 0.20062274229142998 | 7.703506659 |
| RsbHLH023 | RibHLH24 | 0.015461144474228819 | 0.07111170186090837 | 0.21742053796532948 | 5.828828021 |
| RsbHLH025 | RibHLH70 | 0.40562226136076085 | 1.5769994960334106 | 0.2572114083619005 | 129.2622538 |
| RsbHLH027 | RibHLH73 | 0.23877177076792735 | 1.172786747322001 | 0.2035935103403501 | 96.13006126 |
| RsbHLH027 | RibHLH92 | 0.004448851403702223 | 0.029755968680746772 | 0.14951122752662382 | 2.439013826 |
| RsbHLH036 | RibHLH02 | 0.19570167775231978 | 0.5769416561368015 | 0.33920531767932527 | 47.29029968 |
| RsbHLH033 | RibHLH03 | 0.22176746064408787 | 0.801347684816069 | 0.2767431226746347 | 65.68423646 |
| RsbHLH031 | RibHLH109 | 0.012147551393732914 | 0.07081423264197417 | 0.17154110043314397 | 5.804445299 |
| RsbHLH033 | RibHLH112 | 0.053606350470721945 | 0.060663609739843874 | 0.8836656885505658 | 4.972427028 |
| RsbHLH037 | RibHLH108 | 0.03834392019655081 | 0.0883849396700784 | 0.43382866288849947 | 7.244667186 |
| RsbHLH036 | RibHLH115 | 0.025086495466031488 | 0.038912023830810535 | 0.6446977822358344 | 3.18951015 |
| RsbHLH049 | RibHLH06 | 0.019324740573171885 | 0.052443916012647085 | 0.3684839356487345 | 4.29868164 |
| RsbHLH041 | RibHLH07 | 0.005827535146448841 | 0.036642293645983474 | 0.15903849258867622 | 3.003466692 |
| RsbHLH046 | RibHLH10 | 0.02319865989187218 | 0.10331492798555843 | 0.224543155032881 | 8.46843672 |
| RsbHLH047 | RibHLH11 | 0.0017211711512538851 | 0.03523152623505312 | 0.04885315327445081 | 2.887830019 |
| RsbHLH042 | RibHLH13 | 0.07245924640006263 | 0.1692539982506764 | 0.42810951084739324 | 13.87327855 |
| RsbHLH044 | RibHLH15 | 0.02997754313404663 | 0.030836820293227887 | 0.9721347028970441 | 2.527608221 |
| RsbHLH044 | RibHLH16 | 0.048209363553423604 | 0.09293641699420889 | 0.5187349062147258 | 7.617739098 |
| RsbHLH044 | RibHLH17 | 0.23531874477576056 | 1.7593670790365228 | 0.13375193135057836 | 144.2104163 |
| RsbHLH046 | RibHLH19 | 0.3378755489812768 | 1.6284620890186583 | 0.20748137230808175 | 133.4804991 |
| RsbHLH041 | RibHLH56 | 0.4534193422316465 | 2.0575039093783185 | 0.2203735021668312 | 168.6478614 |
| RsbHLH046 | RibHLH49 | 0.2987578060441584 | 1.3248996797117825 | 0.22549466244051772 | 108.5983344 |
| RsbHLH044 | RibHLH95 | 0.17285566938954955 | 0.6487956926278732 | 0.2664254269158559 | 53.17997481 |
| RsbHLH059 | RibHLH97 | 0.007443306347675028 | 0.06175797563403232 | 0.12052380718861068 | 5.06212915 |
| RsbHLH056 | RibHLH98 | 0.03439530747648464 | 0.09334290983571193 | 0.36848334315934717 | 7.651058183 |
| RsbHLH057 | RibHLH99 | 0.005522767917721662 | 0.039278132249241614 | 0.14060668370574816 | 3.219519037 |
| RsbHLH058 | RibHLH100 | 0.010489681481054879 | 0.028695160214789312 | 0.36555577325714184 | 2.352062313 |
| RsbHLH061 | RibHLH101 | 0.051603820914961185 | 0.059247046557615354 | 0.8709939805147681 | 4.856315292 |
| RsbHLH055 | RibHLH104 | 0.06702865393209163 | 0.17125533366105156 | 0.39139600793254536 | 14.03732243 |
| RsbHLH053 | RibHLH105 | 0.10708622533219177 | 0.21534980576961443 | 0.4972664124283203 | 17.65162342 |
| RsbHLH052 | RibHLH106 | 0.019700589673559223 | 0.12372603983983298 | 0.15922751345684563 | 10.14147868 |
| RsbHLH060 | RibHLH107 | 0.0400763110920191 | 0.07311134980306544 | 0.5481544411362894 | 5.99273359 |
| RsbHLH062 | RibHLH10 | 0.35300141318086026 | 1.4468889303220327 | 0.243972709848774 | 118.5974533 |
| RsbHLH065 | RibHLH09 | 0.5107324837047675 | 1.8498334402112036 | 0.27609647041868535 | 151.6256918 |
| RsbHLH070 | RibHLH06 | 0.41479632627860474 | 1.6467340663920365 | 0.2518902928797822 | 134.9782022 |
| RsbHLH062 | RibHLH19 | 0.22244134253595066 | 0.9855850634069088 | 0.2256947175792512 | 80.78566093 |
| RsbHLH068 | RibHLH22 | 0.20283745171509837 | 0.8570272174518259 | 0.23667562427969216 | 70.24813258 |
| RsbHLH075 | RibHLH36 | 0.2641065546890589 | 1.5413181388194896 | 0.17135109750368646 | 126.3375524 |
| RsbHLH066 | RibHLH34 | 0.3008776378689628 | 0.7973741547542703 | 0.3773355783793687 | 65.35853727 |
| RsbHLH062 | RibHLH49 | 0.03334345448885057 | 0.06654235687557404 | 0.5010861660821347 | 5.454291547 |
| RsbHLH065 | RibHLH50 | 0.011437870605245436 | 0.04596938836779259 | 0.24881493992770026 | 3.767982653 |
| RsbHLH071 | RibHLH53 | 0.016009145715167334 | 0.02197959493912452 | 0.7283640012250837 | 1.801606143 |
| RsbHLH068 | RibHLH54 | 0.020050030926930217 | 0.0466395992844692 | 0.4298928643155559 | 3.822917974 |
| RsbHLH073 | RibHLH55 | 0.013998285464251817 | 0.08999546888359014 | 0.15554433615273133 | 7.376677777 |
| RsbHLH064 | RibHLH56 | 0.021595648841424345 | 0.07874561114036938 | 0.2742457456191259 | 6.45455829 |
| RsbHLH070 | RibHLH57 | 0.012273635135024299 | 0.048209363553423604 | 0.25459027521537736 | 3.951587177 |
| RsbHLH075 | RibHLH61 | 0.02425499775839501 | 0.06382776881973733 | 0.3800069813954501 | 5.231784329 |
| RsbHLH066 | RibHLH62 | 0.0698954697788494 | 0.12042316492370694 | 0.5804154858671177 | 9.870751223 |
| RsbHLH067 | RibHLH64 | 0.03596947335938553 | 0.07842960293968279 | 0.45862113298021256 | 6.428655979 |
| RsbHLH072 | RibHLH59 | 0.0169319360389362 | 0.04734581402591502 | 0.35762266184014496 | 3.880804428 |
| RsbHLH067 | RibHLH51 | 0.28548579468677693 | 1.4146662392801612 | 0.20180434561868354 | 115.9562491 |
| RsbHLH080 | RibHLH26 | 0.41590279264230523 | 1.4100390572803012 | 0.29495834919956265 | 115.5769719 |
| RsbHLH083 | RibHLH38 | 0.17799892469157283 | 0.9554609956222466 | 0.18629637997483145 | 78.31647505 |
| RsbHLH077 | RibHLH39 | 0.22912587681210572 | 0.6350875431918585 | 0.36077841435930563 | 52.056356 |
| RsbHLH080 | RibHLH70 | 0.02709098131049572 | 0.041437465139493895 | 0.6537798878212607 | 3.396513536 |
| RsbHLH084 | RibHLH71 | 0.016665571932159014 | 0.06066097979653669 | 0.27473298301572274 | 4.972211459 |
| RsbHLH083 | RibHLH67 | 0.008518785385137563 | 0.07407331100430925 | 0.11500478741448426 | 6.071582869 |
| RsbHLH076 | RibHLH68 | 0.02805867383966142 | 0.05560263082303527 | 0.5046285296996624 | 4.55759269 |
| RsbHLH077 | RibHLH69 | 0.02467163226561566 | 0.05778752464496747 | 0.4269369975127362 | 4.736682348 |
| RsbHLH085 | RibHLH66 | 0.014161640790997486 | 0.0810556661470669 | 0.17471500098836634 | 6.643907061 |
| RsbHLH081 | RibHLH65 | 0.030316726032088587 | 0.09840089101500013 | 0.30809401946845316 | 8.065646805 |
| RsbHLH080 | RibHLH104 | 0.5791158846130748 | 3.07786747963251 | 0.18815491194644282 | 252.2842196 |
| RsbHLH088 | RibHLH10 | 0.3435151608947096 | 1.7749416508190887 | 0.19353603017664633 | 145.4870206 |
| RsbHLH088 | RibHLH19 | 0.015440586515760996 | 0.08429916386027739 | 0.18316417160854848 | 6.90976753 |
| RsbHLH089 | RibHLH18 | 0.010834704096268235 | 0.037931333693063236 | 0.2856399456961265 | 3.109125713 |
| RsbHLH087 | RibHLH20 | 0.045088700290107434 | 0.06931139735264367 | 0.6505236081261556 | 5.681262078 |
| RsbHLH088 | RibHLH49 | 0.2016399045009398 | 1.0677315895289676 | 0.18884887033256526 | 87.51898275 |
| RsbHLH086 | RibHLH55 | 0.22657092610385676 | 1.1809741995749914 | 0.1918508687026313 | 96.8011639 |
| RsbHLH091 | RibHLH02 | 0.018445833547346774 | 0.030236234217932166 | 0.6100572384244571 | 2.478379854 |
| RsbHLH095 | RibHLH03 | 0.02584410748448937 | 0.12458434875592862 | 0.20744265024108433 | 10.21183187 |
| RsbHLH092 | RibHLH04 | 0.03109001753503365 | 0.15434269751303997 | 0.20143497577789155 | 12.65104078 |
| RsbHLH094 | RibHLH05 | 0.058700649154668466 | 0.1379702443073784 | 0.4254587606867727 | 11.30903642 |
| RsbHLH093 | RibHLH01 | 0.04206031907068888 | 0.08621922958482309 | 0.4878299107197384 | 7.067149966 |
| RsbHLH093 | RibHLH106 | 0.19028034463178767 | 0.9865167416935311 | 0.19288100909989392 | 80.86202801 |
| RsbHLH091 | RibHLH115 | 0.2094295904897821 | 0.5648329839956283 | 0.37078144588560896 | 46.29778557 |
| RsbHLH096 | RibHLH36 | 0.004770755550909587 | 0.06777892362280671 | 0.07038700669634551 | 5.555649477 |
| RsbHLH099 | RibHLH34 | 0.014216903882841164 | 0.05802328465688587 | 0.24502066656362556 | 4.756006939 |
| RsbHLH103 | RibHLH41 | 0.012371414526833175 | 0.05891152730317199 | 0.20999989464145258 | 4.828813713 |
| RsbHLH100 | RibHLH39 | 0.012120115626193972 | 0.03827055777957875 | 0.31669555735247973 | 3.136930966 |
| RsbHLH097 | RibHLH38 | 0.016433538024412997 | 0.10149278287583674 | 0.16191829171259695 | 8.319080564 |
| RsbHLH099 | RibHLH62 | 0.22687407039951824 | 0.6987704100081362 | 0.3246761270227178 | 57.27626312 |
| RsbHLH096 | RibHLH61 | 0.25604252899803776 | 1.4279075755115698 | 0.17931309658211358 | 117.0416046 |
| RsbHLH100 | RibHLH69 | 0.22622176597068605 | 0.5392811569343988 | 0.41948761432101167 | 44.20337352 |
| RsbHLH097 | RibHLH67 | 0.16401270363975604 | 1.0354050992524764 | 0.1584043808149748 | 84.86927043 |
| RsbHLH107 | RibHLH43 | 0.009972446093278747 | 0.08515753305268539 | 0.11710585940892602 | 6.98012566 |
| RsbHLH111 | RibHLH48 | 0.01436672640537473 | 0.09746312876192731 | 0.14740678436938198 | 7.988781046 |
| RsbHLH106 | RibHLH47 | 0.02256487888112505 | 0.01773481647496706 | 1.2723491620550849 | 1.453673482 |
| RsbHLH105 | RibHLH46 | 0.012998159027836182 | 0.03931811489743185 | 0.33058957841046405 | 3.222796303 |
| RsbHLH110 | RibHLH45 | 0.016463448296520616 | 0.046871946453998364 | 0.35124311111505413 | 3.841962824 |
| RsbHLH104 | RibHLH42 | 0.04099778533575422 | 0.099407588429628 | 0.4124210835752958 | 8.148162986 |
| RsbHLH106 | RibHLH75 | 0.2159623844377729 | 0.7813006609513518 | 0.27641392773789036 | 64.04103778 |
| RsbHLH105 | RibHLH76 | 0.2319611908538323 | 0.8594185499484542 | 0.2699047988523691 | 70.44414344 |
| RsbHLH007 | RsbHLH010 | 0.3551389285148848 | 1.7017435317794236 | 0.20869121690948034 | 139.4871747 |
| RsbHLH008 | RsbHLH106 | 0.21117681914711733 | 0.8668169892722324 | 0.2436233042968141 | 71.05057289 |
| RsbHLH009 | RsbHLH027 | 0.22900200074953087 | 1.1866896715380384 | 0.19297547306763627 | 97.26964521 |
| RsbHLH012 | RsbHLH027 | 0.11839023695933873 | 0.6918001236965285 | 0.17113358743958954 | 56.70492817 |
| RsbHLH017 | RsbHLH054 | 0.36254690359173525 | 1.6345906722473957 | 0.2217967530019428 | 133.982842 |
| RsbHLH025 | RsbHLH055 | 0.6274215339319562 | 1.988401002652553 | 0.3155407450986837 | 162.9836887 |
| RsbHLH025 | RsbHLH080 | 0.41330395535018993 | 1.4543478465524557 | 0.28418507740767146 | 119.2088399 |
| RsbHLH026 | RsbHLH037 | 0.28347663685367364 | 0.8719078721354281 | 0.3251222358612252 | 71.46785837 |
| RsbHLH033 | RsbHLH095 | 0.20324760427950167 | 0.8453129720620653 | 0.24044065452313754 | 69.28794853 |
| RsbHLH036 | RsbHLH091 | 0.20436888289544125 | 0.576070568518128 | 0.354763624569739 | 47.21889906 |
| RsbHLH038 | RsbHLH048 | 0.48581576172243013 | 1.53078622548493 | 0.3173635571279922 | 125.4742808 |
| RsbHLH041 | RsbHLH064 | 0.46486497381819564 | 2.189070570869132 | 0.21235723507699852 | 179.432014 |
| RsbHLH045 | RsbHLH116 | 0.2739255590927299 | 1.1385169326684828 | 0.2405985815693551 | 93.32106005 |
| RsbHLH046 | RsbHLH062 | 0.3355353303805496 | 1.306066991299845 | 0.2569051454601213 | 107.0546714 |
| RsbHLH046 | RsbHLH088 | 0.33679190675598547 | 1.8653200492234916 | 0.18055448816742603 | 152.895086 |
| RsbHLH047 | RsbHLH107 | 0.3572622079740852 | 0.8972446041783423 | 0.39817704816542243 | 73.54463969 |
| RsbHLH049 | RsbHLH070 | 0.41044479356408337 | 1.5410867900803904 | 0.26633463877960606 | 126.3185894 |
| RsbHLH050 | RsbHLH090 | 0.3774684259463099 | 1.1197180092991479 | 0.33711025705710884 | 91.7801647 |
| RsbHLH052 | RsbHLH093 | 0.19602106724964313 | 1.0091287651454859 | 0.19424782447994415 | 82.71547255 |
| RsbHLH055 | RsbHLH080 | 0.607039 | 2.19837 | 0.276131 | 180.1942623 |
| RsbHLH058 | RsbHLH104 | 0.29026869594373056 | 1.3222195195559188 | 0.2195313952415559 | 108.3786491 |
| RsbHLH062 | RsbHLH088 | 0.22490255877981669 | 1.0783780890080445 | 0.2085563134787867 | 88.39164664 |
| RsbHLH063 | RsbHLH067 | 0.32747951650434837 | 1.3880161115884324 | 0.23593351242125277 | 113.7718124 |
| RsbHLH066 | RsbHLH099 | 0.29834904606901747 | 0.8968700092557522 | 0.33265583974270235 | 73.51393518 |
| RsbHLH073 | RsbHLH086 | 0.23278529605385945 | 1.1956229719121518 | 0.19469791190241809 | 98.00188294 |
| RsbHLH074 | RsbHLH102 | 0.27348233519093196 | 0.9752013728771388 | 0.28043678238893 | 79.93453876 |
| RsbHLH075 | RsbHLH096 | 0.2597704334670342 | 1.5073586124730314 | 0.17233485868425477 | 123.5539846 |
| RsbHLH077 | RsbHLH100 | 0.2361518014009213 | 0.6353489810737184 | 0.37168832946238883 | 52.07778533 |
| RsbHLH083 | RsbHLH097 | 0.1639665499465229 | 1.0572507842288972 | 0.15508765979881625 | 86.65990035 |
| RwbHLH35 | RsbHLH043 | 0.015137467239952226 | 0.06404631330909623 | 0.2363518906528897 | 5.249697812 |
| RwbHLH33 | RsbHLH041 | 0.0058422885490001086 | 0.03259168588759048 | 0.17925702184140777 | 2.671449663 |
| RwbHLH32 | RsbHLH050 | 0.015387621012699113 | 0.1532200983090856 | 0.10042821524404846 | 12.55902445 |
| RwbHLH41 | RsbHLH044 | 0.028744008105494274 | 0.04205305622792499 | 0.683517696067166 | 3.446971822 |
| RwbHLH42 | RsbHLH044 | 0.34921427319593346 | 2.006445283709423 | 0.17404624787490955 | 164.4627282 |
| RwbHLH34 | RsbHLH073 | 0.4942538193652055 | NaN | NaN | - |
| RwbHLH33 | RsbHLH064 | 0.45536529331583603 | 2.359728849025013 | 0.19297356707063304 | 193.4203975 |
| RwbHLH37 | RsbHLH062 | 0.33853191229990287 | 1.335349493074911 | 0.25351558828270865 | 109.4548765 |
| RwbHLH36 | RsbHLH065 | 0.5231851990490949 | 1.8415184157425264 | 0.2841053310010691 | 150.9441324 |
| RwbHLH37 | RsbHLH088 | 0.34312002528946994 | 1.5755218450197712 | 0.21778182662085788 | 129.1411348 |
| RwbHLH32 | RsbHLH090 | 0.395327861395007 | 1.3559891810904858 | 0.2915420468746546 | 111.1466542 |
| RwbHLH35 | RsbHLH087 | 0.38156527321057143 | 1.4954474228714871 | 0.25515124595815475 | 122.5776576 |
| RwbHLH42 | RsbHLH115 | 0.17380741236498493 | 0.38545931332667605 | 0.4509098790867286 | 31.59502568 |
| RwbHLH30 | RsbHLH028 | 0.03410953522577688 | 0.04498888922643221 | 0.7581768701623465 | 3.687613871 |
| RwbHLH31 | RsbHLH115 | 0.05944595586403345 | 0.27724356137935 | 0.21441780493756554 | 22.72488208 |
| RwbHLH28 | RsbHLH083 | 0.17066768432444762 | 0.9170202420394793 | 0.18611114182700897 | 75.16559361 |
| RwbHLH28 | RsbHLH097 | 0.005557296188957681 | 0.08769100354417662 | 0.06337361832286534 | 7.187787176 |
| RwbHLH27 | RsbHLH101 | 0.06108393322318338 | 0.17248112151799144 | 0.354148515997513 | 14.13779685 |
| RwbHLH25 | RsbHLH012 | 0.0782805305010294 | 0.12659039051250082 | 0.6183765622659896 | 10.37626152 |
| RwbHLH24 | RsbHLH017 | 0.030038015337023403 | 0.09923335216447005 | 0.30270080252089226 | 8.133881325 |
| RwbHLH23 | RsbHLH010 | 0.04392570127305756 | 0.06623687210720439 | 0.6631608630607406 | 5.429251812 |
| RwbHLH25 | RsbHLH027 | 0.16792917917284192 | 0.6793118904027688 | 0.2472048282170882 | 55.68130249 |
| RwbHLH23 | RsbHLH061 | 0.3931390602749516 | 1.1881619208586067 | 0.330880036948883 | 97.39032138 |
| RwbHLH24 | RsbHLH054 | 0.3664471751050899 | 1.5652898152716994 | 0.23410819614991416 | 128.3024439 |
| RwbHLH21 | RsbHLH077 | 0.023853290720722398 | 0.052256983712327135 | 0.4564613000251589 | 4.283359321 |
| RwbHLH20 | RsbHLH083 | 0.030353012384637147 | 0.10617371963056488 | 0.2858806538025747 | 8.702763904 |
| RwbHLH22 | RsbHLH084 | 0.05457557155884249 | 0.13111078198047182 | 0.4162554042807189 | 10.74678541 |
| RwbHLH19 | RsbHLH085 | 0.006976794496735171 | 0.08284274667323278 | 0.08421732471346745 | 6.790389072 |
| RwbHLH20 | RsbHLH097 | 0.173860713355826 | 1.13741661515465 | 0.15285578831832594 | 93.23087009 |
| RwbHLH21 | RsbHLH100 | 0.2650342077272634 | 0.6262197307073718 | 0.4232287721561939 | 51.32948612 |
| RwbHLH18 | RsbHLH046 | 0.33602294670292115 | 1.4864364212392753 | 0.22605941424845558 | 121.8390509 |
| RwbHLH15 | RsbHLH043 | 0.32713667305516464 | 1.5465915693597418 | 0.21152105024766993 | 126.7698008 |
| RwbHLH17 | RsbHLH050 | 0.3981681838628346 | 1.112220355001513 | 0.3579939731118236 | 91.16560287 |
| RwbHLH16 | RsbHLH073 | 0.2382146778222181 | 1.1704974030718236 | 0.20351576790948325 | 95.94241009 |
| RwbHLH18 | RsbHLH062 | 0.21981295306839527 | 0.9669796623420759 | 0.22731910672867375 | 79.26062806 |
| RwbHLH17 | RsbHLH090 | 0.047988284182761395 | 0.0794614335070355 | 0.6039191852549769 | 6.513232255 |
| RwbHLH16 | RsbHLH086 | 0.04275215019418098 | 0.1463685017544929 | 0.2920857266537448 | 11.99741818 |
| RwbHLH18 | RsbHLH088 | 0.015492417622393048 | 0.08467131343482401 | 0.1829712684723898 | 6.940271593 |
| RwbHLH15 | RsbHLH087 | 0.027403784145838293 | 0.031905564007973786 | 0.8589029844133013 | 2.615210165 |
| RwbHLH14 | RsbHLH026 | 0.2995638069841405 | 1.0959223540452339 | 0.27334400642381285 | 89.82970115 |
| RwbHLH13 | RsbHLH031 | 0.01337175889349377 | 0.08522117969484581 | 0.1569065218455606 | 6.985342598 |
| RwbHLH14 | RsbHLH037 | 0.07497122521445033 | 0.14327750400314215 | 0.5232588726057523 | 11.74405771 |
| RwbHLH08 | RsbHLH032 | 0.007991196577553166 | 0.03887509825984514 | 0.2055608071814813 | 3.186483464 |
| RwbHLH10 | RsbHLH092 | 0.024035987269768824 | 0.16322867153914064 | 0.14725346376420903 | 13.37939931 |
| RwbHLH06 | RsbHLH052 | 0.18465836692684962 | 0.9683575532307755 | 0.190692339116647 | 79.37356994 |
| RwbHLH07 | RsbHLH094 | 0.011744795914925566 | 0.10064336536118419 | 0.11669716998012132 | 8.249456177 |
| RwbHLH06 | RsbHLH093 | 0.050732928937877696 | 0.10185115586929622 | 0.4981085241976278 | 8.348455399 |
| RwbHLH03 | RsbHLH056 | 0.036392214085892244 | 0.09637990916309189 | 0.3775912884946817 | 7.899992554 |
| RwbHLH05 | RsbHLH063 | 0.01570738038393235 | 0.050866947254070795 | 0.30879345492224947 | 4.169421906 |
| RwbHLH04 | RsbHLH089 | 0.01820339641279261 | 0.03162523847669085 | 0.5755971271555561 | 2.592232662 |
