# Supplementary material for: Comprehensive analysis of bHLH transcription factors reveals candidate regulators of flower development and heat stress response in Rhododendron simsii
Source: BMC Plant Biol. 2025 Dec 8;26:69. doi: 10.1186/s12870-025-07868-x (PMC12797679; doi:10.1186/s12870-025-07868-x)
Supplement: Supplementary file 1 — Supplementary Material 1: Supplementary Figure S1. Chromosome localization of RsbHLH gene family. Supplementary Figure S2. Sequence logos of RsbHLH proteins. Supplementary Figure S3. The Spearman’s corrlation coefficient of RNA-seq data and RT-qPCR. Supplementary Figure S4. The expression patterns of species-sepcific RsbHLH genes in different organs and in different stages of flower development. Supplementary Table S1. Protein sequences of bHLH genes from A. thaliana, R.simsii,R.williamsianum, and R.irroratum. Supplementary Table S2. The promoters of the bHLH genes in R. simsii.Supplementary Table S3. The primers of 12 candidate genes used in this study. Supplementary Table S4. Coding sequences of RsbHLH053 and RsbHLH059 cloned for GFP fusion and subcellular localization. Supplementary Table S5. Physicochemical properties and subcellular localization of bHLH protein in R. simsii.Supplementary Table S6. The distribution of RsbHLH genes on each chromosome in R. simsii. Supplementary Table S7. The classification of the bHLH genes inArabidopsis, R. simsii, R. irroratum and R. williamsianum based on the phylogenetic analysis. Supplementary Table S8. Conservative motifs of RsbHLH protein in R. simsii. Supplementary Table S9. The promoter Cis-element functional classification of RsbHLH genes. Supplementary Table S10. Segmentally duplicated RsbHLH gene pairs. Supplementary Table S11. One-to-one orthologous relationships between R. simsii and other plants. Supplementary Table S12. The expression patterns of RsbHLH family genes. Supplementary Table S13. The expression patterns of RsbHLH family genes in different stages of flower development. Supplementary Table S14. The protein interaction network. Supplementary Table S15. The RT-qPCR data of 12 RsbHLH genes in different stages of flower development. Supplementary Table S16. The RT-qPCR data of RsbHLH genes in high temperature-treated R. simsii. [file 12870_2025_7868_MOESM1_ESM.zip › Supplementary Materials/Table S2.docx]

**Table S2 The promoters of the *bHLH* genes in *R. simsii***

>RsbHLH001

ACGTTCTAAAGTGGTACAGTCATCAAACTAATTTTTAATACTTTTTTCATGAATGATCTATTCACATTAGTCTAAGGCTCCATTTTGTTAAAGTTTTTTTTTTATTTTTTAAGTACTTATTTCTTGCTTTACGAGACAGGTCATTCTCTTTCAATACATCATCTTTAATTCGAAATATAAGTACGCACTTATTTTAGTTGGTAGAGCACACTATAAACACAGGTAATGAAAGTCAAAACTTTTTTGTGGTGGTATTGCAAATAGTGATAGGAGGAAATAATTGGGAAGGACGAAGGAGCATAAGAGTGCAATGATGTAAGAATCACACACAATCAGGGATACCCACAAAGCTGGGGAGTCACTCACCAAAGGAGAAAGACCCACACAATCCCCCTTTGTAAGTGTGTTAATTTGTATGTAGTTTAAGGATTTTCCATTATGATAAAAAAGAGAGTATATTTTCCATGAATTATGGTACCTTGACGATTTGATCAGTTTATTTTGTAAATCAAGATGTGTTTATTGTCATGCAAAATAAAAAAATAAAATAGACTTGATATTGATAAGAGCTTGATCCAAACGTATTGGTCATATAAGAAATAAGGTGCGAGACAGTGACCATGAATCTCCCAGGAAAATTATGCTTCGGTTGGGTGGATTTTGACGTCCTATAAAATTGATTTTTTGGATGGATAATAGATCAAATCGAAACCTACAATACGAACAAATCGAATCGTCAATGGGACCACAATTAATTTCACTTTGTGTCACAGAAAGCAGAGGCGGTCCTAGGGGTGCAAGAGGGGGGCCTGTCCTGTGCACACCTTTCATAGTTTTGTAATAAGTATTGCCATACTTTATGGTAAATGTGACCATAAAACATACTGTATAGTCTTTTTTCATAATAAGTAAAACTGTAATTTTGTGAAGGGAAAATTGCAAAACCACCTCGTTTTTGGCAATTTTAGCTCACTTCTGGTTTTGAAATCAAATAGCTTATATTCACCAATTTTTGATCTAAATGACTGAGATTTATGGAGGAATTCATGTCCTAATACCACATTGAACCATGATAGGAATTGGAGCATATAGGAAAATTCGCTCAAAACTGTCATTACAAATCATGCTGCTGGTCTGTTTTCTGAGATTGTGAGTGGGCCCAATTCCTTGGAATCTTCTAACAAACTTCACAGCACTGGCAAAACGGAATGGAGGGCTCTCTCTCTCTCTCTCTCTCTCTCTCTCTCTCTCTCTCTCTCTCTCTCTCTCTAAACATGTGTATAAATACATAGCTTCCATATATATTGGACTACTCTTTCAATTGAGACGAGTAGCACAAATTATGTTCATCATTTGAATTGAGGGGCTTCCATTTTATGATGTGTACGTAGTTTTAAGTACTCACTGGCCTTTGCCATTTTTTCTGTTCCTTTTCTGGAGACAAAGACCAAGAAGAGTATAAAGGACAGAAACAAACACCAAGTTTTTGTCGATCCAAAA

>RsbHLH002

TATTTCATTTGAACCTTCCACCACCACAGCCTCCCCTGTTTAGGGGATTAATGTTTGAGTCTCCAATACCCAATGGGGGCTACAATATGAATACTGGTTCTTTGTTTGGTGGAGTGGTGGATGAGAGAGAAAGAAGTGGTGGTGGGTAAGGATATGATAATGGAGTTTTTGAGTTCAATGGTGGGGATTGGAGTTGCAGGGGTAGGAAGAGTAATGGGAAGGGCAGCAATCGACTGTTGAATACTGAGAAGCAAAGGAGAGGGCAGTTATCTGATAAGTACGAAGTTTTGAGAAATTTGGTTCCCAACCCCACCAAGGTATTCCCATCTCATTACAAGTTAAAAGAGCAATTTTGTTCACCCTCACATTAGGTAAGGATAAGTTTTTCCACATTCGATGAGGTGTGTTGCGGTTTCGATTCGATAAACAAGACCGTTTATTTTATAGACCTCTCTAAAATTCAATTTGGATGAAAATAACCAAAATTTTGCTCTATCTATTTCTAAACTCAACAAAAAGATTTTTTAATCAAATAATATTTGATTGAGCTTAATTCTTGCATATCTCCTAATGAATCAATTCATATAAAATAAGCGATTCTGATTACCAAATTGAAACCTACATGCGTCACCGAATGTGGGTAAAGTTACCCTCACCCAAGCGAATGTGAACAAAATTACTCTCTTACAACTCTCTCTCTCTCTCTCTCTCTCTCTCTCTCTCTCTCTCTCACACACACACTTTCTTCGATCGGGAAAGAAAGCAATGAAAAAAATTGCAATAGATATGAAAAAGAAAAAGCAATAGGTGGTCTGAGAATCAAATTAGTGTTGTGAGTGTCATCTCCAGAATGAATATGAGTACATAATGATTTTCTATACAAGGTTCTTTGTATTCGTTTTTTAGGGAACGTAGCATAGCTCATATTTAATATTAACTGCTAATAATGCAGATGCTTTTGAGTATCTTACAAACTGTGAGCCAAAACAAGTGTAAAGTTAAAATTTGGATTTTGGCAAGAACTTCTCAGAATAGAACAAAAAACAAACTCAAATTCTATACTGAAGTTCAATGTCGCCAAATCTTGGCCTTGCAAACGTTCAGGACAAATTTGGCACCAAACGTGGTTACTAGCTTTTGGCATAACTCATCCCCTTGAATCTTTGATCACTCTCTCATAATGGGATCTAAAAGCCTATCTAAACAGTATTGCCTTCATTGTAGAAAATAAAAAAAATTGACATTTGCTCCATTGGAGCTGGATTGTCACATTCTTGATTATGCTAGTACTTTGGCAATGGAAGAAAGGCCAAATTTTGGCTTTGTTGTCGCCATTGTAATACCAACCAGCGATGTTCTTAGTTCCTTTAATTTTCTCCCTCTCCATGTTTACATGTCACTACCCATTTAAGGAATTCAAGAACTTGGTGATGCACGTATACATGCTTAAATGTAAAGTTGGCTTTCTTTTGTTTGACATTTTCAACTATTTCTGTTTGG

>RsbHLH003

TTTAAAAAAAATTCAAAACACAAACTAAACAAAGCAAATTAGAGATCGATCGTTAGGCTAATGAGTTACACAATTACCGTGGTAACCTAGTGGTAAACAACTCACCTTTACGGAGTTCAGGTTCAGGAGGTTACAAGTTGAAGGCATGCCCGTCATGTCTTACAATCTTTTTAGTCTGTGGTCCGGCTGAGATCGATGAGGTGGTGGGAAAAACGTATGAGTAATTGATCACTTGCGAAACTCACCGCGGGTCAAGAATCTCTATTATCAAAAATTGTTATAGCTGCTGATTCCATTTCCGGCTTTTTGCTTAGCAAAAGAGTGAAAACAATCCCTGGGCGGACTTTCCAAAGCCTTTCCTTTGGGGCGCAGTACGTTTGGAGAGTCCCCACAAATGTCTGTCCCCGCTGATGGACAAATCGTACCACCCCAATAATGCTTTGGGGCCTTGTTAATCAGGCAGACAAACACACTCTCTCTCTCCTACGGTTAAGGTATTCGGGTCAACTTACATGAAACTCAGCTAATTTCCTCTCCAATCCAGTCAAAAATATATTATTCATGCCGAAAATAGAGCAATCACAGAAAATTACTTTTTTGTGGCTAGACTCTGAAAACCAAACGCTAATCGATTATGTAGAAAACAAATCCACCAACGGAACCCTCGGATGGCCTCCTGTAGCTCTGTTACAGTCTCACCAACTCTCTCAGGCCCCCCAAAATATCTCTCTCGCACCGTAGGAATTTTTCAGCCGGGTGAGTATATTTCACGTGGTACTCGTTTGGCACATTCGGGTCGTCTAATACATTTTTTGATAGCTCGGATTAAAACTCTCTTTCTCCTCTTACCCCAATACTTTCTCTCTCATTTTTTTCTCTCCAAATCTGAAACGTCCAAAAACACAATGAAGGGCACCACGCGGTACTCATCCGACACTGAAAATTTTTCCCGTTCGCACATATTCTTTTACTCTCATCAGATACCTTGTTTCTTTCCCCACCCTTCGTTTCTTACCACATCTGTGACTGTCACACACTCTCCTCTTCTCTGTTTAATCCCCTCCCCAGCCCCAAATTCTACTCGCACAACTGTTTGATTCGTCTTGGACACTCTTCTCTTCCTCGCTCTAATTTATTTTTGTTTCTGGATCTCTGAGTTTTATACTACTAGTACTTCTTGAGCATTTAACGTTGTTTTCCTTGTTTTTTTTCATCGCATCGGGGCTCGTTCTCCTTCCTTGGGCTCACACATTTACTATTTTCTGGGCTCACACATTTATTATACTATTATATATCTCAAGTTCCCTCAAGTTTTGTTAGTATCACTGTGGAATGTTTTTGACTGAGGCATAGAAAAAAAGAATTTATTGAAAGGCATTTGTGGGTGAGAGCCAGGCCCCTCTTTCTCTCTCTCTCTCTCTCTCTCTCTCTCTCTCTCTCTCTCAATCAATATACTGTATAGTACTCTGCAGCAACACCTGTTCACCCTTTTTAGCAGTA

>RsbHLH004

GTGGTATATTAAGTATTTTCTCTTGAGACCGACAAAATGAAGTTTCCTCCTTTACCACCAAATTACGCAGACTACGTGTTTGATCCCTATGATTGAAGAGCCGATGAACTTCTGATTTTGTGGATCTATAAGAGAAAAGTGAAATATGATTGAAAAATCTTAATTTTGACGATTACTGAGATTTGATTATTTTTTCATCGAAACGTCGATGATCGATACTTTTAATACCAATGGAAATGTTATCAACGTTAATGTGCACATATATACTTAAATATAAATTGTTCAAACCGTATCGTGTGGGTTTTATGTGTTGTTGGATGTAATACTTTGCCACACGATGTCCATCTTAAATGTGATTACTTCATAAGGAGGGTTCAATTTGTAGACAATATTTTTTTAAAAATGAAATAGTACGGCCCATGAGTTTCTTGGTCAAATTACTCGCTCTCAATCTCATTTGACCATCTTGCTCACTTTCGCACATGTTCACGCATTACGTGACTTAAGACATGACAATTGCGTTTACTGTTAGAGGCATGCAATTCACTCGGAAGCATGTGTACAAATCCGATATATTTAAAACCGTCTGATGCGCATAAGCCCCACACGACAAACGTGAAACCTACACATATCAAACAATATAGTGTGCACATGTCCGTTCCACTTCTGTATGATTTCTGTTGGAATTGAAATTGGGCATTGCTCCTTCCTGAACGGTGCTACTGGTACAGCAGCTGCGTACAGATTCCTTTTGCGGCCGTCTCGGATTCCGCAAAGATGATCGGAGCCGCTCATTTTGTTCAAAATATTTCGTTTAAAGTCCTTGTAAAAAATCACCTCAATCCGATATCGGGAAGGGTGTTTACGAATGCTTCAAAATTTAAGCGGCTTTAATCATCTTTGAGGAACCCAAGACAGGCGCAAAAGGGATCTGTACGCAGTTGCTGTACCAGTAGCATTTTTGTTCCATATATCACAGCATCTCTGCTGATAATTTACTTTGGTCAACACCCATAGGAAAGAGTCGAAAGACCCCAGGTGCACTGTATCTACAGTGTATAAAAAGAACTCTCAGATCTACACGAAAACAGACAACCAACCACCCAAGTCTTTTTCTGATCCAAGGAAAAAAACAAAAACCACCCAAGTCTTTATTGAGATCACATGAAACACGAGTTTACCTTATCCTAAATTCCACGATCCATGACAAAACCCAGGTAACTGTTCTTTCTTGCTTCGATTGCAGTTCTTCGTGCATCTTTTGTGATTCATACGCATACACATATGCACACAGACGTTTACAAGCACATGGGATTGCCTTTGTGTCAATCTCTCTAGAGAAAGAGGTTCCGACTCTTTTTCGTCAGAAAAAGAAAACACCCAAGTCCCTAAAATTGGACTTATATAGGTAGCTGTGTGTTGTATGGTCTGTGAGAATCTTGGACCCTAAAGAAATTGTTGTTGTTGTAGATTTAGCGTTTGTTGAATTGCTTGAGAGAG

>RsbHLH005

TCCGACTTGCACCTTAACAATCGCTGGTCGTTTCTTTATGACAAAAAATCATTCTAAGACATACGAATAACTTGCACCCTTTTAGTCGTGCTTGGAAAAATGGCTAGAATCGTAAAAGTAGCCTTGCTCCCGAGTTCGTTGGAGTCCGCAGTATTGGGAGATGGTGGTGCAATTGGCCAACAAACATCCAATCATTTGTTCCACGTCTTTGTAATAGCTTCAGTTCTTACCACTTATCAGTTAGGGCGTTTCCAATCAGGAAAAATTTATAGGAAATTTTTTGTTGTTGAAAAGAAGTTAGTTATTTTCGGTAGTAAATAAGTTTATAAAAAATGTTAAATTGTTTCCACTAGTGAATCAATTGTTGATTGGTACGTGAGTGAAAAAGTTGTTGAGAAAGAAATTATGGCTAACAACTTTTTTATTAGTGTAAATGAGATGATGAAATGCTGAAACTTGTCGATTAATGTGAATAAAATGTAAAAAATGCCCAAGTTCTTTCTGATTTTTGCTAGTGGAAACACGACCACCTGGACAGCAGAGACTCCCAACCATCACGCCATAATTGTAAGTACTATGGAGTGGTAATTAATTACCCTCCTGTGTGTCCAAAGGCACTACACGTTCAAATTATTTTATATTATGGAGTATTTTTAATTTGTGAAAAAAGTTTTAATTTTTTTTTGAAAAAATTGCATTATTTTTAACATTTCGTTTTGCGGACCGGACAATCAATTTGAAACGGAAGGAGTATGTGAAATTATAATTCGATATCTAGATGGTTAAAAATGCACCGGTTCGACAATTAATGATTCAAATTATTTTATATGTAAGATAATATTTGATGGAACAGGAAGGCCCACGGCACAATTTTTTAGTGCCACAACACAATATTGCAATTTGCCATTTTCATTCTCAAACCAGAAATTTCCCCTCACGAGTAAATTAAAACCTTGCTCCTCTCTCTTCATTAATTTTCTCAGCCACGGCCTGGGCTCGACCCCGAAGCTCCGGTGCCGAACCGGTATATTCCGGTGGCCACTTGCCACTCTCGAGCCGTTTGATGTAATTTTAGACAACTCAGATCAAAATCTTCTCTCTCCTCTCACTCCTCTTCTTTCTTACTCCTTTTTCTTTCTCCAAATGAGTGAGAGAGAGAGAGAGAGAGAGAGAGAGAGAGAGAGAGAGAGAGAGAGAGATTCATTCCAATTCGCACTCTAAAACTGTAAGCTGAGAGGTACCTCAGTTGACCAAACCCGAGTCTCTGAGCTATATTATTCTTCATTTGTGCTCGAAATTTCTGATATTCCCTATCTAATTTAAGCTGGATTTCAATCCTGATTTGATGTTCAACTCAGTGGTAAACTCGTCACTCTCTGTTGTTGTCACACCACGAATTAGTTTGAGTTGAATTGTTTCCGAAACCTGAAGGCCAAGTGCAAGCAAGCAAAGCAATTTCGTGAAGAAATTTGAGCGGTGGACGGTTTTCAAAGCTCACAA

>RsbHLH006

AGCTCTCTTCTCTCATTTCAGACAGTTAAAAGGGCAAGTTCAAGAAACTCTCTCCGCCAGAACCTCCTCGACGACGCCGGCGTTGCGCTCATTTCTCAGTGGGATGCAATCTCTGGCCTTGAGGTATTCAATCTCAATACGCTAAGTTTTGGTTTCAGATTGTTGGTGTTCTGGGTTTCATGTTTGGTTGGCGATATAATACGGTTTATTTCTGTTAATTTAGCACAGTAGTTAGGGTTTACTCTTCTCAATGAAACCCCCATCAAAGCCAGTTAGGGTTCATTTATTCTAGTGTTTGCGCTTTTTCCCAAACTAGCAATATTCTGTTATTATTTGATTTTGGTTTTCATTTGTTTTAGATGGATGATATTGATCATGATGACATTATGGATGAAGCTGTTTGAGTTTGATTTTCCTGAACATTCTGAAAATGCAAATAACGATCAAGTGACTGAAAATCAAAGGAGTGAGTCTGAAAAAGTGGTGATGGTCTATTAAGCAATCAGAGGTCTCCTTGCTGGAAGCATTTTAAAATTCTTACACCAAAAGGTCATTCGAAACCTAGGGTTGCATACAATTGTTGTGGGAAATCTTATGCATGTCATTCGGGGAACAATGGTACAAAATCCATGACTCATCATCCAGCGTATTAGTGTACCAAATATCCCACGAGCAAGAAATGTACGTGTTCTATGCAAAAAAATCCTTGGTTTTCAAAAAGTAAAGGAACACAATGGTGAAACAAGTGCAAACCTTGTGCTTGTGACCTTTAGTGTGTTAACGCATGTAAACGGGCACTAGAGGAGACGTTAATAATTGATGTGTTGCCATTTTCATTTGTTGAGGGTGAAGGATTCCAACACTTTGTATGCGTCTTTCAACCAAAGCGGGTAAAGCCTTAAAAAAAGCCAAAAGTACTCAAAAAGCCAAAGTATGGTTTATGATTTTGGTAGCATTGAGCTCGATTTGGCTATTGACTATTTCTACAAAAAGCTACTGCAACCCTAATTTGTTTCAATTCTGAAATAAATTAGAATATTGAGGAGCATACACTTTGTTTTCTTTTCTATTTTCACCAGGAAGAAGTTGTTAGCTGATCTTTTGTTAGATTAGAACTCTCCATGTTATAGTATAGACCTATAACATGGTAAAACTTGCATTTGAAGTTCGATGCTTCTCATTTTTTAACCCCCCCCCCCCCCCAAATGGGTTGAGATATTGCCAGTTATCACTTGTCAGGCTCAAATGGTTAAGTTTCATTGGAGAAATCAATGACTTAATTCATCTGCAAGGTTCTAATTATTTAATCATCTTGCTTGTATCAATACTCATATATTCTGTACCTGTAATTAGCTTTTATCAGCCCCAATTAAAATTCATCTGAATACGTCCTTCTCCAGCTATTGGGTACCTATAACTTTTGAAAATTTAGTTCCTTTTGTGCAGACTGAGTCGCTGACTGTTCTGCCACCATATTCAAGCAGTTTTGCTTAGGATAAT

>RsbHLH007

TTTATCCAAAATGCCGTGCGGTCGACCTCATGACCTCGTGCGGCGTAATGAAATCTATTATGCCGTGCGGTGTAGAGGTGTGCCGTGCGATACTAAGGGTCCAGTTCTGGGCTCTTGGGTTTGCTCTTGGCTCTTGGTTTTGCTCTGGGCTTTCGGGTTTTTGGGAAAGTCCCATTTCCAAGCTCTCGAAGATATTTGTTTCATTTATTTCATCATCGGGATGTTTAAAGCACTTTCGAGGCTCAGATTGGAGTGTCTACACACATCTGTTGTATCGATACTGCCTTCAATATCTCTGGTTCTTCACCTTTTGGCCTCCGTCTTGGCATCTATTGGCCAGCCACCCGACAGCTCTTTGAATCTTCGTTTTTGGGCTTCAGGGGCTTCATTCTTGAACACTTGCGCTCTTGGAATCTTCCACGATTGCTCCAAGCTTCCAAACCATACTTTATTAAACGAATTACCTACAAAACGAAAGAAACTACCTTACGTGCAATATCGCCTAAAACGACTTTTAATCTAGATAAAAGCGGTAAACTAATGGACTTAAAGCCCCAAGAACTACGTTATTGGGTGCTTATCATCAATCATTACCCTGATATCTCTCTCCACTCATTATCCCAAAAATCAAACTAAGAAAATTTCTCAAAAATCCATCCAAACACGACATAAGTGGCCAAAATTGAAAATGACCGAAGTGACATAATAGGAAGCACGTTTGTCTCGAGAGAATTGAATGACTAGTCCCGTTCCAGAACGATAAATAAATACTTATTTTTAAAAAAAGCAATTTTAAGCTAAAAAATAATGGGTTTACTAAAATCTACAAATATGTAATATGGGTCTTATTTGAAAAATCTCGATGAGATCTTTTATATGGTGCAAAAAAAAAATTAAAAAAACATTTTTTATTTGCATTATTTTTAGTTTGAAAATATGAAATAAGTACTTATTTTTTAAAAAGGTGTTATGAAACGGAGAATATGAGTCTGAAACAAACCTTCAATATCATTAGTTACGGATTTATGGTCAGTGTGTTTTTCTATAAGCTCGTCAAACTTAGACGAACCAAATAAAATAAAAACAGTTTGAAAATGATTTTTAGACCTGGAATAAACCACAAATTAAGGACACTGTAAAACCTGCTGTGATGTGATCCTAATCCTAATAACAACAAAATCCCTTGGTACGAATCCTATTCATGAAAAATTATTGCATGCTTGTGGAGTATATTCATGTAGTTCTCCAAATGTATTTTATGTTAAAATAATTTAAGAGAAAAAATGACAGACGGGACCATCCAAACAACAAATATACTCATCCTATAAGTATTGAACAAATCTGCTCCCCTTCTCTTGGGGAAAAGAAGGCAAAGCAAAAGAAAGCAATAAAGGACCGACATCACTGCATCACCCTACTTCACACTTTCTATCGTTCTCCTCCTATACTCTCTCTCTCTCTCTCTCTCTCTCTCTCTCTCTCATCACAAATTAACCATCC

>RsbHLH008

CACATGCGGGTACTATATGAAAGTGTCTAAACCCTAGGGTACCCTATGAAAGTGTCTAAAATGATAAACCTTATAAAAATGCTTAAATTACTAAAATCGTATAATAGAAAAAGTGCATTCTAAAATTCTATGAAAATGCTTAAATCCTATAGTATCCTACTATGGACTTTTTAAATTGGACTTGACACCTTACAAAACTAATTGGTAGATTTGAAGACTTATGAAATTTACATAATGAACCTAAGATGAATTTTTTATTTAGTTAACCATTGATAGTTTAAAACAAAAGTAAAAGAAGACTCGGGCTCTATAGCACGAGATCCGAATTGTCCATCTTTGCTAATCCTAAAATCTAAAAATTGTGCAAGATGAACGGCTCGAATCGCACACTATAACCCAAAATTTCCGAGTTCACTAATACAATGATAGAGATGATTTTGTAAAGAAAGTGGATCCTCTCCGGTCTAACTTTTGGACTAGAACAGACAATCAAAATCTTGATCGTTCAATCAATAGTTTATTTTTGTCCAATAACTCTACGCGTGAATAATTGAATTCTTCTTGCAAATAATTTATAAGCATATCTTTCTTTTTTTTCCCGGGTTTATAAGCAAATCTAAACCATCGAGTTTCTTAATGTGCTGCCCAGGTTAGGACTGTTTCCTTATGTCCAATATTTGGATCGGAAAGGATCTATAGGTAAAATACTAGGGACACATACGTACATATGTACACGAATCTCGTACAATCTGCAAGTGGTTGTATTTGTACGTGCGTCAACTGAGTAGATTTTTTCCCCCCACGATAAATATCGTCAAAAAACGATTTCGTCTCTTTGCTATATTATTCGTGGAGAGAGGAGAGAGAAAATTGAGAAATGGACCCTTATCTTCTTCGGCTGCAAAAAGCCACTAACCGGAGGGAAATAATTTCAGTGCCGGATGGATATTATGTGGCGCCCGTCAGCTCATTCAAACTGTTCATTTCATTTTTTAACGGTTTAGATTTGGAGAGAGAAAATGAAAGAGAAAGTATTGGAGTGAGAAAAGAGAGAGAGTTTTTATCTAAACCGTCCAAAAGTGTATCGGACGACTCGAATGCAATGAACGGATACCGCGTGAGATATACCCACTCGGCACGGAAAAATCTCTCGCATAAGGCAAAAACAATTAGTACTACTAGCTCAACTTCAAATCTTCTGAAGCATTTCTCCATTTCCCCATCATCTCCTTCCAAAAATAACACAGAGATAGAGAGAGAGCTCAGAGAAGCCCCCAACGAGGAGAGAGAGAGAGTGAGAGAGTGAGAGAGAGGCGGTTACAGAGGAGATGCAGTTTTATCCATTTCCCCATCATCTCCTTATAAAAACTGAAATTTATGCCGAGAGTTTGATTGATCCCTTGAAACATCTCTCTTCAGTGTCCGCTTCCTTTCGAATCGTGCTTGATTAAGATTTGATTGCTTGCGCAGCTAAATTGATCATTAATCCTATCGCACAAG

>RsbHLH009

CGACTATTTGCAACCACAAATTTCTTAAGTTTAGTCCCTTCTAAAATTATTGATTTTCGGACATAAATGCCCTTTTAAAAATGGACAAAAATACCCTCAATGAAACGACGTCGTTTTAGGTCATCTTCCTCTCTACTCTTCTTCCCCAACCCACCTCCCACCTACCAGTCTTTTTTTTTATCTGCACCTCCCACCTAATAGTACCACCACCTCATCCGTCCCCTCCTTCTACTCCCCACCACCGCCGACGCCGCCGTTACATCCGCCACTACAGCAACTGCCACCGACTCCGTTGTCAGCCACCGATTTTGGTGTGGAACTCGCATTTCTTCACTGAAGAACCCACTCCCATCGCGCATTAGCAAAATGTGCGATGCTAATCGTGCATTTTGTGAATGCGTTCTACGGAGCATGACACATTCAGAATTTGCGTTTTTTGCATTTTTAATAGTCAGAATCTGCGTTTTTAACACCAAAAAAAAAAAACATAACGCATCTTGAAGATGCGTTATAAAGTGTTCAGATTTTAAAATCTGAAATTTGTAAACATTTTCAAGTTTAAAAATCTGAAATTGTCAAAAAATTTTAAAAATGTGTATCCAAATATGTAGGGGATTTTTTTTGAAATTTCGGATGTGGTGGTGGTGTGTACAGCAAAAGAAAGAGAAGAAATAATTGAGAATGTGTTGTTGGTGGTGGTTGTGGCTGACGAATGTGGTGGAGGAAGCGTTTGCTGCTGCTAGTGTTGGTGGTGTCGACTAGTGTGGTGGTAGTGAACGGACAGAGAAGAGAGAAACGGGAAGAGAGGGAAAAGGCCAAAACGGTGGCGGCAGTGGTGTTGGTGATGTTGATGGTGCGTTGTTGATGAACTCCCACGGTGGTTATAGAGTTTGGTGGTGGTGCAGGGGAGAGCAGAAGAGAGAAACGGGAGAGATAGAAACGGGGAAGAAGAGGGTAGTTTTGGAAGTTTAGGGATATTTGTTAGGTTGCATTTAGAAAATTGTTATAAGATTTGTGGGTTAGGTTCAGAACATATTAGGTTTAGGAAATTTGTGGTCGCAAATAGTAAGCTCCTCTCCAAATTATTAACCGATAATTTGGAAATTATTAACCGAAAAATCAGTATTTAGATACTGATTTTCGATTATTAATTTGAGTGTGCGAAAAACAAGCTCTGACAAGGAGATGGCTCAGAACGTAAATTTCAGTAAACGGAGATCGTATTGGGCTGAGCGACACCAATCAATCTCTCTCTCTCTCTCTCTCTCTCTCTCTCTCTCTCTCTCTCTCTATATATATATATATATATCTATATATATAGAGCTCTCTCTATATATATATATATAGAGAGACTCACAGTCACGCGCACTACGCAAAGCCAATTCTCCCATCCCTTTCCTCTTTGACCTTTTTGTAGCTCACTGTCGATGCTCTTTGAAACTCCCAATTCCCCTCGCTCACCGATCCACAACACCTCGACTCAGCCGAGTCACCGCCACC

>RsbHLH010

TTTTGACCCCAAGTTACAAAAGCAGATAAATAAAGGATATTGGGGTGCTGAAAGCACTCATTTTGTTTACAAGTTTCGAGTTAATCCAGTAAAAACACCCAAGAAACAGTCAAGCTGTGCGGCTGTTGAACACAAATTTCAAGTCTATGAAAAAGACAGGAAAATTGTTGAACGAGAGAAATTTTACATGCCTACAGCGGCAGTGTTGGTCCTGACTTTTTCAGGGGGTTAAAGAGAAAATAAAAAATGAGTCCTTTTTGTTAGACTATTAGAGTATCCACAATACTATAATCAAAACTAAAAGGTTTTTAAAGTTAATAATGTTAATACAAAATATGGCTCATAATACTATAATTAAACTTAATAATCTCCTTAGAAATAATCAAATTTTAAACTTTAAATAACCAAAATTAGCAACTTTTAATTTTGACAATAACCAAATTTATTAGAATACCCTACATATTCTCAATCAAATACACCAATCATTATATAAAAATGTTCTATCTCTCTTCCCCTTTACTCTCTCTCTTTCTTTTCTAAAAATATTTTCAAAAAATTATTTTTTTTAAAAAAATAGTTTTTTTCCAAAAAATAGTTTCAAAAACTATTTTCTATTTCTAGTAAATAGTTTCTATTAGTTTCAAAACTATTTTTAGAAAACCTATTTTCTATTGTTCACAATTTTTAGTTTTTTATAGTTTGAAAAGATAATGTGGCAAATTTTGGTTATCCAATTTTGATTATGTCATTGTAGGCATCTACATTGTTAACCGTAACAATCCCTTAAATATATAATCAAAAGCTGATGTGACAACTTTTAGTTATCAATTTTTGATTATAACATTGTAGATATCCTTATAAGGGGTAAAAAAATTTGAGGGGCTTTAAATTCAAAACTTTTTGAAGACCTAAAACAACCGCAACACGCGCTTTAGCTCAAGCCGGCACGTATGCTTAAGTCAGCCCACACATATTATTAGCAAAACAAAAACGTGAAAAGACAAGCTTTCAACCTAAATAATTACAGATGGAAGTCAAAACCAAATTCTTTCGATTAGCAATTCAACTTTCAATAAACAACCTGGATGTGCCATTGTTCACCCTTCACGCCATCGTTTGAGTAATTTGGACGCATATATTATCATTTATTACTGTACCTCTAGAATACATTCATGCACGCCATCCTCGTACGTACAATGTGAAAAGTGTGAGAAAACTGTTGTTCTTGTACGATCAAGAAGGACTTTAAGGAAGGTGGCGAGACTTAAGAGGTTCCGCATGATCGAATTGGTTTCCAAAATTCCATTGATTGCTGTCCCATGAAGCCGCCGCCCACATGATTCATGCACATTCTTCGTTTTTGTTTCAACGAAGACTGATTGAAAAATGGATTTAAAAGGCCAGTTATCGTAGGATTAATTAATTCGGCTTCTACTGATCGACAAATTATTCTCAAACTCTAAAATGAACATTTGTTCCGGAGAGGAAGGAAAGAAGCAA

>RsbHLH011

CGTTCCTTATAATCCTTTTTTTAAACAAGGTTGAGCTATTTGTGACTATCTTCAATACTTTTAGTACATATGTTGGATTAAATTACTTGCATGATCTATGAAATTACAATAGCCTTAATTAATTATTTGAGTCTACAAAATTAAAAATATTTTGATACTCCAAAATATTGTTCTGTTGCATTACTTACAAAATATTATCTCTTTTTTTTTTTGTTGTAAAAATGACCTTTTATTCAATTACTTACTCTTACGATTTCTTTGTTTCCATAACTAAATCTTAATTTGAATTATCTTACATACATAAACAATTACACCAATGAGTACACAATACCGTGCAAAGCACGCGGAATTACACTATTCTATCTATGAAATTCTACTTTTCGCAAAAAACAAAAAAACCCCGGTAGTGTTTGAGTGGGAATTTAGTTTAGTTTAGTTTTGGTAATGGGTGGAGAGAGAAATAGGGTAATGATTGAAGATAGGGGTAATGATTGGAGAGAGATAGAGAGAGATAAGAAAGTAATAATTGAAAAAATAGGGTAATGATTGAAAAAAGATGTAGAGTAATAATTAGAAACAAAATGAAAACTAAAACTAAAATGGCTTCCGAACAAAGCCCCCCGCAACTGTATTTTTTTTTTTTTGAACAGCCCCGCAACTGTATTTGACATGGCTTTTCTCATGTTCTAAAATGTGACTCGACCGCCTGATTGGCTTCTTGTGGTCCCATATCAAGTCCACGTCAATGATCTACAACGTTCAGCATGTAGGCCATGTCAAAAAGTTTACCTACGCGGAAAGTCAAAATAATCAGACATTGATAAAGCATCTATACGATCACATCTGTCCCAACAAAATCAGATGGTCTATTCTTGAAGTTGCATTTGCTGTCCATTTTGGATTGGGAAATCCTGACGGTCACACAGGACACCCTGTACGGCGAACATAGATAATTGATCTTGATAATGAATGGTTAAGATATGATTTTTAATTATGACTGATTTATCGGTCATAATTGATTTTTGATCTGAACTGTTGATTGAAGAGATCGATAGCTGTGTGCAGTGTGCCGCCCTACACATGCACTACAGCACCTCGTATTTGTCCATTTTATAGTCCAATATAGTGCGATCTAACTATTTAATACTTGTACTAAGTTAAAATCTTTTATTACACTGAGACAGTCTGATCCACAAACCTTTTTTTTTTTAGTAATGTGAGATTAACAAACCTTAGAGCGTCTCGTATCATAGTATCAATATGGATTCCATCTATGACTACAATAGAAGTTCTAACAAACTCCCAACGGCAAAGATGCCCTGCCCCTCTCTCACTCTAAATAAATGTTACTCTTGTGAGAAACAGATATATAAACCAACCCACCAACCAAAAACTCACACTAAAGAAGAGACCCTCTCTCTCAAACTGCCATTGAAGACCGCCAGAGGCTCAGGAAAACTTGAATTCGCAAACTCTGATCAATTTGTTCCAATTTTATCA

>RsbHLH012

GTTAATATTAAAATCTAGGGCTGCAAAAGTGCTCATTTATGTCGAGTTTTACAGTGTTCAAGCTTGGCTTGTTCAAATTAATCCAGCTTAGCCACAAAAATCAAGCTTGGGCTTAGATTGTTTATGAAATAAGTCTTTAAACGAGCCAAACATCTTCAAACGAGATGTGTATTTTCAAACAAGTTGAGTTTTATAAATGAATTGAGCAACAACTAACTCAAGCTCGGCTCCTTGCCATGTTTTAAAGTATTTGAGCTTGGCTAGTTCAAAGTAGTCTAGTTTGAGCCACAAAAATCAAGCTTGAGCTTGAATTGCTTATTAAACAAGTCTTTATAAATGAACCAAACATCTTCAAACGAGATGAGCATCTTCAAACAAGTTGAGTTATTATAAGTGAATTGAGCGACAACTAACTCGAGCTCGGCTCCTTTTCCAAAGGAGTTGACAATTTGACTCTGAACTCAGCTCATTTTACGATTACATAACGAGCCGAGCCTCTGATTGATCGCGAACTGTTCGATTTGTGTTGTTCCCACTTTCTCCCACTGACTTGCGTTTTTTTCGCAAAAGCATCCACTAAAGTGAGAAACAAACAAACTGAGAATGAATTCTATCTACCGCCGGTATAAAATCTCATTTTTCCACCACTGTCCATTTTCGGCCCCACCGAAGGTCTATTATTGTAATTTGAACCGTTTATGTTTTAAGATATGTAGTCTAGTATTGTCATGCAAAAAATCAGCTTTATTGGAAGTCGATAGATACATCAAAATTTGAATTTTGGTTTATAAAATGGGCGATTTGATTCTAATTAATGGAGAACGATTTTCGCGTTCCAAAAAAAGCCTAAGGCGCTCCACCTTTTGTCTTCATTAGACAAACTTCATGAATTACTCTCTCAATCTAAAACTTTGTGTAAAAAGGTAGAGCGTCTGAGGTGTTTTATGGAGCGCGAAAACCTATAAAGAACTTTTTCAAAGGAGGGGAAAAGAAAAAACTTTTCCACGAAAAAGTTTTAGATTTTTTGAAAAGTTTTAGATTGCGAGCGTAATTCAGGAAGTTTGCGTAATGAAACAAAAGGTGGACTGCGTAATGAAACAAAAGGTGGACTGGTGGAGTGCCTGAGGCTTTTTTGGGAGCTCGAAAATTAGTCTGATAAATTGCCCATTTTGCAAAGCAAAATTTAATTGTTGATACTCCTATTGATATCTGATTAAGTTCATTTTTAGCGTTGATACATTATTATATGAAATCTATAAAATAAACGATTCAGATTAACAATTAATCCACAATAAAATCAGAAAAGGCAGAGATGAAAAACCCACTTTTCCACGGCCAGTGGAAAGAATTTATTCTCAAACAATAAACACCATCTCAGACCAAAGCAAAGCAAAGCAGAGCACAAACAGAGTTTCCCACTCTAATAAAAAAGCGCCAATAACCTGTCGCCTCCGTCTCCGACACCTCTCTCTCTCTCTCTCTCTCTCTCTCTCTCTCATC

>RsbHLH013

ATTAATAATTAGAACCATCTATTTCTAGTGTTTGCAAAGCACGACTCTTCTTTTTTCTTCTCAAAATTGATAATTCATTCACAAACTGAACTCGAAAATTCTTTGTCGGTAAAGCTCATATTTTCCCAAATTTGTAGACAAGTCATCTTTCTCATATATTACTATTTAGCGAAAACAACTTAATTTTTGTCATTAGCTTGAAGCAAAAAAAAAATTATTGAAAAAGGATTTGACTCCTCCCCAGTCCAAAATGGTTGGGACTAGATGAGACGGTCCGGGACCTTTCATGGGTAAATTTAATGGTTCGGATTTTCTCATATACTTTTACCGGCAAGGATACACTCTTGAGAGTAAAATTGTAAAAATACATCTAAACCGTTGATTGTTGCAAATGAACAATCTCAATCACGACTGTCCCCTCCGATCTAAAACATCAACGGAAGAGGATCAATTTCCATTGGAAAAATATAGGGGAAACGCACCATAGCACTTATGGTGCGGGAAACGCCGCTATTAATCCTAACTTGCTTGGCGCGCAAGTACATATATGCACATGGTGGGCGTTTAGTACTTTAATTGAGCCTATATCCAACGGTTCAGAAACGAGAGCCACGCACTGGACTCAATCAACCAACGGCTCAAGATTAAAATTTTGAAGTCAACCCAGTGGCATTCAACAAAAATTTGACCATCAACATACCATCACTGCGAGAAATTACTGGGTGGATTCGGGGCCCCAACACCCGTCTCTACCACGTATTTTTGTTGGGGTGCACGTGGCGGGGATGTCACAAGACAACGAATTCTTTCCTCTCTTTTTCATATATGAAAAAATTTCAGTGCACCTTCTACATGTCTTAAATAACACCTCAAAAGAACTTACATACTTATCTACGTAGAATCTACCATAACCTTAGTAATAGATTTAATCAACGTAAATGTATAACTCTGCTCTGTTACTGCAGAATGATGTTAAGTATCAAGTACTCCGCACAATATCAATTAATTGATATGCATGTGTCTGTTAATTTAACTCTCGATCTCGTATGGTTATGCCACATGTAAATCGATCAATTTTTTCTACATGTATTTTTGTACATATCAATCAGTATTTGATTCATGGCATCGCTACACATCCGGTACTAGACCGATTTCCATTGCTTTGAAGTTATTTTTTTTTCTGGCACATGAAATCTACAATGTCACACAAATAAATGATATCTATTATCGTTCGATTCTCCCGATATAGTAAGACAGCAACTGTCACAGTTGATCCACATGACAATGCTAGTATTAGAAATCATGAGTAAGCTCCAGCGCACCCAATAACATCTCCTCTCTGTGGGTGTCTTTATTGTACTGATATATACTAACAGCTTCGTTGTGGGGCACCAAAGCTCACAACCAACCCCCTCTCTCTCTCTCTCTTCGTTGTGGGGCACCAAAGCTCACAACCAACCCTCTCTCTCTCTCTCTCTCTCTCTCTCTCTCTCTCTCGTT

>RsbHLH014

AATTTAATCAAAACGGTGTCATCTTACATTCATGTCAATTTTTTTTTGTAGAGGAGCCGGATCCACATAATTTCCCCTTTTATTTTCTTTTCTTTTCTTGATGGAAAATGCTACAAAGTGCACCTATGTTAGTAGCGGTGCTCAGCGGGTCAACCGAAACCGCCTAAATTGCCTAAACTGAAACCGCCTGCAAACCCAGTCGAGATATGGTACCGATGGAGACGGATCCTTATATTTTTTTGAAGGCAAAAGATAATTTTATTAATCTTTCAAATTATTACAAAGATTAAAAGGACCAAGCAAAAAGTCATCACAATCAAAAGGAAAATATCCCCAACTATGTTGGCACCCAAATCCATGACAATTATATGCCAAACAAGACCCGAAAAAGGAAAATGGAGAAGACGGTTAACAAGATGTCAACCGAATAGGGTACAATAAGAAAAAAAAATACCAAAGAAAATTCATGACGGATTCTAATATATGAATTGTCCAAATACCGAACCGCACAACAACCGCAAATGGATACAGATGGAGAAAATGTGACATTTTACTAAGCTAAGGGAGAAGGAGAATCCAACTAAAAGTTGGAGTACATCCACGAGGCCGAAACCCATAACACCCAAAGGCCATACAATGAAGCCCAACACAACCCAAACGAACACATCCCTAGAGTCCAAGAGGGTTGATGTTATGAATTCATATGCTATGAATATAAGATTGAGAGAAACTTATTCACGACAGAGTCATAAACAAGTTATTAATTCATTGCTCCACGCAATCTCAGTCGTATGTTTCAACAATCAATGATTCGGATTTTAAAAAAATTCTTCCAGTGACAAGTTAAACTCTTCCAAAATACTTTTAGACGCGCGTGATTGAACACCATAACGTAAATTCACGGCTCCGCCATAAAAAAGTTTTTTCTCTTGAAGATTAATGTTATGAACTTTTATCCGAAACATAGTGAATTCAAAGTTTCTTTTTCTTTTTTGGTAGCAGCTTAGCTCTTCCTACTATTATGCTCTCTCTTTGATGTATCTCTTTCGAATTTTTATAAAAAGTTTCTTTTGCCGAGAAAAAGGAAAAATTCAAAGTCATTCAAAGAATGCATTATCGTACATGACAGAGGGTGTATTTTGTGACACATCGCAATCAATGATCAAAATCAGGGTGACAAGGTTGTTGAAGCCGAGATTTGCACAGTACCTACGCTTTCATTTCCTCCATATTTGTTCCAAAAAATCACAATTTTCGAAAAAAACAAAATATTCTGTGGACCATCCTGAAGAAAGACCCAGCTAGCTTGCGTACCCTCTCAAATCAGTTTTAGCTTTTTTTCCTTCACCATATATATATATATATATATATATGATTGCGATAGATGTTGTGCTTTTGTTATACACTGATCAGCTTTTCTCCTCTTGATAAAAGCCTCCAACACAAACCATTCACATCAGCCTTTTTTTTTTCTCATTCTTCAAGTGATCAAAGTTTGAC

>RsbHLH015

TACTTTGTGACTTTTTCTTTTGCTTTCAAGATATGTGGTCAAACTTTACATGTTTCATGTTAAGTCAAGATTAGAAAAAAAAAATAGACACAATTGGTCGATTGAATAAAAAAAATGCATACATCAAGCAATATATTCAACACGTTTAAAAAAGGTGAACATACTCGCTCTCCATGGGGCCCACACCGAGCTCCATTTTGGTCATCTATTCCTTTCAATTTTCAGGGCTATTCTTGCTAAAAATTAACCCAATCAGAGAAGGGTAATTAGTTGATCCAAACATTCAATTCTTGGTCTAAATTTTATACTTAAAAAATTCGTGAAGTTAAGAGAATAAAACTGATTTTTCTATGGAATTGGCAAAATAAAAAAAAGTTATTAAATGAATGGTTCTGGTAATCGAAGTGAGACTCGACATGTACTACCCAAATTAAAATGTTCTCTTTGCTTTAAACCCATTTTATTTGTCATTTCTCCAACGTTACGTCATTTTCCAGATAAAAAGCGTAGCACTTCTGTGGCTTTTTTGCAAATTTTCTTTTGCAGGAACTTGATAAGATAACATCTAAAATAATCTACAAAAGGTACTTTTTATTTTAGAATCACAAAAATGCACAATTTTTTTTTTTCACAACTCAATAGCAAGGGATGGCTAGCTGATGCTATGGTGTCTACTCTCCATTTGAAAACACCGTAATTTTTGACATAACCGGACCATTACAAGCATAACCAAGATTAGTGGCATGTATAACCAAGGCATAACCAAATATTGGCATAACACTTTTTAAGTTATGCCTTAGTTATACATGCCACTGGTCTTGATTATTCTTGTAATGGTCTGGTTATGCCAAAAATTATAGTATCTCCAAATGGAGGGTAGACACCGAAGTCATCCCCGCAAGGGATTATTGTCCAGCTGTTCTAGACAAATGACTTCATTAGAATGGGTACGGTACACATCCGGTATAACTAAAAATGTTATGTCTCTCAATGGTTAATGTTATGCCTCTCAAATCTCAATGGTTAAGATAATGTCATCCCAATGGTTAAGTTATGCCTCTCAATGATTAAGGGCACACGGTATGCATGATTACGCATATATTACGCCTATCAATGGTTTAGTTATACGGGTTCATATAATACGTAGTTGTGTACCGTAGTTTTTTCCCTCTTTTTTATAATGTTACTCAACATTTAATATAGAAGCTACATAAATAATTACCATTTGCATAAAAACATTATCTGCTAATGGTAAAATGTGTTCACATTTTGACCAAGGTAGAAGGAAGAATCAACTTAGACCGGACAAGCCCTTATTTGACCACATGCTCAATACTTAAAATGTGTTGTTCCCATTTTTAGCACGTGTTGAATTGGTATTTTGGAGACCTGTGGAAAAGTCCACTCGGTGGTGGGGACCGTTATAAAACCCAATTCCAACCAGATTTTGAAATTCCCGGGAAAAAACAGAGTCTCCCCTCCAGAACAGAGAGAAATAGA

>RsbHLH016

CCAAAAGTTTTTCCTACAAAAGCTAGAGAGATTGAAGCTTTAGCAAGATAAATAATGAGAAAACTTTGTGATAATAGTTCTAAAAATGTCTTTGAATCATTGAATGGAAAATTGTTCAACTAATAGTCTGTTTTGTTGGATGTTTATCTCTTGAAGAATGTATTTTTTAATATGAAATCTATAAAAGCTTACTCCTACCGTATCGCAATTTTTGGATGTTGCGTACTGCATTTCCCTTTGCAGTACGAGGAACCAACACGATTCGCATTCCCTGTAACATAGGGATATGCATCTATAGATCATAGAATACCCTCTGTCTAAGACATTTTATATCTGAAAGTCACGCATGATAGGTCTTGTTTGAAATGGAACTAAAGATGTCTTTTTCACTCATTACAAACGAGCTATCATTCAGATAACAAACAAAAACGTTTTAATAATTTGACGTAGTGGTTGGGAGAATTTTATTACTTTTGTGAGCTGCCTAAGACAAAATTTTAGCTCTTTGCGCTTGCGGGGTTGAATATAAAAAAAAGATTCAAATCTATCTCAATAATGGAGATTTTTTGAAATTCAGTAGGGTCAAATTGACCCCACTTGTCTCTTAATCCGTCTATACAATTCTGAATGTGGGCATACAATGTTTTGACACAAAGTAAGGGATGTGATTTTACATCATTCTTGGGTCCTATTCAACACGAAGGATGTGATTAATCATTTGCCCTGATCTGAGAAATAGATCACATGCCTTAACGGTGTTTTGATTGAAACCACCAAATTGCTGGATAATTTTAGTTCATCTTATTGTTCATTTACCTGTGGTTTTGGCTGGTGGAATCCAATTGGAATGTAGAATAACCTCTGCGTGAAGGTACTTTAGGCTTAGGTATCTCTTATGTCCGAGGGTGATGGTTTGCGATACCATCTACTTGTTTAGTCTTTCTGTATATCTTTTATATTATGTTGTCAATTATTAAAAAAAACTGGAAAAAGAAGAGTAGAATACTGATAGATTACACAGCCAGAGGTTGGGATATTTTTATTAATTGTGACATGGAATCTTAGGCTTTCAATATTTTGATGAACTTGCAATGATGAATGACAAAGCTGGTGGTTTATTTATGCATTTCCATCAGAAAAACCTTCTGCTTGTAAGCTTTACATTAATAGATTGTGACACTGAAACTTAGGCTTTCAATAAATTAACGAATTTGTTTTGATGAAATTACCTCCCCATATTATGTTTGTCTAAATCAGACCAAACTGAAGATTCATTGACTATAATGTGATTTTTAATTTATAATATATCAAGTCTTCTTCTTCATACAACCTTAGTTGGCTTAGTTGTTTTGGAGTTCTGTATGTGGGATTTTTTGAAACACACTTGGGCGTTGTACTTGCTTGCGTGCAAATCTTTCTGTTTTTTTTTTTTTGGGTTGATGTGGGTATTGGTTTCTTCTGTGGTTTTATTAGTGAGGAGAAGTAACAGAGGAGGGAAAG

>RsbHLH017

AGAACAAAAAACTTTAAGGCATTCATTACCGTTTGAAAAGAGAACACTTCAAAGCAAGGGTACATTTGACACAACTGCATAGGAGAACTTTTCTTTTTTTACCTATGTGATTAATTAACCTTACAAATTACAAGTTAAAATGGTGTTTATCAAAGGGAAATGATTTTGTCACTCTCTTTTTTGATAACGCCACTCCCCTGCAAGTGTATTTTCCCCTGTAAGTGTATTCTTGCATCCAAAAATACACTTGCAGAGGAGTAACGTTAACAAAAAAAAAGTGTGACAAAATCAGCAGCCTTAACAAATTCTTTTAGCTCATTGAATCTCCTGCCGCAGTTAGCATTTGCCTATTTTGGAGACCTGCAACTAGACAAGGTGGGGGAGTAATTATTCAGCGTTTTCAGAGTATCACGTGGCGAAAATGACAAGTTACGTGGCAACAATTATGTGATAGAGACACTAGCATTCCGTTTTGTTGGGTGATTAAGAGATAAGGAGTGATCCACATTATATGGTGGCAATCTTTTAACTGCAGGGACTGCCACATGGCAATGCCACACTGTACTCTAGGAGTACGGAATAATTGGCCAGGATGGGAGATGAGTGCTGCAACTTGGAGTATTAATCTAAAGATGATAAAGTAATTTAGTCCACATTCAAGTGAATTGAATTGCAACATAAATGCATCTAGGAAATTGCACTAGATGAATGCCCACACTGTAAACACCAAACTCAAAAGAATAGTGAAAGAATTTTGAATTATAGTAACAAACAGATGATTAAAAGAAAATTCATGAGGAATCTCTACACAATAGTTTGACTTAAAAGATTACTTGGGAAAGTACATTTCCTTTTTAAGTTTAAAAAATGACCCAAAATTTTACTAAGACGAAGAAAAATGATGGGTGGACGGAGTAACATTCAGCGAAACAATATATACTTAGAGAAATGCCGTCGCTATATTATTATACCGTAGCTTTTAAACTCATTACGTGATTTTTTTTCGATGACTTAGGTATCCGGACCAACTTACGCATCTCAACTATCACCGCGGATTATGTAATGTTGTTCATACATACATGTACTCACTATACCTTGGATTGAAGCAAATGAGTGTTCTGATTCACTACACAAGAGTAAAAATAGTTTTTAGTCCAGTGGGGTTTCATTTTCTTCTACTACTCTCTCTTTCCCCTCTTTCTTTTCCTATTTAGCTTCTCTCCCCAGCTATATCTGACCAGAAAAGAACACACCAATGCCGAAACTTGTTCCCCTGAGCTCTGTTTGGTTTTGATTGGTTCAGAATCCACCAACAATAATCAAGAGGGAAAAAATTCCAATTAGTCAAACCGAAAACACATGTTCCTTTTTTGTGACTCCATGGTTCTGGGATCTGGTTCAGTTTTGGGTTTGCTCACCTGATTGTATATACTTATAGAGAGAGAGAGAGAGAGAGAGAGAGAGAGAGAGAGAGGTGAAGACTGGAGTGGCACAGAGTCC

>RsbHLH018

CTCGCACTCGCCCGCCCATTATGTGACTTAGAGCGGGCCCTACATGTTCATATTTTGCTTTGATGGGTATGCAAAAATTATACCCCAATCTCGTAAAAATTATATCATACCGTATTACAAAAGTCATGGCATAAACATAGGTTTGACAAAAGTGTACCTGAAATGCACAGGGAACACAATGGCCAATATTGGACCAACAGGGTAACACAACAGAAGTGCGATTCTCGTTGGTAGACTAAGAATACACGCCCCCACATAAATAGGGAAGCTGAATTATTCAGGCGGCCCATCCCGCTAAAGTTTTTATATTTTTAGTACTTATTTTTGTTTAACGAGACGGGTCATTCTTTTACAATATATATTTATTTTTAATTTAAAAATAAATACTTATTTAAAAAATCAGTATTTATTTTAGTTAGTGGATTGGAACACACCCTCACTCTACCAAGCAACCAATTATAGAGAATTTTTTTTTTGCCAAACGATATACATCATTGGGGGATTAGCGGAGTGTTAGTGTGTTACTCAATGGGGCCTCAGAGGCTATTCACATAGCCCTGAAAGGCTAAAACCCTAAACCTACTCTCTATGGAACTTAAACCTTGGTCACCACTGGAGAGAGAGAAGAACAAACCACATATGCTAGTGTGGTGTTCGACCAATAGAGATTGGAATAGGTTTATCTCCCTTGGATTCAATGGCATGGCCCGATCTAGTTTCGAATTTTCATTTTCTCAATGGTTACTAATTCAGTTCGTATAATTTGGAAAGTACATAATTATTGTTCGCAAAATTGTATTTGTTAACTATGTTACTTTAAGTAGCTATCAAAATCATGTATTCCATAAAAAATAGTCAAATTTCATTAGTTAACTTATGTAGTACTTGTTAGAAAAAAACCCTTAGCGACAGGTCAATTCATATGAAAATTTGTCTTGTTTCATATGAAAATTTGTCTTGACTTTAAATGGGTCTATTTTCGATGGACAGTGAAATTAATATATCAACTTGAGAAATGCGTAAGCCGGTGCGAACGGTCATCTCGGGTGGAGAAAGGAGACGTGTCGCTTTTACGCGGCATCATAGACCACAGACCACTCTGCAACATCCGGTCAAGATCCCTTTCTTTCTTTGTAACGAGGCAGCACCCAAATGGAGTAGTACTAGTATTTATAGGAGTATAGTATATTACTCAAAGTCTCCAACTAATACCTCCATCCCACGTGGGTCTACAATCCCTTCCCTCCTCTCTCTCTCTCTCTCATTATTTTTTCGCAATTGAGAATCCCCCTCTATTATTATTATTATTATTATTATTTAACTACTACTCCACTATTTCACCATCCCGATTTGCCTATAAAATCCCCGATTTCCAGAGAGAGAGAGAGAGAAGTCACACATCCAAGAAAAGCATCACCCAATATTATTGTTTCTTAATTTCAACTCTCTCTTCTCTCTCTCTCTCTCTCTCTCTCTCTCTCTCTCTTAGCTAGAGACACC

>RsbHLH019

GAATGAATATAAGTTTTGAATTTTTATTTTTTTTCGTTTCTAATCTCAGGTTAATATTTGGTAGGGCTTTTTCTTTGGCATGCTTATTCAAATGCTACTGCCATAGTTGTTATGTGAAAGTGTGCATGTGCGGAGGATGACTATTGTTTCGCATGGATGCATAATTGGTAAGTCTACCTACCATCCCTCTCATAACCTGCTGTAAAGGGGAATCTGTGCCTTAGAGTGACTTTATGCATGTGTGGTAGCCATGCATAACGCATGTATACTCGTTCTTGCCACAATAGTATTGCATGCTTATCACACGTCCTAATCTCTTATGCACCACCAGAATGCCAATTGGATGAACATCCAAACTCTGTGCCCCCATCTTAGGTTGTGTTTGGATCAAAGGTTGTTAGAGGAACATATTGAGGGAAAGAAAAAGGTGAAATAAAGTATAAGATGATAAGGGTATAAAGTAAAAACAAAATTTATTTCTCTGGCTTCCCTATCCTCAACTTTTTCTTGGTAAATTTCTCTACAAGTCCATGGGGACCATGACCCTAACACAGCCCTTACTTTCTCACCTAACAAAACACCTCCTGGTTAGATGGCACTGTGCCATGCACTGTTCTGGCACATTGTGACACCCGCATTAAAAACATTTTCTGCATTTTTGGAATGATGATAGATCCAAGAAAAGCCTGCACTGTGGATTGAGTATAACAGATGAATAGGGTTTGGAGATCAAATTTAGGGGGGAACTATTAGGGGCCACATATAGCTCAGACATAGCCTACAAAATTGGGTGGTGGATCAGGGATTCAGTGGTCGTATCAGTAGTCTTTTACATCACCCCATTATTGTAAGTTATTGCAAGTGTTATTTGGGGCCCGCAGCCGTGCCTTTGGGTGGATTGTAATTGCAGCTTTTTTGTTTTCTTGTGCAGGACAATCTCAATCAGGGCCTCCGTCAGAAAATGAGTGGCCTTTAATGGAAATGTTCTTTTAGAAACATCAGATAGATGGAGATGACGTATAATTTGAAGATTATGCTTTCTTGTGGAAAAGCATAGGTAAGATTGTACAACTAATTCAATTTGTCCTTGAGGAAATGGGGAAAAGGAAGATCAGTCCGAGATTCTTTATGAAGTGAAATGGGACTAAATTTTCTTGACCCTAAAGAAAAAGGCAGATCCATGTGGGGTCAGTACTATCTTATGACGACCTATAGCCAAACACTAGGATCTATAATAGTCTTATGGAGTCCTTTTACTTTGTTTACTATTTGGCTCTTAATGAAGTTTTAGTAATGTTGAGGTGACTGGTGGATGAGGCCAAAGTTGATGTTCATTTTGACTAGCTGAAGGCTACTAGTGGATATCACACCAACCAACATCAAGATATAAAGATCACACTTATTAGTGCAGAAAACAGTACACTCCAAAAACAGATGGGTGATAAGAAGCAGGTGGGAATCTTGTAATTAGAATTGAGGAAAAAGTTTATCTGTGGGAAA

>RsbHLH020

CCTCCACACACCAAGTAGCGTCGTTGAACACAAGATCATTCCTAACCAACCATAATGACCAAATTGTCGCTTGGAAAGTTGTTCCCACAAAAACTTCTCCAAATTTTTGAAACCGAACCCAAGACGCCAAGAAAACAAGTCAAACAAGGATGCAAGGCACACCCAACTAACATGCCACCAATCCAAAACCAAAGACCACACAAGCCAAAATCAAAGACCACCCATGCCACACCCTTGCCAATTGTGCAATAATATGTCTTGTTCGTTTTGGGGTCTCAAATGTACCCTTAGACACGGTTTTCAATAGATTTTCTCTATCTCTTTTAATTCATTACTCTAAAGAACCTTTCTCAAAATCCAAACCGAACAAGGTCATACGTCTCCACACGCTGATTGGCTAATCTGATTCGTCATTGGACGACCACTGCTTCATCAAAGAACCATCACTACCACCATTGTAGCGACCTAAAACTAGTCCTTGAAGATGAAGGATAAGCCGAAAGAAATGAAAAAGAAGGGAAAAAAAAAACTCTCAGATCTAAGATCGATCGAGAGATAAAATTCTCACTGGAAAGGAGGATTTATTAAAAGTGAAAACAACCATCACATTAGGAACCTATCAAAGGCGAAGCTTTCCTCTCCCGCCGTCACAATAGGGTTTGGATAGGATAATTAAGGCCATTAGAAAATCTTCAGCAGGAAACTGATATCTCTTGAAAAAACCTATGTTTGTAAATCATATGCTCGATTTTCTTCATATTCTGTTATTTCAAATCAAAATACTTGAATTTTTGGCAACGCGGTACCATCAATATTTACAATCAATATATATATATATATATATATATAAATCATTAATTATGTTTGCAAATAACTTTCCCTTCAATAATTAGACTAACACAAGACTGTAGACCATCAATTCAGCAGCATGCTGGTCCACCAAAGTGTGAATGTAGTAGATGAGTATGGGGTTACAATGATACAGCTAACATCCACTGATTCCCCCATTCATAAAACCTTTTTTAACCAAAAAAATAAATAAATAAATGGCTGGTCAGTCGCTTGTTTGTTTGTTTTTGTTTTTAATAACCAAATCTTCGGACCAGTTTGCACAAATCTCGACTAATTTCAAGATCTTGATGTTAACGATCGAACAAACCTTCCAGTGAAACACTCTAATGTCTTTAAAATTTAAAATGTTTGATTTTCACAATTTTATTTGCTTTCTAATGCTTACTTGGCCGCAACCTTCGGAGTAGCCGATGAGTCAGTTGTTGAAATATCATCACATTGTTAATTTTTAAAAAGGAGGAACGTCAGGGAATCTTGGCATCAAATCTCGGAGAAAATCAAAGATCTCAAACCCTAACAGCCGTATCTCTTTCTTTACCATTATCTCACCAACCCTACCCTCCTCCACTAATCGCCTCCATAATTAAGAAAGCTTTCCATTAATCAATCCTGAAGAAGGAGGCTTCGATCGAATATTGTATATCCAAC

>RsbHLH021

AAAATATAACTCACAATGCTATAATCAAACTTAACAACCTCCTTAGAAATAATCAAATTTTGAGCTTTGGATAATCAAAATTAGCAACCTTTTAACAATAACCAAATTTAATAGACCTTACATATTCTCAATCAAATACACCAATTATTATACAAAATTGTTCTCTCTTCCCTTTTCCTCTCTCTCTCTCTCTCTCTCTCTCATTTGAACAATATTTTGGGAAAAAAATATTTTACTAAAAAATTAATTTTTTTCGAAAATAGTTTTTTTTTCAAAATTTTTTTTAAAAACTATTTTCTATTTCTAGTAAATAGTTTTTATTAGTTTCAAAACTATTTTTAGAAAATTTATTTTCTATTGTTCACAGTTTTTAGTTTTTAATTTGTAGTTTGAAAATATGATGTAACAAGTTTTGATTATCCAATTTTGATTATATCATTGTAGACATCTACGTTGCTAACCTTAACAATCTCTTAGATGAATAATCAAAAGCTGATGTGGCATCTTTTGGTTATCAATTTTCGATTATAGCATTGTGGATACTCTAATAGCGAGAAAAAATCTATTAAGTCGAATAAGGGAGAAAAAACTGAGAATACGATGACATTTAAGGCCAATGATTTTTTATATGTAAAAGAAAAGACACGCTATGTATATTTATTTCAGATATTTAAGGTGAATGATTGAATGGTTAGGATGCATATTTAAATAAACAATACAACACACACGTAGGTGTTTATGTCCGGTGTGGTTCGGTTCGATTTGGTTTTGATTAGAGGGTGGCAAAACAAACTGGATTACATTGTCCCACAAAAATGTACGGTGAAGAGTATGAGAAAGCATGGGTCAGTGTGAAATCACACAATAGTTCGACAGTACCGTCACGTGGTATTCCGGGGCCACGTTAAAACCACTCATTCCTGTGGGATCGTATATGAACTCCAAAAAACGTGTGTTTGTAACTGAAATACTTCGTGGCGGTACTCTCGGACTGTTGAATAATTACTCGTGCTCTAGGGGGACGGAACAATTTTTCGCTTGCAACCGTCCAAGTTCCGTTGAATTACGAGAGAGTCCTTCCTTTTCCCTCTTCTGTGCCCTGTTTTCATGGCAAAGAGACAATCCTTGCGTCGGTGAAATTTCAAACTCCCACCACCTCTCTCTCTCTCTCTCTCACACACACACACACACACACACATCTGGGCGTTAAACATTATACTCCAGTTGCTTCCACTACAAGATTTGGATCCCAGCTGCTTAGAAATCAAGCAGTTTTAGGGAAAAAGACTGTTCGAATTTTCCCGGAAAAAAGCCGACGAATGTATGGCGATTCTCGAGCATTAATTCCTCGCCCAACCGCTATATTTTCCCCAAACTCGAACCCCAGCAATTCCAGATTACGAAGAGCCGACAATATCTGTCCCAATTTCTAAGAGGCTCATAATTTTTCAAATCGTGCTCGTCGCTTTTGGTGTTGAAATTGTAATATTTTTGCTGAAT

>RsbHLH022

GGAAAATCGAATGTTGCACTTATACGCGTCATTTTATTGTTGTGAGAAAGGATCAACTTACCAAAAATAACATGCGACCTTCGTTGGTTCTTGATAGTTCTTGACTGATTTGATCAATGTATTCGTTATTGCATCGGACCAATTGCGCATGCATGTCTTCATTTTTTTTTTATTGTCCATACACGCATGTTTCAGATACATTCAGCCTACTTTTTTCACTTGCTGAGAAAAACAGAGGCAAAAGTACATAAATACCCCCTCAACTATTATTTTTTTCCACGGAAACCCCCTAATCTTTAAAAACGTCCAAGGAGGCCCCCTCAACTATTGGGAAAGGAAAAAAAAAAAAAGCAAACTCCGTTAACGGAACAAATGAAATGACTAAGATACCCTTTTTATTATATACGGTGAGATGATTCACGCTCTGCTCATATGATCGATCAGTGCTCTCTCTCTTCCTCTCCCATGGCAACTCTCTCTCTCTCCTTCTCTCACTCCTGACTTCGACTCTCTCTCTCTCTCTCTCTCTCTCTCTCTCTCTCTCTCTCTCTCTCTCTCTCTCTCTCTCTCTCTCTCTCTCTCTTCCTCTCCCATGAAACTCCATAGCTGCCTATGAACAGAGAGAGAGAAAGAGAGAGAGAGGGAGAGAGAGATTAGGGAGGGGATCTGGAAATGGTGTGGGTCGAAGGGAGGGAGAGAGAGAGAGAGAGAGGCGTCTTAATGGGGAAGAAGATGTATATAGTATGAACAAAAGGCGTTTTTGTCCAAAAAAAGCCCAAATCTTTGACAAGTTAGCTTCCAGCTAACAGTTCTAAGATCGAAGGGTGTCACCTGGGTAACGAAGAATAGTTGAGAGGGTCTCCTTGGCCGTTTTTAAAGGTTAGGGGGTCTCTGTGGAAAAAAAAAAATGATAGTTGAGAGGGTATTTATGTACTTTTGCCAAAAATAGAATGAGGCATATATAAAACCATGCATAGAAGACATGCATCCTTCTACAAGTGTGGCACATAAGTCGTCGAAAATGTGTTAGCAAATGGAGGAGCCGACTAATTGCTTGGGAAGAATGCTTTTAATTGTAGCCATTTTAAGTGTAATTAATGTTTTAATTCTTTTTATGTTACAACTCCTCTAGGATGATACTATAGGAGTTACATACCCCATACTTATTTCGGTTTAGTTTTTTTTATATATACAAATCTCATTTTAACCAAATAAAGAAATGTTGGACCACATTTTTTTAAATAAAAGGAGAGGGTAGGGTGCCGAGCAAAAAAAAAAAGGAGAGGGTAGGGTTCAGCTTGTGGACAAATGTAGGACCAGTAAGCAGAAACAAGTCTTGGTTTGTTTAGGTTAGCTCATGGGCCTCATTTTTATATCCAATACCTTGTATGAGAAGAAAGAGTGTATCAATATACACTGCATTCCAGGCATTGATTAGTACTTCAACTTCTTGCACTAATAATTCAACACATTCTCATTCTGATTAGAGTTTTCTCAGTA

>RsbHLH023

TTAGCGTCCACCTTCAACAACCACCCCTAGTGCCCCACTAGCAACGGAAAGCTAGCTGAGATGCGTGCTTTGTATCAAAAATAAGTGTTGTTGATTTGGCTGAAGGAAGAAAAATGAATTGAATCATATTTGGCTCAAACTAAATAAACTGTAGTTTGACTGTGTTCTTTTTGCATGTCAAAGGTAACGCAATCAGACTCTTATTGATATGCAATTGATATGTTTTTGGCATGGATGGTTTGTACAAGACTAGGAGACCTATTAAATGGATGGTTGGAATCGTGGCGATTTTAAGTTCGCATCACTTGAGAATAAACGGGAGATCATAATCTATGGGGATCGATCCAGAAGACCTATGATGAACTCTACAAGTATGAGCATATATCGGGGTATAATGCAATGTACATAAACTAGTGACAAACCGAATAACTATGACCGTGGTGGCCTAAGTTTTTTCATAACATGGAAATTACAGGATATATCATTACATAAGGGGAGTTTTTACAAAACCTTCTTTCAATTTTTCCTCTCTATCATGGGTAGGGGTGCAAACGAGCCGAGTCGAGTAGAGAGAAGCTTTAGGATGTTTGAGCTCAACTTTTTTTAAATTTTTGTGAGCTCGAGCTGACGAGCCGACTCACTAAAGGAGCAACGCTCTAGTTCGAGTAAAGCCAAAATATTTGATCTCAAACTCGAGCTCGGTCCGAGTACAGAACGCCCCTCTATAAACAAGTCGAGCCCTTACAAAGAAGCAACACTTAATTACACGAGGCAAACTATGACTAATTCAAACTTGACTCATTTACTAAATGAACTTAAATATTTCAAAATTTAAGCTCGGCTCATTTACAAACAAAAGAAAGTCGAACCCTTAACGAATCGAATCTCTATCTAGTCGCGAATAGGCTCGTTTGCTGCCCTCCATACCAAGTAGGGAGTAACAAATTTCTTGATTTTCTTTCTTCTTCATGGATTCCAAATTTGTTGATCCCACACCATTCGAGGTACATATATAGAGTAGTCGTATTTCCCTAGGGAGTATATCTACGATCCCTATTTCTACTTCTTGATCCCTCTATACGATCCCTATATAAATGCAAGAAAATGACGTATCATCTTCTTCGATTTAAAACTCTGTCGGGTCTCCGTTCCCATCCGGTCCCCGCCCTACTCGAGCATACTCCTATTTACACCACCACCAATCCCACGGTCCGCTTCTCTCTCTCTCTCTCTCTCTCTCTCTCTAAACCAGACACCGTAACAAAGATATATATATATATATACATATATCGAGAGAGAGACAGTATAGAAACAAGCATCCAAGCAACGTTGCCTCCGCTACACTTGATAAAAATTGTTCAGTCAAGTGAAGATTACTCCACAATTATCATTTTCCCTCGTTTTTCTGAACTGGGTTTATTTCCAAATTCCCTAATAAGTAGTGCATGAGTTGAATTGGACTAGAGCTCAGTTAGTTGTTTGAAATTGGTGCTCTTAATT

>RsbHLH024

ATTCTTGTAACACCCTATGTAGACCACTGACCCAATAACTTGTAGGAGTTTAATCACATAGCCCAAAATACTTCAGCTCAAGTTACTACTATAGTTTACATGGTTCTTCTTGTATACCCATGTAGACTTCTGATGTGGGATGCGGTATACCGGTATCATAGTCTCCCACTTTTATGGCCTCGCGCCCTTGCAAGGGGTAGAACGTTAGAGTCACCATGATAGTGGTCACATGCCCCCTGAGCCCAAAGTTATCACACTATTTCATGGGTCATTTCCTTGTTCAAGCCTATTTGGTCGTGTACACCGGTTGATCTGATACCACTTGTAATGCCCCATGTAGACCACTAGGCTAGTAACTTCTTAGAGTCTAACTATGTGACCCAAAATGTTGGGCCAAATTACTAGCTAATCAATAGTACCTTAGTGGTTCCTTAAATACCTGGATCATCCATATGACATTATTGATCATGTCGTGGGAGTCTAGAGGGCGAAGCCCCCGAAACCTAGACAGACCTCTTATAGTAATTTGTGTCGTAAACACAAAGACATCATTCTTGTTATACCTAGATGTAACTCTGCTATTCACTGCGTTCGATACATACTGAAAACTGGTGTAACTTGTGAATGCACTAGCTACATAATACGTTGGGGAATTGTGTAGAAATCTCTTGAGTAATTTTTTTTTTTGTGTGTGTGTGTGTGTTCATTTAATTGTTCTTTCCACGCTATGTGGGTATTTGGCCAATTCTCAACAGCAACATCTCTTTTCAAACATCGGTGTCTGTATCATTAATTGTACAAAAGATAAATTTAAAAAATGATGTTATGACTTTTGAGATAAACTGTAGCTATAAATGTAGTGTGACCATTTTTGTACTATATCTTCAGAAGACCATCCCAGTAGCATGCTGGTCCACAAGTGTGAATGTAGTAGGCGAGTGGGGATACAATTATAATACGACAAGTTTTTGGGTGCCGAGCAGGTATTACTGGCGGTACGTGTCGGTGATTCGGACTGTTCAAATATGTTTTGAACGGCCCATGTTTATTCTCTCTCTCACTCCTCACCCCTCACCCCATCACTCTCTCTCTCCTCTTTCTCTATCGCTAAATCCGGACCGTCCAAAAGGCATTTGAACGGTTCAGATCGCCGACAAGGTACCACATATGTGACACCTACCGGCACCCAAAAATTTCTCCAATAATAGAGCTAATAACTACAGTACGCACAAATAAAACCTTCTTTTTTGAGAGGAAAAAATAAAGGGTTGGTGAGTCACTTGTTTGAAATATCATCACATTGTTACTAATTTTATAAAGGAGGGATTTCAGGGAATCTTGGAATCAAATCTCAGAGAAAATCAAAGATCTCAAACCCTAACAGCCGTATCTCCTTCTTTACTATCATCACTACCCTACCCTCTCCATTAATCGATTCAATAATTAAGAAGTTAGCTTTTCATTAATCTGAAGAAGAAGAAGAAGGAGGCTCGATCGAAC

>RsbHLH025

TGACCAAAGATCTTGAGCACTATCAGCAAAAGTAAAAATTTCAGCAACTTTGGGTTCCATAGAATTGAGAAGTAAAGACATGGTGTACTGATTTTCGGATTTCCATTCACCATACTTCTCAACTTCATCAGTTTTAGGAGGCCAGGTTTGGACACTGTTGGAGGGGTTTTAGTTCCATCAATATGCCCATACAATGATTTTCCACCGAGTGCTAGTGCCACGACACTAGACCATGGAACATAATTCAGTCCATTAAGAAGAACTGATGAAAGCCTTGGGGCCGAATTATTTTCTGATTGTCCACCCATCGGTCCCGAAAGAAATAGTACGAAAACAACTGTCAAGCTTCGACAAGATTTGCAGGAACGTAAAGAAAGGGGTACAATACCTTACCTTCTTCCTGCTCTGATACCATGAAGAAACGGAAAAAGAAGAAGTAATTTACGTGATTCAGCCACAGATTGTGACTTACATCCATGGGAGAAGGTAGGAGAATTCTTATTCATGGGTTGCTATAACAGAGCACCCTCAATATAAATACAATTACATGAGAGCAATCAAGGGAATAAATCCCACGGCTTAAGCAGCAAGATAAGCATCCAATCTTCACATAATAGGCAACATAATCACCAAATAATTTGTTACCAAATATGAACATATTTTTCCTCATTCTCCGCCAACACTACCCCTTTTCTTAGACATCGAACATCTCCAATGGTGGAGTTCGTATACTATCTACTATAACAATGAATAGTGACTAATTGCTTGTAATTTATGGCTTTGCCAACTGTAGCTCCACCATCATGTTGTCTCTTCACCATCATGGCGGTGCATGGTCGCGTTGGAAGTCGCGAAGACGAAAATCGCCATTGATGGCGGCGGCAGCGGTCGGTGGAGGGAAAGTCGCCGGTGGTGATAGCGGTCGCAGTGTTGGTGATGGTTTGGTCGTTGTCATCCTCCTCCTCCTCCTCTCTCTCCCTTTTATCTTTCATAGTGTAGAACCAGATGAACACTTTTGATTTCTTTTCTTGTTTTTTTTTTTTACTTCAAGCTGACGTGGGGCTCAATCCACGTCAGCTATCCTAGGTTGGTGCATGTGTTCGTTTCTTGGTGCATAGACTTATTGTTAGAAGCTTTTGAGTCAATTTTACATTTGTATATAATATAGATTGATTGATTGATTGAAGTCATCAATTTCTGATTTTCAGATTGGAATTAAAGATCTTCAAGATTTGATGGGTGGTCCACCAGGCCGTGTGAATATTTAAAAGTGGGTTGCAGTTGTACAGAAGCTAGCACAGTAGTATATCCAAATCTCTTCTTAAATAATTCCTCGTTAGGTCGTTTCTCCAGCTCCTCGTATGACTTTCTCCGTCAGTTTCTGGAGAGAAACCGATCGAAGGGAATCTTTGCAGTACTCTCACAAACCCTAATCAACACTTCTCCTTAATTCCCTCGAAGCTTGACGTGTACACTCTTTCCAAAATACTAACTCAAAAG

>RsbHLH026

TGAGCGACTCGGATCATTGAAATCCGATTGGAAAAGGCGAGTCCTCATTTTTAAATGGGATCCCTCACGGTACCGACTCTATATATATATATACCTATACTGATATAGATATATTGTATGATATATATATATATATATATCCCCAAGGACATGTTGGTCCCTAAAATGTACAAACCAACACCCAACCTAGTTGGAGAGGAGTTCAATTCAATTGTTCTTTGCTGAGGTACGAAGAAGGACAGCAGCAGCAGCTTTATAAAGGAAAATCATGGACCGATGGACGGTCCAGATTAAGACTATCTCTTCCGGTTCAAAACATAGTTAAAGGGTATCCGGATCCTTTTATATTTGTGCCAACAATTCCAAAAAACGTGTCCCTTATAGAAATAAAATAGGAAGTAAACTAAAGGGTATGTGCGGATCAAAAAAAAAAAAAAAACTAAAGGGTATGTGATTAGTCAAGATTCATATAAACTGATTCAGATAAACGGTTATAATAAAAAAAATCCAATTGCAGTGTGAACGCGCATAAGATAACCATAAAATCCTAGAGTTAAGCAATTTGACCTCCCAAGTCCCAACTGTAGTACACGAAACCATATCATTAATCCCCCGATCCAACGGTTCTTACTGATAGGATTTTGTTCCTTAATAAAAAAAAACCTACAAACAAGACGTGGGTGATGTGACTTATTTCCCATTTACTAATTGTATTTTGTAATATTTCAAAGTGTCCGGATCAATTTATCCGACTCTTACGCTCAATATTTACTCTCTTAAAAATCATAATTGTAAATGAAATAAATTATGAGAGTGGAGAACCCAATTAAAAACAAAAACACACATGACAATAGGCCTTCAATTCCTCCCTTGACTCTTTTCAAAATTGTTATGTTGGACTTTGACAATTACTTTTCCAATCTCTCTTCGTGGTGCTCGGGCGCTCTCTCTCTCTCTCTCTCTCTCTCTCTCTCTCTCATGTCTTCGTGTTCACATTTGGTTGCTCCTATTTATCTGGTCATGACTACTTTTCAAGCTACCATTTCGCAAACATATATATATACATCTCTTTCCCCACCTAGGGGCCCAAACCAACAACTTCCAAGAGGTGGGTACCATTTCCCAATTTGTCAAGAAACATGTAGTGTGCTGTTAATTTGATCATATTCTGTATGCCCTCCATCTTCCGCTACTTTTCAAGCCCTCTCTCTCTCTCTCTCTCTTGATTCACAGAACTTTCCAAGTCTGAACTCATCGCATTCAAAATCATTAGACCGTAGTACTATTATTCTTCGATTTCTCACTCTTTGATCTGTGTTTTTCCAACACTCCGTACATTTAGGTCTCAACCCATCGCACTAGTTCATGGGTATAATACCATAGAGGACAAAGGAGGGAGTACGACTGAGAGAAAGATCATCAGATTCAGTAGGAGGAATCTGAATTAATCTCGATCATGAACAAAGTTTAGTAATCTGAATCTCATATAACTACTCCAGT

>RsbHLH027

AGAGAAAAAAGGTATTGACGATTTTAAAAACCAAAAGATTAACTTTAACTTTCAAGGAGGATGCATGAATTCATAAGTATATCTATGACTCTACTAGCATGAAACTTTTTAATTAGGAAGACCTTTAAAGATACAATTACTTTCGTCTAAACCTTTTTACTATACTACAATTTGTACCTGAAAAAAGGAACGTCATCCATCCATCACTCCAAAACCTACTCTTTCATTTTTCATGCGAATCACGGACGAAACTCTCATCCAGTTTTATTCTCTATTTCTATCTAGTTTTATATTGTGGTAATCTTGTTTGGGCCCCAAAATAATCATCGGGAACCGTTCATATCTTAAACTTGTAAAATGTGTCTCTTAACATAAAGTAGATCGTATTATATAGCTATATAAATAGGGGGCGGTTCAGGGACAAATATTTTAACATAAAATGACACATATATAGCATGAAATTCATTTCGGAGTCTCACCCAAATAATCGAAGCCGTTCAAGTTGTTTAAAATATGTTTTTAAGAAATTTCATAGGAAATCAACTCAATTGGATATTGATAAGGGCTTGATCAATTCATTTAATTTTTATCTTGTCAAATTTAGGGACAATGTTTCGATCAAGTTTTTATCGATATCTGAATAAGTTTATTTTTTGCTAAAATCCTTGCATACATGTTTTAAACAAATTAAACAGTTCTGATTATGAGCGTGGGACTCCGATTTATTGAGTGTTACTTTATTTGTCAAAATATGTGTCCGTTAAACTGCCATTATAAACAATTTGTATGTGAGGAAGAGAACTACATTTTCCAAACTTTCACCATCTATTTTGTCATTTACAAATATAAACCTTAACATTACTTTTATCAGATTTATGACCTGGAACTTGAGAAAAGTGAGGGGAAAGAAAAGACAAAAAAAAAAGGGAAGAAAAATGAGAGAAAAAATGTAGAACGCACAATATTATAATTTTATATTTTTCTCTCCTCTTCCTCTCCAAACCAAATAATGGGTAGAATTTTTTTTGAATTCTATTCGTTTCTTTTTATTTTTCATAAGTTTCAAACAAACCATTTACTTCTATATGAAGTTTATAGAAAAGTAATTTTGTACAATGTGACGGAGGTATCCATGACAAGCGAGTGACTCACATTCTATCAACAGAGCAGTGACATCTCACATAATTTAAAATGTCGTATGCAGACAAAATCATCAAAGCACATTTTAAATATGAACGGTTTGAATTATTATTGCAACGGGTTCGTGCGGCCATGGCCCAAAACTGGACTAAAATGGCGAATACAAAACTGGATGGGAGCCTTGTTCCGTGAATAACATTTCACACCTGAAACAAGAAACACGACACAGCCTTTCTCCCGACCAAATTCCAAAAGCAGACCACGAAACACACAAACCACAGTCCACTCAAAACCTAACCCCTCCTCCTCCTCCTCCTCCTCTCTCTCTCTCTCTCTCTCTCTCTCTCTCTCTCCCCCGAC

>RsbHLH028

ATTCCTCCTCACCGCGAGACCTCCCTTCTCTCCCTTTGACTAGTCATCAACCAATCGCACCTCTCCAGCTCACGGATAGTTACACGTAAGCAGTCCTATACGAAACCCGCATTCCCCACACATAGCAAAGATGAACTTGTTTATGTTTTGTTTTCCATTTTGAGAGGGAAACACAAAATTATGGACACATCATCAACCGTGCAGCCGGTTCGGTGGGTAGAGAAGGAAGGGGATATGGGCCTTGGTGGGATAAACATATTTGATAGACAATCTAAGAGCCATCCATGGTATTGGCAAAGACCTACCTACTTACCCCTATCTTTTCACAAGAAGTACCTAAGTAAACTTTTAAATCATTTATATATTTAACCCATTCTTATCCATCCTTTTTAACTACCGAACAAATCCTTCCTTAGTTAATCTTTCTTAACTAATTTTCCCCTATAATATCCACCTTTCTTTTTCATCATACCAAAATTGTATCCAACTGCCAAAGGAGCCGTTAGATTTGCTCTCCTGTCCCTAATACCTTTTCCATTACCCTTGAAAAATGAATTAAATATGAAAAATTGGAAATGAAGATACTCAAAACTCAGATGTGCCTTAAAAAATGGAAAAAAAAATTTGCAATCTCTGTAAATACTGCAATTTGATGGGACAATTGTGTATTTGGAGAAGTTTTTGCATGCAAATTGAGTATTATGTGGCAGTATTCAGTACAAATCGTATTTTAAATGATTCAAATCTAAGCAGTTCAAAATACATTTAGACGGCTTGGATGAACTGCAAAATACCACATAGATTATAACCGTTTTGTTCTCATCATTTCCTCCCAGGGGACAACTCATCAAACTGTACTGCTACCGACAAGAAGGCCACAGGGCACATAAAACCCGCGCCCCGCAAAACAAAATATCATTCCTCTTCTGGCTTCTGCTCATCAAATCTCATCCTGTGATGCAAATTTCCCAATTACCCAATCATCGTTCACAAGACAACGAGCTCTTCACGAGGGCATACCTGTCATTTCGACAACTCCCACGTCACCCCTTCCCCTTCCAATTCTGATTCCCCGACAACCAGTACAAAAATCCCAACCACGTGCCCACCACACGCCAAATCAAAACCCCATTCCATTCTCCTCACACCACCCGTCCGATCAAACTCTCCCTTCCCCATCCAACGGCGTATAATCATCCAACTCTCTCTCTCTCTCTCTCTCTCTCTCTCTCTCTCCTAAATTCAAATACGCCCAAAACGGAAAAGTGACCTTTTCCCGGCACCTGAATCGTCCCCAGAGAATGGAAGAAAGAAACCGACAAAAACAAAAAACGAACAGGTTCATCACCGGCACTTGTCGGTAACTACTTTTTGCCGCGCCCAGCCCAAACGATTCAGGCATTTGTACTACCTCAGACAACCAATACTCACGCGCCACGTGTCCTCACCCCCCGTAACGTCTGTAACCACTTTACACACTCGCCGCCCCATGGATTCCCC

>RsbHLH029

GGGGACTATTTATGGGTGGGTTTTGGGGTGTTATTAGTTAATACTCCCGAATAAGGCCGAAATAATACCCCCTCCTCCCCCATTTCAACTACAAAATAATCTTATCCCCTCATTTTATCCCTTGTCCAAATCAAGTACTAATTTATCCCTCCATGTATAATACTTCATTCAAACCAATTACTATTTAATCTCATTCCATAAATATCACATCAATGCGGATCATCATTTATTAATCAATATCCCCTCTATTTTATCATCCATTCATAAATAATATCCCAAATTCTTATCCCTCATCCAAACGAGTCCATATGTTATCACCTTGGGCCCCTCATAATCAAATATATGGCCCGGCCGGGAGGCCATAATCATCATGGAATCATGTTCATTTGCCATCGTAAGATACAGTGAACCCAAGAGAAGAGAATTTAATTGGCAATGTCTAGCTAGTTAAGCTGTTTTCCCTGGTTCTTTTCTGGATGTATTAGCGAACCTTGGGTCGATTTTGATTCTCTCTTTTTGGTTGGCGTCTTGCCAACTTCTTTCTGTTTTTCGGTGGCGGATGATATATGATTGCCTTCGGCCTAAGTGCCACTATAGTTGGAATGAAGGTTGTGGGTTGTGTTGCCGTTTTCATGTGTTTAGTTAATCTTTGTAACTTCGTTCCTTTGATTAATAAAATTTCTTTGTCGATTAAAAAAAAAAAAAACTGCAAAACTACTATACTACTGTATTCAGTATCAACTTGGGGTCCAGTATTAATAGTTTTTTTTTATCCGTCCAGTATTAATAGTTTACTCGCCATGATTTCCACGAGTCTACCCAGACCTAATTCCTAAATATGAGAGAGAGAGAGAGAGAGAGAGAGAGAGAGAGAGAGAGAGAGAGAGAGTTTCTACACACATACATGCATCTATATTTATATATAGGGAGGAGGATCGAGGAGGGAGAAGCACAGGAAGTCTCAACTGCGTGGAGAATTGCACACGGTACAAGAAGGTAGCTCTCTCTCTCTCTCTCTCTATGCCCATATGATCAAAAGTTATGTGTCTTGAGTTTTTTTTTTTTCCGTCGATACTTTGTTTTTTTTGGGTGCAACGGAATTACGTGGATACTTTATTGATTGGGGATTACATGGTGATAGTTTTTTTTTTTTTTTTTTAATCAGCGTTCATTTTAATCGTAAATTCGAAAGACTAACATATATTGATTCATGAAAATAATTTATGTATTGTAGGACGTCCTGGTTTATATATGCAACGGAATTACGTCGATACTTTATTGATTGGAGATTACATGGTGATAGTTTTTTTTTTTTAATCGTAAATTCGAAAGACTAACATATATTGATTCATGAAAATAATTTATGTATTGTAGGACGTCCTGGTTTATATATCTTATAAATGTTTCGATTGATTATTAGTTCTTCTCTCCTTTTTATATTTTTCTTCATAATTCACCTCCCAATGTAAAATTGAAGGAGGGACTATTTACCGACAGAAA

>RsbHLH030

TTTTCTTTCTTTTTCACAATCATGCAAAGTGACTCGTAAAATGCACACACAATTCGACAGACAACATTCCCTATCGCTCATACGTGTGGGCTTCTGCCAATCGGCTCATGTGAGTGCGTGTGAATATTCTGTATTTAACTAGACTTTTGCTTTTTTTTGGGATCAAATTCACACAACTTTTCTTCCTTAAAATCACCGAAAGAATCTTGTCTCTCAAATAACTACACATTAAAAGAAGAGTTTGATCAAACACTAAGCGCACTTCAATCAATGTTTATGCATAAAATTCTACCAATTAACTAAACTAAAGAGATTGGCCGTGAAAGGTGTAGAGATAATCTTAACCAAAAGCTACCTTAAAAGAAAAAGAGAATGCAACAGGAATTTATCTTTCGATGAATTGAGTGGTATCCTAGGGGGAAAAAAAAAATAAAGGCTCTTGTTATCGTACAGTTGAAATCTAGGAACAAAATAAATTAGTCCTTGTCCTTTGTAACTGGACTCCCACACATAGTTGAAATCTAGGAACAAAAATCTACTTTGATTTCAGCGGAGGTTCCTATATTTCCTTGTGGGCCATCATGTATTTGGACAAGTAGATGCAAGTTTTTAAATGACAGTACCGGGACATGGAACGGTAACCGTGGTTTTTTATTAGGATTGAGCGTTGTATAAAATGTTATGTAACAAGAATCGGTTGTAGCGCTCGTAAATTTGTAACAATAATTGTGACAAAAAAAAAATCTCAATGCCTAAATCACTTGTAGTGGCTGAGTATAAGTATTGGATGTGAACGGGTCTGTGAATTGGACATCGGTTGATATATATGTGCATGCAACTTTTCATCTCAATTGTTTATCAAGGTGTAACAGTATCGAAATGCATGCGCAAAACTAATGTACCGATTGTATAATGCTACTACTTATGTCTGTACCCTTTCTGGGGAAGGGGATACGGTATTTCCATATACTAACATTAACCACTCAGATGTATAACATTAACCACTAGAAGGCGTCAGAAAAATAAATCAAAACAGAATACTATCGGCCATAAAATATTCTGAATTTGGCTCTCGAAGAAAAAATTCTAAACCTCAAAACGGAAAAATGAAGTTGCAAACAGACCTCAAATGACACTTTCTCACATTTTTCATATGAAAGATGAGAGTTCGATTCTCACCAGGAACATAAATACTTGATGTGGTGGTTCATACCCATTAGTTGGCCCCACCCATATGCCGCTTTGCTATTTAAGAGTATGCTAGACATTTATCAAGTAAAAAAAAACTCAGATTCAAACCCTAAAGACCTAGCCTAGGTGGACAACCATATCTTACTTAGATTTTCTGCTGACCCCATATGACTACATCAACTTCCTTCATTTAATACCATGGTCACCTCAACTAACCTGAACCCCCTATAAATATGTCCCTTCAAACCAATCCCACCTCACCCTCATGTCCAAAAACCAATTGGGTAACTTTCAGATTCACATAAAAAA

>RsbHLH031

CTTTTTTTTTTTCTTAGTAATGCAGGGTGTCCGGACCAGTTTGTACGTACCTCAGACTAATTCCTACAGCTCCTCCCACAGCTGTTTCACACGTTTACGCATGAGGGTGAGGTAGCCCCAAAAGTTACTTTTCTATTTTATTCTTTCTTGTGAATCAGGACTGCAAATGAGCGTGAGTACTGTCTACTGAGGTTTCGATCAAGAGCCGAGCGCAAGCCTATTTTCTGTGGCTCAGTTCAAGCTCGAGCCAACCCTATTTCTGATTAGCTCGTTTGAAGCCCTAGGAACTCGAGGCTCAGCTCGATTTAGTAAATGAACCAAGATTGGCTCGTTTATAAACGAGCCGGAGCAACCCAAGTATTGAAAGAGCCAAGCTCGATCGAACACAACAGAGTTCGACTCAACTCGTTTGCATTCCTACCTACTCTTGAAGTCTTGATGTTATCACTTATCTACTACGAGTATATTCATGTTAGTGGAAGGGCAACCAGTGTGAGACGTTGAGATCAGCAACAACTGTGCCTGTCCGCAATCCAACGTCGCTAAGGCAACGGCAGCTCCAGGGTTTTAGTTAGCAGTGACGAAAACATAAAGATATTCTTGATGAATTGTGGAACGGAGACACATATATGTTCCAATGAAGGTATAATTCAATGAAGCAAATCTAAAATAGTAACTTGGCCACTCTTAATCGAATAATGAGCACATGCCCATTATTATTCGCCGAACCCAACTTGCTCATCCTCCATTGTGGCGCGGGTCGATTTCGCGGGTTTGGAGATTTTCTGCCCACCCAAAATATTTTGTAAAGCCAGTAAAAAACCATGTTTGATGTCAAAACATATATTTTACCTATCTTTTTGAGTTTTACCCTTTTTTTTTACCTAAATGAGGAGAAATTAAAATCATTTTTTTCTCACCACCTCGTTTCACTTTCTTATTTATAATGTGTCAATTTAACCCAAATTTTGAAGGGAATACATAAGAACTATACTTGAGAAAAAAAAATTTAGCTCAATCGGATACCATTAAAGTCTTTTTAAGAATTGGTAGATTAATTAATGAAAGAGACTTTATTAGCATCCGATTGAAATAATTTTTTTTAGAAACTTGTATATTAATTTTAAAAAATAATGAGCGATTTTGATCATCTTTATGGTGTCCGATTAAACTACGTAATTTTTAAAAATGTTTGAAATGGAGAGGTAAAATAGAGATATAGACATTTGTGATTGGAGATTGTATGAATAAAATCATCCTCTAAAATGAAGTTTCAGAGAGTAATGGCATGGAGATACTTATCTGGTCATTCATCTCGGCAATATAGTCATTCATTTCGGCAATCGATAGTTTAAATATTTGCGGAGCAAGTGACTGCTCAAATTTGCATGCATTTAAATACAATTTGAACCGTTCTTACCGAGATGAACGGTCGGATTAGCTGCTTGACACTGCTTTCGAAGGGATGCGGTGCACGGTGAGAGGTGAACTTTGGTGGGG

>RsbHLH032

GTTTTTTCTTCACAATCATGCAAAGTGACTCGTAAAATGCACACACAATTCGACAGACAACATTCCCTATCGCTCATACGTGTGGGCTTCCGCCAATTGGCTCATGTGAGTGCGTGTGAATATTCTGTATTTAACTGGACTTTTGCTTTTTTTTGGGATCAAATTCACACAACTTTTCTTCCTTAAAATCACCGAAAGAATCTTGTCTCTCAAATAACTACACATTAAAAGAAGAGTTTGATCAAACACTAAGCGCACTTCAATCAATGTTTATGCATAAAATTCTACCAATTAACTAAACTAAAGAGATTGGCCGTGAAAGGTGTAGAGATAATCTTAACCAAAAGCTACCTTAAAAGAAAAAGAGAATGCAACAGGAATTTATCTTTCGATGAATTGAGTGGTATCCTAGGGGAAAAAATAAAAATAAAGGCTCTTGTTATCGTACAGTTGAAATCTAGGAACAAAATAAATTAGTCCTTGTCCTTTGTAACTGGACTCCAACACATAGTTGAAATCTAGGAACAAAAATCTACTTTGATTTCAGGGGAGGTTCCTATATTTCCTTGTGGGCCATCATGTATTTGGACGAGTAGATGCAAGTTTTTAAATGACAGTACCGGGATATGGAACGGTAACCGTGGTTTTTTAATAGGATTGAGAGTTGTATAAAATGTTATGTAGCAAGAATCGGTTGTAGCGCTCGTAAATTTGTAACAATAATTGTGACAAAAAAAAAAAAATTTCAATGCCTAAATCACTTGTAGTGGCTGAGTATAAGTATTGGATGTGAACGGGTCTGTGCATTGGAACGGGTCTGTGAACGGGTCTGTGCGTGCAACTTTTCATCTCAATTGTTTATCAAGGTGTAACAGCATCGAAATGCATGCGCAAAACTAATGTACCGATTGTATAATGCTACAACTTATGTCTGTCCCCTTTCTGGGAAGGGGATACGGTATTCTCATGTACTAACATTAACCACTCAGATGTATAACATTAACCACTAGAAGGCGTCAGAAAAATAAATCAAACAGAATACTATCGGCCATAAAATATTATGAATTTGGCTCTCAAAGAAAAAAAATTCTAAACCTCAAAAAAAAATGAAGTTGCAAACAGACCTCAAATGACACTTTCTCACATTTTTCATATGAAAGATGAGAGTTCGATTCTCACCAGGAACATAAATACTTGATGTGGTGGTTCATACCCATTAGTTGGCCCCACCCATATGCCGCTTTGCTATTTAAGAGTATGCTAGACATTTATCAAGTAAAAAAAAACTCAGATTCAAACCCTAAAGACCTAGCCTAGGTGGACAACCATATCTTACTTAGATTTTCTGCTGACCCCATATGACTACATCAACTTCCTTCATTTAATACCCTGGTCACCTCAACCAACCTGAACCCCCTATAAATATGTCCCTTCAAACCAATCCCACCTCACCCTCATGTCCAAAAACCAATTGGGTAACCTTCAGATTCACACAAAAAA

>RsbHLH033

TGTGTCGATATATTTTATTTTTCAAACGGTAGGAGAATACAATTATAAGAACTACATGAAACTAGAAACACCTCCACTATCACATAAACCTAGCCCAACTACTAGCTAGCTCCATTGGGCGAGAATTAATCCCAAATAACCTAACTACAAGTCCCATCAAGGGTAGAAATAATCCCAACAAATACAAAAGGAAAAAACCTATACGCAGGCGTGAGAGATTTGAACTCCTTATCTCCTAATAAGATCTAAGAATCATGAGTAGCGGCCTGGTTAATTGAACTGATCCATACCATGTGCATCATTTACAGTAAGGGTGTATTGACAAGCAATACATTATATAAACTGAAAGATCTAACAAGAAAATTTATTTCTACATCCCCTAATTGATGCAAACAGTTGAATGTATGAAACAGCCCTTGACATGCACACTTTTTACAAATTTCAAGGACTATTTTATAAATTCACCCTCTAAAAAACAGGGGGAAAAAAAAGTGTTTTTATCAATTAAGGAAATGTAAAAATTAATTTCTTATAACAATATGATGGGACTTCGGGTTGCTACTGTGACCTCTGAGCCGTCGGATCGTGTATCCGACGGCTCGGATTTCGTCTCGGCAACTAACGATCGAGACCCGTTCATTGCTAAAATGAGATCTCAGCCATCGGATGCACTATCCGACGGCTCGAAAGTGAGCAACACGGTATTGCGCACACCACTGCACAACACTATCCCAACTCAATACGAAGTAGTACAGTAAATTGGGTCAACACGAGCCAGTAAGATGCTGGCTGTGTTATACTCCCCGCAAAATCTGGAATGGGAAGATTTGCGAACGCGACATTATTGTGATTTGTGGGTAGTTGTCTACGCTCCAAAATGACAATAAAAAACGGCGAGAAGGATTTGGGTGAGTGCGTTTCCATTTCCAACCTTTGAAACGAATATTCAACGGCTAGCAGTGGGGGCAACAATAAAAAAGAATTTCCCAATTAGTCAGATGTTGATAATAGAAAACAGAGAGAGAAACTTTTTTTTTTTTATGAGCCGATGCCCGGGTCAATTTACGCAAACCTCAATTAATTCTCGAATTCTGAAATTTACGACCGGACAAATCCTCCAACAGTCACAAAATTTGAAATGTTTGAATCCGTGAAATTCATACATGGTTCAGACAACCTCATAGAGTACAAACGCTTGTTAACTTTTCCATGTCCAGTTTACCGACCATTGGAGTTTAGAAAGAAAATGATTGTTGCATCTCGTCAGATTGGGTTTAGAGAGAAGACTATCGATGATTGTTGCATCTCGTCAGATCAAAGGGCACCCACTTGGAATTCTTTATACCAAATTGTGAATTATTAACGATCTTTCAAAATGTCATATGGCCAGTTCACATCAACCCACCCGTGAAGAGAGAGAGAGAAAGGGATACTTGTTTTTGTCTTATTTGAGAGAGAGAGAGAGAGAGAGAGAGAGAGAGAGAGAGAGAGGCAACCCAG

>RsbHLH034

TTGGTCTAATAAATATATTTATTATATAAAATCAATCTCACACACACACACACACACACATATATATATATATATATAAACTGTTTTTATAGGTTTAATTATGTTTTTATGGGTTCTTTTTTTTAGGTAAAATGACGGCCAATGACGTGTTTTGATAATTAACACACACCAATGACATTTTGTTCATTAACAAATATTCTTAGCATGTCCTTTAAAAGTATTAATTATCAAAACACGTCCTGAGCCATCATTTTCCCTTTTTTTATGTATTGGACTCAAAATTGATCCTTTTTTTATTGATCATTTATTTACTTAATAACTTTTATATCTTTTTTCACTTTTATTTATTTAATATTATTTAAGTATTCCAATTTTCAATCCGATTAAATCCGTGCAAAGATCTTATTTTTTTATTATTTTATCTTTAATGGTCATAATGAATTTTTAACTCGAAAGCCTTTCAACAAGCACTCTAAGGCTACTTTTTTTTGGTAACCGAGGAACAACCCTGCAATTGAGTCACTATTGAACCCGACTGCGCAATCTAAACCTCAGGGGAGACTAGCACAGCAACATACCGCCATGGCCCCCCATTTAAATCATTGTTTATCTCTAGGAGGAATCGAACCCTGCTATGTTGCAAGTGCAGGTTCGTCCTTATTTAGTTTCATGTATCTCTTAGGTGCTCATTAAAAAGTCTTAGAGCCAAAAATTTATTACGACAATTTAAGATGAAATGATAAGAAAAATAAGATTTTCACACTAATTCAAATGAATTAAAAATTGAAACACTTATTTTTTTTACTTTTTATATGTATTTTTAAATTATTTAATATTATATACTCCAAACTTTACTCTCTCTATCTCTCTTTATGAAGGTGGGCATGCAATTTTGGCTCGAAAGCCTTTCAACAAGTACCCTAAAGCTACTTTTTTTTGGTAATCAAAGAACAACCTTGCAATTGAGTCACTTTTGAACCCGACTGCGCAATCTAAACCTTGGGGGGGACTAGACATCAACCCACCGCCATAGCCCCCATTTAAATCATTGTTTATCTCCGAGAGAAATCGAACCCTGCTATGTTACAAGTGAAGATTCGTCTTATTTAGTTTCATATATCTCTTAGGGTGCTCATTGAAAAGTCTTAGAGTCAAAAATTCATTACAACCATTTAGGATGAAATGATAAGAAAAATGAGATTTTCGCACCGATTCAAATGAATTAGAAATTAGAACACTTATTTTTTTTACTCTTTATATGTATTTTTAAATTATTTAATATCATATACTCCAAACTTTACTCTCTCTATCTCTCTCTACGAAGGCGGGCGTGCAATTTCGGAGCAGTAGGTGGATTTGGTAACTTCACTCTCTCTCACTCTCTCTCTCTCTCTTCTCCAAATTCTTACTTCTCCTCCTCCTGGTTTCGTTTCACAATCCAATTCGAAACTCCCTCTTCCAGTCAGAAAAAGTATTTGGAAAACCAAACCAAACCCAAAA

>RsbHLH035

ATCTCATCATGTCAACCTACAGAACAATATATTTTTTCAAATGTCGGCCCCAACATACTCGCTACAATTTCTTTCAAAATAATTCTGGTAGATTAATACGCCGGTTCAATATTGACACAGAATATGAGAATTCGTCTGTCTGAATTCGTCTAGGACAACTATAAAATTCAACCCCCTCCATACTCGAAACATTGTCTACTACACAATACGAAAACTTTTTAAAAAAATTAAAAATATATAGTATATATTTTTAAGTCCTCATTTTGCCTCGTAGTTAATTTTTTTACATTATTGTTTTTTTTTGTCTATACTATTTATACTAATAAATGAGGTTTTGCACATATATTATTGGTCAATACATCAGCTCATAAAGTTATGGTAATTTCTGAACACCCCATTACAAATTCCTGGAGCCGCCATTGGGTGGAGGTGTTGGCGTGGGCCACCGATGATTATCATATCAAGTGTTCGGCTTCAATTTGATCAGGGATTTCGAGTTTCCTTGCATGTTTCAGGAGGTCGTAGAATGCTCGGGGATGTGGTGGTCGTTAGGTGGCGGTGAACGGCGGCTCAAGGGGGACGGCTAAAGTAGCTTGTGTTGTTGGTTAGGGATAGGTTTTGGACTTCTTGGGTAGGCTCATAGGTGTTTGATGTTTGTACGGGCTGTTTGGCCCACTAAATGTTTTTTGGGCTTTTTAATAGGTGTTTTTTTTTTGGAAAATAACAGCCAAGGATGTGTTTGATAATTAATATCCGCTAAAGACGTTTTTAAAATTAACAATGTCCTTAGAGGGTATTAATTATCAAAACACAACCTAAGCCGTCATTTTCCCTTTTTTTTCCATCCAATTCTTGGTTGGATTTTTCTACACTCTCCCGACTTGATGTATCATTTGTAAAAAATTTCATAATAAATTTGTTGCCTATAAAAAAATAAAAAAAAGACTGCCCCAGACAGATTGTCAAACCGAAAAAGAAGAATACCTTTAAGTTCATAACATTTTGCAAAAAAAAAAAATCATAACGTTAATCTTAAATTGATGACATTTGTCCGTAAATTCATACCATGAATTTCAATTCATTACATTTGTATGTTATATGTGTTTTTGTCTTAATGTTTTTTTTTTTTTTTATAATTTTTATATATAAAAGATGTAATGAATTCAAGACAATTCGAAAGGTACATACGGAACATGCGAACACACCGTAGCATTTCCAATAAACAAAGACATAAGTTCTCAAACCCCTCGTATGATTCAAACACGCACACATTCTCTCTCTCTCTCTCTCACACACACACGCGCGCGCTCTCAACTACTTAATTCAAAGTAGGAAGACCTCCCAAAGTTTCTACAAAGACCTGTTTCGGGGAAATTTTTGCTTTTGGTTTTTTGTGATAATTAAGCAGAGGAGAGAGAGAGAGAGAGAGAGAGAGAGAGAGAGAGAGAGAGAGAGATGAATAGAGGTCATGTGTTACAGCAGAGCTCGCCGGTGCAACAA

>RsbHLH036

TATCTGAATACAAATGTGTTCAAAATAGTTTGAGCACTTGAACTCTCATGGAACTTACGAGAATCAAACAATTTATGCATATGCATGTCTGAATTTTTGTGCATATGAAAATGCATACAGATATTTTTCCTTACATCAAATGGGTATACATTCCCTTTTTTTCACAATGAGGACAACTGCATTGCGAGAGGAGTGAGATGTATAGACGTTTTATTTTTGTGCAAGAAAAGTCTGACTACACATTTAAGCGCACAAATAGAAAGATATGTGTCCAAAATTATATGACACACATGAAAATTTTACTCTTTACAATTCCGAGGGTTGATCCAATGGTTAAGACATGAGCTTAAGAGTTTGTACCATTCTAACTAAAGTTTCAGTTTCGAAACCTCTTTTCAAGAAGGCAGTGAAATTAGTACCCAAAATTCGAAGGCCAATACTCCATTGTGGTGGGAAGAGAAGCTTTTGAGACCACCAAATCCAGCACTACAGATATGTGGCCCCCTACTCGAATTTACTATTAAGTAGTATATCTACAAGAGCTTGGGAGAAACTGTGGTTAACCCCACTTGCACCTCTGGTTAAGAGCCATATGCATTTCACAGAGTTTAAACGGGGAGCATCTACTATGTTTCTTCTTCCCTGGGTCACCTGCGAACTGCAAGCCTTTCGCCGCCTTCTACGAGTACCCAAATTCTCTCTCTATAAATACTTATAAATTTACACACATCTATACATATTGAACAACAGTTTCGTTTTCCTTGGAACTCGAAGACAGCTGGCCGTGATGATGATCACAGCTCTTGGGAAAGCTAATTAATGCATAAGGATTTGCGTATGTTGGTCGTTTCGAGGAAATATTACTCGACTTGGACTTTGATTTGGTTTCAAGAGGTGGGGTTTTTTTTTTTTGTTTTTGTTTTTGTTTTTGTTTAATTTTTGCTATCTCCCTTATGGTTGAATGATTGATTGAGAAGATGACAAAGAAATTAAGGGATTTAAAATTGGGGTTTCCTTGAGATTACTGGCTGATTTTGTTTATCCTATCTGGGTTTAAATGACTTTATCTTCTAATGATTGGTTTGCAATAGAATACATGAAATCTTTACCTGAGATGACCTCAACTTGATGATTTTCTTTAGTATCTTATAGTGCAATTAAAGAATTGGGTTTGACTGTTCATTGCTTTGTTAATTATTACTTGTGCTGCTAATTTTTTATCAGCACTGATCATGACACAAGTGAAAGATTTTTTTTCCCAGTCAAATATTTATATATAAAGGTATGTTTTTGGTATATGGGTATTTATATATGCCCTTAAACTCGGTTGAAATTGTCTTATATAGCCACTGTATGTGACTCTCTTTGATGTCACGAAACTTTGTACTTGTTTCTTCAAAGTCGGTTTGTGCTTTTGCTGATATTACAGTTGTTTGGCGGTTTTGGGTCCTTCACTGGTGAATAATACCACAAAGTACAGAAGTTAGCTGTTACTTCATC

>RsbHLH037

ATAACTCATAAAAATTTGGCGCAAAACTAAAAAATGAAAAAAAAATTCAAATATGGACAAAATTTTCAAATTTAAGTTAAGTTCAAAAGAACGCAATTGAAAATGTATATAGAACCAAATTCAAAAGTCACAACATAATATAGATCTAAAAGTCACAACATGATAAATAAACCATGTCCAGAAGTTAAAATATTACATAAACACAGTTCCAAATTTAGCAAGTTATAACCTAATACATAGACATAACAAACCACTCAACTAGGTTACATTAAACCTAACTTAACAGCCCAAGTGGTTAAAAAAAAATAGTGTCCTGTCATTCCATTCATTTTCGTACACTTAGCCCATGCATTGATTCCTAATTAGAGATGTTGAAGTTTGAGTAGTCCCACAGAGGAGAATGGGGGAATTGCTAAGTTGACATTAAAAAGGCTTATAGCAGTTAGCAATTGAACTAAGTTGGAGCAAAAGGAAAAGTACAGAGTGTAAATCGAGCCAATTAAAACTGTAGAGATTAAGTTGACACAAACACTAAACTACACGGACTATTTCAATTTTTTCCCCTAAAATATTTTGTCAGTTTCGTTCAACCCGACCAATCAAAATGTGGCCTTAAAAGTATACAGGGTACAGGAAAGGCAAAAAAGGACATTTTATTTGCTTTCTTGACTCTTTTCAACATCCTCGCGAAGTTGGACTTTGACCTATTTACTTTTCCAAGATTTTCTCGGCTGCTGCTGCTGCTGCTCTCACTGGAACTACTTTTCAAAGTTACAACTGCCTAGCCAGAACAAGTCCTTTTCCCAGTTCTACTTTTTCAAGCTCTTATTCACCAAACCCCTCTCTCTCTCTCTCCTCTTCCCTTTACTCTTTCAAGCTAGTATGCACCAAACATCTTTCTCTCTGTCCTTCACTTTTTCAAGCTCCTATTAACCAAACACAACTCTCTCTCTCTCTCTCTCTCTCTCTCTCTCTCTCTCTCTCTCTCTCCCACATAGTTGGTGCCTATATGTCTATATCATGAGCACTTAAGGAGAGGGAGAAGGGTTGTAGAGAGATCTTGCCTTATGCCTACATAAATCTATGCATATGCATGGACTCTATCTTCCAACTTGATGATGGAGGCCGTGCCACCTTTCTCCAACACATGCTCCGCTCTTTTGCCTCCACTTATATCTGCCTCTGGTCTTACTTACCCCAACCATCCAAGTAAGATATCTCTTTCTCCTTCTCTCTCTCTCTCTCTCTCTCTCTCTCTCTCAATGCCAATGGCTCCTTTGTCTCCAAAATTCACGTCCATACACTCGTCATTATGCATTTCAGCCACACGAATACGATATCTTGAGATTCGTGTACGTATTTCAAGATTCTTTTCGGTGGTGTGACTGAAAGTGCATTGAGACAAGTGAATACGGATTACTGGGTCTCTCTATTCTATGTATATACAAAACCAAATCTTTCACTTATATATATGTATATAGTTGTGTATATATATGTTTG

>RsbHLH038

TTGAGCTTCCACTGTAAGTTTCTTGGAATCTTTAATTCTTCTCATCTTTTCTCTTGGTTTTGGACATTTTCCTTCGGAGATTTCGGGGTTTTTCATTTTTTTCTCTATTTCTTTGTTTTTCTTCCCGCGTTTGGTTCGTTTCTTCTCGGACCTTGCTGGGTTTTTGCCCATGTGTTTGATTGCCCAAAAAATAACTTCTAGGAACATTTGTAATGCTTAGTTTCTTGGACCGGGTTCAAGCAGTTCAGGCAGAGGTATGTAAAGAAGGAAGGCTAAATACCCCAATTCAACCGTTGTATGACATGTGATTTAGGTAATCGTCATGTGGAGTTAATCAAACCACTATATTTGATGCTTTAATTTACTTTGTTTCATTATTCTCTCGGTTTTTCTGTCCTTTACGTATTAGTAGTTTTTTTTTTTTGGGAAAAGTCTGCTCACTCTGCCCTTCTTACAACCAAACTTACAACCGATTTGTCGCAAGAAATTTTGATTCTTGTACTTTCCTAGATGCTATGGTGTGCAACGATATGAGTATAGTAGGTTTGTGTGAACAAATGGCCCTTACAGAGCTCAATGGAGGAAAATGATTCATGTAGCCGGCCCCAAGTGATTGGGACTTAAGGCTCGGTTTGGTTTGGTACTTTCCTAGTGTATGATTTTCCATCCGTATCTTGAAAACTAAGAATTGCTTCACTTCTTTTTGGGTCTTTTTCTCTTTATTGTGAGATCTCTAACTAGGGTTTTTTTCCTCATCCTGTGTTGTACACTATTTTCCTTTATATTTTCACATTCTTTTTGATTGCTAATAATCCATGGCTACTATTTGAAAGTTTCTTTTCTTTGTTTATGGGTGCTATCCTTCATAAATCCTCCAACCAAGTTCAGGGTTGGACTATATTTATACTGATATCTGTAGAATGGGGTGCTACTCCAAATTTATTCACAACCCTGGAATTCTAATGCTTCTTTTAACCACCTACTAATTCATTATTTGATGCTTTTATTCATCTTTGATCTTACTTAACAGCATTCATAGATACCAATGGCTGTTTTTTCCCTACTTTTGCAGGTAACTAGGATATATTGCACAGAAGTAGAGCGTCATGTGGTGGTTGGGTCTTTCAATTAATGTGCTTCGGAAAGCTGTCTCACTGAATTTGTATTGCAGGGTTATCTTATAAATTCCTACCCTTCCTTTTAACCCTGCTTTTTGGTGACTCTAATTTTCGCAATTCCATGGCATCCAATCACTTTTGTTTCAATCCTTGCTATATCCATAGGCTTAGTTGAGAGAGATTAAAGGCCAAAACAACTTATAACTACCCAATCTCTTATTTTAGATTCTGTTCCCCTTGATTATCGCATTTCTCATAATCTCATTTCCATTTGAATTGATATCTCTACTTTTCGTCTATTGTGCGAGTATATCTTGTCCTTCGATCTGAGTTGTAATAGAGGTAGAAGGAAAGATCTTGAATTGTAATAGAGGTAGAAGTT

>RsbHLH039

ACAAATTAATTGAGCATCCAACAGCTCAGGCCCTTTCATCAGTAATATCAGAGCACAAAACCAGCAAAAGCTGGCTGAAACGGTCAGTTGAAGCGCGGATCAACGATGCACAAAGAGAGGTTGACGAAATCCCAGAAACCATTGAAGATTTGGAAAAATATGCGGAGGACACCGTATCAACGATCTTGTACATGACACTTCAAGCAGGGGGTATCAAATCCACCGCAGCAGATCATGCAGCTTCGCATATTGGAAAGGCTAGTGGCCTTCTTTTGCTGCTTAAGTCGTTGCCATACCACGCTAGTCGCAATCGTCTGTGTTCTTATATTCCCTCTAATGTAGCAGCCAACCATGGACTTTTGGTTAACCAGGAGGGTCCACTGGAGATTCGCATGGACTCCCGTGAGGGAATGTGTGATGCCATTTTTGAGATGTCATCTGCCGCTAATGCACATCTTCAGAAGGCCAGGGAATTAGCTGCGACAGTGCCTAAGGAAGCTCGGGCAGTGCTTTTACCTGCTGTGCCCGCCCAGGTTCTATTGGATTCCCTGAATCGTGTGCAGTTTGATGTGTTTGATCCAAGATTGGCAACAGGGATTCTGGGTATTCCTCCTTTGTGGTACCAGTTGAAACTGAAATGGCATTCCTTGAGGGGAAAGTACTGAAAGATAAGTTATATTGTTGCCTTTGCCAATTGAATGGCAAGTTTTTCATCTTGTGGGAAGCTGGGTTGAGTTTCGATCGAATTTTTACTTGTTCAAAATTAGGGTATTTTCCCTAATGTCCCAATGGCAATTTATACACAACTGTTTTGTTTCTTTTTTCATCCGTCGTACTACACTTCACTTTTAGGCACTGTAGTGCCAGGTTTTTTATGCAGGTCCAAATTTTGAGATGCATTCTGGGTCAGTGGAATTTCCTTCAAACAGAGGGCCTTGTGCTTAGGTTGAACGATCTCGATATGTGGTGTCGTTACTCCATATGTTAGGTTATGAGATTAAAGAGGCTATCCATCATTTGGTAAGTTTCTCTAAGTATGCATGTGGTTATTAAGAAGTTTTGAGTACAGTTCAAATCTAGAAGCTCTGTGCAAAGGAAATGCTGCTCTTTACATTGCCCGGGCATTGCTTGCCATCTTGAGGCATTTCTTTCCGGTTTTTCCCCTGGCTCTTTTGTTTGTTACCTTTTTGATTGAGGGCAAATCCAGTCTTTCTGCGCAGTTTACTGTTTCAGCCAAGTATCAGGGCTGGGAACGGGCATATATTAGCTCTCTGTCATCTGTGGAGTTGGAGAGTCCACGTTTACTCCCACTTTCTTATTTTCTGTGTCTATTATTGGGGAAGCAGTTACGAGTGTCTGTACTTCATAGGTTGGTGGCTTCTTGTCCTGACACTTTGAATGATTTAGCCGATTGAGGTATAAGATGATTTTCTTCGGAGTCCCTGGTGCCTGGTGGGGATGTAGCAACAGAGGTTCCTTTGGAGGCAGCATTTTTTTTAT

>RsbHLH040

TTTCTTCACAATCATGCAAAGTGACTCGTAAAATGCACACACAATTCGACAGACAACATTCCCTATCGCTCATACGTGTGGGCTTCCGCCAATTGGCTCATGTGAGTGCGTGTGAATATTCTGTATTTAACTGGACTTTTGCTTTTTTTTGGGATCAAATTCACACAACTTTTCTTCCTTAAAATCACCGAAAGAATCTTGTCTCTCAAATAACTACACATTAAAAGAAGAGTTTGATCAAACACTAAGCGCACTTCAATCAATGTTTATGCATAAAATTCTACCAATTAACTAAACTAAAGAGATTGGCCGTGAAAGGTGTAGAGATAATCTTAACCAAAAGCTACCTTAAAAGAAAAAGAGAATGCAACAGGAATTTATCTTTCGATGAATTGAGTGGTATCCTAGGGGAAAAAATAAAAATAAAGGCTCTTGTTATCGTACAGTTGAAATCTAGGAACAAAATAAATTAGTCCTTGTCCTTTGTAACTGGACTCCAACACATAGTTGAAATCTAGGAACAAAAATCTACTTTGATTTCAGGGGAGGTTCCTATATTTCCTTGTGGGCCATCATGTATTTGGACGAGTAGATGCAAGTTTTTAAATGACAGTACCGGGATATGGAACGGTAACCGTGGTTTTTTAATAGGATTGAGAGTTGTATAAAATGTTATGTAGCAAGAATCGGTTGTAGCGCTCGTAAATTTGTAACAATAATTGTGACAAAAAAAAAAAAATTTCAATGCCTAAATCACTTGTAGTGGCTGAGTATAAGTATTGGATGTGAACGGGTCTGTGCATTGGAACGGGTCTGTGAACGGGTCTGTGCGTGCAACTTTTCATCTCAATTGTTTATCAAGGTGTAACAGCATCGAAATGCATGCGCAAAACTAATGTACCGATTGTATAATGCTACAACTTATGTCTGTCCCCTTTCTGGGGAAGGGGATACGGTATTCTCATGTACTAACATTAACCACTCAGATGTATAACATTAACCACTAGAAGGCGTCAGAAAAATAAATCAAAACAGAATACTATCGGCCATAAAATATTATGAATTTGGCTCTCAAAGAAAAAAAATTCTAAACCTCAAAAAAAAATGAAGTTGCAAACAGACCCCAAATGACACTTTCTCACATTTTTCATATGAAAAATGAGAGTTCGATTTTCACCAGGAGCATGAGTGCTTGATGTGGTGGTTCATATCTATTGGTTGGCCCCACCTATATGCCGCTTTGCTATTTAAGAGTATGATAGACATTTATCGGGTGAAAAAAAAACCTCAGATTCAAACCCTAAAGACCTAGCCTAGGTGGACAACCATATCTTACATAGATTTTCTGCTGACCCCATATGACTACATCAACTTCCTTCATTTAATACCATGGTCACCTCAACTAACCTGAACCCCCTATAAATATGTCCCTTCAAACCAATCCCACCTCACCCTCATGTCCAAAAACCAATTGGGTAACTTTCAGATTCACATAAAAAA

>RsbHLH041

TTTTCAAGAAAAAATTCAAATTTTTTTCACAAGTTTAAAGAACTCGTCAAGATCTAGTAAGTTCAATTTTTTTCCAACTTTTCTTACGAAAATAGTATTATTTTTTTGTCTCCGTTTTGCAGACCGGACAAAGATTATGGGACAGAGGGAGTACTTATTAAGAAAACAAATATCATTACACCTTTTATATTTTACCTTCACTTTCTCTATTTACTATGTATGAAGATATCATCATTACACTTCTACTCACTAACTTTTTAAAATTGCATCTACTTTCAGAGACAAAATAGAAATGTATCATTTTTTATCTATTAATTTTATAAAATGGACACTTATTAAGTGACAATCCAAAATAAAATACTGGACTAAAAAATGGAAGGAGGGAGTAAAATAATTTACCAGAGCAGTTGGAGAGTAGTTAGTCTGTCTTTTAAAAATCATGTCACCTGTTAGAGCAGGGCAGGTAAGCAAAGGAGTAGGCTTTCTCTCTCTTCACCTCTTCATATGCCAGTCTCTCTCTCTCTCTCTCTCCCCTATCGGTAACCAATCTTTGCTCTGTAAAGCAGGAACTTGATGCTTCCATCACAATTTTCTGCAAGTACCCAGTTGGTGAATTTGGTCTCTCATTTGTCTAATTCAAGCAACCGTTTGGTGCTCTCTAGGGTTTCTATCAAACACCCAATTTAGGTCCACTGTTTAATTAAAGCCTCTGCCCCTTCCAGTCTCTGCAATTCAAGTAGAATTCTGATGCTTTTATTGCCCTTTTCGGGTACCCATCTGAGAACCTTTTAAAGGGTTCTATCCAACTACCCTCTTTTGGGTTCTTGTGCAGCTGTTGATTCATATCTTCTACTTCCACCTCCGGAAAATTTCAATCTCAAGGTTTTCTTTAGAATTATTCCCATTTTGAAGCTTGTTTTTAAAATCAAAAAATTGGGTGGAGGATGTGAAAAAGATATAAAATTTATTCTACCCATTAAACAAAAGAGTGTGAAAAAGGGAATCTGCTTTCTTTTTGGTTAGTCTTGTGAGCTGTTACTTAGCTATTGTCATCTAATTTTTTTCTTTTTTTTTTTTGGTCCACTTTATGCTCTGAAAGGTTCACACTTTTTCTTGTCTCTGGGAAATTACTTTTTTCCCATCAGGGCAGGCTTGATCCTAGGGTTCTTTGAAAGCAGATATAACTAGCTGGTGCTGTGTAGATTTTATGCCTGGTGCATTGGATTTCTTCTTCTTTTTCTATGTGTTATATTGGATGCTGCAATTGAGAGGATTCCTTTTGCTTTAGCCCCCTTCTCACTTCTATAAAGCTGCAAAGGCTATTTGTTCCTTGTTTAGTGTTGCAAGCTATAACTCACCTAATTTTAGTCAAACCCCCTTTTGCCTTTATATTTATATAGTTCCTTGTGGTTTGGGGGAACTTTTGAATTGGGGGTTTCTTTTCAAAGGGTTAAGGGCTTCCCCATAGGATTTGGAACTTCCTTTGTTTACTTTGAGGAA

>RsbHLH042

TTCATTTTGAGTTAACCCTGACATGAGGCCAGTGCAATGCCACAGTTTTGATTCTGAAGTGGTCATAGGGCCTGTTTGGCCACTTCATTTTTCCATTTCGAGGTTTGTGTTTTTAGCTTTTGGGTGTTTGATAGCCAAACCCAGAAAGTATTTTACCCGAGTGCAATTTCAGAAATGAACTAGAGAATGCATTATTCAAAAATCACCATTAGATGAATTTTTCAGATTTTGATTATACTTCTTGCCAGGGAGTGCAATTTTGTTCACCCTCATTAAAAGATGATGTGGATTCTATCATAAAGTGCTTACCATGCAATATATCTTTTGGTAAGTATGATATCCACCTTGTGGTGGAATTCACACCCTTTCAATTACTAAAAATGAGAGTAATGTTCACCCTCTTAAGTGAAGAATGAACAAAACTAAAATAGTACTCCTGAAAGATGTGGGATATGACCAAAATAGTACTCCTGCTGCTGTTAGGAGACTTGGGACAACAAAGACTGGCTTTGCGTTCCAAAACCCAACACACACACACACACACACACACACTCTCTCTCTCTCTCTCTCTCTCTCTCTCTCTCTCTCTCTCTCTCTCTCTCTCAGACTGGCTTTGCATTCCAAAACCCAACACGCACACTCTCTCTCTCTCTCTCTCTCTCTCTCTCTCTCTCTCTCTCTGTGTGTGTGTGTGATATTTAGGAGTAATAAGTTTTTACTCCGTTTATTATTTTCTTCTTTTATGTCTCTTTCCAATTATTATTCATAAACTTCCCTCAAAACTTTTACCAAAATAACCTCTGTTGGTTTGGTGAAGATTTTGTTTGGATCAAGGATTTGTTGATGCACTAAGAAGGAAAATTAAGGATATTGGACAAATAAATTTAACCGATTACCAAGAGAATGGAACTACATTCAACTTTTCCTATGTTTAAATTCTACCTTCAGTTATTATTGGTCAATGCATGCACTTTTTTTAAAGGATTTTTTTTTTTAATTTCTAAGAATTTTAGTAATTTTTAACGTGTCCTTTTCATGCACCCACAAATTCCCTTTCTTTCTTTTTTCCATAAACTAAAATCTCCTTTTAAACTGTATCTTTGACACCCAAAAAAAATAAAAAATTTACTTTGACAAAGAGAACCGCTTTTGTAACTGCCTCAGTTTCTTCGACACGGTTTCTTTCTCCTATTTTCTCTCAACCGTTTTTACCAAGGGAAAAAAACCTAACACAGAAAAAGAAAAAGAAAAAGAAAACAAGAGATGCTGTATAACGGTATCAGACTCTCTCTCTCTCTCTCTCTCTCTCTCTCTCTCTCTCCTTGTCTAATTGCTTAATTTCTTAGGTAGCTTTTATAATTCTCTCCCTCTCCCTCTCGCCTTGTCTAATTGCATAATTTCTTAGGTAGCTGTTATAATTCCAACTGTGGCTCACTCCAGCATCTCAATTACAAAGAGACGGATGATATTTTCCCACTTCAAGCACAATCAGAGCTAGCC

>RsbHLH043

GTAAAAAATCGATAAAGCCCTTATTGATATTCGAATGAGCTGTTTTTTATAGGGATCCGTTAAAAATATTTGAAACAAATTGAGTGACTCCGATCATTTATATGGGACCCCGAGATGGGTCTCACTCAACGCCATGTACAAATGGTGTACCCATAGAAGGACTCACAAAGAAACACTAATGTTAGGGTTACTGAAATAGAGGATACCAAAAACATACAGACTGTGTAGAGCCAACTTTGGGATCCACACGAATAATTTGAACCACTCATTAGTGTTAAAATAATTTTTTGAGTAGCCCATGCAAACAAACAGCTCAATTGGTTATCTAATCTTTCAAGTTTTCATTCAGAATTTGATAACCAAATGATCGGATCAGTTTGCGCACACCTTGATTAAATCTAAAGCTTTGAAGAAAACCTCGTAAAATTTAAACATGTGATCTTATAAAGAACAAACACTTATTCACTTTCTCGTTTAGTAAATGATTTCTACAAAAGTTTTATAAATGATTTCTACACTTCGGGTTAATACTCCAAAAGTTTAGTAAATGATTTCTACACTTTTTTTTTGGTTATAATTCACACTATCATTTTTGTTTTTATCCTCATGAAATTACAATATTTCTTTTGAATGTGTTAAAAAAAGAGCAACATTATAGTTTCGTGTGGATAAAGACAAAAAAGAAAAAAGAAAAAAGAAACGTAGATTAAAAAAAGGGTAGTTTGACATCAACTCACAAAATATTCACTTTTTGTGAATACAAAGAACCTGACTTGATCTATGAGATGAGACAGAAACGTGGTTCAACATTTTCAGAGTTGCAAATTACTAAAATAATACTTATATCAGATTTCGGTGAAAAAGACATGAAAATCACCTAGACAGAGATAGACAGGAGAAGAAAAAAGCAGATTCGATTCGAGTCTAAGGCTCTCTAGGCACTAATTAACACCCAATAATGTAATGCGTTCTTAATCTTTACTTATCTCCCTGCTTTTTATCCCCACAAGATTACAATTCATAGCTTAGGATGCTTGTACAAACTTATGCAAATCATGATTAAATCTTGAATGACCATCTCACCGTCCATTAGGAGTCTAATCTAATAATTTAGGCCTGAGCAAAGCCTTGTATGAATAAGCTCTAGAAGAATTGGTATGTTTCGAACGTCAAGTCTACAAGGGAGCAAACTTTTACGTCGTATTTTTCCTTTGCAAGAATTTCTGGGGCCACCTCACCCCGACTTAAGTGAAAAGGTTGTGTGAGGGACGGTAGCGGGTAAAGATTAGTCTTGAGACGCTCTGTATTAGAAAGAAAAAAAAAAAATTACGGGTTTTCTCCCTATATAATATTTCCACTTGGTCCAGCTACACTTAAACAAGGCAAGTCCTTAGATGAGAGAGAGAGAGAGAGAGAGAGAGAGAGAGAGGGAGGAGAGAGATGGAATATGATGGGCATGGTTTCTTGGAGGAGTTTGTAGCAACAACCATACCAATTGAA

>RsbHLH044

CAGGGGCACTAAACAGTGGCAGAGCCAAGATTTTTACTCCAGGGGACTGGTTTCATAGATATATTTTTATCGGAACGTATGCTTATTGTATAAAATAAATTTTTTATTTTGTAATACATTACATTTGTACATTGAAATAATTTTAGCCTAATGTAATTAATATATATCAATCATTTTGTTTTTTTTCCTTTATGCTACTCCAACTGTCACATGTTTTTTTTTTGATAACTTGTGTATTTTATAACCAACAAACTCAAAAACGAAGGGGAGTTAAAACCCATATACAAGAACCTATACAAAGGAAATCCTCAAACATTCTACCAAATCTACAGATTTGACGTCCCAAGAACACTTCCTGAAATATTCCAGCGTTGGCAAAGCCTTTGATTTTCGAGAGAGTTGGAAAATGCTCCCAAGTGCAAACACGAAACCTCACACTATCTTCAATACTTGCCATTACAGAGTCAGCTCCTTTTTATGTTTCCCTCCAAAAAAAACTCAAGAATTCCTTTCCAGCAAGAGATGGTAAACCCCAGCTGCCAGGACCACTTTTAACAAGAAGGATCTGAAACTTTTACCTCTGCAATATATGATCGATGGCCCACATAAGTTCACGATCCCATACTTATGGAGTTCTCTGAATCTGGAATCGATGGAGAATTATCTGCCAGATACTGCTTGAGTAGCTACAATTACTGCTCGAGCAACTGTCACGTGCTTATTTTTCATTTATGAAAGAAATTGAAAAATTAGAATGTAAAATTAGAATAGGTTTCTTATCAAATCCAAATCAAAATAATTAAGATTATAAATAGTTATACACGAATAATTATCAAATCAAATTATACAAAATAAAATTTTTAAACTCGAGAGCTCGGGAACCAGGATCGGGAGGTCCGGGCCCAAGCCCAAGCCCAACATAACTCCGCCACTTAGGCTCCGTTCGGCTAAATAAGCCAATTGGTTTATTTTTTTTTTGTCCTTATTCAATTTTTTTCGTATTTGTTAATTTTTCGTCAATATTTTGGAAATTATTGCATCGTCATGATAAGATAAATTTAACAAGTAGAAAATTATGATTGAACTCTAATTTTTTTGAATAAAGACAAAAATAAGCTAATAAGCCAATTTTTTCATCTTTATTAAAAAAAATTGGATTTCGATTATAATTTTTTACTTTTTAGATTACTCTCATCATGATGATGCAATAATTCTTAAAAAATTGATAAAAAATTAAAAAATACTAAAAAAATTTAAATAAAGACAAAAAAATAAAATAGGGAAATAATTTTACCCTGTAAGTATTCCGAAAACAATCAACCGGTTGCCCAATGGTTGTTCAATTAATGGAAGCGGGGCTTAGACGCAAAAGCGTTTACTTTCGCGGACAAGGTGATAGAATGATATGGACGCTGTCACTCTCAACGCTTCCGTCTCTTCCATTCCCTTCTCAATTATAATCCATTACCCACTCTCTGACCATAAACATTTTCACAAAAA

>RsbHLH045

GATTTCAGTAAGAATTGATCAAGCACTTACCGATATCTAATTGAACTAAATTTTCAATTAAACTAATAGCAGGTCTCGTACCATCCATTCTTAATCTTGCCAAAACCGTCATGGCCTCAAAATGAGCATTTCGGACTTGATGGGCCCCACATGAGCGGTCGATACATTTTGGAGATGTATGTAGCCGTAGTATTGTTCTTGGACATCTACTCCTGTTTAATTAAGGTTGTAGGTTCAAATAAGATGACCAAGGGTCAGCAAGCAAGGTACATGAGGAGGCTTCCCAAATTAAATTAAAAGCAATCAAAAACAACAATTTGGTATATATACAAAGAACAAAGTAATATAGGAACAAAGTAATTTATTTGAGTGTGGTCCGCACCACATCAAAATTGAATATGTACTAGTAATAATTATCATGATATTTTTATGGATAAAGCCAAAATATAAAATTGGGTTTTGCAATCTACCAACCAAGTGTGATTACATTTATTTAGGCAAAAAGAATCCAGCATCCCCACAATCTAAAAAGGAACTTCAAAAAGCGGTCCGTGCAATCGGAAATTGGTTTTAATGGTTTGAATTTGTTAACAAATTCTTACAGGTACAAATGATCATATGATCTTACGGTAAGAATCTTTCACAAACCTAACCCATTAATTGTCCAATTGAACAGTCAAGATGCAGCTCCTTCCGTCTCTATATTTAATACTTAATAGTAGTCTATTCCGTCCTCGATAGAAGCTCCATCCATCCTTATTTAATAGTCTCCCGTTTTATTCTAGTCGGATAAAATTACTTAGTTATATTTTTAAACAACGCGCTTCTAATAAGGGTTCCGTTATTTTCGTTAAATGTGGACGTCGCTGGTGGCCGTTGGATCATGATTTCAATGGTTCAGATTTTAATGAAATTTTTTCGAAGAAAGTTAATTATGACCAGCCATAAACCATCAGCAAATCCGGATCATTGAAATCATAATTCAACAGCTTACAACAAAAGTCCGCAAATAACAAAAAATAAGGGTGCCCTAAGGCTCCTATATTTTTAAATTAGTAATGAATTTTATATCTACTATGAATCTTATTTATATGATAGATCTCAATTAGTTCTGTAAAATATTCAAAATTATATAAACTTTATAAATTATGAGATATAATCAATTGAAAAGTAATTCGAATTCTAACAAAACAGACTATCAAATGGGGACGGAGAAAGTAATCAAGATCAATCAGGGGACAAATACCACACGGAAGAAGACAAGAATAAGAGGAATAAAAAGATAACTATCATGGTCCCGTGGAAGAAGAAAATCCATATAGTATTTATCTATTTTAATTTACTCGGCTAACCAAGGGACAGGATTTGATATCAATAAGACCTTTCATTTGAGTAATCAAATCAGTACATGACTATACCACCCCTGGAAAAAAGTCAACGAAATTCTGGTTCTTACTTTGGTATAAATATCGAGATGCAGCGAATCGTTTTCATCACCGT

>RsbHLH046

AAAAATTTCCAACTTTTTGTTTTTGGTTGTTTCGTCATCCGTGTTTAAAAAAGTTAAAAAATTATATAAAAACGAAAAAAATATGCAAATTTAGGCTTCATTTGTTTGGGCGTAAAATGTTTTATCTATTTTCCGGTGTTCGGTTGTGTAAAAAATGAGAAAAATATTTTTTATGTAAAATATTTTCTATTAATTGGATGAAAATGACTTACCCTTAAATCTTCCATAAGTTATTTTCCAAAATAAACCCGCTACCTTCTACGGCAATTCTCATTGCTCAGTTTCCTCAATCGTCAATTCTGACCCACGTTCAATTTCAATATTCTTGAATCTCTTCACCGTCTAAGCCTTCCTATAGATCTCTCTACACTATTTTATTGATTTTCTGAAAATATTTTCAAGCGCCAACCAAATATCTGAAAATAAAATTTTGAAGTTTGTTTTCTGAAAAAATATTTTACATCAAAACAAACGGAGCCTTATAAAACTTTTTTTATCTTAAAAATTAATCTCAAAACTGACAACCAAACAAAATGAATAATAATACTCCCTCCGTCCCTTTTTTAGAGTCCTGTATTCCATTTTGGGTTGTCCCTTAATAAGTGTCTATTTTTGTAAAGTTAGTGGGTAAAAGTTGGTGAATTTTCTATTTTGCCCCTAAAAGTAGATTCCATTTTAAAAAGTTAATGAGTAAAAATGTAATGATGATGTTTTAATAGAGGGTAAGGATGGAAAGTGGAGGTAAAAGTTGATGTGAAAGGTGAAATGATGATATCTTTTTAATAAATTGGAATTACGAAATAGGACTCTTAAAAAGGGACGGAGAGAGTAATGAATAACAATAATAATAACAACGGAGATACCCACGAGTTGAGCGTGGGTCCACAATCGCCACAAAAGGAAAGGTGGGGAGTTTTGGATTGGGAAATTCTGTAGTGCAAGATCTTGCAAAGCATCTTATAGGGCACACAGGGGTCATCGATCTTAACAATGAATGGTTGAGATGTGGTTTTTACTTTTCCGGAGAAGAATTTAATTTCTCCCAGAAAAATTTAATTTAAATCTGGACCGTTACAAACACTCACGGACGGGTGTGACGTGGCCTACACGTCCTATAGTGTAGTGCAGGGTGCGGGTTTTCGGATCCGGGGAGTTCCTACTTACTTCCTAGAGTTCGAAGGTGATGCCCAACTAAAGGAAGATAAAGGGCTACTTGGTTTGGACTTATCCTTCAAAATCTCTCTCTCTCTCTCTCTCTCTCTCTCTCTCTCTCTCTCTCTCATATCGGTTTCTTTAGAGGAAACAGTTCTGAGGCCGTGAGCTCCCAAGCTTTCCTGCCCTCTCAACCCTCTCCACAAAAAAGATAATTAATTTGTCTACATGTGAAACAAAAAAAAATGAAGAGAAGATCAGAGTGAAATATACAAAAGAGATAAGGTGCATGTGGGATCAAAGGAGGGCATATCAAAGCATCGATCGGTTAGGCCCCCCAAAACCCAT

>RsbHLH047

AAAAAGGGAAAATTTTAAAATAGCACCTGAACTTTGCACCAAATGTCACAGAGACACCTGAAAAACCCACTTGATTTTGTATACAGTGGCTTTGTTATTCTGAATGCAATACGTTTGTGCTTTCTAAAAAATACAGCAATATTTCTCAATCTTTTTATTGATAGGTTAACAACCACATAACAGAACACAGTTTATTATAAACCCAAGGCTGCTTGTTTTTTTAGTGGAAGAGTAAGAATTGCATATATAGGAGATAACAATTTATAAGGGATACAACATGATACAACGAACTATATAGGTGTTGGTGTGTCGGGGTTGTTTCTTTTCTTTTGTTGGCTTCTTGCCAACGTCTCTTTTGGGTCTCGGTTAGCTTTTGTTTGTCCTTATTTCTTTTAATCTTTGTTCTCTTTTTAGAGATTAATATAATTTCTATTGCCAACAAAAAAAAAAAAAAAAACTACGAAGACAAAATATGGAAATCTGCATTGATGCCGCTCCCCAAAAGTGCCTCCCCTGCGGCTGCCGTAGTAGGAGTAGAAGAAGAGGACATCATGGGAGAATCACATCGAAGACAGGTGATGACAAACAAACACATCCAAAACCATTACAAGAAACATTAAACTCTGACAAAATAAACAGGAATTTGTTTTGAAAAACCTTTGAAAATGAGCCATCATCATCGAAAGGGTTAAAATATATACTAGTGTCAAAAGGAGGTTGGTAATGCCCTGATTGGCTCAACCACAAGGGGCAAGGGGCCACCTAATTTAAGAAAGGCACGACTTATCAAAAAAAAAAAAAAAGAGAAGGCATGAAGCCTATCAGGTAATGCCAAAAAAAAAATTTTTTGATACCAAAATTTTGTGCATGAGAGTTTTGTACTCAGGTGCAAACCTTGTGATACGAAAGAATTTTGGCAATAAAACAAAAACAAAAAATTACATTATCGCTTCTTAATTATCGCAATATCGCTGGCAAAACAATGGACAATAAGTACATTGGTCTAATGACCTAAGAGCATCTCCAATCCTTACCCATTTTATTATCCATACCCAAAATTTGAGTAAAAAGCTTAAAACCAATCTTCAATGTTATACCCATTTTTAGTTCTACCCATATTCTCACCAAAAATTTGAGGAGACTCAAACCCATACCCATTTTTTCATAGCCTATTTTCACTCTCATTAATTACAAATTTGCCACTCATTAATCACAAGTATGCATGTCATTCAAGAATATATTTGGGTACAAATATGGGTTTAACATTGGAGATGCATTACCCAAATCAAGCATATGACTCAAATTTCTACCCAAATTTGGGTGAAAAAAAAAGGGGTAAGGATTGGAGATGCTAAAAGTTGACCATGTATGGGAATCGCCAATTTAAAAATAAAAATTCCCTCTAAATATATATATGCCCAATCGGGAAAGAATTTAAACTTTTTTTTTTTTGTCGTAAATATTTAATCCCGTTGGTACCTTTTCTTTTCTACTTGCGAG

>RsbHLH048

TTGGTTAGCTCTCATTGTAAGTTCGTAGCTTTTCGATCTTTTCCTGTCTTGATATTTAGCTATGCTTATTATTTTGTCCCTTTGAGGCTGTGATGAGAAACAACGCTTTCTCCTCCATCTTCTTCTACATTTCTTACCATTTCTCACCAAATTACTCGATCCAAGGGAGGTATTGGTGAGAAACCCTACTACTTTCTATTCTGTTCTCACCAAATCATTCAATCCAAAGGGGCTGTTAGTTTTGCTTGACCCCTTCTGCTTTGTTTTGTGTTCTTTGTTTTTTCTTCTCGTTCATTGGTTTATTTTAGATCCGTATTTATTTTTGTGTTTCATCCTGGAATTTTCTCCAGCTTTCGGTTCCTAAGAAGAATTAGGCAACGATATTAGTTTCTTTTTCTTACCATTATCAGGGTTCTCAAATTACACGAGGCCCAATTTTATCGAAGCAAATTAAGTCTTCATCGGTTTCTTGTGCTGTTTTTTCTTGCTTAATAAGAGAGATCTCAGTTTGGTTGGTATTTTCACAATATATTCGGCAGAATACTTTTCACAAACTTTAAAAACTTTTTTTTTTTTTTTTTTGTGGGAATTTTCTGTAAAGAATTCAACTTTTTAGGAAAATATCAGTTTTTTTTTTAATTTTTAAATTCTGTGTAAAGAATTCAACTTTTTTGTTGATGGGATTTTTTTTGGAAGAATTCAACTTCTTTTTCCCCTGGGAATTTTGTGTAAAGAATTCAACCTTTTAGGGAAAAAAATCATATATATTTTCTTTTCCTTCATTTTCTCAGCAAACAACCAGGGAAAATATCCTTTTTTCCGTTAGATCTATTTCATTCCCTCTTGCATCCGTATGTGTACAGTGTAAACACACACGTATATGTATACATATGCCTGATATGTGTTTAACCCTTGCAGGTATTTGGATCAGGTGGTGAGGTGAAGACTTCAAAAATAGTCGTGGCGATCGGATTATTGGAACAGCATGCGGTGGTATTCTCTCTCAGTTGATGTTTTCCGGAAAGTAGTCTCCTTGAATTTGTACTGTCGCGTTATTCTGTAGCAATACCCTTAAATCAATTCAAACACAAACCCTTTAACTCGCCCTTTTCGTCAATACCTTCTTAATCCTCCATGGGATCCAATCTGTTTGTTGTCATTCCTTGCTACGTCCGCTGGATTAGCTGAACCTCTCTTGATTCCCTGCTTCATTGTTGAATCTATAATTTCTACATTTAATCCTATCAATCACCGGTCTTTTTCGTTAGTCCCTCTGTTCAATCCTTGCTGGCATTTTCTTTGATTTTTGTTTATTCGAAGGTAGCGGTGTTGTTTCTCGGTAAGAACTGTTGGGAAAAGTTGTGGGTTTTTCTTTGATTCTCGATAGCTGTGTTGTTTTCGATAAGAAGGGTTGTGAAGTTGTGGGTTTTGCGTTGATTCTCGTTTCGTCGAAGATAGCGGTGTTGTTTAGATAAGAAAGGGTTGGGGGAAGTTGTAGTT

>RsbHLH049

AGAAAACCAAAGGCAATTCGGAAACCCGAATATCTAAAAAGTACGATGGATCCCTGCTATATTCTCTTGTAAAGTACAAAAAGTGGGAATAAATTATAAAGGCTATGTTCGTTTAGCATATTTAGTTTGTTTTAACTTTATTTTCCAATCATTATCCTATATCTTTCTTCAATCATTACCCTATTTCTCCAATTATTAATTTCATTTCTCTCTCTATCTCTCTCCATTTATTACCTATCTCTCCAATCATTACCCTATTTCTCTCTCCACCCACTACCAAAACTAAAATACCCAAATGTGCTAAACAAACATGGCCAAAATCTTTCTCCAATATATCGCATCCATTCTCTATAATGCTTCAAAGTTTTATATTGTGAAAGAGATTTTCTAATTTATTCTATCTTTTCAACTTTTTATATTTTATGAGGGATTTTGGAAATATTTTCAATGTCATTTTTAGAAATTTGGTTTCTAAAATGGAGATAAGGTATCCATTTGTAGAAACCCAAAAGTAAATTTGGAAATCAAATTTCTAAAAAGAACGATGAGTCCCTCTAAGATTTTCTCTCCTAAAGATACAAAAAGTTAATAAAAATAAGTGATAAATTATAAAAATTTCCCCCAATATAGATATTTAGAAAAACATTATGGAGAATTGGAACAAAGGAAGTGAAATCCGACACTGACGCATCCGTGAGCATTTGCAATCTTTAGTATTACACTATTTTACTTTGGTGACTATCTCTAATGTCTATATCTTCGATATCATGTATTGAACTAATGTTCACCATAGAGTAAATCTCACTTGTCATTTACTCAATCAATGTGATGGTAACACATCACGTAATAATCAAAATGCAGTCACCAATGAGGAATTTTTCAGTGCCGAGCGGGTATTGCCGACGTGGCACCTGCTCGGCGCACCCGAGCCGTTCGATGCATTTTTGGACGACTCGGATTAAAACACACTCTCTCCTCTCATCCTAACACTTTCTCTCTCCTCTTCTCTCTCCAAATCTGAGTCGTCCATACACATAATGGGCGGCTCGGATGACCGAGCGCGCACCACGTAGTACCCGCTCGTCACTGAAAAATTTTTCGGCACCAATAATGGGCCCCTGCGTTATGGCTGTTGGACTTTGAACATTTGTGAATCGATTTGACAGGAAACAAAAAAAAAACTAACATGTACTACAAATAGCAAAAGATTAGGGGTCATTCTGCAAACAAGTTGGAATACCGAGCCGTTTCTGCAAAAACTTCTAGTCACATTTATGATTAAGGCGGGAGATAAATCGAATTTGATCACGCATTAGCTGTTCTGTAACCACCATTAACCCTCCAAATCTTCATTCTTCGCAGAGAGAGAGAGAGAGAGAGGTAGCTTTCCTTAGAAAGTGCGTGCAGACAAAACCCAGCGATTGACGCGATCCTTCCGGTTCGTCTTCTTCTTTTTCCTTGTACACAATCATCGTGTCCTGTGATTAGAAACGTAAAGAT

>RsbHLH050

CCTCGGTTCCGACTTTGGCGAGTTGGTGGTTTGATAATGGGTTCCGCAATCTCGAGATACACATTTGGGAGTCCTGCTTCTATGCTACACTATGGTCCATCTGGTCGGTACGAAACGAGTACAACTTCAGCAACTCCACAAAACAACCGTGGGAGGTGGGGGATTTGGTCAAAACTAGGGTTGCGATGTGGATGAAGGCGAAATTCAACATCAAAGTCTATTCTGTGGAGGACTTTAAGATTTTTCTAGATGAAATCCGGAAATTAAATTGTAAAATTTCTCTAAGGTGTTTTATCCAACTTTCTGTTGGATGCTTGTCTTTTCCTTTTCGGAGTATCCTAGCTTTTTGCTAAGGTGCTCCTTCTCGATATACCCTTTTGAGCTATAATAATATTTCGTTTGCCGAGAAAAAAAAAATTTATCTTTTGCCACGCATGAGATGACACGATAATAAACAATGATGCTATTCGTTTTTCTTATCAGTTAAGTTTTTGAATTGTTAGTTCTTATTAAAAATTTAGATATAACACCTAGTCATGTACTTTCCTTTCAAATCTTCATTTTTGACTAGTGTGATTCATTCGACTCTAAAAACTAGCTAGCATGGTGGTTAATGGTTGAGATTTATTTCTAAGATTTAGATTCAAAACCTCAGATCTGTAACTAACTCAGGACCAGTTTATACGAGACTCTCATAGGCTTTAATGATGAAGCTCTCGCTAGTTAACAGTAAAACTGGTCTCCAATAATTAGAGCAAAAAATAATAATTAGAAGAAGCACAAACAAATGGACCCGGGCACTCTGAATTATGAAAAAGGAAGTGGGACTCATTAGACATGATCCAATTCTATCATGGTCATAAGCTTACAGGTGCCCCCAATTTCATTGGTGATGATCCAATTGAGCCAGGGGAACGTGCTTCATAATGCCCTTCTAGGCAGCAATCATGTCTCATGTTTCAAAAAAAATTTGTAACCATAATTGTTGATTATTTTTTAGAATAAAGAAGAGAGAAAATAATGCTCATCCGCTCTTTTTAATTAAGAAGAAAGAAAAAGCCCACCAACTTTGTTCTTAAAAAAACAAAACCAAAATATCAAATATCAAATCATTATAAACAACTAATATATCATATTATATTCCCCAAGTTGCTATATATAGTAGTCTTCCCACTGTTTTGATAGTCTGTGCCCAATATCACTTTTTTATTCCCCCTCTCTCATTTCTTCTCCTAAACCCTAACATTTCTCTTCCTTCACTGCACACTCTGATAAAACTCCTAGTCTCTCTCTCTCTCTCTCTCTCTCTCTCTCTCTCTCTCTCTCTGATAATACTGCTAGTCTAAACCCTCTCTCCCTCTACCTTCCCCATCCCTCTGCACTTCAGTTTTAATTTCTCTCAGTTCCCCAGCTTTTACACAACTTCTCCTTTTCCCAATAACAAAATATTTTCTCTTTTTTAGGGTTTCAGCTATAAAAAAGCCTCATCAACTTATTAGG

>RsbHLH051

AATAGCATTTTCCCAATTAGGACATTTACTAGGTCAAAATGGGCATGCAAGATCAATTGTGAATAATGGTGGAAAGAGTAAAGTTCCTTTGGGCCTACCCCTGACATTTTGAGAGCCTAAAACGAACTTCAAAATTTGGGTCCTTTTCGTACTTCTATGAGTGAAAAAAATAATAGTTTATCAATCTAAAAATTTTAAAAAATATATAACAAAGTGGATTAAATTTCCGAAGTAGTGATTATAACAAGAATTAACAATTAGCACTTAAATATCACCCTTTTTTTTTTAATTTTTAGCGAAAAAAAATAATTACGTTTGCATAATCAATCTGTCCTGTGATATTCTTTTGTAATGGATAATATAACTTCAAAATGGGACCTTCGGAACTTTTATAAGGAATAATTATGGGCACTTTGGGTTTCTTCATAATGGTTTTTTTGTTTTTAGCTTAATGGGCTTATTTTTTATATTTATCACCCAATAAAAATTTGGGGGCCTGAGGCACAAGCCTTCATCGCATTCCATCATGAACAGATGTACAAGTAAAAAGCAGGATTGAAGCAAGGTCACTCGGCTTGGTCGTATCCCAGACATAATAGGGATCTTCATTCCTTCATTACCTCCACCATTAAACAATACAAACCAAGGCACTTTGTTCATACTTAATACATAACATTCATCTGCACTCTCCGTCATCTAACTTGATTGCGGGAGAGTCCACGACCAGCAACAGTATCATTGGCCATTGGACTTGTTTTGCTGGAGTCCCTTAATTAAATTGTTTAAACAAGAAATGCAACCACCGTTTTTTGCATCATCACTCACTTTACATATTGTTCCATATTTCCATATGCTTTACACATTTCCCGGATACTTTTCAAATATACAAATATTTTAGAAGTTTGTAATGTGCAATTATCAACCGCCAATAGCATAACTTCATCACAATATTCATGGGCACCACGTACTATTTGTACTTCCTGTCAAATAAAACAGCACGCAACTTACGAACACTGATCCTCTGTACCGAAATTCTTCCGTACTGAGGAATTCTGACAGTTGGATCGCATATAAACTAGTAAAAACATATAAATGCTCATTTAATACATGGGTATACAAAGGATTTTTTGTGCCAATTAACTTACAACCTAATCTGAATTATTTGCCACCGCGTAAAAGTTCAAAACCACAAGGCCCAGTAATAATTAGTATTTCCATATCTCCCTTGTCTAGAAACTTTCTATATATGTCTTCCATCACACTATCACGTGCAACATTCTGTTCATCTCATTATTGCATACCCATATAAATGCCCCCACCCTTAAGGCTAAGAGGACCCATGGGATTTTGTTGAGACTTCTTTTATATTGGTTGCCTTTAGAGTTTAGATACAAATATTTCTTACACACAGACACAGACACAGAGAGAGAGAGAGAGAGAGAGAGAGAGAGAGAGAGAGAGAGAGAGAGAGAGAGAGAGAGAGAGAGAGAGAGAGAGAGA

>RsbHLH052

TTTAGCCAAAATAAAATACTAAGGTTTAATTTGTACTCGGTGATGAACGAGAGATGTTAGACTATATTATAGTATATAAAATTAACGGTAGTCCCGCATGTGGGTTCATGAATACAAGCAAGCAAGATCTATGTACAGTACTACCATATCTTGATGCTGAGAGAAAAGAATGAGGATAATACCAGGCCCGGCTCGGTCTTGACATTTTCTGGCACTAAGCGGTGTTTGAAAATGGGACCTCTTATTGTTTTGTATATTAATTAGTTGTTCAATATGAGAACCAATTAAGACGGTCTTGTTGTTCAATGGTTAGTGTTTGTACCACCTCTTTTGTATGAGTTCCAATATTGTTGGCGTTAAAAACTGAGGTGGCCAGGCATCGATCGAACTCTCGACCCCATGCTCAAAACACACACGGGCCTTACCACTATGCTACATTAACCTATTTTGTAAAATATTTCACATAAACTATAACATTTAATATGTTTCGGTATTTGGGCCTAATGAACTTATACCTAGGGCCGAGACTGAATAATACCGGAAACATCGCTTATCTTGTAGAAGCTCATACATTTAGTATTGAGACCCGTAAGAATGTGTGAGGAAAAAAGATGTTGAGACATCACGTGGCGATACCCGAAGATCATCAAATAAGTTTTCCTATCTCAACCCTTCATTGGTAACCTGATACTGAAACCCGTAAGAATGTGTGAGGAAAAAAGAGATTGAGGCATTACGTGACGATACCCGAAGATCATCAAATAAATTTTCCTATCTCAACCCTTCATTGGAATCCTGATCAAAAAATTAGATTTATAAGACGCATATGGGCTCACCCAATGATCTGAATTGTTCAATGAAGATGCACCAAAAAGGATCATAGATCAATGACAAAAATTAATTAACTGAACAATACAATGATCTCAACCGTGCATTGAGGTATAGCACCGTAGCACTAAAAAAGATCATCAAATCGGACGTTTTTGTGCACATAATCAAAACGTATTTGCTTTAATAAAAAAGAAGAGAAACAATCAACTTACTAACTAGACACTTGAGTTATCAAAACAAATACTACTACTATCATTTATACAGATATTTGCTGTAGTTTTAATTGGAACTCCTGCAAGTACATGGTGAAATTGGTCTTTCAAGATCAATTAAGGTGTGCGAGAGTTGGTTCGGACACTATCATATGATTCCTGAATTGAGACGCAAGATAATCCTTTTCTCTCTCGGTTTAACCCTTCTGGATTTCTCCCCCTCTCGATCCCCACTTTCAGTTCTCTCTCTCTCTCTCTCTCTCTCTCTCTCTCTCTCTCTCTCTCTCTCTCTCTCTGATCAAGAGAATTCAAGATGTGGCCTTTGCCATGAGAAGGGTGTTGTCTGCTCCATAAATTAGAGGAAAAGCCAACAACATAGGGTTAGGGTTATCCCTCAAGACTCAAATGCTGGTGGAATTACCCAAATTATACGTAACATATACTGCAAAGCTTTTCT

>RsbHLH053

TAACAAATGTTCATAACATGTCTTTGGCGGGTATTAATTATCAAAACACATCATGGACCATCATTTTTTCATTATTTTTTATGTTTTTTTTAAACAATTATGGAGAAGGACAATTAATCAGGACAGAGAAAATAACAACCCATTTTAATTTTCTGTTTGGATCCCTACAAATAAAAATAAAGGGAAAACTTTGGAATACTTTATCTCCTCTCACGTTTCTCATTTCTTCTCTTCTTGCCTTTTATGACTCGAAATTATTTCTCTCTCATTTCTTCACTCATATGTACATGTCACAGACTTTTAGGAAAATTTATTTTTCTTGGTACATTGTCATATGATGCCTACTATCCATCACAATTCATTTATTTATCCGAGAAACCTACAGCCACCGACCCATGGGGTCTCAAATCTGCACCGGCGGCACGTGGGGTCCACTCCGGGCCTCACACAGATGATCAGAGCAGTTCATAATTTCTAAAAAAGATTGAGTGGACCCTTGAAGAATAAGTTCAATCTGATATGTGTAAAAACTCGATTCAATCTTCTATTTTTTCATTCCGATTCTGGATTCGTGAAATAAATAAAAGATTGGATTGATCACTTACACATATCGGATTGAGATGATTTTTTTAAGAGTCTACTTAATAATTTCTTTTAAATTATTAAACGGCTCGAATAATCAATGTCAGATTCAGAGTGGACTCCGCTTGTCGAAAGATGCAGATTCGGGACCCGAGGTCGTTGGCTGTAGCAAGATCCTTTATCACAATACATTTATTTGAATCTATACACTGATTATAAATCCCACACAAACATGTGGTGGTAGATGATGCTCACAAATTAACTACTCTTAAATAATCACTCCGAATAATTACTCTTAAACCTTAGTTCAATCAGGCTCCGTTCCAGAACACCTTCTTAAAAAATAAGTACTTATTTCACATTTTCAAACTCAAAAATAATGTAAATGAAAAATAATTTTTCAATTTTTTTTGCACCGTATTAAAGATCTCAATGAGATCTATCAAATAAGATCCATAGTGATAGAAAAATTATTTGCGTAAGCACATGATTTTTAAGCTTGAAATTGTCTTCTTAAAAAATAAGTTCTTATTTGGGATTCTGGAACGCACCCTCAGTCTCTAATCTCCGCCGGCTTCCCCAGCCAAAGCCGCCGAGTAAATCGCGCGAAGCCTTCGTCGAAGGCGCCCTTTTCTTTTTCCACTCGCTTTCTCTCTCTCTCCTCAAACCCAACACCCTCTAAAATTCAGCTCAAATGCAATCCAAATAAATCCCCAAACCCAAATGAACCCGAAATTGTCGTCCCCCACCACCACCCTCCCTTATCTCTCTCTCGCACAACCAATCCTCTCTCATCTCCCTTACCTCCGCCCTCCAGCCGCCTTCTACTTCTCTCTCCTCCCAATAACAATCAGCTCTAAAACCCCACTACTCTCTCTCTCTCTCTCTCACCTGGAGGCCTCGTTCCCTCTGTATGGG

>RsbHLH054

CTTTATTTGCACAGGGGAATTTTCAAACATCCACCCAACTTGGTAATATGGAAGTATATGAAATCAGTTCCATAAAGAAAATTGGGTACCATGGAATCCCATTCAATGTATTTTGGAATTTAAATCATTTAATGTTAGATGGAGTCTTAAGCTTAGTTACCTCCTCAGACTGCAAAATGATACCGTAAAGAAGAAACAATGAAATTGCATGGAGCAACCACCACAATTTACTATCCTTTTTTTCTGTCATTATCGAAAACCAAAATCACTGAGGGCAGAGGATTTAATCCAAGTCATTTGGTTGCCTTGGCTTATGCTTTTTGGGAGGAAATGTGGAGAAAATTTTGGGAGAAATGCAAAAAAGTGGGTTTTGGAGAGAGAGAGAGAGAGTGTTTATGTGTGTGTCCGGGAGAGGGATGAGTAATTCAACAAAGAGTCATCACTAATATTTTGGTAGGAACACTTTTAATTTTGGTATAAACAATATTAATTTTGGCATTATCAGCTGCCTTAATTTAAGTTTGTTGAAGGGTTTTCTTCAGCTTTTAGTCAAGAATTTGACAGTTTGAGGTGGAAAAATTGCTGGAAAAAAAAAAACAGAAAGTCCAATGGTACCAGTGGATACAAATCCATTCTTGGGCCCATTTTGAGTATAAAAAAATGATCGGAACTGCTCATTTTTTTAAAAATATTTTGCTTATGGTATTTGCAAAAAATAAGCTTAATTCGATATCGGTAAAAGTGTTTACGAAACATCCAAACTTTGCTCTAGAATTCAGGCTTGAATTTTGGGATAAAGTTGAATGTTTTGTAGATACCTTAACCGATATTGGATTGAACTTATTTTTTATAGGAGCTGTAAACAAAGTATTTTAAACAAAATAAACGATTTTGATCATTGTTTTGAAATTCAAAACGGGACAAAAAAAAGATTTGTACACACTAATACAGATTTTATATCTTTACCAGTAACGTTGAAAAAAAAAAGGGTGAAAGAATTGCCTTTAAGAGCTCCCAAACAAGAGAACTTATTATGCAGCTGATAAATAATAGAATTTGCAATCTGCCATTGCTGTCCTCTTCACCCTACCATAAAAAGCAACTGGGTCTTCTTCTTTTGAGCTTCAGAGCAGGATCTGATTGAGGAAATGGATGATTAAGAAGAAACCCATCTCAAAAATAAATCCCAGAGTGAGTTTCTTATGTGCTCTTTTGCTAGATAGTCTTATCTAATCTACTTTCTTCCCCACAAAACACTTTCTTTCTTTTCCCCCTTTACCCTTTTTCAATGTGGGTGATGACTTTTTTCTATTCTCAACATCAGATGCTTATGAAAACTCTGATCTTGGTTTTCTATCTTTACCCCAAAAGCTGAACTAAAGCTACTTCAGAAATCCTAAAGTTCTCCTTTAGAATGGCTGTCATAGTTTTTGTTTTTGCTTCTTTTGACTTACTGAGTAATGGGTTGTTTTAAAATTTGTGACTTGGGTTTTTAGGCTT

>RsbHLH055

GATCTACTGAAATGATATTTTCGTCCGGCAATAATTCTAGGGACACACCCACTTTCACACCCAAACACACATCCACCACTCACATGAGTGGTGGGCCCCATACACACACTAGAGTGTGTAGTGTGTGTGGGACCCACCACTCATGTGAGTGGTGGATGTGTGTTTGGGTGTGAAAGTGGGTGTGTCCCAGCACCACTCTTTTGGTTACACTTCGTTTATTATACACAATAACAACAAAATGACAGATGAAAGAAATTTAATTTGAATCTAACTACCTTGAAAAAGAGGTTAAGTTGGGGATGCGTAGTGCCAGAGTTGGAGAAGAAGGAAGCTTTAAAATCATATAAAGTGTTTTTTCAAAACAGAGTCTGTTAGCTCATACTTCCTCACATGAGTCACTTCACTTGTTTCAGGCCGGCATGAACTTTGTTGAGTGTACTGTATGCATGTTAAAGATCAGCACGAAAGCACTTGTCTTACCATCTTTTTTTGATAACTCAGGTGTTCGGACCAGCTTGCGCGCACCTCGACTGATCCTTGGGGATCAATTTCATCGCCCATTTGCGAGAGTCCCAATTAAAGCCAAAGCAGCAAGTGTCTTACAATCACAGACCTAGGTTGTTTCTTATATCTATGTGTTCTGTTTTATGGTACGTGCTAGTTGTGTTTTAGCAAATCATGTACAAGGAAGATATAGACAGTTGGCAAATATATCTGTGCTCCACGAATGTTAAGTCCAGTCTATTCCGATGTCTTCTAGTATACTCCAAATAATTGAAATCTTAACCTTTGAATATTTTATATGTATAAAGAGCTATCAGACTTAAATTTGAAATCCAATAATCAGACCATGGGCCCATATATCAAAATCTCCCACCTAAATCTTGCTCCCTTGCAATAATGATGAGTGCCAAGAAATTACATTAAAAATTCTACCTGGCTTTTTCTATTTTGAAGGAGAGGGGGCTTAGAAAAGTAGTACTCCACTATTTTATTTCTGACAAAGATCCTGTTACTGTCAATTCTTCCATGTTTTCTCAATGTCCTACTGGCAGAATCCTAAGATGTGTCCATCTCAACGATCATGAGAGTTGTTCACACGTGAATGCCATTGAAATTAGAGAAATGCTAAGCGCAAAGAAAAGTTATTCAGAAATTATTTTGAAAGAGCAAATCAATGGTATATGTTCACTTTGAATTGTATCTGTACCGTTGATTTGTCCATTCAGGATAATTTTCGGATAGATTTTCTTTGTACCTAACACTCTTCTTGAAATTATTGCAGCCCTCCAAATGTATAGGGTCTTAAATAAAGGAAACTTGTCAATCCTTTTGTAATTATGTGGAAAAATCCAAATATGTCCGAGGACTAGGGACGCTAGTCCCTCAGAAGATTTAAAATCTCTAAGTAAACATACCTAAACGTGTGTCCCTTCCTTTTAGAAAGAGAAGATAAGACAAGAGAGAGAAGCAAAAAGAGTGACTTCTGAGCATTTTGGT

>RsbHLH056

AGTACAAAGATCAAGAGCGTGAGCACAAGACATGTTGAAGAATTATGTGACTAAGGTCGCACCAGTTATTTATTATGCAACAAATCAAAAGTATGAAAATTATACAAGTATTTGAAGCATTGAAAAAGAGACTTCATCAAACTTCTTGGGAGTTTCCACATCCATGCCAAACCTTTCGTGTCAACATAGATCACGACTACATAATGCAGCGATTAAAGCTCTTCATATTGACTTTGCCTAAACGTCATAAGAGACATGATCAAGATTCAGAGCTATGCTTGTCACCTTGTTTTTTTCTTTAATTGCTTTTTACAATACAAAAATATTTTACTACTGTAACCTTTCGTGTTTTTGGCACGGGGCTCACCGCCTAATATGTGAAAATACAAAAGGCTACTGTTAGGCTACAACTCCACTTGCCAACTAATTAAGATAAAAATGAGACGAACATCATGAGTTGAGAGAGTCGACAAAAGATACACAGAAACTGTTTAACCGATGATGACTTTTCTAGGGGTTGTGGTTAATAAAAAGTAATTATTCCGTTCCCAAATAATACCGTTCTTTTTGAGTTCTTGTGTCCACACTTGAATTAAAAACCTCTTGCGAAGATTTGGTACGACTCTTAATTGAAAATGACCAACAATTGGAAACAGATAGAGCAAGACTGCAAGAGCTTATTGACTCTCCTTCATTTACACTTTTGTTTTCAGTTTCTGATACTCAAAAGTACTCAAATATCCACAATACCGTAGGGCGAAAGAGAAAGTAGCACACTCAAAACGTACACCTTTCACATATCTGGCCATGACTACATAACATATGGAAATTATCCACTTTAGGAGCAACGGATTTTTATATTATCTTTTTGTTTCAAGCCTGAAATACTATCTTTTTGTACTACGGAAAGTATTGCTGAAAAGTGAATCGCCAAGTGTTCTTGGCATGGAGTTTAAGAGTTTACTAATGTTCAATTCTTTTTACCGCGCGGCAACTCTTGGGCCGGGCTCTCACTCCATGCATAAGCGTGAGAAAGGTTGTGAGAGATGTTGTAGAGATCAGTTCGCAGTGCGCGAAAGCTGATCCGGTACACTCCGCATTAACGACAAAAAACAAAAGTGTTGGAAAAGTGGACGGACTATCCATTGCTCTTGAAAGCATGGTGAATGTACATACAATGATGATAGGAATTAAGCTATCTATCTTAGCTAGTTGGCAAAATCTCTTTTGTAGATACTCCTCTTTCTCTGGCATCCAATTAGAATCCCCTCTCCCTGTCAATCTACTTATAGGGACCAAACTCTCAGCCCCCCTGTCCCCCATGCATGTAATACTGTTCTTTCTTATCCCCAAAATAAATACTCACTAATCCTTCCCCTCCTCCCAATCCTCCTCTCCATCATTCCCACTCCCACTCAAGGAAATACCAACATATATAAAACCTAATTACTCTCTCTCCCTCTCCCCCCCTCTCTCTCTCTCTCCAAAATAGTAAGAAAG

>RsbHLH057

TGAGGTGATTAAATCTTTCACTACATGCAATTGTCTTACAACCAAAAGTTGTCTAATCTACACGGACATGAGGTCTAGCTCAAAAGGTACAAAGCGTGTTACTAATAACTTGATTTGTGTCGAGAGTTCCAACTTCTCCTAATTTTGGCTAACTACATGAGACAATATTCTTGCCTTCCTATCTGTCGAATACCATGGCTAGGGCACTATTTTTACACTTATAGAGCTAATTTTTGTTAAGGTTATCAGAATAAATAGTTTAGAAATAAGAAACGGATCAGACCAAGATCGATCATTTGTTTGGAATCTTGAAATAAGGCTCTATGAGCCTTATGTCCATCATTTGTGCACAAGGTTAAGCCATACGCTAATTAATTGCGTAATCTTTCAAAATGCTCTATGTTCTCGTTCCTAATTTTTGCACTGCGCTCACGCTCTTAATTCTCTCATTCATGAATTTTAGTAGGTTGAAATGTATATCAAAAACTGTTTTACTTCCGTGCTCCTGCACCCACTCACGCTAGTGAATTATGTGCATATGTTATTTTTGTTCGACAAGCATATCTTTATTTCTTCAAAATAAAAAATGGATGAGACATATTAAGAAGAGTTTTTTTATAATTTATCCTTTTTTTATTTAGTATGGAGACAACGCTTTTCTTTTTGAGACCAAATCCCCAAAAAAAAAATAATCAATTCCTCCACAGTTTTCTCAAAAAGTACAAAAGATAAACAAAATGTGAAATAAATTACAAAATTTTCCATTAATATATCTTATTTATTCCCTACTTTGAAGAAATAGAGATGGGTCCTGATGCTGTTAGCCTGTTACATGGGCCAGGCTGTTCGGGCACAAGGCATTGTTTGGGTAGGAACAAAAAATGATGGGGAGGAGAATGATTGGGATCCGTTCAAATAAAGCTGCAACATGTATCAGTGTACAGAGATCATGATTGGGGCCCACTTATGGGAATCTCAGTAACAAACATCCCTACCCTGGCAAAAAAAAAAATCAGATAATACAGGAGCAGCACCCTTCACTTTCAGTCACCCCCCTCAACCTACCCTTTATATATGCACATCCTCTCTCTCTCTCTCTCTCTCTCTCTCTCTCTCTCTCACATCTTGGAATTCTTGATTTTTTACAAGGCCTTGCAGCTGCATCTTCTCTGATAATTTCTTCTGGGATTTTCTTTACATAAAGCAAGAACTTCTGGGCTTGAACAGGTTTCATCTTTTTGACACAAAGTCCAGGCATAGTTTTCATTCACAGGTTAGGGTTTGTAACTTTTTTCAAAACCCATCTTTTGTATGTATAACTTTTGTCTGGGTCTGAATTCTACTTTGGCATATTTTGTCCCTATTTGGTCCATGAAACTAGTACTGAAATTGTCTTTCTTGTCATTTCTGTTGTCTCTTTAATGCATGTTGCAGACAGAAGACCCTGAAATATAGGAGACAAACAAACATCAAAAGGCCTGCCGATTTGGATCCCTAAAA

>RsbHLH058

GATTTTACGAGAATCTTATTCCTACATAATTACTCATTTACTAGGAACCACTACAACAGCAGCCGCATCCGGTGCAGCACTCGCAGGCTTAACAACATTCTTAACTTCCCAAACCTTGTTCTTGGGGTGGTCTTTACCAGCTCCAACAGCATCCGCACAGGTCTTGTGACCAAATACCATGCACTTCTCACAGCTAGGAGGCTTCCGTGGATACCAAACTTTAATAGGAAAGTTAGTACCATTGGCCAAAATCACATCGAAACTTCCAGGTAGAAAGAGCCAACAGCAACCTCTGCGCATATCTTTGCATAACTGAGCCTCTTACAAGTTTCAGTCATTTTATCTGCATATAAAGAAACACCCACTGCACTTGCTGCATTGCTCAAGCCTTCAGCTATCCCCAACTCAAGAGGAACATTGTAGAATTGAGCCCCTCAACGCATATCTTTGCATAGCTGAGCCTCTTACAAGTTTCAGTCATTTTACCTGCGTATAAAGGAGCACCCACTGCACTTGCCACATAGCTCAAGCCTTCAGCTATCCACAACTCAAGAAGAACATTATAGAATTGAGCCCACGAGGGAATCTTCTTCAGTTGATCCTTCTGTAAATTCATCTGGGGGAACCATTGCTTAAGCATCATTAATCTATTCCCAAAATGCCAAGGTCCAGCTTCAGCCACATCCCTATAGGCCGCAGCCCTATGAAACGGAAAGAAGAAATAGCCCTCTTCATTGGACAACATAGCAGTTAGGCCAAACTTTCCCCAAATCTTTCCCCAAATCTTATGAGCAATAGTCTTCACAGCCATGAAAGGCAATTTACAATAAAAAAGATACCCTACAACACAATCCTTCCATTTGGCCACCCCAAATTTGCAACATCCTCAGGAGGGCTAACCACAACCCTACCATTTGATACGACAAACGAATAATATTGCAATTTCATACTTGGAGTCGCAGATTCAGCACCACAACCCTATCCTTCCAGGACATATTACTAGGACCAAAGACACCAGGTACAGAAGGAATACCTGTCACTGGACCATGACTCATTCAGCAGTTTCTCCTTCTCCTCCAAAAAGCGCCTTCAATTTGATCACTTCAGCTTTCAATTCGTTGATTTGCTTTGTAGCCGATCATCCGTGGAAACCCCCAAGTGTTACGACAACGACATAGACTTCTTCTCAAGCAGTTCGATGATGTTAACTTTCTCAGAAGCCCTAGGTGGTGATGAACTCAAAACCCTAGCACACGAAACCCCTGAGCAAAGAATACAACCATCGAGAATAGGAAAATGATCCTCATAATCCCCAGCCACGATGGCCAGGAAGAAGAATTAATGCTCACAGCTATGGGCATTTCAGGAGCTCTTGTTTGCATGTAGAAGATTGTTCATATCCTTTTCTCTTCCCAGCTGTTATTAACGCGAAATTGATCATAGTTCCGATAGCTTGTGTATTTTTTCATATGATTATTACGTGCTGAAGACTTTTGATAA

>RsbHLH059

TAGTCCTTTTTAGGAGTTCGTATAATTTTTCAATCGATTATATCTTGTGATTTATAATATTTTGTATAATTTTGAAAATATTATCTTATAGAATTAATTGAGATCTATAAAATAAGATTCATATTAGATACATAAAATTATTATCAATTTAAATATATAATTCATTTTTTATTCAGTTAGAATTGAAGGAGACTATCAAAAATCCGAACCGAATGAGTATATTTTACAAAAAAAGCTACGTGCACAGCAAGCTGTGCACAGATTCCATTTTCTTCCACCTCAGGTCGCAAAAAGATGATCGGAGCCGCACATTTTATTCAAAATATATCGTTTAAGGTCTTTGTAAAAAATAAGCTTCAATCGATATCGTTAGAGGCGTTAACAAAACACCAAAAAATATGTCTATAAATTTTTGCTTGTTTTAAGGAGAGTGATTAATCACCTCTTTTTCTTTTTTTTTTTTTTTGTTAATGTCACCCCCTTATTTTCTTTAATCACCTCTTTTTCTTTTTTTTTTCTTTTTTTTTTGTTAATGTCACCCCCTTATTTTTCTTTGTTAATGATCACGCTATATGGGCTATTTGTGTGCGTATATATAATATGCACATGGTTTGACATGTAAAATAAACAAGCCATTGGAGTACTACTATTATTCCTGCCGACGAGTAGTGGAGTATTTCTCAACCTCCCCGTTAGTCTCCAGTCTCCCAAATCCCAATCATCAAACTTTCTTTTCAATTTCCTTCCTTTGTTTGACATAAAAAAAACTACATATAACCACGGTTTGATTTCTAACCCTAAACCCAATAACCATAGTCCACAACTGCAGTTGTGAATTTTTAGCAGAGAGAGAGAGAGCACAATAACCAAGCCTCCCTTCTCCTCCCTTCTCTCTTCTTTCTCTCTCCAAAACTTCTTCCTTTCCCTACAAACAGATACTCCTAAAAAGTTCCCCCAGTTTTTTTCTGTCAAACCACCGATCGTTTGTTTCCCGGGAAAATCATTTCCTCATTGGCTTGTCACTGTCCGGGATTTTTTTTGCTACAGAGAGAGTCGTTGCCTGCCTTGCTTCTCAACCGTCATTTCTGGTGTGTATTCTCTTTCAATCTTCTGGTCCGGATACATACAGGCAGTTATTTTAGTCCTTTTTTAAAAAAAATAAATTTATTTCGGTTTCGTCGATTGAAAAGTAGCTGTTAACTCAGATTTAATACTTTTTAGATCTGAGAAATAGCCCTATGATCGACTGGGTATTGGAAAAGAAGATGTCTTGTAGTCTAGCAACTAAGATGCGTCTGTTTTTGACCTGGTTGTTTAAATACTAAACGTCTTGGATTTTGACCTCTGCGGGAAAATTGGGGTTTAGGATTTGTTTTGTTTGGGTATAGATTCATCTGGGGAATTATAAATTGGTATTTTTTGTTGTTCTGTTTGGGTCATCTGGGTATTGAATTTTGGACTTTTTCTTTTTTTGGGTTCTTTTATTGGAGGGTTGTAGAG

>RsbHLH060

AAGTAAAAGGTTTGGCATTTTAACAGATTTCTCTCGTCATGAAAAGGCTATAATGTTAAAAAAAATTAAAAAAAAACTAACAAATGCGATTTTTTTTGAATAAAGATAAAAAAAAAAAACTTTCTGACTTAATTAAAATGTCAAACCACCCCTAAGTTTCGGTTTATCTTTTTAAATACTATTTTGGAGTGATATATCTCAACTGAGTGAACCAGGTTTGAAGTCAATTCATACATATGAAAATAATTTGCACTCCGTTTTTTATAATTATATTTCAAAATTTATCTTTTAGTACTTCAAATTGTATAATTCTATAATACGCTATAGTATCATACTACTAGTATACAAAATTATAACTACAGTTCAAATTTTATCTTTCAGTTACACTATTAAACAAATTTTAAAGTGCATCTTTAAAAAAGTGAAGGGCAAAAATAATTTTTTAACCATACTATTTTATTCATGGTTAGCTGTTTTGTCTGGGATTTAAAATTTCCAACCATGTCATATTCTCTCAAAACAATTCTTCCCCTTAATTAAAAACTTCCCACGTTCTCCCAATTCCTAAACTACAATCTCACTCATTCTCATTCTGCCTAACCATCCTTAAGTTAAGCACTGACTAATAAGCTTAATTAGTCACCCCATTTTTATATATTTTTGACTCATTGGAGTAATCTTAAGCATGGAGTGTGTGTGAACCCAAAGGCCTAGCTATGTGAGTGTATGATGTGTGTTCTTGGCTTTTCTGCGTCCAACATCCCCATCAAGACTCGTTAGGTAAGCCCGCACCATTCATCGAAGTGAGCATTCTGATGCGCATAAATCATTAATCATCATACTATTTATTGGAATAGATTCTATACTAGTCCGCCCGTGTGCTTGTGTAAAGCCCTATTCATGAGAATAGAAGCTCACTACACTCGAGCTTAATATGCAGTGTTTGAATCTCATATTTGTGGAAAAGATAATTTCAACCAATGTTTATCATTCCATTTCTCTTCAACACTTGTGTCCAAGTTCCCCAATTTACTAACAGCACGTGTCCCAACAGCAATATGAATACCTCTAATCATTCTGATTTCATTTTCATTTTAAGCCTAATTATGAGTTTAATAAGGTAAGGCTAATCCAAGAGGGTGGCCTTATTATATAATTTTAATCCCATCATTGCATGAACTTGGTCCTTCTCTGAGTTGTATTTTGCCTGGAAATAATTGATGGCCCCACAGTGGACTCCCTAAAGAGATTAGTTACTAGTTACAGAGAACTAGGACCTACCCACCCCTAATCAATTTGTTTTTTTTTTGAATATCACCCCTAATCATTAGTAATTAAAAAATCTCATATAAAATTCAAGTCAACCCCCCTTAGTATAAATATGACTCCAAGTTTTTGCTAATTTCAAAGCATTAATCCCCAAATTAGTCCATATTGCCCCTCTCTCCCCTTCCTCTCTCCAAAATCATACTTGCAATTTTTTAGGGAAAGTAGAGAGAG

>RsbHLH061

GATTTTTGATCAAGTCATGTGAGTGCAAGAGGTAATTTAGAGTAGAATAAGGGCTGTTTTGGAGCCCTGTTTAGTGCCAAAATGGAAGTATTTTGGAACCTCTGTAGCGCCAAAATGAAAAAAGTTAGATGGTCCCCCTCCCCTTTTGGAGGGCTTTACACGAAGAGGGGAGAGAAGAAAAGAAAATTCAAATGAGTTTGGTTGGATAGGTGGGTCCCATACAATGACATCATCTTTTAAGTGTATTTTAAGGGCAATTTTTCTTTGTATGTAGCATTTACAAAAGATTATAGATTGTACTCCATCCGTCCTTTAAAGTTTGGCATTTTCAGAAATACATGCAATTTTTAAATTGCATATATCTTTCAATCCATAATGTTTTAGGCGATTGTTGGAACTTTGTTTAATGGAACAAATTAAAATCTATCAAATAAGATTCATATTATTTATAAAAATATTACGGATTAAAAGATATAGTTAATTTTTTCATAGGGCTGGAATAGTGAAAGTGCCAAACTTTATGGGACGGAGAGAGTAAGATATAATCAATTAGAAAATGGCATACACTCCCAAAGAGGACCAACAAATTGGAACAAACAGTATAATTAATTAATCTGGTACTCTATTTAATCTGGTACCACGGACTATAAGAGATTCCTTATTTTAATAAAATATGGTTCTCCCATTTTCCGATCAAATTTCGATAATCCGAACTGTTCAATATATTTAGATTGTGATTTTAAGGGTTTCTCGCGAGAAATCAACAACAAAAAAAAAGAAAAGAAAAGAAAAGAAAAGAACGGAAAGTGCTTCATCCGAGCAGTTTTCATTGAATGGTTTAATGAAAAACTGCTAAATGGAAGGTCCGCATTTTATTAAAATAGGAAATCCCTTATGAGATACATACATACCATGAGCTTCTAGGACCTTAAATATGGACCTCATATTGTCTTTTTGTTCATAGAATGGAAACGCTAGCGTGCATTGAATGGAAAAACTTTGGAACAATGCTAATACAATCTAAGCCATTGTTTTTTATGTTAAATTGCGTGCGGTAGTTTGTATTAGTAAACAGAAAAGTCTAGTAACACCGTATTTTGAACTTTAGACTCAAGTGTACGTTGTGGTCTCCACACGGGTGTTTGTGTAGAGTCTACTTCACTTATGTCTATAGAGTAAAAAACGCAGTGTCAGTAACATTTGGTGAATCCATTACAATATCTAAGGTGTTAAATATACTGCCCTTTCAAAACGAACGTCCAAAAGGCCCAAATGCACTGCACGAATTTCACATTAGCAATATCCAATTTGCATCCAAAAATTTGTCGTACCGGGAACACGTCTACCACACAAGTCCCCCAAACCCTTATCGCCCTGGCCTATTTAGTCCGGGTACAAATGCCTTAGAATTACTAGTATCAGTTTAGCTCAACTCATTCGTCCTCTTCCCTTCTCTCTCTAAGAAAGCTAGTGATAATAATAAACAAGAAGCAGCTAACC

>RsbHLH062

CAAACGTCTCGCCTAATAATTTGGAAAGAAGCTACAAGAAAATTAAAACTTAATTATTATTAGGGCATTTCAAATCAAGAGCCTTTTTTTTTCAAAACAAAATCGTTATTTTATTGAACTCTCGAAGATTTCTAGTTATTTTGATTTTGCTTATAACGTAAAAGTGGTTTAAAAACTTAATCAGTTTCCGGTACTTATTTTTCAGTTTTCAGCTTTATCAAACAGAGTCTTCCTGTTTGGCCTTTTCTCTTCCATCCTAATTACTTAATCATAATCTGAATTAACTAATTGATCGATTTTGACAGTCATACATTCGGAGCACGATTATTTTACTCATCGTTCAATTTTTTCAAGACAAATAGTCTTTAATCATGTGACTCTCAAAAATTGATTGTGGTTTTCTCCCCCCCCCACCCCCCCCATGGACAGTAAACTTTGACAAGTGCAACTCAATAAAATTTCAATAATTACGAGACCCCGTATTGGTACTTGAATTCCCATTGCTTTTTCGAAGCCGGAGGCTATAGAATTTTACTCTCTTGTGGTTTGGCGACGATGAAGAGATTGATTCGGTCTCTTTGTTAACGTTTGAGGACGCGAGGCATGCCCATCTATTCTAGCCCGTCGGCTTCTTGTCATCATGTTAATTTCAGCGTTTGCCAGTTGTTGGGTCCATTTAGCATCTCTTATATAGACAGACCCCCAAATAAAAATGGAATTATGGAATTTTATCTTCATAAGAATCCATCCTTTTGACCTTTCACATCTGTCATTGATCGTACTTGATGCAAATGCAAATAATAAGGAAACAGGTAATTTAACATTTGTTTGGGAAAACACTATTTATCAAGGAGCTACGAAGTTTCCTGCAAGAAAAATCTGTCATAAGTTGTTAGTTTGATCAATAATTCCTCAGCGAGGAGTGCTAGGTACAAAGAAAATATGCAAAGAAAGAACCCTTAAAGTATACATGAACGGTCCAGATTCACTGTGCAGTGCATCTGAGCCGTTTAGCTACACTTTAAGGGTCGTTTCTTTGCATATTTTCTTTGCACTTAGTATTTCTCTTCCTCAGCTAACAATTGATTCGTATTCTCACGTGCAAAGTTGCTATAAAACACATGTGGTTGGAGGCAACTTGGGTTTTTCCGTCCCACGAGGTCTTTGACCAAACGACATTAGAATAGAAGTGAGCGCAGTTAAGTGACAAGAGTTCAACTCCTCTTCTAAAACGTTAATCTAACAATTTCAACAATACTGACTTTATCTACCAAACAACATTAGGATAGAGGTGCGCACAGTTAACTAACAGGAGTTCAACTCCACTTCTAAAACGTTAATCTAACAATTTCAACAATACTGACTTTGTTTACGGGAAAAATGATTTTTCTGCTTAATTGTATGCTTCTTTAAAAGGAAAAAAAAAAGAAACCAATCCTTTTACTCTTCGCTGTTTCCATATTTGTGTCTCTTTCCATCCAAGAGAGAGAGAGAGAGAAA

>RsbHLH063

ATCAATCCCGCTTACATATGTGTATGTATGTCATATATATTGAGTATGTTACAATGCGCAAGAATCAAATAAATACTAACTATGAAGTTCACTATATTCATTCAACAAGAAAAATCACATGTACCACCACATAAACATGGAGTCTAAACAAATATCAAATGACTTTCACGGCAACTGGAGAGTCTACGCACGTACAAGAGTTAGTCTCAAGTTTCGAAACAACATAACCGACAGCCATCACGAACTGTCCATATTTTAGATTCCAAATTGTTTTCCACTCAATGTGCAGGGATGACAACAGATAGGTTCGATTCACATTCTGTGATTACCGGTTACCATACCTAGAATCAGAATGATAATCGAAAAATATGATCTTTAGTACTGTATCATAATCAAATAGTACGGGGTAGATTAATTTTTAACCAAACGCACCGTAGTGTGACATTAAGGGTTAGAATATGCTGCTTGTATATATGTAGCATTCAATCCTCATATGATTTTTTTGTACCGAGCATTCTTAAAATTTTCTGTTTATTTATCTCCACTCATTATCATCATTGAAAACCTTATACATGTAACCATGCACAAATCACAATTATGATATAGTCTCTACTTATGGGGAATGCAGCTTTATATCTAAGGGGCATAAATGAAATTTAACCCCCACATCTTTTCTTTCTTTTCCCTTTGGGCCTTGGGGTGAGTATTTACTCCAAACTCAGTTTCCCTACCAAACAAAACAATGATTTATTTAATTTGTCTGTGTTATTTGACTTTATGTGTGCAATATGCATGTCTTATTTAATTGTATTCAATGCATATGGTAAGTTGTAATCTCTTTTGTTAACGAAGTTCTAATTGTTCCACTTAAAAACCATGCGTTGATATTCATCACGCTTTGTTTTTATTTTCTCTTACTTTAATTGGGGTCTTAAATATGGTATTTTTTCTTTAAAGACTAATTGAGGTGCGCTTGGCCCGACACTAGGAAGAAAAATATTCATCACACCAGATAGCAGTAGAGGTGTCAAACGTGCCGTGCCTGCCCAACTCATTTAGTTTCATGCTTACCGTGCTTCATACGTGCCCTGTGCCTGCACCGTGTCGTGCCCGCCTAAAATCGTGTCGTGTCCGGCCCACTTCTGCGCCATGCTCATGTCGTGCTCATCTAAGATCATGTCGTGCCCGTGCTAGTCCGTCTAAAACCGTACTCATGCCATAGCGTGCTACAATTAAATGTGTCATGCCCGTGCCAGCCCGACCTATTTGACAGCTCGGCATGGTAGTGTTCCAGTTAATTTTCCCAAACGACAACTTAAAATATCAGCAGGGGTCCTAGAGGAGAACCAAGTGGCTCCATTTTAACTCCATGCTAGTAGTATTTGAGTTTTCATTTTCTTCTTCAAAATTCGAACTCCAATCACTCTGCCCATTCTTTATATATCTGTTCTCAACTTGGTACCACAATAAACAGCAACCTCACATCAGCAGAGGAGAGAC

>RsbHLH064

ATTTTCATTATGATTCATAACATTTTTGTACATCTTGTAATTTTTATACTAAAAAATTATAACTTTTTAACAATACAATTCGTAACATTTAGGATTTGTAACTTTTTACCAATACGATTCATATTCTCTATGAATCATAACATATTGGGGGTGCATATGATACATACTCAAATAAGGGATGTATAATGTAGTCTCTTTTGGCGTTTTTTCACTTCAAGACCATTTCCATAGAGTACGTTAAGATTCCGACCAATTGGATGTTTGTATAGTAGCTGAACCAGTTGCGAAACAAAATGAATTTTAACACGTAAAGCCGCTAAATTAATTTGTCTTAAAAACTGTTGGTATCATTTACTTTTCAGACATCTTTTGTTTAGTTTGTCGTCACAATGTTTGGAATCACTCATTCGTTTCAAAGGAAAATTTGGTTATGCTTATAATGGTTTTGTTATGCCAAAAGTTACGGTATCCCCAAAATGACCGGGGACACCGAAGTCATTCCCTTTTACCAATATGATTCATCTCTATGAATCAAAACACTTTGGGGGTGCATATGATACATACTCAAATAAGGGGTGTATATTGTAGTCTCTTTTGGCGTTTTTTCACTTCAAGACTATTTCCATAGAGTACGTTAAGATTCCGACCAATTGGATGTTTGTATTGTAGCTGAACCAGTTGTGAAACAAAATGAATTTTAACACGTAAAGCCGCTAAATTAATTTGTCTTAAAAACTGTTGGTATCATTCACTTTTCAGACATCTTTTGTTTAGTTTGTCGTCATAATGTTTGGAATCACTCATTCGTTTCAAAGGAAAATTTGCATCCATAAGAAAATTGCCTAATTTTTGGGAGCTCTTGACAAAACGGCTTTCGAATAGAAACTTGGCAAACCATTGGAGATGCTATTATTGCATAGTTGCACCCATTGGAAATTTAGTCGTTGAATGGTGGTTTGACATTGTTTTCTCTATGGGAATCAAACCAAAAAAAGTGAACCTGCTCCTCTCCGGGGAGCTTGTTCGTGCCATTAACTAATGCCCCCTTGTGAATAAGATGCCCTTGTTATCTTATTCATCTCCTTTATCTTCTGAAAGGGTTCCCTTTCCTGTCTCCGTGAAGCTTTTCCACCTTTTAGTTTTTCAATAACTGGGTGTTTGGGGCATGGGGTTTTTGCTAAGCAGGTGTTAACCGGCTGGATCCGCCAGTTAGATTAAATTACTGTTACATTGGATTCTAATTACTTAGCCTTTGCCGGTTTGAGGGTGCTGCTAACTGATTCATTTCACTTTTCTTTAGAGCACTCTTCTCTTGTTTCTATAAGGCTGCTAAAGTTACTTGTTCCTTGCTTACTCCTGCCAAAAAAATTCTTTACCAGCTAACATTGTCGATACCCCTTCAATAAATAAACTAACAATTTGTTTCTGTACCTTGCGAACTCAAAACAGTTAACTTAAAACCTCCACTGGTTTCTGCACTCTGGGTAAATTCATCGGAAA

>RsbHLH065

ATTGTTTGGCCTTTGTGGAATTCGAACATGAGATCTTATGGAGAACAATCACTTATTTGTTTTCTCATGCCTACTTGGCCAAACCCCTTGGGATTACATAAGTGATGATTTAGAAAAATCAAGAAGGGACATATGGTCTGGCTTAATTCTGCAAATTTTTGTCCCCTACAGAAATTCTTCTCTTATATTTCCCACTTTGTGTAGAACACTTTGGAGCTAAGGCCAAAAGAATACCAGCATTCTCAGGGGCACGCACAGGGAACCAGCAAGCCCTTTGTCATCTTTTGTCACCAAACTCTGCTCCAATTTTCTTATCTTTTGGTCAAAAGAAAGAAATGGGGAAAAGGAAGCTTTCACATGTGACACATGTCGGTGAAATGGCCAAAGTTTTTTTTGTTTGATGCAGAACAGTGGTGAAAATGAAACATGCAGTATTGCAGTGCGAGTGTTGCATGGGTTAAAGGTAAGCTATGTACGTGGAGTTCAAATCTCTATGCAAATTGGTTAATTCTGCATGTTTTCATGACATTTATGTTAACAAGCTGAATCGATATGACACGGGATTAATCGAGATGTACATGAGTTAGTCAGGAAGAGTTATAGAAAAATCAACATGCTAAGTCGCTATGCCGCTAGCCCACCACCAAATAAGATTTCATTGGGTTGAAAAATCCAGAGGGGTTAGCTAAGTGGTTAAGATCGGAGATTTAGGGGATTTTGCTCTCCTATGGTATCAGATTAGAAACTTCTCAAGTGTTATTGACTCTTTGGGGCCAATTCATAAAAAGCTTTGTTCTAAACTTAATCGAAACCTCATTACGTGGGCAGTGAAATTGGTCTCTCAAAATTAGTCGGGGTGCACGTAAGCTGGAAACCTGAATTATAAAGCATTTCTCTTGGAGCACCGAATAATCTTTGGTCAAGGAGGAGACAAAGCTGCTTTTTTTCCTTCGGATCCCCCAAGAAAAAGGTATTGCCAATGCCGTAATCAACTGCGCCGGAAAATGATTGCAATATTTTTTTTCGCACTTTTTCACACCAAAGTTTACTCCAGAACACAACAGCTTGTTGGAAAAATATTCCTTTATGTCCCCTGTCATCCCTTTCTTTTTTGTCTGGGTATCGTGATTGGTCTTATTTAAATCGGGGACTTTATCGTCATGTTTTGACGAAGAGTGTTTTGGTCAGTTTTATTTGCACATTAGCTAATTTTTTAGCTATCGATACTAAAGCTCCATGCAAAATTGACTCTGTAAGAATTGATAGCAGTAAATATTGCCTCAGGTCCGGTAATAGAGATCGCCGAAGCTCATATTTCAACAACTATTTAATTGGCTTTTAACAACAAATATACTCGAGATTTTATCATCCAGAGGCTATTAAAGCAAAATCGTACCCAGGTACTATTTCACTTTTACTAGTATTAAGTTTTGTAATCTCTCAATATTCTAAGATCTCTCTCTCTCTCTCTCTCTCTCTCTCTCTCTCTCAGGTGGACAA

>RsbHLH066

TTTTAGTTTTTGGATTCATCTTGACGAGACGCGTGAATTGAAAAACAAAAAAGTGAGGTACAAAACTAACAGATGCAAAAAAATTTCAAATTATCGGCGAATGAAAAACTCAAACACACAACGTGAGTTATCACTTATCACTCATTCACCTAATAAAAGACCAAGACCTTGTTCCACCAAGACTTTTAGAACTTTTGGAAAAAGTTCTGATTTTGTTCTTATTTAATTTTTTTTTTCATATTTGTTAGTTTTGCGCCAAACTTTTGTGATTTATTAATTTTGGAAAAGTAAAAAAATTTACTTTTACCCAAATATTTCGAAAAATAACCACTTTTAAGTATAAAAAAAAAAGTCGATTTTTTTTGTTGTTGCTAAAATGTGGTTAATTCCCAAAATATTTTGGTAAAAGTAAAATCTTTTAGTTTTCGGATTCATTTTGACGAGACGAATCGAAAAACAAAAAAAGTGAGGCACAAAACTAACAGATACGAAAAAATTCAAATAAGGACAATCAAAAAATTAAAAAAAATTGTTAGTGGAATGAAGTCTAAAAGGCATATCCTCATTTCCATCCGTCAAATTTATCAATACGTGATCTGTGGTGAACACCGCATGGACAACACCATGTGATTAATACTCCTGCAATTTTTGAATAAGGCCCCGTTTCCACCAACAATTTTGCACTACATGTGTTTTAGGTTCTTTTGTTAACTTGTTCATATATACTCACCCACAACTTCTTTCTTTCCATTTTCCCCTCTATTTTGACCCACTCATATACCTAGGGCCGGCTCTGAGCCGAGACGAACAAGGCGGCGGACTAAAGCTCCTGAATTTTGGATCAAAACTAAGGCCCCAAATAGGTATAGCCAGATACGTAATATATATTTATTCTAGCACTTATATACTCACAACTAAACTAGCTAGCCTAGTGGAAAAGTTTAGGTTCTTTATTCAAGAGGTACATAGTTCAAGTCTTAAGGGTTTTATTTTTCCTACCAAATATGAATATTTTTTTTCCTTATAAATTCGTCTTAGGCTCCGAAAATCTTAAAGCCAACCTTGCATATACCATCTACAACTTACAAGTTAACATTTTTAGATTACAATAAATAGTTCATAACTTTTTGGTTAGCTGCAACCTAAGTACTGTCCCAAACCTCTACATACATGTGTAACGTGTTTTCTTATACATACAAACGTACATTTAGATATAGATATAGAGAGAGGAGAGAGAGAGGGAGAGAAGGGGTTTGATGAGACCAGTCAAATGTAAAGGAAACTGCATGGTTTTGAGTTAAAAAAATTTTTTGGCAAAATTTTGGAGGTGTCCTCTCTATTCTCATGTATATATATAACCTGACCTCATAAGATTGTATCAGACCCACTTTTCCCCCTACTTGACTACTACCTCTCTGGAACCAACAGCAGTACGCCCCTGCTCTCCTCTCTTCCATCACTTTCCATTTTCAATTTCAAAGCTCTCAAAAATATAAAAAA

>RsbHLH067

ATATTACTAATATAAAGAAAATTGATTTCACACTTTCCTAATTGATGCAAACACTTTGTTTTTCATTTTTTTTAGCAGGTGAATTTACCTAACAGTTCCAGGGACCGTTCCGTAAATTTACTCGCTAAAAGACAAAAAAGAAGAAGGAGTGTTTGAATTAATTAAAAGAGTGTAGAAATCGATTTCCTAAATATAAATTCCCCATTTCACTCATACCATTGGGGCGTATTCGCAAATACACCACTTCATCTGACAAACATTGCTCAAAATCTCTTGAGCATCTCCAAAGAAATAGAGACTCAAAATAGCAATTTTTTTAATTTATAAAAGTGCATATAGAGAATTTTATATCACTATCTCATTTTTACCCACAAATCACCATTTTTTATTTGTACTTTCAAAAGCTCGACCATTTTCAACTTCTCCAAAAACTTTCACGTTCCTTCAAAGTTGTACTACTATGATGTCCATTCTTTTTCATGCAGATGCAGAGACTCTACTTGTGATATCTATTCTAGAAATCACATATTGTGTGAGATAAGTTTTTCTCTACTTGTGCCTTGGTGGGATAGAAACAGTGGCGGAGAGAGATAAGGACCAATGGGTTCCCGTTGGATTTTGGGTTTACTTTTCAATTGTAATCTTTAAAATTTTAGCATAGCACCTCCACTTTGACATATGTATAGCTAAACACTTTTTTGATAGAAAACTTTGAAGAAATAATTCAAAAGAAAATCAAGGTGTGTCAAATCTTTAAAAAGGAGATATACACAACAACAACAACAACAACAAAAAAACACTCAACAAATTGATAGTTTAGACTTTGAAGAAATAGTCCAAAATCGATACTTTTTTAATAGAGACTTTGAAGAAATAGTTTAGTACGAATTTCTGGCTATGCCACCGGCACCGGGTGGACTTCTTCTTTCCAAGATATGTTGCGTTGAGAAATTCTATGGTCACGTCTAGTGAAGGCAACTACGCAGGTCACTCCTTCATTCCTTAAAAAAAAAAAAAAAAAGGTTCCATCTCTAATAATGAAGTGAATGGTTGCAAAGTCGAATACTCAAATATCGAGAAAAGCCGAACACAATAAACCTTGAGTTACTGGAGATTTTTCCGATCATTAGTTCTGGAGCTCCGGTATTAATTGAGATACGCACAACCTAACGTGTACACATGATTGGCCGGAGGCGGTTAAGTAAGAAAACAAGATAGTAATAACACTAATATAGTGAATGATTAAGGGGACACATACATGCATCATGACACATACATTCATGCAATCATGACATAACAGCCTTGCACAAACGTAACGTTTATAGCCAGAGGCCACAACGCGTCACATTTGCCTCAAAACCATAACAAAGTCTGAAAAAAGCCACTTCCCTTTTCCTCCTGCAACGTCCTCTTCTCCCTTCCCTTCCGGCTTCCATTACTATAAAAACAAACATCCCAATCTCTCTCTCTCTCTCTCTGTCCTCTCTCTCAAAGCAAAAG

>RsbHLH068

GCCATAGTTGAATCAGCCTCATTGGTCGAGTATTGACAATGGATTAAGTAGGCGTCATTTAATTCTACCCCCCCCTTGAAATCCCCGGCCAAATTAATTTTCCGGGTTACCCCGGCCCACCCTTTTCCTCCTTCCTTTTCCTTTTTTCCCCCCCTTCTTTACATTTCTCTTCTTTTTTTTTTTTCCTTTCCGTTTCCTTTTTCATTTACCTTTCTTTTTTTTAAGTTTGAAATTTTTTCCTTAATAATTGGTTTTTCTAAAATTTTGGCTTTGAAAGTAATTAAAGTGTTTCTTTCTTTTTCCTTTTTTTTTTTTTTTTGGCCCTTTAAGAAAAAAAAAACAAAATGTGTTTCAATTTCAATCCTTTGGAAACCGTAGGGTATTATTTTTAATTTCCCCAAATTCTTAATAAATTTTGGTTCAACCTTTAATTAAACCCAAGAGGGTTTAAACAAATAATATTTAAGGGTTTTTTAAGCCTTTAAACCAAAAATTCCTTATCACATTAAATAAAATATAAAAAACTCTTTAACCTGATTAACGAATTGCAATTAAGCATTAATTTTGAAATATTTTTAAATTCAAAGGGGATTTTAAGGGATTAAATTAAATTAACAATTAAATTAAATTAAAAATGGGGGCCCAACCGCCCCCCATTGGAAACCCCTACGAAGTTAAAACGGTTTTATTAAAAAAATAGAAAACCCCTTTCAAATGGGTTAAATGATTTGAAGCAACTATACTTGTTGGAAACAAAAATTTAACAAAATTTTTGTCATAGTTAAAAAAAGTTTAAATATAAATTTGTATTTGGATTGTTTTTTGAAAAAAACGTAAAGCCGAAAAATTGTAACTTAAGAATTTTTGACAGAGAGAAAAAAAATTCAAACTGGAAAAAAAGAAAAGGAAATAAAATGAAAAAAAAAAGCCCAAACCGGAAAAAAAAGGAAAAAAAAAAAGAAAGAAAGAGCAAACTGTAAAGAAAGAAAGGAGAGAGAGAGAGAGAGAGAGAGAGAGAGAGAGAGAGAGAGCAGGGGGGGCGGGGGTAATCCTGGAAATGGTGGTGGGGCCGGGGAGGGGGGGTGTGTCGGGGGGGGGGGGGGAGATTAGCAATTTACTTGGATTAAGTATGCATCATTTAATTCTGAACACAATAGTACGCTATTGAATTTGAGTACCAACGGATACAAATGTAATTTTCTGTACCGACTACCATCGTATTGATCAGAGTCGACTGACACGGATGCAATTTTCGATTGGTAATAATATCACAAACCCCCGGTCAATAACATACCTAACAATTGCTATTTAAAACCTTGCAATTCATACCAGAAATACAAACCAACCCCATCACCCTTAAGCACTATATAAGTTTGAAACTCTACAAATAGGCCTTCATCAGCAGTTGCTTTTCTCCATAAAGATTCTTGTCTTGTCGAAGTATACTTATAGTAGCTCTCTCTCTCTCTCTCTCTCTCTCTCTCTCTCTCTCTCTCTCAT

>RsbHLH069

ACTCCATAATCCATATCAAAGATTGATCTCGATTTTAAAGAAAAAAGGTTGATTCGGTAAGATTTTCAATGTAGTGAATTTATTTTATGTTGCACCTATTTTTTCACCCCAAATCATTTTTTTATCAACTTTATTTTTTTTTTTTCATACTCGTTCATGTTATCTTATATTCTTTGAAAATATATTTGTAGGGTTAACCAATAATCCTTGGCTCTTTGCTTGAAAAACTAGACGGACTGGACTGGACTGGACAAGCCCAAGTCCTATAGCAAACTGTGGCCAACTTCCTCAGCAAGAGCCCAAAACGAGATTTTTTGTTTTGGGCACAACAAAACTTTGATTATACGCAGAAATAAGGGAGTATTGATCTAATTAGCATCACATAATTTTCGAAAAGGGATCCGACCGCACTTACATCGGAGCCGAAACTAGAGTTTTTCACGGAGTACTACTATAGGGACTTGAACTTAAGAACCGACCGATGAATTTTAAGTACTTACCCATTAAACTAGCCGCTTGACAATACAAGAACCTATCAAAACTTGATAGTTGATTTCACGAATTGGGGTTTGTACATGACTTTTATTTGGGACATAGCTGTAGTTTTTCAAATGTTTGGCGAATCAATATTAAGCAAAATGTCGGGTTGGTTAGTGTACTTCACCCAAAGATAATATATACTAGCATTGACACTTCAATTTTCCACTCTCCTTTTATTTGAACACACTTTTCAATTTTTCTTATTCAAAATTACACCTATTATGTGCATAAAAATGTTGTTAATTTTTGTTATAATTTCACGTGAATAAAATTAAATAGAGCAGATTTAAATAAAAATGGTGCCAAAATCAATTCCAAAACTTTCCCAAAACCTTGCTACTCCAATAGTTTTTTCTTTTCGACGGTAAGTGGTGTCAAGACCAACTTACACAAACCTCGATTACTCTTCTTCTCCAGTCTAGTCGGTCATCCCCTCCGGGACTATGTGATTCACAAAAAAATTCTTTCTTTGTTTCTTGATCAATTGCCAAATTTCGAACATTTTCATGTTCGAGTTTACAGCCCATTCCGTTGTTGAGAGCCCCCTCGGGGTTTTGCTGGTCCAGTAGTTGCAAGTATTCCCTCCGTTTCTATTTAATTGTCTACTACAGTTATCTCTTGACCTATATTATGTTTTATGTTTTTGAAATCATTGTCTTATAGAAAAAATTGAGATCTATCAAATAAGATTTATATTGTATATTTTAGCAATATACGTTGAGCGATATAAACCGTTGAAAATACACGCAGAATCGTAATGGACAATTAAATAAGGACAGGGAGAATATTAAACTTGCTATACCCCAAGCCGACCACATAAATGAGAGTTACTGTACAACGCTTCGTTGCCATTTTGAGAAGGCACACAAAGAGAGGGAAGCAGAGAGAGAGAGGGAGAGGGCGAGCAAAGCGACGAAGAGGATCATCATCATCATCTCTGCAACGGACTAAGAAAGAAAG

>RsbHLH070

TATCCAACCAAAAACGTCATCCTGGAACTCAACGTGTGAGGGGATTTAACTCCAACAACTTTGGTAGTATTACACCCAAACACAATCTCATACTCACATGAGGGATGGGTTCTGCACATGTTAGAAATGTTAATTGTGCGCATGACCCATCTTTAATATAAGTATGAGTGTATAAGTAAGTACGTATTTGGGTGTGATTTTAGAATTATTGTAACTCATCGCTTGAGATATTGGAAATACAAACTTCACATAGCGGACAATATTTAATGCGCAAAAAGTGAACATTGGATAATGCACAAAAAGTGCATTAATAATTAAAGGTGAATAGGTATGCGAAAGTGCATTGATATCTATAGGAAGTAATCATATCAAACATATTTCGTCGAATTCTCTAGTGGTTAATTAACGACTAGAATGTAATTAGTTATGCGAGAAAGGAGCAATGTAATTCTACAAACGTGAGGGAGAAATTTGTAATATTTAGAAATTTCAAGGGTGATTTTAATAATTTACCCCAAAATGAAAACAAATACGTAACCAAGTAACATAACTTTAGAAACACAAATACAAGCACAACTGTGACTAGAGCCGTTGATACATTTTACGATTTTCAAGCACAATTGTGTTTTAAAATTGTGTCCGTTACGTTGTTAAAGAAAGGATTGTTTCACTTTCATAGGCTCACAAGCCACGACGGAGTTCATCTTGATAATTGAAGTTGCTCATCATGTAGCGCATGTCGAGACAATAGATCATGTCAGTCGGATATTAAATGGAATCTGATTCAAACACATTTTAGCAGAAGGAAAATGGGTTTCAATATGTTCGACCCACTGGATCAAAACTAAATTATGGAGTAGTATTTTCAAAGGAAACAGGTTTGGATCACACCTACACATTGTTATCCAGCGGACTGTGACTTTTTGCGTGATCCTAAGTCTTGGCGTGCTCTACAGGATCATGGATATGGGCTCGAAAGGTGCCCAAATTTGGTGGACCGGACCGGAGAGGAGACACCCGCCTTCGCAAATCACTCTCCCTGATTACGCGTGCCGTTTCGTTTCATTTAAAAACCCGCGCACAGTTTCCACTCACTTCAATGTCAGCTAAAGTACATGTTTTTTTTGCCCTTTCTTTTGAACGGCAGCTAAAGTACATGCTCTGTGTGTGTGAGAGAGAGAGAGAGAGAGAGAGAGAGAGAGAGAGAGAGAACGGGGTTCAGAAAGAAGAGACGCAACCAACTGGAAGAGAGAGAGAGAACGGGGTTCAGAAAGAAGAGACGCAACCAACTGGAAGACGTACCAAAAAACAGAAAATAAAAATCCACACTCAACAGACAGACATCATCAGTGTGGAAAAATTGGAAATGGGTTTTGGAGAGAGAGGAAACAAAGGCGACGCCGTCCCTGTGTTTCCTCGTAATTTCCTCAGTACTCTCAAGCTCTAGTCCGGTTAGCGTTGTTCTTCAGGCTACGTTTGCTTGAGTGTCTGTCAGTTATA

>RsbHLH071

GATGAGGAATTTGTCTCCAGAACAACAGATGCTACTTCTCATATAGGTTTTCGCCAGGGCCCACGCTTTTTATCTACCGATTATTCGCACAGACTCGTGTATTTACGTGCTATATAGGGTACTGATTTTTCTAACTTTGTACCGAATTTAATATGCGATCGACAGCAAAAGCAGATGGAAAAGGCACTGGTCAGAAGGGCAACGCGCCTCGGTCCAAGCATTCCGAGACGGAACAGCGTCGGAGGTGCAAAATTAACGAGAGGCACGTACGATACAGTCTTTTTTTCTGTTGTGCAATTTTGTTGATTGTGAGTGCTCTTCGGTTTATAATTTGTCCAAACGTTTTGTTTATTGAGAAGTTATATGCGTTTGTAAATTGGTAAGAATAGCAGCTATGGTTGTTGGAGCAGTATTTTTTTGATAACTCAAATGTCCGGGCCAACTTATGCTGCCCTCGACTAATCATTGGGGCCCAATCCCACCGCCCACTTGCGGGGAGCCCAATTAAAGCCGGAGCAATTACTCCATATGGCCCCAAAGGAGTTGATTGCACATGTGAGGTCTCGTACCTGAGAGCAAACCCTTCAATCCTAAGCCTTGACCACCAGGCCAACCCCTTGGGGTTTATGGATGTTGGTATAGTATGTTTACTGGTTACATGTTTGAGCTGTAGGATATGGTGTTCCTTTGTTGGAATATTCTTTAGCGATTTGCATACAATGATGGGTTTCATTGGCTGCTAGAAAACATGTACTTCCAGGGTGGACTAGTATTTACCTGCAATTCTTGACAAACTTGTAATCCCAAAATGTTGGGAAAGTTTGGGTTAGTCCTTTTGGAGATATACTAGAGTGTTCCTTGTTCGTCGGAATTTGGACTTCGATGGGGTGAGGTGTGTAAAGGATAGTGAGAGATTTGAAGAGAGGTAGTGATTTATGGGCTAGAGAGAAGGTGGCATAGGGAGGTGTATGGCCAAATGCCCCGATTTTGGGAAAAAAAAGTTATTGATGTCATGAACGTTCTATCACCATAACAAAATTATTTATTATGCAACAGAAGCCATTCTAAAATGGAATGGTTGAAGGACTACTATGATCCCACGATTTTGTGAGTTGATCTTCAAATTAATAGTTGCAGGGTGATTTACCAACTTTTTCTCTAGTGATTCTAGATGATTGGAATCAACATGACTCGTGAGGGTCAAATTACTTAAAGGCCTAACCCCTAAATTGGGTTCTAAAGCGCTTAGTTCTGTTATCAAAGGATGATGGAATGCGTTTTGCTCTATTTATGCAGTATTTGTAAACTTAGTTGGTTAGTTATCATTTATGGCCAGGTTTACACAAACCTTCACTTGAATTCTGTGTTGATCCCCTCTTTAGTAACTCTGGTGATTGTATCCTGCAAAGAATGATTGAATACATCTAGCTCTTTCTTGTGTGCAATATTTTTGTAAATTAGTTGTGCTCGGGGCTTATTAGTTTGATGTCCAGATTTCAG

>RsbHLH072

ACGTACATTGTACAGGGGGGGTCGGGTCAACGCGTAACTGTTGTGAAGTCAACCTTTGGTTTAACCATTTGTTTTGCAGCATTTTTAATGGGGTTTCCTTCTTTCTTTATTACCATTATTTTTTCAGGTTAATTATAGATGACTTTAACACAATTAAATGGAATCGGCTCCCTCCTAAAAAAAATTTAGACCAATCTAGTTTTTGATTTCATCAAGTGAGATTTAAATTGTTCATTGGTACGGCTTGTCGACAAGCTTATCACAACAAAAGTCATGTTAATCGAATGCTAATGAACAACTGATCGGTATATTTTAATTAACATGAAATAAATGAGTTTCGATTCCACATAGGAGTATCAATCTATTTTTTTCACAGCTGAGATGTGTACCAAAACAATATTGATCGACATTGGATTAAAACAGGATCATTTGACCTTCGCAATAGCTAGCCACAACACCCAACCACCACTAGCTTGACTGATCCACAATCCATTTAGTAACCATTATCCGATTACCAAGCCACCATTTTAATTAGGTTTAACAGTTTTATCGATCATATGTGATACTCCATATATTCATCTCTATTTGTTTGTCCATTTTGAGAAATCCAACTAACACAACATTACATGTTAAAGGTTTGAACTTTCCCAAAACATGCACTTCTATTTAGATTGGACTTGTTTCAATCCTTGAGAGAAGGGTAAAAGAGAAAACTCATACAAATTTGTTCTTTTATTTTTTAAAATGGATGGATACTTTTAAACATGACAAAGTAAAAAAGATGACAACAAATTGGGACAGAATGAGTATGTTATTTTATCATGGTGCCACAACTGGGAAAGGAAAAACAACATGGTTGGAAAGGCAACAAGAATGTAGCAGTGGTTTGCAGGTGAAGTTTGCATTTAATAGCAAAAGTGGTTTTGATTTCAATTATGTAATAACCCTAAATCCAAATTGACAACAATCCAAAGTGTTTGATAGACCATGGCCGGTAAAAGTCCTAGAGAATTTAAGTAGTGGGATATATGGAGGGTTGACATTGTCGTTAAATAAAGAGTCCTTGGATTTGAACTGCAAAAACTACAAGCATGTGGAGTGATACCTGACAAAACTAAAATCTAGGGTTTAGTTTGTAATTGTCGCGAATATATATTTGCACGCCTCAATTTCTATGCGATCGGAATCACCACCAGCCCAAAATCTTAATTCGAACACATACAGACCCTCTCTCTCTCTCTCTCTCTCTCTCTCTCTCTCTCAATTTTCTGGAGGAAGTGAATGCGCCTCCGATCCTCAGGGTGGAGAACTTGTCTGAAATGCTCTGTCTTTCATTTTACATGCCACGGATCTGAAGAGCGAACGTTCCAATTCCCCTTCAAGTTTTCGAATTGCGAGAAGAGTTCGGAAACTCGAAGCAGTTGTTTATCTGGAAAATTCGGACGCTGGATCTGATTCGAGATATCGTAAAACAATTGCGTGGGAAATAGCGTGCTGTTG

>RsbHLH073

CAACTGTTCTAAAAACAGACTTGCGAATGCGATATCGAATGTCATAAAAGACTTAATTGAACTCTCCCTAACACCATTTTGATTTTAGAAAAGATGGTGGAAAAACGATCCGACATTATGAATTATATAAAATGGTAAGAAAAAAGTAACATTGCTATCTCAGACCGTGCATACCGTAGACTCCAAAAAAGAAATTAATGTTTACTGTGGCAGTTACATGCAAACTCTATAAAAGGAGTGGGAACCCATAGCCTGCCTTAGAAACAAAGAGAGCTCAGGTGTGCTATCAGTCCAATGTGAGCATTCCAATCCTCTCTATCAGTCATGGAGATATCTTTATTAATGACTAGTATTATTCCTTAGCTCTCTCGAATCCTATAAATGAACCTTTTTCCATTACTCTTTGTATGTTTGAAAACCATTTCTAGAATTAGTTACACAGATAGCTCAAGGCTTGGTTTTAATGCCCATTATCCTGTCATTTTCATTGCCCCTCGTGCAGAAGTTAAGAAGAAGAAGAAGAAGAAGAAGAGCTCATGGACTCGCCGATGCCGTGGCCATCTGAGCTGGTACGAAACTGATCTTGCCCATGGAAGAGTTCATTATAAGAAGAATACTTATGATATATACAAAGTGATGAAATGCACTGGATAATCCAGTGGGGATTTACGTTCTTTTGTAGCCAACTAGCCATATAACCTACCTAGATTGTCGTTAATCAAGTCCTTCAGGGATTCTCCTTTCCTGAATATTCGTGTCTTTCTATATTCTTGTTTGTTTTTTTGTTTTTTTTTTTTAATTATAAACACAGACAAAGTTTCATGGACAAGGAGAAGCTACACAAAGCAAAATATATGGCCTACACATTGTCACTGCTCATGGAGCGTTGCTACTTGGTGCTTCAAAAAGATCTTCTTTATCACACATTAACGTGCGGGCCACACACTTGATAACGGGACCTATACAAATATTTGTGCAAAAGGATTTAGAGCACCATGTGACAGTACTCCGGAACAAATAAAAGGACGCTCTCTCCTTAGCTCTCTATCCTACATGAAATTTCCTCAATATTTCCATTTTTTTATTCATTATTACACACTTTTTACTAGATATAAAATATATACTTTATTTTGCTTGAAAGCGAAACTACTATGTTCGATATTTTGCATAATACCTATCAGTTTTTTTTCTATTTTTGTTTTTTAAATTGTTGTGAAATTTACGTAATATTTTTGTGAGAAAAGAGAGTTAATTCCAAGCATTATTCATGCCGACCCACTGATGCACAGGACAGCAAGGAACAAATTCTCACTGATGTTTTTTTTTTTTTTTTTTTACCCCATGCATCGAATGGGCAAGACGCTAGTATGCTTTATACTCATCATATAAATAATACAGTAATTTACCCATCACTCTTTCAGTTTGACCTTTTATATGAGATAGTAGTTAAAAATCAAATACAATAATGCAAATGCTAACCACTGTCTAGTAAAAAAAATT

>RsbHLH074

CCCTATTTCGTGTTACGGTGGAATTATACCAAAAAGGGAACAACTGGCCATATTGTTGAACCGACATTGCATATAGTATAAACTATAAAGGAAAAAAAATCTTTATCGAGAAGCGCATAAGTTGATCCGAACATTTGAATTATAGAAAAAAAAAATTGTACAAGCAAAGACCAAAGGGTAGAGAGTGGAAAATATGAAAACAGATTATGGTTATGATTAAAACTATGATTAAGGGAACTCTTTTTGAAAAAGGGGATTGAATATTGATACTGGTAAAGATTCTCACTCTTACTGCAATTCCCCTGAAACTTTACAGAGCGCATGGAAGGGCATGAGCAACCCCAAAAGTGGAAGTCAGAGCTTTCCCCACCCAATAAAAATCCACAAAAAAAAAGTACGAAAATATGCATATAAAAACCAATCAAATGACAAAATAGTTGGTTATTTAAGATTGAAAAAATGCTTCTTGGAAATCTCTCATGCTCCCCATCTGCAGAAATATACTCTCACAGCTCTCACAGCCCCACCAGACTGACCCTTTTTTTTCCCAAAGCCCATTATTATATTTTATCAAGTTCCACTTTTTTCCTGGCATTTTATTGTGACCTAACAGAGTACCATTCGTTCTTCCTTTCTGTATGTGTTCCAATTTATCATCCAACCAACATGTACAATACCAAGAATCAAGATTTGGCCCTCTCCATTGTGTGAAAAAAAGGTTCCTTTTCTTTTCTGCTGTTCTTGGCTTTTGCTGTCTTTTGATTGTGTGAGTTCTTGCAGCAGTTTCGCAATGGGTTTGGGGGTTGTTTGTTTGTGTTAGAATATTGGACAAGTTTTAGTTTTATTTTTTATTTTATGGGTAAGTTGCACAAGTTTTAGTTGCCAAAACTGACGTTTGCAATGGCTGTTTTTGGAGGTGAGATGAAGGAATTAATTAAAGAAGTTTGTTACAAGAGCTGGCACCAGATTGTAGCTAGCACACAAGCTGATGTAAAAGCTGAGGTTTGATCCAACTTTTATTCTGTTAGCTGCAAAATAAAAAGTCCAAGACTCATAATTCTTGCACTCTGATTGGCTCCTTTTCGCGGTTTTTCTCCTCGGGTGTTAGCTGGATATTTCGCTAAGGTATGTGTAGAAAATTTCAGCTCTTCCTTGATATTATAGTTTTTTTCATGGCCTCTGCTTTTTTTTCTTTTGGTTACTGTTTTTGAAACTTCATTGTCAAATTATTTTAGAACATAACCCATGAAAGCGATGAGTTTTTCGCATTTCGAAAGTAACCACCTTCGAAGTACTTTTTTTTTTTTTCCCCTTTACAAGTATTTTCAAGTTGTTGAAACCGATGAGTCTCCAGTGTCTTGGAAAACAATTTTCTGGAAATTGTTCTGCAAAACTGACTCCGAAAATAGCAATTTGCAATGGATTAATGATTTTATCTTAGGGTGGTTTCTACTTGAATTCTACAGGTGATTCATTTTTCAAAACTCCAGCAATCCATTA

>RsbHLH075

TATTTCTGGTCATATTCCAGAGCCTAGATGATGTAAAGCCAAGCATTGGACAAGAGGTACAACTGGCGTACGACCTCTCACAAATGGCGTACGGCAATCATACCAAGGTCCAGTGGCTGGCCCTTGCATCATAACTCAATCCTGGCCTCTTTTTATCTCCACACTCTGTTTATGGGGTTTCCTGTCCATTTTCATGGCTTCAAGCCATTTCATATGTCTGTAATCTATATATATGCTGCTATTAAACTGCGTAATTTGTCCTGAGTGTGCACTGATCCATTTCAGAGCCCAGAGGGTTGCAAACAAGGACTGTGAATGCATAAGAATGGAGTACGCCAATTGTCAGGTGGCGTGCGCAGGTCATGTTGGTATGCAGTGGCCTGACCAGTGCATACCGATGTGTTGCCTGTTCACATCTCTGAAAGACAACGTACCCCATCTTGTTCAAGCTGTTCACAGTGCTTGTTCTCAATCTACGTTCATTTATTGATTCGAGCAAATCATCGTCCATAAATATTTTATCACATAACTGCAACTTGTACAATTTTAGTCCCCTTTAACTGCTCCCCCGTCCTAACTGGCTAAAAAATGATCAATGAGAGAAAAATGCAAACATTTTACAAAAATGCCTGAATAGAGACCGATCTCCGGATTGGGCGAGAGGGGTGCCATAAAACCCTTCCCCTCTCGTAACCTGACTCCCGAACCTCACATATTGAAGATGACGATGGTCCAGTCTACAAGTCTTTTCAAATCAAACAAATATAGCAAGCGTTTTTGAATTCGGTTCCTTGGGTATTTTACCGATAAAACCCGAGTGGCGACTCTGAATCGAAACGTCTCGCCGCGCTTTTCAAAGAGGGCCGCACCCAAATTTTTCATAAATAAAAATTTTCCGGGGCGTGTGCCCACAATATCCCACGTGATATCCGTTCAGCATATTCTAGCCATCTAATACACTTTTGGACTGTTCCGATTGAAATTCTCTCTCTCTCTCTCTCTCTCTCTCTCTCTCTCTCTCTCTCTCTCTCTCTCTCTCTCTCTCTCAATTTGAGCCGTCCAAAAAAACAATGGATGGCTTGAATGTGGCGTGCAGACACCACGTAATACCCACCCGGCACTGGAATTTTTCCGAGCGATCCTCTCCCGATACCATGCGCAGCGAGTGCAGTGCGCACTCCCACTTGGCTCTCCCCCATCTCTGAAGCTTCACTTTCAGATCACTGTTTCTCTCTCTCTCTCTAACTTCGCTTTCACACGCTCTCTCTCTCTCTCTCTCTCTCGCAATCAGCTCCTTAAATAACACAGTAACCTCCTCACTCCCTCCTTAACACTCCCCCTAATAACTAACCAAAAATCTAGGGTTCCAATTCCTCCCAAGTCTTATCTCCTTCAGCTACAATACATACATTCACACAGACCCTACATACATATACATAATACTCACATGTGTGATTGTGTAAGCGAGAATTTCGAGTGAATTGTGAAGAATTCATTGAA

>RsbHLH076

AGAGAGAGAGAGAGGAGGGGCTTCAAAGGTGGAGAGATTTGAGAATATTGAAATTGAACATGGATCAAGACTGACGATCGAGAAAATTGAGCAGTGGGAATTGTAGTAAAAGATCTCATTACGAAAAATAACTTACGGAAGATTTAAAAATAAGTCATTTTTATCCGATAATAAAAAATGTTTTACATATAAAATATTTTTTATATTTTTTACACAACTAAATACCAAAAAATAAAAAAATATTTTATCCGAAAATATTTTACCCCTAAACAAAACAAATGGAGTTCAAAAATATTTTGGGCAAATTTAAACTGCTTCTATCATTTGTGTGGGATTTTGAGGCGGGTTTCACAAAACGCGGTGTTGGAATGGTGGTACCCATTTAAGCAGTCTTTTCTATTACCCTCCTCTCTCTCTCTCTTCTGTATTTAAGCACCAAGCAGTCTTTTCCATTGCCCTTTCTCTCTGTCTCTCTTCTGTACTGTGCTTCTGAAGCTCTTTCTGATCTGTTTCTCTCTCCATCTTTACTTCGAGCAGCTTAGAAAAAAAAGTTTTTCTCTTGGAGGGCATGAAACGTTGACAGTACACATCAGCTCGCTGTAACTGAGAAAGAGGAGAAGGGTTTGAACAAGAACCCCCCCCAAATTCAAGAAAAGTTCTCCTCAGTGAAAAAAGCTTCAAAATTGTTGACAGAGAGTACCAAAAGTAGTGCAGATTCTGAAATGGGTATCTTTCTCTTCCTACTTTTGGTACTCTTTCTCTTTCTCTCTCTCTCTCTGAGCTGGGGGGTCCGTTGCTCTGACTAGGGTTTTTGTTGCAGGGTTTGAATGGTCATTTATGATGCTGAAATCAATGATTTTCCCTTAAATTAAATGCTTGTGGACACTTTATGAGATCTTTTCTGAACTGGGTTTGTGTTATCCTTGTCATTTGGGGGTTTCCCCTTGTGTAATTTCTAAGGGTGCTACATAGCCTACTTGGATGACTGCAAAATTTTGTAAATATTAGTGGTCACATTCATCCTTCTGATATTTGGCACATATGTTGGCACTTGGTATTGGTTTGATACTTTCAATTTTGGGTCATTTTCCCTGAGGACTTCTTCCTTGCTGTTTCACTGTGTCTAGTGATGAATACAACTTGTTAAGGCATTTTTCAATGGTATTTTGAGCTTAGTAGGAAATGAAACTTTTGTCCACAGACACCTAACTTTCCACTTTGCAATGAATCTTTCTTTGTGAATCTGTAAGTTCTGGATTTCTTGATTGAAATTGGGTATGAAGTTATTGCTTTCGTTGGGTGTTGAAATATTGCTTATGATGTCTCTTTCACTTATTTTTTCCCTAGGTCTTTGATCATCTTTGTTGAGGCAATTAGGCAGTCGGGCAATTAGGCTTTGGAGGTCTCCAGTTTTGCTGTCTCAATTTTCATAGGTCTTCGGTTGTTCAAAGATAACTGTAAGATATTGTATCTTAAATTGGCTATTCTCGTTGAAAAGAA

>RsbHLH077

AAACCGGTTGAATAATTTCGATTCGATTCCTGTTTATAAAATTTTATAAACTGGAAAAATAATTTCGATCCGGTTTATATGTTTTAAAAACCAGTTGAAACCGGACTGGTTATATATATTATACATATATATATATATATATAAACATAGGGGAGGGGTTGAATTATGGATTTTTGGTTGATATTTTATAGAATATATTGGTCTAATAAATATATTTATCATATAAAAACTGTTTTTATGGGTTTAATTATGTTTTTATGAGTTTTTTTTTTATGTATTGGACTCAAAATTGATCCTTTTTTGTTGGGCCCAAAAACAATGGTTGAATTATAGATTTTTTGTTGATATTTTATAGGTTAAATTGTGAATTTTTTGATGTGTTGGGCCCAAAATATGGCTCATGAATAAAAACCGGATAAACCGGACCGGAACCAGTTGAGCCGGTCAAAATCGGAACCGGTTTATCTCAACCGGTTCTGGTTTCTAAAAATCTCACACCGGATAACCGGTTTCGACCGAAAAATACACCCAAACCGGTCGAAACCGGACCGGTAACACCCCTTAGCACAAGTGAAGGCGTCCCAAAGCAAGCCTCGCTCCTAACAAAGAGACACCCCATATTTGGAGATTAGGCTCCGTTCCAAAACACCTTCTTAAAAAATAAGTACTTATTTCACATTTTCAAACTCAAAAATAATGTAAATAAAAAATAATTTTTCAATTTTTTTTGCACCGTATTAAAGATCTCGATGAGATCTATCAAACAAAATCTATATTGATAGAAAAATTATTTGTGTAAATACATAATTTTTGAGTTTGAAATTGTCTTCTTAAAAAATAAGTACTTATTCATGTTTCTGGAACGGGATTATTTCCGCTCGATAGACGTATCAAAATTTAAAATTTTGGCTTATAAAATGGATGGTCATAGACAATTTAATTTGACAGGATCAAACCATCCATTTTATAAACTATACTTTTGATTAATCTATCTGTATCCAATCAAGTTAATTTTATGCATGCAAATACTACGTTATGAACCCTACAAAATAAACGTTTCAGATCGGTAATTAACGCACCAGAGGACCCAAAATAATGGTAGTGGGAAATCCATAGTTTCAGGAATTTAATCTCATTTAATTTTGTGCACGCCATATTTTATTGCACAATTTTTTTTTTCTTTTTTTAACTTTAATTGCACACGTATGTACGTAACACTGTTGTTTGCCAAAAGTAGGCCGGAGTAGTTACGGTGGTGGTGGTCGCCGATTAGAAGCCTGACTCCAACTTCCTCCAACCAATTCTCTCAATCATCGCAACAGGCGTCGAATCCATGTCTATTTCTCCAACGATCAAGGCACTTCTTTGAGGTAAAGGACAATCGATCTCTCCATTATTTCACCTGGGTTTGTTTCTCTTTCTTATAATCAATTGATTAATTTCGGAAATTCTTAAACAGGAGCAACCACATAATCCTCATACCCATTTGAAAATATCATC

>RsbHLH078

AGTAGTACCATTGATCATCTTCTTTCCTTTGGTCGTTGAGTGGAAAAGATTAAAATCACACCCAAAAAAAAATTGTATGAAAATGTCTCTCTGAAAAAAGGATGAAAACAGTAAATTCAAACACAAATCATTTGAACGTGAGAGCATTTACTTAACATCATCATGTAATAGGTTGAATGCAGTACCACTTCAACAATTCATAGCACTACTCATCCAATACTTATCTTTCTATTTTTCATGGTACTTCGGAATATCATACGGAGTAGCTTAAAACACAAAGAGGTTCCTCATTAGAAGTGTGTGCGAGTACAACAGAGAGTTACAAACCAAAACAACTAATTTAGTCTTGAATTTGCCTGTTATTAAGGCTCTCCCTAATCATAAACTCCCTTATGGAGATGGTAGTGTTCGTTGTGAGATAGCCTGGTTATAGTTATAGTTTTTTTTTTTTTAAATGTGCAGTGGAATGCGTTTCACTCTGACACTGTGAATGTTGATTTATTATAGGTTAACTAGAATGAGTTCTCTATTTGTGCGTGTCATTCTTGCGCAAGCACTATGCTAATCTTCTCTGAAAAAGGTTAACTAGCTAGAATGACAATGTGTACAAGGGGAGGTACAACATTAAAGATACTCCACTGAAGACTTTGACAGAAGCTTGGATGGCATTAGGAGAGTGGAAATATAGTTAATCCACCACCGTGGAGAAGTTTTTGGGGGGTATTCACTTTGTTTTGTCATTCAATAGTTTTTTTTTAAACGGCAAAAATATTGATATTGATAAACTCGATAACGGGTACATTGATGGAGAGAGAAGCAGAAGAGAAAAAAAAAAAAGCAAAGATGGCGGCCACATACCATGCCATGGAGCCACCACAGTGCTTTATAAGGTAGAGGACCCGTTATCGAGTTTATCAATAATAATATTTTTGTCACCACAGTCGTTCAGTACTTTGTTGGATGAATTTTTTTCCTTTTGCTCTTGGGTTTTATATATGATTGCTGGGATGTTCTATAGAATTTAATATAATGTTATTCATTTGTGCTAAAAGGGAAAAGAAAAAAAAAAAAAAAACCCAATACATTTCAAATCAGAAAATAGCCTAGAAACTTTGGATATGTACTTACCAAAAAAAAAACAAAAAACTTTGGATATGTAAAGATTTATCAGTTGCAGAGACGTACAGTGCCCAACAACAACAGGAAACCCTAAAAACCATATTTCTTGTTGGTCCCACCTTCCGACTATAATAATTCTCCACAAACCTTAGTGGAAAAATTCAGGCTGCATATTTTATTGTCTGCCCTATTATTGGTACAACCTTCAATGAAACGTCACCACCAACGGATATATAAGATATGTCCTCTTTAAATTCCCATCCCCACCACTAGCAACAAAGAAAATTATAAGACGAGAGAGAGAGAGAGAGATATCTAGCTAAAGAGACAGAGAGAGCCTCAACTGATCAACACCATTTTCAAATTTGCAACACCATTAGA

>RsbHLH079

AAAAAGATTTAAAAGTACGAAACAAACCTTTAGAAGATCTAGAGAGTGGTTGTATTTCATTTCTCTTTGTCATTTTCTATCCACTTTGATAAATCTCCATTTCTTATTACCAATAATATAGGAGTACTACATAAGGAGAAACATAGATAAAAGATGACATAAAATAATATTAAAGATTTAAAAGATAAGATGAACACTTTTCAACAACAAAAAAGAAAACAAATTAATAGTCAAAACTGAATTGTGGAATTAAATTTTAAGTGAATCGATCAGACAATTCACTGATTCACTTACAATTCATTTTTTATTTTTTTAGACAGCCTCTTCATATCAATTACCCATGGAATCGTACGATTCTAATAACTATGGTTCTCTGACGAAGGAAGATGGTGATGCTGGTGATCCAATGACGAATCAGTTGATAGGCTTGTTGGCCTCGTTGCAACAATTGTTTGGGTTTTTAATCAGTGTAAGTGTTTTTTTTTTTTTTTTTTTTATAGAATAAGAGTGCGAATGTGGTGGTATATTCTTTGGCTAGGAAAGGCGATTGTGATTTGTTTGTGACTTTCTAGTTCTTTGATTAGATGTTACTTCTAGGTGATTATTTTATGTATTCCTATTTTTTTGTCAATGAAACTATTAACGGGATAACTTGCGCACATTTTAACTAATTTCAAAATCTTTAAGATACGGACCGAACATAATCCCAAATACATTTCTTCGGAGCGGTTTTGGGGATAACTAATTAGACACCTAATTTCTCGGTCAATTAAAAACTGCTCAAAATAAGCACTTTTCGGTCATATTTTTTGCTAATTTCTCTCGGGTATTCTGAAAATCACATTCTGCACACATTGAATGGTTCAAATTATAAAAATTTAATTGTAAACTATAAGATTGAGATTTAGAGTATTTTTTAAATACTTTTTTGGGTGTACCTTGAACCGCTGCTAACATTTCTTTAATGCATATCCTCAGTTTTCACCCCATACTTCAACCAAAAAGCCCTTGGTGGTCCCAACCACCGGATATATCAAAATGAATGTGGACCCACCAAGTATTCACCACCACACAATATGGAACTGGCACGTGTCCGTCCCAAACTCTTACTTTTCTCCCAAAAATTTAATTCGACAAATGTAAAGTTGAAAGACCATAACCTAACCATCAAATCCGAACCCAAGATCTTTGGAGTAATTATTCGGTAGTAGCCCGGGAGTACCATCATGGTGTTCCGGATTACTTTAAAATTACAAATAGTTATATGGAGTGCAGACATTTAATAAAGGACTCCATAAGAATGTGTGCATAATTGTTAGTAGATGGTCCAAATACCAAGTAACGATTTTCTTATATTACCGAATAATTTCTCGGAAGATCTCTACGTGCATATAATAACCTAACCAACTTATTAGTCTCAGAACCCATTTGCGCCACTTACAAAAAACCCATTTGGGTAGGAGAGCAGCTTCTGAACGAGTGATCAACACGGCCTGATCC

>RsbHLH080

CTTGTGGTGCAACGCTGAAACCGGATTCAGGCCTGAATCAGCTTACTGTTTTGCTTATAAATAGCAGAATCGAAGATAGAAAATGGGTCCAACACCAATCAAAGCTAACGCTTACAAACACTTTCCTACTTTCAGCAAAGTAGCCTATCGAGTACTTCGTGCTCAAACCCTCATTCAAACTTACTTCATCAAGTAGTTTGGATTTTATTTTCCTTGTAATCAAACTTTATTATCACGGTGTAATAAAGTCTACGTTTTTCACGTTCCATTTCCTATACGACTAGGAAATATTAAGTCCACAACCGTGTGGCAAACCACGAACCTTCTTACGATCTTTAACCTTTGGGTCGTACACAATCGTTCTACGATAGAAGTCAAGATTCACGGGAATCTTCACAACAAGTTAGGCGCTACAAGTCTTGGCACGCCCGCGCTCCACGCATTCACACCCAGTGTGCCAAAAACGCGAGTTTTCAATAACATATTGTGAGTTTGAGATGATTTGGTTCAATTCAGTAATAAGAAGTGAAGTTACTATATATATGGTTTTCTCTTTAGTAACTAAACTCACTGGTACCAATTTCTTGTCTTTATGAAAGGATATTGAAAGGAATTGCGAAAATTTATATGAAGACGTACGTATAAAAGTACTTGAATTTTGACTAGACTAACCTAGCTACGACTATTTAGAGTTTTGTAAAAAGATTAACAAAGTCATTATACATAAAATATTCAAAGTCATACTTAATATCGCTATTAAACATAATATTTAGATAGGACCGGGCAGTGTCGGCTCAAAGATATTTAGGGCCTGAAGAGAAAGTCCTAACATGGGGCCATCTAGCTAGCTCTGCATTTTTGAACTAATTAAGTTAATTTGCAAAATGTTGAGGAAACTTTTCGAACCCAGCTCCGATCCTAAAGCATCCGAAGAACATAAGTCGCCACACCACTAATTAAGATGATGTTACTCATGAAAGGCAATTGAAACTCATTATTTAGAACTATAACTGTATTTTTTGGGGCCCTCTTGAGGGCAAAAATTCTTGGGGGCAAGAGGCGACAGCTTCCCCTCTAAGCTGGCTATGGGACCGGACTCCGTATTTTAGTTCTGCCTGCGTCAGAAGGTAGTAAAACATGATAAAACAAAGATGATCATCAACACACGTATACTGTACAGCATTAGTACTAATCAAATTGGCGCCTAAATTATTGGAGCTAATCATTTATAATATCTTTTGCTTGTAACATAGTTTAATAACCCACCAAGTGTGAATGCCAGAGGGGTGGGGTTTTCAGCTGTATAAAACTACAGTACCACCATCAAGCTTTTTAATTTAAGCAGCATCTGGTTTTAGGGCATTGTCTATTCCCTCGTATCCCCCCTTTCCTGTCAAGAAAATCTCAAACCCTAACTAACGTATCTCCTTCTTTATCACCCTCCATTGATACGCCAGAACCAAATCTTGGTGAGAATAAATCACAACTTGGGGCCTGGCA

>RsbHLH081

TTTCTGTATTACAGTAGTATTAAGAAGGTTGGTATTTTTATAAGCAGTAGTGCTACAGGTACGGCAAATCCCGCACAACAATTGCATACAGATTCATTTTGTGATTGTCTTGGGTCCTACAAAGATGATTGGAACTGCTCATTTTATTCAAAATATATTGTTTAGAGTCCCTATAAAAAATTATCTCAATCCAATACCGACAAGGACGTTTCCAAATCATCCAACTTTGCTTCAATAATTCAAGCCAAATTCTGAAGCAAAGTTGTATGATTCGTAAATGCCTTTACCGGTATCGAATTGAGATGATTTTTTACAGGGACCTTAAACAAAATATTTTGAACAAAATGAGTAGCTCAGATCATCTTTGTAAGACCCAAGACGGAGGCAAAATGGATCTATGCGCGCCTTGTGTACAAGGTTTGCTCCCTAGCATTTCTGTTTTACAAACCTCACGATTGAATCCGTTTAGACACGATGATATTATCATGATTGGAATGTTGGATTCAAGTGTTTTTTTGGTAATGGAACATTGTTGGAATCTACCCAATTATTTGGCTCACCAAATTGTAGGGGTTTTTTTTTTTTTCAATCGATAGAAGAGATTATTTACATCATATATGGAGTACAAGTATAAACTCAAGATACTACATCAATTGTAAGAGTACAACTCAACCAATATGGCTCGCTCATTAAAGGGTATAAAAAAAACCCCTCTAAGGAAAAAACACCTACACGTAGCCAGATGTATGTAGATTCGAACTCCTGACTTCTTAGTGAGATGCATGAGTCTAATTTGTATAGAAAAAATAGATCTAATGAAACGAGTTTTGATATTTGATCGATTTAATTTATTTCCTCATTGAGATTAAATTCTTTCCACCTGTAGTGGAAAACCCCACTTTTCCACCACCGTCTTTTTCGGTCCTATCATGTACTTATTGTTGATCTGAACCATTCATCTTGTAGACCCCACATAATAGTATATCAATGCGAAAAATAAACTTGATCGGACATCAATAGAAATATTAAAAATTGAATTTTTTGCTTTGTAAAATAGACAGTTTATCTTCGTACTTTGTTTGTCCATTTTTAAAAAAATACCTTCTTAAGAGAGCATAATCATTGCATTTATATATGTCTCCAATTTTCCAAAAGCTACCCTTCAACTTTTAAAATTTGTTCAATTGAATTCAAAAGATAAGGGGAAAGAAGGGAATTTCATGGCATTTACTCTATGAACTCTTCAAATTAAACGAATAACTCGGGACAATTCAAACTAAAGAATTAGACAAATAAATTGAGACAGAATAGATTTATGCTAGATGATTGAGATGATTGCAATTATTAAAATAAAATTAAAAAATGGACAAATAAATTGTGATGGGAAACGCTAATGTCTCTCTCTCTCTATATGTAAATCTCCATCTCATGTCTAGTACTCTATTTATTTCCTAGACTACTATACTATACAATAGAACTACCATCCTATTCGTAGTCAAC

>RsbHLH082

TCTTATTGGTAGGAGTTTAGACTTTTCATGAGCAGATGATGATTGGGTTTTGTGGGGCAGTTTGGCTTCCATTAAACCATCTTTTTGGTCTCTTTTTTTTTATTTTTAATTTTTTTTTCAATATTTAGATTAGATTCGCGTCAAGTTTTTGGAATTAATCATTAGACGAGTAAAATTTTAGAAGTAAAAAGTTATGATGAAAAAAGGGAAAAAAAAAAAGAGTTCACTAAATAGAGAAAAGAGTTCACTTAAAGAATTAGTGGATGGAAATGAGGTTTTGGTCAATCTGAGTATGAACTTTGGTCGAATCTACTTCCGGTTGTAGATTCATGAGGAGATGGTTCAAACCATTTTCCTTCAGTTTAATGGAGAACTTTTTCAGTGCGGAACGGGTACCACATGGTGCCCGAGCAGTGCATCCCAGCCGTCCATTCGGGTTTTGGATGGCTCAAATAAGGAGAGAGAAAGAGGTCTGCAGAGAGGAGATAGAGGGTTTTAATTTAGACCATTCAACACAATTTTGGATGGCCCGGATGCTTTGCTCGGGCACCACGTGGGCACGCCCACATGGTATCCACCCGGCACTGAAAAATTGATATGGATAAATGATAACATAGTAGTTGAGTTGGTAAGGGATTTTTTAAATAACCTGAATAGCCTTCTGTCTGTTTTGTGTCTGCTATTATTAAAATGTGAATGATAGTATCCTCAGCATTTCATCATCACCTAATGTTCAGGTTCCTTTATATTATATGTCACAGAGCTACCAAGTTTTTTCTTCTTTGACCAAAAATTGTTCATGCTTTGTAGTTTTGTTGTTGTTTTATCAATAATTATTCATACTACCTCGGTTTTCGTGCACGGTACTATATTACTACTATTTATCATGACAGATATTATGTCTAGAATCATAGTGTTCTACCGTCTTGAAGAAAATCGTCTTAATAAAATGTCAGCCACTTGTTTTTCTATTAGTTTGGGGGACTCTCCACGGGTGGGTTTTCGCCTTTTCTGTGCTTATCCCCGTGAAAATGTTTCACTCGTAAGATTTCGAGCCTCAGGGCAATGACCAACCATAGCGATACCAAAATGGGTTTCGTGCTACACACAACCACATAAATACTTTCCGAGTAATTTGTATTAGAAGTTTTGTTACTCTTGTTATTTCTACCTCTCCTCGTATACACAGAGTTATACATTATCGGTGTCATTGTAACTTCTCGTTCTTTCTGGAGGACTCCTATAGTGGTATTGAACTGCCTAATAATCTCAGCAATTTCCCACACAATATTGTCTGCTCGATTTTTGTCTTTTTATTTCGGGGAACTTGACGTACTTTGATGTATACATCAGCTGATGGTTTCAATTATTTTCTTTTGGACCTCTCTCTCCTGGATTTTCGAAGCTATCTTGTAGATTATAATTGGTTTCTTCTGTTCATTTGTATTGGTCTCTACTGCAGTTTTTATATAGTGAGAAGCAGCAGCACTAGAGGGAAAG

>RsbHLH083

ACTAGGAAAGATTAAATTAAGTGTCCGTGCAACGTGCGACAAGGACACGTGGTGTCACAAAACCTTTTTTCACCACACATTTGTGTGGGTTTCACATTAATTCTTTGCGATATGGTTTGCACAAATATGTGGTAAAGAGAAGGATTGGAGCACCATGTGTCTTTACCCCTGCGGCAATAAATAATTTCTCTTTAATTAATAGGGCTTCCAATAAAAAAACAAATCAAAAAAAAAAACACAACCAGTCCACAATAGAAAATAAAAGTTTAAATTCTGTTTAAGGTTCCAAATCTTCATATGATGGGTGATTTTATAGGAAAAAACAAGGGGGGTGTGTATTATTTTCTGTTCAAAAATACACAATTAATCATTTTGAAAACAAAAGTGCTCAGTATCCAAAAAATAATATCCCGAAATTATCCCGAATAAACTTTTAAAAGTATTTATGACTGTTAATAGACTTTTATTAGCAGTAAGAAGTACCTGTTCGGTGTGATTTGGGGGTACCTAGAATATTGCTCTTTTCAAAATATCTCCATCATTCTTAAGTTTTTTTTTATCTTTTTAATTTACATAGTTTTTTTTTTTTTGTCCTTGCACGGTTAACTCAACTCTAGAAAGAAAAGACTAGTGTTTGACATGGTCGTAAAAAAATCCTAAAAGTGAAAAAACTGAAAAATCAAAAGTCATTTGAAGTTTCTGAAAATCTTAATTCTAGGAAAGAAACAATAACTTATGCCTATGGAGTACCGTCTTTTAATTACAATAATTTCGTTTAAATATTTCTAGCAAAAATACTTTCAGCAGAGACAATCTGCAGAGATCCACGCAGAGACAAATGCGTTTAGCCCCATTTCGAGTCCTGAAAAAATCTAGAAAAATATCCATAAATTTTTGAATATTATTTTATGGAGCCCTGTAAAAAATCAACTCCAGTGGATGTCGATAAGTATTACTTTTGGATTCGTAGAGGCGAAATAAAATAATATTTAAAAATTTATGGATATTTTTCTAGATTTTTTCGAGATCCGAAATGAGGCCAAACGCATTTGTCTCTGCGTGGTTCTCTGCGAATTGCCTCTGCGCGTAGCAGAGCTCTATTTCTAGTCCTCCAGTAAATAGTACTCCTATAAAAACAAATTAAAAAATACTCCTATAAAAGTACAGATTTTCCGTGTCTAAAATGCCAAAATGGGTCTGAAGGTTGGCGCAGAAAGTACCAAGTTAGAGAGAGAGAGAGAGAGAGAGGGGACCACGACTTCATCACAAATCAAAGTGAATGTCTTTTGCCACTTCCTTCATACACATCTCTTTTGCCTCACACCAATCCATGTACTTCATCCCTTCTTCATATTCTCCCTTTTCGTCTTCCTCTGAAACATAAGGTAGATAACTAGGGTACGACCCGAAAGCCCTTAAATTCCAACCTCTCACACACACACACGCTAACACAATCAGCGAGCGAGAGTAGGAGCGGGAGCGAGAGCACGAGCGAGAGAG

>RsbHLH084

GCTAAAGTAATAAAGTTGGTTTATAAGGTGTAATTTGGTCCAAAATTTAAAGAGTCTGATGCGGATGCTCTTACGTACATTGGTTCCCTAACAACCCACTATTATGCAGTCAATTTTGGGTTCATAAATTCCTTGAAACTATTGACCATACTTGTTCAACAAATATGAGGGATCAGTGCCTTTATAAAACGTATGTTGTGCATCCGCCAAATCTAACCTACTCATTTTATCCGTCTCCTTGAGCAAGTGACCCATATCAAAATCCAGGTGGATTGAAGATCGATAGTTATGTGATTGGAGCGCTTTCCTTTGAATCTTGTTTTTTCTTCAAGAAAAATGCGTTCTATTTTGCATGGTTACCAGCATTCAGTAAATTTAAATTTCTATGACTGCGACTTAATTGGCAAAACAAAGAAAATGAAGAATGAAAATATGAGATATACTTATTTATGGAGCCACTATAAAGCAGTTCTTTACCTCCTAAGTGATCTCAGCTGTATGTTTTTGCATTAGATGACCCGGATTAAAAATAATATATTAACGCTACTATTACCGTTGTTTTTAATCTAGATCATCTAAAATATTTTTAGATGGCTGAGATTGAGTTCCGTAAACACTTGTTTACTACTGCTCTATAAACAAGATTTTCTCAATGAAGAATTCAGGCCTTATCGCACAACTTGCTTGCTGATCCTCCCCAATTAGTATAAACAATCAATAAAAAAAATAAAAAGTGAATGCCCTCCACTTTTTTTTTGTTTGGTAATCAAATGCCCTCCACTTTACCCAGTCTGATAACCGTGGATGAAATTATTACCGTTACCTGAACCTTGTACCGTGCAATACAATACGATATAGATTTGGAAAGCTAGCTACCACTATCTCAAAATTTGTCATTATTACTGCAAGTTGTTTTTGTGATGTCTCCTTAAATTAATTCCAAGGAGAGAGAGCGAGGGCAATATGGGTAAAGAATCTAGCCCCTTTCTCCTGTTCTTTCCTCCCTATGCCCCCTTTTTATACACCCCCGCCTTCTCTCTCTCTCTCTCTCTCTCTCTCTCTCGATCTCGAATAACTCTGAAGAACGTCACTCGTTTTCTTTTGCTTTGATTTTTGCATTGGGTGGTCTGTTTCCACACACCATGGGTTTCTGTGCTCCCATCGTCTCCCTGAATTCCATGTGATTTCCCTCAACCATCAACTTCATACATAACGGTAAGGACACCACTAGTCAGTTATACGGACACACACACACACGCAGAGAGAGAGAGAGAGAGAGAGAGAGAGAGAGAGAGAGAGAGAGAGAGAGAGAGGCACCCATCAGAAAATTAAAGAAAGAGTAGGATTGTCATGATAGTTGGGTCACTTTCATTGTTTTACTCCTTTGAGGCATTTCTTTTACTGGTGTTTTCTTGATCTCCGCCACCTAATCCATCTTCATCACCATCTGGCTTCTTTAGTTTCATCTTTTAGTTTCTCCCCTGAGAGAGAGAGGACGAA

>RsbHLH085

TATATATATCTAGAGATTAAAAATTACATGGGTTCTGTTGTTGTGATAAGTATATACATCTAGAGAGAAGTGGTTGTGAATTGGACCAAAATCAAATGTATTAGTATCGATCATTTTGAAAATTCAAACCTCAAAATCAAATGTTGAGCGTTTATTAATTTATGAGTGTCATGAAAAGTGGAGAGGTGGGTAAAACAAAGTGGGAGTGGAAGAGCGTTGGGTACTGGCTGGCCTGTGATTCCCATTAATAAAAAGAGAGAGGTGAAAGTGAAGGTGATGAGTGTTAATCTTGTGAGGTGGAAATTAATCCACCCAGCTCCTCTCTCTCTCTCTCTCTCTCTCTCTCTCTCTCAAGTCAAAGCCAACTCTAACAAGACAACTATGCACGGCCCAGTATCTTTATATAACTCCATCTTGTATTTTCAGACCTTTCAGTTATCAACTTCTTTAGGGTTGTACTCCAGCTGATATATATGCGTGCTTCTGCTCTTCAAGAATCATAACCCCACTAGCTTTGACTCTTTGTTTTTTTCTCTTTCTCTTTCCAATCAGGTATCTAAACTTGTTTGTCCACAACTATATCATATATTTGCTTATACGAGAGAGAGAGAGAGAGAAAGAGAGAGAGAGAGAGAGAGAGAGAGAGAGGTGTATATATTTATTTGTGTTTTGATCGACAAGAGATATTTATCTATAAGAACTTGCTTTTCTAACAATACCCACAAATTTTTAGGCGAGATAATAACCTCGGCTATGCTCGTTTAGTATTTCTACCAAAAATTTTTTATTTCGAAAACCACTTTAAATATTTTTGTTTCAGAATTTTCTCGTTTTAATTTTAAAAACAATTTTTTTATATATATATTTTGCAAATTCCATTTTGTTCTATTTTTGGGAGGAAAAATTTTTCATTGAAAAATTTGTTTTTAGGGTATAACTGTAAACCAACTAAACAACCACAAATTTGAATTCTATTTTTAAAGAAAAAAAGTGTTCTTGACAATAAACAAACGGTACCTTGTCTTTTAACATTTTCTTACAGGGAATTATACTATGCACAAACACACTATTTTGTTCTTATTTTTTCAAACTTGCAAAATGGACCATTTATTTTTGGAAAAGTTTGTCAAGAATTAATAAATAAGGGTAAAATCTTGTAATTCCAATTTTTTTTTTACAAAAAGTGGTGGGAATTTTTCAAAATTTAATTACGAGTCGTAATTAAATTCCTAATTTTACTATAGTTTTTTTTTTTTTTTTTTTCAGGAGGTGGACCCAGGCCGTGAAATTTCAGTGGGGCATTATTTGCATGAATTGAGCTCTCTTTGAGTGTTTCTGATAAAAGCTACTTCTATTGTTAGTAATGGACAAAGAAGGGAACTACTCGGTAGTACGTATAACTCAAATCCCATAACTTCCCTTGTCTACCATATTTTCTTGATAAATCTTTTCATCCCCACACATAATTTTTTTTTCCCCACTCTAAAACCCTAATTCCTG

>RsbHLH086

TGCCTTTGGCATTTTCCATGCCTGAAATTGAATGAAATCAATAATTTTTGTTTTTTGGGGTAAGTAATGAAAGCTACATTTGTACCCAAAGGACACAAGATCTGAAGAGATCTAAAGACATGCCTGAAATTGGATGAAAGCAACATTATTATCAGTAAGTCTAGGAACACTTCTACTGACCACTCTCTGATCACATCTCTTTCATATATTGTGGTGCCCATTCCACAATTAGATTTGTGGGGTCGATACATATGTGAGAGAGGTGTGGTCGGGGAGTTGTCAATAAAAGCGTACGCAACATTGTTGGATTATTATTGACTTACAACTCTATATAAGGACACATGCAAGATCTGATGAGAGATAGAGAGAATTATAGGCTTAGTTCTAGTTGAAGATGAGATTCCCTCTTCATGCATTGTATATCTTTCATTTCCTTCCTAGTGTGAAAGGATTTCAAAAATTTAAGCCTTCCAAACATTTATGGTTCACCTTAAGTCAATATATATTTCATGAGAGAGCTATTGACTAATGTCATCTTTCAATTCTCTTGTTATTTTGTGATTTTCCCCTTTGTACGTATACTTTTACCTTCTATGTGACTAGTGTTTGATAGGATTGTGGACATTCATTCACTGGGACTGGAGAGAAGCTAGCCTTAACGCTTTGGCTAACGTTATAGACGTAGATCCGAACAGAACCGTCCGATGAACATGGAGCTCACTCTATATATGCACGTAAGATTTACACAAACTGAACGATTATAAACGCATGCGCATTTTTTTAGAATATGTGTCTTATTTAAAACATCTGTGTTCGGACTTCGAAGCATTATTATGATCCGAATGCTTTTTTCATGATAAATTATCCAATTCGGAGAGAGAAGTGTCACAGCTTAAGATTCAGTGTAAACGTTTTCATTTGCTTCTCGTTGGCCCTTCTGCAGGGTTAATTTATTGAGGAAGAGAAGAAGAACTGATTCATGGCCGACTTGCCGGTGTCATGGTTATGTGATCTGGTAAGAATTTTTTCATGAATCTGGTCGCTTACGAAGCACGATTGGATTTGACAAATCCTAACCATGATATTAAGACGCCATCGTTTGATTTATTACATTGTTCAAATGGAAGATGAAATTAATCAATGGAGTCTTGAGTCTACTGCTTTGAACCTGAACTGATCCCCCGAGTTGCTTGGATTTGCTTTAGAGGCAATGAAATGGGATTTAGTTGACACAGTCATTTTTCTATCGTTCGCACTTAGCACTGCTGTAGTTTCAGTAACATATGTAGTATATAATGTTGCATGTGTGTCTGAGTTTAAGCACGAGCAGTGCTACAGATACAGATTCGGATATGACTGTGTCTGAGACGTTCGATACACACACAATTCGTACACATCAAACGGCTCTGGACCGAATAATTCCAGATTTATGTCCGGATCTTTATTCAGATCTGTGTCGCATATTTATGTATTACCGAGAATTCGATGTATTTGCAGGGA

>RsbHLH087

CAAGAGAGGGAGCAAAATGACATACAAGAAAAACAGTGCATAGTTTTCCATGCTTTGTTACAAGTGGAAAACTCAAGGAAAAGAAACCCACGAAACCAGTATGGGGCCTAACACAGAATAAGAGAGATTATCAGTCTTGCCGTTCTATTTCTACTGTAAAAGGAAGTTTAGGAAAAGGAATGTATTTCAAACTCAAAAATCATGCGTTTATGCAGATAATTTTTCTACTAATATGAATCTTGTTTAATAGATCTCATTGAGATCTTTTAAACAATGAAAAAAAAAATCAAAAAATTATTTTTCATTTTCATAATATGTGAGTTCAAAATTACCTTCTTTTTGAAAATGAATGTTTAAAAAGAAATTTGAAAAAAAGAACTTCTAGAAAAAGAAGATAATTTCAAACTCAAAAATCATGAATTTACGCAAATAATTTTTCTACCAGTATAGATCTTATTTGATAAATCTTATTAAAATCTTTTAAATGATGAAAAAAATATTAAAAAAATATTTTTCATTTTCATTTTCATTGTATTTGAGTTTAAAATTATTTTCGTCTAAAAGAAAAAAATATTTTTTTTCAAAAGTGGAACAACGCATCTAGCTCAGGGGCTAAAAAAATAGAGTACATGTGCAGGGAACCTGATAGGATAGGACCATTGATGCAGCCAGTTCCATCTTTTTCGGTTCAGACAGATTTGTGGGTCTAACCTCTCAAATATACAGTCCGATATATATAGCAATATCGCAAAAGCAGATTCCTTTCCGTTTTTGTCTGTAGACACTTTTGTAGTACATACTTGAGTATTAAATTTTTTTTTTTTTTTGTCTCAATTCAAATTCTTTTTGCATTTTGCGTCAAATTTTTGTGAATTATAGATTCATCTCAACGAGAGAAATTGAAAAAATAAAAAATCACTCCGTAAATTCAAATCCATTTGTTGTAATATAGAAAAAAAATAATTCAAAAATTAGTCTGTTTTGAATTATTTTGTCTTTATTCAAAAAAAATTATTTTCAAACAAAATTCGTTACTTTTTTAATTCTTCTCGACGAGACGAAATAATAATCTATAAAAATTTAACGCAAAACTAACGAATGCGATTTTTTTTTTTTGAATAAAGACAATCTTCAAGGGTTATTGCGAAACATTCCTATGTATGCTTATTACATACGCCAACGAACAACTGCAACCCCATGTTATTAAGAGCTTGCTTGGATTCGGATTTGGGATTTTGGTTTCTATCCCTATGCACGGTCCCCAATAAATTCTCTCTCAATTATTACTCTCATTTCTCTTTTTATCTCTCTCCATTCAAAATCCAAATACCAAAACGAACAAGCCCTAAGCCTCACGAGAGAGAGAGAGAGAGAGAGAGAGAGAGAGAGAGAGAGAGAGAGAGAGAGTTACTAATTGCTACCTCCAGGAAGACAGACTTGAGCTTTTCATTAAGCTGCTCCTTCCACTGGCATCCCTATGTGCTTAAGGGAGAGAGAGAT

>RsbHLH088

TGTAATAATTTGAGAAGTGGCAAAATTATTCTCCTTATCAAAATCCAAGAGCTTCTTCATCATTTGTGTAGAGTAGTAGACTTGCCATGTCATGTACTACCATCTCTCTAGAAAGGGGGGAATCTTTTGAGAGAAAGAGAGAGAAGATGAGAATGTTTCGTGGAGATTTGGGTTAGCATAGAAAGGTTAATCAAAAAAAGAGGTAATGAAAAAGGTGAAATTGTGGGGTTAAACACAAAAGTGGACACAAGTGTTTGGAAAGGGGAGGAGTTGGTCTTTTATCATAGTAGGAGTAGATGATTTGCAGGCTACTGAACACTACACTTTCCTACGGCCCTTTGTTTTTGTGATCAGTCACATGGAAAGCCAGATTCCCTTCTGCATCAAACGAGGCCCCACGCAACCTTGCTGACTCAATCTCTGCCTCTCCCTCTCTCTGCTTACAAGAATTATCGGTGGTCTTATAACACCGTCACATGTGTTTTAATATCCTTCTACACTACACAGTTGAATAGGGTTTGGGATTAAGCTTGTAAACTCCACGTAAACGGATAATGGAGAAGGGTGTAGGAGTGCCATGTGGCGGTATATTGATGACACTGAATAATTACTCTTAACATTGGGTGGGGAGCTATTTTGCTACATGGTGTACGTGCTTTATCTTCACAGATCTAAGATTAGTTGAGATTCACGTAAAGTGATCTGGACGCTCGATTATTGATCAAAAGCATACTACTTTCTCAATATGGGACACCCTTATGTAGCTTGAAATGTATTCAAACCGTTTATTCACACATATACGTCTTTCCATGCATAAAATGGAAACAATGTAGCGTGCATTGACAAGCGTTGTGTCCGTTCGATACACGAGTCCAAAAATCACATTGGTGTGATTTTTTTGTCTCATACATCGAACATACATAGACGCTAGCAAAAACTTAAGCCGATGAAAATTTATTTTCATATTTATTTTGTTGGAATAACTTTTATTTCGTATGGTAACTGAAACATTATTGTTTGGAGATTGGTTGTCTCCATTTTTCCTTTTTCAAAAGCTTTTGAAAGGACGACAGGTTTTTAGTCCAACGACACTTTCTCACTTCTTCCATATGGAGGGTGAGGTTTCGATTCTAATCAGATCGCGAGTGCTTATTGTGGTGGTTGATACTTTTTGAACTTGCCCCACCCGTATGACGCTTAGCAGTGCCCCTAGATATCTATCTCAAGTGAAAAAAAAAAGTTTTTGAAAGGAAAAAGACATTAAATCTAAAGGTTAATCCAGTTGCTTGGCGAGTTTGCTTCCCACACAATTGACCAAATTTCAATTCTTGGAGTTTAGCTCTAAGAAAGTAATTTATTGTAATTTTTCTAGTCACCGTCTAAAAAAACAGGAAAGAGACATTTCTTTGTCCACACTCTTTCCATATATATGTCTCTCCATCTGCCTTTAGAAACAGGAAAGTGGAGAGAGAGAGAGAGAGAGAGAGAGAGAGAGAGAGA

>RsbHLH089

TGATTTTGAAGCAAAACAAGTATAAGGGGGTCACTGTTTCAGTGGTATTCTGTGAGCAGCCACTTTGTGCATATTGCCAAAGGTTAGGTCTATCTCCAATCCTCCCCCGCCCATAGCCCCAATGAGTGGGGACTAAATGGGTTTGTTGTGTTTGTTTGTTTAGTGTTCTTTACGAAATGATCTGGCGTTTCATAGGTTCCCCGATGTACAAAAGTTCCATGGGCGAGAAATCCAGCATATGATGGTCCGTTATTTTGCATGAACCAATGCAATGACAAGATAAATAAACATTACTCAACGCCCGAAAAAGGTGGATTGATGCATAGAAGCTGCTCACCTGTCATGGCACACCATGGCAAGATGGGTAATGACTCCATTATGTCTTTTAACCACTGATGACTACTATCTAGTTAGTGCTCCCGGGGCATGCCCCAACCGCCATTTTCCCCCACATTAATTACTTCCCTATAATATCAGCCCTGTCAATTGTACATCATTTCTAACTGAATGCTAATTGTTGCTCACTCATTTCGTGGAATGCCCAACCCTAATCGCCCAACCCTCACGGTATTACAACGAAAATGAACTGACATGGGTACAATTAGTAGGGGAATGGTACAGAATCAAGGTCCTTACCGGAGTAGCCGATGCGTCCTTTATCGGTGGTGCGGGTGCAGGAAAAGAAGCACTCATAGCCCTCGGCCACAAGCAAATCCGCCGTTAGGTCCTGTCTAGATATCTTGGTCTCCTGTAAACAGATGATATCGGCATCGAGAGAGTCGAGTAATTTGAGGAGAGAACCGAACTGCGAGACCCGAGGCCTGAGGCCGTTGACGTTGTACGTCACTATCTTCATTTTCTACATTTCACAACCACCAAATGCTGCATATGGAGAACGACTACGAGTGTGTTCTTCTCGACCTCTTTGTTGGCGACAGAGTACTCCTATGTATGTATGTATACCTCTGGAAAACAACAAGTACTATCCTATATTGAACTTTTCTGTCGGTCGGAGATTGTGTTCCGTAAACCCTTTCTCCGATCAACCTCTGAACTGAAGTCGACCATCCAAGGAACGAGAACTGAAATCAAGTCGGACTTTTGGGTCAAAATGGGCTTCTTGAAATTATTGGGCTTTAATTGGGTGGGTCTGGCCTACCTCTATATTCAGTCCCAAATCATTTTCACAAATCTCTTTCCAAGTGGCCCAACGTTAGATACCACCGCTGTCGTTTTCGGTTGGTGCGCTATCCTCGTGGCTCGTGGAAACCACGAGTCCGTAGCTAGCTCAAAGCGATGCATTCCCCTGAATGATGTCTACTCCTCTCTGTCTCTCTTCTCGAATCTCTTTGTACTACATCATCCTCCCATCAGAATTCAGAGGTGATCAAGATCAAAATCTCCACCGGTACCCCATAATCTAACAAGAATCAATAAAACCCTATAAGCAACGATTTCTAGTGGCCTTTCAGTTTATTACGGAATTCCCTCTACCCTGCA

>RsbHLH090

CGTGGATTTGAATCGTGGATGCCCGCCCTCTTCCGCGATACCCCTCCGTGGACACTCTTCCGCAGAGGTGACTACTGGCCGTGTTTACCCACGCGTCATTGTTTCATGGGATGGTCAACCATTCTTCTCACGCGGGTCCCACCTCCCCGTGTCCTTTTGCTATTGTAGGGTCAAATAGTCGTCTCTCATTTCAAACTGGCAATTCTGACCGCGCCTACAGGAAGTAAATCGCCGGGGCTTCCAAATAGCTTTGTTCCAAGTTTGATGTGTAGAGCCTATCGCATAGATAGTCTTACAACGTTCAAGTTGATCAGACATTAATATCTTATTGATCGGGCCAAATTATTCAAGACAAACTTGTTTCGTTGTTACCAATGTCGGAGTGGCTTTACCTATCTCCAAAGATGTTTTAGGCGTCGATATACTGGGCCGGTTTTCGGCAAGGAGGTGAGGGGTGGCAGATTTCTCTTTTTTTTTTTGAACGGTATGTGAATCTCGGTCTAGTTACCACCGTAAGCAAAGTGAATCATCAGTGAGAGTACAAAAGTGGTCAGGTGTAGAAGTGAATTAGAAGCATGTTTATCTCATCTCATCGACCTTGACAAGCGTGCCAAGGCAACAATGAGACAGCGCAGAATTGTGCTGCACCAATTTTGAAAGATTTCTTTTGGACCCCATTTCTGAATTTTGGTGGTTGTCCTTCTGACAGATGTCATGTGATTGTGGGCCTTCTTTTGCTCTTCTATCCAAAAGGAGGAAGGGTCAGATTATTTTTATTTTTATTTTTTTGGATCTAAAAGAAAATGTGACACATTCAGCCCAAGGATTTCGAGTGATATTGAGATATAATGGTTCATGCACCTTAACCATGCTCTCCAATTTGAAACGGTTGATAATATGCAGATACTAGTTGATAAAATTTTAGTTAGAGGAAAATGAAGCTGGCCAATTGTAGTGGGATTAATGTGGATTTACGAATGAAATTCTGCTGTCACCAAAGTGGAGGAATTGGAACCTCAATTTCCAACTGAAATCATGTGGATGGATCAGCTACTGTGACAGAAGTAGACTTTTGGTCCATAAAGTAGAAAGTGTGAATCGCACTGTACGCAAAATGTATAAAATAAATTTTAAGGGGCCCGGTAGTTTTTGAACTTAATTGACCGTTTGTTCTGTCTTAAGGTTTTAATTTTCATTATCAACAACTCTTGGGTTAGTATCACCTCATCCCATGGGATGCGTAAATGTGGAAAACGGATGTACAATGGACATGAATTTCAAAAAAAAGTCTGAGATATGTACAACCTGTCCCAAAACGTCATGTATTAAAAAAAATGTATAAAATAGATCACAACTTGCTATATATATAGTCTTCCATTGACGCATCCTTGTAAGTAGACTCTGTTTGGCTCCTATCTCTCTATGCCTCCTCTTTTCAGTTCTTCATAACTCATCTCTCTCGCTCTCTCTCTCTCTCTCTCTTAGTGTACGAGAATTAAT

>RsbHLH091

CCATGAGAGTAGTTGAGAGGTGTGCAAAATGGACTTTGAAACCAATTACTCCATAAAAAAAAAACCAGTAAAATATAAATAGACAATAGATACGTGATCATCCAATTATATGCCGGTCCACCGTAATTTGGGTGAACTTGATCAATTTGGGTGAACTTTACATAACTTTTCAGGAGTTTTTGGGCACGGTATATTTGGTTGCTTTCTCTTAATCTGTAGAAGATTCGGGTAAAATTTTTACACATTTTTTATCTCGAAGAGAGGAAATGAAAAAGTATAAAAACATACACTAAACTTTGTTGAAAAAAGCTTATATAATATGACGAAAAAGACCCAAATTTTTGAAGACAAAAAATATGACAGTAAAAATTGGCCCCAAACTTTACAAAGATTGGGACGGGAGAAAAAAAGGCTGTGTAAAAAAAAAAACCCTCCAAAATTAGGCCAAGCTCACCCCGAGAACGCTAAACTTCATGATAAAGAGCTGTGAGCAAGTCTTAGTCTTGCCCTTATTTAAAGAGCAAGCCATCGCCTCGAGAGTTTCTTGGTTACACGTGCCCCTTTGCTTGGTTCACTTCACTTGCAAATCCCAAGCCTTTTGCCTTCTTCTTCTACGACTCGTTAAGAATCCTCCCTATCTTTCTAATTATGTATATGATGCATGCCAAGCTGAGAACTTTAATTGGTTCGTGAGGATCATCATAGCTTTGGGAACCTTATTTAATGCATGTGGATTAGCGTATGATGGTGGGTTCGAGGAATTTATCATTACCCAGAAACCAAAAGTCTATGAAGTCTGGGAAATGTCGTTGGAGGGCTAATTTTATCCCAAAAGAAATTGAATCTTTTCCGATGGTTTTGATGGGATTTCAGTGGGTACAAGTAGTGTGAGCTGATCTTGGGGAATTCTTGTTCTGGGACTTGGTTTGATTTACTTTGAAAGGTATCTCTCTCTCTCTCTCTCTCTCTCTCTCTGTGAATTGAATGATTAACAAGATGACAAAGATTAATCAATAAAAAAACTTGATTATTTTAGATTATTGGTTGGTTTTGGAAATCTAGCCTGGGTTTAACTTGGTTCTTCCTCTAATGATTGAATTTGCAATTAAAAAATCTCACTTTTCTTTTACTCAAAAGACCTCAGTTTGATATTGTTCTTTAATTCCTGTAGGTGCCATTAAAGAAGGTTTTGACTTTTACCAGCCTACCTGCATTGCCTTTATGAGTTGTGCTATTTTTATATTGCTGCCGATATGGGCATGAGTTATAAATGAGACTTTTCTTGGAAAATGTATGCATACACGCGTGAATATGTTATGTAGGCAACTACACATATCCTTTTTCAAAGTCGGTTAGTGTTTTGCTGATGTTACATTGCCTTGGCTGTTTTGGGGTTATTTTTGTTTGCAGTAGGAGGTGGATGATACTAAATATGGCCAAGTGCTCAAAATAGGGGCTTTCTCTCTCTCCCTATATTAGTTGTTTTCACTTCTTCAAATC

>RsbHLH092

GTCATTAATGCGTAAAGTGCATGCATTAATAGTACAAAGGCTTGCATGGCGACAAAACACATTTGCGACAATATATCATCACTGCACATTCACGTAACGCCCGAATTTTGTAATAATTAGACAGTTGGGGATCACCACTGATCGATCACATGATAATTAAGAACCCCTATTAATTGCACCCAAATTACACTTTCATTTTAAAATCAATTAAGAAACCAATTAGTCCCGGGAAATGAATCAAGATAGCTCTCATTTGATGATCCCTCATAAATGAATAAGTACTGTTGTCTAATGTTCTGCCGAGAGAGTCTTGTTAAATGTGACTAACCAATTATTGATGGCTTCTGCCAATTAGGCCCCATTTGATAACAAGTTTGAGATTTGGAATTGAAGTATTAATTTCATGGGAATATTAATATTCTGTTCGGTAACAAAAAATAGTTCGGACTATTAGTTTATTGGGATGTCCAATCCGGTCTTTAGGAGGTATGAGCAATACTCAATGGGACTTGACATTCGTAGTCTAATCTCAACTCCTTCTCATTATCAATTTCTTCTATCAATCCAATTTTATCATTTAATCTCATTTCAAACAAAAATGATATTAATAATCCCATCATCTAATTTTATCTCAAACAAACATCATTATCAATCCCTTCTCATTAATTAACAATCTCTTCTCATTACCAATCCCAATCCTAATATCATCTTCTTACGAATCTCATTCATCTATCACTGTTATCAAATGAGGCCAATGTTAAAAAAACTTGTACCTTATTTTCAACTCATTATTCAATCTAAACTCTGGACTCACTCGATAAACGTGCCAAACTATTCGAGTTCTAAATCGTATTCAACTCGTTCAAGGAAATTGATTTTAGACTTCAAAAATTGATGGATGGACTCCAAAACAACTTCGTTTTGTGTATCAATACAGTGCAATTTTGGAGCCCTTTCATCAATTTTTAGAGTGCCAAAATCACAATCCCTCGTTCAATTTGTAAATCTGGTCAATAATTCAGCTCCTACCCGACTATATGGTCATGACATAGGGTGTGTTTGGCTCTTCCGATCTACGCCAGAGAGTGAGTGTGTGTGTGTGTGTGTGTTTGGGGATGTGGTGAATGTCATGGCTGTGGCCAGGTGGGCCAACACCATCGCAGTGGGACCCAAAAATGGAGAGAGAAAAGAAAATATTGTACAAGGCGCTAGGTAGGCTAGCTTTAGCTAATCCGTCTTTTTGTTTTGAAGCCAGTAACGGCATTTCGGCTTATGATTGTATTTTGGGATCTTCCTAGCCCCAAAGCGAATCACCTCATCTAGCTCTTCTCTTCTTTCACACCTTAGACAACCTTTCCTTTTGCCACCCTTTTTTAAACCCCTCCCCACCTTCCCTTCTTCCCTCATAAGACTCTCTCTCTCTCTCTCTCTCTCTCTCTCTCTCTCTCTCTAAACATTTTGTTCTCTTAATTGTTTTCCTTTTCTTCTAACTAGCTAGCC

>RsbHLH093

TATTTTCTTTTTTAATAGATTCATTCGGGGTTACAGTTTGGTTATTTACTTCTTGGGGATGATAGCACTCGCCATGGCTCAAATACTTGGTGCTACAGCACAAATCTAAATCGAAGTTTGCAATGAGCTTAGCATTTGGATGCTTGATGAAGTTGTCCAATTTAACCATGCTTAAAAAACAAATCAGCACATTTTTCTTCTTTATTCCAGCGAAATGGAAAATGACATTTTTTTTTTTTTTGTCTTCCATCACTCATGTGAAGCTTGATTGTGTCGGTGCTAAAAGCAAAACGCTAATCGTTGGTGTCTTTTATGTTCAAATCTGTTAATTTTGGTGCCTCTAGAGTCATGATTGCTTATTGTCTCTAAACATATTGACAACTCACAATGGGTTCTTTTTGGCTCCCATGTAAGTGCTAAACAAATAACTGATCACTTGTTATCTATCTAATCCTTAGGCCCGGCACTGGGCGTAGGCTCACCAAGCCATCCCTTAAGGCCTCTAGTTTTGGACCCCCAAATATTGGTATAGGTCAAATATCATACATGTTAAAAGTTGTTGTGCTATTGGTATCGACGGGTACCATACCGAAAATGGACCGACAGCCGCTCTCGGCCAACTCCAGCCACCGGACGGCCGATCCGAGCGGCCCAAAAATTCTAAAAAAATAAAACGATGGAGCCTAGCTACGTAAGAATCAACGGCATCCGATGTGTGTAGGTGTTCAACCCAAACACCCTAAAATCTTGATGGCTTTTAGTATTATACTTCCTCCGCTCTATATTGAGAGTTCACCATGAAAAGTCGAGCCATTTTTCAAATCAATAAATCAACTACTTCAATGTATAATTTTTTAAATTTTTTCGCGCCAAATTAAAGTATTAATCGAGATCTTTAGTACGGTGCGAAAAAAATTGAAAAATTATGTACGGAAGTAGTTGATTTTTTTATTTGAAAAATGGTAGGAGGGAGAGAGTGAGACTCTCAATATGAAACGGACGGAGTACATTGTTTGGGAAGCATGTGGCAATCTTTGTTCTTGAAGTGTACGTGTACATCAATATACGTAGTATATATAGTAAGATTCTGCAGACATGTATGGGAGAAGAAGCAAGGATATCTGAGCGGTGAGCCAGACATTATTTGCGAACCCCAACTCAGAATTTTAACGCTAAGTGTAGCCTTTTCGGTCAACTTGTGGTGGTGTTTCTATGCATATTTCTATATTGATTTGGTCCCCACTTCTATTCGTTGCAGAAATTGTTGCAGTGTGCTTGACAGCATCTATGAAAAATTACAAAAACAATATTGAGAGAGAAAGAGAGAGGGGAGTCCCCTTTTTTGGCACTTTTTGATAGGGAAGCAAAATACCTATTATGCCCTCTCGCTCAGATAAATGGGATTTGCTACAAATTAGAGCAAGTCAAAAGCATTGGGTTGTCACTGTAAGCATCATATATGCTGGGGTTATCATCACCATTAGATAGCACAAAGAAGAA

>RsbHLH094

AATTCTGTGGCGCTCTGCCCCATTGCTTCTCTCTCCTCTCTCTCATCTATCCACGAGTAATTACGTGTCGTTCCACTTTCTTTACCTTATTTTTTAAAAAGAAGAAAATTTCAAGTTCAAATATAATAAAATTTTAAAATTTTTTTGCACCGTTTAAAAGATATCTATCAAACAATATCCATATTGCTAGAAAAATTATTAGCGTAAACACATGATTTTTGAGCTTGAAATTACCTTTCTTTTCTAAAACCTCCTTTTTTTACAAAACTGGACACACCATCTTAGGTTGTGATCCGTTAAGAATTTTTAAAAAATACTCCTTCCGTTTCAAATTAAGAGTCCCTTATTTCATTTTGGGATGTTCTGATTGTCCCTTTCCAAAAATAAGTGTAGAAATTTGATGAAATTTGATAAAATTTCAAAAGTGTCCTTTTTATTTGAATACAAATGGTGCAAAATATGTAGAATATATAGATAATGGTAGAAATTATGTGTAATGATTTCATGTCTCCTAAAAAAAGTTGAAATTCAAATGAGGGACTCTCATTTAGAAACGGAGGGAGTAAATATTTTATCCATCCCAAATTACTGGTCTCTTTCTTTCTTTTTATAGATGTCTCAAAATGAATGTCTATTTTAAAATGTCAACATAAAAATTAGGCAACATTATTCTTTCATCCCTTTTTTTTGGTAACCATTTCATCCTGTCATGACAAAGCATAAAGTAGGGACAAATTGAGAAAGTCAATACCAATTATATTTCACATCATTCTACCAATAAAAACTCGTTTCTCCAAAAGGGCCAAGAAAATTGGGATGGAGAGAATACTTATTTGTAATTTTGAAGCTTAAAAATAATGGACTTACTGAAATAATTTTTGTGCAATATGATCGAAAGCCCGAAAGTACCTCCACGTGATGTTTCAGATCACTTCAAAATCTCAAATTTAAGTGGAGTTCATACATTTAGTCATGATTCCTACAGAAATTTGTAATAGTTCGAAGCACAATGTGCTGGTACTCCCAAACTATTTGAATAATTTCAATTTATCCACACTCTCTCTCTCTCTTTTATTTATTCCGGCTACTAGTGTCAAATTCCGGTACCCAATGTCAGGATTCCGGTTCTACTTTTTCTCATCTATTTATACCCCATTCTCACTCTTCTTTAGCCTTACACAAACCACCCTCCCCTTTTTCGCAAGACAAACAGCGGCCAACCATTCTTCTCTTGGCGAAAACAAAGACATAAAACACCGTACAGTGGTGTGTGCAAACTCACGTATATAGTTAGTGAGAGAGACAGCAATAACACGTTTCGGTTAGAGAGAGAGAGAGAGAGCGAGGGTTAGGCATGAAAACGTACAAAGTTGCCTAATCCTCACAGTACCCACCCTCTCCCTTTTACTTTCCGCAAAGCACATTTCTTGGCTCCCCTTTTCCTCTCTCTCTCTCTCTCTCTCTCTCTACCTTCTCTGTCTGTTTCTATCTGTCTTGGTG

>RsbHLH095

GCATTAGGTGAGAGGATTAATTGTGTTCAAGTTGGATCTCCTCTCATGAATTTTTCATGAATAATTTTCTTGTTTGGCTCTTGTGGGGTTGTGTTCAAGTTGGATCTCCTCTCGTGAATATTTTGGTGTAGATCTGGATCATTCTAGCTAATCCAACTCTGCAAAACAGGGAACCACGTTATAGCATACGTACCCAGCCGGAGTGGATTGACTGATATTGGCTAAGTAAGTCCTATTGGAAGGAAGGGCATTACAAAGTTAGAGTAAGGACAGCGTACCTCGAGGGGTTGGTACATACCTTATTATAGAGAGTTGCTTACATTGGCCTCCAAGGTAGCTCGTGCCCCCAATGTTGTCATGAAGGCTTCTTGGACTAACGTCATGCATGACGATTGTCGTGTATTGCAGTGCCACGCTGTCAGGCAACATGTGACCGAGCCACGCAGCCGAACTAAGACTAGTGGCATGCGGCCGAACCGAATAGCTTTGGGCTGATGCGTCACCAACCCAACTACACGTGATCTTCCAGGTCCAACCAGACAACTCGACCCGTCCGCAAAGCTCCTAATTGGGTGACCCGCTTGATCGCCTATGGACGCTAGCGGTCGGGTTTGACTGGGCTCCTCTTAGATTGGGCCCATCAGACAACCCAGATGGCCAACCCGCATGCCGGCCCACTACGGTTCTTATAGGACGTGGCAAAACAAGGTTGTATCTTCTGAGGGGTAGCATGATTTACTAGTTACTGTAATCTGTTGATATTTTCTACAATAATGGAACAGGAGTGGGCTAGTCCAGTTAGTTTGTAAGAAGCTAAGCTAATTATGTGGTGCCCAACTCATGACCTCAGTATAAATTAGTGTAACACACGTGCATAAAACTGTTCCAAAACATGATCAGCCAGTGAGATGTTGCCTGTCATACTGGCGCAATATATGGGCTGAGATCTTTTTGTGAAAGCGAACCTGGATTTTCAACGGTTAATTTAGGAGACACACACGTTTAGTCCCTATAAAAAAAATAGAGAAAAAGAAAAAGGATTTTAAAACAGAGAAGATTATTAGCTAGTTTTTGCATTTCGTCTACTCAAAGAAAATGGATTTTAATACGATGGAATTGTTATCTTGATGAGAGAGAGAGAGAGAGAGAGAGAGAGAGAGAGAGAGAGAGAGAGAGAGAGAGAGAGAATATAAGTTGAAGCATATAGTCTCCAAGACTTGATGGATTGGACAAGGAAAAGCTCAAGCCAAAACTAAAGGGTAAGCCTTCTCTCTCTCTCTCTCTCTCTCTCTCTCTCTCTCTCTCTCTCTCAATTTTTTATGTTTACAGAATCTGACTTTCAGAAACAATTTTTGTAAAATCTAAACTTTTGCATAGGCTACACCATAAACAATGTAGACTATAGGATTGGCATGGTACCACAGTTGTTTTGCATGAAGAATCCATGAAATCGATCTCCCTGTTTGTGGTTCTTCTTGTGGTAAATTTCGCAGGGAATCA

>RsbHLH096

AGGTGTTTTTTTGATAATTTTAAAAGGTCAGGAGCCTGTGTGTGATTTGTGTGAAAGGTCGGGTGCTATTTTAAAATTTTCCCTTTTTTTCAATCGGCAAGTTTATTAAATTACCCTTCACATCAGGAAAAATACTCAAGAGACAAGGATAAATTAGTAATTTCACATGAATAACAGACAAAAAAAAAAAGAGAAGGTGCCAATGGATAAAAAGGAATGTTAAAATCATTTCTCTACGTTACGAGTAGTAATATATACTTGTAAATATTGCAACAGTACTTATGTTATCGATATGAAACACTTGTAGTTTGGGTTGTTTATTCGCACAAATTCTGCGTCGCCACCAACCATGGAATACTTCTTTAGGATAGGTTGCAAAAGTTTCTCCCGTAAATTACTTGGTCCCAATGTATGACCTTCTTGTAATATAGTAGATAAAAAAAAGGGCCGTGATTTTACACTTCTCCATTCGATAATTGTACTTATTTTCTTGTCTTCTAATGAAAAAATACACACAAAGCTGCACAGAAAAGAAAAAAAATGGAGTTTCATTATTAAAAAAGAGTGTAAAATTATTTTCCTGAAAACAATTTTTTCCATTAAAAAAGAGAGAAAAAAGAAGTTTACGTAATAAAATTAGTTTCGATTACGTAAACAATTCAAAAAAAACCCAAATAAATAAATCCTCTCAATGTACTTGCTCCGTGGAAGATTATGAACGGCGTTCCTTTTACTTAACCCATCAACCGTGGATCCGACATAAAATCTGTTGACTGCTATTGCAGAATGATCGGACGGTAGAGAGCGAAAGTAGTTTCTACTCGGGAAAAGGACGGATTAATTATATAAAGATAGATCTCCTTGTCATCAAGCGTTAGACCCTCCGAGAAAACCTCACTCAAAAGTCACTTCCTTTAAGCTCCCGAGGTCCAGTCTCAGTCTCTCTCTCTCTCTCTCTCTCTTCCAGTGTGTGGTGGGAACCAGCGCGCGCCAGACGACAGTGCGCACACCAACAACCTCCACTGTCGAAGGAGATCACACAGCTTTAACTCTCCTGTTCTCTCTCTCTCTCTCTCTCCACACGTACTCTCTCAGTTTCTCTTCATGGGAGCCCAGTAATCCAACTCTCCTTCTCTCCGCTGGTACAGAACTTCAAATACCGAGACACTGATAAATGCGTGACTTTGTATTGTAAGTGTCCACGATCCACCGAATCTTAGCTCTGTGTTTTCGTCCCGCCGATTAAAAAAAACCTTCAAATAGCCTAAGTAATTCAACTCTCCTTCTCTGCACTAGTATAGAACTTCAAATACCGAGATATTTATAAATACGTGACTGGGTATTGTAGGTGGCACGATCCCCTGAATCTCAGCTCTGCGTTTTCGTCCCGCGGATAAAAAAAAAAAACCTCAAATAGCCAGACAGACCGTGTTTAATTTGTCAATGAGCTGGAAATGGCTGTGATGAGAAGTTCGGATCCGAATCGGGTGGAGAGTTGAG

>RsbHLH097

TATGTCTGCGGAATTATTCCTGTAGAAATTGTTGGTCAAAAAGGATGCCGTAAGAAGAAAGGAAAGTTGACCCAAACAAAGGTGAGCCAAACAGTACTTTAAACACCCTATGGTTTAAAGTGTGTGTGTATATATATATATATATATAGGCGGGATGGTTCCACGGACAACTATTTAGACACCTAAAAAAACACCCAAAATCTCAATCTCATAATTCCCAATCAAATTTTTATAATCCGAACCGTTCAATGTGTGCAGAATGTGATTTTAAGGGTGCCTACGAGAAATTAGCAAAAAAAATAACCGAAAAGTGCTTGTTTTGAGCAGTTTTTAATTGAACCGTTCGTTAAATCTAAGCTAAAAACTGCTTAGATCAAGCCCTTCCGGTCTTTTTTTTTGTTGATTTTTCGCGGGTACCCTTAAAATCACGTTCTGATCACATTGAACGGCTCGGATCATTAAAATTTGATCGGGAACTATGAGGTATTTTTTTAGGTGTCTAAATAGTTGTCCATAGAACCACCCCGATATATAGGATGATGCTATGGTGTCCTCGGTCATTTTGGGGACACTGTAATTTTTGGCATAACTTTACCATTAGGAGCATAACTAAAATCAATTACAAGTATAACAGAACCCAAATAAGGCATAACCAAGGAATATCCAAAAAACCAACCAAGGCGTAACTGAACCCAACCAAGGCATAACAATTATGCTTTGTTATGATTTTAGTTATGCATTGCTATGGTTCTGTTATGCTTGTAATGAGTTTTGGTAATATTCATAATGGTTTTGTAATGTCAAAAGTTACGGTGTCCCCAAAATGACCAGAGACACCGAAGTCATTCCCATATATATATATATATATTATGTATATAACTAGGGAGGGGGTGGTGGTGGGAGAAGTGCTGTGTTGAAGCTTCACCACTATATATATTAAGGTCCCCAAGAGAAAACAAAAAGATATACTACTACTAGTACCCAACACTTTCGAAAAGGAAAGCTAACTAGTACTCAGAAAAGTCGAAGGCAAAGAGCAAACACTCTCTCTTCCATCCCAAATCAAAGCTAGCAAAAGAGGTCTGTCTTTATTCACTTTCATTCCTCCTTCCTTCCTTCCTTCCTTCCTTTTATCCCCTTGGCAGCAGATCGAGCAGAGGAAAGAAGAATTGTTAGCCCAACTCCTGTCACCCTTTTCTCTTCTTATTCCCCCCTTTTCATCTTTTCGTGAAACAAACATGAGATGATTTAGGACTCAACCCGAAAGCCCTTCAATCCCAATCCCAATCCCTTTTGGTATATCCCGATACACACGCAGTATCCGCTCCAGAATTTTTTCAAGCCCTCACACCATCACATCGACAACTATTTTATTGAGTTGTTCATCAATTTAGGGTTGTTTGCCCCCCAGGCGATTAGCTAAGTGACCGGCCTACAGAGAGAGAGAGAGAGAGAGAGAGAGAGAGGGGAAAGGGAAAGTGAATTTTTGGGTGCATAGAG

>RsbHLH098

TTGTTGCAGCATGATTTTCAACGATTTGCTTAGCTACTTTACCGAGATTCTCATTTTACCTATGCTGATATTGTTGCTCTAAGCACATGCATTGTTTGAATCTCTATTCTAGTGTACCAATTGAACTACACATGTCAATGGGCTCTGTTTCAATTGGCATATCCTGATTCTCCTCATGTGGGATATTAAGGTTCGAACCCCGTCGTGTGCGTGTGGTATTCAGAGTTATGAACTAACATATTATCCGCTAACTCTTGCGAATGTATACCGGAGTACAATATTGGGAAAAGGAAAAAACTACACATAATTACATGCAAACGTATCATGCTACAACTACATCCAAAAATACACACCCTCTCACAAAACACAAGGGTCCCAGTGTGTGGGACCCTTGTGTTTTGTGAAAGGGTGTGTATTTGAGTGTGTATTTTTGGATGTGGTTGTAGAATTTTTGATCTATAAAAAGTGGCCGGTGAAGTGTGGATGCAAAGATCAAGAGTCAAGGGTCAGCCCCTGACCGCCTGACACATCAGTAGCCAGTAGGCCCAGTACTGGTAGCCCACCTTACTGGACGTTTAGCGTCATGGTACATAATCTCATAGAATTTGTAAAATGGTTGGCCCCACGACAATAGTAGTACTAGTTTTTTTTATATTCGGATCAATTTGCATGCTGGATGAATTTCGAGACCTTAAAGATAATAATATTACAAATTTTTCAGTAGTAAATGTCTAGTCTTCATTTGATTCGAACATGGAGAATGGATACGTATTTATTTTCTCACACCCAATCACGGAAGGCTTCATTCTTTCTAACTTTTTTGTCTAAAATTACTTTTCAGTAATTTACATTTTAGTAACATTTTGTTTTTGTTCTTTTATAGTAATTTTCGGTGTTAAGCAGTCATCTCAACGAGATAAATTAAAAAAATATAATTATATGGATTGATGCTGAAATTTGTTTTATATACGCCGACATTTTAGATAAAAAGACAGTTGTTTCATTTGATCATAATTTTAAACATTTTAGACTCATACCGTCGAGACATATAATTAATTTAAGAAATATAACGCAAATGTAACAAAAAAAATTTAGAGAAAAAGAAAAAGCACACCCAATATATATCCCGTTCCACAAACCTTCTTCAAAGTAAGGAGAGAGTAATGTTCTAGTTGGCCCACTGGGCATTGATCTCGTCGAAAAGTTATCTGCTCCATGTGGCTATCAGCGCATTAAAAAATGACCCACACAAACCATAGAGTTGACAACCGATGATTCCCCTTTCCTTCCTAAGTTTCCGTCTCTCTCTCTCTCTCCTCCCTCTCTCTCTCTCTTCTCTCTCTCACTATATAAAGGACTTGGGAAGTCGTGGGATTCTTCCAAGTTCTTCAGGGTATCAGACTTCTCACTTCGATCCCTTTCTCCTTTTAACATAAAATCTGTTGTTTTAAATTTGTGGTGCAATACATATATATACTATAGTCAATATTAATAATTGAT

>RsbHLH099

TTAGACCTCGTTCGATTTGAGATTTTGAATTTGAATTCTTAAGTAATGAGTGTAGAGAAAAATAAAGTAATGATTAAAATATGAATAATAATTAAAAAGAGATACGAAAAAAATAAGAATAATAATTAAAAAAAATAAGATAATAATTATAAATCAAAATTCAAAATCCTTAACCGAACAAGGTTTAATGAACCGAGCCTACCAGGCCCTTTGGGCATCTCTAAGGATTAAAAAAAAAAAAAAAAAACCCGAGCCCACCAGGATGGACCTGGGGAGTGTGCTGCGCAGCTCCGTGCTGCGCGGCCTCCGGCGCCGCCTTACCGGCATCGGTTCTACGATCGGAGACGTCCACTGGGTAGAGCTCGTCGAATACTACAAGTGTACCAAAAATCAACCCGGTCAAGTATCGTTAAGTGTCTCATTGGAACACATATAATTCAAAAAGAATGGGATTCAGTGGTTTTGATCCAGTGTTTCGTATCCATTACGATTCATTTTTTTTCAAGTTATATGTATTCCGATGAAGTACTTAACAATATTTGATCGGGTTGATTTTTGGTACACTTGTAGTATTCGACGAGCTCTACGCAGTGGACGTCTCCGATCGTCAAACTGATGCCGGCAAGGCGGCACCGGAGGCCACGCAGCACACTCCCTTTGTCCCACCAGGCTTTGGGCGTTTTTGCGGGGGGAAAAAAAAAAAAACCGAGCCTACCAGAGATTGAAAAAGATCCCCTCTTAAATGACATGGAGTAGTCTAGACTCTAGGTATATAGAAGGCAGAGACTAGAGAGGGGTTGATAGGACCAGTTAAATGTAGACTGTAGAGGAAGCAGAACAGAGTCACAAAAAGTTAAAACCTATTTCAATTGTAGGAGCAAACTGTTCAGTGGTTACGGTGCGCCGGCACGAGATGCGGCATGCTCTTATTCCACCTCACACGTTGTAGTGGGATCGACTCATTTTGCCTTGGATCGTGCCATAATGTATGATAGAGAAGGGGCTCGAGACACCCGGTGGCGATGCCCCAATAACATAGAATAATTTTTCAAATTTTAGATACTCTCTCCCCTAGGTTTATATAAACTTGACACTCTTCGCTTGTATCGATAGTTCTGGTGCGTCGCCACGTAATGCCGCATGTTCCTACTCCATCTCACACATGCAAAATGTGTCGTTGCATAAGATAGAATAGAAAAGGGGCCCGAGACACTCAGCGGCGATGCTCCAGTGCATAGAATAATTTCCAAATTTTAGATACTCTCTCCTCAGGCTTATATAAACCTGACACTCTTCGCTTGTCTCAGACTAAAACTTTTTCCCCCTTGACTGCTGCCTCTCTCCTTCATCATCTAGACTTCTACCATCACCTCTCTCTCTCTCTCTCTCTCTCTTTCTCTCTCTCTAGTCTCTAGTCTCTACCCTCATCTTCAATCACTTTCCTATAGCTATACAGACCTTCCAATTTCATTTCCAAAGCTTTCTCTCCAAAAAACAAAA

>RsbHLH100

CCGTCCAAAATTAAATGACCCGTTGGCTGAGAACACCATTCCAACTTTCCTCTCTCTCTCTCTCCAAACAAAAGAAACCAGACGGTCCATACCCCTCTCTCTCTGAACCATACCCCTCTCTCTCTCTTTCTATTTCTTGCTCTCACCCTTTCTCCCTGTTCGATATGATAATGGAAAAAGGAACGACTGAAACCCTAACTCCCTTATATCTCTCACCTATTGTTCCCATTGATCTCTATTTTAGATTCACCTGCGAGATCCCTCTCTCACCTCTCTCTCTCTCTCTCTCTCTCTCACTAGATTCTAGCATGTTTCGATTACTTCGGCATAGATTTGCAAAAACTAGCGATCCATTGCAAATTCTGGCGAACTTTTTGGCAAATAGAGGTAACTCCAATCGTCCATTTTGTGGTAATTGCAGTTAGGTATGATGATGTTTGATGACCTGTCAATTATAGCTGCAGCCAAAAGGGAGATGGAAATATCCATCTCCTTTCCCATCTCTGTCATTTTACATCCAGATTTGTGCCATTTTACATTCCCATTGGAGCATAAGTTTTTGCATCTGGGCTGTATTATAAGTGTAGAACTTGGTTACCATCTCCCATTGGGGATGCTCTTAGGAGGAAAAAGATCTATATAAGGGAATTTCCCCCCAAAATGGTGGATAAAAATGAGAGAAAAAATTTAATTTGGATGGAACTTGCCACTTTTCATCCAAAATATAAACGGACGGGTGTTATTAAGTGATAAAGATTGATGGGGTGTGTCTAATCGATTTTAAAGACAAAAGGGTGTAAGTGATAAAGAGTGATAGATGAGGGGGTGTAAATAATTTTTGCCAAAATAGTTTTCACATTATCAATTTTCAAAATCAATGAGATTTTCTCTTTCATTGATTGATTTGGAGAGAAAGTAGACTATAGTATTATGCACATTACCATTTCTCTCATCCCCTTTTTTTTTTCCCTTCCAATTTTCTCTAGACCCCTGTCCCCTGATAAGTTCGCCATCCAAATTCCCCGGATAAGTAGTCCGGGTGCAAAGGATAACCAAACACTGGATAAGGGGAGCCCAAATTAACTTATTCCGGCTATTACCCCTTCGCTTCTCTGCCGGCCAAACACTGGCCCAAAAATTCTAATTTTGAATTATTTCCCAGGGAGTAGTTAATAGTGGAGTGCGGTACGGTGGTGGAGGTCGCCGGCGAGGCCGATTAGAAACCAACCCCCACAACCCCCCCCCCCCCCCCCCCCCCCCCCCTAATACTGTAATTTTGAGACATCAAAGGACTTCAGTCATAACCTCTCTCTCTATAATACTGTAATTTTGAGACATCAAAAGGACTTCAGTCATAATCTTCTCTCAGGTATTGTTCAACAGATCTCTCTCTCCCATGTCTCCCTACCCAAGATTCCAATTGGGTTTCTTTCTATTTACCATAAAAATTCTATTTTGTGAAATTCGTGTAGGTTTTCAGGCACTATTTGTCATACCCAG

>RsbHLH101

CGGTAATAAGATAGCCGAACAGGGGAACAAGTTGCCCTAAAGAGTGAGGTAACAGGAATGTTCCAGAATGGTAATATTTTATAAAGGGATGAGATCCCGAGTATGTAGGATCTTGGATAGCTACCCAACGTGAGTGGGAGGTTTGGCCAGTTATGGAAGAGAGTCCTGAACTGACTCTTTTAGTGAACCGATAACGAAAGAAATGCCCAAAATATCCTGGATTCTTGTACGTGATGAGAATCCTAAGATAATAGGGATGCTTGGTGAGCAAGCATCTATGAGTCTATAAATATGAGGTAAAACTGGAGAAAAAAGGTACGCAATATTTGGCTATCTATTATTGCTTTCCTACTGATAATTAGGGTTGTTTACAAGTACGTGATACTAACTTAGGCATCGGAGGGCCTTTGCCACGAGAGGCAAAGGTGCACTCACCCCTTGTCTGTTTCGCAGATTAGGGATCCGACGAAGAATTAGGGTTCCGGTGTAAGGGCGCGTGAAGCCGTTGCAGTTTGGTATCATTCATAGCACCAATTGTTTTCATCCCCCTACATCTGATCTGATGGACGCGCTCGCGGAGATGTATGAACAGCTGTCCGCGGCAAACAAGGTGCACTTGATGAAGAAGTTGTTCAACTTCGAGATGCCTGAAGGAGGTTCGTTTATGAAGCATCTCAGCGAGTTCAATACACTAGTGGATCAGTTGATCTCTGTTGGTATTGAGTTCGATAACGAGGTTCAAGCCTTGTTGATCCTGTCTCAGTTGCCAGAGACTTGGCAGGGTACGGTTACTGCGATTAGTAATTCAGCTGGGAAGGAGAAACCCAATTGAATGATATAATCAGTTTGATTCTGACTGAGGAAGTTAGAAGGAAATCGATCGAGGGAGCGTCGGGCTTTAGCGGTTCAAGTTCCGCTCTAAGTTTTGAGCAGAGGGGCAGAAGCCAAGCCAGGGGTGGTCAGAACCAGAACAAGAGCCGGTCGAAGTCGCGCGGCAAGAAGTCCCGCGGCAAAACACAAGTGTGTGGCATTAAAGGTGAATGTTGGAACTATGGCCAGGCGGGTCACATGTCTTCAGTTTGCAAGGCTCCAAAGAAGAATAATGGTGAGCAGGAGGCCCACATCGTGCAAGATGCACTTATTCTTTCAATGAACAGTATCATGGAGTCATGGGTGATTGACTCAGGTGCGTCATTCCACGCGACTGCTAGCAAGGTTCTGAAGAACTATGTAGCAGGCGACTTCGGGCAAGTATACTTGGGCGATGATGAGCCATGTAGTATAGTGGGAATGAGCGATGTGCAGATTGAAACGCAGGGGTTTAAATGGTGTTTGAAGGATGTCAGACACGTCCCGCAGCTGAAGAGGAATCTTGTTTCAGTTGGGCAACTGGGAGCAGCGGGATATACCTCAACATTCACAGGAGACGCATGGAAGGTCTCCAATTGTACCATGGTGGTAACGCGCGGAAAAAGGTTGGTACTTTCTATTTAACTTCAC

>RsbHLH102

CTAAAAAGAAACAAATCGCAAAAATATACAGTATATACTACTACTATGTGCACAGCCCCACCAAAGGTAAAGGGACCTATGTCTTTTTTCAAAGTATTCCTTACTTTAATAATATTTAATACCTCCTTTCAACTGTTGAAACTTGAAAACTGTCGTAATTATTGGGAGTTGGGCGGCTGCTGTCCTTCAGGGACAACAGTGTGCTGTTGTCCCGGCTGAGAGGGTCTTGCTTCGACTATCGAAAACGTTCGTACAGTTCGTCGAGTTGAATAAATATGTAAAAAAATTAGCTCGATCAGATATCATTAAGTGTCTAATCAAAATCCTTTTGCCTTGAAAAGAATGGATTCGAAACACTGGATTAAATCTACTAGAACTCATTATTCGCAAGTTATATGTGTTTCGATTAGGTACTTAATAATATCCGATCGAGCTGATTTTTTATATATTTGTTCAATTCGACGAGTTCTACGAAGTGAACATTTTCAATCGTCGGAGCGGGACCCTCTCAGCCGGGACAGTACGCTGCTCAAGGGACAGCAGCCCCCAAGTCCTAATTATTGTATATATCAACTTCTACCTTTTCCTCACACTTCACATGCTAAGTCTCTGCCAGTTGCTTGAGGCATAACAAGTCCTCTCTCTCTCTCCTCCATTGTGTGAACCCAAGAAGGTTTCTTTTCTTGTACTCGTGTTCTCAATTTTTTTTTGGGTTATGAGTTCTTGAAGAAGTTTAATTTGTAATGGTGGTCAAAATTTAATCGGTAAAACCTGACGTTAGCAAAGGGTCCTTTTTTCTCTCTTGATGACCACATGCAGTTTTCAGGATATACATGAATTAAAGATGGTTTGTTAGAGCTGGTGATAGATAGTAGTAATTAACAACCAAGGAGATACATGAGAGATGATGTTTGATTCAACTACTTATGTTTTTTATAATTAAATTGACACCAATAAAAACTTCAAACCCCCCAATCCTTTGATTTTGATTGGCTCGTTTTGTAGGGTTTCCACCGCGACGCATCGATTGGATCTCGACTTGGTATGTCTTATTATTACAAAAAAAAGTTCTTCACTCGATTTTGTTGTTGGATGTTGGTATATACACTGATTGCCTTTGAAATCTCCTCTTTACTTATTATAGTCACAACTTTCACATTGATAGGGCTCATAATTAACTAATATTGATTCATTTAGGAGATTTTTCTTCATGTGCGCACCCAGTTGCGTGATCTCAGTGTTATGTTAGGATCTTTGATGCCTTCTCGATGTACCCGTTATCGAGTTATTAGTATTTTTTTTTGGCTGATCAAAAACTTTTTTCGACTTATTTAGTTGCTTCTTCTTCTTCTTCTTCTTGATATGTTAAAGATCTACCTCATGATTTATCTCATTAATTTTTCTGAAATATTCCTCAGTCGCTTGATGCATTCAATGGATAGTTTGTGTTGGGATAGCCCACCCTTATGGAACCATCACCAAGATAATGGAGAGAGCTTC

>RsbHLH103

CAAAAAACAGCAGGGGAAAGTTCTGTTAAACGGTGACGTCGCACGTTGGTTGCCGCACACGTGGCTTACCACTTACGGTGTAATCCTCTGTTGCCGTACAAATTCTGTCTTCGCCTGTGACCGTCGGATGACACCACAAGCAAAATATATATTACTACTAGGATCTGTTTGGATCTCATAAAATTTTTTTGGTCAACTTTTGTATGGATGAAATGGATTTGTTTCCTTTTTTACATATAATCTTTGAATCTTATCCTTTCAACCTATGTCAATTGATGATGGAAGAACGAAATCATAGTAAATAAACGAATTTTCAACACTTACTGCTGGTGAAAATGGAGAGATCAAACGAATAATTCTGATGTATCAAATGAATTCACGACATAGTTCTTATGACGTATGCAAATTTTCATTTTAGCAAGTCAATATATAATAATTAGAAGAATTAGAACAAGTTAGAGTTCAACGGAAATTTGGTTTATTAGTTCAAGCCCATGGATATGTTCTTTTCAGTATCCTATGTATTTTAGATTTTTTACTCTACCAAACGGTCCCTAATTTTTTTCCCCATCAACGGTGGAGAATCATGAATATCAAGATCACTCGATGCACATCAACGCTTGCGATCGAGAGTGTAGAAGCGTCTTTCATAAAATTTCCAACTGAACATGGAACGGTAATTGAAGACGACAGCAGAGGATCTTACTTAAGAATTATACAACGTTGCTAACCACATAGATATCTGTGTACAGATTTTATTGTGAGATCTATCACGGGTCTCACACAAACGATCCGAACCGTTCATTAAATGTAAAACATTTTTTCAAATGTTCTCGTGAAAAATCAGTTCAATTTAATATCTATAGATGCTCGATTCAATCATTTAAATTTTCATTCAAATTTTGGGATAATGAAAAGTTAGATGATTAGATTGAGTACATATAAATATTGGATTGAGTTAATTTTTCACAGAGACCCTTGAAAAAATATTTTATATTTAATGAACGGCTCGAATCATTTGTGTGGGATTTATAGTACATCCTACAACAAAATCTATGCACAAATTATCTGTGTAGATAGACTTATCCAGAATTATATTATTAAGATTGTGAATCATTATGTATAAATTGAAGAAAACGATAAACATACATTTTACCGACAATAAAATATGTATGCAATATTCGACTAAAATATGAAACCCTCGAATTAGCCATATGTGAATCCCACCCCATTCGAGGGCGTTGTGGATGAATATTGCGTATGTAGCATTGTTGTTCTCCGTATTATCAATCATTTTATTTCATACCTATAGTTATAGAATTGTTATATATTGGATCATTGTTTTTTTTTATCTAACAAAAACAGGACTTTATAATTGGACTTGATAAGAGAAATTAGGACTGTCCAGTCTTACCCCCAAAGAAAGAGAGAGAGAGAGAGAGAGAGAGAGAGAGGCTCTCTCCTGACACAACACCAAACCACCTGATCCTGTAGCAAACG

>RsbHLH104

TATAGTAACAGTCGCGGTTGTTAAACCGTCTTTATAGACGTAAAGAATACTAACATTTTGTGCTAGGATAAAGTGGAAGTAAGGATTCGAATTCCATCAACATCTACCATGGAAAAAGGTACATCAAGCAATGTAAATCAAGGGAGTCAAAGCCTGATATGTGTGAAAATTAATAATCCTTTATTTTATACATTCACATTGTTATAGTCTCTCTCCAAGAATTATGGAAGTTTGAGAAAGAAAAAGAGGAAGAAAAAGACAGGATGCAGAGGAAAGGAATTAAAAAGTTCGTTGACAAGCACAATCACATCTTTCTCAAGTTATTTGGGTATAGATTTTAAGTTCAATTTAATAAATAAGCATGGACTCAGCTAGTCAGTCTTTTTCTAATCACGCACAAGTTCACCTTATAAATTAATTTGATCATTGGTCCACATTACTCTCTCTCTCTCTCTCTCTCTCTAAATTAGACAAATAAAAAATATAGATTGAAGTTGAAATAGTTTATAAAACAAAAAAATGACTCAAAAGTTGTACTCCTTTTATAGTTATATTGTCTTTTCCATTTTTTTTAATTTCTATTATATCTTCTAATTTTTTGAAAAACAAAAAACAAGCAACTAATTGAGGCCAGAGGTGATGAAATAGTATACCTCTAATAGCTATAATTATAAACTCTATATCAATGACCAAAGAACCGTAATGCCTCAAAATCTTGATCTTACTCTTGAACAAAAATCAAATATCATAAACTAAACGAGCTGAGTAATAAATTTCTTGATTTAGTATGAAAATTCAACGAGCTTCGAAAGAATATTCAAACTTGTTTTTATTGCTGTAACAAATCGATTTAAAAACAAACTTTTAACGAGAGAGAAAAAACTCAAATTCCACCAGCTTGATCCGTTTATCGGCCCAACCATTAAATAAACGTCTGTTTCTTAAAAGAAGGAAAATTGATCAAGAAAGATAGCCCCTATGCTTATTCCATACGGCAAGGCAAGTCTTTTTCGTCGGAATTTACCAAAAAAAAAATCTTTTTAGTCGGTAAGTCAACATTTGGGTAATATGGTCAATTCGCTTTGTTGACGCCCATTTCGCAATAACACGCACACGCCAGGGGTATTACCGTACACAAAATTCCCAGATCTTGCCGAAATCAAGAAAATTCCGAGATCTCAGGCTTCTGTGAGTTCCGATTGGATACAGTATACCACTCACAAAGAGGAAAGTAAACATTTTCTCTCTCTCTCTCTCTCCCACTTTCTCTCTCTTCACTCCACTGCCTCCCGCCATTCCCACTCCTCCCACCTCCCTTTGCTCCTTTTTCTTTAGAGAGAGAGAGAGAGAGAGAGGAAGAAAAAGGCTAACTTTTTCTCAGAAGTTTTCAGGTGAGGTCGAGGGTTTTTTTTTTTATAACTCAGGCGTCTGGGCCAAGTTTGCACGAACACAATTAATCTTGTCAACAATCCTAGCGCAGAGAGATCGATTTGAGTTGGA

>RsbHLH105

GTGGATGATAGAAGCAACAAAGATTATTACTATATGTTACATGATTGTATGAGACCCATGTTCAAAATTTTGTAATCTGTTCGCTTGTCATGGAGGAAAATCATTTCAAAAAAGATAGTGAAGTAAAAGAATTCTAAGCATTCATTTGGTAAATAAAAATTCAAGAGAAATTTGAAAAACCGTAATACAGTCATAGCGTAGAAACTGGTGGACAAATAAAATTCATGCAAAATCTTGTATATTTTCATGAAAAAAATTGGAGACGCAGGTCCCATAATTTTTCTGATAATGGAACACATCCAAAATGCAAGAAAGTTAAATATTCCCGCACTTTTTTTCCCCTGCAACTGAACGAGGCCAATTCAATGTTGAATGTATAAAGTAGAGTTTGATTTTGTGGGCTTGTTCTACCATCTATACGTACCAAAGAATTTCATGATCCCGCACGAAGAATATCAACCATGAAAATAAGAAGTTTGTTAATTGTTTGGTTATTGATTTCGAAAGATCAAAGCCCAATAATGTCCGAATAAAACATAAAAAAAATAAAATAAAATCACAGGATCTAATGATTTGACAAAATGAAAAAATGTGAATTGTACGAAATGAACATACTCGTACATCAGATATGTTCAAAAAAAACAAAACATGCATCAGATAACTTCATCATCATAATGATTTTTTTTTTTACTTCTGGTAACCTTTGTCATAAGGTTTAAAAGTAAAATTCTAGAGAATCTTTTCTGTTTTGAGCCAAGACCAAAACAGAGATAAATCTCACTCTAGGGTGAAAAAGAGTTGTTTCCTCTGCAACTCTTCCCTCCCTTTCTCCCTCCCAGGAGATGAGAGCAAAAAAGGGATTCCGGGATGAGTTGTAAACGTAGACGAATTATGAGTTCGAAACTTGATAAACTCTTGTCTCTTGGGTCGACGTCAAGGGAGAAGTCTCCTGCAATCCATAATCCCGGAGATTTGAGGATGAGCAATAGCCAAAATGTGCGTAAGCTGCTCCGGGTCCCTTTGCTTTGACATTTATTCCCCTTCCCCTTGCGCTCTTTACGGCGAGAGAAACAGAGAGGAAAATTACTACCAGACAGACACAGTACATAATATACAAAAGAGGACAAACAAACAAGCAGAACAGTTTCCTCTCTCTGTCTATATAACTTCATCTCTACCCAACCCAAAACCTTCCCCCCTCCTCTCTCTCTCTCTCTCTCTCTCGATTGAGCTTTTTCAGCGCCAACACAATCTCCTCTCACCAACCCATAGCCCCATTCGACCCTACTCCAATCCCCATCAATACCCAATTCCCACCTCTCAATTTCCCTCCCCACCTATACCTCACCTCTGTTTTCCTCTCTCTGTTACACCTTCCCAATTTGTTTCCTTTCCTTCCCCATTCTCCATCAATTACACTCAATTTCTCCCCATTTCCCTCCTGGGTCTCCCTCTTTGTTTCTCAATTCACCCCAAATTCCCACCAATTCTCAATCTTTC

>RsbHLH106

GAAATAGTGTGGAAATCATTTCCCTAAAACAATCTACCATATACCAAATCATTATATTTGTGTGCGTCCAAAGTGTATTGGACGGTCCAGATTGAAAAAATACTATTCGCCCAAAGAGTTAAACATTCCAGCAAATAGTTTTTTAAAAATTCAAACCGTTCAATGCTGAAACGGACAGTTGTGATTGTTCACGAAGTCTCCGTGCAAAAGTTTCTCTCCTCTTAACCCTGTCTTTACTTAAATACGGAGTACTCGTCGTACTTGCCAAACTCGTCTGAACGTACTGTCTGTGATTAGGGGCAGGGTTCCAATAAAAATGGCGGTGCGGCCACTGTGTTTGTGTACTTTGATGAGATTACTTGGAACAGTAACCATGGACAAATAAAATTTACTCCGAAAATATTCCGAAAAGAATAATTAATGGTTGAAATTCACTTTAATATATGTGACTCAATCATCAAAATAATTCTTAATCACTAATTGCTTTTTTCAAAAGATGATGCTATGGTGTCCCCGGTCATTTTGGGGACTCCGTAATTTTTGGCATAACTTTACCATTAATAGTATAAGTAAAATCATTTACAAGCATAGCAGAATCCAAATAAAGTATAACCAAAGAATATCCAAAAAATCAATAAAGGCGTAACTAAACCCAACCAAGGAATAACAAATAAGTTATGTCTTGCTATGGATTTTCGTTATACTCATAATGATTTTATTATGCCAAAAGTTACGGTGTCCACAAAATGACCGGGAACACCGAAGTCATTCTCTTTTCAAAATACTTTCGAGACAAATTTTCTTTATACCTAACATTTCTCGCCACATTTGCATATGCTACGTACCTACAATTTCCATTTCGTCTTTTTGCTGTAACATCCTCCTCATTCACACTCCAGTCCAGAGAGAGAGAGAGAGAGAGAGAGAGGGAGATACACTCCAGTATATGACCTTATCCTACCACGAAAGACAAACGACGAGCAAATACAAAAACCTCAACCGCAATTCCTCATAAACAAATTTCCATTTCCCATCATTTCCTGCCAACAATATCACAGACGAGCGAGACTCGACTTATTACCAGAGAGAGAGAGAGAGAGAGAGAGAGAGAGAGAGAGAGAGCAACAGTAGCAGAAAGAGGAGAAGCAGCAGCTCTAGCTCCCATTTTTGGAATTAAAGCTATAAAATCACAGGCTTGATCACCTGCAATTCATAGTCGTGTAAGCTTGAGCCCTAAAAAACGTCTTTTTATTGTGATTGAAAATTGTGCGGAACAGATTTGATAGTTCATTGAGTTTTTTCTTTTGGTAAGTTAAGGTTGGAAAATAAATTTTGGAACATTTCTCGATTTGTGCATGTTGTGTTAATCTTTGCACGGAGGCCATACTAATCTTCTCCGTATCGTTTCAATTTTATCATCGAATCGAATGTCCCCACCGGGACGTCCTCGATCAAGTTATATTGACAAGCAAATATTCCTTTCTCTATTGCAGCTAGACA

>RsbHLH107

TTACATTATTTTTTAGTTTAAAAATGTGAATTAAATACTTATTTAGATTTTGTAAAACAATCCTTCTTATTTTTTTTGAGAAATTTTAAAATAAGTTATTATAATATAAGAAGGTTTCTGGAATCCACCCCGCTGAACAAGATGAAAAAAGTGAAGTCACAGAGAAAGGGTACAATTATAATTCGCGAGATAATAAGACAAAAAAGAAAAGGCGAAGAAGTTGAGCTTTACAAAGAAAAAACGGGGAGGGAAATAGAATCCCACCAACCAGTTATAAAAAACGAATCTATATATAGTAAATAAATAAATAAAATGGCCAATCATTCCATAGTTTTGGAGTACCACATGGTATCATTGAGCACTGCCACATATTACCCTCTACAAATAAAAGATTGCCTCAATATAATATGAGTCATTCCTTGCCTCCCAACTATCAAAGAAAAATGAATGCGTCTTTGCAGCAAAATTTGCCCCAATATAAATGGTCACGTGGTGCTCCAAAAGCGCCAAATAACTACTCTACAGAAAAATAGAATTCTACCAAACTAGCACACTCTCTCTATAAGTTGAAAATTCACGAGACCCAAACCAGGATAGCTGTTTGTTTGTTGGCCGGTGCTCTCTCCAAACTCTAATCAGATCTTCAAAATCTCTCTCAAGCCCCAAATCTTTTGTCTCCTTCTCTGCACGCTCTCACACATAATTTTCTTGGGTTTTTTTATTAAATCCCATTGTAGATTCATCAATTTCCTCAACTGGGTCTCACGTATTCAAACCCCCACACATCGAAATGCTCACGATCAAGAAGAAAATGTGGATGTTTACGTGATCTTGTCTCCAAACCAGCCAAAAGTTTTCATCTTTCACGTGATGGCAGGTATGACCACACCCACAACCACAGTAGCTCAATTCACTGCTTCTAAGCTCAAGTTTCGGCTCAGAATCCTCTCTTTCTGGTTTGCACCCACCACTCGCCCTCCTGGTTCCGATGACCCTTAGATCATCAGTACCGAACAGTATTAAGTTTCTTTCTCCAACCGATACAAGGGATAAGTAAAGTCTCAAATTGCTATTTGTAGCAGTAATATTTTTTCTTGATCTGTGAAAGTCTGTATTGTTAGAATAGTTGGATTAACTCCTTGGAAGAGGGTTGAACTCCTCACCTCCAATCTCTCAGAGCACTCCTGACGCCGTTGGGCCAAAGGTCTCTTGGCTCTTCGTAGCAGTAATATACTTACTTTCTTTTTTCTCGGTGAGCGCCTTGAAAGAGGGTTGAACTCCTGATCACCTTTCTCCGGGCCAAAGGCTGGTTGGCTCTTCGTAGCAGTAATATTTTTCTTGATCGGTGAAAGTTTGTATTGTTAGAATAGTTGGATTATCCCCTTGGAAGAGGGGTTGAACTCCTGACGTGGTATCTCCGTGCACTCCTGATGCCATTGGGCCAAAGGCCTGTTGGCTCTTCGTAGCAGTAGTATTAGAAGTGTAGTGTAATATTAAAAC

>RsbHLH108

TCTTTCAATTATTTTCATGGTTGGAAAATACTAAGTCTTTATCCCGCAAAGGCAAACCATGAACCATATACGGCCTCATCCCTTAGGTCGTACACAATCGCTCTAGAAGAAGTCAGATTTAAAACATTCAAGCCTTGACACTGCAATCTAGTCCTGACACGCCCACATCAGGCCTAGAACTGAACGACTCTCGGTTCCTAGGCCAATTTTTATTTGGGAAAATGACGGCACAGGACGTGTTTCAATAATTAAAACCCGTCAAGGACAAGCTAAGAACATTTGTTAATGTTGAAAATGTTCTTGGCGATATTAATTATCAAAACACGTCATTGACCGTCATTTTTCCTTTTTATTTTTTGTGGAAAAAACAGGAGTGGACTAAAACCAGAATATAACATTAGGAATAATGTTGGGAATCCAAGAAAAAATGTCGCATTTCTGATTTTCTATGTACGCCCAAGTAAGAGAAAACGTACATTTACTTGTTCTGAATTTGTCCTTTTTCCTATCTTTTTTTGTTGGCATCTTTGCCCCCGTTTGATAACCATATTAGCTCTTGGGATTGGATTACTAATTTCATGAGACTATTAATACTGTGTTTGATAATTCAAAATGAGTTGGGATTATTAGTCCTTAGGACTAACAAGGTGGGTATATATTAAGGGGCATTGTAGGGATGGTATTAATAGTTCAGTCTCAATCAATTCTCATTATTAATTTCTTATATCATTCCAATTCTATCATTTAATCTCATCTCAAATAAACATGATATTAATAATCTCATCATCCAATCTCATCTCAAACAAATATACTCATTATTAATCCCTTCTCATTACCAATCGCAATCCTAATTCCATTTCCTTACGAATCTCACGTATCTATCACTGTTATCAAACTCGATCTTTGTTCCTTACATATCCGTATAATTTTTTTTAACCTGGAGTAAAGGAAAAAAAACCAAAAACAAAGAGAAATCAAAAAGGAAAGGTAACGGTTGTTAATTTGAGAGTTAAGATATTGTAGTAAACTACTGTATAAAAATCAACCGCCACTGGCCACATCAAAGGACCATGTAACCGAACGTATCCTAAAAAATGTAACATTATAAACCCTAGACACAATATAAGTATTCCACGTGTCCTCCTAATTATTAGGAGTTTGTTACTCCATTCCTAGCTTTATCACACCTCATTCCTCTCAGTAACTCCCCACCACGCCCCTCTCTCTCTCTCTCTCTCTCTCTCTCTCTCTCTCTCTCTCTGATTTCTAGTAGTGTTAGTTTGTATAGTTTGATAAGGTTTTTTAACTTGAGGTCCACTGTCATTCAAACCAACAACAAATTAAAGGGACAGTAGTATCTATCTTCCAACAGGTATCTCTCTCGGACCCCTCTATCTCTCATGTTTAACTAACTGCTCGATCAATCTCTACTTATTATGACATGATCATTTGATTAAAGTTTTACTGTTGTAAATTTTGTAGGATTAATTACTTACTCCC

>RsbHLH109

AATATTAGGGGAGTGATTTTTGCACTTTCCAAATTGTTAACCGCACTCTCCTTGAAAATTCAAAAATTAATCAATATAGAATTAATTTTCGCACTTCTCAAATTGTTAACTTTATAAGTACGATTAACAATATTAGGAGTGCCAAGATCACTCACCACCTCCACCTACATTGCCATGGAAAGTATAGGCCCACAAACAAACTGAACCGTGAAAGTGAAGTGTCCTATGGTCCGGAAATTCTGAAAAAAGTTGAGCCGTTGAATTTATAAAAAGCCGAAAAGGCATATTATTCTATACGGTGACGTGCAGCTCTATCAAACGCCTTAATGATACCATATCAGATCCCTAATCCCTTTGTAGTCAAGGAACAACATTTTCCACCCTCTAAAAAATTACTCATTTAAAAAAAAAAAAAAAACGAAAATACTAAGGATACCGACAATGTACCAACGGCGTGTGGCGCGCGGTGTGCGGCTTACATTTGGATTCCACACGGATAATTCGAACTGTTGATTAATTTTAAAATAATTTTTCGAGTAATTTTGAACTCTGAATGTTGAGTTGAAATAATGGATAAACCCTTTTATACATATTCAATTAAGCTGATTTTTTGCAAGATTACTCAAAAAATTATTTTAAATTTAATGAATGGATTGGATTATTCGTGGGACCCAAAAGTGTGCCCCACACGCTATCGGTACATTGTCGGTACCCTCCCCTATCGGTACCTATAGTCGGACTCATAAACAAATTACTTCTAAAGTAGTGATTTTCACTTATTTTTTTTTATAAACGCACTCCATTTTTGGTCCTTTGGTGAAATTTTTATGTATTCTTTTTCAGTAAAAGACAAGAAGTGGAGTGCATTTATTAAAAAAAAATGTGAAAATTATTTACATTCATATATTATTATATTGAGAGCAATGAGAAATAAAAACGTACAATACTCTTTCTATCCCAAAAAAAGTAATAAGTTCGACATTCGTGTCTCTAAAAACTTTATCTATGTTTTACAATTTACAATAACTTTTACGATTTTGAAACTTTTTTCTGATAAAATTGAGGAAACAAGATTAAGACTGTACTTAAAAAAACATTATAAATAGACAGTTTTTCAGAATGCCAAAAAAGTAAGGTTGTCATCTATTTTGGACGAGAGTAGTGTATATATAATAAAAAGAAAAGTAGAATTCGATTCAAAATACCTTATCTTTTTCACGTTAATTTGTTTATTGTTTAATAAAGCTTATTCCCTTATTTAATTGTTTACCTTTGACGTTAAATCAAACATGCCAAATGGTTTTGTCGAATCATCATTCGCCAGAAAAATGTCAACCAGATTCCGGTAATTACCACCGTCCGTACAATCCTAACCGTCTATATATATACATACAACACAAACCCAAAATTATGCAATTCTACTTTCACACACAGACACATCTCAACTGGCACTACAAAGAGAGAGAGAGAGAGAGAGAGAGAGAGAGAGAGAGAGAGAGC

>RsbHLH110

AATAAAATAACTCCTCCCTTGTATATTGTCAAATACTATTATTTTCTTGCCAACAACGACATTGCAAAGGAACAATTAGTCTTCTTCTTTCTTTAGTAACTCTCAAGGCATTAGTCAACTTATCATGTCCCAGTAGTGCACAAAGCACACGTTTAGCCCGTGGAAGGACGTTGCCATGTCTTATTTCAAGAGTTTGAACTGATTTAAAATTCAATGCACGGGTTGAGGCTCGTGTCATCGTTGTTTAAGCGTTTGTGACACACACCTCCATCACGCGTTGCATGTTTGAGCAAATACTAATACTTAACAACCAATGATCACCTTTGGTTTACTTGTACACTACTAGCTCCAATTTAAAAACTCAAGCATAGAATGTTTACAGCTGCATTATTGTGGACATGGATAACCACAGATTGAGATGCTCAATTACAACCAACATAAAAAATTTGACCTTTTTTTCTTTTTTTTTTTTTTGTAAGTCAAAACTTGAAATCGATAGATGGCCTGCTCTTAAAGTCAGGAGAGAGAGAGAGAGAGAGAGAGAGAGAGAGAGAGAGAGCCCCAATCATAACATTCAAAAATCACTGGTAATATATTCTATTCTCACATAGTTAGATGAGGTGGTGATAATGTTAAAATGAACGTTCTGATTCACAATGCCAATGAGCCTTATAACTATATAAGAAATTTCTAGGGGAGTGAGATTTTCGCATTCTCCTTCTTTATAAACCTATAACCGCACTCCCATTTTTGTCTTTATTCATGCGAAGTTACAACATTACCCTTTAATGATCCTACTTTTTGACACATTTAAGGGGGAGTGCGGTTATAAAGAAAGGGAATGTGAAAATCACCCAGATCGAGTATTTATATGTCTTTTACTTAACCAATCTGTTTAATCACAAACTCATAAAAAAACTCTAACATGTTTCATTTAGAGTTACAAGTCCAAGTGATCTTGTGCACTGCTGTAATCTCTCCAAAAAAATTTCCCATTGTGACCATGAAGATAAAAAGTTTGAAATATAATCACGGAAGTCTCTATCTTAAGGAAAAGCCAAATTTATTAATCATTTTCCTGCAGGGGCGGAGTCAGGATTTTCTATAAGTGGGGTCGAATGAGTAAAAATGAATTTTACTTATATGGGTTAGGCCAAAAATGAATTTTAAAGGGAGTCAAATGAGTAAAAATTAGGTGAAAATTATACTAATCTCAAAAAAAAATTGGGAGGCAATGGGGTCAGTTGCCTCCCCTTGACTCTTAATCCCTCCGCCATTCCCCTGCAAACGTATCCGTATATGATTGGTTCTCTATTCACGGCCGGCCAGTTATCATGAGCTTAATATTCAATTCACGACCCATCAAAGTCAACTTCTTCCTAGTTTAATGCGAACCTTTTAATGTGATTCCTTTCACATGGAGAATGATGTACGTTTTTACTACTAGTATATATACGTGTTATATGTACTAGAATACGGTACAATATACGCGTATGTACA

>RsbHLH111

AATGTTTAGCTGTCGATTACCGAAATAGATGGCCAAATCGGCCGTCTTCCATGATCCCTTTGTCCCAATTTTAAATTTTTTGATGAAGGTCAAGAGGGTAATTTCACCTACTAACTAACCTAGGTAATTGCTGACTAGAGAGAGGTAGAGACCGTAGAGTGAACCATTTAAATACATACACCCAAATCTAAACTCCCAATGTGGGAGCCAGCACAACTGATGCACCGACAAAGGAAAAGGAAAAAGAGTCCTGACAAACTTTTTTCCCGAGTCCTGAGTCCTGACAAACTTTTTCCCGGCGTCAGGACTTTTCCAGCAGTCGGCGACAAATTATTGGTTAAGCCCACCCTAATAACTACACTCCTCCACACTGAACAATACCCTGCACCTATCCTGTTCTCCCACAATCCCAACTTCCATTCTCCCCTCCACCTAATCTTTCCCTCTCTCTCTCTCTCTCTCTCTAGAAACGCACGTGAGCGCAGGCAGAGGCAATGCAGGGAGGGAACATACAACTAACAAAATCAACGGCACACATTCAGCGGACTTAAATGCTGCCCAAAGCAGTCCTTTTGTGCTCCCAAAATGAACCCAGTCTCGAGCTCTAAAAATATAATCCAAATCGTTTGTTTTGTAAGGCTTATTGGTCAGATTATCATTTCAGAAAAAAGTATAATCCGAATCATTCGTTCCGTACGTAAAGTTTGTTTAGAAAATGAGCAACAGTTATGATGTTAACTAAAATGTACGTTCACAAGAACTATGTACACAGCAATCATTCGGTCACAATGACAATTCGACGAAATTTTTAATCATCCGACAGCATCAAGAAAATGAACGGCTTGAATTATTTTTACAAAGTCTATAGGGTGCACTTTTGGGGCCAAACATGTTCCCCCATCGGTTTGGAATCACGCACACTAAACTGGCTGGTAAACCAACCAGTCAAAACAACACTATCAGACAACTGTACATCCCCCCCTCAAACCCTAACTACTATTTCTCTATCCAAAACTCCCATTCTCTCTCTCTCTTAAAATTCTCTCTCTTAAAATCTTTCATTACCCCATCTGATCCACAAGCTTAACGACCCTTCTTCCAAAAATTCAGTAGAATCTTCCCACCACTCCCCTCCTCAAAGTCATCATCTACCTCTTTCTTTCACCTGCAAGTCTGCACTCTCTCTCTCTCTCTCTCTCTCTCTCTCTCAGTCATCAATCTTTGCCCCCTTCTCTCTCTGAGTACTCTCTACCTAGATCTACCTCTCTCTTTTACTCGTAGTTGATTAAATGGTTTGATTTTCAGCTCACATGCTCCATTGGAGGTTGTTTCCACCACCGGTCCCTTATATTCCCATCTCATCTAAACATATCCTTTTTTTTTTTTTGGGCCCTTTGTGGATATGTCGTGGTGGTTTTGTTTTCTAACGACAGAAGAAGGATTTGGGGGATCCAATTAAGAAAGTCGTACGTGGTGGTGATAGGCCAGCTGCGTACGTAG

>RsbHLH112

GTTCATAGAATGGGAATGCTAGCGTGCATTGAATGGAAAAACTTTGGAACAATGCTAATACAATCTAAGCCATTGTTTATGTTAAGTTGCGTGCGGTGTATTAGTAAATGGAAAAGTCTAGTGATACCGTATTTTGAACTTTATAGACTCAAGTAAACATTGGGGTCTCCATACAAGGCCCCGTTCAGTTGCCAGGAAAGTACAAGAAATGATGAAAAAAACAAATTTTCCATAACTTTTGTAGTGTTTAGTTGGCAGAAAAGTAGTGAGAAACATGTTTTTAAGTTTTCCTAGAAGATTTACTTTCTTGCAAAAGTGATCCGGCCAAAACTCTGAGATTTTACTTCACGGGAAAAGTTTTCTCCATTGTGGGACCAAAATAATTGGCGAGTAAGAAAAAAGTGAATGACGCCAATACCAAAAAAATACGAATTAGGTTCTTAGAAAGACACTTGAGATTTACGTGAGCAACAACAATTTCGATCACTATGGTGCTTTCTGAGATAATTGTTGTGAAAAAAGATTGATAGTTTATTTTTTTTGAAAGTATTTATTTTATCTTGTCATTAGTCTTCAATTTTGTTTTGTACTAACTAATTTTCAATGAGTTTTTCATCGGCAACTAATTAAGCATGGTTGTTTAATATTTATTTATTTAGTTATCATCTACTTATTTTTTAAACTTCATTTGTACGGTTGATTATCAGAGAATGTCAATTTAAATGGGAAATTTGCATCACCATTCAGAATATAGCAAATTGAATATGGAATTGCAAGTCAATTTTTTTATTTCATGTTAAGGTTTTTCATTAGATAAAATACATTTTCCGTATGTGTTAATTTTTTAAATATGATAGCCTATTATTTAGCTCTCAAAATTACTTTTATTCAAAATTAACCACAAGAATAAAAACAAAGAAGTTTCTCATAAGTGTCTTTTCCCGGCAAATGAACTACTATACTTAGTTTTCCTGCAAAAAAACATTGTCAAATGAACACTCACAGGAAAATAGATTTTTTTCCTTTATTTCACTTTACTGAACTTTTCCTGAGAATAATTTTCCGGTACAACATTCGTGGCAACTAAACGGAGCCCAAGTGTTTGTGTAGAGCCTACCACACTTATGTCTATGGAGTAAAAAACGCGGTGTCGGTAACATTTGGTGAATCCATCACAATATCTAAGGTGTTAAATATACTGTCCTTTCAAAACGAACGTCCAAAAGGCCCAAATGCACTACACGAATTCCACGTTAGCAATATCCAATTTGCATCCAAAAATTTCTCGTATCGGGAACACGTCTACCACACAAGTCCCCCAAAACCTTATCGCCCTGGCCTATTTAGTCCGGGTACCAAAAATGCCTCAGAATTACTTGTATCATTTTAGCTCAATTCATTCCTCCTCTTCCCTTCTCTCTCTAAAAAAGCTAGTGATAGTAATAAACAAGAAGCAGCTAGCTACTTATCCAAAAAAAAAAAAACAAGAAGCAGCTAACC

>RsbHLH113

GAAGGACCAAATTCAATTCTCTTATATATGTAGAGCGAGTATGGTCCCCTGAAGAGTGTACTACTAGTTACTTACTATATAAGGAAACTGGTTCTCCGTACTCACTCCTTTTCAAATTCTTCGTCCTTTATTTTTTTGAAAGTCTAAACAAATTGACTATTTCTCTTCATTTATAATATAGATTATATTAAATATAAATATTATTTGATAGATCTCAATGAATTCTTTTATTATACTGTAAAATTTTTAAAATCACCTAAAAGATTATATAGATTGTACTCCATCTGTTTCATAAAGTTTGACATTTTCAGAAATACGTGCAATTTTTAAATTGCATATATCTTTCAATTCATAATGTTTTAGGCGATTATTGGAACTTTGTTTAATAGAGCAAATTAAAATCTATCAAATAAGATCCATATTATTTATAAAAAATATTACGGATTAAAAAATATAATTAATTTTTTCATAGGGCTAGAATAGTGAAAGTGTCAAATTTTATGGGATGGAGGGGGTAAGATATAATCAATTAGAAAATGGCACGCACTCCCAAAGAGAACCAACAAATTGAAACGAACAGTATAATTAATTAATTAGGTACTCTATTTAATCTGGTACCACAGATTATAAGGGATTCCTTATTTTAATAAAATATGGATCTCCCCTTTTGTAATCAAATTTCGATGATCCGAACCGTTTAATGTATTCAGATGGTGATTTTAAGAGCTTCTTGCGAGAAAACAACAAAAAAGAAAAAGAAAAAAAGAAGAAGAACGGAAAGTACTTCATCCTAATAGTTTTCATTGAATGGTTTAATGAAAAACTGCTCAAATGAAAGGTCCGCATTTTATTAAAATAGGGGATCTCTTATGAGATACATACATACAGTGAATTTCTATTAAGGACCTTAAATACGGACCTCATATTATCTTTTTGTTCATAGAATGGGAACGCTAGCGTGCAATGAATGGAAAAACTTTGGAACAATGCTAATACAATCTAAGCCATTGTTTTTTTATGTTAAGTTGCGTGCGGTAGTTTGTATTAGTAAATAGAAAAGTCTAGTGATGCCGTATTTTGAACTTTATAAACTCAAGTGTAAAAGTGTAAATTGAGATTTCCATACAAGTGTTTGTGTAGACTGTAGAGCCTACCATACTTATGTGTATGGAGTAAAAAACACGGTGTCAGTAACATTTGGTGAATCCATTATAATATCTAAGGTGTTAAATAAACTGCCCTTTCAAAACGAATGTCCAAAAGGCCCAAATGTACTGCACGAATTCCACGTGAGCAATATCCAGTTTGCATTCAAAAATTTCTCGTATCGGGAACAAGTCTACCACACAAGTCCCCCAAACCCTTATCGCCCTGGCCTATTTAGTCCGGGTACCAAAAATGCCTCAGAATTACTAGTATCATTTTAGCTCAATTCATTCCTCCTCTTCCCTTCTCTCTCTAAGAAAGCTAGTGATAGTAATAAACAAGAAGCAGCTAACC

>RsbHLH114

TGTTAGAAATGATATTTTTGTATGCGAAGTATGCATTTTAGCGTGTGATGGGGCTATGAGAACTGTATATGAATTGTGTGTATTAGGGCTATGTGAACTGTATATTTTAGTGTGTGGTGAGGCTATGTGAACCATGTATTTTCGGTTATGTGAACTGTGTGATGGGCTATGTGGACCAATGTTCTAAAACAAGGTATTCCTCGCCGAGTACTCGCTCCAAGGTGCCTGGCCGATGCGACTAATCCCTACTCAGTGGACATTACTCGCCGACTAACGCCGAGTACTCGCTCCAGCCTCCAAGGTGCCTGGTCGAGCGAGTACGAAGTAGCAAGTTTTAGAACACTGATGTGGCTATGTGAACTGTGTATTTTAATGTGCGGAGAGGCTATGTGAACTGTGTATTTTTCAGCTCTCACAGGCAGTTCACATAGCCAATTCTCATAGAGATAGTTCTCATTGACAAGGGTATATTTTCCGAATTAATATGGGTCAGGATTATTTTAAAAAGATTGGGTAGAAATAATATATTTAGGGTTGGGATGAAACCAAAGCTAAAAAAATGGACCGCCATCTAAAAACCCCAAAATTTAAAATTATTGAGAAAAATTTGTTCTACTTCAGATATATTTTGGTTAAAAGAGTATATGATGTATGTTTGATGCTTGTTTATACATATTGATGCTTCATGCTTCTTCATATAACTATGATTCCACTCATTTAAATTAAATATTATTTATTTGTTTTTGCCTTGTCCTCTGACTTGTCAGTGTCCCCATTCTTTTAGAAATCTCTTGTCCGTGTCTGTATCCAATTGTCCATGTCTGTATCCCGTGCTGCATAGCATATAATTGGGTCTATCTTTTCCCGAGTATGCAAAGCGTACTTCTTAAATGAAATTTCACTTGATTGCTAGTATGTGCTATTGGTAATTTTTATGTTTGAACTGTGCAGGGATCTAGGCTTGGGGTTTCCCATTAATGAAGTGATAAGTTAGAAAATGGAATATACTACTGTTGAATTAGGCTGTATGGTGCTCCTTTTTTATTTGTTGATCTGTAATTGCTAGATTTGATTTCATGTTCTGAACCTTTTTAAAATTTGTCTACCAACCTATGTAATTTGATCTATCCAGTTTGTTACATTGGACGTGCTGGTATGATTGGTACTTATGCCTTATAATTTGCTTTGCAGGCTGAATATTTCCGTCAGTTGCTTAAACCTGTGACGTAGTCTAGTGTAGTAAAGGCCTTGGGAGATTGGGATTTTACTTCCCTGTAGCTGTGATCGCACATTGGAAGGCAGTAACGTCTACGAAGCCTTGGTTCAAATAGTGGTTAAATTGGACGGCCGTACTGTGATGGATACTCATTAAGTGTGCTTTTTAGTCATCTCGGTTTTCACTCCTTTAGGAACTTCCATTAAATTAAATTTGAAATCGACATTCACTGTCTCAAAAGCCACGGTTATACCTTTTGGTGAATTTACTCATTCGAAGGACCA

>RsbHLH115

ACGATTTAGATTCATGGTACCATAAATTTGAACCGTTCATTTCTCTTTCATGAAACACTTTCATGCGTCCTAGCATCCCACCACCCCAAATCTCAAAGGGCATTTTCGTTGGACCACTTAAAGTGAGTAACAGGTACAGGATATAGGGTGAGTTATCGAATGTCGGTTTGCTAGGGAATTTATTTTTTTTAGCGAAAGATCATCCTAGTTATATTAAAATAAAAAAATTACAACTACAATAACAAAGGAAATACAACAAAATAAAACATAAAACAAAGAAAAGTCGCACAAATCAGCACAGAGTCGAACCCATCAAACACACTACTGAAAGAGAGTGGACCAGATCTTCACCAAGCTGAAGCTGGTTCCTACCAAGTGTTAGTGAGAAAGTAATAAGTGTTCATTCATAAGGTCATAGGTTTCATTCTGACGGACACCAAACATTTCAAACCTTGGGACCACTGAAAGTTATGCCTGGTCGTTACCTTCAGCCCCCAGAATTAATTGAGATTTGTGCAAACTGACCCGAACATCCGATTATAAAAAATAAAAAATTGGTTCCTACCGCATACTGAAGGAACACCATGCCATTTGAATTTAAAGGCCTCTTTCTACATCCACATGACCCAGACATTGCATCAGGACCTCTCACAACGCTTTCACAGCACTGTCCTTATCCACTGATCCATCAAATACCAGTGGAACCATCCATTTCTTGATATACAGTATTCAAAGATTCGGAAGAGCATCAAATCTCAGAGTTTTTTCTGCGAGTCTACGAAGGGTTTGAACTTTGAACCGATGAATTAACCTCTCTCGGCTAAAAATCAGGTCGATCCGATATTTAATTGCTCGACGATTCAAGTCGTTTATTTAGTTGCTCGACGAATTAATCTGTCAAGTTGAAAATCAGGTCGATCCAGTATTAAAAAAGACGTGGTCGAATCTTATTTGTCTCTCGATAAATGCATGGTCAAAATATGACCATTCATTTATCTTGTGACACATACGTTTTGTTCAGAAAACCATTGAATAATCGATTCCACTGGACCAGATTTTATGCACAATGCTTAAGTACACCGAAGATACGTCGAAGCCTATAGAAAGACCGACGCAGATTGTCAGAAAAACTCACAACCATTCTTATTTCGCGTCATATAATGGATCGTCACATGAAAAGATCTTGATTCCATTAATCCACCACGCCCACGTTTAACAAGTGTGTGCTCTGTGCAAAGCAATTGTGAATTAGTACAATAATTACTCCCAGAAACATGTCAAACATGCACTAACTCTGCGCACGTGTGGGAAGAATCTCATTCCCACACGTCAAAATTGCCTCCTTTTCCCTCCAGCGTCTTCAGATTATCCCTGTCCTTCCCAATCCGGGTGGTAAATAAAACCAAGAACAACTTCAAACAACCCAACAGAATTAAACCCTCTGTCAATAATTCGTTCACTCACCGTAAACCAGTCGAATTTTCCGGCCACCGATCGGGA

>RsbHLH116

CCGTATTTATACTGTATACTTTATATATAACGTAAATGATCTCTTCTATTGATTGAAAAAAAAACTCGCAGATTTAAACAACACGGTTTTGTCTATTCTCGAGATAATGACCGTGCCAAAACAAGAGAAGAAATTGACTCGAAGGATCATACCAGTCTATTCAATTGCCAACTTTTCTCTTGCGTCATAGGGACCATGTTCCTTCTAGAATTTTGGTTTTGGGTTAATGAGTGGAAAGATAAATGAAAGAAATAATTAAAAAAAAATTTATTAAAAATCATGCGCAAAGAAAAAAGTTCAAAACTAAAAACCGAACAAGCGTAAAAATTCTCTCTCCAAATAGTAAGGGAATCCGCCCCGAGATACTCCTATGCATCAAGTGGCGGATAAAAAAAAAAATGTATCAAGTGATTTTTATTCGCAGAATTATGTGATAATCTCCAAACATGATCACGGAGTTGAAAATCTTGTCCAAAAAAAAAAAAAAAATCACGGGGTTGAAAATCAATTGGTTGGGTATAATAAAAAAAATTAGTACAGAGAAAAAGTATTTTACTCCTTTTTCAAACAAAGAATTGTTTTTTGACTTTGTTGAATATACTTGGCTTTAAGCGAACGAATTTGATTTCAATACCACCTTTAGGCTTCGGTGTTCTGGCCTTAATTTAAACTCAACGCTTAAATCTGAATTTTTTTTTCGTATTTTAGTTTTGCGCTAAACTTTTTTGGGTAATTGATTGGTCTTGACGAGAGGAATCGAAAAAGTAAAATTTTGGCACTTTTACACAAATATTTTGAGAAATAATCACTTTGACATTTTTGCCCTAAAAAATGATTATTTCTGACATTTTTGAGTTAAAAAGTGGTTACTTATCAAAATATTTGGATAAAAGTGCCAAAATTTTACTTTTTAAATTCTTCTTGTTGAGAAAAATTAAAAACCCAAAGAAATTGGCGCAAAACAAAAAAATTCAAATAAGGATAATATTTTGAAATTTAAGTTATAGGTTAAAAGAACGCAGCCAGTATTGTCTAATCTTTTCAATTTGTCCTATTCTACACCCTGAAAAGTCAGCTCAGAATAAATTCTAGTAGTCATTTCTTTGGTTCGTACACTTATTTTGATACTCCTCCGTTTCAAAATGAGTGACATTTTTGTTTCAAAATAGGGATCTTTTTAGGATTCTGTGTTATTTTCAAATGCTTATATCTTTCGATTTATGAAGTTTTATGTGATTTTAAAAATTTTGTTTAATAGAACTAATTATGATCTATCAAGCAAGATCCATATCGCATATTTTTTGACATTCATATTGAAAGATATAAACAGTTGAAAATGACACGCTTTTCCAAAAAGGCCACTTGGAACGGAGGGAGTACTATAAATAGTGACCGAAACCCATATCATCATTTTATGAAACCAACACAAGCAGTACAAGAAGCTGATAAAGGAGGAGAATTTTTGGCTGTGCAAAAGAAGCTGAGCGCTAACAACCGATC
