# Supplementary material for: Comprehensive analysis of bHLH transcription factors reveals candidate regulators of flower development and heat stress response in Rhododendron simsii
Source: BMC Plant Biol. 2025 Dec 8;26:69. doi: 10.1186/s12870-025-07868-x (PMC12797679; doi:10.1186/s12870-025-07868-x)
Supplement: Supplementary file 1 — Supplementary Material 1: Supplementary Figure S1. Chromosome localization of RsbHLH gene family. Supplementary Figure S2. Sequence logos of RsbHLH proteins. Supplementary Figure S3. The Spearman’s corrlation coefficient of RNA-seq data and RT-qPCR. Supplementary Figure S4. The expression patterns of species-sepcific RsbHLH genes in different organs and in different stages of flower development. Supplementary Table S1. Protein sequences of bHLH genes from A. thaliana, R.simsii,R.williamsianum, and R.irroratum. Supplementary Table S2. The promoters of the bHLH genes in R. simsii.Supplementary Table S3. The primers of 12 candidate genes used in this study. Supplementary Table S4. Coding sequences of RsbHLH053 and RsbHLH059 cloned for GFP fusion and subcellular localization. Supplementary Table S5. Physicochemical properties and subcellular localization of bHLH protein in R. simsii.Supplementary Table S6. The distribution of RsbHLH genes on each chromosome in R. simsii. Supplementary Table S7. The classification of the bHLH genes inArabidopsis, R. simsii, R. irroratum and R. williamsianum based on the phylogenetic analysis. Supplementary Table S8. Conservative motifs of RsbHLH protein in R. simsii. Supplementary Table S9. The promoter Cis-element functional classification of RsbHLH genes. Supplementary Table S10. Segmentally duplicated RsbHLH gene pairs. Supplementary Table S11. One-to-one orthologous relationships between R. simsii and other plants. Supplementary Table S12. The expression patterns of RsbHLH family genes. Supplementary Table S13. The expression patterns of RsbHLH family genes in different stages of flower development. Supplementary Table S14. The protein interaction network. Supplementary Table S15. The RT-qPCR data of 12 RsbHLH genes in different stages of flower development. Supplementary Table S16. The RT-qPCR data of RsbHLH genes in high temperature-treated R. simsii. [file 12870_2025_7868_MOESM1_ESM.zip › Supplementary Materials/Table S9.docx]

**Table S8 The promoter Cis-element functional classification of *RsbHLH* gene.**

| **Functional classification** | **Cris-acting elements** |
| --- | --- |
| low-temperature responsiveness | LTR |
| abscisic acid-responsiveness | ABRE |
| auxin-responsiveness | AuxRR-core |
| defense and stress responsiveness | TC-rich repeats |
| drought-inducibility | MBS |
| gibberellin-responsiveness | TATC-box, GARE-motif, P-box |
| light-responsiveness | ACE, G-Box, 3-AF1 binding site, AAAC-motif, GT1-motif, Sp1, MRE, ATC-motif, ATCT-motif, Box 4, GA-motif, GATA-motif, I-box, L-box, TCCC-motif, TCT-motif, AE-box |
| MeJA-responsiveness | CGTCA-motif, TGACG-motif |
| plant growth and development | AT-rich element, MSA-like, ARE, circadian, O2-site, CAT-box, GCN4_motif, AT-rich sequence, HD-Zip 1, AACA_motif |
| salicylic acid-responsiveness | TCA-element |
| wound-responsiveness | WUN-motif |
